# Enantioconvergent Cobalt‐Catalyzed Hydroalkylation for the Construction of Fluoro, Chloro, and Trifluoromethyl Stereogenic Centers

**DOI:** 10.1002/anie.202508637

**Published:** 2025-06-18

**Authors:** Uttam Dhawa, Xile Hu

**Affiliations:** ^1^ Laboratory of Inorganic Synthesis and Catalysis Institute of Chemical Sciences and Engineering École Polytechnique Fédérale de Lausanne (EPFL), ISIC‐LSCI Lausanne Switzerland

**Keywords:** Allenes, Asymmetric catalysis, Chlorine, Cobalt catalysis, Fluorine, Hydroalkylation

## Abstract

Organic compounds containing a chiral, halogenated aliphatic carbon center are useful in medicinal chemistry, but their enantioselective synthesis remains difficult. Here, we develop a general approach to access these compounds through cobalt‐catalyzed hydroalkylation of allenes and alkenes with dihaloamides. The reactions of allenes and terminal alkenes lead to alkylation only at the terminal position. In contrast, the reactions of internal alkenes occur via chain‐walking and alkylation at the terminal position. These reactions operate by enantioconvergent transformation of racemic secondary alkyl halides to give enantio‐enriched products. The method exhibits broad scope and high chemo, regio, and enantioselectivity. The synthetic utility of the method is demonstrated by further transformations of the products as well as the application in late‐stage functionalization of complex substrates.

## Graphical abstract

Cobalt‐hydride catalyzed enantioconvergent hydroalkylation with allenes and alkenes is achieved with high chemo‐, regio‐, and enantioselectivity. This method allows the streamlined synthesis of enantiomerically enriched C(*sp^3^*)–fluorine, –chlorine, and –trifluoromethyl stereocenters.

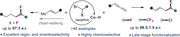

## Introduction

**Figure 1:**
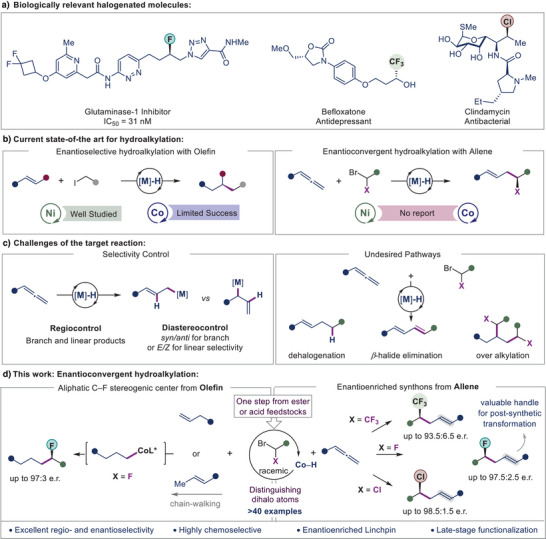
Enantioconvergent cobalt‐hydride catalyzed hydroalkylation. a) Representative examples of biologically active molecules. b) Previous advancements (left panel) and remaining challenges in hydroalkylation with allenes (right panel). c) Challenges of the target reaction: Selectivity control with allene (left panel) and undesired pathways with dihalo amides (right panel). d) Summary of the current work: Enantioconvergent hydroalkylation with olefin and allene.

The installation of halogens such as fluorine and chlorine into organic molecules is of interest to medicinal chemists because judiciously placed halogens can dramatically enhance their pharmacokinetic properties (Figure 1a).^[^ ^1^, ^2^, ^3^, ^4^ ^]^ There is a notable increase in the presence of halogens in FDA‐approved drugs in recent years.^[^ ^3^, ^5^ ^]^ Despite progress in aromatic halogenation reactions,^[^ ^6^, ^7^ ^]^ existing strategies for enantioselective construction of C(*sp^3^*)–X (X═F, Cl, CF_3_) bonds^[^ ^8^, ^9^, ^10^, ^11^, ^12^, ^13^ ^]^ remain underdeveloped, with limitations such as multistep synthesis for starting materials or the use of expensive or hazardous halogenating reagents.^[^ ^14^, ^15^ ^]^ Therefore, catalytic, enantioselective, and modular methods for creating halogenated aliphatic stereogenic centers are in good demand.^[^ ^16^, ^17^ ^]^

With their geometrically distinct structural features that exhibit diverse reactivity patterns,^[^ ^18^, ^19^ ^]^ allenes are recognized as valuable synthons for catalytic transformations.^[^ ^20^, ^21^, ^22^, ^23^ ^]^ In recent years, nickel‐hydride,^[^ ^24^, ^25^, ^26^, ^27^ ^]^ and to a lesser degree, cobalt‐hydride,^[^ ^28^, ^29^, ^30^, ^31^ ^]^ catalyzed hydroalkylation of olefins has been developed for stereoselective C(*sp^3^*)─C(*sp^3^*) bond formation (Figure 1b, left panel). However, to the best of our knowledge, nickel or cobalt‐hydride catalyzed enantioconvergent hydroalkylation of allenes remained elusive until now (Figure 1b, right panel). Compared to olefins, allenes pose a bigger challenge in regio‐control due to the presence of multiple reaction sites.^[^ ^20^ ^]^

The products, which contain F‐, Cl‐, and CF_3_‐substituted aliphatic stereogenic carbon centers, are potentially bioactive compounds or attractive linchpins^[^ ^32^ ^]^ for synthesizing structurally more complex molecules. The production of enantio‐enriched alkyl chlorine is noteworthy as there are fewer methods for the synthesis of these compounds compared to their F‐containing counterparts.^[^ ^3^ ^]^ To develop this system, we had to overcome the challenges associated with this type of hydroalkylation, such as regio‐ and diastereo‐control of reactions with allenes as well as the side‐reactions with the dihaloacetamides, such as dehalogenation,^[^ ^33^, ^34^ ^]^ β‐halide elimination,^[^ ^35^ ^]^ and overalkylation (Figure 1c).

Here we describe a cobalt hydride‐catalyzed regioselective and enantioconvergent hydroalkylation of allenes with dihaloacetamides that gives access to a large variety of enantioenriched α‐haloamides (Figure 1d). The dihalo substrates, easily accessible from ester or acid feedstocks,^[^ ^36^, ^37^ ^]^ have rarely been explored as simple precursors for stereodefined C(*sp^3^*)─F^[^ ^38^ ^]^ or C(*sp^3^*)─Cl^[^ ^39^ ^]^ motifs. Note that metal‐hydride catalyzed hydroalkylation involving a dihalo substrate and an allene had not been previously reported, even for racemic reactions.

We show that cobalt catalysis is much more efficient than nickel catalysis in these reactions. Furthermore, we show that this approach can be extended to use alkenes as reagents. In the case of internal alkenes, a chain‐walking event occurs before hydroalkylation. While nickel‐hydride catalysis is efficient for olefin isomerization,^[^ ^40^, ^41^, ^42^, ^43^ ^]^ to the best of our knowledge, there was no report of cobalt‐hydride catalysis for enantioconvergent coupling of secondary halides with olefins via a chain‐walking pathway.

## Results and Discussion

### Optimization

**Figure 2:**
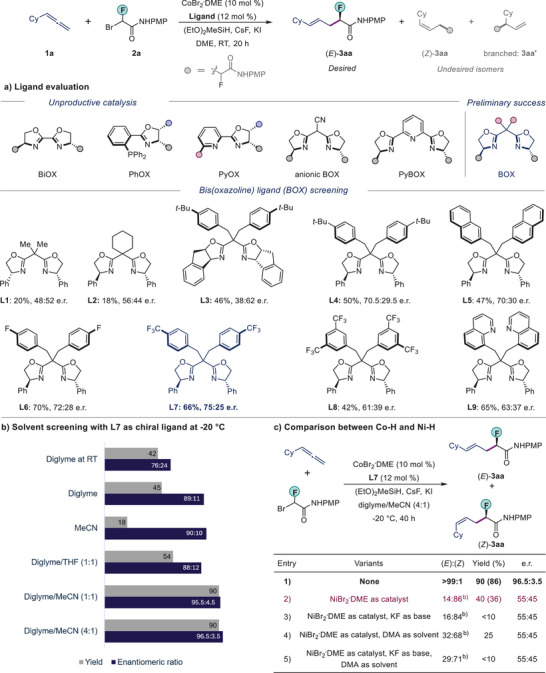
a) Optimization of ligands. Conditions: CoBr_2_.DME (10 mol %), ligand (12 mol %), **1a** (0.20 mmol), **2a** (0.10 mmol), (EtO)_2_MeSiH (0.25 mmol), CsF (0.25 mmol), KI (0.10 mmol) and DME (1.0 mL) at RT for 20 h; yields were measured by ^19^F NMR of the crude reaction mixture. The e.r. was determined using chiral HPLC analysis of the product after purification. b) Solvent screening: Conditions: CoBr_2_.DME (10 mol %), **L7** (12 mol %), **1a** (0.20 mmol), **2a** (0.10 mmol), (EtO)_2_MeSiH (0.25 mmol), CsF (0.25 mmol), KI (0.10 mmol) and solvent (1.0 mL) at RT for 20 h or −20 °C for 40 h. c) See Supporting Information for full details. ^b)^ Some other minor isomer was also detected. Isolated yield is shown in the parentheses. PMP, 4‐Methoxyphenyl; DME, 1,2‐Dimethoxyethane; THF, Tetrahydrofuran; DMA, Dimethylacetamide, RT, room temperature.

We initiated our studies on a model reaction between cyclohexyl allene **1a** and bromofluoroamide **2a**. We screened various ligand scaffolds that are commonly employed in enantioselective metal‐hydride catalysis (Figure 2a). Among ligands such as bioxazoline (BiOX), phosphino‐oxazoline (PHOX), pyridine‐oxazoline (PyOX), anionic bis(oxazoline), pyridine bis(oxazoline) (PyBOX), and bis(oxazoline) (BOX) ligands, only some BOX‐type ligands gave some promising enantioselectivity (Figure S1). When **L1** was used, the desired product **3aa** was obtained in 20% yield but without enantioselectivity. A modification at the bridge position from **L1** to **L2** did not improve the outcome of the reaction. Recently, **Indabox** ligand **L3** was shown to be effective in cobalt catalysis.^[^ ^31^ ^]^ Yet for our transformation, **L3** gave a poor performance (46% yield, 38:62 e.r.). Gratifyingly, **L4** bearing a *tert*‐butyl substituent on its backbone provided the product **3aa** in 50% yield, and with a 70.5:29.5 e.r. Ligand **L5**, with a sterically bulkier napthyl‐ substituent, did not improve the enantioselectivity. We hypothesized that rather than altering the steric environment at the backbone, we could try to alter the electronic nature of the aryl substituent to enhance both the reactivity and enantioselectivity. Encouragingly, a 4‐fluoro‐benzyl substituent (**L6**) slightly improved the enantioselectivity to 72:28 e.r. Replacing the 4‐fluoro substituent with a 4‐trifluoromethyl group (**L7**) yielded a considerable increase in enantiomeric ratio (75:25 e.r.). Further reducing the electron density in the aryl ring by introducing two trifluoromethyl groups (**L8**) was detrimental to both yield and enantioselectivity (42% yield, 61:39 e.r.). Similarly, the use of 8‐quinolinylmethyl substituted BOX ligand **L9** could not improve the efficiency of the reaction (65% yield, 63:37 e.r.). Next, we examined the effect of various solvent systems on our reaction, using **L7** as the optimal chiral ligand (Figure 2b). After an extensive screening, we found that a cosolvent system of diglyme and acetonitrile in a 4:1 ratio provided the desired product **3aa** as a single regio‐isomer (*E*) in 86% isolated yield and with 96.5:3.5 e.r. at −20 °C.

As nickel‐hydride was more extensively used for hydroalkylation, we carried out a comparison study between cobalt and nickel catalysis for this reaction (Figure 2c). When we used NiBr_2_ **^.^** DME as the metal catalyst in combination with ligand **L7**, the yield of **3aa** was reduced largely to only 40%. Moreover, the enantioselectivity was much lower, now with 55:45 e.r. Interestingly, the (*Z*)‐isomer was the major product, with an *E*:*Z* ratio of 14:86 (entry 2). We also tested some other reaction parameters, which are commonly employed in nickel catalysis (e.g., DMA as solvent and KF as base). As summarized in Figure 2c (entries 3–5), these variations gave even worse yields and selectivity. Thus, cobalt‐catalysis is much more efficient for this reaction.^[^ ^30^ ^]^

### Substrate Scope

**Scheme 1:**
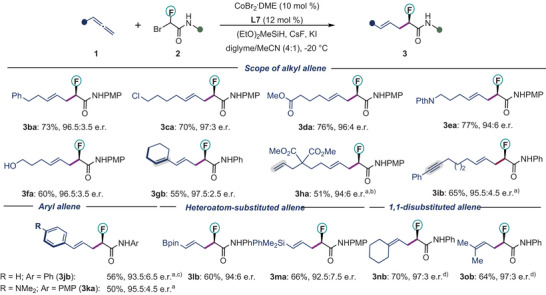
Scope of allene **1**. Conditions: CoBr_2_ **^.^** DME (10 mol %), **L7** (12 mol %), **1** (0.20 mmol), **2** (0.10 mmol), (EtO)_2_MeSiH (0.25 mmol), CsF (0.25 mmol), KI (0.10 mmol) and diglyme/MeCN (0.80:0.20 mL) at −20 °C. ^a)^0 °C, ^b)^10% other isomers were observed. ^c^diglyme/MeCN (1:1), ^d)^−35 °C. PMP, 4‐Methoxyphenyl.

With the optimal catalyst and reaction conditions in hand, we first explored the scope of the allenes for this transformation using bromofluoroamides **2** as the reaction partner (Scheme 1). A wide range of substituted allenes **1** were converted to the desired products **3** with excellent regioselectivity and high enantioselectivity. Functional groups, including halides (**1c**), enolizable ester (**1d**), and phthalimides (**1e**) were fully compatible, providing the desired products **3** in up to 77% yield and with up to 97:3 e.r. A free hydroxyl group (**1f**) was tolerated (60% yield, 96.5:3.5 e.r.). The transformation was highly chemoselective for the allene motif for the substrates containing internal (**1g**) or terminal olefins (**1h**) or aryl alkyne (**1i**). Moreover, aryl allenes (**1j–1k**) were amenable to this transformation, with high regio‐ and enantioselectivity, albeit with reduced yields (56% yield, 93.5:6.5 e.r. and 50% yield, 95.5:4.5 e.r., respectively). In addition, other heteroatom‐substituted allenes are suitable reaction partners, as exemplified by allenylboronate (**1l**) and allenylsilane (**1m**). 1,1‐Disubtituted allenes (**1n** and **1o**) also reacted efficiently to provide homoallylic fluorides **3nb** and **3ob** with 97:3 e.r. In all these cases, *E*‐alkene products were observed as the exclusive regioisomers.

**Scheme 2:**
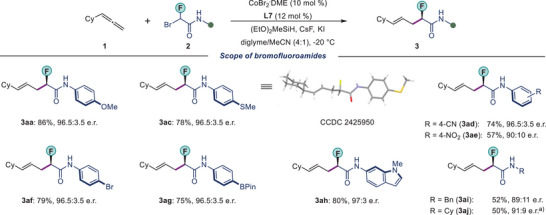
Scope of bromofluoroamides **2**. Conditions: CoBr_2_ **^.^** DME (10 mol %), **L7** (12 mol %), **1** (0.20 mmol), **2** (0.10 mmol), (EtO)_2_MeSiH (0.25 mmol), CsF (0.25 mmol), KI (0.10 mmol) and diglyme/MeCN (0.80:0.20 mL) at −20 °C. ^a)^−35 °C.

Next, we explored the scope of bromofluoroamides **2** in our standard conditions (Scheme 2). Our protocol was applicable to various substituted amides, irrespective of the electronic nature of the functional groups. A range of aromatic amides, containing either electron‐donating (‐OMe, ‐SMe) or electron‐withdrawing (‐CN, ‐NO_2_) functionalities all afforded the enantioenriched fluorinated scaffolds **3aa–3ae** in good yields (57%–86%) with high regio‐ and enantioselectivities (up to 96.5:3.5 e.r.). Bromofluoroamides bearing sensitive functional groups such as cyano (**2d**), nitro (**2e**), bromo (**2f**), and Bpin (**2g**) were viable substrates. The products contain synthetic handles for late‐stage modifications. Our protocol was not limited to the aryl amides. An *N*‐heteroaryl amide (**2h**), as well as aliphatic amides (**2i–2j**), could be successfully used in our method to provide homoallylic fluorides **3ah– 3aj**. A slight drop in enantioselectivity was observed for the aliphatic amides **2i–2j**. The absolute configuration of **3ac** was revealed by single‐crystal X‐ray diffraction analysis. The configuration of other products was inferred from the result.^[^ ^44^ ^]^

**Scheme 3:**
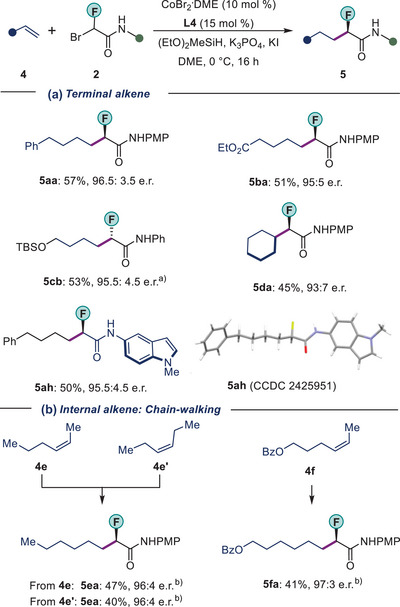
Monofluoroalkylation with unactivated alkene **4**. CoBr_2_ **^.^** DME (10 mol %), **L4** (15 mol %), **4** (0.20 mmol), **2** (0.10 mmol), (EtO)_2_MeSiH (0.25 mmol), K_3_PO_4_ (0.25 mmol), KI (0.05 mmol) and DME (1.0 mL) at 0 °C, 16 h. ^a)^ *ent*‐**L4** was used. ^b)^RT. PMP, 4‐Methoxyphenyl, DME, 1,2‐Dimethoxyethane.

Under slightly modified reaction conditions, unactivated alkenes **4** were also suitable reaction partners. The reactions with bromofluoroamides **2** provided access to enantio‐enriched fluorinated compounds **5** (Scheme 3a). Overall, good to high enantioselectivity (93:7 to 96.5:3.5 e.r.) and moderate yields (45%–57%) were obtained for the desired products (**5aa–5ah**). Notably, we could also achieve monofluoroalkylation of cycloalkene **4d** with high enantiocontrol (93:7 e.r.). The absolute configuration of **5ah** was revealed by single X‐ray diffraction analysis. The configuration of other products was inferred from the result.^[^ ^44^ ^]^

In recent years, chain‐walking based on olefin isomerization has been used as a tool for remote functionalization.^[^ ^42^, ^45^ ^]^ We found that when internal alkenes were used in our reaction system, chain‐walking occurred to yield remote C(*sp^3^*)–H alkylation at the terminal position, irrespective of the position of the double bond. These reactions gave access to monofluorinated products **5ea–5fa** (Scheme 3b). The reactions have high enantioselectivity (up to 97:3 e.r.) and complete regioselectivity.

**Scheme 4:**
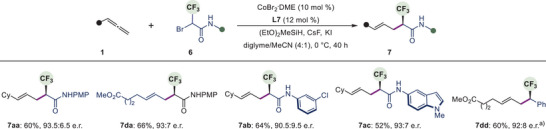
Synthesis of CF_3_‐substituted C(*sp^3^*) stereocenter. Conditions: CoBr_2_ **^.^** DME (10 mol %), **L7** (12 mol %), **1** (0.20 mmol), **6** (0.10 mmol), (EtO)_2_MeSiH (0.25 mmol), CsF (0.25 mmol), KI (0.10 mmol) and diglyme/MeCN (0.80:0.20 mL) at 0 °C, 40 h. ^a)^−20 °C. PMP, 4‐Methoxyphenyl.

The enantioselective incorporation of the trifluoromethyl group, a metabolically stable bioisostere of the methyl group,^[^ ^3^ ^]^ into molecular scaffolds is a topic of extensive research. In this context, we were pleased to find that our protocol could be applied using racemic bromotrifluoromethylacetamides **6** and allenes **1** as reaction partners. These reactions gave compounds containing enantioenriched C(*sp^3^*)–CF_3_ stereocenters (Scheme 4). Overall good yields (52%–66%) and high enantioselectivity (90.5:9.5 to 93.5:6.5 e.r.) were obtained irrespective of the substitution on allenes or amide moieties, thus providing a practical and straightforward method for asymmetric introduction of the trifluoromethyl group. It is noteworthy to mention that α‐trifluoromethyl benzyl bromide, a substrate without a carbonyl group, was also found as a viable substrate in our reaction conditions, providing the desired product **7dd** in 60% yield with 92:8 e.r.

**Scheme 5:**
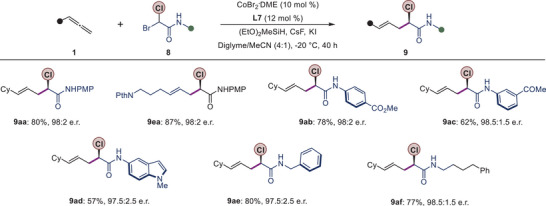
Synthesis of chiral C(*sp^3^*)–Cl center. Conditions: CoBr_2_ **^.^** DME (10 mol %), **L7** (12 mol %), **1** (0.20 mmol), **8** (0.10 mmol), (EtO)_2_MeSiH (0.25 mmol), CsF (0.25 mmol), KI (0.10 mmol) and diglyme/MeCN (0.80:0.20 mL) at −20 °C, 40 h. PMP, 4‐Methoxyphenyl.

Despite the significant synthetic utility of chiral alkyl chlorides,^[^ ^32^ ^]^ methods to construct chlorinated chiral carbon centers are limited. Success has primarily been achieved using electrophilic chlorination reagents.^[^ ^3^ ^]^ We found that our method is applicable for the reactions of bromochloroacetamides **8** with allene **1**. These reactions afforded the desired chlorinated products **9** in high yields (Scheme 5). Excellent enantioselectivities were obtained for the majority of the substrates (up to 98.5:1.5 e.r.). In addition to cyclohexyl allene **1a**, an unbranched allene bearing pthalimide group **1e** was also a suitable reaction partner, providing **9ea** in 87% yield and with 98:2 e.r. The method proved viable for amides containing various sensitive substituents such as ester **8b** and ketone **8c** to provide **9ab** (78% yield, 98:2 e.r.) and **9ac** (62%, 98.5:1.5 e.r.) respectively. High enantioselectivity was achieved regardless of whether *N*‐heteroaryl **8d** or aliphatic amides **8e–8f** were employed (up to 98.5:1.5 e.r.). In contrast, when halothane, a substrate lacking a carbonyl functionality, was used, the desired product **S10** was obtained in 36% yield with an enantiomeric ratio of 77:23 (Figure S2).

### Synthetic Application

**Scheme 6:**
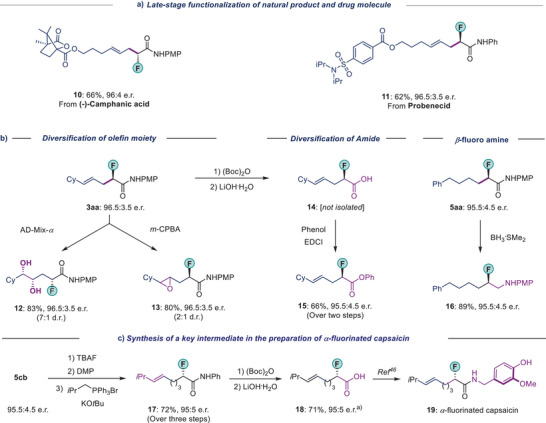
Synthetic Application. a) Late‐stage functionalization, b) Diversifications of olefin and amide moieties, c) Synthesis of a bioactive molecule. See Supporting Information for full details. ^a)^There was determined after performing a subsequent transformation.

To demonstrate the potential of our methodology, we applied our protocol to the late‐stage functionalization of a natural product and a drug molecule (Scheme 6a). Allenes derived from camphanic acid and probenecid (a drug used for gout treatment) afforded the homoallylic fluorides **10** and **11** in good yields and with high enantioselectivity (66%, 96:4 e.r. and 62%, 96.5:3.5 e.r., respectively).

We further showed that product **3**, being enantioenriched alkyl fluorides with both an alkene and an amide moiety, could act as linchpins for the synthesis of other valuable chiral compounds (Scheme 6b). For instance, asymmetric dihydroxylation of alkene in **3aa** proceeded efficiently to provide the desired diol **12** with multiple stereocenters with good diastereoselectivity. Epoxidation of alkene in **3aa** afforded the corresponding epoxide **13** in 80% yield with 96.5:3.5 e.r. We could also show that the amide motif in **3aa** could be converted to an acid in **14**, which was subsequently converted into the corresponding ester **15** in a good yield. In addition, reduction of **5aa** with borane dimethylsulfide yielded the chiral β‐fluoro amine **16** in 89% yield. Overall, the enantiopurity of the compounds was well preserved throughout these downstream transformations.

The utility of our method was also demonstrated through the synthesis of a key intermediate to the synthesis of α‐fluorinated capsaicin **19**. Capsaicin is used in pain‐relief medications, and the α‐fluorinated analogue (**19**) has been tested.^[^ ^46^ ^]^ Starting from the hydroalkylation product **5cb**, the olefinated intermediate **17** was obtained without any loss in enantiomeric purity (Scheme 6c). A mild hydrolysis of **17** yielded the free acid **18**, which could be converted into **19**, in one step according to a previous report.^[^ ^46^ ^]^

### Mechanistic Studies

**Scheme 7:**
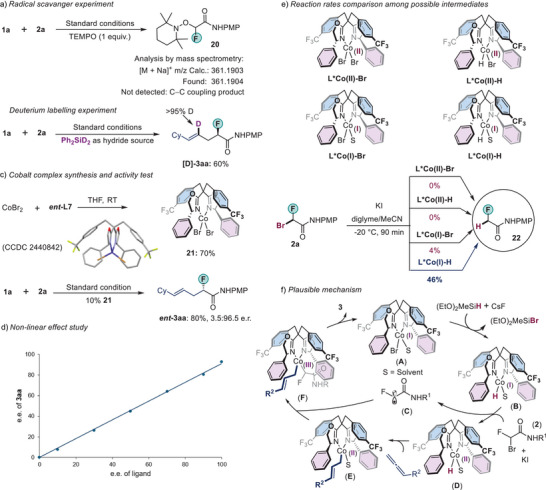
Mechanistic studies. a) Radical scavenger experiment with TEMPO was performed under the standard reaction conditions. b) Deuterium labelling experiment. c) Catalytic activity of cobalt complex. d) Nonlinear effect study. e) Kinetic probes to compare reaction rates among cobalt species. f) Outline of a plausible mechanism. See Supporting Information for full details of a)–e). TEMPO, (2,2,6,6‐Tetramethylpiperidin‐1‐yl)oxyl. THF, Tetrahydrofuran. PMP, 4‐Methoxyphenyl.

To shed some light on the mechanism, a series of control experiments were carried out (Scheme 7). When we added one equivalent of a radical scavenger, (2,2,6,6‐Tetramethylpiperidin‐1‐yl)oxyl (TEMPO) in the reaction mixture, the product **3aa** was not observed (Scheme 7a). Analysis of the crude reaction mixture by mass spectrometry revealed the presence of an alkyl‐TEMPO adduct **20** originated from bromofluoroamide **2a**. This result suggests the formation of an alkyl radical from an alkyl halide electrophile. Next, we conducted deuterium labelling experiments with Ph_2_SiD_2_ as the deuteride source. We exclusively observed deuterium incorporation at the *γ*‐position of the fluorine atom. No H/D exchange was observed among the three carbon atoms of the allene moiety (Scheme 7b). This result suggests that the insertion of cobalt‐hydride into the allene yielded only the terminal cobalt‐alkyl species. We were able to prepare the cobalt complex **21** from the reaction of CoBr_2_ with *ent*‐**L7** in a 70% yield (Scheme 7c). This complex was characterized by X‐ray diffraction analysis. (Due to the poor single‐crystallinity of the samples, the resolution of the structure was low, but the connectivity of the compound could be established).^[^ ^44^ ^]^ When we employed the cobalt complex **21** as the catalyst in the standard reaction conditions, we obtained the desired product ***ent‐*3aa** in a comparable yield and with similar enantioselectivity. This result suggests that complex **21** is in the catalytic cycle or could enter the catalytic cycle. We did not observe a nonlinear effect, indicating that the enantiodetermining step involves a species with a cobalt‐to‐ligand ratio of 1:1 (Scheme 7d). To probe the active cobalt species responsible for the activation of alkyl halide, we performed a study to examine the reactivity of a potential cobalt intermediate towards the alkyl halide **2a** (Scheme 7e). The cobalt(II)‐H, cobalt(I)‐Br, and cobalt(I)‐H species were made in situ from complex **21** (Supporting Information). Neither cobalt(II)–H nor cobalt(II)–Br species seemed to react with **2a**. In contrast, both cobalt(I)–Br and cobalt(I)–H species reacted with **2a**. However, the reactivity of the cobalt(I)‐H species is much higher: in the same period (90 min) and under the same conditions, the reaction of in situ generated cobalt(I)–H species with **2a** gave the debrominated product **22** in 46% yield, compared to the 4% yield by the cobalt(I)–Br species. These findings suggest that cobalt(I)‐hydride is the main active species responsible for the activation of the secondary dihaloamide.

Based on these studies and the literature precedent^[^ ^30^ ^]^ including nickel catalysis,^[^ ^47^ ^]^ we propose a catalytic cycle (Scheme 7f). The mechanism involves the formation of a Co(I)‐H (**B**) from the reaction of a Co(I) halide species (**A**) with silane in the presence of CsF. The activation of an alkyl bromide (**2**) by the Co(I)‐H gives a Co(II)‐H (**D**) and an alkyl radical (**C**). The presence of KI likely promotes this reaction due to an in situ Br/I exchange. Insertion of the Co(II)‐H into the allenes gives a Co(II)‐alkyl species (**E**), which traps the alkyl radical to furnish a cobalt(III) species (**F**). Reductive elimination of species **F** gives the hydroalkylation product **3**. Similar to previous examples of nickel catalysis,^[^ ^48^, ^49^, ^50^, ^51^ ^]^ the enantio‐determining step is the reductive elimination step.

## Conclusion

In summary, we have developed a general method for cobalt‐hydride catalyzed enantioconvergent hydroalkylation using dihaloamides as reaction partners. We first identified ligands and conditions for the reactions with allenes, which were elusive substrates in hydroalkylation reactions. We then expanded our protocols to include alkenes as substrates. These reactions provide a streamlined access to enantiomerically‐enriched compounds containing C(*sp^3^*)‐fluorine, ‐chlorine, and ‐trifluoromethyl stereocenters. Our reactions have broad scope and high functional group tolerance. They are highly regio‐ and enantioselective. The reaction protocol is suitable for late‐stage functionalization of complex substrates, and the products can serve as valuable synthons for downstream transformations. The unique efficiency of cobalt catalysis in these transformations compared to nickel catalysis suggests the potential for the former for further developments.

## Supporting Information

Experimental procedures and compound characterization data that support the findings of this study are available in the online version of this paper in the accompanying Supporting Information. Further data are available online in Zenodo: https://doi.org/10.5281/zenodo.15571047. The authors have cited additional references within the Supporting Information.^52–58^

## Conflict of Interests

The authors declare no conflict of interest.

## Supporting information



Supporting Information

Supporting Information

## Data Availability

The data that support the findings of this study are available in the Supporting Information of this article.

## References

[1] S. Purser , P. R. Moore , S. Swallow , V. Gouverneur , Chem. Soc. Rev. 2008, 37, 320–330. 18197348 10.1039/b610213c

[2] J. Han , A. M. Remete , L. S. Dobson , L. Kiss , K. Izawa , H. Moriwaki , V. A. Soloshonok , D. O'Hagan , J. Fluor. Chem. 2020, 239, 109639.

[3] D. Chiodi , Y. Ishihara , J. Med. Chem. 2023, 66, 5305–5331. 37014977 10.1021/acs.jmedchem.2c02015

[4] W.‐Y. Fang , L. Ravindar , K. P. Rakesh , H. M. Manukumar , C. S. Shantharam , N. S. Alharbi , H.‐L. Qin , Eur. J. Med. Chem. 2019, 173, 117–153. 30995567 10.1016/j.ejmech.2019.03.063 PMC7111421

[5] N. A. McGrath , M. Brichacek , J. T. Njardarson , J. Chem. Educ. 2010, 87, 1348–1349.

[6] W. Wang , S. Song , N. Jiao , Acc. Chem. Res. 2024, 57, 3161–3181. 39303309 10.1021/acs.accounts.4c00501

[7] R. A. Rodriguez , C.‐M. Pan , Y. Yabe , Y. Kawamata , M. D. Eastgate , P. S. Baran , J. Am. Chem. Soc. 2014, 136, 6908–6911. 24758725 10.1021/ja5031744 PMC4333596

[8] Y. Liang , G. C. Fu , J. Am. Chem. Soc. 2015, 137, 9523–9526. 26203662 10.1021/jacs.5b04725 PMC4610818

[9] C. Sandford , R. Rasappan , V. K. Aggarwal , J. Am. Chem. Soc. 2015, 137, 10100–10103. 26244235 10.1021/jacs.5b05848

[10] X. Jiang , M. Gandelman , J. Am. Chem. Soc. 2015, 137, 2542–2547. 25630234 10.1021/jacs.5b00473

[11] R. K. Quinn , Z. A. Könst , S. E. Michalak , Y. Schmidt , A. R. Szklarski , A. R. Flores , S. Nam , D. A. Horne , C. D. Vanderwal , E. J. Alexanian , J. Am. Chem. Soc. 2016, 138, 696–702. 26694767 10.1021/jacs.5b12308 PMC5562432

[12] F. Chen , Q. Zhang , Y. Li , Z.‐X. Yu , L. Chu , J. Am. Chem. Soc. 2024, 146, 11418–11431.

[13] C. Lee , M. Kim , S. Han , D. Kim , S. Hong , J. Am. Chem. Soc. 2024, 146, 9375–9384. 38512796 10.1021/jacs.4c01548

[14] W. Liu , J. T. Groves , Acc. Chem. Res. 2015, 48, 1727–1735. 26042637 10.1021/acs.accounts.5b00062

[15] K. Hirano , Synthesis 2022, 54, 3708–3718.

[16] R. Britton , V. Gouverneur , J.‐H. Lin , M. Meanwell , C. Ni , G. Pupo , J.‐C. Xiao , J. Hu , Nat. Rev. Methods Primer 2021, 1, 47.

[17] P. H. S. Paioti , S. A. Gonsales , S. Xu , A. Nikbakht , D. C. Fager , Q. Liu , A. H. Hoveyda , Angew. Chem. Int. Ed. 2022, 61, e202208742. 10.1002/anie.202208742 36017964

[18] S. Yu , S. Ma , Angew. Chem. Int. Ed. 2012, 51, 3074–3112. 10.1002/anie.201101460 22271630

[19] L. Liu , R. M. Ward , J. M. Schomaker , Chem. Rev. 2019, 119, 12422–12490. 31833759 10.1021/acs.chemrev.9b00312 PMC7382613

[20] R. Y. Liu , Y. Yang , S. L. Buchwald , Angew. Chem. Int. Ed. 2016, 55, 14077–14080. 10.1002/anie.201608446 PMC5198719 27723269

[21] L. Lavrencic , U. Dhawa , A. Blumenstein , X. Hu , ChemSusChem 2023, 16, e202300703. 37432646 10.1002/cssc.202300703

[22] Z.‐X. Wang , Y. Xu , R. Gilmour , Nat. Commun. 2024, 15, 5770. 38982181 10.1038/s41467-024-50227-x PMC11233658

[23] T.‐D. Tan , K. Z. Tee , X. Luo , P.‐C. Qian , X. Zhang , M. J. Koh , Nat. Synth. 2025, 4, 116–123.

[24] Y. He , J. Chen , X. Jiang , S. Zhu , Chin. J. Chem. 2022, 40, 651–661.

[25] Z. Zhang , S. Bera , C. Fan , X. Hu , J. Am. Chem. Soc. 2022, 144, 7015–7029. 35413202 10.1021/jacs.1c13482

[26] Z. Wang , H. Yin , G. C. Fu , Nature 2018, 563, 379–383. 30337711 10.1038/s41586-018-0669-y PMC6296363

[27] S. Bera , R. Mao , X. Hu , Nat. Chem. 2021, 13, 270–277. 33380741 10.1038/s41557-020-00576-z PMC7610379

[28] Y. Li , X. Lu , Y. Fu , CCS Chem. 2024, 6, 1130–1156.

[29] Y. Li , W. Nie , Z. Chang , J.‐W. Wang , X. Lu , Y. Fu , Nat. Catal. 2021, 4, 901–911.

[30] Z.‐L. Zhang , Z. Li , Y.‐T. Xu , L. Yu , J. Kuang , Y. Li , J.‐W. Wang , C. Tian , X. Lu , Y. Fu , Angew. Chem. Int. Ed. 2023, 62, e202306381. 10.1002/anie.202306381 37254230

[31] Y. Li , D. Liu , X. Hu , J.‐Y. Zhang , Q.‐W. Zhu , B. Men , G.‐W. Gao , P.‐W. Chen , Y.‐Z. Tong , Z. Chang , Z. Li , X. Lu , Y. Fu , Nat. Synth. 2024, 3, 1134–1144.

[32] Y. Liu , S. G. Bender , D. Sorigue , D. J. Diaz , A. D. Ellington , G. Mann , S. Allmendinger , T. K. Hyster , J. Am. Chem. Soc. 2024, 146, 7191–7197. 38442365 10.1021/jacs.4c00927 PMC11622607

[33] R. Doi , M. Yasuda , N. Kajita , K. Koh , S. Ogoshi , J. Am. Chem. Soc. 2023, 145, 11449–11456. 37141012 10.1021/jacs.3c03471

[34] C. Douvris , O. V. Ozerov , Science 2008, 321, 1188–1190. 18755971 10.1126/science.1159979

[35] T. Fujita , K. Fuchibe , J. Ichikawa , Angew. Chem. Int. Ed. 2019, 58, 390–402. 10.1002/anie.201805292 29953707

[36] F. Liang , N. Chen , K. Cheng , Q. Wang , Org. Lett. 2023, 25, 8168–8172. 37922199 10.1021/acs.orglett.3c03461

[37] D. Yamane , R. Tetsukawa , N. Zenmyo , K. Tabata , Y. Yoshida , N. Matsunaga , N. Shindo , A. Ojida , J. Med. Chem. 2023, 66, 9130–9146. 37393576 10.1021/acs.jmedchem.3c00737

[38] U. Dhawa , L. Lavrencic , X. Hu , ACS Cent. Sci. 2024, 10, 1657–1666. 39220696 10.1021/acscentsci.4c00819 PMC11363326

[39] A. Fantinati , V. Zanirato , P. Marchetti , C. Trapella , ChemistryOpen 2020, 9, 100–170. 32025460 10.1002/open.201900220 PMC6996577

[40] D. Janssen‐Müller , B. Sahoo , S.‐Z. Sun , R. Martin , Isr. J. Chem. 2020, 60, 195–206.

[41] Y. Wang , Y. He , S. Zhu , Acc. Chem. Res. 2022, 55, 3519–3536. 36350093 10.1021/acs.accounts.2c00628

[42] C. Romano , R. Martin , Nat. Rev. Chem. 2024, 8, 833–850. 39354168 10.1038/s41570-024-00649-4

[43] F. Zhou , Y. Zhang , X. Xu , S. Zhu , Angew. Chem. Int. Ed. 2019, 58, 1754–1758. 10.1002/anie.201813222 30548518

[44] Crystallographic data for **3ac**, **5ah**, and **21** have been deposited at the Cambridge Crystallographic Data Centre, under deposition numbers CCDC 2425950 (**3ac**), CCDC 2425951 (**5ah**), and CCDC 2440842 (**21**).

[45] H. Sommer , F. Juliá‐Hernández , R. Martin , I. Marek , ACS Cent. Sci. 2018, 4, 153–165. 29532015 10.1021/acscentsci.8b00005 PMC5833012

[46] M. Winkler , T. Moraux , H. A. Khairy , R. H. Scott , A. M. Z. Slawin , D. O'Hagan , ChemBioChem 2009, 10, 823–828. 19267374 10.1002/cbic.200800709

[47] C. Fan , U. Dhawa , D. Qian , D. Sakic , J. Morel , X. Hu , Angew. Chem. Int. Ed. 2024, 63, e202406767. 10.1002/anie.202406767 38682392

[48] O. Gutierrez , J. C. Tellis , D. N. Primer , G. A. Molander , M. C. Kozlowski , J. Am. Chem. Soc. 2015, 137, 4896–4899. 25836634 10.1021/ja513079r PMC4576934

[49] X. Lin , J. Sun , Y. Xi , D. Lin , Organometallics 2011, 30, 3284–3292.

[50] C. L. Wagner , G. Herrera , Q. Lin , C. T. Hu , T. Diao , J. Am. Chem. Soc. 2021, 143, 5295–5300. 33792294 10.1021/jacs.1c00440 PMC8851433

[51] J. Diccianni , Q. Lin , T. Dia , Acc. Chem. Res. 2020, 53, 906–919. 32237734 10.1021/acs.accounts.0c00032 PMC7958188

