# Supplementary material for: Enantioconvergent Cobalt‐Catalyzed Hydroalkylation for the Construction of Fluoro, Chloro, and Trifluoromethyl Stereogenic Centers
Source: Angew Chem Int Ed Engl. 2025 Jun 18;64(32):e202508637. doi: 10.1002/anie.202508637 (PMC12322659; doi:10.1002/anie.202508637)
Supplement: Supplementary file 1 — Supporting Information [file ANIE-64-e202508637-s001.pdf]

Supporting Information

Enantioconvergent Cobalt-Catalyzed Hydroalkylation for the Construction of Fluoro, Chloro and Trifluoromethyl Stereogenic Centers

Uttam Dhawa and Xile Hu*

Laboratory of Inorganic Synthesis and Catalysis, Institute of Chemical Sciences and Engineering,

École Polytechnique Fédérale de Lausanne (EPFL), ISIC-LSCI, Lausanne, Switzerland

Correspondence to: xile.hu@epfl.ch

Table of Contents

1. Instrumentation and Chemicals S3

2. General Procedure for Enantioconvergent Hydroalkylation S5

3. Reaction Optimization S6

4. Synthesis of Starting Materials S8

5. Product Characterization Data S17

6. Synthetic Application S83

7. Mechanistic Experiments S107

8. Crystallography Details S116

9. NMR Spectra S125

10. References S258

1. Instrumentation and Chemicals:

All reactions for enantioconvergent hydroalkylation were set up in 16 mL Teflon-screw capped vials (unless otherwise noted) under an inert nitrogen (N_2) atmosphere using glove-box techniques. Solvents were bought from the commercial sources and transferred to the glovebox without exposure to air.

NMR: ^1H, ^{13}C and ^{19}F NMR spectra were recorded on a Bruker Advance 400 Spectrometer. ^1H and $^{13}C\{1H\}$ chemical shifts were referenced internally to residual solvent peaks relative to TMS (δ = 0 ppm) at 299 K.

TLC: Merck silica gel 60 F 254 plates; detection with UV light or by dipping into a solution of $KMnO_4$ (1.5 g in 400 mL H_2O, 5.0 g $NaHCO_3$), followed by heating.

Flash column chromatography (FC): Flash column chromatography was performed using silica gel (Silicycle, ultra-pure grade). Preparative Thin Layer Chromatography (PTLC) was performed using glass plates from Merck KGaA, Darmstadt, Germany. The eluents for column chromatography and PTLC were presented as ratios of solvent volumes.

Automated flash column chromatography : Automated flash column chromatography was performed using Biotage® Isolera™ One Flash Chromatography Instrument on Biotage® Sfär Silica D Duo 60 µm columns (10 or 25 g with flowrates of 40 and 80 mL/min, respectively) with detection at 245 and 280 nm.

Preparative Thin Layer Chromatography (pTLC): Preparative thin layer chromatography was performed using silica-coated glass plates from Merck KGaA, Darmstadt, Germany. The eluents are presented as ratios of solvent volumes.

GC and GC-MS: All GC analyses were performed on a Perkin-Elmer Clarus 400 GC system with an FID detector. All GC-MS analyses were performed on an Agilent Technologies 7890A GC system equipped with a 5975C MS detector.

HPLC: High-performance liquid chromatography was performed on an Agilent HPLC instrument. The utilized column (with a chiral stationary phase), eluent mixture, and retention times used for the determination of enantiomeric ratio (e.r.) are given below in the details of relevant experiments.

Optical rotations were measured on a Polartronic M polarimeter using a 0.5 cm cell with a Na 589 nm filter.

High-resolution mass spectra (HRMS): by electrospray ionization (ESI), atmospheric pressure chemical ionization (APCI) and atmospheric pressure photoionization (APPI) method were performed at the EPFL ISIC Mass Spectroscopy Service.

IR spectra were recorded on a Bruker Vertex 80 FT-IR spectrometer.

All reagents were either prepared according to reported methods or purchased from Sigma Aldrich, TCI, Acros Organics, Alfa Aesar, Fluorochem, Enamine and ABCR. Anhydrous $CoBr_2{\cdot}DME$ from Strem Chemicals, anhydrous diglyme from Thermoscientific and Sigma Aldrich, anhydrous MeCN and ethylene glycol dimethyl ether from Thermoscientific, CsF from Sigma Aldrich, KI from Alfa Aesar, $(EtO)_2MeSiH$ from Acros Organics were used.
Racemic compounds were prepared using a racemic ligand.

2. General Procedure for Enantioconvergent Hydroalkylation

2.1 General Procedure 1: Enantioconvergent Hydroalkylation with Allene

To an oven-dried 16 mL Teflon-screw capped vial was added (4*S*,4'*S*)-2,2'-(1,3-bis(4-(trifluoromethyl)phenyl)propane-2,2-diyl)bis(4-phenyl-4,5-dihydrooxazole) **L7** (7.47 mg, 0.012 mmol, 0.12 equiv.). The vial was introduced in a nitrogen filled glovebox. A magnetic stir bar (6 x 15 mm), cobalt(II) dibromo(1,2-dimethoxyethane) (3.09 mg, 0.01 mmol, 0.10 equiv.) and anhydrous diglyme/MeCN (0.8 mL/0.2 mL) were added and the mixture was stirred for 15-30 min at room temperature until it became a clear blue solution. Then racemic electrophile (0.10 mmol, 1.0 equiv.), allene (0.20 mmol, 2.0 equiv.), anhydrous cesium fluoride (38 mg, 0.25 mmol, 2.5 equiv.) and potassium iodide (16.6 mg, 0.10 mmol, 1.0 equiv.) were added to it. Then methyldiethoxysilane (40.5 µL, 0.25 mmol, 2.5 equiv.) was added dropwise to it. The vial was then sealed with airtight electrical tapes, removed from the glove box immediately, and stirred for 40 h at -20 °C, maintaining 600 rpm. After that, the reaction was diluted with EtOAc, transferred into a one-necked flask, and the solvents were removed under *vacuum*. The crude mixture was purified by automated flash column chromatography to yield the products.

2.1 General Procedure 2: Enantioconvergent Hydroalkylation with Olefin

To an oven-dried 16 mL Teflon-screw capped vial was added (4*S*,4'*S*)-2,2'-(1,3-bis(4-(*tert*-butyl)phenyl)propane-2,2-diyl)bis(4-phenyl-4,5-dihydrooxazole) **L4** (8.98 mg, 0.015 mmol, 0.15 equiv.). The vial was introduced in a nitrogen filled glovebox. A magnetic stir bar (6 x 15 mm), cobalt(II) dibromo(1,2-dimethoxyethane) (3.09 mg, 0.01 mmol, 0.10 equiv.) and anhydrous DME (1.0 mL) were added, and the mixture was stirred for 15-30 min at room temperature until it became a clear blue solution. Then racemic electrophile (0.10 mmol, 1.0 equiv.), olefin (0.20 mmol, 2.0 equiv.), anhydrous potassium phosphate tribasic (53.1 mg, 0.25 mmol, 2.5 equiv.), potassium iodide (8.3 mg, 0.05 mmol, 0.50 equiv.) were added to it. Then methyldiethoxysilane (40.5 µL, 0.25 mmol, 2.5 equiv.) was added dropwise to it. The vial was then sealed with airtight electrical tapes, removed from the glove box immediately, and stirred for 16 h at 0 °C, maintaining 600 rpm. Afterward, the general procedure (**GP1**) for analysis of the crude reaction mixture and product isolation was followed.

4. Reaction Optimization

Figure S1. Screening of Ligands

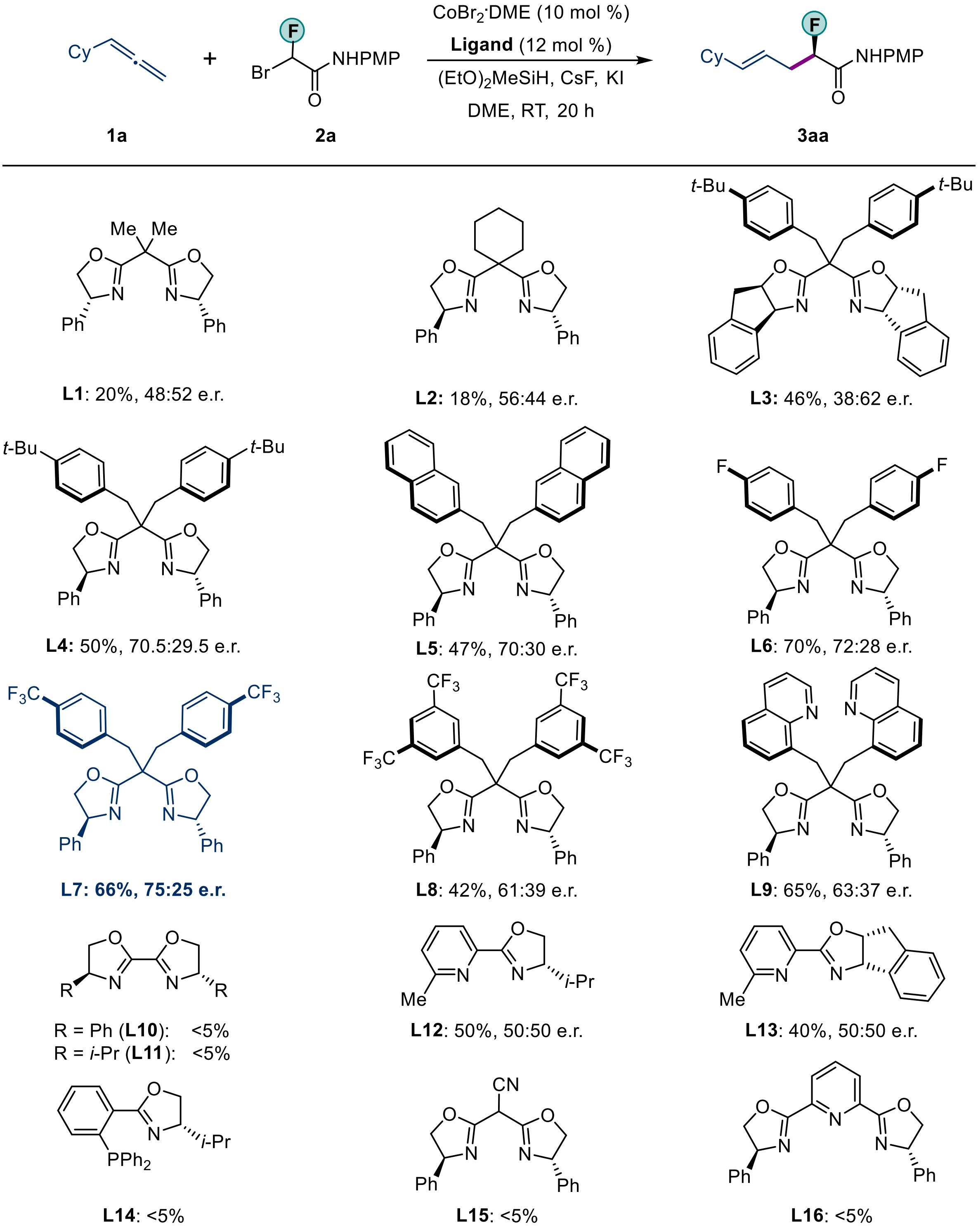

Conditions: $CoBr_2$.DME (10 mol %), ligand (12 mol %), **1a** (0.20 mmol), **2a** (0.10 mmol), $(EtO)_2MeSiH$ (0.25 mmol), CsF (0.25 mmol), KI (0.10 mmol) and DME (1.0 mL) at RT for 20 h; yields were measured by ^{19}F NMR of the crude reaction mixture. The e.r. was determined

using chiral HPLC analysis of the product after purification. DME, 1,2-Dimethoxyethane. PMP, 4-Methoxyphenyl.

Table S1. Screening of Different Reaction Conditions

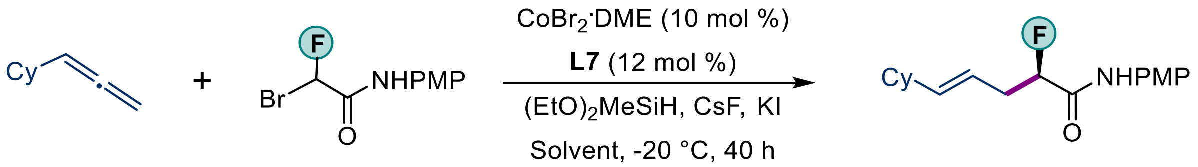

1a **2a** **3aa**

Entry	Catalyst	Base	Solvent	(*E:Z*)	Yield (%)	e.r.
1[a]	$CoBr_2$·DME	CsF	DME	>99:1	66	75:25
2[a]	$CoBr_2$·DME	CsF	diglyme	>99:1	42	76:24
3	$CoBr_2$·DME	CsF	diglyme	>99:1	45	89:11
4	$CoBr_2$·DME	CsF	MeCN	>99:1	18	90:10
5	$CoBr_2$·DME	CsF	diglyme/THF (1:1)	>99:1	54	88:12
6	$CoBr_2$·DME	CsF	diglyme/MeCN (1:1)	>99:1	90	95.5:4.5
**7**	**$CoBr_2$·DME**	**CsF**	**diglyme/MeCN (4:1)**	**>99:1**	**90 (86)**	**96.5:3.5**
8[b]	$NiBr_2$·DME	CsF	diglyme/MeCN (4:1)	14:86	40 (36)	55:45
9[b]	$NiBr_2$·DME	KF	diglyme/MeCN (4:1)	16:84	<10	55:45
10[b]	$NiBr_2$·DME	CsF	DMA	32:68	25	55:45
11[b]	$NiBr_2$·DME	KF	DMA	29:71	<10	55:45
12	---	CsF	diglyme/MeCN (4:1)	---	---	---
13[c]	$CoBr_2$·DME	CsF	diglyme/MeCN (4:1)	---	<5	---
14[d]	$CoBr_2$·DME	CsF	diglyme/MeCN (4:1)	---	---	---

Conditions: $CoBr_2$·DME (10 mol %), **L7** (12 mol %), **1a** (0.20 mmol), **2a** (0.10 mmol), $(EtO)_2MeSiH$ (0.25 mmol), CsF (0.25 mmol), KI (0.10 mmol) and Solvent (1.0 mL) at -20 °C for 40 h; yields were measured by ^{19}F NMR of the crude reaction mixture. The e.r. was determined using chiral HPLC analysis of the product after purification. Isolated yield is shown in the parenthesis. [a] RT, 20 h. [b] Some other minor isomer was also detected, [c] Without **L7**, [d] Without $(EtO)_2MeSiH$.

3. Synthesis of Starting Materials

3.1 General Procedure for the Synthesis of Allenes (1)

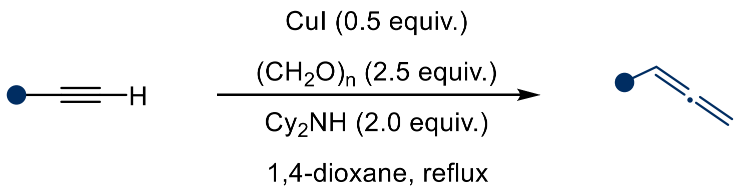

The allenes were synthesised according to previous literature procedure on gram-scale.[52-54] Alkyne (1.0 equiv) and Cy_2NH (2.0 equiv) were added to a solution of CuI (0.5 equiv), and paraformaldehyde (2.5 equiv) in 1,4-dioxane (0.2 M), and the resulting mixture was refluxed overnight. The reaction was then cooled to room temperature and filtered through a pad of celite, then concentrated. The residue was extracted with Et_2O or EtOAc (3 x 50 mL) and water (3 x 50 mL) and the organic portion was dried over anhydrous Na_2SO_4. After solvent removal, the crude mixture was purified by automated flash column chromatography to yield the products **1.** Then the products were distilled using Kugelrohr distillation set-up to remove the traces of metal impurities.

3.2 General Procedure for the Synthesis of Bromofluoroamides (2)

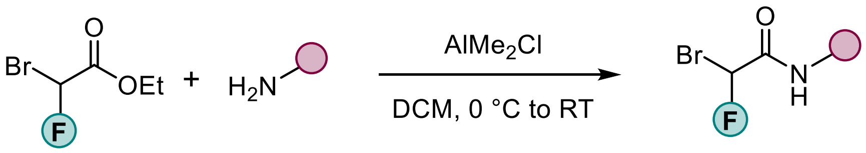

Bromofluoroamides **2** were prepared according to previous literature procedures on gram-scale.[36,38] To a solution of dimethylaluminium chloride (0.9 M, 10 mmol, 2.0 equiv.) in dry dichloromethane (40 mL), amine (10 mmol, 2.0 equiv.) was added dropwise at 0 °C and the mixture was stirred at this temperature for 30 min. Ethyl bromofluoroacetate (5 mmol, 1.0 equiv.) was added at this temperature and stirred overnight. The reaction was quenched with 2 M HCl until all aluminum salts were dissolved. The aqueous layer was extracted with dichloromethane (3 x 50 mL). The aqueous layer was extracted with dichloromethane (3 x 50 mL) and water (3 x 50 mL) and the organic portion was dried over anhydrous Na_2SO_4. After solvent removal, the crude mixture was purified by automated flash column chromatography to yield the products **2**.

2a, **2h**, **2i** were prepared according to previous literature procedures.[38]

2-Bromo-2-fluoro-*N*-phenylacetamide (2b)

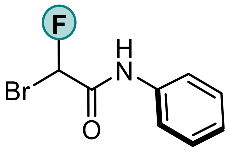

^{1}H NMR (400 MHz, $CDCl_3$) δ 7.96 (s, 1H), 7.64 – 7.56 (m, 2H), 7.45 – 7.37 (m, 2H), 7.27 – 7.20 (m, 1H), 6.75 (d, J = 51.0 Hz, 1H).

^{13}C NMR (101 MHz, $CDCl_3$) δ 162.34 (d, $^{2}J_{C-F}$ = 20.3 Hz), 135.99, 129.29, 125.75, 120.25, 84.75 (d, $^{1}J_{C-F}$ = 268.1 Hz).

^{19}F{H} NMR (377 MHz, $CDCl_3$) δ -146.57.

^{19}F NMR (377 MHz, $CDCl_3$) δ -146.57 (dd, J = 51.0, 3.9 Hz).

HRMS (APCI/QTOF) m/z: $[M + H]^+$ Calcd for $C_8H_8BrFNO^+$ 231.9773; Found 231.9762.

FTIR (neat): ṽ = 2989, 1670, 1554, 1445, 1143, 758 cm^{-1}.

2-Bromo-2-fluoro-*N*-(4-(methylthio)phenyl)acetamide (2c)

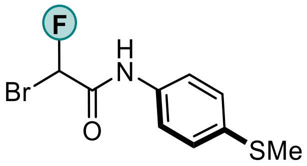

^{1}H NMR (400 MHz, $CDCl_3$) δ 7.90 (s, 1H), 7.60 – 7.42 (m, 2H), 7.35 – 7.18 (m, 2H), 6.72 (d, J = 51.0 Hz, 1H), 2.49 (s, 3H).

^{13}C NMR (101 MHz, $CDCl_3$) δ 162.23 (d, $^{2}J_{C-F}$ = 20.0 Hz), 135.73, 133.34, 127.64, 120.84, 120.81, 84.70 (d, $^{1}J_{C-F}$ = 268.1 Hz), 16.27.

^{19}F{H} NMR (376 MHz, $CDCl_3$) δ -146.67.

^{19}F NMR (376 MHz, $CDCl_3$) δ -146.66 (dd, J = 50.9, 3.8 Hz).

HRMS (ESI/QTOF) m/z: $[M + H]^+$ Calcd for $C_9H_{10}BrFNOS^+$ 277.9651; Found 277.9654.

FTIR (neat): ṽ = 3292, 1678, 1527, 1400, 1054, 815, 504 cm^{-1}.

2-Bromo-*N*-(4-cyanophenyl)-2-fluoroacetamide (2d)

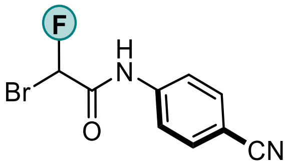

^{1}H NMR (400 MHz, $CDCl_3$) δ 8.16 (s, 1H), 7.75 (d, J = 8.8 Hz, 2H), 7.68 (d, J = 8.8 Hz, 2H), 6.74 (d, J = 51.0 Hz, 1H).

^{13}C NMR (101 MHz, $CDCl_3$) δ 162.69 (d, $^{2}J_{C-F}$ = 20.6 Hz), 140.04, 133.48, 120.23, 118.36, 108.89, 84.24 (d, $^{2}J_{C-F}$ = 268.0 Hz).

$^{19}F\{H\}$NMR (376 MHz, $CDCl_3$) δ -147.49.

^{19}F NMR (376 MHz, $CDCl_3$) δ -147.49 (dd, J = 50.8, 4.0 Hz).

HRMS (Sicrit plasma/LTQ-Orbitrap) m/z: $[M + H]^+$ Calcd for $C_9H_7BrFN_2O^+$ 256.9726; Found 256.9721.

FTIR (neat): $\tilde{\nu}$ = 3234, 1674, 1600, 1541, 1410, 1065, 840 cm^{-1}.

2-Bromo-2-fluoro-*N*-(4-nitrophenyl)acetamide (2e)

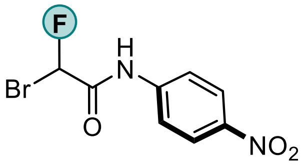

^{1}H NMR (400 MHz, $CDCl_3$) δ 8.28 (d, J = 9.1 Hz, 2H), 8.16 (bs, 1H), 7.91 – 7.70 (m, 2H), 6.76 (d, J = 50.9 Hz, 1H).

^{13}C NMR (101 MHz, $CDCl_3$) δ 162.66 (d, $^{2}J_{C-F}$ = 21.0 Hz), 144.72, 141.59, 125.22, 119.89, 84.18 (d, $^{1}J_{C-F}$ = 267.6 Hz).

$^{19}F\{H\}$ NMR (376 MHz, $CDCl_3$) δ -147.61.

^{19}F NMR (376 MHz, $CDCl_3$) δ -147.61 (dd, J = 51.2, 4.1 Hz).

HRMS (Sicrit plasma/LTQ-Orbitrap) m/z: $[M + H]^+$ Calcd for $C_8H_7BrFN_2O_3^+$ 276.9624; Found 276.9618.

2-Bromo-*N*-(4-bromophenyl)-2-fluoroacetamide (2f)

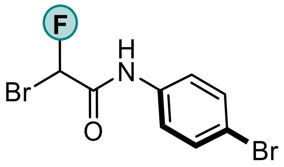

^{1}H NMR (400 MHz, $CDCl_3$) δ 8.01 (s, 1H), 7.52 – 7.42 (m, 4H), 6.71 (d, J = 50.9 Hz, 1H).

^{13}C NMR (101 MHz, $CDCl_3$) δ 162.43 (d, $^{2}J_{C-F}$ = 20.2 Hz), 135.07, 132.30, 121.84, 118.61, 84.55 (d, $^{1}J_{C-F}$ = 268.0 Hz).

$^{19}F\{H\}$ NMR (376 MHz, $CDCl_3$) δ -146.99.

^{19}F NMR (376 MHz, $CDCl_3$) δ -146.99 (dd, J = 50.9, 3.9 Hz).

HRMS (ESI/QTOF) m/z: $[M + Na]^+$ Calcd for $C_8H_6Br_2FNNaO^+$ 331.8698; Found 331.8700.

FTIR (neat): $\tilde{\nu}$ = 2986, 1680, 1566, 1496, 1144, 853, 505 cm^{-1}.

2-Bromo-2-fluoro-*N*-(4-(4,4,5,5-tetramethyl-1,3,2-dioxaborolan-2-yl)phenyl)acetamide (2g)

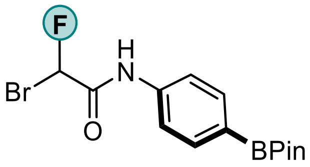

^{1}H NMR (400 MHz, $CDCl_3$) δ 7.96 (s, 1H), 7.89 – 7.75 (m, 2H), 7.69 – 7.49 (m, 2H), 6.71 (d, J = 51.0 Hz, 1H), 1.34 (s, 12H).

^{13}C NMR (101 MHz, $CDCl_3$) δ 162.28 (d, $^{2}J_{C–F}$ = 20.2 Hz), 138.54, 135.97, 119.07, 84.68 (d, $^{1}J_{C–F}$ = 268.0 Hz), 83.35, 24.88, 24.87.

^{19}F{H} NMR (376 MHz, $CDCl_3$) δ -146.59.

^{19}F NMR (376 MHz, $CDCl_3$) δ -146.59 (dd, J = 51.1, 4.1 Hz).

HRMS (ESI/QTOF) m/z: $[M + H]^+$ Calcd for $C_{14}H_{19}BBrFNO_3^+$ 358.0628; Found 358.0628.

FTIR (neat): $\tilde{\nu}$ = 2870, 1668, 1548, 1488, 1142, 1073, 824 cm^{-1}.

2-Bromo-*N*-cyclohexyl-2-fluoroacetamide (2j)

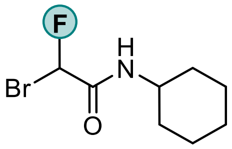

^{1}H NMR (400 MHz, $CDCl_3$) δ 6.59 (d, J = 51.1 Hz, 1H), 6.15 (s, 1H), 3.84 (dddd, J = 15.3, 11.5, 9.2, 3.9 Hz, 1H), 2.05 – 1.92 (m, 2H), 1.77 (dh, J = 15.0, 3.5 Hz, 2H), 1.71 – 1.62 (m, 1H), 1.48 – 1.34 (m, 2H), 1.31 – 1.17 (m, 3H).

^{13}C NMR (101 MHz, $CDCl_3$) δ 163.76 (d, $^{2}J_{C–F}$ = 20.2 Hz), 84.85 (d, $^{1}J_{C–F}$ = 267.2 Hz), 48.74, 32.74, 32.48, 25.34, 24.69, 24.63.

^{19}F{H} NMR (376 MHz, $CDCl_3$) δ -147.31.

^{19}F NMR (376 MHz, $CDCl_3$) δ -147.31 (dd, J = 51.0, 2.7 Hz).

HRMS (ESI/QTOF) m/z: $[M + Na]^+$ Calcd for $C_8H_{13}BrFNNaO^+$ 260.0062; Found 260.0073.

FTIR (neat): $\tilde{\nu}$ = 3236, 1661, 1573, 1093, 1047, 658 cm^{-1}.

2-Bromo-2-fluoro-*N*-methoxy-*N*-methylacetamide (S1-1)

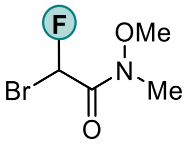

^{1}H NMR (400 MHz, $CDCl_3$) δ 6.96 (d, J = 51.3 Hz, 1H), 3.80 (s, 3H), 3.26 (s, 3H).

^{19}F{H} NMR (377 MHz, $CDCl_3$) δ -152.41.

^{19}F NMR (377 MHz, $CDCl_3$) δ -152.41 (d, J = 51.3 Hz).

^{13}C NMR (101 MHz, $CDCl_3$) δ 164.75 (d, $^{2}J_{C–F}$ = 25.3 Hz), 80.06 (d, $^{1}J_{C–F}$ = 255.4 Hz), 61.50, 32.56.

HRMS (nanochip-ESI/LTQ-Orbitrap) m/z: $[M + H]^+$ Calcd for $C_4H_8BrFNO_2^+$ 199.9722; Found 199.9720.

FTIR (neat): $\tilde{\nu}$ = 1681, 1421, 1389, 1174, 987, 795, 616 cm^{-1}.

3.2 General Procedure for the Synthesis of Bromotrifluoromethylacetamides (6)

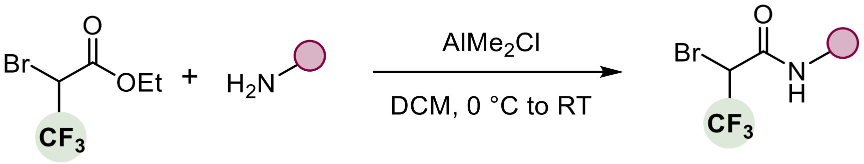

To a solution of dimethylaluminium chloride (0.9 M, 1.01 mL, 905 µmol, 2.0 equiv.) in dry dichloromethane (10 mL), amine (905 µmol, 2.0 equiv.) was added dropwise at 0 °C and the mixture was stirred at this temperature for 30 min. Methyl 2-bromo-3,3,3-trifluoropropionate (100 mg, 453 µmol, 1.0 equiv.) was added dropwise and the reaction mixture was allowed to warm up and stirred at room temperature overnight. The reaction was quenched with 2 M HCl until all aluminium salts were dissolved. The aqueous layer was extracted with dichloromethane (3 x 10 mL) and water (3 x 10 mL) and the organic portion was dried over anhydrous Na_2SO_4. After solvent removal, the crude mixture was purified by automated flash column chromatography to yield the products **6**.

2-Bromo-3,3,3-trifluoro-*N*-(4-methoxyphenyl)propenamide (6a)

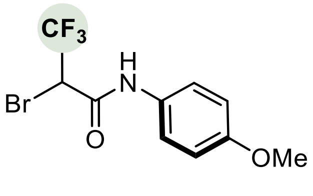

^{1}H NMR (400 MHz, $CDCl_3$) δ 7.82 (s, 1H), 7.49 – 7.33 (m, 2H), 7.02 – 6.77 (m, 2H), 4.73 (q, J = 7.1 Hz, 1H), 3.83 (s, 3H).

^{13}C NMR (101 MHz, $CDCl_3$) δ 158.66 (d, $^{3}J_{C–F}$ = 1.8 Hz), 129.20, 122.25, 122.24 (q, $^{1}J_{C–F}$ = 275.1 Hz) 122.14, 118.01, 114.35, 55.53, (q, $^{1}J_{C–F}$ = 33.0 Hz).

^{19}F{H} NMR (376 MHz, $CDCl_3$) δ -68.80.

^{19}F NMR (377 MHz, $CDCl_3$) δ -68.80 (d, J = 7.1 Hz).

HRMS (ESI/QTOF) m/z: $[M + H]^+$ Calcd for $C_{10}H_{10}BrF_3NO_2^+$ 311.9847; Found 311.9852.
FTIR (neat): $\tilde{\nu}$ = 3259, 1664, 1548, 1511, 1246, 1114, 828 cm^{-1}.

2-Bromo-*N*-(3-chlorophenyl)-3,3,3-trifluoropropanamide (6b)

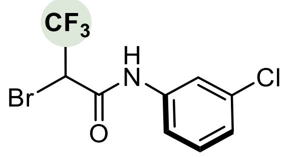

^1H NMR (400 MHz, CD_3CN) δ 9.18 (s, 1H), 7.72 (t, J = 2.1 Hz, 1H), 7.48 (ddd, J = 8.2, 2.0, 1.0 Hz, 1H), 7.38 (t, J = 8.1 Hz, 1H), 7.23 (ddd, J = 8.0, 2.0, 1.0 Hz, 1H), 4.98 (q, J = 6.8 Hz, 1H).

^{13}C NMR (101 MHz, CD_3CN) δ 161.12 (d, $^3J_{C–F}$ = 1.8 Hz), 139.55, 134.81, 131.28, 125.79, 123.31 (d, $^1J_{C–F}$ = 277.2 Hz), 120.65, 119.20, 42.65 (q, $^2J_{C–F}$ = 33.0 Hz).
$^{19}F\{H\}$ NMR (377 MHz, CD_3CN) δ -69.60.
^{19}F NMR (377 MHz, CD_3CN) δ -69.60 (d, J = 6.9 Hz).
HRMS (ESI/QTOF) m/z: $[M + Na]^+$ Calcd for $C_9H_6BrClF_3NNaO^+$ 337.9171; Found 337.9175.
FTIR (neat): $\tilde{\nu}$ = 3261, 1660, 1545, 1144, 1112, 619 cm^{-1}.

2-Bromo-3,3,3-trifluoro-*N*-(1-methyl-1*H*-indol-5-yl)propenamide (6c)

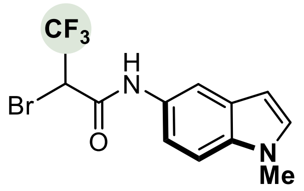

^1H NMR (400 MHz, CD_3CN) δ 8.71 (bs, 1H), 7.73 (d, J = 2.0 Hz, 1H), 7.28 (dt, J = 8.7, 0.7 Hz, 1H), 7.16 (dd, J = 8.8, 2.1 Hz, 1H), 7.11 (d, J = 3.1 Hz, 1H), 6.36 (dd, J = 3.1, 0.9 Hz, 1H), 4.87 (q, J = 6.9 Hz, 1H), 3.70 (s, 3H).
^{13}C NMR (101 MHz, CD_3CN) δ 160.34 (d, $^3J_{C–F}$ = 2.1 Hz), 135.13, 131.09, 130.01, 128.92, (d, $^1J_{C–F}$ = 280 Hz), 115.96, 113.22, 110.26, 101.22, 42.98 (q, $^2J_{C–F}$ = 32.8 Hz), 32.90.
$^{19}F\{H\}$ (376 MHz, CD_3CN) δ -69.66.
^{19}F NMR (376 MHz, CD_3CN) δ -69.65 (d, J = 6.9 Hz).
HRMS (APCI/QTOF) m/z: $[M + H]^+$ Calcd for $C_{12}H_{11}BrF_3N_2O^+$ 335.0007; Found 335.0000.
FTIR (neat): $\tilde{\nu}$ = 3245, 1662, 1545, 1508, 1250, 827, 799 cm^{-1}.

3.2 General Procedure for the Synthesis of Bromochloroacetamides (8)

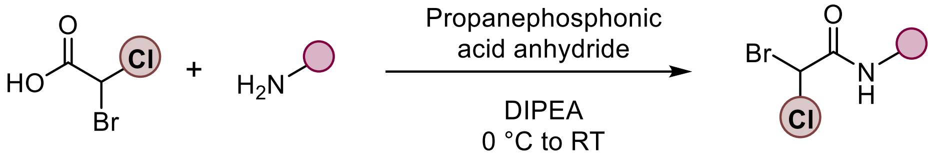

Bromochloroacetamides were prepared according to literature procedure.[37] To a stirred solution of bromochloroacetic acid (175 µL, 2 mmol, 2.0 equiv.) and 1-propanephosphonic acid cyclic anhydride (50%, 1.79 mL, 3 mmol, 3 equiv.) in dry CH_2Cl_2 (10.0 mL) was added DIPEA (661 µL, 4 mmol, 4 equiv.) and amine (1 mmol, 1.0 equiv.) at 0 °C. After stirring for 1 h at ambient temperature, the reaction mixture was diluted with CH_2Cl_2 and brine. The aqueous layer was extracted with dichloromethane (3 x 10 mL) and water (3 x 10 mL) and the organic portion was dried over anhydrous Na_2SO_4. After solvent removal, the crude mixture was purified by automated flash column chromatography to yield the products **8**.

2-Bromo-2-chloro-*N*-(4-methoxyphenyl)acetamide (8a)

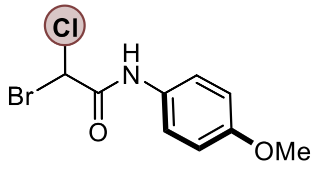

^{1}H NMR (400 MHz, $CDCl_3$) δ 8.10 (s, 1H), 7.57 – 7.34 (m, 2H), 7.01 – 6.75 (m, 2H), 6.04 (s, 1H), 3.81 (s, 3H).
^{13}C NMR (101 MHz, $CDCl_3$) δ 162.04, 157.40, 129.31, 122.09, 114.37, 55.53, 52.31.
HRMS (ESI/QTOF) m/z: $[M + H]^+$ Calcd for $C_9H_{10}BrClNO_2^+$ 277.9578; Found 277.9576.
FTIR (neat): $\tilde{v}$ = 3245, 1662, 1545, 1508, 1250, 1027, 827 cm^{-1}.

Methyl 4-(2-bromo-2-chloroacetamido)benzoate (8b)

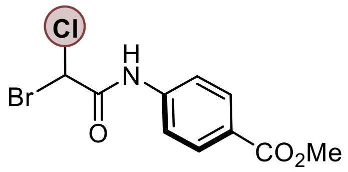

^{1}H NMR (400 MHz, $CDCl_3$) δ 8.30 (s, 1H), 8.14 – 7.99 (m, 2H), 7.74 – 7.56 (m, 2H), 6.05 (s, 1H), 3.92 (s, 3H).
^{13}C NMR (101 MHz, $CDCl_3$) δ 166.35, 162.16, 140.48, 130.95, 127.03, 119.37, 52.21, 51.99.
HRMS (ESI/QTOF) m/z: $[M + H]^+$ Calcd for $C_{10}H_{10}BrClNO_3^+$ 305.9533; Found 305.9534.

FTIR (neat): $\tilde{\nu}$ = 2989, 1720, 1675, 1276, 1111, 766 cm^{-1}.

***N*-(4-Acetylphenyl)-2-bromo-2-chloroacetamide (8c)**

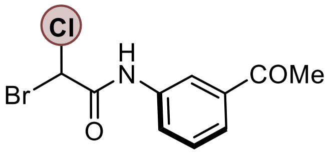

^{1}H NMR (400 MHz, $CDCl_3$) δ 8.44 (s, 1H), 8.03 (t, *J* = 1.9 Hz, 1H), 7.88 (ddd, *J* = 8.1, 2.3, 1.0 Hz, 1H), 7.71 (ddd, *J* = 7.8, 1.7, 1.0 Hz, 1H), 7.43 (t, *J* = 7.9 Hz, 1H), 6.02 (s, 1H), 2.57 (s, 3H).

^{13}C NMR (101 MHz, $CDCl_3$) δ 197.78, 162.45, 137.92, 137.12, 129.65, 125.47, 124.78, 119.70, 52.11, 26.76.

HRMS (ESI/QTOF) m/z: $[M + H]^+$ Calcd for $C_{10}H_{10}BrClNO_2^+$ 289.9583; Found 289.9586.

FTIR (neat): $\tilde{\nu}$ = 3303, 1712, 1668,1609, 1554, 1480, 1276, 801cm^{-1}.

2-Bromo-2-chloro-*N*-(1-methyl-1*H*-indol-5-yl)acetamide (8d)

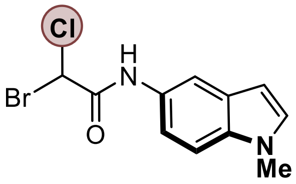

^{1}H NMR (400 MHz, CD_3CN) δ 8.80 (s, 1H), 7.74 (d, *J* = 2.1 Hz, 1H), 7.28 (d, *J* = 8.8 Hz, 1H), 7.18 (dd, *J* = 8.8, 2.1 Hz, 1H), 7.11 (d, *J* = 3.1 Hz, 1H), 6.36 (dd, *J* = 3.1, 0.9 Hz, 1H), 6.23 (s, 1H), 3.70 (s, 3H).

^{13}C NMR (101 MHz, CD_3CN) δ 162.95, 135.12, 131.05, 129.85, 128.94, 116.13, 113.42, 113.33, 110.24, 101.18, 54.01, 32.91.

HRMS (ESI/QTOF) m/z: $[M + H]^+$ Calcd for $C_{11}H_{11}BrClN_2O^+$ 300.9738; Found 300.9736.

FTIR (neat): $\tilde{\nu}$ = 2989, 1671, 1545, 1421, 1133, 863, 555 cm^{-1}.

***N*-Benzyl-2-bromo-2-chloroacetamide (8e)**

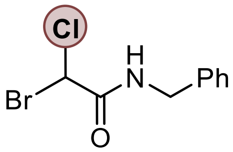

^{1}H NMR (400 MHz, $CDCl_3$) δ 7.47 – 7.22 (m, 5H), 5.97 (s, 1H), 4.59 – 4.43 (m, 2H).

^{13}C NMR (101 MHz, $CDCl_3$) δ 164.43, 136.79, 128.94, 128.00, 127.71, 51.85, 44.35.

HRMS (ESI/QTOF) m/z: $[M + H]^+$ Calcd for $C_9H_{10}BrClNO^+$ 261.9634; Found 261.9641.

FTIR (neat): $\tilde{\nu}$ = 2989, 1660, 1545, 1144, 1112, 620 cm^{-1}.

2-Bromo-2-chloro-*N*-(4-phenylbutyl)acetamide (8f)

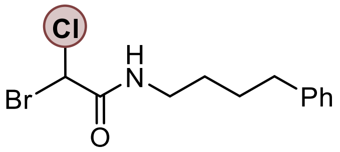

^1H NMR (400 MHz, $CDCl_3$) δ 7.34 – 7.07 (m, 5H), 6.46 (s, 1H), 5.91 (s, 1H), 3.35 (tdd, *J* = 6.9, 5.9, 1.6 Hz, 2H), 2.66 (t, *J* = 7.4 Hz, 2H), 1.75 – 1.49 (m, 4H).

^{13}C NMR (101 MHz, $CDCl_3$) δ 164.41, 141.81, 128.42, 128.41, 125.95, 51.98, 40.27, 35.36, 28.65, 28.37.

HRMS (APCI/QTOF) m/z: $[M + H]^+$ Calcd for $C_{12}H_{16}BrClNO^+$ 304.0098; Found 304.0085.

FTIR (neat): $\tilde{\nu}$ = 2934, 1664, 1557, 1210, 803, 688 cm^{-1}.

2-Bromo-2-chloro-*N*-methoxy-*N*-methylacetamide (S2-1)

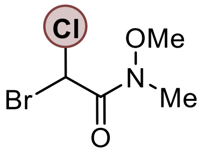

^1H NMR (400 MHz, $CDCl_3$) δ 6.55 (s, 1H), 3.84 (s, 3H), 3.30 (s, 3H).

^{13}C NMR (101 MHz, $CDCl_3$) δ 164.79, 61.83, 48.28, 33.05.

HRMS (nanochip-ESI/LTQ-Orbitrap) m/z: $[M + H]^+$ Calcd for $C_4H_8BrClNO_2^+$ 215.9421; Found 215.9426.

FTIR (neat): $\tilde{\nu}$ = 1679, 1443, 1387, 989, 794, 616 cm^{-1}.

5. Product Characterization Data

(*R*,*E*)-2-Fluoro-*N*-(4-methoxyphenyl)-7-phenylhept-4-enamide (3ba)

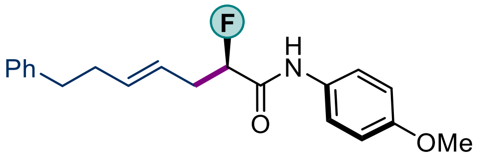

Prepared according to **GP1** with 2-bromo-2-fluoro-*N*-(4-methoxyphenyl)acetamide (**2a**) (26.2 mg, 0.10 mmol, 1.0 equiv.) and penta-3,4-dien-1-ylbenzene (**1b**) (28.8 mg, 0.20 mmol, 2.0 equiv.). Automated flash column chromatography (10 g SiO_2, gradient elution: hexane to 20% EtOAc in hexane) afforded the desired product (+) **3ba** as white solid (24 mg, 73%).

^{1}H NMR (400 MHz, $CDCl_3$) δ 7.83 (d, *J* = 6.5 Hz, 1H), 7.49 – 7.42 (m, 2H), 7.28 (tt, *J* = 7.2, 1.2 Hz, 2H), 7.23 – 7.14 (m, 3H), 6.93 – 6.86 (m, 2H), 5.70 (dtt, *J* = 15.4, 7.8, 1.2 Hz, 1H), 5.49 (dtt, *J* = 15.3, 6.9, 1.4 Hz, 1H), 5.01 (ddd, *J* = 49.7, 6.8, 3.8 Hz, 1H), 3.82 (s, 3H), 2.87 – 2.56 (m, 4H), 2.36 (q, *J* = 7.4 Hz, 2H).

^{13}C NMR (101 MHz, $CDCl_3$) δ 167.30 (d, $^{2}J_{C\text{–}F}$ = 18.2 Hz), 156.90, 141.74, 134.79, 129.67, 128.48, 128.28, 125.83, 123.07 (d, $^{3}J_{C\text{–}F}$ = 2.9 Hz), 121.91, 114.26, 91.52 (d, $^{1}J_{C\text{–}F}$ = 188.9 Hz), 55.51, 35.70, 35.57 (d, $^{2}J_{C\text{–}F}$ = 20.0 Hz), 34.39.

^{19}F{H} NMR (377 MHz, $CDCl_3$) δ -188.19.

^{19}F NMR (376 MHz, $CDCl_3$) δ -187.97 – -188.38 (m).

HRMS (ESI/QTOF) m/z: $[M + Na]^+$ Calcd for $C_{20}H_{22}FNNaO_2^+$ 350.1523; Found 350.1529.

$[\alpha]_D^{23}$ = +60.6 (c = 1.2 in $CHCl_3$).

FTIR (neat): $\tilde{\nu}$ = 3334, 1660, 1599, 1514, 1453, 1068, 822, 698 cm^{-1}.

HPLC: The enantiomeric ratio (96.5:3.5) was determined *via* HPLC analysis using a CHIRALCEL® IA column, with hexane:isopropanol = 98:2 at a flow rate 1.0 mL/min detected at 254 nm wavelength. Retention time: t_{major} = 30.7 min and t_{minor} = 41.7 min.

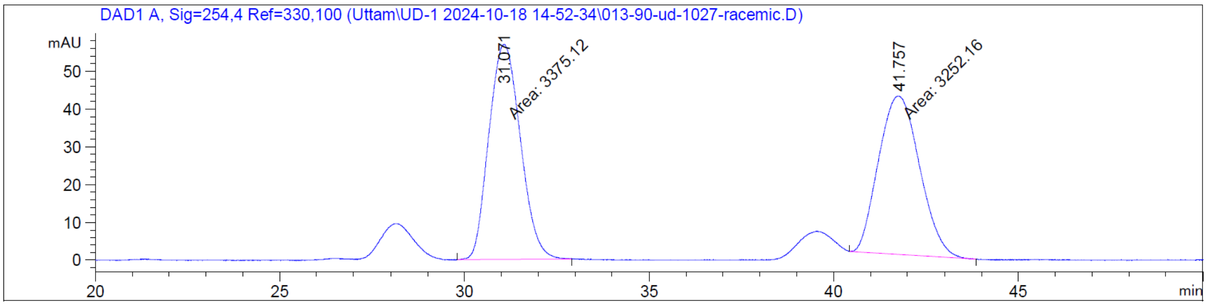

Peak #	RetTime [min]	Type	Width [min]	Area [mAU*s]	Height [mAU]	Area %
1	31.071	MM	0.9891	3375.12256	56.86988	50.9277
2	41.757	MM	1.2902	3252.16040	42.01035	49.0723

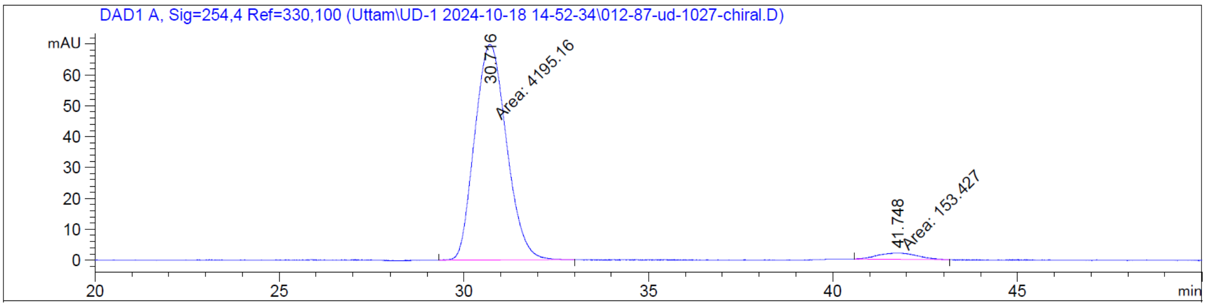

Peak #	RetTime [min]	Type	Width [min]	Area [mAU*s]	Height [mAU]	Area %
1	30.716	MM	1.0015	4195.16406	69.81663	96.4718
2	41.748	MM	1.2110	153.42749	2.11155	3.5282

(*R*,*E*)-9-Chloro-2-fluoro-*N*-phenylnon-4-enamide (3ca)

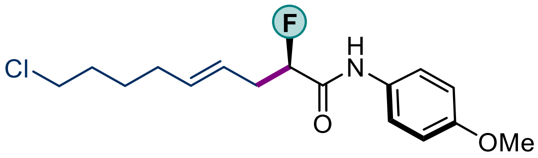

Prepared according to **GP1** with 2-bromo-2-fluoro-*N*-(4-methoxyphenyl)acetamide (**2a**) (26.2 mg, 0.10 mmol, 1.0 equiv.) and 7-chlorohepta-1,2-diene (**1c**) (26.1 mg, 0.20 mmol, 2.0 equiv.). Automated flash column chromatography (10 g SiO_2, gradient elution: hexane to 20% EtOAc in hexane) afforded the desired product (+) **3ca** as yellow oil (22 mg, 70%).

^{1}H NMR (400 MHz, $CDCl_3$) δ 7.85 (d, J = 6.6 Hz, 1H), 7.54 – 7.39 (m, 2H), 6.94 – 6.82 (m, 2H), 5.62 (dt, J = 14.0, 6.7 Hz, 1H), 5.47 (dt, J = 14.8, 6.9 Hz, 1H), 5.01 (ddd, J = 49.6, 6.6, 3.9 Hz, 1H), 3.80 (s, 3H), 3.47 (t, J = 6.7 Hz, 2H), 2.71 (ddtd, J = 51.6, 29.2, 15.0, 6.2 Hz, 2H), 2.05 (q, J = 7.1 Hz, 2H), 1.79 – 1.67 (m, 2H), 1.50 (q, J = 7.5 Hz, 2H).

^{13}C NMR (101 MHz, $CDCl_3$) δ 167.23 (d, $^2J_{C–F}$ = 18.2 Hz), 156.88, 134.99, 129.68, 123.00 (d, $^3J_{C–F}$ = 2.9 Hz), 121.76, 114.27, 91.54 (d, $^1J_{C–F}$ = 188.9 Hz), 55.51, 44.92, 35.50 (d, $^2J_{C–F}$ = 20.0 Hz), 31.89, 31.75, 26.36.

$^{19}F\{H\}$ NMR (377 MHz, $CDCl_3$) δ -188.33.

^{19}F NMR (377 MHz, $CDCl_3$) δ -188.33 (dddd, J = 49.8, 29.9, 26.6, 6.5 Hz).

HRMS (ESI/QTOF) m/z: $[M + Na]^+$ Calcd for $C_{16}H_{21}ClFNNaO_2^+$ 336.1137; Found 336.1143.

$[\alpha]_D^{23}$ = +38.5 (c = 0.75 in $CHCl_3$).

FTIR (neat): $\tilde{\nu}$ = 2957, 1661, 1540, 1247, 1068, 966, 650 cm^{-1}.

HPLC: The enantiomeric ratio (97:3) was determined *via* HPLC analysis using a CHIRALCEL® AD-H column, with hexane:isopropanol = 90:10 at a flow rate 1.0 mL/min detected at 254 nm wavelength. Retention time: t_{major} = 16.3 min and t_{minor} = 22.5 min.

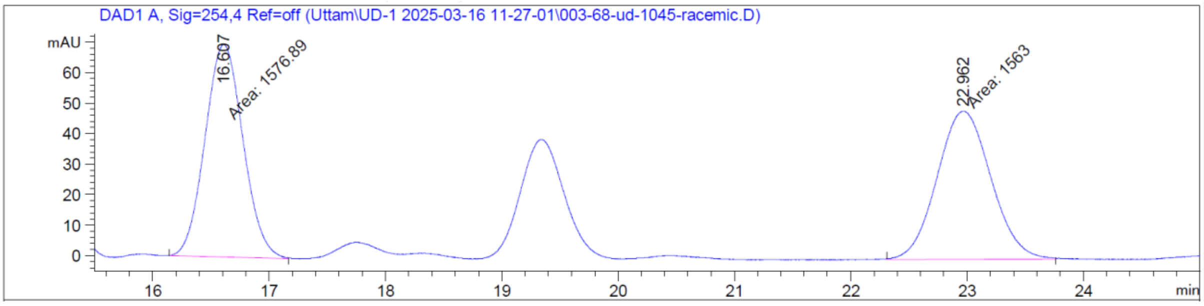

Peak #	RetTime [min]	Type	Width [min]	Area [mAU*s]	Height [mAU]	Area %
1	16.607	MM	0.3779	1576.88745	69.53776	50.2211
2	22.962	MM	0.5359	1563.00012	48.61055	49.7789

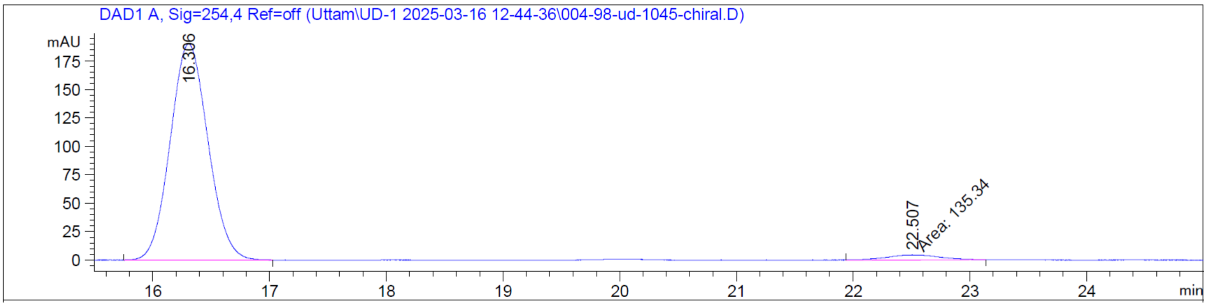

Peak #	RetTime [min]	Type	Width [min]	Area [mAU*s]	Height [mAU]	Area %
1	16.306	BV	0.2642	4279.22803	190.04727	96.9342
2	22.507	MM	0.5143	135.34045	4.38590	3.0658

Methyl (*R*,*E*)-8-fluoro-9-((4-methoxyphenyl)amino)-9-oxonon-5-enoate (3da)

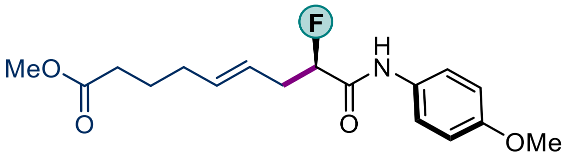

Prepared according to **GP1** with 2-bromo-2-fluoro-*N*-(4-methoxyphenyl)acetamide (**2a**) (26.2 mg, 0.10 mmol, 1.0 equiv.) and methyl hepta-5,6-dienoate (**1d**) (28 mg, 0.20 mmol, 2.0 equiv.). Automated flash column chromatography (10 g SiO_2, gradient elution: hexane to 30% EtOAc in hexane) afforded the desired product (+) **3da** as sticky solid (24.5 mg, 76%).

^{1}H NMR (400 MHz, $CDCl_3$) δ 7.82 (d, *J* = 6.4 Hz, 1H), 7.43 – 7.34 (m, 2H), 6.87 – 6.73 (m, 2H), 5.53 (dt, *J* = 13.9, 6.7 Hz, 1H), 5.41 (dt, *J* = 14.9, 6.9 Hz, 1H), 4.94 (ddd, *J* = 49.6, 6.7, 3.9 Hz, 1H), 3.73 (s, 3H), 3.58 (s, 3H), 2.78 – 2.48 (m, 2H), 2.21 (t, *J* = 7.5 Hz, 2H), 1.99 (q, *J* = 7.1 Hz, 2H), 1.63 (q, *J* = 7.4 Hz, 2H).

^{13}C NMR (101 MHz, $CDCl_3$) δ 174.02, 167.25 (d, $^{2}J_{C–F}$ = 18.2 Hz), 156.86, 134.43, 129.71, 123.53 (d, $^{3}J_{C–F}$ = 2.9 Hz), 121.81, 114.25, 91.45 (d, $^{1}J_{C–F}$ = 188.9 Hz), 55.49, 51.50, 35.50 (d, $^{2}J_{C–F}$ = 20.0 Hz), 33.24, 31.87, 24.28.

^{19}F{H} NMR (377 MHz, $CDCl_3$) δ -188.29.

^{19}F NMR (376 MHz, $CDCl_3$) δ -188.29 (dddd, *J* = 49.8, 29.7, 26.8, 6.5 Hz).

HRMS (ESI/QTOF) m/z: $[M + Na]^+$ Calcd for $C_{17}H_{22}FNNaO_4^+$ 346.1431; Found 346.1433.

$[\alpha]_D^{23}$ = +30.9 (c = 1.00 in $CHCl_3$).

FTIR (neat): $\tilde{\nu}$ = 2923, 1733, 1660, 1540, 1514, 1245, 1175, 821 cm^{-1}.

HPLC: The enantiomeric ratio (96:4) was determined *via* HPLC analysis using a CHIRALCEL® IA column, with hexane:isopropanol = 85:15 at a flow rate 1.0 mL/min detected at 254 nm wavelength. Retention time: t_{major} = 12.7 min and t_{minor} = 14.7 min.

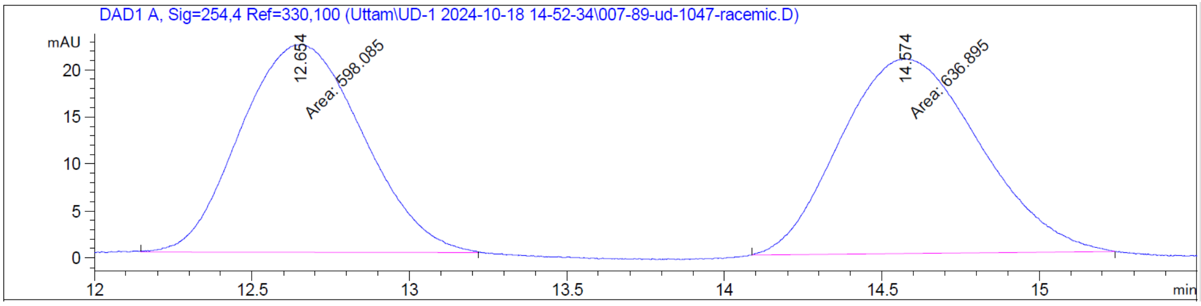

Peak #	RetTime [min]	Type	Width [min]	Area [mAU*s]	Height [mAU]	Area %
1	12.654	MM	0.4505	598.08459	22.12896	48.4287
2	14.574	MM	0.5121	636.89514	20.72888	51.5713

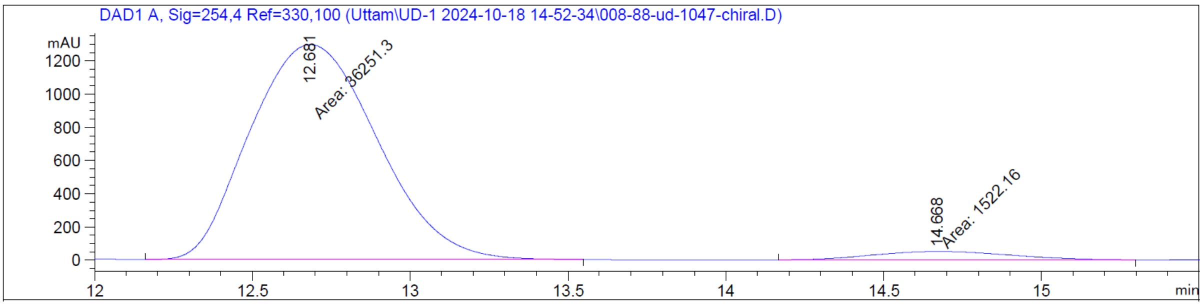

Peak #	RetTime [min]	Type	Width [min]	Area [mAU*s]	Height [mAU]	Area %
1	12.681	MM	0.4664	3.62513e4	1295.31140	95.9703
2	14.668	MM	0.5033	1522.16370	50.40993	4.0297

(*R*,*E*)-8-(1,3-Dioxoisoindolin-2-yl)-2-fluoro-*N*-(4-methoxyphenyl)oct-4-enamide (3ea)

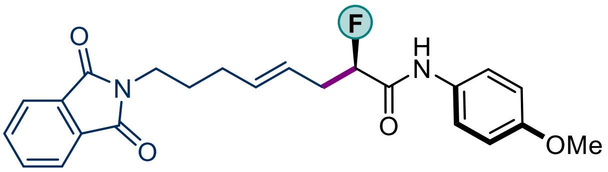

Prepared according to **GP1** with 2-bromo-2-fluoro-*N*-(4-methoxyphenyl)acetamide (**2a**) (26.2 mg, 0.10 mmol, 1.0 equiv.) and 2-(hexa-4,5-dien-1-yl)isoindoline-1,3-dione (**1e**) (45.5 mg, 0.20 mmol, 2.0 equiv.). Automated flash column chromatography (10 g SiO_2, gradient elution: hexane to 50% EtOAc in hexane) afforded the desired product (+) **3ea** as sticky solid (31.5 mg, 77%).

^{1}H NMR (400 MHz, $CDCl_3$) δ 7.91 (d, *J* = 6.2 Hz, 1H), 7.75 (dd, *J* = 5.4, 3.1 Hz, 2H), 7.63 (dd, *J* = 5.4, 3.1 Hz, 2H), 7.41 – 7.36 (m, 2H), 6.79 – 6.74 (m, 2H), 5.56 (dt, *J* = 15.3, 6.5 Hz, 1H), 5.49 – 5.39 (m, 1H), 4.93 (ddd, *J* = 49.6, 6.4, 4.0 Hz, 1H), 3.68 (s, 3H), 3.56 (td, *J* = 7.0, 1.1 Hz, 2H), 2.74 – 2.47 (m, 2H), 2.01 (q, *J* = 7.0 Hz, 2H), 1.70 – 1.64 (m, 2H).

^{13}C NMR (101 MHz, $CDCl_3$) δ 168.39, 167.28 (d, $^2J_{C–F}$ = 18.2 Hz), 156.77, 134.04, 133.89, 132.13, 129.80, 123.53 (d, $^3J_{C–F}$ = 2.8 Hz), 123.17, 121.81, 114.18, 91.38 (d, $^1J_{C–F}$ = 189.0 Hz), 55.44, 37.35, 35.48 (d, $^2J_{C–F}$ = 20.2 Hz), 29.72, 27.81.

^{19}F{H} NMR (376 MHz, $CDCl_3$) δ -188.33.

^{19}F NMR (376 MHz, $CDCl_3$) δ -188.33 (dddd, J = 49.6, 30.0, 26.1, 6.3 Hz).

HRMS (ESI/QTOF) m/z: $[M + Na]^+$ Calcd for $C_{23}H_{23}FN_2NaO_4^+$ 433.1540; Found 433.1541.

$[\alpha]_D^{23}$ = +36.4 (c = 1.00 in $CHCl_3$).

FTIR (neat): $\tilde{\nu}$ = 3321, 1695, 1663, 1531, 1400, 1299, 715 cm^{-1}.

HPLC: The enantiomeric ratio (94:6) was determined *via* HPLC analysis using a CHIRALCEL® IA column, with hexane:isopropanol = 80:20 at a flow rate 1.0 mL/min detected at 254 nm wavelength. Retention time: t_{major} = 23.6 min and t_{minor} = 34.7 min.

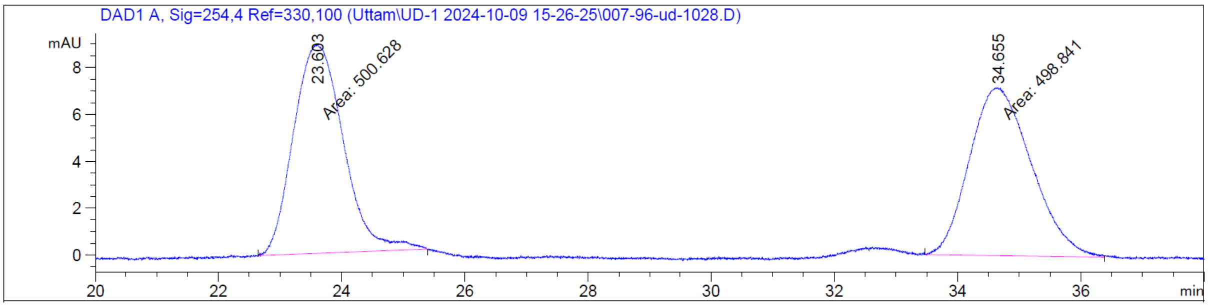

Peak #	RetTime [min]	Type	Width [min]	Area [mAU*s]	Height [mAU]	Area %
1	23.603	MM	0.9365	500.62784	8.90942	50.0894
2	34.655	MM	1.1590	498.84140	7.17375	49.9106

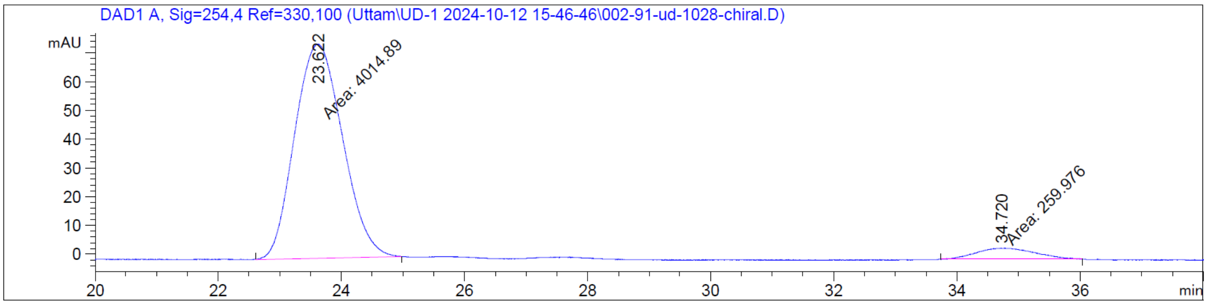

Peak #	RetTime [min]	Type	Width [min]	Area [mAU*s]	Height [mAU]	Area %
1	23.622	MM	0.8998	4014.89233	74.36865	93.9185
2	34.720	MM	1.1150	259.97614	3.88618	6.0815

(*R*,*E*)-2-Fluoro-8-hydroxy-N-(4-methoxyphenyl)oct-4-enamide (3fa)

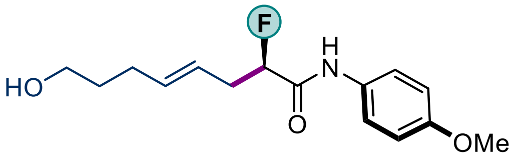

Prepared according to **GP1** with 2-bromo-2-fluoro-*N*-(4-methoxyphenyl)acetamide (**2a**) (26.2 mg, 0.10 mmol, 1.0 equiv.) and hexa-4,5-dien-1-ol (**1f**) (19.6 mg, 0.20 mmol, 2.0 equiv.). After stirring for 40 hours, the reaction mixture was diluted with EtOAc (4.0 mL) and the solvent was removed in *vacuo*. Then crude mixture was stirred with NaF (21 mg, 5.0 equiv.) in THF for 12 hours. The organic phase was extracted with water (3 x 5.0 mL) and EtOAc (3 x 5.0 mL). The combined organic phases were dried over Na_2SO_4, and the volatiles were removed to afford the crude product. Automated flash column chromatography (10 g SiO_2, gradient elution: hexane to 50% EtOAc in hexane) afforded the desired product (+) **3fa** as yellow oil (17 mg, 60%).

^{1}H NMR (400 MHz, $CDCl_3$) δ 7.89 (d, J = 6.4 Hz, 1H), 7.51 – 7.40 (m, 2H), 6.94 – 6.82 (m, 2H), 5.65 (dt, J = 14.2, 6.7 Hz, 1H), 5.57 – 5.41 (m, 1H), 5.01 (ddd, J = 49.7, 6.6, 4.0 Hz, 1H), 3.80 (s, 3H), 3.61 (t, J = 6.6 Hz, 2H), 2.69 (dddt, J = 36.1, 29.0, 14.6, 8.3 Hz, 2H), 2.12 (q, J = 7.1 Hz, 2H), 1.66 – 1.57 (m, 3H).

^{13}C NMR (101 MHz, $CDCl_3$) δ 167.26 (d, $^2J_{C–F}$ = 18.2 Hz), 156.88, 135.03, 129.70, 123.00 (d, $^3J_{C–F}$ = 3.2 Hz), 121.80, 114.27, 91.50 (d, $^1J_{C–F}$ = 189.0 Hz), 62.31, 55.50, 35.53 (d, $^2J_{C–F}$ = 20.2 Hz), 32.00, 28.99.

^{19}F{H} NMR (376 MHz, $CDCl_3$) δ -188.05.

^{19}F NMR (376 MHz, $CDCl_3$) δ -188.06 (dddd, J = 49.5, 29.2, 26.5, 6.4 Hz).

HRMS (ESI/QTOF) m/z: $[M + Na]^+$ Calcd for $C_{15}H_{20}FNNaO_3^+$ 304.1325; Found 304.1322.

$[\alpha]_D^{23}$ = +61.9 (c = 1.2 in $CHCl_3$).

FTIR (neat): $\tilde{\nu}$ = 3321, 1660, 1599, 1245, 1049, 821, 636 cm^{-1}.

HPLC: The enantiomeric ratio (96.5:3.5) was determined *via* HPLC analysis using a CHIRALCEL® IA column, with hexane:isopropanol = 80:20 at a flow rate 1.0 mL/min detected at 254 nm wavelength. Retention time: t_{major} = 9.8 min and t_{minor} = 12.4 min.

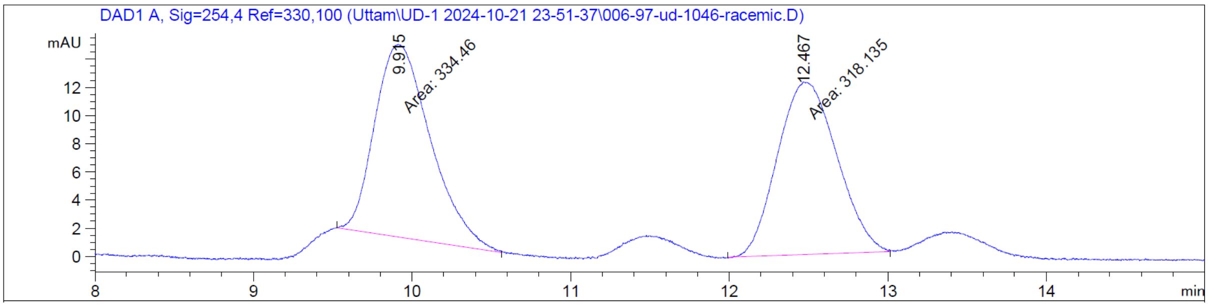

Peak #	RetTime [min]	Type	Width [min]	Area [mAU*s]	Height [mAU]	Area %
1	9.915	MM	0.4079	334.46017	13.66436	51.2508
2	12.467	MM	0.4331	318.13464	12.24364	48.7492

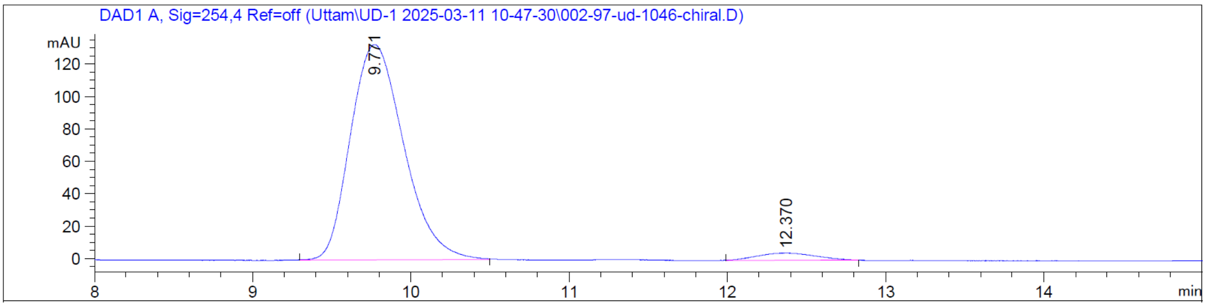

Peak #	RetTime [min]	Type	Width [min]	Area [mAU*s]	Height [mAU]	Area %
1	9.771	VV	0.2826	3106.90283	132.49533	96.4638
2	12.370	VB	0.2965	113.89539	4.50360	3.5362

(*R*,*E*)-5-(Cyclohex-1-en-1-yl)-2-fluoro-*N*-phenylpent-4-enamide (3gb)

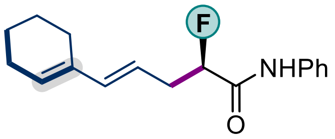

Prepared according to **GP1** with 2-bromo-2-fluoro-*N*-phenylacetamide (**2b**) (23.2 mg, 0.10 mmol, 1.0 equiv.) and 1-(propa-1,2-dien-1-yl)cyclohex-1-ene (**1g**) (24 mg, 0.20 mmol, 2.0 equiv.) (45.5 mg, 0.20 mmol, 2.0 equiv.). Automated flash column chromatography (10 g SiO_2,

gradient elution: hexane to 50% EtOAc in hexane) afforded the desired product (+) **3gb** as yellow oil (15 mg, 55%).

^{1}H NMR (400 MHz, $CDCl_3$) δ 7.94 (d, J = 6.6 Hz, 1H), 7.62 – 7.49 (m, 2H), 7.35 (t, J = 7.9 Hz, 2H), 7.20 – 7.12 (m, 1H), 6.20 (d, J = 15.6 Hz, 1H), 5.72 (t, J = 2.7 Hz, 1H), 5.54 (dt, J = 15.0, 7.2 Hz, 1H), 5.04 (ddd, J = 49.8, 7.1, 3.8 Hz, 1H), 3.00 – 2.62 (m, 2H), 2.11 (h, J = 3.7 Hz, 4H), 1.71 – 1.53 (m, 4H).

^{13}C NMR (101 MHz, $CDCl_3$) δ 167.50 (d, $^{2}J_{C–F}$ = 17.8 Hz), 138.14, 136.62, 135.17, 129.48, 129.13, 125.01, 120.12, 117.88 (d, $^{3}J_{C–F}$ = 2.9 Hz), 91.58 (d, $^{1}J_{C–F}$ = 189.3 Hz), 35.85 (d, $^{2}J_{C–F}$ = 20.0 Hz), 25.79, 24.50, 22.49, 22.41.

^{19}F{H} NMR (377 MHz, $CDCl_3$) δ -187.66.

^{19}F NMR (377 MHz, $CDCl_3$) δ -187.66 (dtd, J = 49.8, 28.4, 6.8 Hz).

HRMS (ESI/QTOF) m/z: $[M + Na]^+$ Calcd for $C_{17}H_{20}FNNaO^+$ 296.1427; Found 296.1424.

$[\alpha]_D^{23}$ = +54.9 (c = 0.75 in $CHCl_3$).

FTIR (neat): $\tilde{\nu}$ = 2927, 1690, 1601, 1546, 1260, 1135, 1019, 802, 758, 693 cm^{-1}.

HPLC: The enantiomeric ratio (97.5:2.5) was determined *via* HPLC analysis using a CHIRALCEL® IA column, with hexane:isopropanol = 95:5 at a flow rate 1.0 mL/min detected at 254 nm wavelength. Retention time: t_{major} = 9.2 min and t_{minor} = 11.3 min.

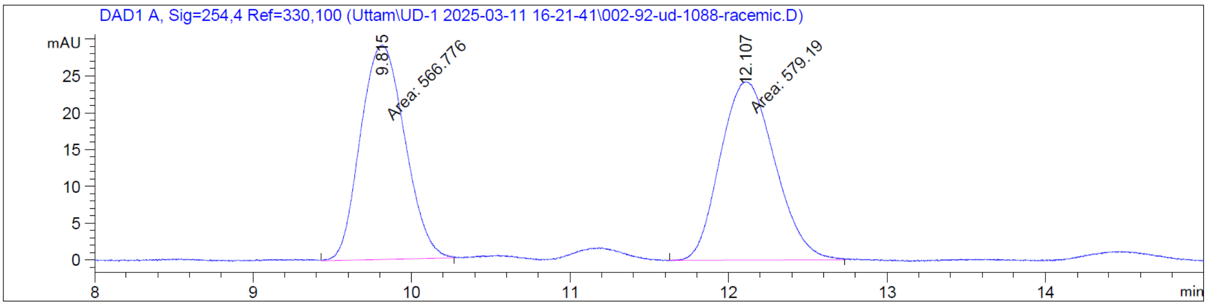

Peak #	RetTime [min]	Type	Width [min]	Area [mAU*s]	Height [mAU]	Area %
1	9.815	MM	0.3241	566.77600	29.14701	49.4583
2	12.107	MM	0.3988	579.19043	24.20703	50.5417

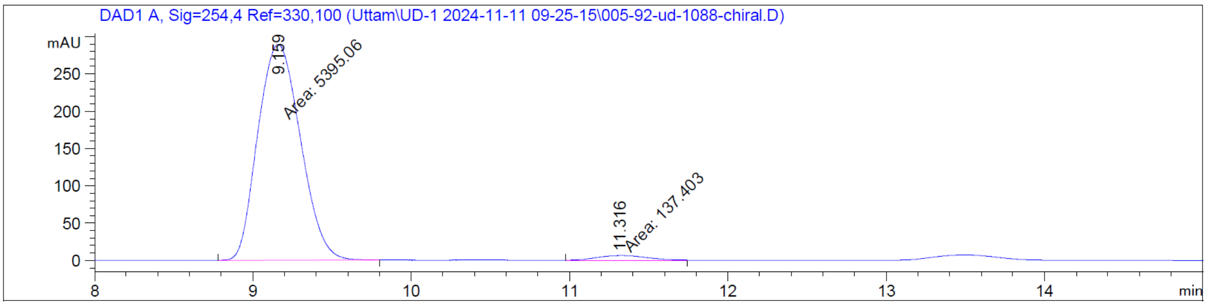

Peak #	RetTime [min]	Type	Width [min]	Area [mAU*s]	Height [mAU]	Area %
1	9.159	MM	0.3111	5395.05566	289.02002	97.5164
2	11.316	MM	0.3634	137.40254	6.30121	2.4836

Dimethyl (*R*,*E*)-2-allyl-2-(6-fluoro-7-((4-methoxyphenyl)amino)-7-oxohept-3-en-1-yl)malonate (3ha)

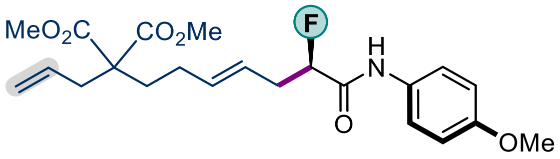

A modified procedure **GP1** was followed with 2-bromo-2-fluoro-*N*-(4-methoxyphenyl)acetamide (**2a**) (26.2 mg, 0.10 mmol, 1.0 equiv.) and dimethyl 2-allyl-2-(penta-3,4-dien-1-yl)malonate (**1h**) (47.7 mg, 0.20 mmol, 2.0 equiv.) at 0 °C. Automated flash column chromatography (10 g SiO_2, gradient elution: hexane to 50% EtOAc in hexane) afforded the desired product (+) **3ha** as yellow oil (21.5 mg, 51%). 10% other isomers were observed.

^{1}H NMR (400 MHz, $CDCl_3$) δ 7.86 (d, J = 6.4 Hz, 1H), 7.52 – 7.39 (m, 2H), 6.91 – 6.85 (m, 2H), 5.75 – 5.32 (m, 3H), 5.15 – 4.89 (m, 3H), 3.83 – 3.78 (m, 3H), 3.70 (s, 6H), 2.80 – 2.55 (m, 4H), 2.03 – 1.88 (m, 4H).

^{13}C NMR (101 MHz, $CDCl_3$) (one carbon less due to overlap): δ 171.54, 171.52, 167.21 (d, $^{2}J_{C–F}$ = 17.8 Hz), 156.89, 134.16, 132.31, 129.67, 123.33 (d, $^{3}J_{C–F}$ = 2.8 Hz), 121.86, 119.04, 114.26, 91.42 (d, $^{1}J_{C–F}$ = 189.0 Hz), 57.35, 55.50, 52.38, 37.21, 35.49 (d, $^{2}J_{C–F}$ = 20.2 Hz), 32.00, 27.29.

^{19}F{H} NMR (376 MHz, $CDCl_3$) δ -188.03.

^{19}F NMR (376 MHz, $CDCl_3$) δ -188.03 (dtd, J = 49.8, 28.5, 6.5 Hz).

HRMS (ESI/QTOF) m/z: $[M + Na]^+$ Calcd for $C_{22}H_{28}FNNaO_6^+$ 444.1798; Found 444.1806.

$[\alpha]_D^{23}$ = +52.5 (c = 0.50 in $CHCl_3$).

FTIR (neat): $\tilde{\nu}$ = 2925, 1729, 1675, 1538, 1512, 1242, 830 cm^{-1}.

HPLC: The enantiomeric ratio (94:6) was determined *via* HPLC analysis using a CHIRALCEL® IA column, with hexane:isopropanol = 90:10 at a flow rate 1.0 mL/min detected at 254 nm wavelength. Retention time: t_{major} = 18.3 min and t_{minor} = 23.7 min.

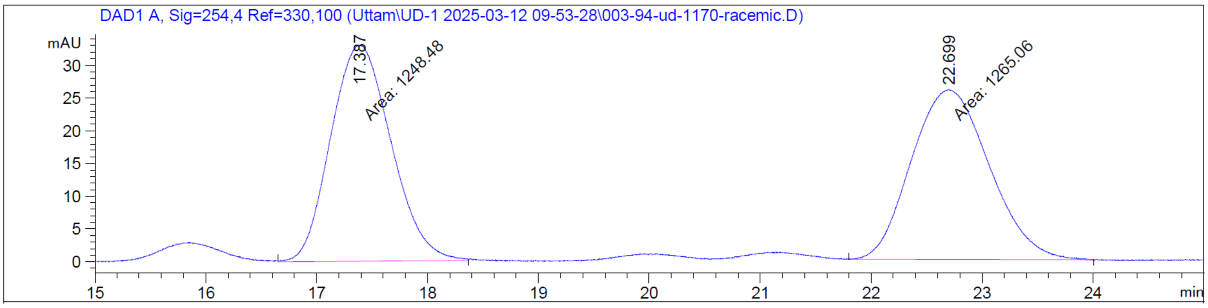

Peak #	RetTime [min]	Type	Width [min]	Area [mAU*s]	Height [mAU]	Area %
1	17.387	MM	0.6298	1248.48108	33.03766	49.6703
2	22.699	MM	0.8133	1265.05701	25.92346	50.3297

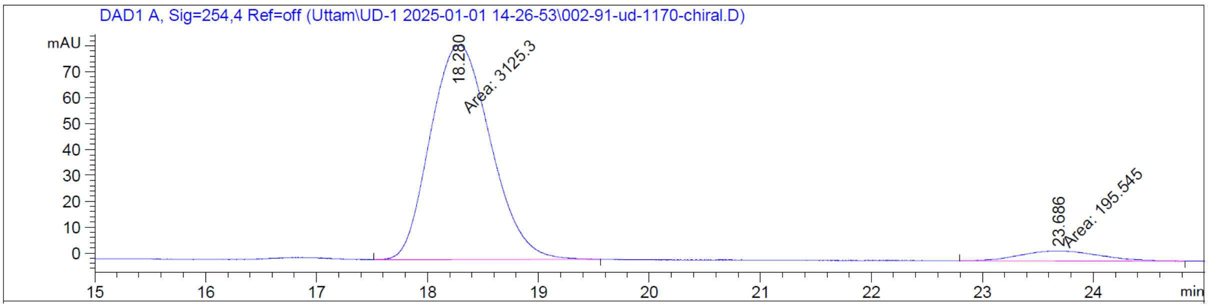

Peak #	RetTime [min]	Type	Width [min]	Area [mAU*s]	Height [mAU]	Area %
1	18.280	MM	0.6276	3125.30200	82.99820	94.1116
2	23.686	MM	0.8353	195.54451	3.90191	5.8884

(*R*,*E*)-2-fluoro-*N*,11-diphenylundec-4-en-10-ynamide (3ib)

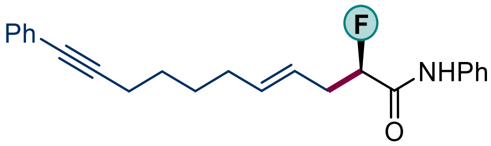

A modified procedure **GP1** was followed with 2-bromo-2-fluoro-*N*-phenylacetamide (23.2 mg, 0.10 mmol, 1.0 equiv.) (**2b**) and octa-6,7-dien-1-yn-1-ylbenzene (**1i**) (36.5 mg, 0.20 mmol, 2.0 equiv.) at 0 °C. Automated flash column chromatography (10 g SiO_2, gradient elution: hexane to 20% EtOAc in hexane) afforded the desired product (–) **3ib** as yellow oil (22.5 mg, 65%).

^{1}H NMR (400 MHz, $CDCl_3$) δ 7.87 (d, J = 6.7 Hz, 1H), 7.58 – 7.44 (m, 2H), 7.37 – 7.25 (m, 4H), 7.22 – 7.18 (m, 3H), 7.12 – 7.05 (m, 1H), 5.67 – 5.53 (m, 1H), 5.42 (dtt, J = 15.3, 6.9, 1.4 Hz, 1H), 4.96 (ddd, J = 49.7, 6.7, 3.9 Hz, 1H), 2.81 – 2.49 (m, 2H), 2.29 (t, J = 6.7 Hz, 2H), 2.07 – 1.96 (m, 2H), 1.54 – 1.40 (m, 4H).

^{13}C NMR (101 MHz, $CDCl_3$) δ 167.50 (d, $^2J_{C–F}$ = 18.2 Hz), 136.63, 135.52, 131.54, 129.13, 128.19, 127.51, 124.97, 124.03, 122.54 (d, $^3J_{C–F}$ = 2.8 Hz), 119.99, 91.59 (d, $^1J_{C–F}$ = 189.0 Hz), 90.15, 80.71, 35.53 (d, $^2J_{C–F}$ = 20.2 Hz), 32.08, 28.39, 28.09, 19.21.

^{19}F{H} NMR (376 MHz, $CDCl_3$) δ -188.09.

^{19}F NMR (376 MHz, $CDCl_3$) δ -188.09 (dddd, J = 49.5, 29.5, 26.8, 6.8 Hz).

HRMS (ESI/QTOF) m/z: $[M + Na]^+$ Calcd for $C_{23}H_{24}FNNaO^+$ 372.1740; Found 372.1735.

$[\alpha]_D^{23}$ = -2.0 (c = 1.00 in $CHCl_3$).

FTIR (neat): $\tilde{\nu}$ = 1679, 1443, 1387, 989, 794, 616 cm^{-1}.

HPLC: The enantiomeric ratio (95.5:4.5) was determined *via* HPLC analysis using a CHIRALCEL® AD-H column, with hexane:isopropanol = 98:2 at a flow rate 1.0 mL/min detected at 254 nm wavelength. Retention time: t_{major} = 23.1 min and t_{minor} = 26.4 min.

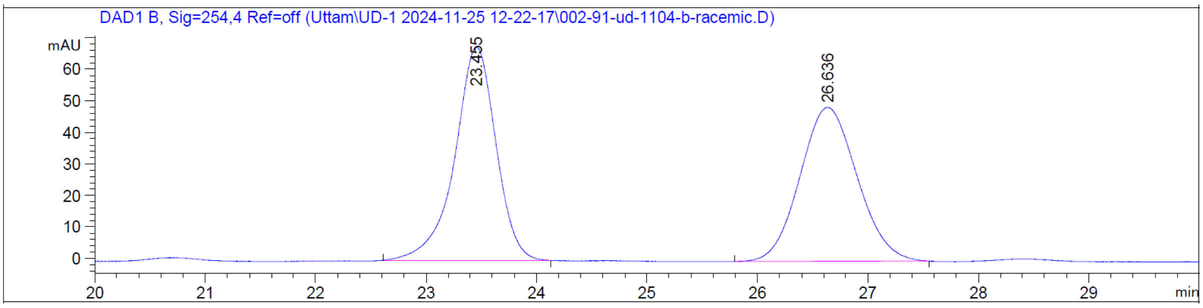

Peak #	RetTime [min]	Type	Width [min]	Area [mAU*s]	Height [mAU]	Area %
1	23.455	BV	0.3066	1765.30164	67.84731	50.0196
2	26.636	BB	0.4223	1763.91956	48.91021	49.9804

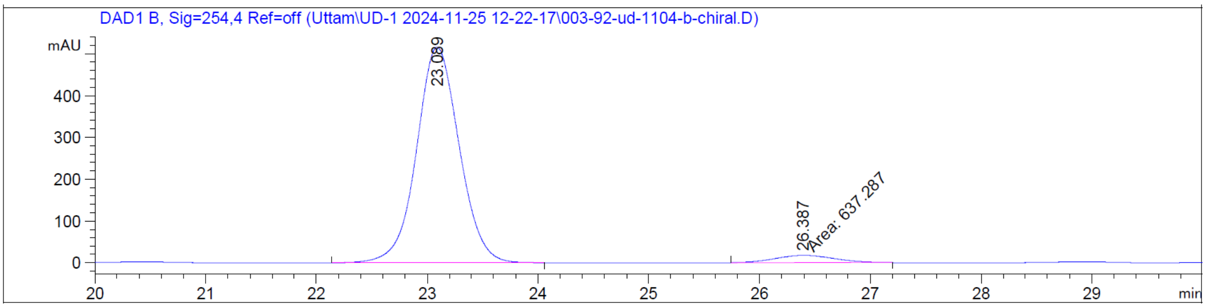

Peak #	RetTime [min]	Type	Width [min]	Area [mAU*s]	Height [mAU]	Area %
1	23.089	VV	0.3186	1.38085e4	515.27112	95.5884
2	26.387	MM	0.6098	637.28674	17.41905	4.4116

(*R*,*E*)-2-Fluoro-*N*,5-diphenylpent-4-enamide (3jb)

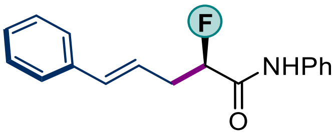

A modified procedure **GP1** was followed with 2-bromo-2-fluoro-*N*-phenylacetamide (**2b**) (23.2 mg, 0.10 mmol, 1.0 equiv.) and propa-1,2-dien-1-ylbenzene (**1j**) (23.2 mg, 0.20 mmol, 2.0 equiv.) at 0 °C for 24 h. Automated flash column chromatography (10 g SiO_2, gradient elution: hexane to 15% EtOAc in hexane) afforded the desired product (+) **3jb** as colourless oil (15 mg, 56%).

^{1}H NMR (400 MHz, $CDCl_3$) δ 7.98 (d, J = 6.2 Hz, 1H), 7.62 – 7.51 (m, 2H), 7.38 – 7.28 (m, 6H), 7.25 – 7.20 (m, 1H), 7.20 – 7.13 (m, 1H), 6.59 (d, J = 15.8 Hz, 1H), 6.24 (dt, J = 15.8, 7.2 Hz, 1H), 5.13 (ddd, J = 49.6, 7.0, 3.8 Hz, 1H), 3.09 – 2.79 (m, 2H).

^{13}C NMR (101 MHz, $CDCl_3$) δ 167.30 (d, $^2J_{C\text{–}F}$ = 18.2 Hz), 136.86, 136.54, 134.56, 129.15, 128.57, 127.61, 126.29, 125.09, 122.38 (d, $^3J_{C\text{–}F}$ = 2.8 Hz), 120.14, 91.32 (d, $^1J_{C\text{–}F}$ = 189.7 Hz), 35.92 (d, $^2J_{C\text{–}F}$ = 20.2 Hz).

^{19}F{H} NMR (376 MHz, $CDCl_3$) δ -187.80.

^{19}F NMR (376 MHz, $CDCl_3$) δ -187.80 (dtd, J = 49.8, 28.0, 6.8 Hz).

HRMS (ESI/QTOF) m/z: $[M + H]^+$ Calcd for $C_{17}H_{17}FNO^+$ 270.1294; Found 270.1291.

$[\alpha]_D^{23}$ = +73.2 (c = 0.50 in $CHCl_3$).

FTIR (neat): $\tilde{\nu}$ = 3343, 1666, 1535, 1415, 1070, 714, 691 cm^{-1}.

HPLC: The enantiomeric ratio (93.5:6.5) was determined *via* HPLC analysis using a CHIRALCEL® IA column, with hexane:isopropanol = 99:1 at a flow rate 1.0 mL/min detected at 254 nm wavelength. Retention time: t_{major} = 18.1 min and t_{minor} = 26.0 min.

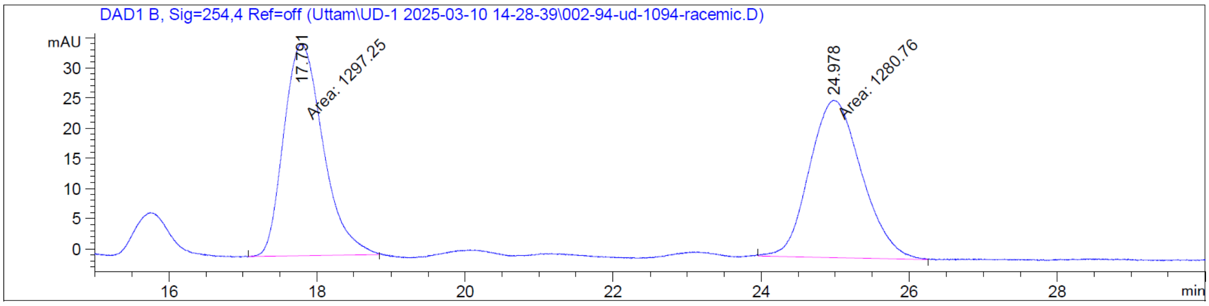

Peak #	RetTime [min]	Type	Width [min]	Area [mAU*s]	Height [mAU]	Area %
1	17.791	MM	0.6162	1297.25244	35.08635	50.3199
2	24.978	MM	0.8177	1280.75793	26.10490	49.6801

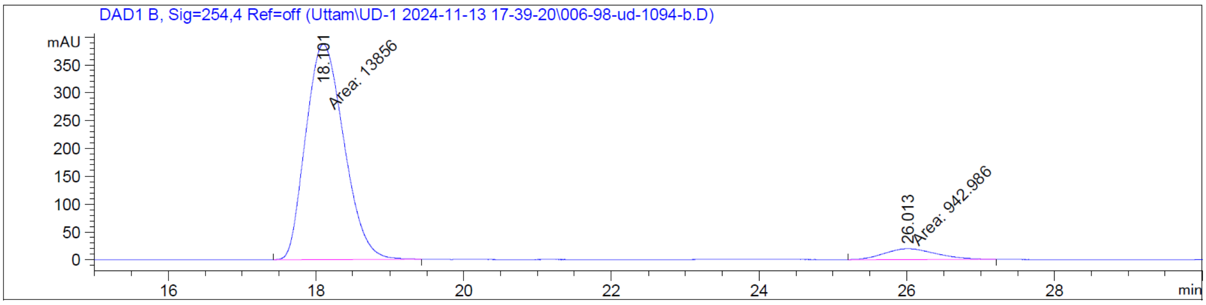

Peak #	RetTime [min]	Type	Width [min]	Area [mAU*s]	Height [mAU]	Area %
1	18.101	MM	0.5975	1.38560e4	386.52106	93.6280
2	26.013	MM	0.8139	942.98560	19.31034	6.3720

(*R*,*E*)-5-(4-(Dimethylamino)phenyl)-2-fluoro-*N*-(4-methoxyphenyl)pent-4-enamide (3ka)

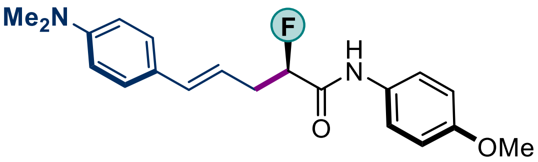

A modified procedure **GP1** was followed with 2-bromo-2-fluoro-*N*-(4-methoxyphenyl)acetamide (**2a**) (26.2 mg, 0.10 mmol, 1.0 equiv.) and *N*,*N*-dimethyl-4-(propa-1,2-dien-1-yl)aniline (**1k**) (31.8 mg, 0.20 mmol, 2.0 equiv.) at 0 °C for 24 h. Automated flash

column chromatography (10 g SiO_2, gradient elution: hexane to 30% EtOAc in hexane) afforded the desired product (+) **3ka** as orange solid (17 mg, 50%).

^{1}H NMR (400 MHz, $CDCl_3$) δ 7.87 (d, J = 6.5 Hz, 1H), 7.45 (d, J = 9.0 Hz, 2H), 7.25 (d, J = 8.8 Hz, 2H), 6.91 – 6.84 (m, 2H), 6.69 – 6.62 (m, 2H), 6.48 (d, J = 15.8 Hz, 1H), 6.01 (dt, J = 15.8, 7.2 Hz, 1H), 5.09 (ddd, J = 49.6, 6.8, 3.8 Hz, 1H), 3.80 (s, 3H), 3.03 – 2.74 (m, 8H).

^{13}C NMR (101 MHz, $CDCl_3$) δ 167.35 (d, $^{2}J_{C–F}$ = 18.2 Hz), 156.90, 150.10, 134.29, 129.68, 127.20, 125.51, 121.97, 117.87 (d, $^{3}J_{C–F}$ = = 2.2 Hz), 114.26, 112.43, 91.65 (d, $^{1}J_{C–F}$ = 189.0 Hz), 55.50, 40.53, 36.04 (d, $^{2}J_{C–F}$ = 20.2 Hz).

$^{19}F\{H\}$ NMR (376 MHz, $CDCl_3$) δ -187.95.

^{19}F NMR (376 MHz, $CDCl_3$) δ -187.95 (dtd, J = 49.8, 28.3, 6.7 Hz).

HRMS (ESI/QTOF) m/z: $[M + H]^+$ Calcd for $C_{20}H_{24}FN_2O_2^+$ 343.1822; Found 343.1822.

$[\alpha]_D^{23}$ = +52.8 (c = 0.33 in $CHCl_3$).

FTIR (neat): $\tilde{\nu}$ = 3314, 1663, 1514, 1260, 1092, 1022, 799 cm^{-1}.

HPLC: The enantiomeric ratio (95.5:4.5) was determined *via* HPLC analysis using a CHIRALCEL® IA column, with hexane:isopropanol = 90:10 at a flow rate 1.0 mL/min detected at 254 nm wavelength. Retention time: t_{major} = 20.6 min and t_{minor} = 28.1 min.

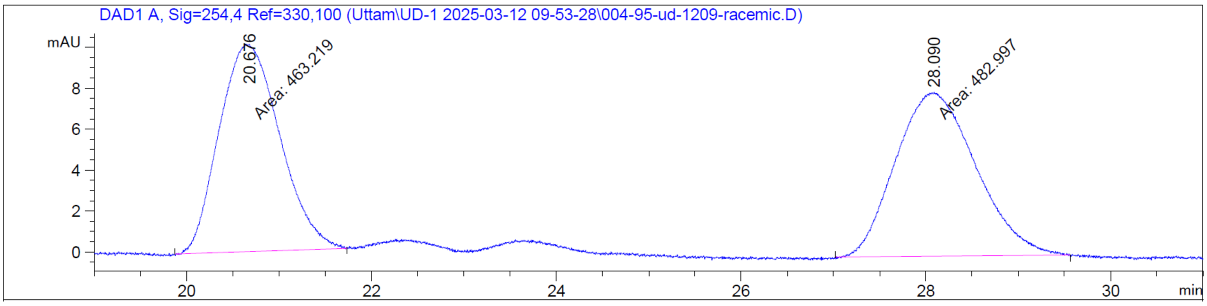

Peak #	RetTime [min]	Type	Width [min]	Area [mAU*s]	Height [mAU]	Area %
1	20.676	MM	0.7617	463.21863	10.13512	48.9549
2	28.090	MM	1.0036	482.99695	8.02088	51.0451

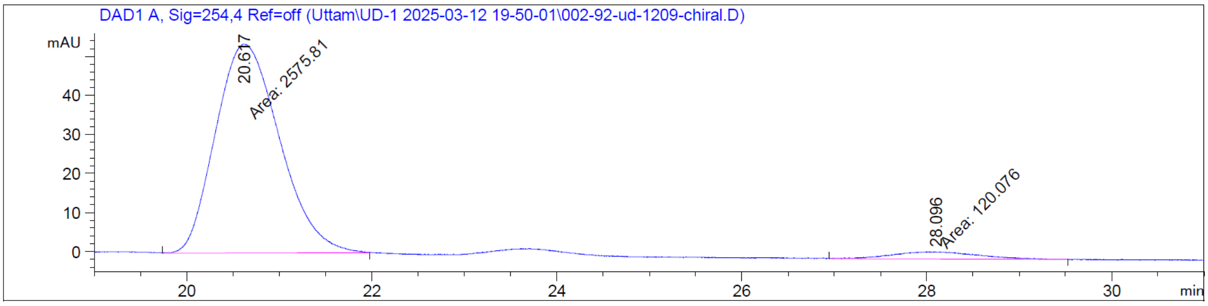

Peak #	RetTime [min]	Type	Width [min]	Area [mAU*s]	Height [mAU]	Area %
1	20.617	MM	0.8024	2575.81177	53.50052	95.5460
2	28.096	MM	1.0776	120.07567	1.85712	4.4540

(*R*,*E*)-2-Fluoro-*N*-phenyl-5-(4,4,5,5-tetramethyl-1,3,2-dioxaborolan-2-yl)pent-4-enamide (3lb)

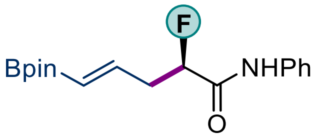

Prepared according to **GP1** with 2-bromo-2-fluoro-*N*-phenylacetamide (**2b**) (23.2 mg, 0.10 mmol, 1.0 equiv.) and 2-allenyl-4,4,5,5-tetramethyl-1,3,2-dioxaborolane (**1l**) (35.9 µL, 0.20 mmol, 2.0 equiv.). Automated flash column chromatography (10 g SiO_2, gradient elution: hexane to 20% EtOAc in hexane) afforded the desired product (+) **3lb** as yellow oil (19 mg, 60%).

^{1}H NMR (400 MHz, $CDCl_3$) δ 7.96 (d, J = 6.7 Hz, 1H), 7.59 – 7.50 (m, 2H), 7.41 – 7.30 (m, 2H), 7.21 – 7.09 (m, 1H), 6.63 (dt, J = 17.9, 6.6 Hz, 1H), 5.66 (dt, J = 17.8, 1.6 Hz, 1H), 5.09 (ddd, J = 49.7, 8.2, 3.4 Hz, 1H), 3.09 – 2.86 (m, 1H), 2.86 – 2.64 (m, 1H), 1.26 (s, 12H).

^{13}C NMR (101 MHz, $CDCl_3$) δ 167.22 (d, $^{2}J_{C–F}$ = 17.8 Hz), 145.81 (d, $^{3}J_{C–F}$ = 2.2 Hz), 136.57, 129.13, 125.05, 120.11, 90.75 (d, $^{1}J_{C–F}$ = 189.7 Hz), 83.34, 38.52 (d, $^{2}J_{C–F}$ = 20.2 Hz), 24.79, 24.76.

^{19}F{H} NMR (376 MHz, $CDCl_3$) δ -187.43.

^{19}F NMR (376 MHz, $CDCl_3$) δ -187.43 (dddd, J = 49.5, 31.7, 24.7, 6.6 Hz).

HRMS (ESI/QTOF) m/z: $[M + Na]^+$ Calcd for $C_{17}H_{23}BFNNaO_3^+$ 342.1656; Found 342.1654.

$[\alpha]_D^{23}$ = +5.3 (c = 0.75 in $CHCl_3$).

FTIR (neat): $\tilde{\nu}$ = 3325, 1679, 1443, 1366, 1144, 796, 755 cm^{-1}.

HPLC: The enantiomeric ratio (94:6) was determined *via* HPLC analysis using a CHIRALCEL® IA column, with hexane:isopropanol = 95:5 at a flow rate 1.0 mL/min detected at 254 nm wavelength. Retention time: t_{major} = 10.1 min and t_{minor} = 11.6 min.

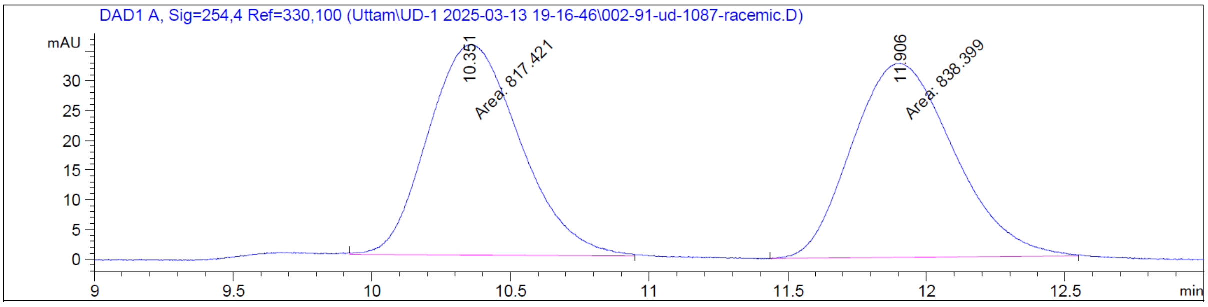

Peak #	RetTime [min]	Type	Width [min]	Area [mAU*s]	Height [mAU]	Area %
1	10.351	MM	0.3848	817.42090	35.40590	49.3665
2	11.906	MM	0.4280	838.39948	32.65115	50.6335

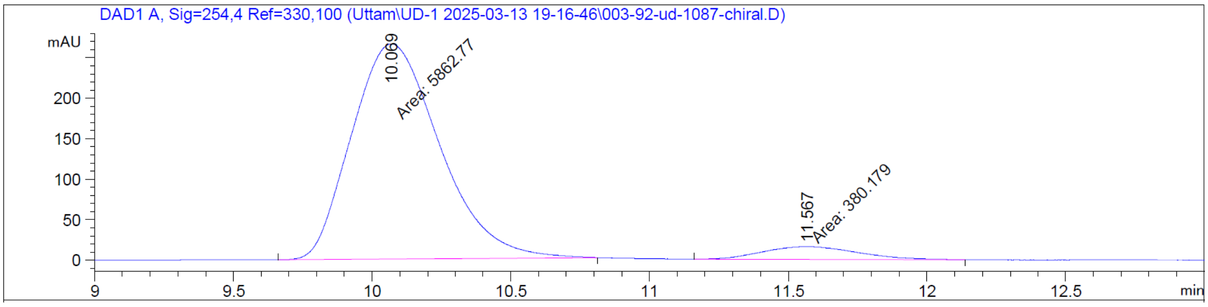

Peak #	RetTime [min]	Type	Width [min]	Area [mAU*s]	Height [mAU]	Area %
1	10.069	MM	0.3676	5862.76953	265.83176	93.9103
2	11.567	MM	0.3991	380.17902	15.87682	6.0897

(*R*,*E*)-5-(Dimethyl(phenyl)silyl)-2-fluoro-*N*-(4-methoxyphenyl)pent-4-enamide (3ma)

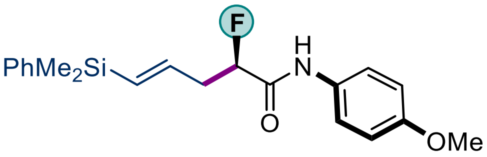

Prepared according to **GP1** with 2-bromo-2-fluoro-*N*-(4-methoxyphenyl)acetamide (**2a**) (26.2 mg, 0.10 mmol, 1.0 equiv.) and dimethyl(phenyl)(propa-1,2-dien-1-yl)silane (**1m**) (34.9 mg, 0.20 mmol, 2.0 equiv.). Automated flash column chromatography (10 g SiO_2, gradient elution: hexane to 20% EtOAc in hexane) afforded the desired product (+) **3ma** as yellow oil (23.5 mg, 66%).

^{1}H NMR (400 MHz, $CDCl_3$) δ 7.86 (d, J = 6.4 Hz, 1H), 7.54 – 7.22 (m, 7H), 6.99 – 6.78 (m, 2H), 6.32 – 5.90 (m, 2H), 5.09 (ddd, J = 49.7, 7.1, 3.7 Hz, 1H), 3.81 (s, 3H), 3.04 – 2.60 (m, 2H), 0.33 (s, 6H).

^{13}C NMR (101 MHz, $CDCl_3$) δ 167.14 (d, $^{2}J_{C–F}$ = 18.2 Hz), 156.93, 140.57 (d, $^{3}J_{C–F}$ = 2.4 Hz), 138.40, 133.93, 133.80, 129.61, 129.01, 127.79, 121.92, 114.28, 91.11 (d, $^{1}J_{C–F}$ = 189.4 Hz), 55.51, 39.40 (d, $^{2}J_{C–F}$ = 19.8 Hz), -2.59, -2.64.

^{19}F{H} NMR (376 MHz, $CDCl_3$) δ -187.83.

^{19}F NMR (376 MHz, $CDCl_3$) δ -187.83 (dtd, J = 49.8, 28.5, 6.4 Hz).

HRMS (ESI/QTOF) m/z: $[M + Na]^+$ Calcd for $C_{20}H_{24}FNNaO_2Si^+$ 380.1458; Found 380.1455.

$[\alpha]_D^{23}$ = +70.5 (c = 1.20 in $CHCl_3$).

FTIR (neat): $\tilde{\nu}$ = 2954, 1670, 1600, 1534, 1512, 1466, 1233, 758 cm^{-1}.

HPLC: The enantiomeric ratio (92.5:7.5) was determined *via* HPLC analysis using a CHIRALCEL® IA column, with hexane:isopropanol = 95:5 at a flow rate 1.0 mL/min detected at 254 nm wavelength. Retention time: t_{major} = 13.9 min and t_{minor} = 17.1 min.

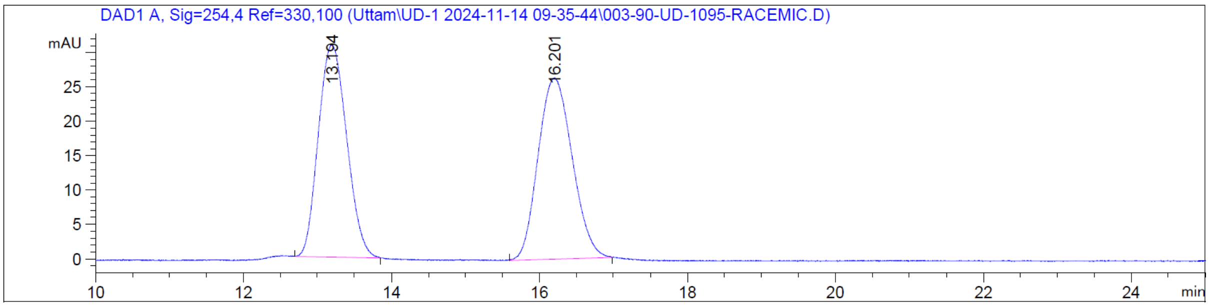

Peak #	RetTime [min]	Type	Width [min]	Area [mAU*s]	Height [mAU]	Area %
1	13.194	BB	0.3123	822.70605	30.88362	49.3935
2	16.201	BB	0.3749	842.91046	26.33364	50.6065

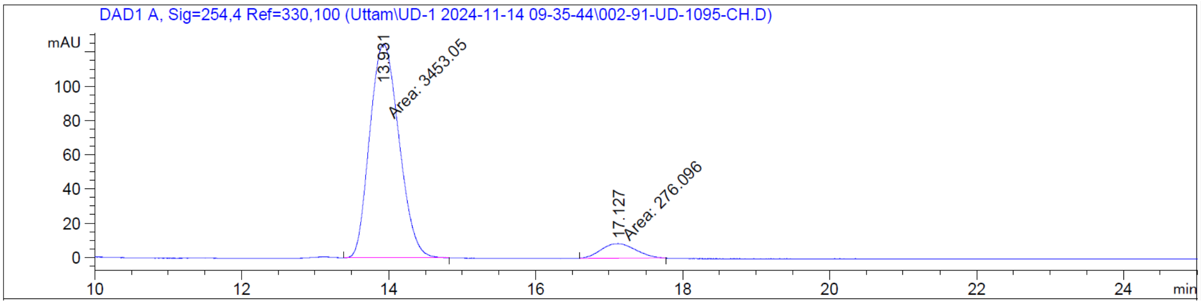

Peak #	RetTime [min]	Type	Width [min]	Area [mAU*s]	Height [mAU]	Area %
1	13.931	MM	0.4605	3453.04590	124.97083	92.5963
2	17.127	MM	0.5383	276.09586	8.54773	7.4037

(*R*)-4-Cyclohexylidene-2-fluoro-N-phenylbutanamide (3nb)

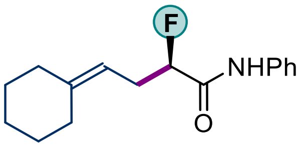

A modified procedure **GP1** was followed with 2-bromo-2-fluoro-*N*-phenylacetamide (**2b**) (23.2 mg, 0.10 mmol, 1.0 equiv.) and ethenylidenecyclohexane (**1n**) (90%, 29.8 μL, 0.20 mmol, 2.0 equiv.) at -35 °C. Automated flash column chromatography (10 g SiO_2, gradient elution: hexane to 20% EtOAc in hexane) afforded the desired product (+) **3nb** as a colourless oil (18 mg, 70%).

^{1}H NMR (400 MHz, $CDCl_3$) δ 7.86 (d, J = 6.7 Hz, 1H), 7.60 – 7.40 (m, 2H), 7.34 – 7.22 (m, 2H), 7.08 (td, J = 7.3, 1.2 Hz, 1H), 5.09 (t, J = 7.4 Hz, 1H), 4.93 (ddd, J = 49.8, 6.6, 4.0 Hz, 1H), 2.81 – 2.53 (m, 2H), 2.05 (dt, J = 22.7, 5.5 Hz, 4H), 1.43 (dt, J = 14.3, 6.1 Hz, 6H).

^{13}C NMR (101 MHz, $CDCl_3$) δ 167.77 (d, $^2J_{C-F}$ = 18.2 Hz), 144.82, 136.73, 129.12, 124.89, 119.97, 113.02 (d, $^3J_{C-F}$ = 2.9 Hz), 92.03 (d, $^1J_{C-F}$ = 188.6 Hz), 37.24, 30.25 (d, $^2J_{C-F}$ = 20.0 Hz), 28.91, 28.60, 27.71, 26.74.

^{19}F{H} NMR (377 MHz, $CDCl_3$) δ -188.10.

^{19}F NMR (377 MHz, $CDCl_3$) δ -188.10 (dddd, J = 49.8, 29.7, 27.4, 6.8 Hz).

HRMS (ESI/QTOF) m/z: $[M + Na]^+$ Calcd for $C_{16}H_{20}FNNaO^+$ 284.1427; Found 284.1421.

$[\alpha]_D^{23}$ = +41.1 (c = 1.00 in $CHCl_3$).

FTIR (neat): $\tilde{\nu}$ = 2974, 1676, 1535, 1444, 1113, 751, 691 cm^{-1}.

HPLC: The enantiomeric ratio (97:3) was determined *via* HPLC analysis using a CHIRALCEL® IA column, with hexane:isopropanol = 95:5 at a flow rate 1.0 mL/min detected at 254 nm wavelength. Retention time: t_{major} = 7.9 min and t_{minor} = 9.2 min.

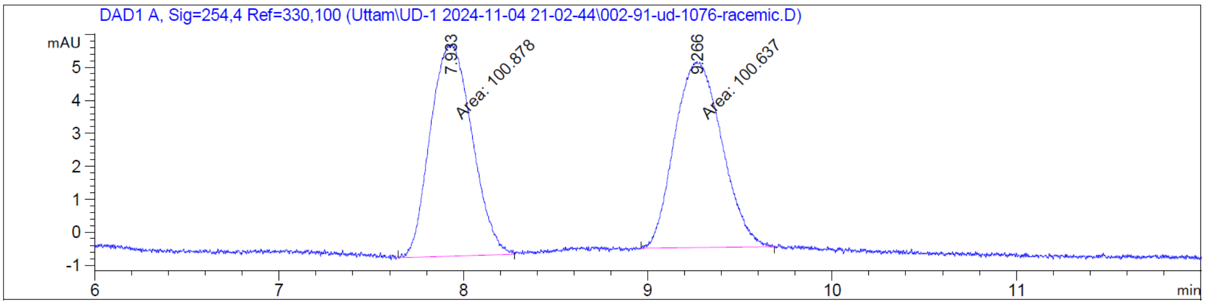

Peak #	RetTime [min]	Type	Width [min]	Area [mAU*s]	Height [mAU]	Area %
1	7.933	MM	0.2620	100.87825	6.41789	50.0600
2	9.266	MM	0.2978	100.63663	5.63181	49.9400

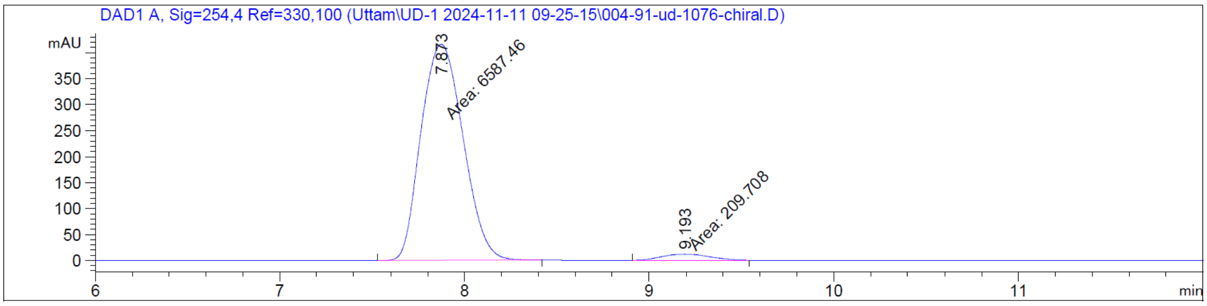

Peak #	RetTime [min]	Type	Width [min]	Area [mAU*s]	Height [mAU]	Area %
1	7.873	MM	0.2638	6587.46240	416.21210	96.9148
2	9.193	MM	0.2930	209.70834	11.92845	3.0852

(*R*)-2-Fluoro-5-methyl-*N*-phenylhex-4-enamide (3ob)

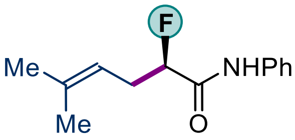

A modified procedure **GP1** was followed with 2-bromo-2-fluoro-*N*-phenylacetamide (**2b**) (23.2 mg, 0.10 mmol, 1.0 equiv.) and 3-methylbuta-1,2-diene (**1o**) (19.6 µL, 0.20 mmol, 2.0 equiv.) (**1n**) (90%, 29.8 µL, 0.20 mmol, 2.0 equiv.) at -35 °C. Automated flash column chromatography (10 g SiO_2, gradient elution: hexane to 20% EtOAc in hexane) afforded the desired product (+) **3ob** as a colourless oil (14.2 mg, 64%).

^{1}H NMR (400 MHz, $CDCl_3$) δ 8.17 – 7.77 (m, 1H), 7.56 (dd, J = 8.3, 1.3 Hz, 2H), 7.35 (t, J = 7.9 Hz, 2H), 7.16 (t, J = 7.5 Hz, 1H), 5.22 (tt, J = 7.2, 1.6 Hz, 1H), 5.01 (ddd, J = 49.7, 6.9, 4.0 Hz, 1H), 2.73 (dddq, J = 44.8, 29.7, 15.2, 6.8 Hz, 2H), 1.73 (s, 3H), 1.66 (s, 3H).

^{13}C NMR (101 MHz, $CDCl_3$) δ 167.78 (d, $^{2}J_{C–F}$ = 18.2 Hz), 136.73, 136.64, 129.12, 124.92, 120.02, 116.57 (d, $^{3}J_{C–F}$ = 2.8 Hz), 91.94 (d, $^{1}J_{C–F}$ = 188.6 Hz), 31.22 (d, $^{2}J_{C–F}$ = 20.2 Hz), 25.85, 17.99.

^{19}F{H} NMR (376 MHz, $CDCl_3$) δ -187.88.

^{19}F NMR (376 MHz, $CDCl_3$) δ -187.88 (dtd, J = 49.5, 28.5, 6.5 Hz).

HRMS (ESI/QTOF) m/z: $[M + H]^+$ Calcd for $C_{13}H_{17}FNO^+$ 222.1294; Found 222.1289.

$[\alpha]_D^{23}$ = +38.0 (c = 1.00 in $CHCl_3$).

FTIR (neat): $\tilde{\nu}$ = 3312, 2919, 1674, 1537, 1445, 1052, 754, 692 cm^{-1}.

HPLC: The enantiomeric ratio (97:3) was determined *via* HPLC analysis using a CHIRALCEL® IA column, with hexane:isopropanol = 95:5 at a flow rate 1.0 mL/min detected at 254 nm wavelength. Retention time: t_{major} = 7.6 min and t_{minor} = 9.0 min.

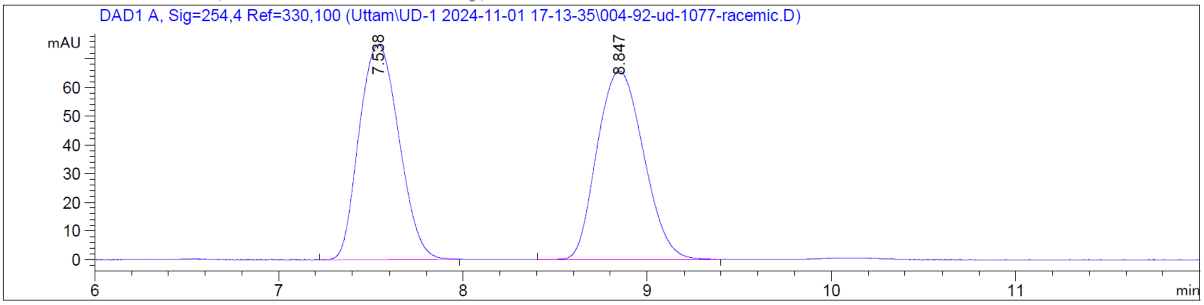

Peak #	RetTime [min]	Type	Width [min]	Area [mAU*s]	Height [mAU]	Area %
1	7.538	BV	0.1898	1126.94177	74.99649	49.4257
2	8.847	BB	0.2106	1153.13135	65.74032	50.5743

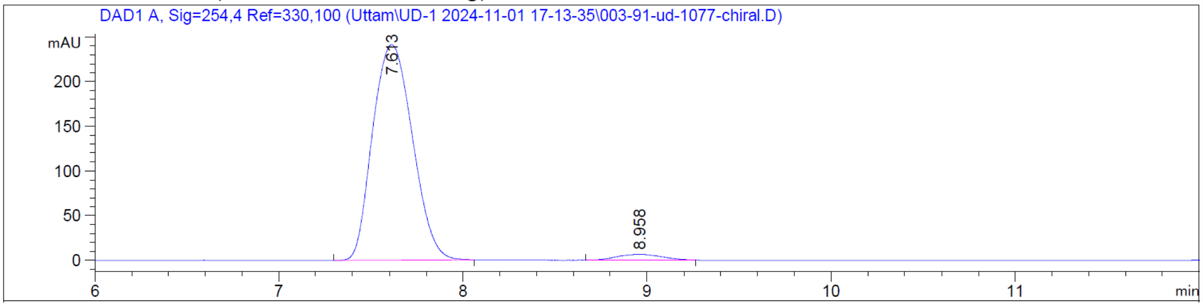

Peak #	RetTime [min]	Type	Width [min]	Area [mAU*s]	Height [mAU]	Area %
1	7.613	BB	0.2388	3626.81030	241.38690	97.0347
2	8.958	BV	0.1996	110.83330	6.56106	2.9653

(*R*,*E*)-5-Cyclohexyl-2-fluoro-*N*-(4-methoxyphenyl)pent-4-enamide (3aa)

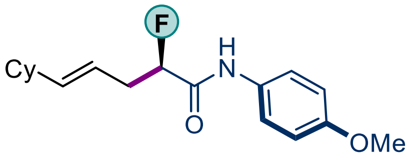

Prepared according to **GP1** with 2-bromo-2-fluoro-*N*-(4-methoxyphenyl)acetamide (**2a**) (26.2 mg, 0.10 mmol, 1.0 equiv.) and propa-1,2-dienylcyclohexane (**1a**) (24.4 mg, 0.20 mmol, 2.0 equiv.). Automated flash column chromatography (10 g SiO_2, gradient elution: hexane to 20% EtOAc in hexane) afforded the desired product (+) **3aa** as a white solid (26.5 mg, 86%).

^{1}H NMR (400 MHz, $CDCl_3$) δ 7.77 (d, *J* = 6.3 Hz, 1H), 7.42 – 7.32 (m, 2H), 6.85 – 6.76 (m, 2H), 5.62 – 5.46 (m, 1H), 5.33 (dtd, *J* = 15.3, 7.0, 1.2 Hz, 1H), 4.93 (ddd, *J* = 49.7, 6.8, 3.9 Hz, 1H), 3.73 (s, 3H), 2.76 – 2.45 (m, 2H), 1.86 (dtd, *J* = 10.8, 7.1, 3.2 Hz, 1H), 1.67 – 1.55 (m, 5H), 1.21 – 0.95 (m, 5H).

^{13}C NMR (101 MHz, $CDCl_3$) δ 167.38 (d, $^{2}J_{C–F}$ = 17.8 Hz), 156.86, 141.74, 129.70, 121.86, 119.66 (d, $^{3}J_{C–F}$ = 2.8 Hz), 114.25, 91.75 (d, $^{1}J_{C–F}$ = 189.0 Hz), 55.50, 40.68, 35.64 (d, $^{2}J_{C–F}$ = 19.8 Hz), 32.90, 32.88, 26.14, 25.97.

^{19}F{H} NMR (376 MHz, $CDCl_3$) δ -188.29.

^{19}F NMR (376 MHz, $CDCl_3$) δ -188.28 (dtd, *J* = 49.8, 28.4, 6.4 Hz).

HRMS (ESI/QTOF) m/z: $[M + Na]^+$ Calcd for $C_{18}H_{24}FNNaO_2^+$ 328.1689; Found 328.1689.

$[\alpha]_D^{23}$ = +51.1 (c = 1.20 in $CHCl_3$).

FTIR (neat): $\tilde{\nu}$ = 3308, 2918, 1660, 1530, 1514, 1238, 1029, 823 cm^{-1}.

HPLC: The enantiomeric ratio (96.5:3.5) was determined *via* HPLC analysis using a CHIRALCEL® IA column, with hexane:isopropanol = 95:5 at a flow rate 1.0 mL/min detected at 254 nm wavelength. Retention time: t_{major} = 13.6 min and t_{minor} = 17.7 min.

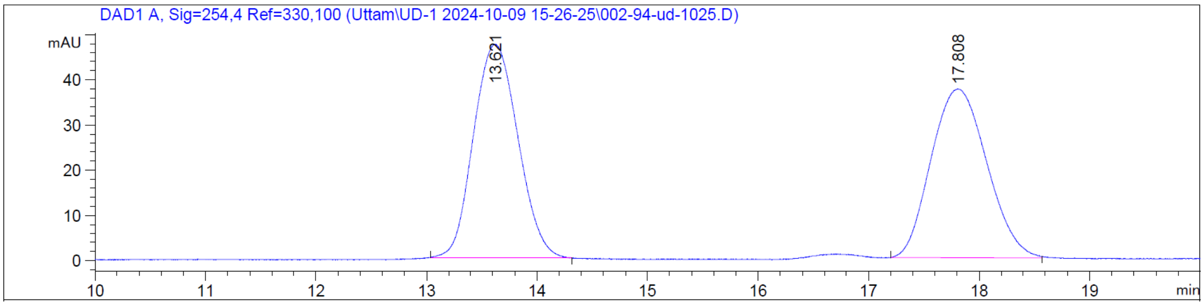

Peak #	RetTime [min]	Type	Width [min]	Area [mAU*s]	Height [mAU]	Area %
1	13.621	VB	0.3293	1322.78088	47.29702	50.2327
2	17.808	BV	0.4112	1310.52637	37.36232	49.7673

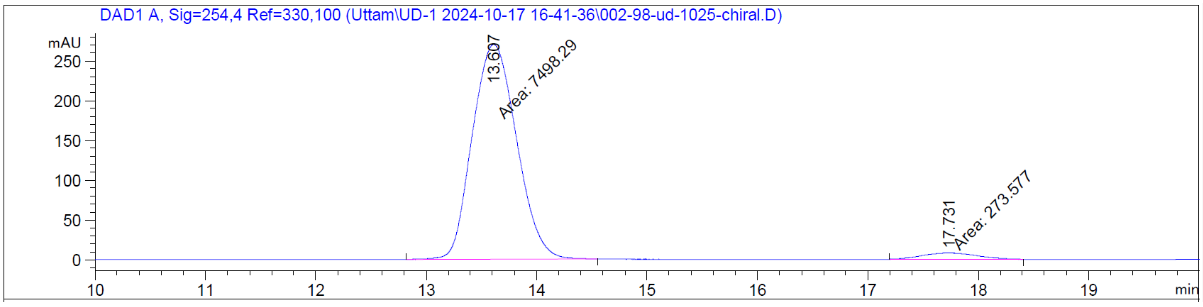

Peak #	RetTime [min]	Type	Width [min]	Area [mAU*s]	Height [mAU]	Area %
1	13.607	MM	0.4629	7498.29443	269.97394	96.4799
2	17.731	MM	0.5731	273.57678	7.95601	3.5201

(*R*,*Z*)-5-Cyclohexyl-2-fluoro-*N*-(4-methoxyphenyl)pent-4-enamide (3aa)

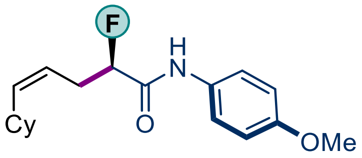

Prepared according to **GP1** with 2-bromo-2-fluoro-*N*-(4-methoxyphenyl)acetamide (**2a**) (26.2 mg, 0.10 mmol, 1.0 equiv.) and propa-1,2-dienylcyclohexane (**1a**) (24.4 mg, 0.20 mmol, 2.0 equiv.) with $NiBr_2$.DME as catalyst. Automated flash column chromatography (10 g SiO_2, gradient elution: hexane to 20% EtOAc in hexane) afforded the desired product (+) **3aa** as a

colourless oil (11 mg, 36%). *Z*:*E* ratio of the crude mixture was 86:14. Some other isomers were also observed.

^{1}H NMR (400 MHz, $CDCl_3$) δ 7.86 (d, J = 6.7 Hz, 1H), 7.54 – 7.35 (m, 2H), 6.98 – 6.81 (m, 2H), 5.50 – 5.26 (m, 2H), 5.01 (ddd, J = 49.8, 7.0, 4.0 Hz, 1H), 3.80 (s, 3H), 2.95 – 2.60 (m, 2H), 2.43 – 2.15 (m, 1H), 1.79 – 1.59 (m, 5H), 1.32 – 0.94 (m, 5H).

^{13}C NMR (101 MHz, $CDCl_3$) δ 167.33 (d, $^{2}J_{C–F}$ = 18.2 Hz), 156.85, 140.56, 129.77, 121.75, 119.56 (d, $^{3}J_{C–F}$ = 2.8 Hz), 114.25, 91.62 (d, $^{1}J_{C–F}$ = 189.0 Hz), 55.50, 36.40, 33.07, 33.00, 30.59 (d, $^{2}J_{C–F}$ = 20.2 Hz), 25.98, 25.82, 25.80.

^{19}F{H} NMR (376 MHz, $CDCl_3$) δ -188.16 (0.89H), -188.29 (0.11H).

^{19}F NMR (376 MHz, $CDCl_3$) δ -188.16 (0.89 H, dtd, J = 49.8, 28.4, 6.6 Hz).

HPLC: The enantiomeric ratio (55:45) was determined *via* HPLC analysis using a CHIRALCEL® OD-H column, with hexane:isopropanol = 95:5 at a flow rate 1.0 mL/min detected at 254 nm wavelength. Retention time: t_{major} = 39.9 min and t_{minor} = 15.7 min.

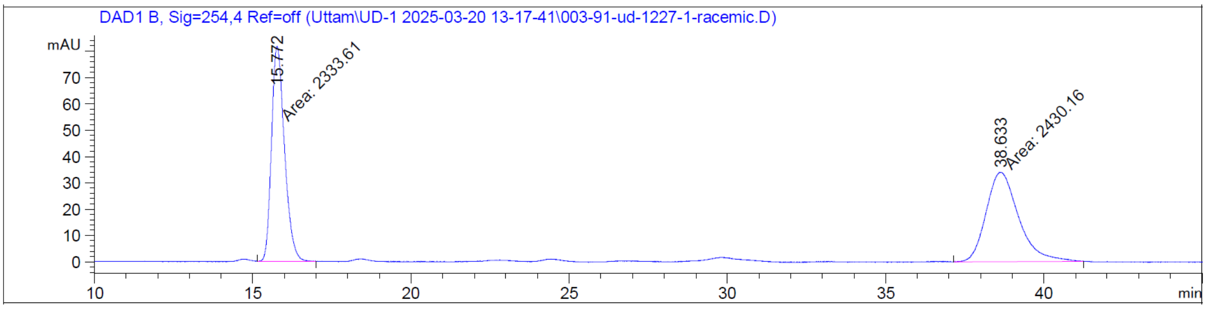

Peak #	RetTime [min]	Type	Width [min]	Area [mAU*s]	Height [mAU]	Area %
1	15.772	MM	0.4760	2333.61133	81.71055	48.9867
2	38.633	MM	1.1910	2430.15503	34.00674	51.0133

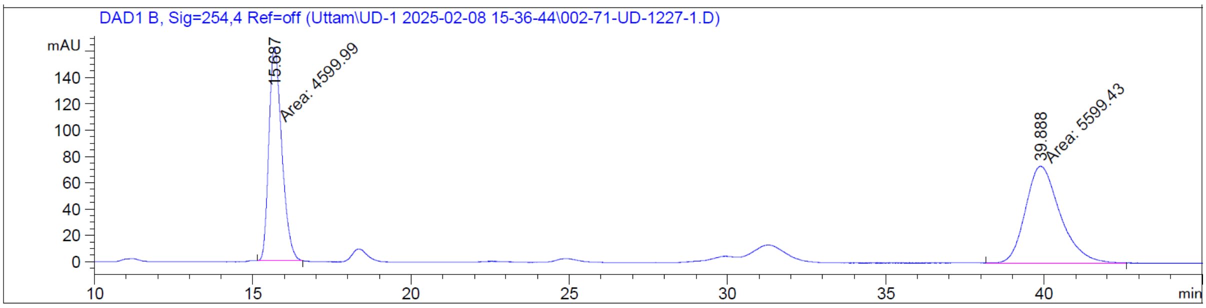

Peak #	RetTime [min]	Type	Width [min]	Area [mAU*s]	Height [mAU]	Area %
1	15.687	MM	0.4722	4599.98779	162.37187	45.1005
2	39.888	MM	1.2673	5599.43457	73.63783	54.8995

(*R*,*E*)-5-Cyclohexyl-2-fluoro-N-(4-(methylthio)phenyl)pent-4-enamide (3ac)

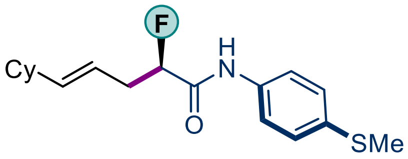

Prepared according to **GP1** with 2-bromo-2-fluoro-*N*-(4-(methylthio)phenyl)acetamide (**2c**) (27.8 mg, 0.10 mmol, 1.0 equiv.) and propa-1,2-dienylcyclohexane (**1a**) (24.4 mg, 0.20 mmol, 2.0 equiv.). Automated flash column chromatography (10 g SiO_2, gradient elution: hexane to 20% EtOAc in hexane) afforded the desired product (+) **3ac** as a white solid (25 mg, 78%).

^{1}H NMR (400 MHz, $CDCl_3$) δ 7.90 (d, J = 6.7 Hz, 1H), 7.56 – 7.42 (m, 2H), 7.32 – 7.19 (m, 2H), 5.58 (dd, J = 15.5, 6.7 Hz, 1H), 5.39 (dtd, J = 15.3, 7.0, 1.2 Hz, 1H), 5.00 (ddd, J = 49.7, 6.7, 3.8 Hz, 1H), 2.84 – 2.51 (m, 2H), 2.47 (s, 3H), 1.93 (dtd, J = 11.0, 7.4, 3.5 Hz, 1H), 1.77 – 1.56 (m, 5H), 1.26 – 0.96 (m, 5H).

^{13}C NMR (101 MHz, $CDCl_3$) δ 167.50 (d, $^2J_{C–F}$ = 18.2 Hz), 141.87, 134.50, 134.18, 127.86, 120.65, 119.52 (d, $^3J_{C–F}$ = 2.8 Hz), 91.72 (d, $^1J_{C–F}$ = 189.0 Hz), 40.67, 35.60 (d, $^2J_{C–F}$ = 20.2 Hz), 32.88, 26.13, 25.95, 16.53.

^{19}F{H} NMR (376 MHz, $CDCl_3$) δ -188.18.

^{19}F NMR (376 MHz, $CDCl_3$) δ -188.18 (dddd, J = 49.5, 29.3, 27.2, 6.6 Hz).

HRMS (ESI/QTOF) m/z: $[M + Na]^+$ Calcd for $C_{18}H_{24}FNNaOS^+$ 344.1460; Found 344.1452.

$[\alpha]_D^{23}$ = +34.8 (c = 1.00 in $CHCl_3$).

FTIR (neat): $\tilde{v}$ = 3331, 1666, 1588, 1528, 1497, 1400, 1068, 815 cm^{-1}.

HPLC: The enantiomeric ratio (96.5:3.5) was determined *via* HPLC analysis using a CHIRALCEL® IA column, with hexane:isopropanol = 95:5 at a flow rate 1.0 mL/min detected at 254 nm wavelength. Retention time: t_{major} = 12.0 min and t_{minor} = 17.3 min.

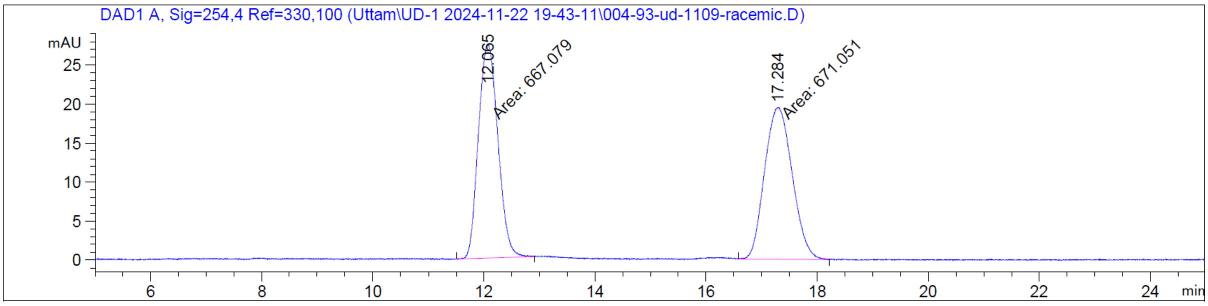

Peak #	RetTime [min]	Type	Width [min]	Area [mAU*s]	Height [mAU]	Area %
1	12.065	MM	0.4061	667.07892	27.37594	49.8516
2	17.284	MM	0.5753	671.05090	19.43968	50.1484

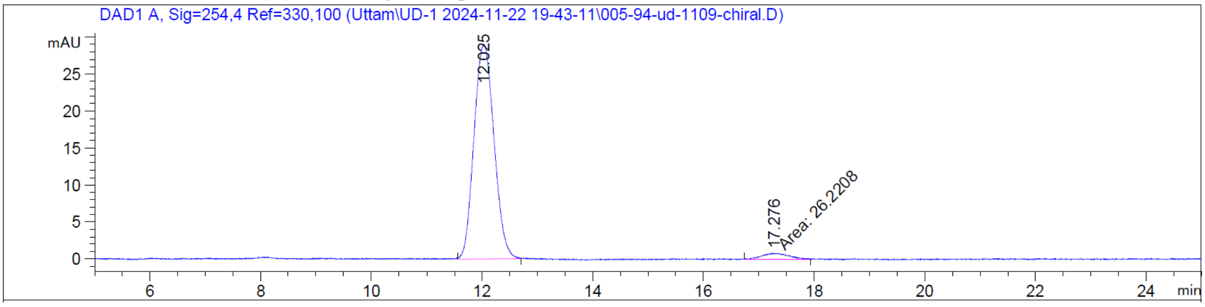

Peak #	RetTime [min]	Type	Width [min]	Area [mAU*s]	Height [mAU]	Area %
1	12.025	VV	0.2948	730.14032	29.04356	96.5333
2	17.276	MM	0.5322	26.22075	8.21143e-1	3.4667

(*R*,*E*)-*N*-(4-Cyanophenyl)-5-cyclohexyl-2-fluoropent-4-enamide (3ad)

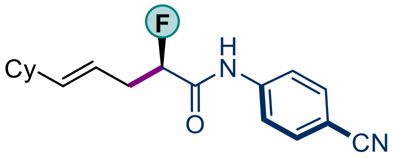

Prepared according to **GP1** with 2-bromo-*N*-(4-cyanophenyl)-2-fluoroacetamide (**2d**) (25.7 mg, 0.10 mmol, 1.0 equiv.) and propa-1,2-dienylcyclohexane (**1a**) (24.4 mg, 0.20 mmol, 2.0 equiv.). Automated flash column chromatography (10 g SiO_2, gradient elution: hexane to 20% EtOAc in hexane) afforded the desired product (+) **3ad** as a white solid (22.2 mg, 74%).

^{1}H NMR (400 MHz, $CDCl_3$) δ 8.10 (d, J = 7.1 Hz, 1H), 7.74 – 7.68 (m, 2H), 7.67 – 7.60 (m, 2H), 5.59 (dd, J = 15.5, 6.7 Hz, 1H), 5.38 (dtd, J = 15.4, 7.0, 1.3 Hz, 1H), 5.03 (ddd, J = 49.6, 6.6, 4.0 Hz, 1H), 2.82 – 2.52 (m, 2H), 1.92 (tdt, J = 10.6, 6.6, 3.3 Hz, 1H), 1.73 – 1.59 (m, 5H), 1.25 – 0.98 (m, 5H).

^{13}C NMR (101 MHz, $CDCl_3$) δ 168.02 (d, $^2J_{C–F}$ = 18.7 Hz), 142.31, 140.57, 133.38, 119.84, 119.06 (d, $^3J_{C–F}$ = 2.9 Hz) 118.60, 108.06, 91.69 (d, $^1J_{C–F}$ = 188.9 Hz), 40.66, 35.50 (d, $^2J_{C–F}$ = 19.8 Hz), 32.85, 32.83, 26.08, 25.90.

^{19}F{H} NMR (376 MHz, $CDCl_3$) δ -188.34.

^{19}F NMR (376 MHz, $CDCl_3$) δ -188.34 (dddd, J = 49.4, 30.0, 26.5, 7.0 Hz).

HRMS (ESI/QTOF) m/z: $[M + H]^+$ Calcd for $C_{18}H_{22}FN_2O^+$ 301.1716; Found 301.1712.

$[\alpha]_D^{23}$ = +13.8 (c = 0.33 in $CHCl_3$).

FTIR (neat): $\tilde{\nu}$ = 3329, 1702, 1690, 1537, 1410, 1251, 1177, 871, 841 cm^{-1}.

HPLC: The enantiomeric ratio (96.5:3.5) was determined *via* HPLC analysis using a CHIRALCEL® IA column, with hexane:isopropanol = 98:2 at a flow rate 1.0 mL/min detected at 254 nm wavelength. Retention time: t_{major} = 29.8 min and t_{minor} = 36.7 min.

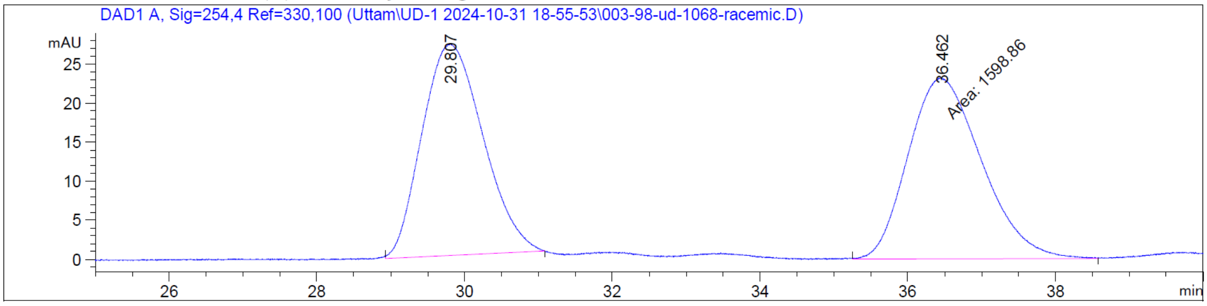

Peak #	RetTime [min]	Type	Width [min]	Area [mAU*s]	Height [mAU]	Area %
1	29.807	VB	0.6710	1551.77441	27.01924	49.2527
2	36.462	MM	1.1519	1598.86414	23.13462	50.7473

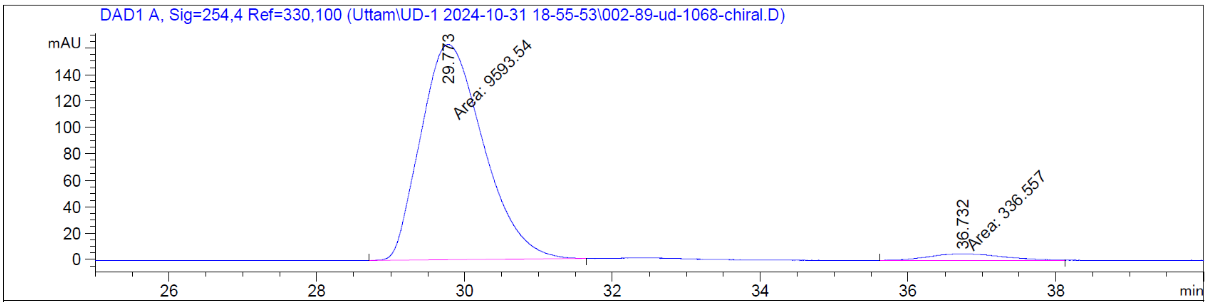

Peak #	RetTime [min]	Type	Width [min]	Area [mAU*s]	Height [mAU]	Area %
1	29.773	MM	0.9808	9593.54004	163.01682	96.6107
2	36.732	MM	1.1200	336.55750	5.00820	3.3893

(*R*,*E*)-5-Cyclohexyl-2-fluoro-N-(4-nitrophenyl)pent-4-enamide (3ae)

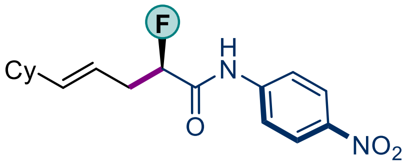

Prepared according to **GP1** with 2-bromo-2-fluoro-*N*-(4-nitrophenyl)acetamide (**2e**) (27.7 mg, 0.10 mmol, 1.0 equiv.) and propa-1,2-dienylcyclohexane (**1a**) (24.4 mg, 0.20 mmol, 2.0 equiv.). Automated flash column chromatography (10 g SiO_2, gradient elution: hexane to 20% EtOAc in hexane) afforded the desired product (+) **3ae** as a white solid (18.5 mg, 57%).

^{1}H NMR (400 MHz, $CDCl_3$) δ 8.27 – 8.22 (m, 2H), 8.20 (d, J = 6.9 Hz, 1H), 7.79 – 7.72 (m, 2H), 5.59 (dd, J = 15.5, 6.7 Hz, 1H), 5.38 (dtd, J = 15.3, 7.0, 1.3 Hz, 1H), 5.05 (ddd, J = 49.6, 6.6, 4.0 Hz, 1H), 2.87 – 2.51 (m, 2H), 1.93 (tdt, J = 10.7, 6.6, 3.3 Hz, 1H), 1.72 – 1.58 (m, 5H), 1.27 – 0.97 (m, 5H).

^{13}C NMR (101 MHz, $CDCl_3$) δ 168.08 (d, $^{2}J_{C\text{–}F}$ = 18.9 Hz), 144.14, 142.40, 142.29, 125.15, 119.46, 118.99 (d, $^{3}J_{C\text{–}F}$ = 2.9 Hz), 91.72 (d, $^{1}J_{C\text{–}F}$ = 189.3 Hz), 40.67, 35.49 (d, $^{2}J_{C\text{–}F}$ = 19.6 Hz), 32.86, 32.84, 26.08, 25.90.

$^{19}F\{H\}$ NMR (377 MHz, $CDCl_3$) δ -188.38.

^{19}F NMR (377 MHz, $CDCl_3$) δ -188.38 (dddd, J = 49.7, 30.3, 26.5, 7.3 Hz).

HRMS (ESI/QTOF) m/z: $[M + H_{-1}]^+$ Calcd for $C_{17}H_{20}FN_2O_3^-$ 319.1463; Found 319.1463.

$[\alpha]_D^{23}$ = +45.7 (c = 0.75 in $CHCl_3$).

FTIR (neat): $\tilde{v}$ = 3336, 1674, 1595, 1336, 1030, 961, 753, 687 cm^{-1}.

HPLC: The enantiomeric ratio (90:10) was determined *via* HPLC analysis using a CHIRALCEL® IA column, with hexane:isopropanol = 99:1 at a flow rate 1.0 mL/min detected at 310 nm wavelength. Retention time: t_{major} = 23.5 min and t_{minor} = 29.2 min.

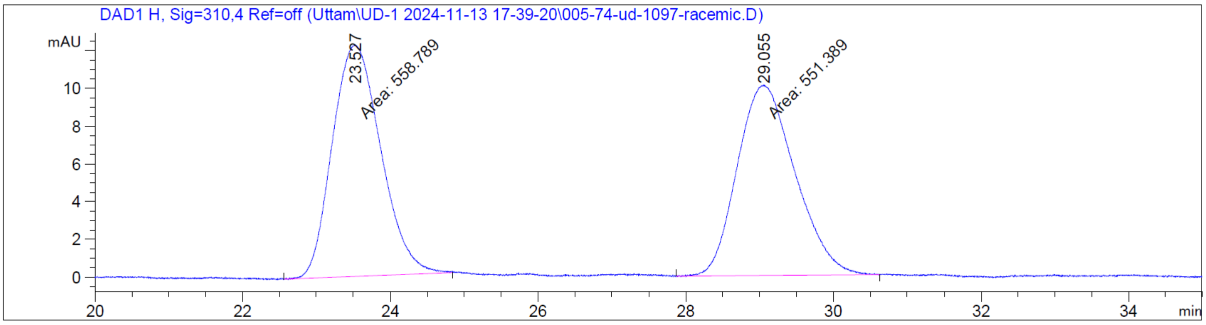

Peak #	RetTime [min]	Type	Width [min]	Area [mAU*s]	Height [mAU]	Area %
1	23.527	MM	0.7593	558.78918	12.26522	50.3333
2	29.055	MM	0.9080	551.38855	10.12066	49.6667

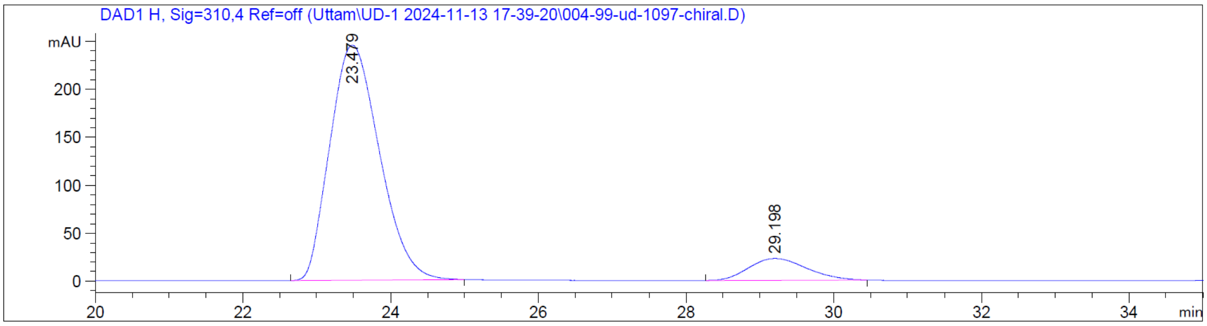

Peak #	RetTime [min]	Type	Width [min]	Area [mAU*s]	Height [mAU]	Area %
1	23.479	BB	0.5493	1.14119e4	245.05959	90.2114
2	29.198	BB	0.6363	1238.28174	22.75752	9.7886

(*R*,*E*)-*N*-(4-Bromophenyl)-5-cyclohexyl-2-fluoropent-4-enamide (3af)

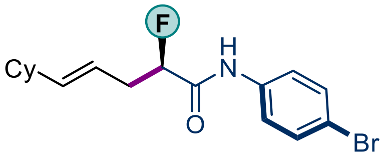

Prepared according to **GP1** with 2-bromo-*N*-(4-bromophenyl)-2-fluoroacetamide (**2f**) (31.1 mg, 0.10 mmol, 1.0 equiv.) and propa-1,2-dienylcyclohexane (**1a**) (24.4 mg, 0.20 mmol, 2.0 equiv.). Automated flash column chromatography (10 g SiO_2, gradient elution: hexane to 20% EtOAc in hexane) afforded the desired product (+) **3af** as a white solid (28 mg, 79%).

^{1}H NMR (400 MHz, $CDCl_3$) δ 7.92 (d, J = 6.8 Hz, 1H), 7.46 (bs, 4H), 5.58 (dd, J = 15.5, 6.7 Hz, 1H), 5.39 (dtd, J = 15.4, 7.0, 1.3 Hz, 1H), 5.00 (ddd, J = 49.7, 6.7, 3.9 Hz, 1H), 2.86 – 2.47 (m, 2H), 1.93 (dtd, J = 10.8, 7.2, 3.3 Hz, 1H), 1.77 – 1.60 (m, 5H), 1.28 – 0.94 (m, 5H).

^{13}C NMR (101 MHz, $CDCl_3$) δ 167.62 (d, $^2J_{C–F}$ = 18.6 Hz), 142.01, 135.71, 132.10, 121.55, 119.36 (d, $^3J_{C–F}$ = 2.8 Hz), 117.62, 91.72 (d, $^1J_{C–F}$ = 189.0 Hz), 40.67, 35.55 (d, $^2J_{C–F}$ = 20.2 Hz), 32.87, 32.85, 26.11, 25.93.

^{19}F{H} NMR (376 MHz, $CDCl_3$) δ -188.29.

^{19}F NMR (376 MHz, CDCl3) δ -188.28 (dddd, J = 49.5, 29.8, 27.1, 6.8 Hz).

HRMS (ESI/QTOF) m/z: $[M + H]^+$ Calcd for $C_{17}H_{22}BrFNO^+$ 354.0869; Found 354.0866.

$[\alpha]_D^{23}$ = +48.0 (c = 1.00 in $CHCl_3$).

FTIR (neat): $\tilde{\nu}$ = 3311, 1670, 1593, 1520, 1396, 1073, 1012, 963 cm^{-1}.

HPLC: The enantiomeric ratio (96.5:3.5) was determined *via* HPLC analysis using a CHIRALCEL® IA column, with hexane:isopropanol = 95:5 at a flow rate 1.0 mL/min detected at 254 nm wavelength. Retention time: t_{major} = 8.9 min and t_{minor} = 12.0 min.

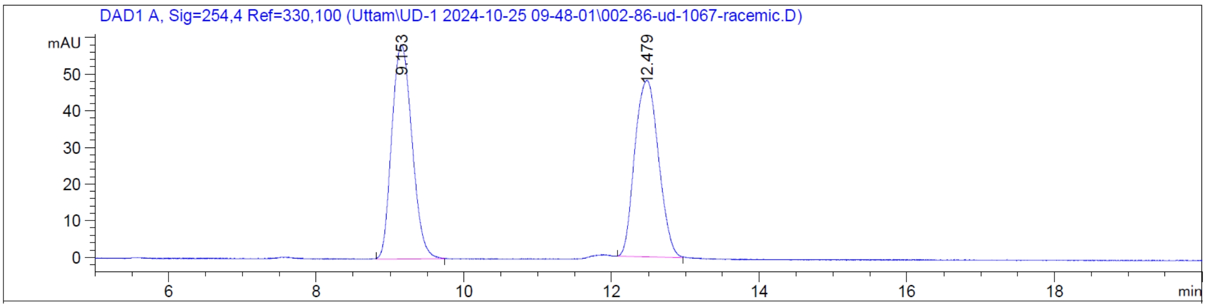

Peak #	RetTime [min]	Type	Width [min]	Area [mAU*s]	Height [mAU]	Area %
1	9.153	VB	0.2245	1114.08984	58.40854	50.8186
2	12.479	BV	0.2625	1078.19897	48.20185	49.1814

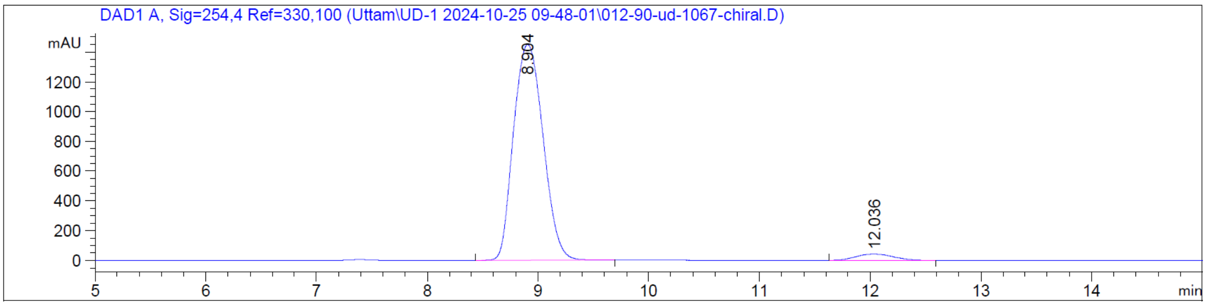

Peak #	RetTime [min]	Type	Width [min]	Area [mAU*s]	Height [mAU]	Area %
1	8.904	BV	0.2214	2.68546e4	1447.84143	96.4829
2	12.036	BV	0.2710	978.93964	42.38588	3.5171

(*R*,*E*)-5-Cyclohexyl-2-fluoro-*N*-(4-(4,4,5,5-tetramethyl-1,3,2-dioxaborolan-2-yl)phenyl)pent-4-enamide (3ag)

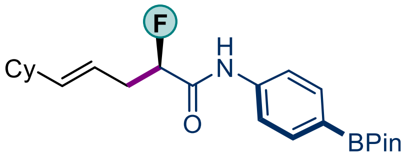

Prepared according to **GP1** with 2-bromo-2-fluoro-*N*-(4-(4,4,5,5-tetramethyl-1,3,2-dioxaborolan-2-yl)phenyl)acetamide (**2g**) (35.8 mg, 0.10 mmol, 1.0 equiv.) and propa-1,2-dienylcyclohexane (**1a**) (24.4 mg, 0.20 mmol, 2.0 equiv.). Automated flash column chromatography (10 g SiO_2, gradient elution: hexane to 20% EtOAc in hexane) afforded the desired product (+) **3ag** as a white solid (30 mg, 75%).

^{1}H NMR (400 MHz, $CDCl_3$) δ 7.96 (d, *J* = 6.8 Hz, 1H), 7.84 – 7.73 (m, 2H), 7.63 – 7.48 (m, 2H), 5.58 (dd, *J* = 15.5, 6.7 Hz, 1H), 5.39 (dtd, *J* = 15.3, 7.0, 1.3 Hz, 1H), 5.00 (ddd, *J* = 49.7, 6.7, 3.9 Hz, 1H), 2.85 – 2.52 (m, 2H), 2.00 – 1.82 (m, 1H), 1.72 – 1.63 (m, 4H), 1.34 (s, 12H), 1.30 – 0.99 (m, 6H).

^{13}C NMR (101 MHz, $CDCl_3$) δ 167.59 (d, $^{2}J_{C–F}$ = 17.8 Hz), 141.91, 139.23, 135.87, 119.48 (d, $^{3}J_{C–F}$ = 2.4 Hz), 118.83, 91.72 (d, $^{1}J_{C–F}$ = 189.0 Hz), 83.82, 40.67, 35.58 (d, $^{2}J_{C–F}$ = 20.2 Hz), 32.86, 32.85, 26.12, 25.95, 24.88, 24.87.

^{19}F{H} NMR (376 MHz, $CDCl_3$) δ -188.02.

^{19}F NMR (376 MHz, $CDCl_3$) δ -188.02 (dddd, *J* = 49.8, 29.7, 27.1, 6.8 Hz).

HRMS (ESI/QTOF) m/z: $[M + Na]^+$ Calcd for $C_{23}H_{33}BFNNaO_3^+$ 424.2439; Found 424.2444.

$[\alpha]_D^{23}$ = +60.4 (c = 0.50 in $CHCl_3$).

FTIR (neat): $\tilde{\nu}$ = 2924, 1684, 1607, 1590, 1528, 1466, 1234, 759 cm^{-1}.

HPLC: The enantiomeric ratio (96.5:3.5) was determined *via* HPLC analysis using a CHIRALCEL® IA column, with hexane:isopropanol = 95:5 at a flow rate 1.0 mL/min detected at 254 nm wavelength. Retention time: t_{major} = 10.1 min and t_{minor} = 6.8 min.

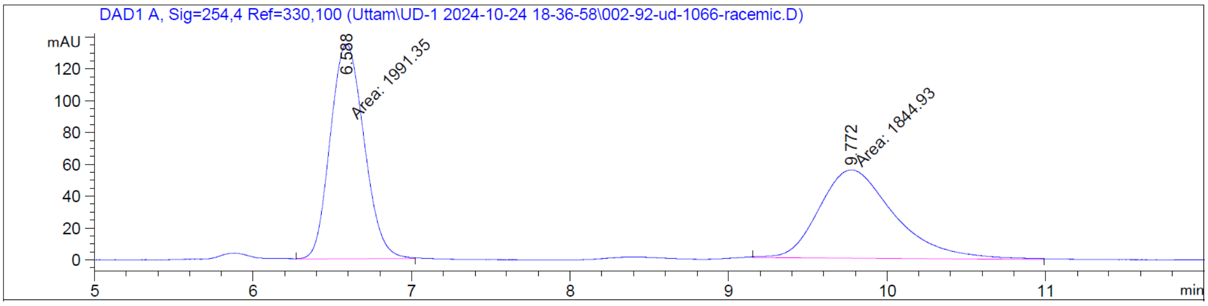

Peak #	RetTime [min]	Type	Width [min]	Area [mAU*s]	Height [mAU]	Area %
1	6.588	MM	0.2459	1991.35059	134.98131	51.9083
2	9.772	MM	0.5526	1844.93127	55.64563	48.0917

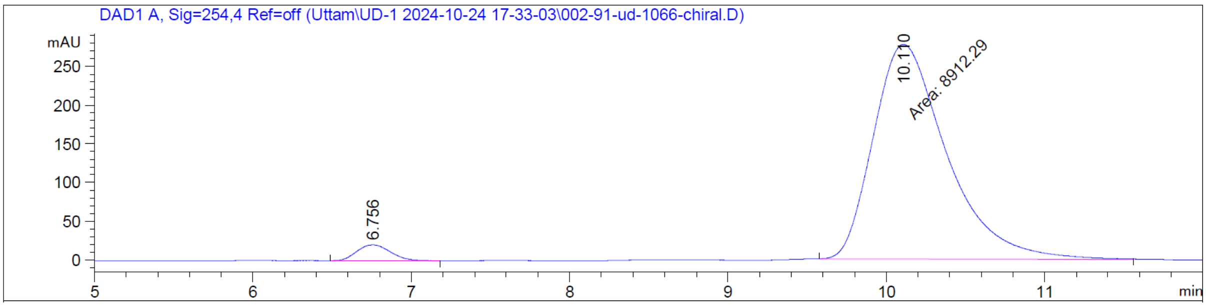

Peak #	RetTime [min]	Type	Width [min]	Area [mAU*s]	Height [mAU]	Area %
1	6.756	BB	0.1753	302.80066	20.56656	3.2859
2	10.110	MM	0.5363	8912.28711	276.99130	96.7141

(*R*,*E*)-5-Cyclohexyl-2-fluoro-*N*-(1-methyl-1*H*-indol-6-yl)pent-4-enamide (3ah)

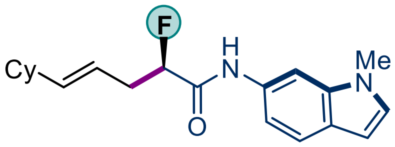

Prepared according to **GP1** with 2-bromo-2-fluoro-*N*-(1-methyl-1*H*-indol-6-yl)acetamide (**2h**) (28.5 mg, 0.10 mmol, 1.0 equiv.) and propa-1,2-dienylcyclohexane (**1a**) (24.4 mg, 0.20 mmol, 2.0 equiv.). Automated flash column chromatography (10 g SiO_2, gradient elution: hexane to 20% EtOAc in hexane) afforded the desired product (+) **3ah** as a white solid (26 mg, 80%). ^{1}H NMR (400 MHz, $CDCl_3$) δ 7.96 (d, *J* = 6.5 Hz, 1H), 7.87 (t, *J* = 1.3 Hz, 1H), 7.30 – 7.22 (m, 2H), 7.06 (d, *J* = 3.1 Hz, 1H), 6.46 (d, *J* = 3.1 Hz, 1H), 5.61 (dd, *J* = 15.5, 6.6 Hz, 1H),

5.44 (dtd, J = 15.3, 6.9, 1.2 Hz, 1H), 5.04 (ddd, J = 49.8, 6.8, 3.8 Hz, 1H), 3.79 (s, 3H), 2.86 – 2.55 (m, 2H), 2.03 – 1.87 (m, 1H), 1.77 – 1.59 (m, 5H), 1.28 – 0.97 (m, 5H).

^{13}C NMR (101 MHz, $CDCl_3$) δ 167.39 (d, $^2J_{C–F}$ = 18.2 Hz), 141.61, 134.49, 129.84, 128.88, 128.53, 119.86 (d, $^3J_{C–F}$ = 2.9 Hz), 115.69, 112.87, 109.40, 101.14, 91.86 (d, $^1J_{C–F}$ = 188.6 Hz), 40.70, 35.75 (d, $^2J_{C–F}$ = 20.0 Hz), 32.97, 32.90, 26.16, 25.99.

^{19}F{H} NMR (377 MHz, $CDCl_3$) δ -188.10.

^{19}F NMR (377 MHz, $CDCl_3$) δ -188.10 (dtd, J = 49.6, 28.4, 6.4 Hz).

HRMS (ESI/QTOF) m/z: $[M + H]^+$ Calcd for $C_{20}H_{26}FN_2O^+$ 329.2029; Found 329.2025.

$[\alpha]_D^{23}$ = +30.2 (c = 0.50 in $CHCl_3$).

FTIR (neat): $\tilde{\nu}$ = 3318, 1671, 1545, 1494, 1260, 971, 801, 718 cm^{-1}.

HPLC: The enantiomeric ratio (97:3) was determined *via* HPLC analysis using a CHIRALCEL® IA column, with hexane:isopropanol = 90:10 at a flow rate 1.0 mL/min detected at 254 nm wavelength. Retention time: t_{major} = 17.3 min and t_{minor} = 23.3 min.

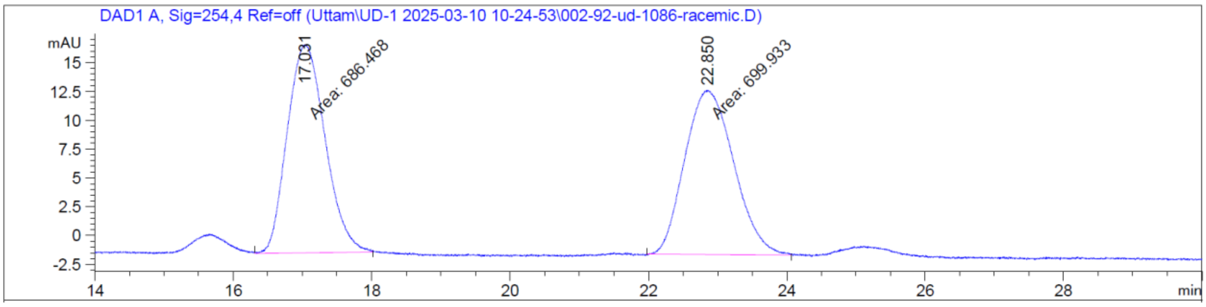

Peak #	RetTime [min]	Type	Width [min]	Area [mAU*s]	Height [mAU]	Area %
1	17.031	MM	0.6342	686.46820	18.03978	49.5144
2	22.850	MM	0.8195	699.93311	14.23535	50.4856

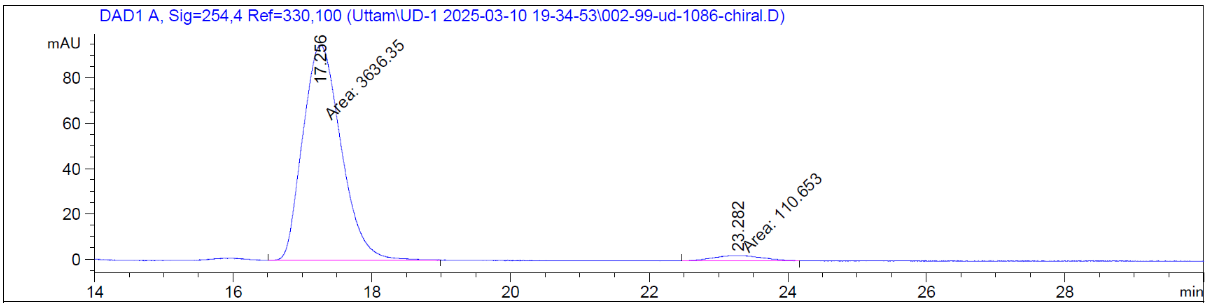

Peak #	RetTime [min]	Type	Width [min]	Area [mAU*s]	Height [mAU]	Area %
1	17.256	MM	0.6372	3636.34937	95.11331	97.0469
2	23.282	MM	0.7900	110.65292	2.33437	2.9531

(*R*,*E*)-*N*-Benzyl-5-cyclohexyl-2-fluoropent-4-enamide (3ai)

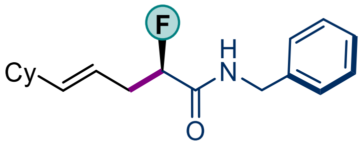

Prepared according to **GP1** with *N*-benzyl-2-bromo-2-fluoroacetamide (**2i**) (24.6 mg, 0.10 mmol, 1.0 equiv.) and propa-1,2-dienylcyclohexane (**1a**) (24.4 mg, 0.20 mmol, 2.0 equiv.). Automated flash column chromatography (10 g SiO_2, gradient elution: hexane to 20% EtOAc in hexane) afforded the desired product (+) **3ai** as a white solid (15 mg, 52%).

^{1}H NMR (400 MHz, $CDCl_3$) δ 7.38 – 7.27 (m, 5H), 6.57 (s, 1H), 5.56 (dd, *J* = 15.5, 6.5 Hz, 1H), 5.35 (dtd, *J* = 15.4, 7.0, 1.3 Hz, 1H), 4.95 (ddd, *J* = 49.5, 6.4, 3.9 Hz, 1H), 4.55 (dd, *J* = 14.7, 6.2 Hz, 1H), 4.42 (dd, *J* = 14.7, 5.5 Hz, 1H), 2.63 (dddt, *J* = 38.1, 30.3, 15.1, 6.7 Hz, 2H), 1.91 (s, 1H), 1.68 (tt, *J* = 13.3, 3.3 Hz, 5H), 1.25 – 1.10 (m, 3H), 1.03 (qd, *J* = 12.6, 3.7 Hz, 2H).

^{13}C NMR (101 MHz, $CDCl_3$) δ 169.42 (d, $^2J_{C–F}$ = 19.4 Hz), 141.54, 137.62, 128.77, 127.81, 127.71, 119.66 (d, $^3J_{C–F}$ = 2.4 Hz), 91.66 (d, $^1J_{C–F}$ = 187.4 Hz), 42.96, 40.64, 35.58 (d, $^2J_{C–F}$ = 19.8 Hz), 32.89, 32.81, 26.16, 26.02.

^{19}F{H} NMR (376 MHz, $CDCl_3$) δ -190.47.

^{19}F NMR (376 MHz, $CDCl_3$) δ -190.47 (dddd, *J* = 49.6, 30.6, 26.3, 4.7 Hz).

HRMS (ESI/QTOF) m/z: $[M + Na]^+$ Calcd for $C_{18}H_{24}FNNaO^+$ 312.1740; Found 312.1735.

$[\alpha]_D^{23}$ = +47.3 (c = 0.75 in $CHCl_3$).

FTIR (neat): $\tilde{\nu}$ = 2973, 1660, 1535, 1449, 1260, 1117, 1077, 968 cm^{-1}.

HPLC: The enantiomeric ratio (89:11) was determined *via* HPLC analysis using a CHIRALCEL® IA column, with hexane:isopropanol = 95:5 at a flow rate 1.0 mL/min detected at 214 nm wavelength. Retention time: t_{major} = 8.5 min and t_{minor} = 9.5 min.

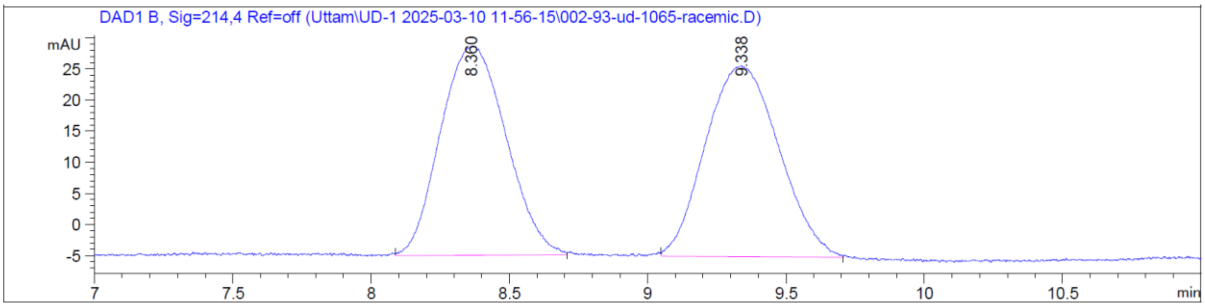

Peak #	RetTime [min]	Type	Width [min]	Area [mAU*s]	Height [mAU]	Area %
1	8.360	VV	0.1946	555.36719	33.55798	49.4443
2	9.338	VV	0.2179	567.85065	30.69028	50.5557

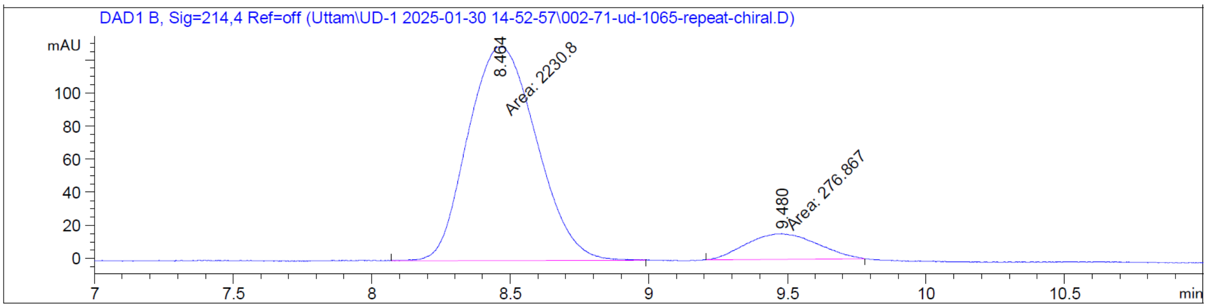

Peak #	RetTime [min]	Type	Width [min]	Area [mAU*s]	Height [mAU]	Area %
1	8.464	MM	0.2876	2230.80493	129.26450	88.9592
2	9.480	MM	0.2979	276.86722	15.48933	11.0408

(*R*,*E*)-*N*,5-Dicyclohexyl-2-fluoropent-4-enamide (3aj)

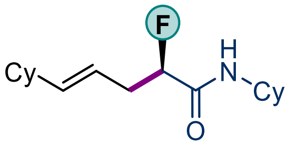

A modified **GP1** was followed with 2-bromo-*N*-cyclohexyl-2-fluoroacetamide (**1j**) (23.8 mg, 0.10 mmol, 1 equiv.) and propa-1,2-dienylcyclohexane (**1a**) (24.4 mg, 0.20 mmol, 2.0 equiv.) at -35 °C. Automated flash column chromatography (10 g SiO_2, gradient elution: hexane to 20% EtOAc in hexane) afforded the desired product (+) **3aj** as a colourless oil (14 mg, 50%).

^{1}H NMR (400 MHz, $CDCl_3$) δ 6.08 (d, J = 7.5 Hz, 1H), 5.47 (dd, J = 15.5, 6.6 Hz, 1H), 5.27 (dtd, J = 15.3, 7.0, 1.3 Hz, 1H), 4.78 (ddd, J = 49.8, 6.6, 3.9 Hz, 1H), 3.73 (dtdt, J = 13.7, 9.4, 4.6, 2.3 Hz, 1H), 2.72 – 2.31 (m, 2H), 1.84 (tdd, J = 10.8, 5.4, 2.8 Hz, 3H), 1.73 – 1.48 (m, 8H), 1.41 – 1.25 (m, 2H), 1.24 – 0.89 (m, 8H).

^{13}C NMR (101 MHz, CDCl3) δ 168.49 (d, $^{2}J_{C–F}$ = 19.0 Hz), 141.26, 119.84 (d, $^{3}J_{C–F}$ = 2.8 Hz), 91.55 (d, $^{1}J_{C–F}$ = 187.0 Hz), 47.77, 40.64, 35.62 (d, $^{2}J_{C–F}$ = 19.8 Hz), 33.18, 32.92, 32.88, 26.18, 26.01, 25.45, 24.77.

^{19}F{H} NMR (376 MHz, $CDCl_3$) δ -189.84.

^{19}F NMR (376 MHz, $CDCl_3$) δ -189.60 – -190.12 (m).

HRMS (ESI/QTOF) m/z: $[M + H]^+$ Calcd for $C_{17}H_{29}FNO^+$ 282.2233; Found 282.2229.

$[\alpha]_D^{23}$ = +25.8 (c = 0.50 in $CHCl_3$).

FTIR (neat): $\tilde{\nu}$ = 3302, 1645, 1544, 1443, 1107, 1056, 968, 724 cm^{-1}.

HPLC: The enantiomeric ratio (91:9) was determined *via* HPLC analysis using a CHIRALCEL® IA column, with hexane:isopropanol = 95:5 at a flow rate 1.0 mL/min detected at 210 nm wavelength. Retention time: t_{major} = 5.7 min and t_{minor} = 6.8 min.

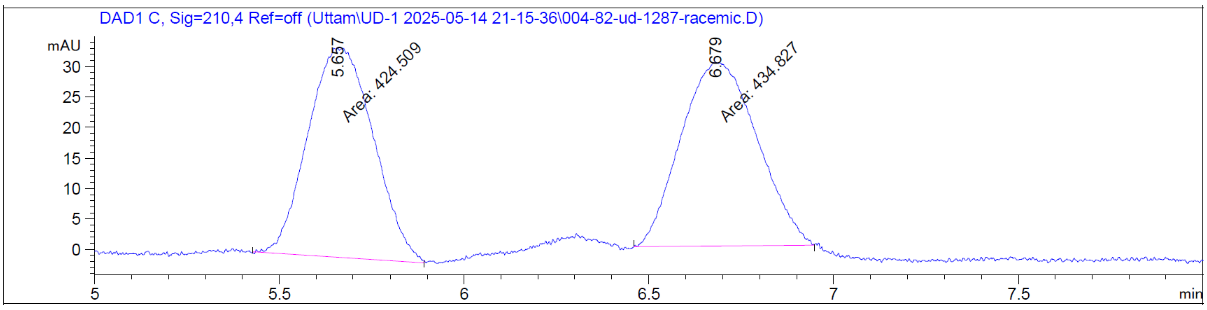

Peak #	RetTime [min]	Type	Width [min]	Area [mAU*s]	Height [mAU]	Area %
1	5.657	MM	0.2053	424.50922	34.46410	49.3997
2	6.679	MM	0.2398	434.82690	30.21704	50.6003

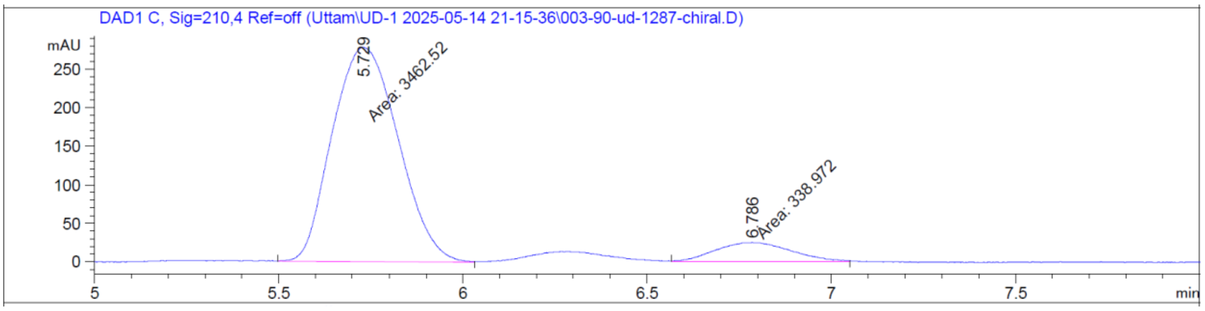

Peak #	RetTime [min]	Type	Width [min]	Area [mAU*s]	Height [mAU]	Area %
1	5.729	MM	0.2072	3462.51904	278.48169	91.0832
2	6.786	MM	0.2344	338.97238	24.10666	8.9168

(*R*)-2-Fluoro-*N*-(4-methoxyphenyl)-6-phenylhexanamide (5aa)

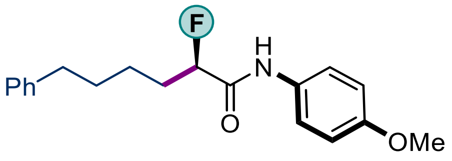

Prepared according to **GP2** with 2-bromo-2-fluoro-*N*-(4-methoxyphenyl)acetamide (**2a**) (26.2 mg, 0.10 mmol, 1.0 equiv.) and but-3-enylbenzene (**4a**) (26.4 mg, 0.20 mmol, 2.0 equiv.). Automated flash column chromatography (10 g SiO_2, gradient elution: hexane to 20% EtOAc in hexane) afforded the desired product (+) **5aa** as a white solid (18 mg, 57%).

^{1}H NMR (400 MHz, $CDCl_3$) δ 7.89 (d, J = 6.7 Hz, 1H), 7.53 – 7.40 (m, 2H), 7.32 – 7.25 (m, 2H), 7.23 – 7.14 (m, 3H), 6.94 – 6.84 (m, 2H), 5.00 (ddd, J = 49.9, 7.6, 3.6 Hz, 1H), 3.80 (s, 3H), 2.64 (t, J = 7.9 Hz, 2H), 2.21 – 1.87 (m, 2H), 1.75 – 1.65 (m, 2H), 1.58 (ddd, J = 9.9, 7.2, 4.9 Hz, 2H).

^{13}C NMR (101 MHz, $CDCl_3$) δ 167.85 (d, $^{2}J_{C–F}$ = 17.9 Hz), 156.87, 142.20, 129.75, 128.39, 128.34, 125.79, 121.81, 114.26, 92.17 (d, $^{1}J_{C–F}$ = 187.3 Hz), 55.50, 35.66, 32.28 (d, $^{2}J_{C–F}$ = 20.2 Hz), 31.00, 24.13 (d, $^{3}J_{C–F}$ = 2.7 Hz).

^{19}F{H} NMR (376 MHz, $CDCl_3$) δ -188.09.

^{19}F NMR (376 MHz, $CDCl_3$) δ -185.55 – -190.88 (m).

HRMS (nanochip-ESI/LTQ-Orbitrap) m/z: $[M + Na]^+$ Calcd for $C_{19}H_{22}FNNaO_2^+$ 338.1532; Found 338.1523.

$[\alpha]_D^{23}$ = +40.6 (c = 1.00 in $CHCl_3$).

FTIR (neat): $\tilde{\nu}$ = 2924, 1663, 1533, 1514, 1237, 1030, 699 cm^{-1}.

HPLC: The enantiomeric ratio (96.5:3.5) was determined *via* HPLC analysis using a CHIRALCEL® IA column, with hexane:isopropanol = 95:5 at a flow rate 1.0 mL/min detected at 254 nm wavelength. Retention time: t_{major} = 19.4 min and t_{minor} = 27.4 min.

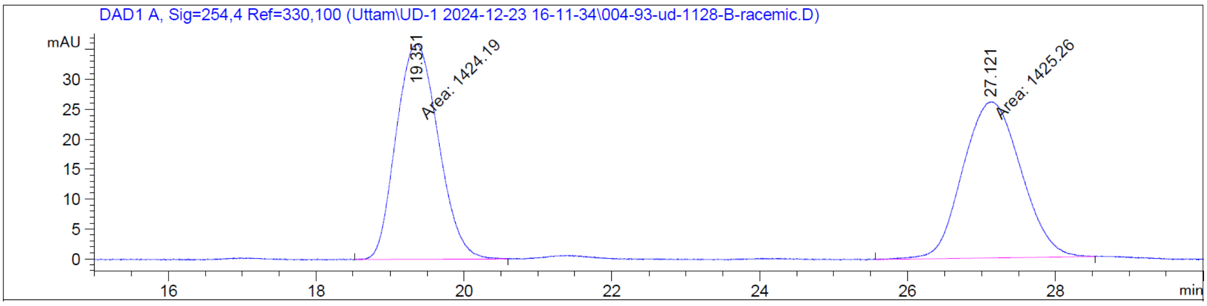

Peak #	RetTime [min]	Type	Width [min]	Area [mAU*s]	Height [mAU]	Area %
1	19.351	MM	0.6603	1424.19482	35.94685	49.9814
2	27.121	MM	0.9095	1425.25513	26.11838	50.0186

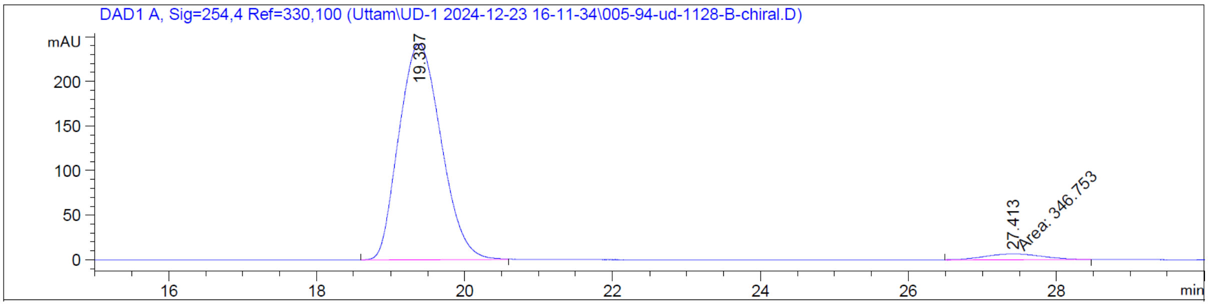

Peak #	RetTime [min]	Type	Width [min]	Area [mAU*s]	Height [mAU]	Area %
1	19.387	BV	0.4627	9552.47168	241.88631	96.4972
2	27.413	MM	0.8909	346.75281	6.48704	3.5028

Methyl (*R*)-6-fluoro-7-((4-methoxyphenyl)amino)-7-oxoheptanoate (5ba)

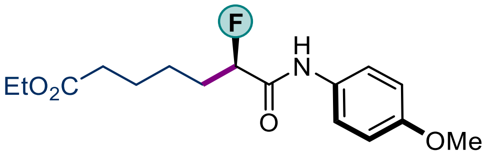

Prepared according to **GP2** with 2-bromo-2-fluoro-*N*-(4-methoxyphenyl)acetamide (**2a**) (26.2 mg, 0.10 mmol, 1.0 equiv.) and ethyl pent-4-enoate (**4b**) (25.6 mg, 0.20 mmol, 2.0 equiv.). Automated flash column chromatography (10 g SiO_2, gradient elution: hexane to 20% EtOAc in hexane) afforded the desired product (+) **5ba** as a colourless oil (16 mg, 51%).

^{1}H NMR (400 MHz, $CDCl_3$) δ 7.88 (d, J = 6.6 Hz, 1H), 7.53 – 7.36 (m, 2H), 6.95 – 6.82 (m, 2H), 5.00 (ddd, J = 49.9, 7.6, 3.7 Hz, 1H), 4.12 (q, J = 7.1 Hz, 2H), 3.80 (s, 3H), 2.33 (t, J = 7.4 Hz, 2H), 2.16 – 1.89 (m, 2H), 1.70 (pd, J = 7.1, 3.2 Hz, 2H), 1.58 – 1.53 (m, 2H), 1.25 (t, J = 7.1 Hz, 3H).

^{13}C NMR (101 MHz, $CDCl_3$) δ 173.38, 167.67 (d, $^2J_{C–F}$ = 17.8 Hz), 156.87, 129.72, 121.77, 114.27, 91.97 (d, $^1J_{C–F}$ = 187.5 Hz), 60.32, 55.50, 34.03, 32.07 (d, $^2J_{C–F}$ = 20.3 Hz), 24.47, 24.02 (d, $^3J_{C–F}$ = 2.9 Hz), 14.24.

^{19}F{H} NMR (377 MHz, $CDCl_3$) δ -188.16.

^{19}F NMR (377 MHz, $CDCl_3$) δ -188.16 (dddd, J = 49.8, 29.8, 25.6, 6.5 Hz).

HRMS (nanochip-ESI/LTQ-Orbitrap) m/z: $[M + Na]^+$ Calcd for $C_{16}H_{22}FNNaO_4^+$ 334.1431; Found 334.1421.

$[\alpha]_D^{23}$ = +24.0 (c = 0.50 in $CHCl_3$).

FTIR (neat): $\tilde{\nu}$ = 3341, 1731, 1661, 1540, 1247, 1190, 1091, 822 cm^{-1}.

HPLC: The enantiomeric ratio (95:5) was determined *via* HPLC analysis using a CHIRALCEL® IA column, with hexane:isopropanol = 90:10 at a flow rate 1.0 mL/min detected at 254 nm wavelength. Retention time: t_{major} = 15.6 min and t_{minor} = 22.9 min.

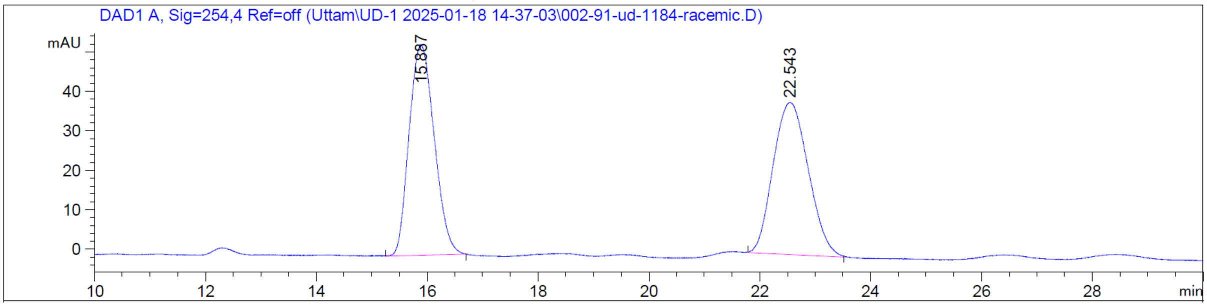

Peak #	RetTime [min]	Type	Width [min]	Area [mAU*s]	Height [mAU]	Area %
1	15.887	BB	0.3691	1680.51233	53.40664	49.6906
2	22.543	VV	0.5166	1701.44238	38.53493	50.3094

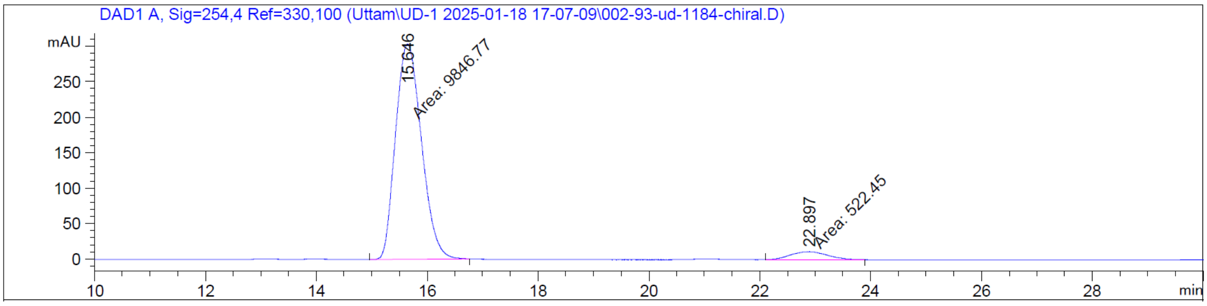

Peak #	RetTime [min]	Type	Width [min]	Area [mAU*s]	Height [mAU]	Area %
1	15.646	MM	0.5405	9846.76563	303.65717	94.9615
2	22.897	MM	0.7635	522.44965	11.40426	5.0385

(*S*)-6-((*tert*-Butyldimethylsilyl)oxy)-2-fluoro-*N*-phenylhexanamide (5cb)

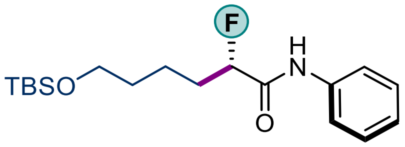

A modified **GP2** was followed with 2-bromo-2-fluoro-*N*-phenylacetamide (**2b**) (23.2 mg, 0.10 mmol, 1 equiv.) and (but-3-en-1-yloxy)(*tert*-butyl)dimethylsilane (**4c**) (37.3 mg, 0.20 mmol, 2 equiv.) with *ent*-**L4** as chiral ligand. Automated flash column chromatography (10 g SiO_2, gradient elution: hexane to 10% EtOAc in hexane) afforded the desired product (-) **5cb** as a colourless oil (18 mg, 53%).

^{1}H NMR (400 MHz, $CDCl_3$) δ 7.93 (d, J = 6.5 Hz, 1H), 7.56 – 7.48 (m, 2H), 7.35 – 7.27 (m, 2H), 7.11 (tt, J = 7.5, 1.0 Hz, 1H), 4.97 (ddd, J = 49.9, 7.7, 3.6 Hz, 1H), 3.59 (q, J = 4.3 Hz, 2H), 2.15 – 1.80 (m, 2H), 1.55 (q, J = 3.2 Hz, 4H), 0.84 (d, J = 0.9 Hz, 9H), -0.00 (d, J = 1.0 Hz, 6H).

^{13}C NMR (101 MHz, $CDCl_3$) δ 168.07 (d, $^2J_{C-F}$ = 18.2 Hz), 136.69, 129.13, 124.94, 120.00, 92.19 (d, $^1J_{C-F}$ = 187.1 Hz), 62.72, 32.29, 32.23 (d, $^2J_{C-F}$ = 20.3 Hz), 25.95, 20.91 (d, $^4J_{C-F}$ = 2.9 Hz), 18.34, -5.30.

^{19}F{H} NMR (377 MHz, $CDCl_3$) δ -188.06.

^{19}F NMR (377 MHz, CDC13) δ -188.06 (dddd, J = 50.0, 30.2, 25.9, 6.9 Hz).

HRMS (ESI/QTOF) m/z: $[M + Na]^+$ Calcd for $C_{18}H_{30}FNNaO_2Si^+$ 362.1928; Found 362.1941.

$[\alpha]_D^{23}$ = -29.3 (c = 0.66 in $CHCl_3$).

HPLC: The enantiomeric ratio (95.5:4.5) was determined *via* HPLC analysis using a CHIRALCEL® IA column, with hexane:isopropanol = 98:2 at a flow rate 1.0 mL/min detected at 254 nm wavelength. Retention time: t_{major} = 9.9 min and t_{minor} = 7.9 min.

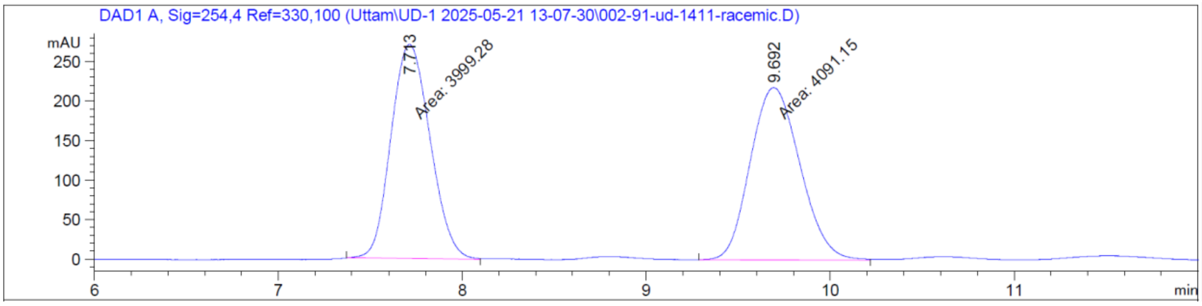

Peak #	RetTime [min]	Type	Width [min]	Area [mAU*s]	Height [mAU]	Area %
1	7.713	MM	0.2461	3999.27881	270.84012	49.4322
2	9.692	MM	0.3126	4091.15308	218.13901	50.5678

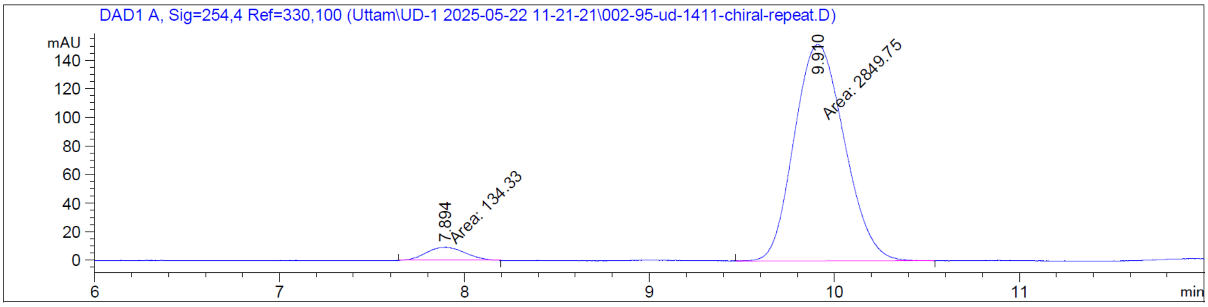

Peak #	RetTime [min]	Type	Width [min]	Area [mAU*s]	Height [mAU]	Area %
1	7.894	MM	0.2404	134.32962	9.31296	4.5015
2	9.910	MM	0.3137	2849.75073	151.40137	95.4985

(*R*)-2-Cyclohexyl-2-fluoro-*N*-(4-methoxyphenyl)acetamide (5da)

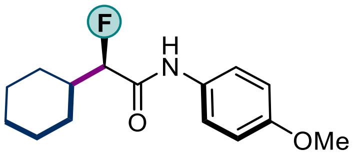

Prepared according to **GP2** with 2-bromo-2-fluoro-*N*-(4-methoxyphenyl)acetamide (**2a**) (26.2 mg, 0.10 mmol, 1.0 equiv.) and cyclohexene (**4d**) (16.4 mg, 0.20 mmol, 2.0 equiv.). Automated flash column chromatography (10 g SiO_2, gradient elution: hexane to 20% EtOAc in hexane) afforded the desired product (+) **5da** as a white solid (12 mg, 45%).

^{1}H NMR (400 MHz, $CDCl_3$) δ 7.85 (bs, 1H), 7.51 – 7.40 (m, 2H), 6.96 – 6.80 (m, 2H), 4.81 (dd, J = 49.8, 2.8 Hz, 1H), 3.80 (s, 3H), 2.17 – 1.93 (m, 1H), 1.86 – 1.74 (m, 3H), 1.71 – 1.60 (m, 2H), 1.40 – 1.12 (m, 5H).

^{13}C NMR (101 MHz, $CDCl_3$) δ 167.42 (d, $^{2}J_{C–F}$ = 18.6 Hz), 156.84, 129.76, 121.80, 114.26, 95.60 (d, $^{1}J_{C–F}$ = 189.4 Hz), 55.51, 40.28 (d, $^{2}J_{C–F}$ = 19.0 Hz), 28.96 (d, $^{3}J_{C–F}$ = 2.4 Hz), 26.15, 25.89, 25.81, 25.64 (d, $^{3}J_{C–F}$ = 4.3 Hz).

^{19}F{H} NMR (376 MHz, $CDCl_3$) δ -197.18.

^{19}F NMR (376 MHz, $CDCl_3$) δ -197.18 (ddd, J = 49.9, 31.7, 6.8 Hz).

HRMS (nanochip-ESI/LTQ-Orbitrap) m/z: [M + Na]$^+$ Calcd for $C_{15}H_{20}FNNaO_2^+$ 288.1376; Found 288.1367.

$[\alpha]_D^{23}$ = +68.0 (c = 0.50 in $CHCl_3$).

FTIR (neat): $\tilde{\nu}$ = 2927, 1660, 1531, 1514, 1248, 1031, 822 cm^{-1}.

HPLC: The enantiomeric ratio (93:7) was determined *via* HPLC analysis using a CHIRALCEL® IA column, with hexane:isopropanol = 95:5 at a flow rate 1.0 mL/min detected at 254 nm wavelength. Retention time: t_{major} = 15.2 min and t_{minor} = 23.3 min.

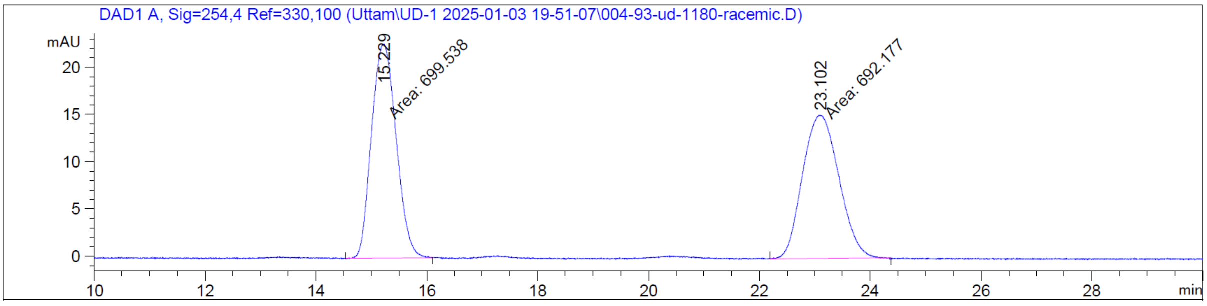

Peak #	RetTime [min]	Type	Width [min]	Area [mAU*s]	Height [mAU]	Area %
1	15.229	MM	0.5144	699.53821	22.66577	50.2644
2	23.102	MM	0.7599	692.17749	15.18084	49.7356

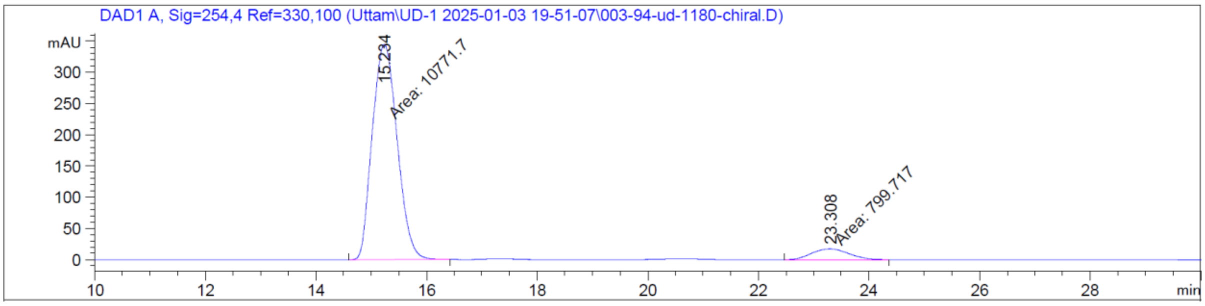

Peak #	RetTime [min]	Type	Width [min]	Area [mAU*s]	Height [mAU]	Area %
1	15.234	MM	0.5208	1.07717e4	344.70215	93.0889
2	23.308	MM	0.7713	799.71674	17.28145	6.9111

(*R*)-2-Fluoro-*N*-(1-methyl-1*H*-indol-5-yl)-6-phenylhexanamide (5ah)

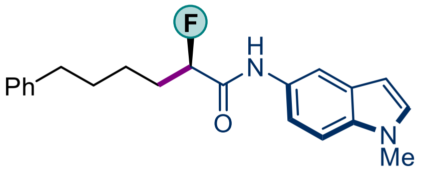

Prepared according to **GP2** with 2-bromo-2-fluoro-*N*-(1-methyl-1*H*-indol-6-yl)acetamide (**2h**) (28.5 mg, 0.10 mmol, 1.0 equiv.) and but-3-enylbenzene (**4a**) (26.4 mg, 0.20 mmol, 2.0 equiv.). Automated flash column chromatography (10 g SiO_2, gradient elution: hexane to 20% EtOAc in hexane) afforded the desired product (+) **5ah** as a white solid (17 mg, 50%).

^{1}H NMR (400 MHz, $CDCl_3$) δ 8.01 (d, *J* = 6.7 Hz, 1H), 7.89 (d, *J* = 1.8 Hz, 1H), 7.32 – 7.25 (m, 4H), 7.18 (dt, *J* = 7.4, 3.1 Hz, 3H), 7.06 (d, *J* = 3.1 Hz, 1H), 6.46 (d, *J* = 3.1 Hz, 1H), 5.04 (ddd, *J* = 50.0, 7.6, 3.6 Hz, 1H), 3.79 (s, 3H), 2.65 (t, *J* = 7.6 Hz, 2H), 2.23 – 1.92 (m, 2H), 1.72 (p, *J* = 7.1 Hz, 2H), 1.65 – 1.55 (m, 2H).

^{13}C NMR (101 MHz, $CDCl_3$) δ 167.86 (d, $^2J_{C–F}$ = 17.8 Hz), 142.27, 134.49, 129.87, 128.93, 128.55, 128.41, 128.33, 125.77, 115.64, 112.83, 109.42, 101.15, 92.31 (d, $^1J_{C–F}$ = 187.1 Hz), 35.69, 32.97, 32.38 (d, $^2J_{C–F}$ = 20.0 Hz), 31.05, 24.19 (d, $^3J_{C–F}$ = 2.5 Hz).

^{19}F{H} NMR (377 MHz, $CDCl_3$) δ -187.89.

^{19}F NMR (377 MHz, $CDCl_3$) δ -187.89 (dddd, *J* = 50.1, 30.3, 26.1, 6.7 Hz).

HRMS (nanochip-ESI/LTQ-Orbitrap) m/z: $[M + Na]^+$ Calcd for $C_{21}H_{23}FN_2NaO^+$ 361.1692; Found 361.1685.

$[\alpha]_D^{23}$ = +27.3 (c = 1.00 in $CHCl_3$).

FTIR (neat): $\tilde{\nu}$ = 3349, 1657, 1581, 1534, 1464, 1085, 796 cm^{-1}.

HPLC: The enantiomeric ratio (95.5:4.5) was determined *via* HPLC analysis using a CHIRALCEL® IA column, with hexane:isopropanol = 80:20 at a flow rate 1.0 mL/min detected at 254 nm wavelength. Retention time: t_{major} = 22.9 min and t_{minor} = 37.0 min.

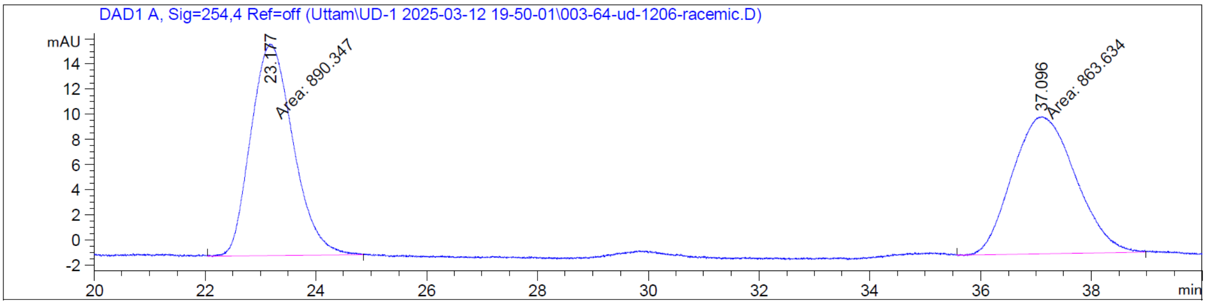

Peak #	RetTime [min]	Type	Width [min]	Area [mAU*s]	Height [mAU]	Area %
1	23.177	MM	0.8824	890.34711	16.81761	50.7615
2	37.096	MM	1.3176	863.63422	10.92402	49.2385

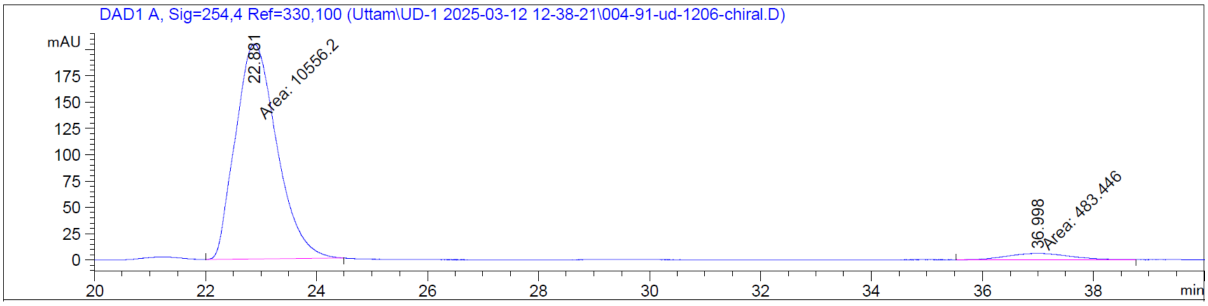

Peak #	RetTime [min]	Type	Width [min]	Area [mAU*s]	Height [mAU]	Area %
1	22.881	MM	0.8616	1.05562e4	204.20766	95.6208
2	36.998	MM	1.3204	483.44604	6.10218	4.3792

(*R*)-2-Fluoro-*N*-(4-methoxyphenyl)octanamide (5ea)

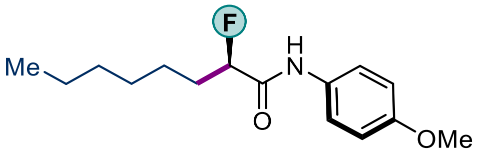

A modified general procedure **GP2** was followed with 2-bromo-2-fluoro-*N*-(4-methoxyphenyl)acetamide (**2a**) (26.2 mg, 0.10 mmol, 1.0 equiv.) and *cis*-2-hexene (**4e**) (16.8 mg, 0.20 mmol, 2.0 equiv.) at RT. Automated flash column chromatography (10 g SiO_2, gradient elution: hexane to 20% EtOAc in hexane) afforded the desired product (+) **5ea** as a white solid (12.5 mg, 47%).

^{1}H NMR (400 MHz, $CDCl_3$) δ 7.88 (d, J = 6.4 Hz, 1H), 7.50 – 7.42 (m, 2H), 6.92 – 6.84 (m, 2H), 4.99 (ddd, J = 50.0, 7.7, 3.6 Hz, 1H), 3.80 (s, 3H), 2.13 – 1.99 (m, 1H), 1.98 – 1.84 (m, 1H), 1.55 – 1.44 (m, 2H), 1.38 – 1.24 (m, 6H), 0.88 (t, J = 7.1 Hz, 3H).
^{13}C NMR (101 MHz, $CDCl_3$) δ 167.97 (d, $^{2}J_{C–F}$ = 17.9 Hz), 156.83, 129.81, 121.76, 114.26, 92.29 (d, $^{1}J_{C–F}$ = 186.5 Hz), 55.50, 32.45 (d, $^{2}J_{C–F}$ = 19.8 Hz), 31.57, 28.83, 24.37 (d, $^{3}J_{C–F}$ = 2.7 Hz), 22.53, 14.04.
^{19}F{H} NMR (376 MHz, $CDCl_3$) δ -188.07.
^{19}F NMR (376 MHz, $CDCl_3$) δ -188.07 (dddd, J = 50.0, 29.8, 26.3, 7.0 Hz).
HRMS (nanochip-ESI/LTQ-Orbitrap) m/z: $[M + Na]^+$ Calcd for $C_{15}H_{22}FNNaO_2^+$ 290.1523; Found 290.1532.
$[\alpha]_D^{23}$ = +14.0 (c = 0.75 in $CHCl_3$).
FTIR (neat): $\tilde{\nu}$ = 3332, 1660, 1599, 1538, 1465, 999, 768 cm^{-1}.
HPLC: The enantiomeric ratio (96:4) was determined *via* HPLC analysis using a CHIRALCEL® IA column, with hexane:isopropanol = 95: 5 at a flow rate 1.0 mL/min detected at 254 nm wavelength. Retention time: t_{major} = 12.1 min and t_{minor} = 18.3 min.

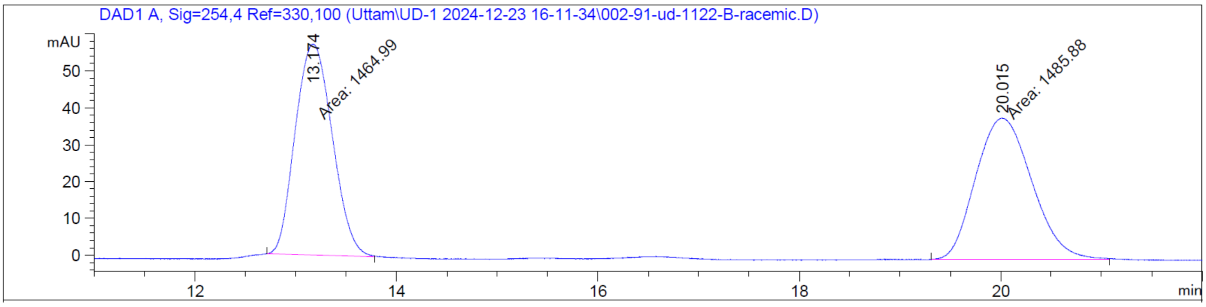

Peak #	RetTime [min]	Type	Width [min]	Area [mAU*s]	Height [mAU]	Area %
1	13.174	MM	0.4263	1464.99121	57.28112	49.6460
2	20.015	MM	0.6456	1485.88281	38.35704	50.3540

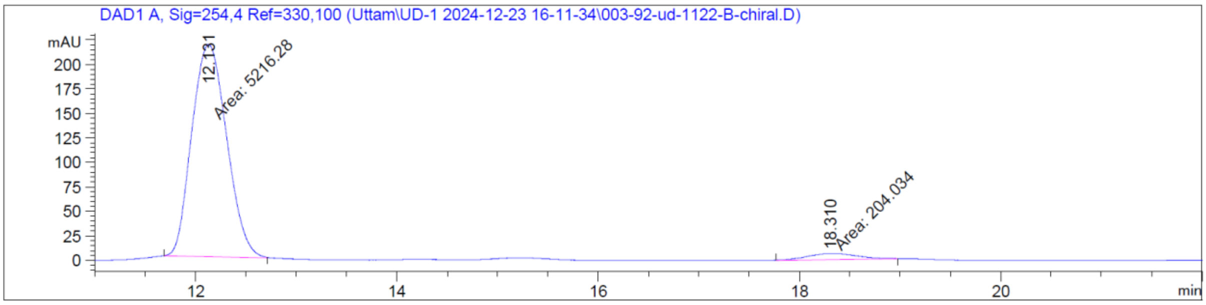

Peak #	RetTime [min]	Type	Width [min]	Area [mAU*s]	Height [mAU]	Area %
1	12.131	MM	0.4008	5216.28320	216.92879	96.2358
2	18.310	MM	0.5604	204.03377	6.06827	3.7642

A modified general procedure **GP2** was followed with 2-bromo-2-fluoro-*N*-(4-methoxyphenyl)acetamide (**2a**) (26.2 mg, 0.10 mmol, 1.0 equiv.) and *cis*-3-hexene (**4e'**) (16.8 mg, 0.20 mmol, 2.0 equiv.) at RT. Automated flash column chromatography (10 g SiO_2, gradient elution: hexane to 20% EtOAc in hexane) afforded the desired product (+) **5ea** as a white solid (10.5 mg, 40%).

HPLC: The enantiomeric ratio (96:4) was determined *via* HPLC analysis using a CHIRALCEL® IA column, with hexane:isopropanol = 95:5 at a flow rate 1.0 mL/min detected at 254 nm wavelength. Retention time: t_{major} = 11.9 min and t_{minor} = 18.0 min.

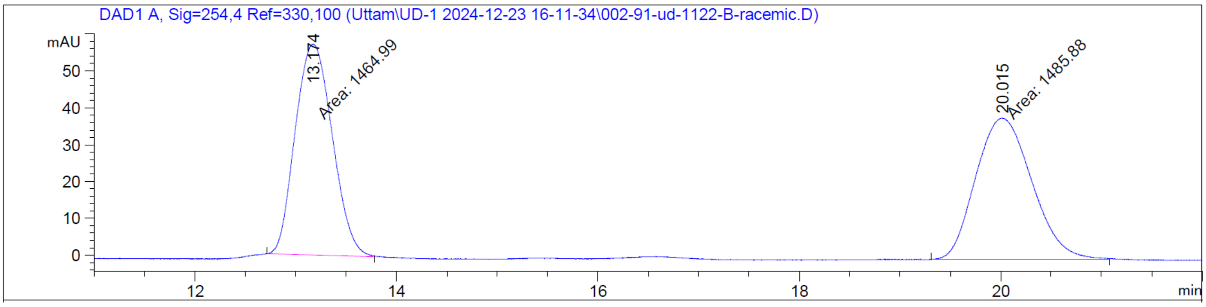

Peak #	RetTime [min]	Type	Width [min]	Area [mAU*s]	Height [mAU]	Area %
1	13.174	MM	0.4263	1464.99121	57.28112	49.6460
2	20.015	MM	0.6456	1485.88281	38.35704	50.3540

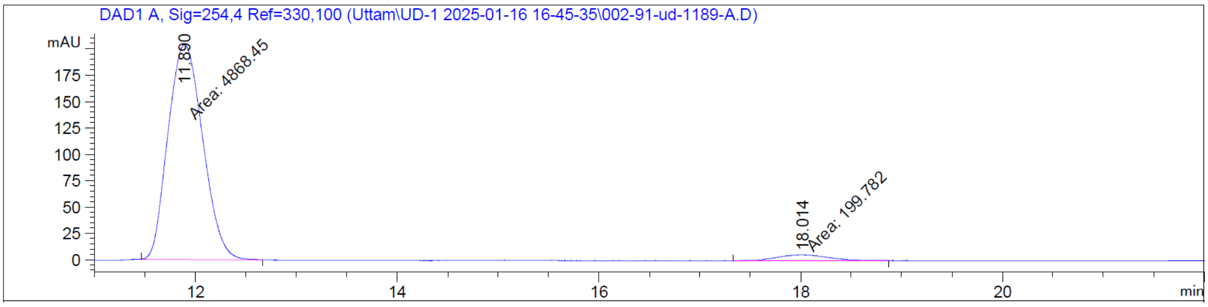

Peak #	RetTime [min]	Type	Width [min]	Area [mAU*s]	Height [mAU]	Area %
1	11.890	MM	0.3979	4868.44629	203.90430	96.0582
2	18.014	MM	0.5948	199.78180	5.59808	3.9418

(*R*)-7-Fluoro-8-((4-methoxyphenyl)amino)-8-oxooctyl benzoate (5fa)

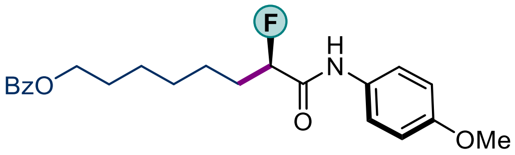

A modified general procedure **GP2** was followed with 2-bromo-2-fluoro-*N*-(4-methoxyphenyl)acetamide (**2a**) (26.2 mg, 0.10 mmol, 1.0 equiv.) and (*Z*)-hex-4-en-1-yl benzoate (**4f**) (40.9 mg, 0.20 mmol, 2.0 equiv.) at RT. Automated flash column chromatography (10 g SiO_2, gradient elution: hexane to 30% EtOAc in hexane) afforded the desired product (+) **5fa** as a white solid (11 mg, 41%).

^{1}H NMR (400 MHz, $CDCl_3$) δ 7.88 (d, J = 6.4 Hz, 1H), 7.50 – 7.42 (m, 2H), 6.92 – 6.84 (m, 2H), 4.99 (ddd, J = 50.0, 7.7, 3.6 Hz, 1H), 3.80 (s, 3H), 2.13 – 1.99 (m, 1H), 1.98 – 1.84 (m, 1H), 1.55 – 1.44 (m, 2H), 1.38 – 1.24 (m, 6H), 0.88 (t, J = 7.1 Hz, 3H).

^{13}C NMR (101 MHz, $CDCl_3$) δ 167.97 (d, $^2J_{C–F}$ = 17.9 Hz), 156.83, 129.81, 121.76, 114.26, 92.29 (d, $^1J_{C–F}$ = 186.5 Hz), 55.50, 32.45 (d, $^2J_{C–F}$ = 19.8 Hz), 31.57, 28.83, 24.37 (d, $^3J_{C–F}$ = 2.7 Hz), 22.53, 14.04.

^{19}F{H} NMR (376 MHz, $CDCl_3$) δ -188.09.

^{19}F NMR (376 MHz, $CDCl_3$) δ -188.09 (dddd, J = 50.0, 29.8, 26.3, 7.0 Hz).

HRMS (nanochip-ESI/LTQ-Orbitrap) m/z: $[M + Na]^+$ Calcd for $C_{22}H_{26}FNNaO_4^+$ 410.1744; Found 410.1733.

$[\alpha]_D^{23}$ = +22.9 (c = 0.75 in $CHCl_3$).

FTIR (neat): $\tilde{\nu}$ = 2930, 1716, 1679, 1600, 1536, 1512, 1274, 1112, 712 cm^{-1}.

HPLC: The enantiomeric ratio (97:3) was determined *via* HPLC analysis using a CHIRALCEL® IA column, with hexane:isopropanol = 75:25 at a flow rate 1.0 mL/min detected at 254 nm wavelength. Retention time: t_{major} = 9.9 min and t_{minor} = 16.9 min.

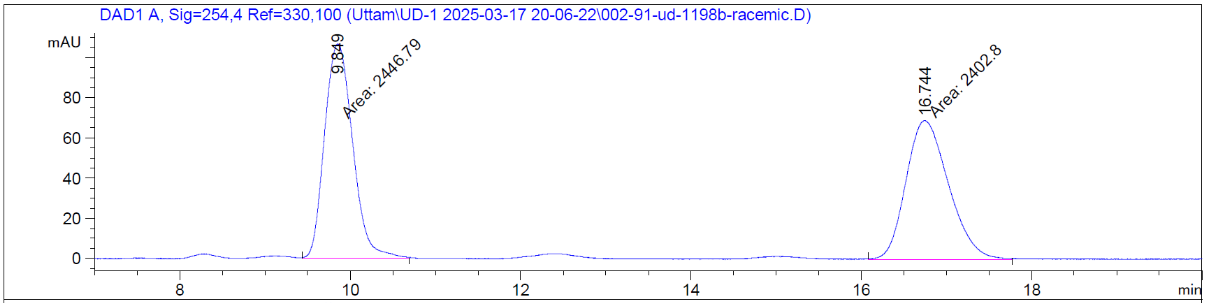

Peak #	RetTime [min]	Type	Width [min]	Area [mAU*s]	Height [mAU]	Area %
1	9.849	MM	0.3825	2446.78760	106.61805	50.4536
2	16.744	MM	0.5802	2402.79663	69.02793	49.5464

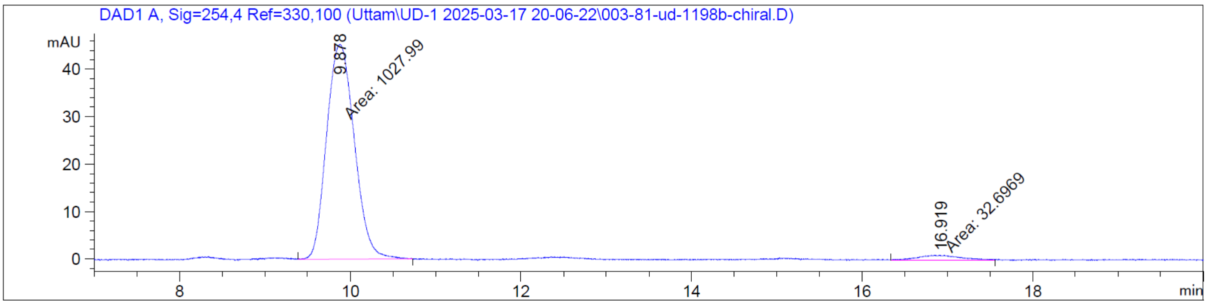

Peak #	RetTime [min]	Type	Width [min]	Area [mAU*s]	Height [mAU]	Area %
1	9.878	MM	0.3777	1027.98596	45.35852	96.9174
2	16.919	MM	0.5438	32.69690	1.00216	3.0826

(*R*,*E*)-5-cyclohexyl-N-(4-methoxyphenyl)-2-(trifluoromethyl)pent-4-enamide (7aa)

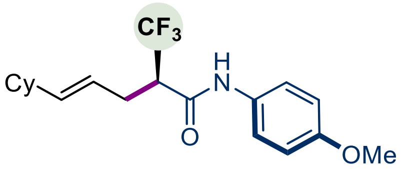

A modified general procedure **GP1** was followed with 2-bromo-3,3,3-trifluoro-*N*-(4-methoxyphenyl)propanamide (**6a**) (31.2 mg, 0.10 mmol, 1.0 equiv.) and propa-1,2-dienylcyclohexane (**1a**) (24.4 mg, 0.20 mmol, 2.0 equiv.) at 0 °C. Automated flash column chromatography (10 g SiO_2, gradient elution: hexane to 20% EtOAc in hexane) afforded the desired product (–) **7aa** as a white solid (21.5 mg, 60%).

^{1}H NMR (400 MHz, $CDCl_3$) δ 7.42 (s, 1H), 7.38 – 7.33 (m, 2H), 6.87 – 6.81 (m, 2H), 5.57 (dd, J = 15.4, 6.8 Hz, 1H), 5.33 (dt, J = 14.8, 7.0 Hz, 1H), 3.78 (s, 3H), 3.02 (dqd, J = 10.2, 8.4, 4.3 Hz, 1H), 2.67 (ddd, J = 14.2, 10.3, 7.2 Hz, 1H), 2.57 – 2.41 (m, 1H), 1.90 (dtt, J = 11.1, 7.5, 3.5 Hz, 1H), 1.74 – 1.55 (m, 5H), 1.28 – 0.96 (m, 5H).

^{13}C NMR (101 MHz, $CDCl_3$) δ 163.94 (d, $^3J_{C–F}$ = 2.8 Hz), 157.02, 141.25, 129.95, 124.98 (q, $^1J_{C–F}$ = 280.7 Hz), 122.43, 121.30, 114.17, 55.48, 52.66 (q, $^2J_{C–F}$ = 26.1 Hz), 40.58, 32.84, 29.50 (d, $^3J_{C–F}$ = 2.4 Hz), 26.07, 25.91.

^{19}F{H} NMR (376 MHz, $CDCl_3$) δ –67.75.

^{19}F NMR (376 MHz, $CDCl_3$) δ -67.75 (d, J = 8.4 Hz).

HRMS (ESI/QTOF) m/z: $[M + H]^+$ Calcd for $C_{19}H_{25}F_3NO_2^+$ 356.1837; Found 356.1836.

$[\alpha]_D^{23}$ = –50.7 (c = 0.75 in $CHCl_3$).

FTIR (neat): $\tilde{\nu}$ = 3294, 2921, 1660, 1541, 1514, 1238, 1112, 829 cm^{-1}.

HPLC: The enantiomeric ratio (93.5:6.5) was determined *via* HPLC analysis using a CHIRALCEL® IB column, with hexane:isopropanol = 95:5 at a flow rate 1.0 mL/min detected at 254 nm wavelength. Retention time: t_{major} = 14.5 min and t_{minor} = 20.0 min.

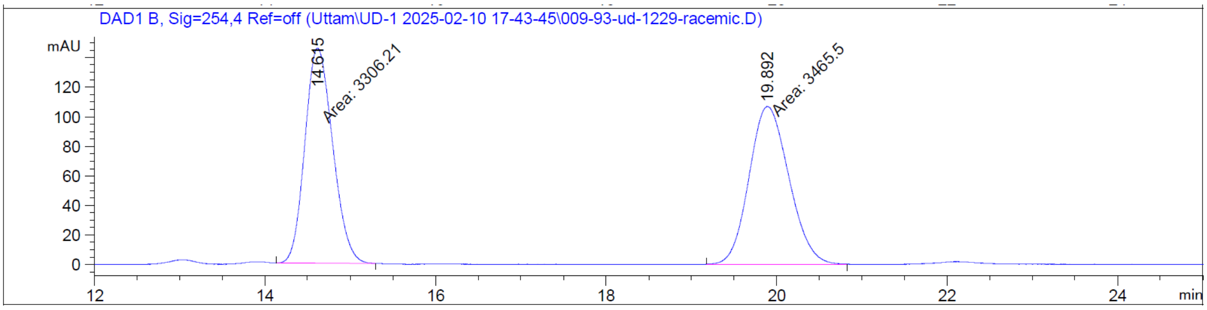

Peak #	RetTime [min]	Type	Width [min]	Area [mAU*s]	Height [mAU]	Area %
1	14.615	MM	0.3782	3306.21143	145.71811	48.8239
2	19.892	MM	0.5407	3465.50073	106.82378	51.1761

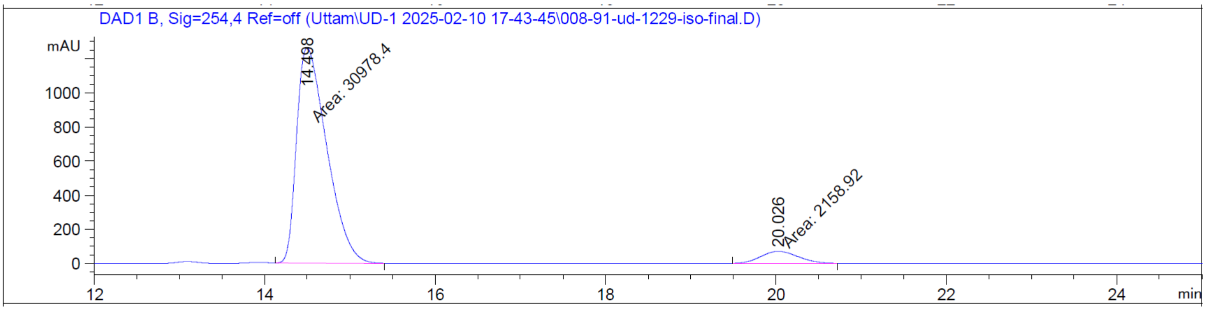

Peak #	RetTime [min]	Type	Width [min]	Area [mAU*s]	Height [mAU]	Area %
1	14.498	MM	0.4097	3.09784e4	1260.16992	93.4849
2	20.026	MM	0.5075	2158.91699	70.90366	6.5151

Methyl (*R*,*E*)-9,9,9-trifluoro-8-((4-methoxyphenyl)carbamoyl)non-5-enoate (7da)

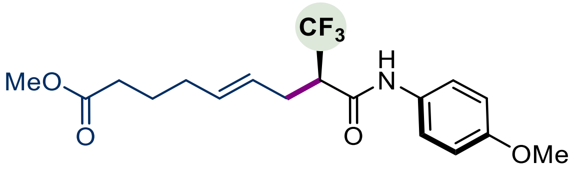

A modified general procedure **GP1** was followed with 2-bromo-3,3,3-trifluoro-*N*-(4-methoxyphenyl)propanamide (**6a**) (31.2 mg, 0.10 mmol, 1.0 equiv.) and methyl hepta-5,6-dienoate (**1d**) (28 mg, 0.20 mmol, 2.0 equiv.) at 0 °C. Automated flash column chromatography (10 g SiO_2, gradient elution: hexane to 50% EtOAc in hexane) afforded the desired product (–) **7da** as a white solid (24.5 mg, 66%).

^{1}H NMR (400 MHz, $CDCl_3$) δ 7.49 (s, 1H), 7.43 (dd, *J* = 9.0, 2.2 Hz, 2H), 6.89 – 6.84 (m, 2H), 5.57 (dt, *J* = 15.2, 6.8 Hz, 1H), 5.42 (dt, *J* = 14.9, 7.1 Hz, 1H), 3.79 (s, 3H), 3.65 (s, 3H), 2.99 (dqd, *J* = 10.5, 8.4, 4.2 Hz, 1H), 2.78 – 2.66 (m, 1H), 2.56 – 2.42 (m, 1H), 2.26 (q, *J* = 7.4 Hz, 2H), 2.10 – 1.96 (m, 2H), 1.68 (pd, *J* = 7.0, 2.8 Hz, 2H).

^{13}C NMR (101 MHz, $CDCl_3$) δ 174.29, 163.62 (d, $^{3}J_{C–F}$ = 2.4 Hz), 156.93, 133.71, 130.17, 124.90 (q, $^{1}J_{C–F}$ = 280.8 Hz), 122.06, 121.95, 114.20, 55.51, 52.62 (q, $^{2}J_{C–F}$ = 26.1 Hz), 51.57, 33.34, 31.84, 29.38 (d, $^{3}J_{C–F}$ = 2.3 Hz), 24.12.

^{19}F{H} NMR (376 MHz, $CDCl_3$) δ -67.91.

^{19}F NMR (376 MHz, $CDCl_3$) δ -67.92 (d, *J* = 8.3 Hz).

HRMS (ESI/QTOF) m/z: $[M + H]^+$ Calcd for $C_{18}H_{23}F_3NO_4^+$ 374.1579; Found 374.1575.

$[\alpha]_D^{23}$ = –37.3 (c = 0.33 in $CHCl_3$).

FTIR (neat): $\tilde{\nu}$ = 2929, 1733, 1665, 1545, 1240, 1114, 823 cm^{-1}.

HPLC: The enantiomeric ratio (93:7) was determined *via* HPLC analysis using a CHIRALCEL® IB column, with hexane:isopropanol = 95:5 at a flow rate 1.0 mL/min detected at 254 nm wavelength. Retention time: t_{major} = 19.5 min and t_{minor} = 25.1 min.

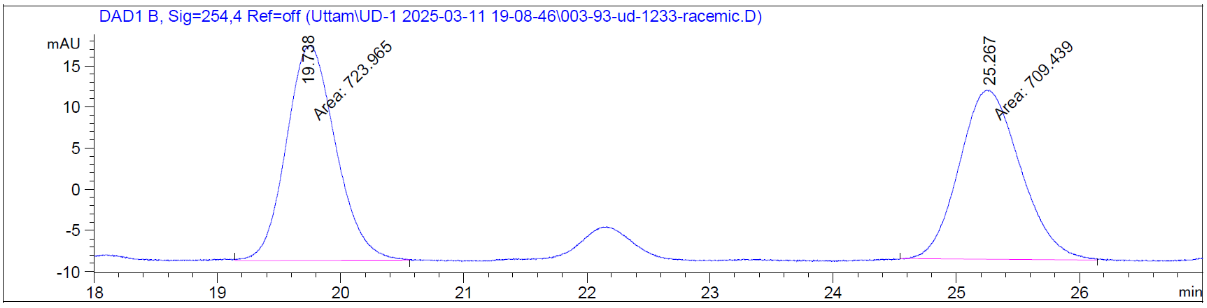

Peak #	RetTime [min]	Type	Width [min]	Area [mAU*s]	Height [mAU]	Area %
1	19.738	MM	0.4627	723.96478	26.07932	50.5067
2	25.267	MM	0.5756	709.43927	20.54253	49.4933

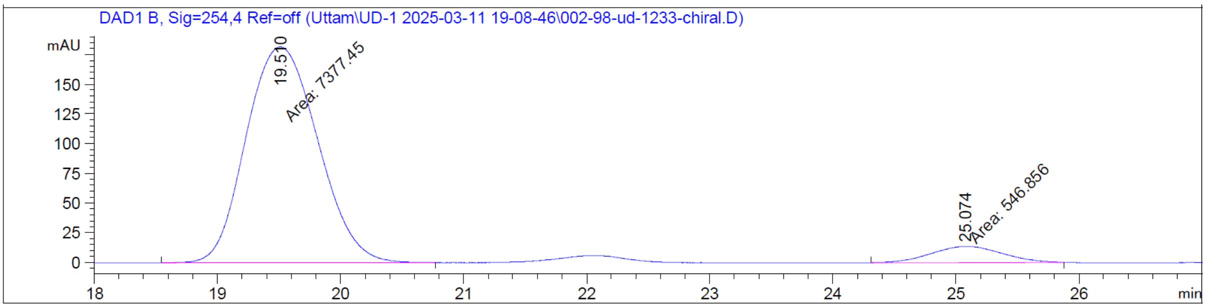

Peak #	RetTime [min]	Type	Width [min]	Area [mAU*s]	Height [mAU]	Area %
1	19.510	MM	0.6762	7377.45361	181.82332	93.0990
2	25.074	MM	0.6704	546.85608	13.59621	6.9010

(*R*,*E*)-*N*-(3-Chlorophenyl)-5-cyclohexyl-2-(trifluoromethyl)pent-4-enamide (7ab)

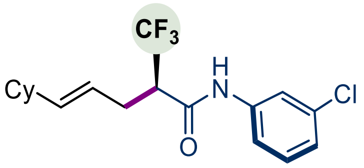

A modified general procedure **GP1** was followed with 2-bromo-*N*-(3-chlorophenyl)-3,3,3-trifluoropropanamide (**6b**) (31.5 mg, 0.10 mmol, 1.0 equiv.) and propa-1,2-dienylcyclohexane (**1a**) (24.4 mg, 0.20 mmol, 2.0 equiv.) at 0 °C. Automated flash column chromatography (10 g SiO_2, gradient elution: hexane to 20% EtOAc in hexane) afforded the desired product (–) **7ab** as a white solid (23 mg, 64%).

^{1}H NMR (400 MHz, $CDCl_3$) δ 7.65 – 7.55 (m, 1H), 7.43 (s, 1H), 7.38 – 7.29 (m, 1H), 7.29 – 7.20 (m, 1H), 7.18 – 7.08 (m, 1H), 5.58 (dd, J = 15.4, 6.8 Hz, 1H), 5.33 (dt, J = 14.9, 7.1 Hz, 1H), 3.03 (dtd, J = 16.7, 8.3, 4.3 Hz, 1H), 2.68 (ddd, J = 14.1, 10.2, 7.3 Hz, 1H), 2.60 – 2.46 (m, 1H), 1.90 (dtd, J = 12.0, 7.8, 3.8 Hz, 1H), 1.70 – 1.50 (m, 5H), 1.26 – 0.93 (m, 5H).

^{13}C NMR (101 MHz, $CDCl_3$) δ 164.13 (d, $^{3}J_{C\text{–}F}$ = 2.8 Hz), 141.65, 137.96, 134.79, 130.06, 125.25, 124.78 (d, $^{1}J_{C\text{–}F}$ = 280.7 Hz), 120.98, 120.54, 118.31, 52.94 (q, $^{2}J_{C\text{–}F}$ = 26.1 Hz), 40.55, 32.81, 29.47 (d, $^{3}J_{C\text{–}F}$ = 2.4 Hz), 26.04, 25.87.

^{19}F{H} NMR (376 MHz, $CDCl_3$) δ -67.66.

^{19}F NMR (376 MHz, $CDCl_3$) δ -67.66 (d, J = 8.3 Hz).

HRMS (ESI/QTOF) m/z: $[M + H]^+$ Calcd for $C_{18}H_{22}ClF_3NO^+$ 360.1342; Found 360.1338.

$[\alpha]_D^{23}$ = -2.0 (c = 1.00 in $CHCl_3$).

FTIR (neat): $\tilde{\nu}$ = 2951, 1673, 1534, 1512, 1466, 1233, 758 cm^{-1}.

HPLC: The enantiomeric ratio (90.5:9.5) was determined *via* HPLC analysis using a CHIRALCEL® IB column, with hexane:isopropanol = 99:1 at a flow rate 1.0 mL/min detected at 254 nm wavelength. Retention time: t_{major} = 11.1 min and t_{minor} = 14.9 min.

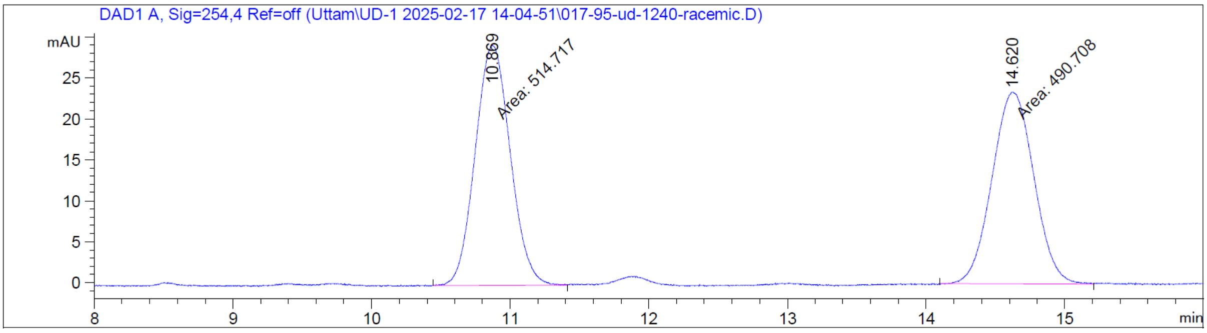

Peak #	RetTime [min]	Type	Width [min]	Area [mAU*s]	Height [mAU]	Area %
1	10.869	MM	0.2929	514.71710	29.29239	51.1940
2	14.620	MM	0.3495	490.70810	23.39993	48.8060

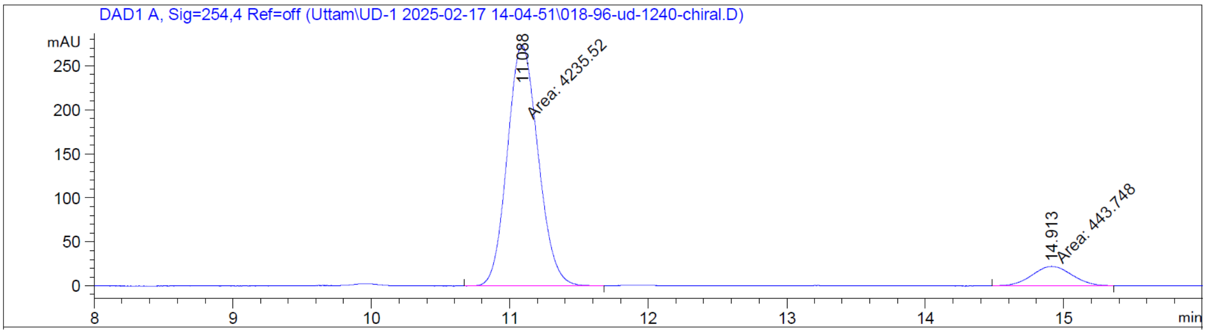

Peak #	RetTime [min]	Type	Width [min]	Area [mAU*s]	Height [mAU]	Area %
1	11.088	MM	0.2583	4235.52246	273.26920	90.5167
2	14.913	MM	0.3335	443.74768	22.17931	9.4833

(*R*,*E*)-5-Cyclohexyl-*N*-(1-methyl-1*H*-indol-5-yl)-2-(trifluoromethyl)pent-4-enamide (7ac)

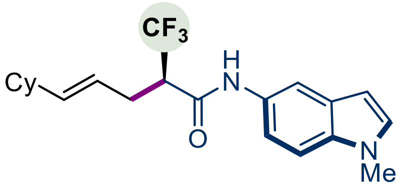

A modified general procedure **GP1** was followed with 2-bromo-3,3,3-trifluoro-*N*-(1-methyl-1*H*-indol-5-yl)propanamide (**6c**) (33.5 mg, 0.10 mmol, 1.0 equiv.) and propa-1,2-dienylcyclohexane (**1a**) (24.4 mg, 0.20 mmol, 2.0 equiv.) at 0 °C. Automated flash column chromatography (10 g SiO_2, gradient elution: hexane to 30% EtOAc in hexane) afforded the desired product (-) **7ac** as a white solid (19.5 mg, 52%).

^{1}H NMR (400 MHz, $CDCl_3$) δ 7.72 (d, *J* = 1.9 Hz, 1H), 7.20 (s, 1H), 7.18 – 7.14 (m, 2H), 6.99 (d, *J* = 3.1 Hz, 1H), 6.37 (dd, *J* = 3.0, 0.8 Hz, 1H), 5.58 – 5.48 (m, 1H), 5.32 (dt, *J* = 14.9, 7.0 Hz, 1H), 3.71 (s, 3H), 2.95 (dqd, *J* = 10.0, 8.5, 4.3 Hz, 1H), 2.65 (ddd, *J* = 14.1, 10.2, 7.1 Hz, 1H), 2.47 (dt, *J* = 14.2, 5.6 Hz, 1H), 1.89 – 1.78 (m, 1H), 1.64 – 1.53 (m, 5H), 1.15 – 0.92 (m, 5H).

13C NMR (101 MHz, CDCl3) δ 163.69 (d, $^3J_{C–F}$ = 2.7 Hz), 141.17, 134.59, 129.90, 129.12, 128.48, 125.09 (q, $^1J_{C–F}$ = 280.4 Hz), 121.49, 116.08, 113.45, 109.35, 101.14, 52.99 (q, $^2J_{C–F}$ = 25.6 Hz), 40.60, 32.98, 32.86, 29.57 (d, $^3J_{C–F}$ = 2.3 Hz), 26.09, 25.93.

^{19}F{H} NMR (376 MHz, $CDCl_3$) δ -67.71.

^{19}F NMR (376 MHz, $CDCl_3$) δ -67.71 (d, *J* = 8.3 Hz).

HRMS (ESI/QTOF) m/z: $[M + H]^+$ Calcd for $C_{21}H_{26}F_3N_2O^+$ 379.1997; Found 379.1991.

$[\alpha]_D^{23}$ = -27.5 (c = 0.33 in $CHCl_3$).

FTIR (neat): $\tilde{\nu}$ = 3295, 1659, 1547, 1493, 1446, 1246, 1161, 787 cm^{-1}.

HPLC: The enantiomeric ratio (93:7) was determined *via* HPLC analysis using a CHIRALCEL® IB column, with hexane:isopropanol = 90:10 at a flow rate 1.0 mL/min detected at 254 nm wavelength. Retention time: t_{major} = 20.0 min and t_{minor} = 31.7 min.

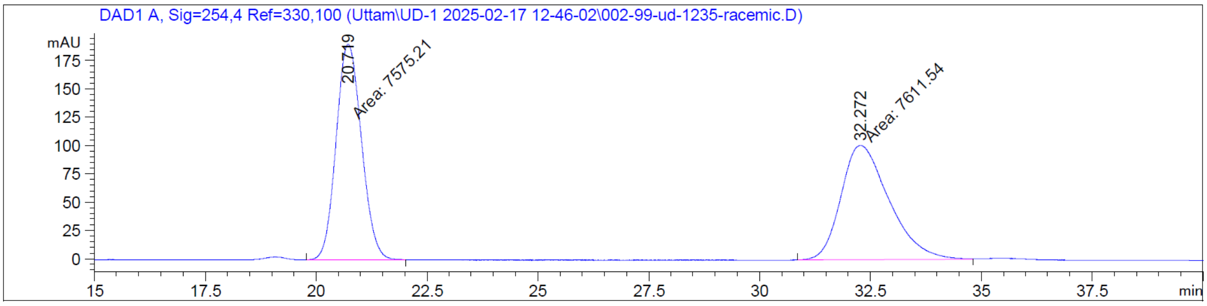

Peak #	RetTime [min]	Type	Width [min]	Area [mAU*s]	Height [mAU]	Area %
1	20.719	MM	0.6625	7575.20605	190.57013	49.8804
2	32.272	MM	1.2559	7611.53613	101.01212	50.1196

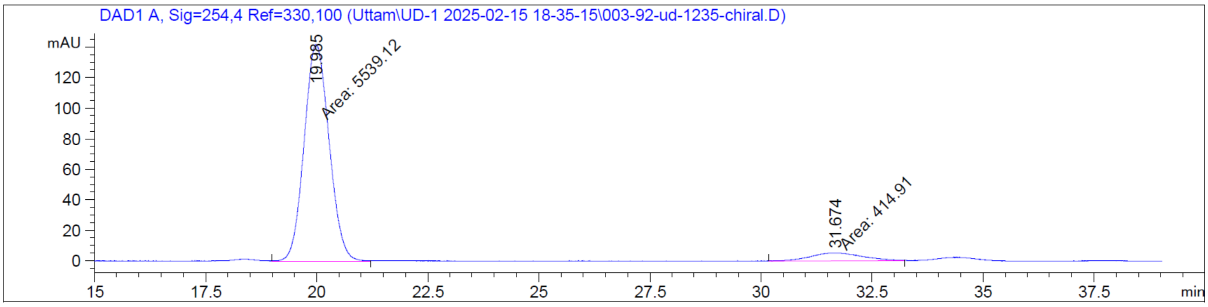

Peak #	RetTime [min]	Type	Width [min]	Area [mAU*s]	Height [mAU]	Area %
1	19.985	MM	0.6502	5539.11816	141.98282	93.0314
2	31.674	MM	1.3277	414.91000	5.20849	6.9686

Methyl (*R*,*E*)-9,9,9-trifluoro-8-phenylnon-5-enoate (7dd)

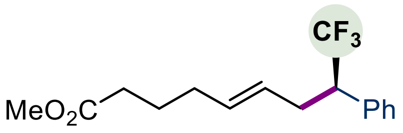

A modified **GP1** was followed with (1-bromo-2,2,2-trifluoroethyl)benzene (**6d**) (23.9 mg, 0.10 mmol, 1.0 equiv.) and methyl hepta-5,6-dienoate (**1d**) (28 mg, 0.20 mmol, 2.0 equiv.) at -20 °C. Automated flash column chromatography (10 g SiO_2, gradient elution: hexane to 20% EtOAc in hexane) afforded the desired product (-) **7dd** as a colourless oil (18 mg, 60%).

^{1}H NMR (400 MHz, $CDCl_3$) δ 7.40 – 7.04 (m, 5H), 5.30 (dtt, *J* = 15.0, 6.7, 1.3 Hz, 1H), 5.11 (dt, *J* = 14.8, 7.0 Hz, 1H), 3.57 (s, 3H), 3.19 (dqd, *J* = 10.7, 9.3, 4.7 Hz, 1H), 2.64 (dddd, *J* =

14.1, 6.9, 4.6, 1.1 Hz, 1H), 2.55 – 2.42 (m, 1H), 2.10 – 1.98 (m, 2H), 1.92 – 1.72 (m, 2H), 1.54 – 1.40 (m, 2H).

^{13}C NMR (101 MHz, $CDCl_3$) δ 174.03, 134.5 (q, $^3J_{C–F}$ = 2.1 Hz), 132.65, 129.13, 128.57, 128.06, 127.20 (q, $^1J_{C–F}$ = 276.1 Hz), 126.44, 51.43, 50.47 (q, $^2J_{C–F}$ = 25.9 Hz), 32.99, 32.18 (q, $^3J_{C–F}$ = 2.6 Hz), 31.62, 24.29.

^{19}F{H} NMR (376 MHz, $CDCl_3$) δ -69.56.

^{19}F NMR (376 MHz, $CDCl_3$) δ -69.56 (d, J = 9.4 Hz).

HRMS (ESI/QTOF) m/z: $[M + H]^+$ Calcd for $C_{16}H_{20}F_3O_2^+$ 301.1415; Found 301.1410.

$[\alpha]_D^{23}$ = -35.6 (c = 0.75 in $CHCl_3$).

FTIR (neat): $\tilde{\nu}$ = 2928, 1735, 1437, 1256, 1158, 1111, 702 cm^{-1}.

HPLC: The enantiomeric ratio (92:8) was determined *via* HPLC analysis using a CHIRALCEL® OD-H column, with hexane:isopropanol = 99:1 at a flow rate 1.0 mL/min detected at 210 nm wavelength. Retention time: t_{major} = 5.8 min and t_{minor} = 5.4 min.

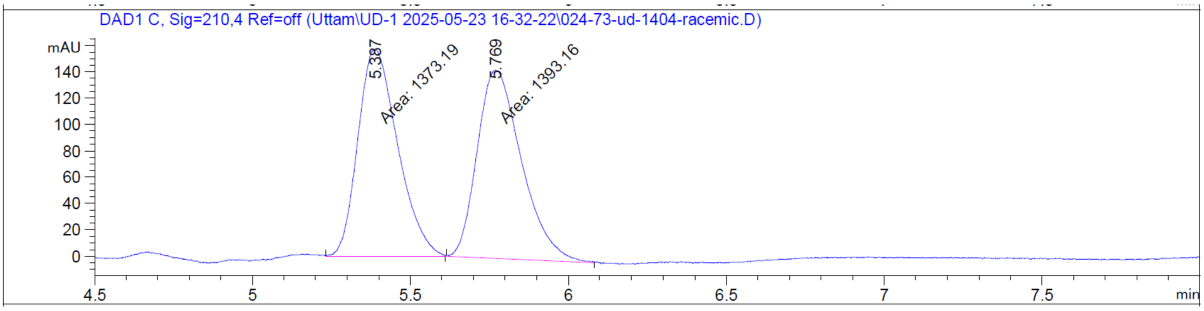

Peak #	RetTime [min]	Type	Width [min]	Area [mAU*s]	Height [mAU]	Area %
1	5.387	MM	0.1456	1373.19360	157.18687	49.6391
2	5.769	MM	0.1628	1393.16272	142.58928	50.3609

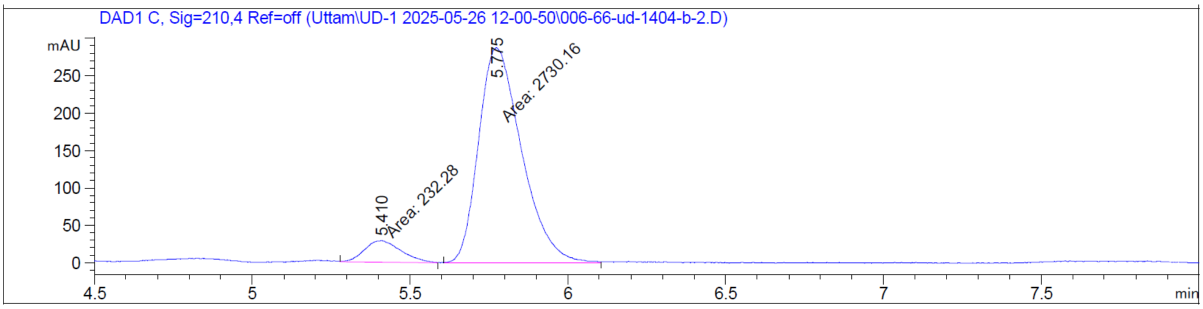

Peak #	RetTime [min]	Type	Width [min]	Area [mAU*s]	Height [mAU]	Area %
1	5.410	MM	0.1345	232.27962	28.78616	7.8408
2	5.775	MM	0.1580	2730.16382	287.96127	92.1592

(*R*,*E*)-2-Chloro-5-cyclohexyl-*N*-(4-methoxyphenyl)pent-4-enamide (9aa)

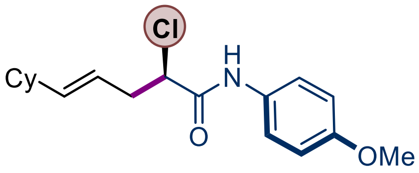

General procedure **GP1** was followed with 2-bromo-2-chloro-*N*-(4-methoxyphenyl)acetamide (**8a**) (27.9 mg, 0.10 mmol, 1.0 equiv.) and propa-1,2-dienylcyclohexane (**1a**) (24.4 mg, 0.20 mmol, 2.0 equiv.). Automated flash column chromatography (10 g SiO_2, gradient elution: hexane to 30% EtOAc in hexane) afforded the desired product (+) **9aa** as a white solid (25.5 mg, 80%).

^{1}H NMR (400 MHz, $CDCl_3$) δ 8.12 (bs, 1H), 7.53 – 7.35 (m, 2H), 6.97 – 6.81 (m, 2H), 5.58 (ddt, *J* = 15.4, 6.6, 1.2 Hz, 1H), 5.41 (dtd, *J* = 15.3, 6.9, 1.2 Hz, 1H), 4.45 (dd, *J* = 7.2, 4.5 Hz, 1H), 3.80 (s, 3H), 2.84 (dddt, *J* = 14.6, 6.6, 4.6, 0.9 Hz, 1H), 2.72 (dtt, *J* = 14.5, 7.2, 0.8 Hz, 1H), 1.94 (dtt, *J* = 10.3, 6.5, 3.3 Hz, 1H), 1.76 – 1.60 (m, 5H), 1.30 – 1.00 (m, 5H).

^{13}C NMR (101 MHz, $CDCl_3$) δ 166.34, 156.96, 141.95, 129.93, 122.02, 120.70, 114.22, 61.25, 55.50, 40.69, 38.77, 32.89, 26.14, 25.95.

HRMS (ESI/QTOF) m/z: $[M + H]^+$ Calcd for $C_{18}H_{25}ClNO_2^+$ 322.1574; Found 322.1570.

$[\alpha]_D^{23}$ = +52.1 (c = 0.50 in $CHCl_3$).

FTIR (neat): $\tilde{\nu}$ = 3276, 1661, 1606, 1544, 1510, 1447, 1172, 970 cm^{-1}.

HPLC: The enantiomeric ratio (98:2) was determined *via* HPLC analysis using a CHIRALCEL® OD-H column, with hexane:isopropanol = 95:5 at a flow rate 1.0 mL/min detected at 254 nm wavelength. Retention time: t_{major} = 17.7 min and t_{minor} = 24.5 min.

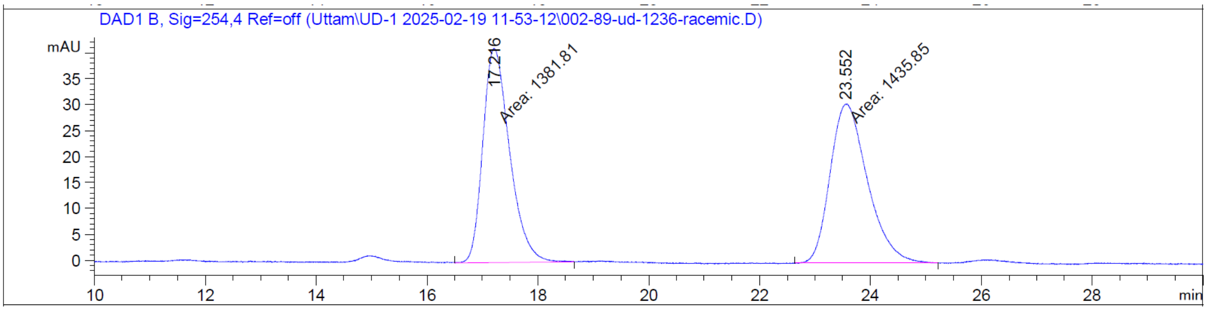

Peak #	RetTime [min]	Type	Width [min]	Area [mAU*s]	Height [mAU]	Area %
1	17.216	MM	0.5577	1381.80652	41.29115	49.0409
2	23.552	MM	0.7802	1435.85461	30.67184	50.9591

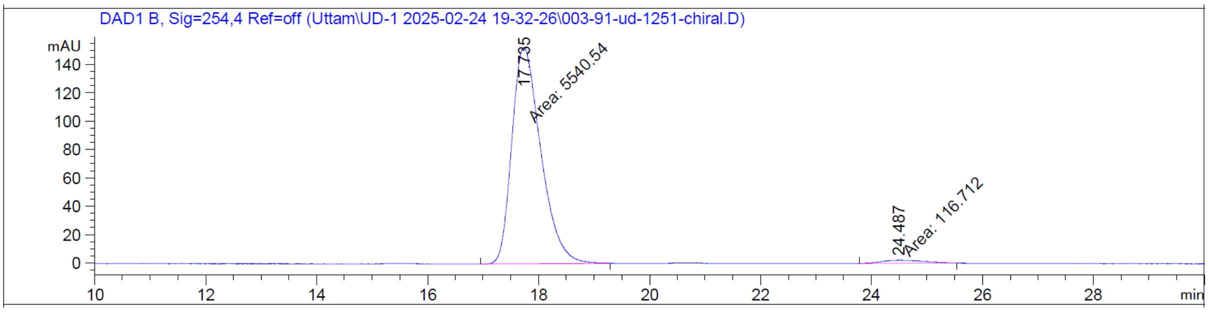

Peak #	RetTime [min]	Type	Width [min]	Area [mAU*s]	Height [mAU]	Area %
1	17.735	MM	0.6051	5540.54248	152.59973	97.9369
2	24.487	MM	0.8430	116.71201	2.30740	2.0631

(*R*,*E*)-2-Chloro-8-(1,3-dioxoisoindolin-2-yl)-*N*-(4-methoxyphenyl)oct-4-enamide (9ea)

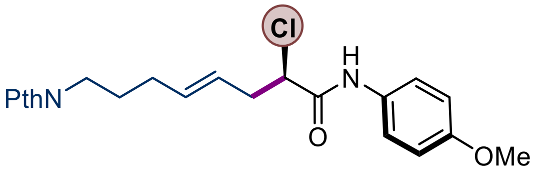

General procedure **GP1** was followed with 2-bromo-2-chloro-*N*-(4-methoxyphenyl)acetamide (**8a**) (27.9 mg, 0.10 mmol, 1.0 equiv.) and 2-(hexa-4,5-dien-1-yl)isoindoline-1,3-dione (45.5 mg, 0.20 mmol, 2.0 equiv.). Automated flash column chromatography (10 g SiO_2, gradient elution: hexane to 30% EtOAc in hexane) afforded the desired product (+) **9ea** as a white solid (37 mg, 87%).

^{1}H NMR (400 MHz, $CDCl_3$) δ 8.25 (s, 1H), 7.81 (dd, *J* = 5.4, 3.1 Hz, 2H), 7.70 (dd, *J* = 5.5, 3.0 Hz, 2H), 7.50 – 7.39 (m, 2H), 6.92 – 6.77 (m, 2H), 5.62 (dt, *J* = 15.2, 6.5 Hz, 1H), 5.57 – 5.41 (m, 1H), 4.45 (dd, *J* = 6.9, 4.7 Hz, 1H), 3.75 (s, 3H), 3.64 (t, *J* = 7.3 Hz, 2H), 2.82 (dt, *J* = 14.2, 5.5 Hz, 1H), 2.73 (dt, *J* = 14.3, 7.0 Hz, 1H), 2.08 (q, *J* = 7.1 Hz, 2H), 1.78 – 1.69 (m, 2H).

^{13}C NMR (101 MHz, $CDCl_3$) δ 168.40, 166.28, 156.88, 134.22, 133.91, 132.13, 130.04, 124.66, 123.18, 121.98, 114.16, 60.77, 55.45, 38.59, 37.35, 29.68, 27.84.

HRMS (ESI/QTOF) m/z: $[M + H]^+$ Calcd for $C_{23}H_{24}ClN_2O_4^+$ 427.1424; Found 427.1419.

$[\alpha]_D^{23}$ = +52.8 (c = 1.00 in $CHCl_3$).

FTIR (neat): $\tilde{\nu}$ = 3306, 1705, 1656, 1535, 1510, 1244, 1171, 1032, 830 cm^{-1}.

HPLC: The enantiomeric ratio (98:2) was determined *via* HPLC analysis using a CHIRALCEL® OD-H column, with hexane:isopropanol = 80:20 at a flow rate 1.0 mL/min detected at 254 nm wavelength. Retention time: t_{major} = 16.7 min and t_{minor} = 21.0 min.

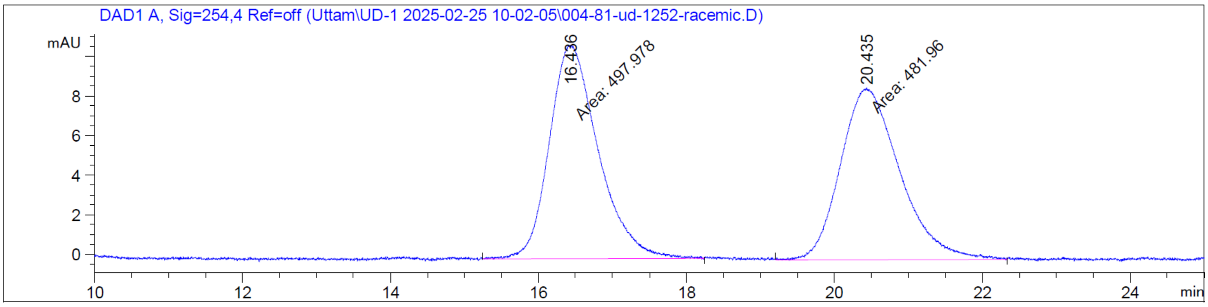

Peak #	RetTime [min]	Type	Width [min]	Area [mAU*s]	Height [mAU]	Area %
1	16.436	MM	0.7672	497.97787	10.81818	50.8173
2	20.435	MM	0.9232	481.96048	8.70079	49.1827

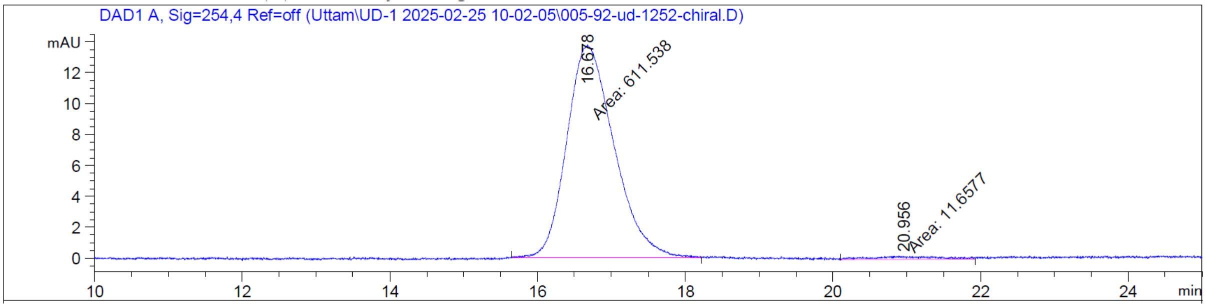

Peak #	RetTime [min]	Type	Width [min]	Area [mAU*s]	Height [mAU]	Area %
1	16.678	MM	0.7397	611.53821	13.77968	98.1294
2	20.956	MM	0.8667	11.65769	2.24188e-1	1.8706

Methyl (*R*,*E*)-4-(2-chloro-5-cyclohexylpent-4-enamido)benzoate (9ab)

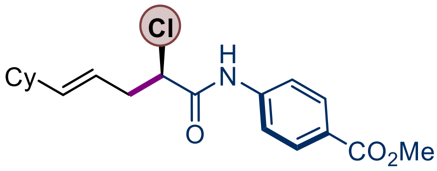

General procedure **GP1** was followed with methyl 4-(2-bromo-2-chloroacetamido)benzoate (**8b**) (30.7 mg, 0.10 mmol, 1.0 equiv.) and propa-1,2-dienylcyclohexane (**1a**) (24.4 mg, 0.20 mmol, 2.0 equiv.). Automated flash column chromatography (10 g SiO_2, gradient elution: hexane to 30% EtOAc in hexane) afforded the desired product (+) **9ab** as a white solid (27 mg, 78%).

^{1}H NMR (400 MHz, $CDCl_3$) δ 8.36 (s, 1H), 8.08 – 7.98 (m, 2H), 7.67 – 7.59 (m, 2H), 5.58 (dd, *J* = 15.4, 6.7 Hz, 1H), 5.46 – 5.33 (m, 1H), 4.47 (dd, *J* = 7.1, 4.7 Hz, 1H), 3.90 (s, 3H), 2.88 – 2.79 (m, 1H), 2.72 (dt, *J* = 14.5, 7.2 Hz, 1H), 1.93 (tdt, *J* = 10.6, 6.7, 3.3 Hz, 1H), 1.73 – 1.60 (m, 5H), 1.28 – 0.98 (m, 5H).

^{13}C NMR (101 MHz, $CDCl_3$) δ 166.71, 166.46, 142.29, 140.97, 130.86, 126.42, 120.37, 119.15, 61.09, 52.10, 40.67, 38.66, 32.84, 26.09, 25.90.

HRMS (ESI/QTOF) m/z: $[M + Na]^+$ Calcd for $C_{19}H_{24}ClNNaO_3^+$ 372.1342; Found 372.1338.

$[\alpha]_D^{23}$ = +63.9 (c = 1.00 in $CHCl_3$).

FTIR (neat): $\tilde{\nu}$ = 3020, 1720, 1684, 1601, 1525, 1435, 1252, 1111, 512 cm^{-1}.

HPLC: The enantiomeric ratio (98:2) was determined *via* HPLC analysis using a CHIRALCEL® IB column, with hexane:isopropanol = 95:5 at a flow rate 1.0 mL/min detected at 254 nm wavelength. Retention time: t_{major} = 13.8 min and t_{minor} = 16.6 min.

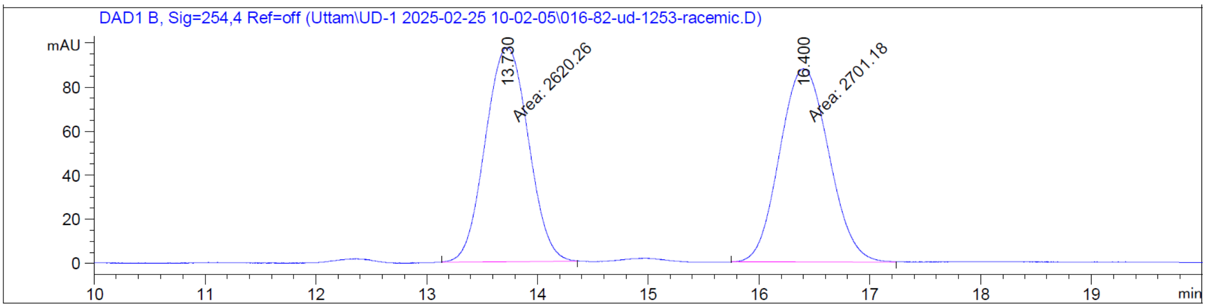

Peak #	RetTime [min]	Type	Width [min]	Area [mAU*s]	Height [mAU]	Area %
1	13.730	MM	0.4489	2620.26196	97.28535	49.2397
2	16.400	MM	0.5136	2701.18115	87.65911	50.7603

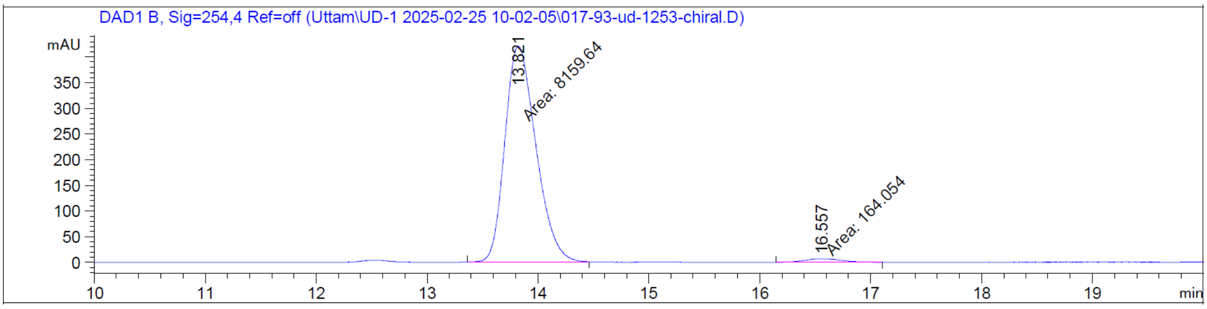

Peak #	RetTime [min]	Type	Width [min]	Area [mAU*s]	Height [mAU]	Area %
1	13.821	MM	0.3237	8159.63623	420.17377	98.0291
2	16.557	MM	0.3871	164.05441	7.06256	1.9709

(*R*,*E*)-*N*-(3-Acetylphenyl)-2-chloro-5-cyclohexylpent-4-enamide (9ac)

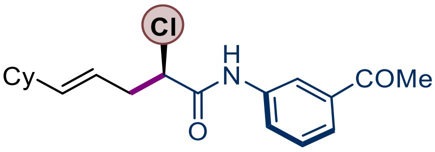

General procedure **GP1** was followed with *N*-(3-acetylphenyl)-2-bromo-2-chloroacetamide (**8c**) (29.1 mg, 0.10 mmol, 1.0 equiv.) and propa-1,2-dienylcyclohexane (**1a**) (24.4 mg, 0.20 mmol, 2.0 equiv.). Automated flash column chromatography (10 g SiO_2, gradient elution: hexane to 30% EtOAc in hexane) afforded the desired product (+) **9ac** as a white solid (20.5 mg, 62%).

^{1}H NMR (400 MHz, $CDCl_3$) δ 8.33 (s, 1H), 8.03 (t, *J* = 1.9 Hz, 1H), 7.88 (ddd, *J* = 8.1, 2.3, 1.0 Hz, 1H), 7.74 (dt, *J* = 7.9, 1.2 Hz, 1H), 7.46 (t, *J* = 7.9 Hz, 1H), 5.59 (ddt, *J* = 15.5, 6.8, 1.2 Hz, 1H), 5.41 (dtd, *J* = 15.3, 6.9, 1.2 Hz, 1H), 4.48 (dd, *J* = 7.1, 4.7 Hz, 1H), 2.91 – 2.80 (m, 1H), 2.74 (dt, *J* = 14.5, 7.1 Hz, 1H), 2.62 (s, 3H), 2.01 – 1.88 (m, 1H), 1.73 – 1.59 (m, 5H), 1.28 – 0.99 (m, 5H).

^{13}C NMR (101 MHz, $CDCl_3$) δ 197.58, 166.80, 142.24, 137.91, 137.39, 129.46, 124.87, 124.59, 120.44, 119.60, 61.09, 40.68, 38.70, 32.87, 26.69, 26.10, 25.92.

HRMS (ESI/QTOF) m/z: $[M + H]^+$ Calcd for $C_{19}H_{25}ClNO_2^+$ 334.1574; Found 334.1568.

$[\alpha]_D^{23}$ = +42.0 (c = 0.5 in $CHCl_3$).

FTIR (neat): $\tilde{\nu}$ = 3319, 1710, 1670, 1594, 1543, 1437, 1247, 970, 796 cm^{-1}.

HPLC: The enantiomeric ratio (98.5:1.5) was determined *via* HPLC analysis using a CHIRALCEL® IB column, with hexane:isopropanol = 95:5 at a flow rate 1.0 mL/min detected at 254 nm wavelength. Retention time: t_{major} = 14.3 min and t_{minor} = 18.3 min.

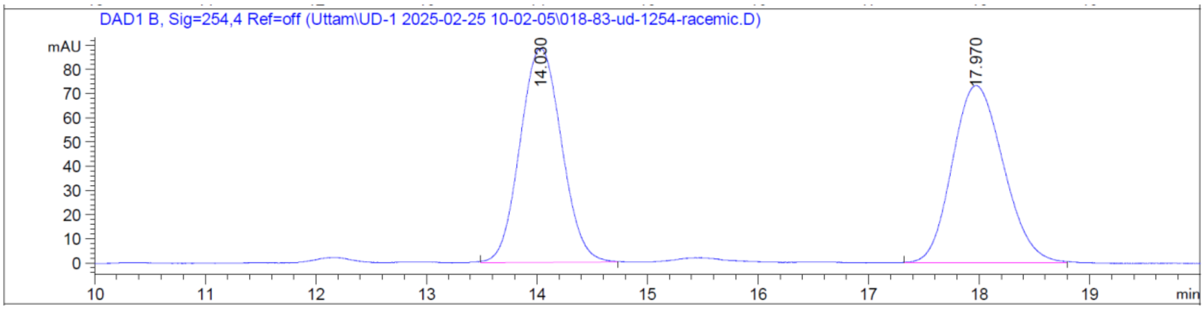

Peak #	RetTime [min]	Type	Width [min]	Area [mAU*s]	Height [mAU]	Area %
1	14.030	VV	0.3052	2303.72070	88.49225	49.5440
2	17.970	BV	0.3772	2346.12866	73.14428	50.4560

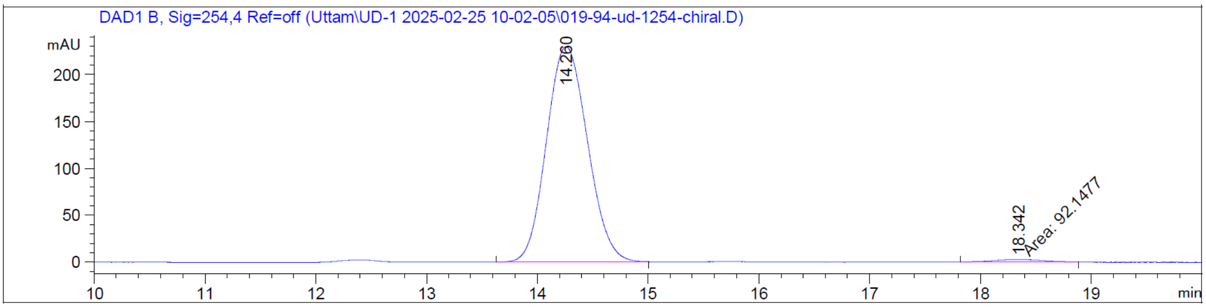

Peak #	RetTime [min]	Type	Width [min]	Area [mAU*s]	Height [mAU]	Area %
1	14.260	VV	0.3062	5923.52441	229.82776	98.4682
2	18.342	MM	0.5071	92.14767	3.02880	1.5318

(*R*,*E*)-2-Chloro-5-cyclohexyl-*N*-(1-methyl-1*H*-indol-5-yl)pent-4-enamide (9ad)

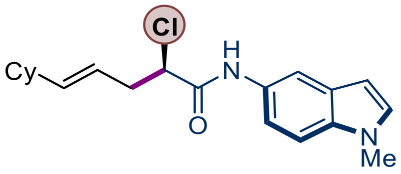

General procedure **GP1** was followed with 2-bromo-2-chloro-*N*-(1-methyl-1*H*-indol-6-yl)acetamide (**8d**) (30.2 mg, 0.10 mmol, 1.0 equiv.) and propa-1,2-dienylcyclohexane (**1a**) (24.4 mg, 0.20 mmol, 2.0 equiv.). Automated flash column chromatography (10 g SiO_2, gradient elution: hexane to 30% EtOAc in hexane) afforded the desired product (+) **9ad** as a white solid (19.5 mg, 57%).

^{1}H NMR (400 MHz, $CDCl_3$) δ 8.25 (s, 1H), 7.87 – 7.79 (m, 1H), 7.27 (d, J = 1.3 Hz, 2H), 7.06 (d, J = 3.1 Hz, 1H), 6.46 (d, J = 3.1 Hz, 1H), 5.60 (dd, J = 15.4, 6.6 Hz, 1H), 5.45 (dt, J = 13.0, 6.0 Hz, 1H), 4.50 (dd, J = 7.3, 4.5 Hz, 1H), 3.78 (s, 3H), 2.94 – 2.82 (m, 1H), 2.76 (dt, J = 14.5, 7.2 Hz, 1H), 1.96 (d, J = 8.0 Hz, 1H), 1.75 – 1.61 (m, 5H), 1.28 – 1.00 (m, 5H).

^{13}C NMR (101 MHz, $CDCl_3$) δ 166.39, 141.80, 134.56, 129.85, 129.12, 128.51, 120.91, 115.85, 113.09, 109.38, 101.17, 61.45, 40.70, 38.87, 32.97, 32.91, 26.16, 25.98.

HRMS (ESI/QTOF) m/z: $[M + H]^+$ Calcd for $C_{20}H_{26}ClN_2O^+$ 345.1734; Found 345.1736.

$[\alpha]_D^{23}$ = +42.3 (c = 0.50 in $CHCl_3$).

FTIR (neat): $\tilde{\nu}$ = 3270, 1652, 1542, 1457, 1242, 961, 751, 720 cm^{-1}.

HPLC: The enantiomeric ratio (97.5:2.5) was determined *via* HPLC analysis using a CHIRALCEL® IB column, with hexane:isopropanol = 80:20 at a flow rate 1.0 mL/min detected at 254 nm wavelength. Retention time: t_{major} = 16.7 min and t_{minor} = 23.4 min.

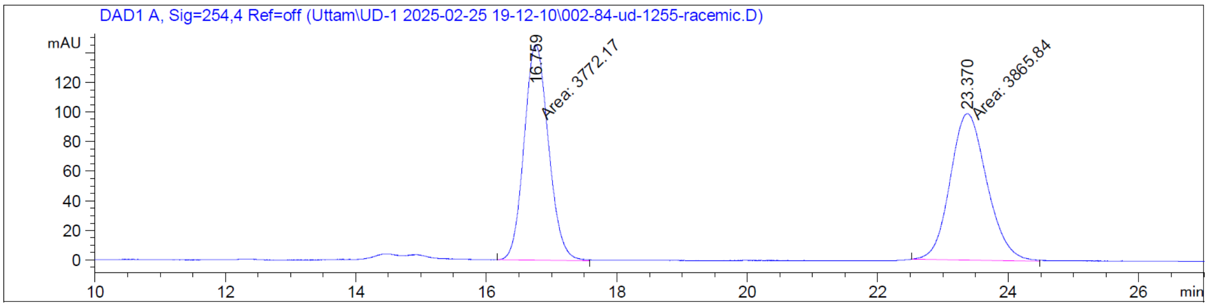

Peak #	RetTime [min]	Type	Width [min]	Area [mAU*s]	Height [mAU]	Area %
1	16.759	MM	0.4323	3772.16553	145.42279	49.3868
2	23.370	MM	0.6521	3865.84448	98.80035	50.6132

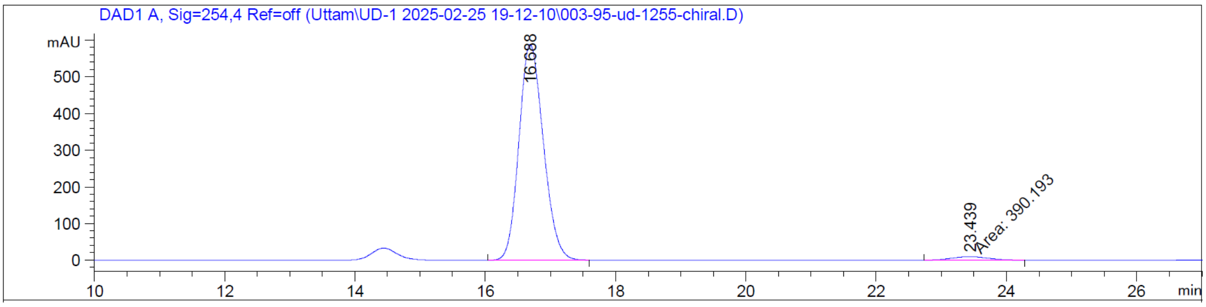

Peak #	RetTime [min]	Type	Width [min]	Area [mAU*s]	Height [mAU]	Area %
1	16.688	VB	0.3237	1.53183e4	588.54199	97.5160
2	23.439	MM	0.6446	390.19287	10.08925	2.4840

(*R*,*E*)-*N*-Benzyl-2-chloro-5-cyclohexylpent-4-enamide (9ae)

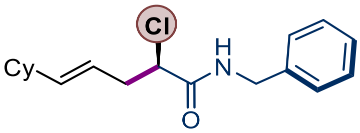

General procedure **GP1** was followed with *N*-(2-bromo-2-chloroacetyl)benzamide (**8e**) (27.7 mg, 0.10 mmol, 1.0 equiv.) and propa-1,2-dienylcyclohexane (**1a**) (24.4 mg, 0.20 mmol, 2.0 equiv.). Automated flash column chromatography (10 g SiO_2, gradient elution: hexane to 30% EtOAc in hexane) afforded the desired product (+) **9ae** as a white solid (24.5 mg, 80%).

^{1}H NMR (400 MHz, $CDCl_3$) δ 7.35 – 7.17 (m, 5H), 6.76 (t, *J* = 5.7 Hz, 1H), 5.48 (ddt, *J* = 15.4, 6.6, 1.3 Hz, 1H), 5.28 (dtd, *J* = 15.4, 6.9, 1.3 Hz, 1H), 4.47 – 4.26 (m, 3H), 2.70 (dddt, *J* = 13.3, 6.7, 4.7, 0.9 Hz, 1H), 2.61 (dt, *J* = 14.4, 7.1 Hz, 1H), 1.85 (tdt, *J* = 10.6, 6.6, 3.3 Hz, 1H), 1.65 – 1.56 (m, 5H), 1.21 – 0.92 (m, 5H).

^{13}C NMR (101 MHz, $CDCl_3$) δ 168.38, 141.71, 137.55, 128.79, 127.73, 127.71, 120.74, 60.92, 43.85, 40.63, 38.66, 32.90, 32.83, 26.16, 26.00.

HRMS (ESI/QTOF) m/z: $[M + H]^+$ Calcd for $C_{18}H_{25}ClNO^+$ 306.1625; Found 306.1631.

$[\alpha]_D^{23}$ = +12.3 (c = 0.50 in $CHCl_3$).

FTIR (neat): $\tilde{\nu}$ = 3285, 1657, 1538, 1449, 1258, 970, 697 cm^{-1}.

HPLC: The enantiomeric ratio (97.5:2.5) was determined *via* HPLC analysis using a CHIRALCEL® IB column, with hexane:isopropanol = 95:5 at a flow rate 1.0 mL/min detected at 210 nm wavelength. Retention time: t_{major} = 11.5 min and t_{minor} = 13.2 min.

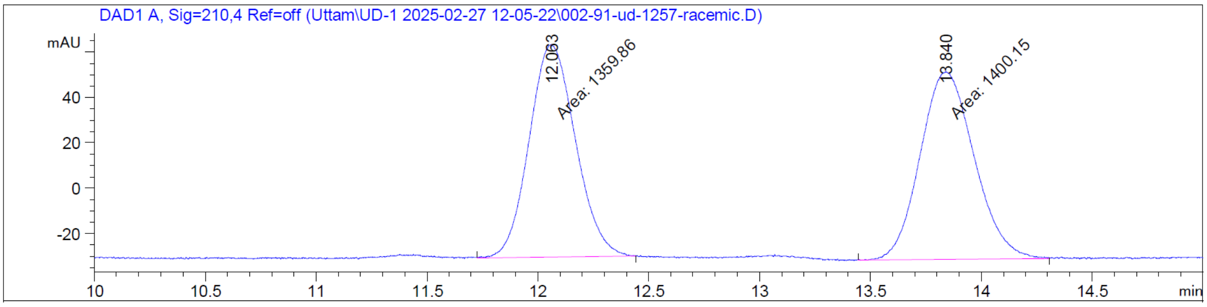

Peak #	RetTime [min]	Type	Width [min]	Area [mAU*s]	Height [mAU]	Area %
1	12.063	MM	0.2417	1359.85730	93.75597	49.2700
2	13.840	MM	0.2824	1400.15259	82.62598	50.7300

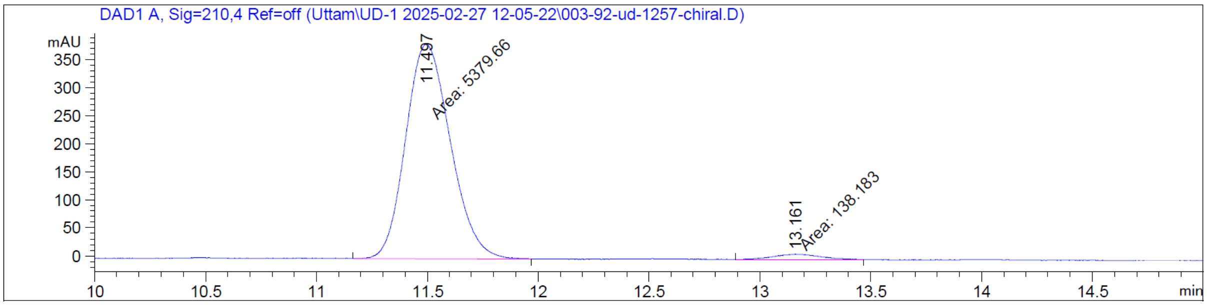

Peak #	RetTime [min]	Type	Width [min]	Area [mAU*s]	Height [mAU]	Area %
1	11.497	MM	0.2340	5379.66162	383.10684	97.4957
2	13.161	MM	0.2380	138.18297	9.67581	2.5043

(*R*,*E*)-2-Chloro-5-cyclohexyl-N-(4-phenylbutyl)pent-4-enamide (9af)

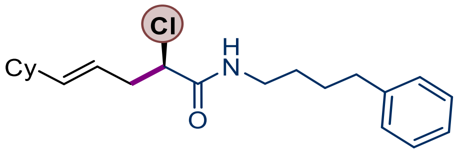

General procedure **GP1** was followed with 2-bromo-2-chloro-*N*-(4-phenylbutyl)acetamide (**8f**) (30.5 mg, 0.10 mmol, 1 equiv.) and propa-1,2-dienylcyclohexane (**1a**) (24.4 mg, 0.20 mmol, 2.0 equiv.). Automated flash column chromatography (10 g SiO_2, gradient elution: hexane to 20% EtOAc in hexane) afforded the desired product (+) **9af** as a white solid (27 mg, 77%).

^{1}H NMR (400 MHz, $CDCl_3$) δ 7.25 – 7.16 (m, 2H), 7.15 – 7.07 (m, 3H), 6.44 (t, *J* = 5.8 Hz, 1H), 5.46 (ddt, *J* = 15.4, 6.7, 1.2 Hz, 1H), 5.27 (dtd, *J* = 15.3, 6.9, 1.3 Hz, 1H), 4.25 (dd, *J* = 7.3, 4.5 Hz, 1H), 3.22 (tdd, *J* = 7.1, 5.8, 1.7 Hz, 2H), 2.68 (dddt, *J* = 14.6, 6.6, 4.5, 1.0 Hz, 1H), 2.61 – 2.50 (m, 3H), 1.85 (dtd, *J* = 11.5, 7.2, 3.1 Hz, 1H), 1.68 – 1.53 (m, 7H), 1.49 (tdd, *J* = 10.0, 6.6, 4.8 Hz, 2H), 1.23 – 0.91 (m, 5H).

^{13}C NMR (101 MHz, CD_2Cl_2) δ 168.01, 142.26, 141.35, 128.35, 128.27, 125.73, 121.10, 60.94, 40.69, 39.58, 38.62, 35.37, 32.92, 32.90, 29.02, 28.63, 26.16, 25.99.

HRMS (ESI/QTOF) m/z: $[M + H]^+$ Calcd for $C_{21}H_{31}ClNO^+$ 348.2094; Found 348.2104.

$[\alpha]_D^{23}$ = +15.3 (c = 0.33 in $CHCl_3$).

FTIR (neat): $\tilde{\nu}$ = 2921, 1654, 1557, 1448, 1211, 1179, 968, 698 cm^{-1}.

HPLC: The enantiomeric ratio (98.5:1.5) was determined *via* HPLC analysis using a CHIRALCEL® OD-H column, with hexane:isopropanol = 99.5:0.5 at a flow rate 0.5 mL/min detected at 210 nm wavelength. Retention time: t_{major} = 32.3 min and t_{minor} = 30.5 min.

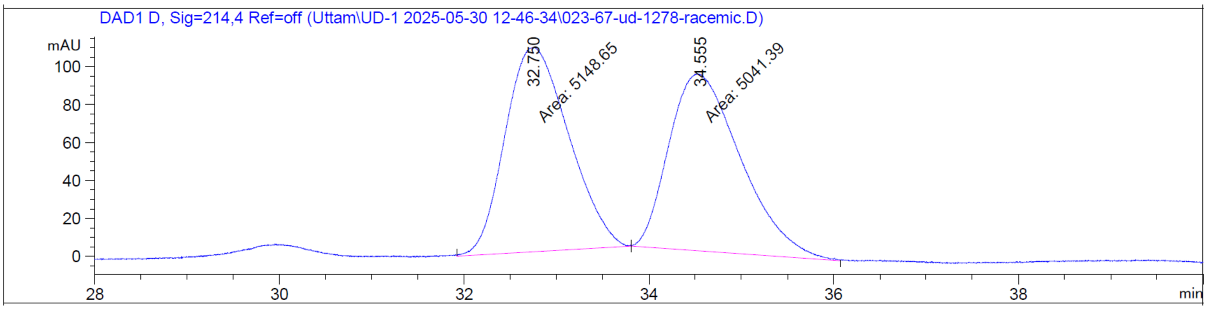

Peak #	RetTime [min]	Type	Width [min]	Area [mAU*s]	Height [mAU]	Area %
1	32.750	MM	0.7973	5148.65381	107.63165	50.5263
2	34.555	MM	0.9011	5041.39063	93.24981	49.4737

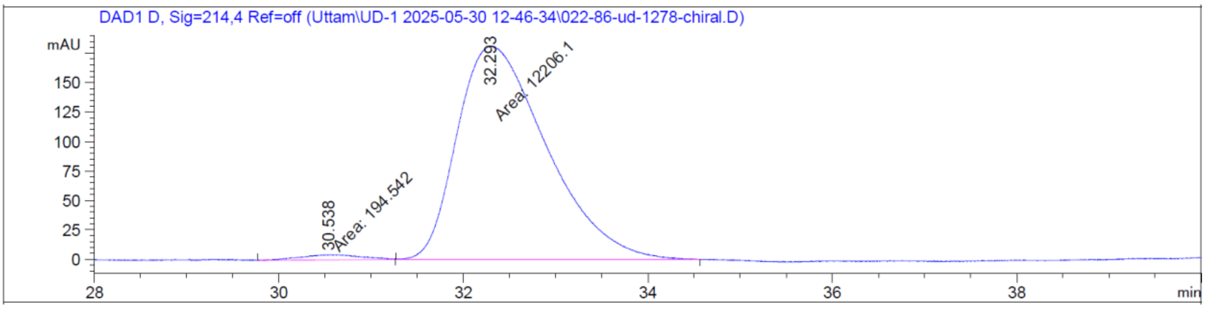

Peak #	RetTime [min]	Type	Width [min]	Area [mAU*s]	Height [mAU]	Area %
1	30.538	MM	0.7289	194.54163	4.44800	1.5688
2	32.293	MM	1.1230	1.22061e4	181.15852	98.4312

6. Synthetic Application

(*R*,*E*)-7-Fluoro-8-((4-methoxyphenyl)amino)-8-oxooct-4-en-1-yl (4*S*)-4,7,7-trimethyl-3-oxo-2-oxabicyclo[2.2.1]heptane-1-carboxylate (10)

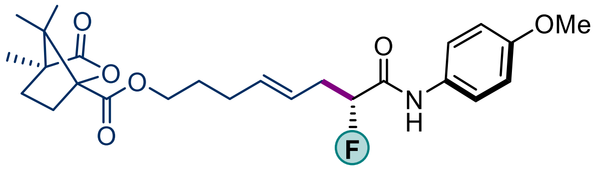

Prepared according to **GP1** with 2-bromo-2-fluoro-*N*-(4-methoxyphenyl)acetamide (**2a**) (26.2 mg, 0.10 mmol, 1.0 equiv.) and hexa-4,5-dien-1-yl (1*R*,4*S*)-4,7,7-trimethyl-3-oxo-2-oxabicyclo[2.2.1]heptane-1-carboxylate (**1p**) (55.7 mg, 0.20 mmol, 2.0 equiv.). Automated flash column chromatography (10 g SiO_2, gradient elution: hexane to 75% EtOAc in hexane) afforded the desired product (+) **10** as a white solid (30.5 mg, 66%).

^{1}H NMR (400 MHz, $CDCl_3$) δ 7.92 (d, *J* = 6.3 Hz, 1H), 7.51 – 7.38 (m, 2H), 6.90 – 6.84 (m, 2H), 5.61 (dt, *J* = 13.9, 6.6 Hz, 1H), 5.49 (dt, *J* = 15.3, 6.9 Hz, 1H), 5.00 (ddd, *J* = 49.6, 6.6, 4.0 Hz, 1H), 4.25 – 4.14 (m, 2H), 3.79 (s, 3H), 2.70 (dddt, *J* = 51.4, 28.8, 14.6, 7.7 Hz, 2H), 2.39 (ddd, *J* = 13.3, 10.7, 4.2 Hz, 1H), 2.11 (q, *J* = 7.1 Hz, 2H), 2.01 (ddd, *J* = 13.6, 9.4, 4.6 Hz, 1H), 1.90 (ddd, *J* = 13.0, 10.7, 4.6 Hz, 1H), 1.78 – 1.62 (m, 3H), 1.10 (s, 3H), 1.03 (s, 3H), 0.93 (s, 3H).

^{13}C NMR (101 MHz, $CDCl_3$) δ 178.15, 167.18 (d, $^{2}J_{C\text{–}F}$ = 18.2 Hz), 167.09, 156.86, 133.76, 129.72, 123.87 (d, $^{3}J_{C\text{–}F}$ = 2.8 Hz), 121.81, 114.24, 91.36 (d, $^{1}J_{C\text{–}F}$ = 189.0 Hz), 91.14, 64.80, 55.49, 54.77, 54.10, 35.45 (d, $^{2}J_{C\text{–}F}$ = 20.2 Hz), 30.65, 28.96, 28.77, 27.98, 16.78, 16.74, 9.71.

^{19}F{H} NMR (376 MHz, $CDCl_3$) δ -188.40.

^{19}F NMR (376 MHz, $CDCl_3$) δ -188.40 (dddd, *J* = 49.7, 29.4, 26.4, 6.5 Hz).

HRMS (APCI/QTOF) m/z: $[M + Na]^+$ Calcd for $C_{25}H_{32}FNNaO_6^+$ 484.2111; Found 484.2092.

$[\alpha]_D^{23}$ = +54.6 (c = 1.00 in $CHCl_3$).

FTIR (neat): $\tilde{\nu}$ = 3354, 1784, 1747, 1679, 1536, 1514, 1205, 1168, 1105, 1060, 830 cm^{-1}.

HPLC: The enantiomeric excess (96:4) was determined *via* HPLC analysis using a CHIRALCEL® IA column, with hexane:isopropanol = 80:20 at a flow rate 1.0 mL/min detected at 254 nm wavelength. Retention time: t_{major} = 15.5 min and t_{minor} = 20.8 min.

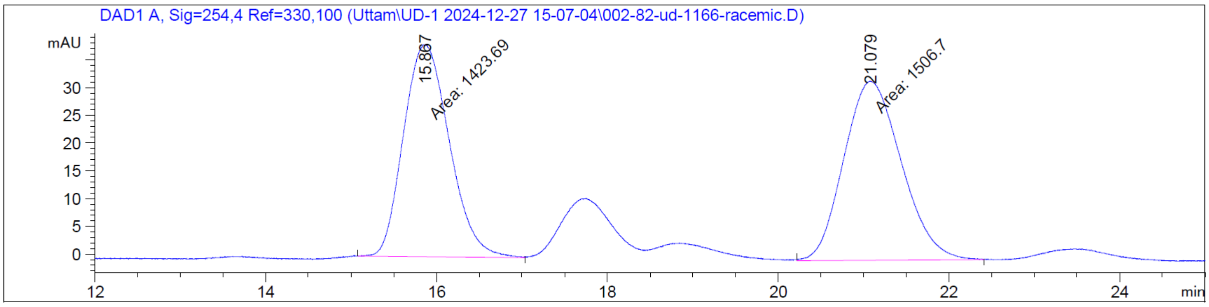

Peak #	RetTime [min]	Type	Width [min]	Area [mAU*s]	Height [mAU]	Area %
1	15.867	MM	0.6208	1423.68579	38.22381	48.5836
2	21.079	MM	0.7787	1506.69702	32.24699	51.4164

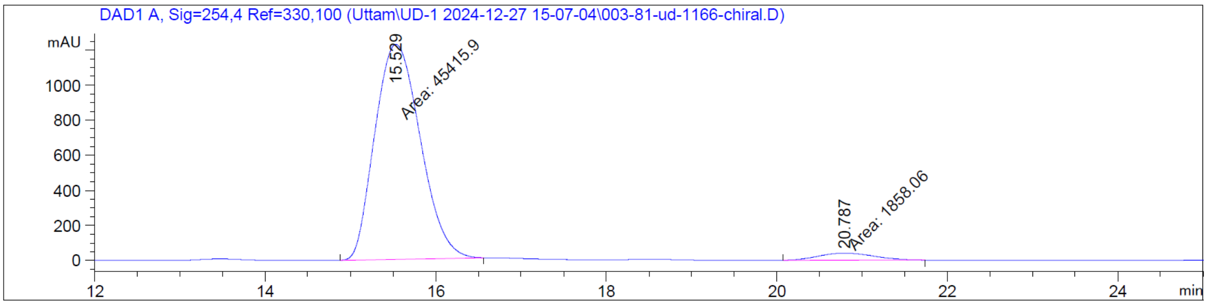

Peak #	RetTime [min]	Type	Width [min]	Area [mAU*s]	Height [mAU]	Area %
1	15.529	MM	0.6169	4.54159e4	1227.08252	96.0696
2	20.787	MM	0.7618	1858.06470	40.65225	3.9304

(*R*,*E*)-7-Fluoro-8-oxo-8-(phenylamino)oct-4-en-1-yl 4-(*N*,*N*-diisopropylsulfamoyl)benzoate (11)

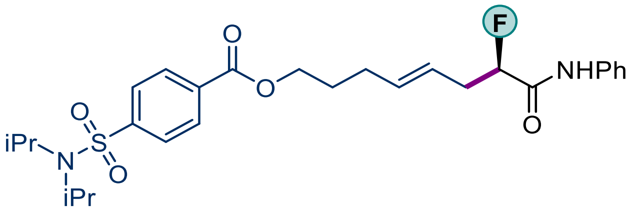

Prepared according to **GP1** with 2-bromo-2-fluoro-*N*-phenylacetamide (**2b**) (23.2 mg, 0.10 mmol, 1.0 equiv.) and hexa-4,5-dien-1-yl 4-(*N*,*N*-diisopropylsulfamoyl)benzoate (**1q**) (73.1 mg, 0.20 mmol, 2.0 equiv.). Automated flash column chromatography (10 g SiO_2, gradient elution: hexane to 30% EtOAc in hexane) afforded the desired product (+) **11** as a white solid (32 mg, 62%).

^{1}H NMR (400 MHz, $CDCl_3$) δ 8.14 – 8.09 (m, 2H), 7.99 (d, *J* = 6.6 Hz, 1H), 7.88 – 7.83 (m, 2H), 7.60 – 7.53 (m, 2H), 7.33 (dd, *J* = 8.5, 7.4 Hz, 2H), 7.19 – 7.10 (m, 1H), 5.67 (dt, *J* = 14.0, 6.7 Hz, 1H), 5.52 (dt, *J* = 15.3, 6.9 Hz, 1H), 5.03 (ddd, *J* = 49.6, 6.4, 4.0 Hz, 1H), 4.32 (td, *J* = 6.5, 4.2 Hz, 2H), 3.15 – 3.04 (m, 4H), 2.88 – 2.59 (m, 2H), 2.19 (q, *J* = 7.1 Hz, 2H), 1.84 (p, *J* = 6.9 Hz, 2H), 1.62 – 1.48 (m, 4H), 0.87 (t, *J* = 7.4 Hz, 6H).

^{13}C NMR (101 MHz, $CDCl_3$) δ 167.39 (d, $^{2}J_{C–F}$ = 18.2 Hz), 165.24, 144.23, 136.61, 134.09, 133.63, 130.17, 129.12, 126.99, 124.99, 123.66 (d, $^{3}J_{C–F}$ = 2.5 Hz), 120.01, 91.37 (d, $^{1}J_{C–F}$ = 189.3 Hz), 64.79, 49.95, 35.45 (d, $^{2}J_{C–F}$ = 20.0 Hz), 28.91, 28.05, 21.95, 11.17.

^{19}F{H} NMR (377 MHz, $CDCl_3$) δ -188.20.

^{19}F NMR (377 MHz, $CDCl_3$) δ -188.20 (dddd, *J* = 49.4, 29.9, 25.9, 6.4 Hz).

HRMS (APCI/QTOF) m/z: $[M + Na]^+$ Calcd for $C_{27}H_{35}FN_2NaO_5S^+$ 541.2148; Found 541.2167.

$[\alpha]_D^{23}$ = +35.3 (c = 0.75 in $CHCl_3$).

FTIR (neat): $\tilde{\nu}$ = 2973, 1723, 1690, 1537, 1444, 1382, 1273, 1158, 1088, 922, 753, 692 cm^{-1}.

HPLC: The enantiomeric ratio (96.5:3.5) was determined *via* HPLC analysis using a CHIRALCEL® IA column, with hexane:isopropanol = 85:15 at a flow rate 1.0 mL/min detected at 254 nm wavelength. Retention time: t_{major} = 21.1 min and t_{minor} = 25.8 min.

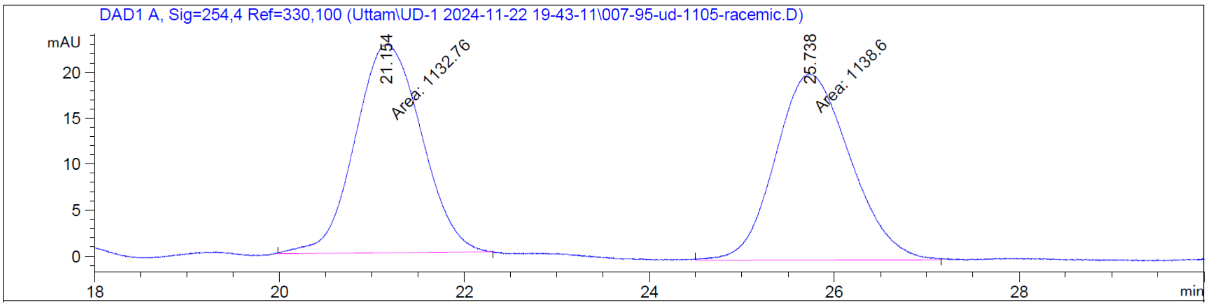

Peak #	RetTime [min]	Type	Width [min]	Area [mAU*s]	Height [mAU]	Area %
1	21.154	MM	0.8325	1132.76099	22.67822	49.8714
2	25.738	MM	0.9367	1138.60229	20.26010	50.1286

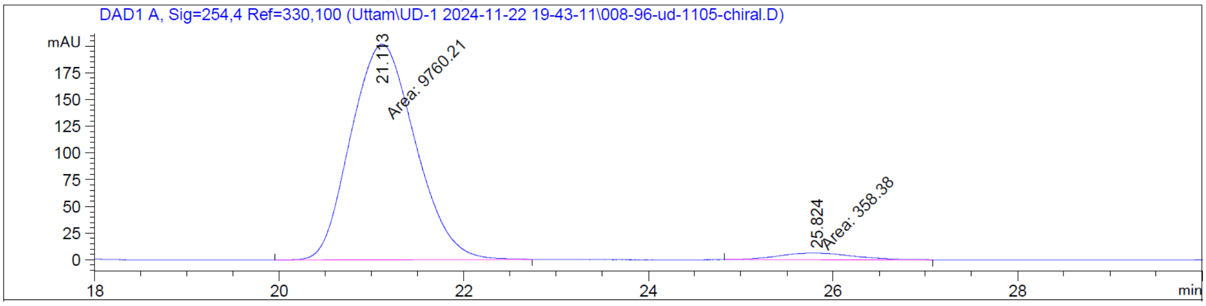

Peak #	RetTime [min]	Type	Width [min]	Area [mAU*s]	Height [mAU]	Area %
1	21.113	MM	0.8065	9760.21191	201.68895	96.4582
2	25.824	MM	0.9365	358.37985	6.37817	3.5418

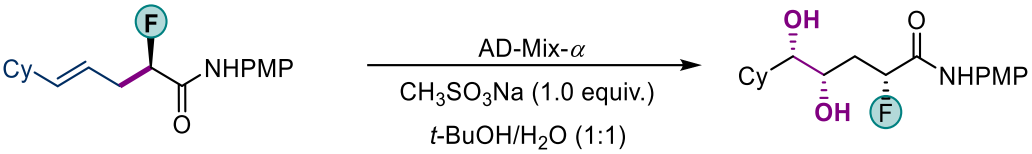

3aa **12**

(2*R*,4*S*,5*S*)-5-Cyclohexyl-2-fluoro-4,5-dihydroxy-*N*-(4-methoxyphenyl)pentanamide (12)

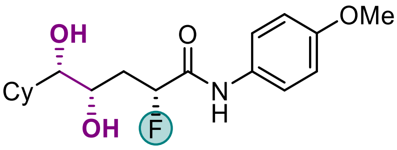

A modified literature procedure was followed.[55] A vial equipped with a magnetic stir bar was charged with *t*BuOH (0.5 mL), H_2O (0.5 mL), AD-mix-*α* (68 mg), and sodium methanesulfonate (5.0 mg, 0.050 mmol, 1.0 equiv.). The mixture was stirred until both phases became clear. Then, (*R*,*E*)-5-cyclohexyl-2-fluoro-*N*-(4-methoxyphenyl)pent-4-enamide (15 mg, 0.050 mmol, 1.0 equiv.) was added in one portion, and the heterogeneous slurry was stirred until the substrate was fully consumed. The reaction was quenched at 0 °C by adding aq. solution of Na_2SO_3, followed by warming to room temperature and stirring for 1 hour. The mixture was extracted with EtOAc. The organic layers were combined, washed with brine, dried over anhydrous Na_2SO_4, filtered, and concentrated under reduced pressure. Automated flash column chromatography (10 g SiO_2, gradient elution: hexane to 70% EtOAc in hexane) afforded the desired product (+) **12** as a white solid (14 mg, 83% yield, 7:1 d.r.)

^{1}H NMR (400 MHz, MeOD) (OH peaks are missing): δ 7.41 – 7.33 (m, 2H), 6.83 – 6.75 (m, 2H), 5.04 (dt, *J* = 48.9, 6.0 Hz, 1H), 3.86 (td, *J* = 6.6, 3.0 Hz, 1H), 3.68 (s, 3H), 3.02 (dd, *J* = 7.6, 3.0 Hz, 1H), 2.16 – 2.04 (m, 2H), 1.92 – 1.84 (m, 1H), 1.70 – 1.53 (m, 4H), 1.46 (tdt, *J* = 11.1, 6.9, 3.2 Hz, 1H), 1.22 – 0.91 (m, 6H).

^{13}C NMR (101 MHz, MeOD): Major isomer: δ 169.37 (d, $^2J_{C\text{–}F}$ = 20.2 Hz), 157.06, 130.21, 122.57, 122.44, 113.55, 89.46 (d, $^3J_{C\text{–}F}$ = 184.2 Hz), 77.31, 66.85 (d, $^3J_{C\text{–}F}$ = 4.3 Hz), 54.45, 39.74, 36.66 (d, $^2J_{C\text{–}F}$ = 20.2 Hz), 29.25, 28.57, 26.22, 25.89, 25.82.

^{19}F{H} NMR (376 MHz, MeOD) δ -189.24 (s, 0.88H, major diastereomer), -191.63 (s, 0.12 H, minor diastereomer).

^{19}F NMR (376 MHz, MeOD) δ -189.24 (0.88 H, dt, *J* = 48.7, 23.0 Hz, major diastereomer), -191.63 (0.12 H, ddd, *J* = 49.6, 40.2, 15.5 Hz, minor diastereomer).

$[\alpha]_D^{23}$ = +14.4 (c = 0.50 in $CHCl_3$).

FTIR (neat): ṽ = 3345, 2918, 1663, 1535, 1515, 1414, 1110, 1031, 818 cm^{-1}.

HRMS (APCI/QTOF) m/z: $[M + Na]^+$ Calcd for $C_{18}H_{26}FNNaO_4^+$ 362.1744; Found 362.1743. HPLC: The enantiomeric ratio (96.5:4.5) was determined *via* HPLC analysis using a CHIRALCEL® OJ-H column, with hexane:isopropanol = 85:15 at a flow rate 1.0 mL/min detected at 254 nm wavelength. Retention time: t_{major} = 11.9 min and t_{minor} = 26.1 min.

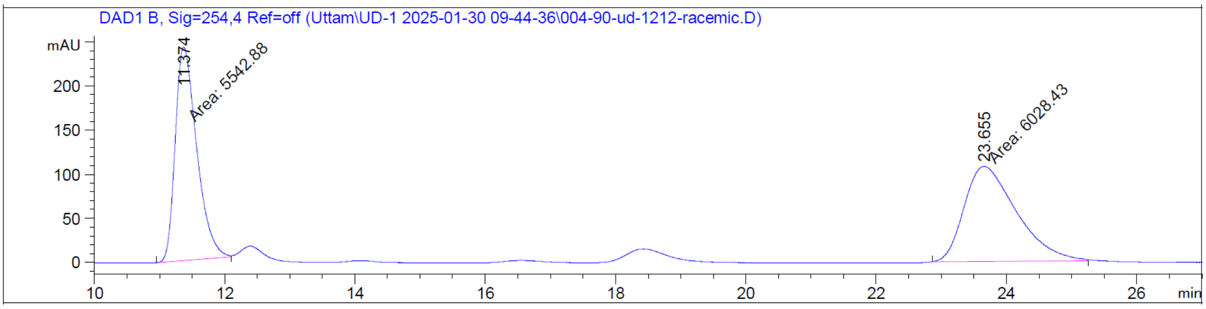

Peak #	RetTime [min]	Type	Width [min]	Area [mAU*s]	Height [mAU]	Area %
1	11.374	MM	0.3819	5542.87695	241.92616	47.9019
2	23.655	MM	0.9292	6028.43066	108.12767	52.0981

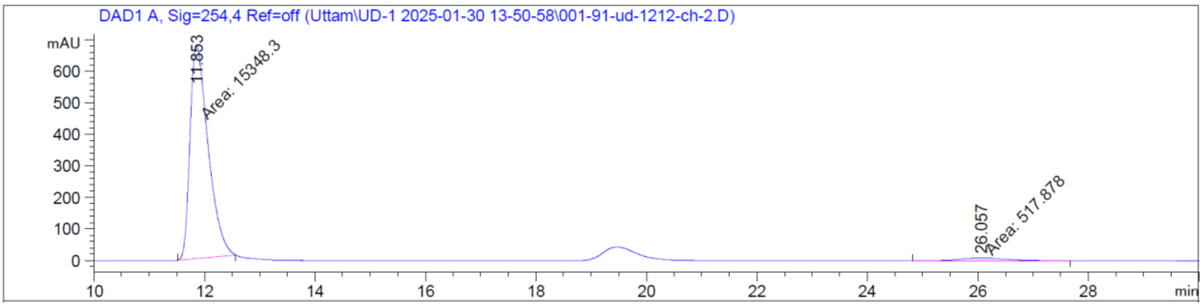

Peak #	RetTime [min]	Type	Width [min]	Area [mAU*s]	Height [mAU]	Area %
1	11.853	MM	0.3778	1.53483e4	677.10632	96.7360
2	26.057	MM	1.0300	517.87750	8.37991	3.2640

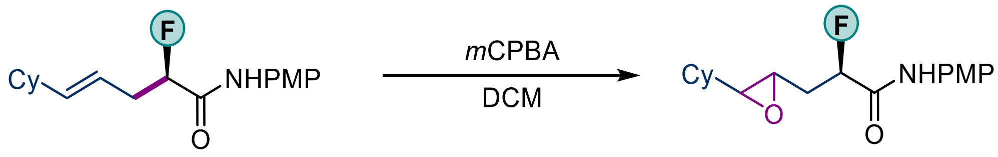

3aa **13**

(2*R*)-3-(3-Cyclohexyloxiran-2-yl)-2-fluoro-*N*-(4-methoxyphenyl)propanamide (13)

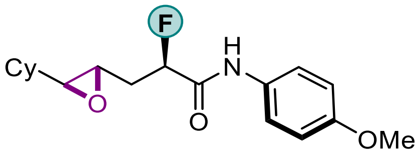

A modified literature procedure was followed.[56] A 4 mL screw-cap reaction tube with a stirring bar was charged with (*R*,*E*)-5-cyclohexyl-2-fluoro-*N*-(4-methoxyphenyl)pent-4-enamide **3aa** (15 mg, 0.050 mmol, 1.0 equiv.) and 1 mL of DCM. *m*-CPBA (0.09 mmol, 1.8 equiv.) was then added to the mixture, which was stirred at room temperature for 2 hours. Upon completion, 1 mL of saturated $NaHCO_3$ was added, and the reaction mixture was extracted with dichloromethane (3 × 5 mL). The organic layers were combined, washed with brine, dried over anhydrous Na_2SO_4, filtered, and concentrated under reduced pressure. Automated flash column chromatography (10 g SiO_2, gradient elution: hexane to 30% EtOAc in hexane) afforded the desired product (+) **13** as a white solid (13 mg, 80% yield, 2:1 d.r.).

^{1}H NMR (400 MHz, $CDCl_3$) δ 8.08 – 7.80 (m, 1H), 7.47 (dd, *J* = 9.0, 3.4 Hz, 2H), 6.95 – 6.78 (m, 2H), 5.15 (dddd, *J* = 49.5, 20.6, 7.6, 4.0 Hz, 1H), 3.80 (s, 3H), 2.96 (dtd, *J* = 9.0, 5.4, 2.2 Hz, 1H), 2.59 (dd, *J* = 6.8, 2.2 Hz, 1H), 2.40 – 2.06 (m, 2H), 1.83 – 1.60 (m, 5H), 1.25 – 1.03 (m, 6H).

^{13}C NMR (101 MHz, $CDCl_3$) (Major diastereomer): δ 166.81 (d, $^2J_{C–F}$ = 17.8 Hz), 156.94, 129.60, 121.72, 114.28, 89.88 (d, $^1J_{C–F}$ = 188.2 Hz), 63.40, 55.50, 52.87 (d, $^3J_{C–F}$ = 3.2 Hz), 39.86, 35.36 (d, $^2J_{C–F}$ = 19.4 Hz), 29.57, 28.83, 26.23, 25.60, 25.46.

^{19}F{H} NMR (376 MHz, $CDCl_3$) δ -187.17 (0.38H, minor diastereomer), -188.57 (0.62H, major diastereomer).

^{19}F NMR (376 MHz, $CDCl_3$) δ -186.82 – -187.41 (0.38H, m, minor diastereomer), -188.57 (0.62H, dddd, *J* = 49.3, 30.8, 24.8, 6.3 Hz, major diasteromer).

HRMS (APCI/QTOF) m/z: $[M + Na]^+$ Calcd for $C_{18}H_{24}FNNaO_3^+$ 344.1632; Found 344.1617.

$[\alpha]_D^{23}$ = +24.9 (c = 0.50 in $CHCl_3$).

FTIR (neat): $\tilde{\nu}$ = 2925, 1685, 1675, 1538, 1514, 1248, 1034 cm^{-1}.

HPLC (Major Diastereomer): The enantiomeric ratio (96.5:3.5) was determined *via* HPLC analysis using a CHIRALCEL® AD-H column, with hexane:isopropanol = 95:5 at a flow rate 1.0 mL/min detected at 254 nm wavelength. Retention time: t_{major} = 35.7 min and t_{minor} = 44.1 min.

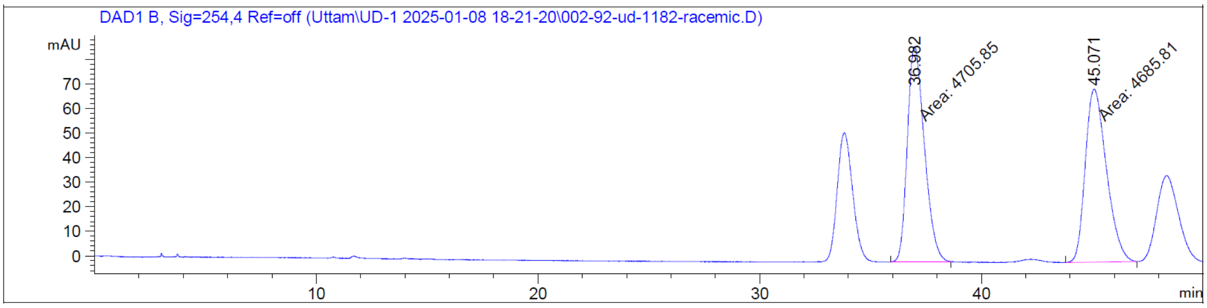

Peak #	RetTime [min]	Type	Width [min]	Area [mAU*s]	Height [mAU]	Area %
1	36.982	MM	0.8934	4705.84619	87.78703	50.1067
2	45.071	MM	1.1077	4685.81250	70.50620	49.8933

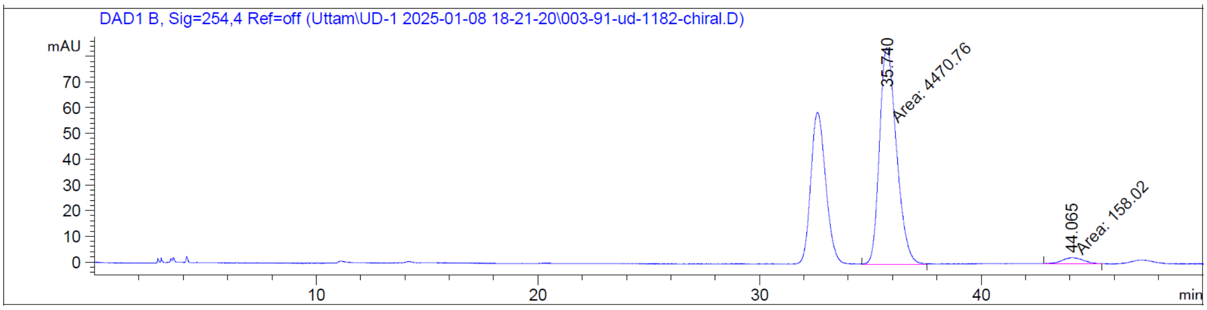

Peak #	RetTime [min]	Type	Width [min]	Area [mAU*s]	Height [mAU]	Area %
1	35.740	MM	0.8853	4470.75977	84.17042	96.5861
2	44.065	MM	1.0637	158.01979	2.47595	3.4139

HPLC (Minor Diastereomer): The enantiomeric ratio (96.5:3.5) was determined *via* HPLC analysis using a CHIRALCEL® AD-H column, with hexane:isopropanol = 95:5 at a flow rate 1.0 mL/min detected at 254 nm wavelength. Retention time: t_{major} = 32.6 min and t_{minor} = 47.2 min.

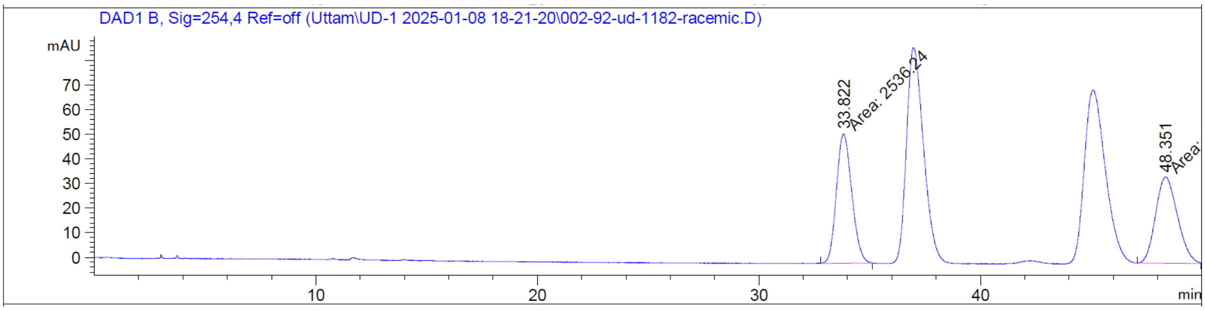

Peak #	RetTime [min]	Type	Width [min]	Area [mAU*s]	Height [mAU]	Area %
1	33.822	MM	0.8040	2536.23853	52.57640	51.2236
2	48.351	MM	1.1443	2415.06641	35.17570	48.7764

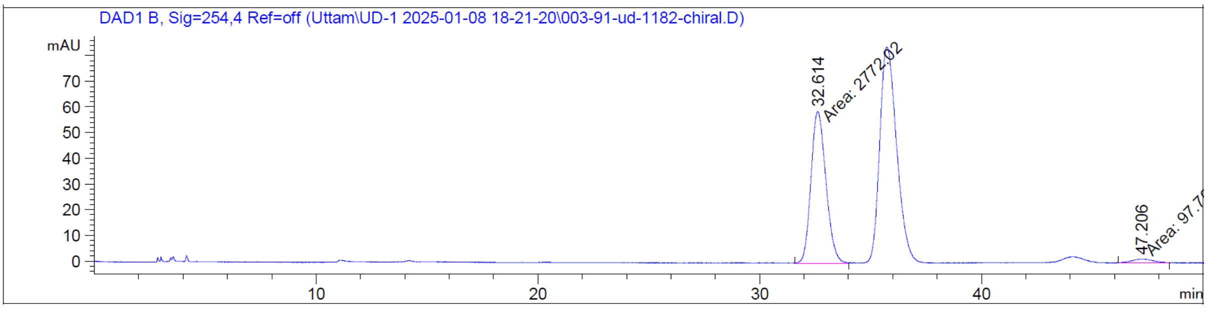

Peak #	RetTime [min]	Type	Width [min]	Area [mAU*s]	Height [mAU]	Area %
1	32.614	MM	0.7798	2772.01807	59.24984	96.5954
2	47.206	MM	1.0874	97.70206	1.49754	3.4046

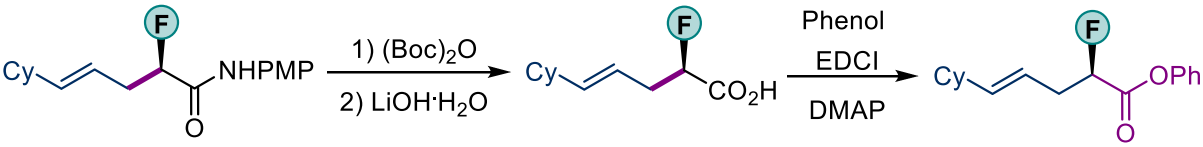

3aa: 96.5:3.5 e.r. **14** **15**

A modified literature procedure was followed.[57] To a solution of (*R*,*E*)-5-cyclohexyl-2-fluoro-*N*-(4-methoxyphenyl)pent-4-enamide **3aa** (45 mg, 0.148 mmol, 1.0 equiv.), 4-dimethylaminopyridine (0.16 mmol, 1.05 equiv), and triethylamine (0.16 mmol, 1.05 equiv.) in dichloromethane (1.0 mL), di-*tert*-butyl dicarbonate (0.30 mmol, 2.0 equiv) was added. The solution was stirred at room temperature for 12 hours. The mixture was then concentrated, and after a short filtration through silica, the crude product was used in the next step.

To the solution of the crude product in THF (0.5 mL) and H_2O (0.5 mL) was added LiOH (3.0 equiv.). The reaction mixture was allowed to stir for 2.5 h at 25 °C. The reaction was washed with DCM (3 × 3 mL), the aqueous layer was acidified with 1 N hydrochloric acid and extracted with DCM (3 × 3 mL). The combined organic layers were dried over anhydrous Na_2SO_4, filtered and concentrated under reduced pressure. The crude acid **14** was directly used for the next step without further purification.

The crude acid **14** (24 mg, 0.12 mmol, 1.0 equiv.) was added to a solution of 1-(3-dimethylaminopropyl)-3-ethylcarbodiimide hydrochloride (2.0 equiv.) and 4-dimethylaminopyridine (0.2.0 equiv.) in CH_2Cl_2 (1 mL) at 0 °C. The phenol (2.0 equiv.) was then added. The reaction mixture was allowed to be warmed to room temperature slowly and stirred overnight. The solution was diluted with CH_2Cl_2 and washed with 1 N HCl (2 ×5 mL) and brine (10 mL) sequentially. The organic layers were combined, washed with brine, dried over anhydrous Na_2SO_4, filtered, and concentrated under reduced pressure. Automated flash column chromatography (10 g SiO_2, gradient elution: hexane to 10% EtOAc in hexane) afforded the desired product (–) **15** as a white solid (27.5 mg, 0.098 mmol, 66% yield, over two steps).

^{1}H NMR (400 MHz, $CDCl_3$) δ 7.40 (t, J = 7.9 Hz, 2H), 7.30 – 7.24 (m, 1H), 7.10 (dd, J = 7.7, 1.6 Hz, 2H), 5.64 (dd, J = 15.5, 6.6 Hz, 1H), 5.55 – 5.43 (m, 1H), 5.16 (ddd, J = 48.6, 6.2, 4.9 Hz, 1H), 2.75 (tdd, J = 21.3, 14.9, 8.4 Hz, 2H), 1.98 (dtt, J = 11.2, 7.7, 3.3 Hz, 1H), 1.77 – 1.59 (m, 5H), 1.20 – 0.78 (m, 5H).

^{13}C NMR (101 MHz, $CDCl_3$) δ 167.92 (d, $^2J_{C-F}$ = 24.6 Hz), 150.01, 142.01, 129.57, 126.31, 121.28, 119.19 (d, $^3J_{C-F}$ = 3.7 Hz), 88.60 (d, $^1J_{C-F}$ = 186.7 Hz), 40.74, 35.86 (d, $^2J_{C-F}$ = 20.9 Hz), 32.87, 26.13, 25.99.

^{19}F{H} NMR (376 MHz, $CDCl_3$) δ -191.17.

^{19}F NMR (376 MHz, $CDCl_3$) δ -190.74 – -191.87 (m).

HRMS (ESI/QTOF) m/z: $[M + Na]^+$ Calcd for $C_{17}H_{21}FNaO_2^+$ 299.1423; Found 299.1419.

$[\alpha]_D^{23}$ = -6.5 (c = 1.00 in $CHCl_3$).

FTIR (neat): $\tilde{\nu}$ = 2975, 1785, 1592, 1492, 1192, 1117, 743 cm^{-1}.

HPLC: The enantiomeric ratio (95.5:4.5) was determined *via* HPLC analysis using a CHIRALCEL® OD-H column, with hexane:isopropanol = 99:1 at a flow rate 1.0 mL/min detected at 214 nm wavelength. Retention time: t_{major} = 5.3 min and t_{minor} = 5.0 min.

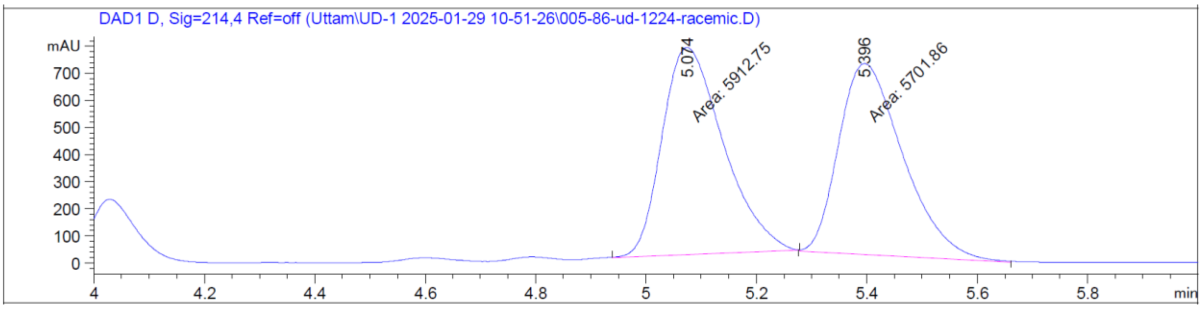

Peak #	RetTime [min]	Type	Width [min]	Area [mAU*s]	Height [mAU]	Area %
1	5.074	MM	0.1289	5912.75195	764.73615	50.9079
2	5.396	MM	0.1349	5701.85791	704.42126	49.0921

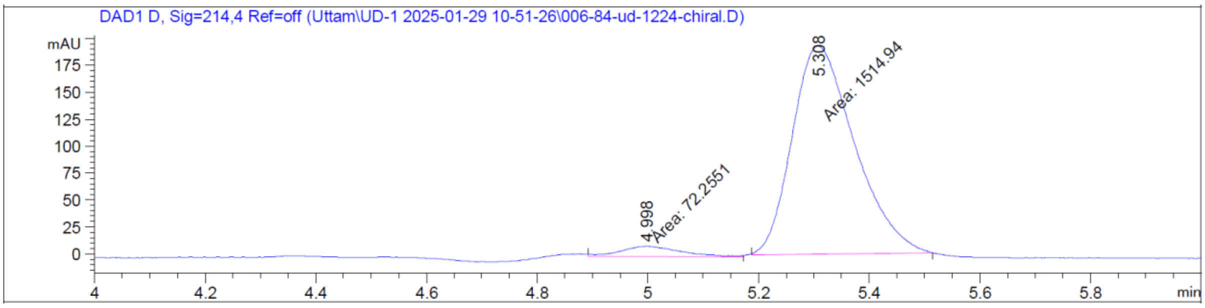

Peak #	RetTime [min]	Type	Width [min]	Area [mAU*s]	Height [mAU]	Area %
1	4.998	MM	0.1263	72.25513	9.53203	4.5524
2	5.308	MM	0.1306	1514.93665	193.39148	95.4476

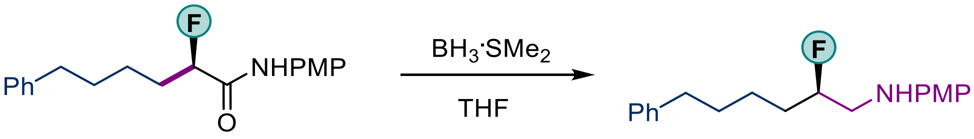

5aa: 95.5:4.5 e.r. **16**

A modified literature procedure was followed.[12] Borane-methyl sulfide complex (9.03 µL, 95.1 µmol, 2.0 equiv.) was added dropwise to a solution of (*R*)-2-fluoro-*N*-(4-methoxyphenyl)-6-phenylhexanamide **5aa** (15 mg, 47.6 µmol, 1.0 equiv.) in THF (1.0 mL) at 0 ºC in a 10-mL Schlenk tube. The reaction mixture was allowed to warm to room temperature and heated to reflux for overnight. Then the reaction was quenched with NaOH aqueous solution (1 M, 1.0 mL). The resulting mixture was extracted with EtOAc (3 x 10 mL). The combined organic layer was dried over Na_2SO_4, filtered, and concentrated under reduced pressure. Automated flash column chromatography (10 g SiO_2, gradient elution: hexane to 10% EtOAc in hexane) afforded the desired product (+) **16** as a white solid (12.8 mg, 89% yield).

^{1}H NMR (400 MHz, $CDCl_3$) δ 7.35 – 7.24 (m, 2H), 7.22 – 7.12 (m, 3H), 6.84 – 6.74 (m, 2H), 6.67 – 6.54 (m, 2H), 4.68 (dtt, *J* = 49.8, 7.7, 3.7 Hz, 1H), 3.75 (s, 3H), 3.36 – 3.08 (m, 2H), 2.63 (t, *J* = 7.6 Hz, 2H), 1.82 – 1.41 (m, 7H).

^{13}C NMR (101 MHz, $CDCl_3$) δ 152.52, 142.30, 141.94, 128.40, 128.33, 125.77, 114.95, 114.60, 92.93 (d, $^{1}J_{C–F}$ = 168.6 Hz), 55.81, 49.21 (d, $^{2}J_{C–F}$ = 21.4 Hz), 35.78, 32.75 (d, $^{2}J_{C–F}$ = 20.3 Hz), 31.26, 24.72 (d, $^{3}J_{C–F}$ = 4.7 Hz).

$^{19}F\{H\}$ NMR (377 MHz, $CDCl_3$) δ -186.53.

^{19}F NMR (377 MHz, $CDCl_3$) δ -186.15 – -186.90 (m).

HRMS (ESI/QTOF) m/z: $[M + H]^+$ Calcd for $C_{19}H_{25}FNO^+$ 302.1920; Found 302.1920.

$[\alpha]_D^{23}$ = +1.5 (c = 0.50 in $CHCl_3$).

FTIR (neat): $\tilde{\nu}$ = 2924, 1685, 1606, 1588, 1512, 1235, 1143, 1090, 820 cm^{-1}.

HPLC: The enantiomeric ratio (95.5:4.5) was determined *via* HPLC analysis using a CHIRALCEL® IA column, with hexane:isopropanol = 95:5 at a flow rate 1.0 mL/min detected at 254 nm wavelength. Retention time: t_{major} = 11.4 min and t_{minor} = 10.6 min.

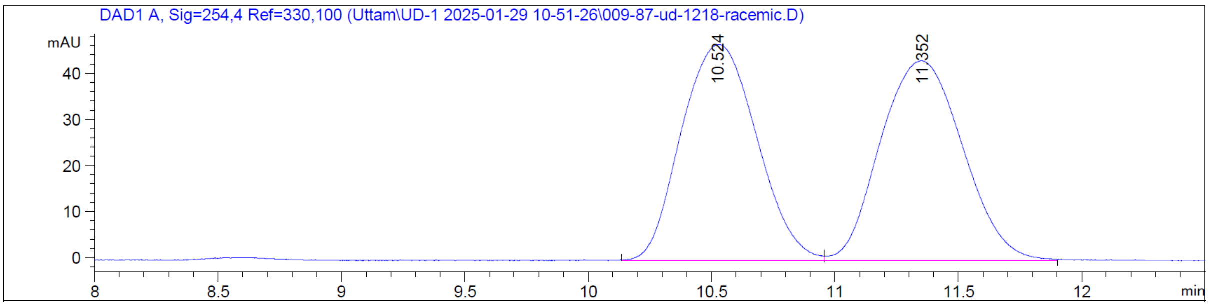

Peak #	RetTime [min]	Type	Width [min]	Area [mAU*s]	Height [mAU]	Area %
1	10.524	BV	0.2496	990.88000	46.78089	49.7904
2	11.352	VV	0.2714	999.22235	43.27758	50.2096

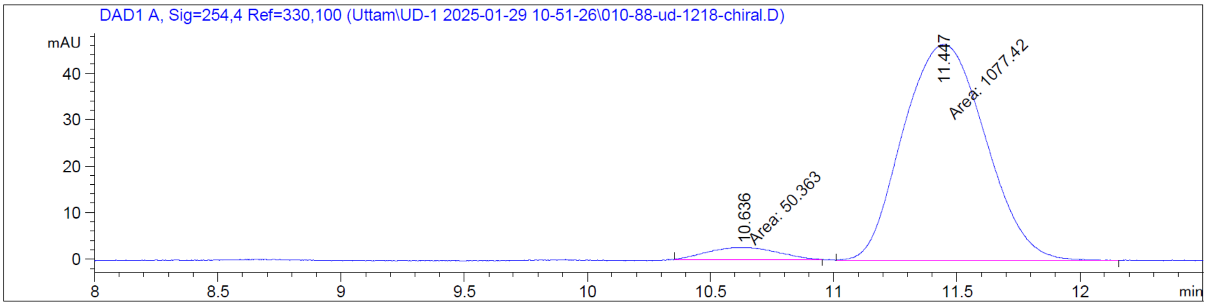

Peak #	RetTime [min]	Type	Width [min]	Area [mAU*s]	Height [mAU]	Area %
1	10.636	MM	0.3263	50.36304	2.57250	4.4657
2	11.447	MM	0.3863	1077.41711	46.48356	95.5343

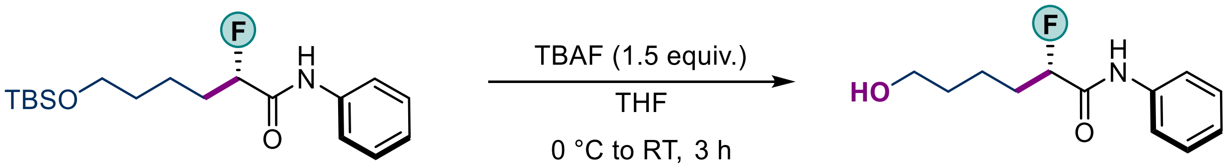

5cb **5cb-1**

To a stirred solution of (*S*)-6-((*tert*-butyldimethylsilyl)oxy)-2-fluoro-*N*-phenylhexanamide (**5cb**) (80 mg, 0.24 mmol, 1 equiv.) in THF (5 mL) at 0 °C, was added tetrabutylammonium fluoride (1 M, 0.35 mL, 1.5 equiv.) dropwise under nitrogen. After addition was complete, the reaction mixture was allowed to stir at RT for 2 h and was monitored by TLC. The mixture was then diluted with EtOAc (3 mL) and H_2O (3 mL). The resulting mixture was extracted with EtOAc (3 x 10 mL). The combined organic layer was dried over Na_2SO_4, filtered, and concentrated under reduced pressure and directly used for next step without further purification.

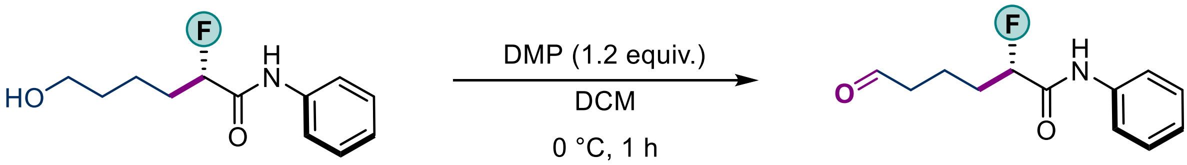

5cb-1 **5cb-2**

To a solution of (*S*)-2-fluoro-6-hydroxy-*N*-phenylhexanamide (**5cb-1**) (1 equiv.) in CH_2Cl_2 at 0 °C was added dess-martin periodinane (120 mg, 0.28 mmol, 1.2 equiv.). The reaction mixture was stirred for 1 h at 0 °C and was monitored by TLC. After complete conversion, the reaction mixture was quenched by the addition of saturated aqueous $Na_2S_2O_3$. After washing with saturated aqueous $Na_2S_2O_3$, then saturated aqueous $NaHCO_3$, and brine, the organic layer was dried over Na_2SO_4 and concentrated under reduced pressure. Automated flash column chromatography (10 g SiO_2, gradient elution: hexane to 40% EtOAc in hexane) afforded the desired product (-) **5cb-2** as a colourless oil (48 mg, 90% yield, over two steps).

^{1}H NMR (400 MHz, $CDCl_3$) δ 9.59 (t, *J* = 1.3 Hz, 1H), 7.90 – 7.78 (m, 1H), 7.41 – 7.33 (m, 2H), 7.20 – 7.11 (m, 2H), 6.99 – 6.92 (m, 1H), 4.83 (ddd, *J* = 49.7, 7.4, 3.8 Hz, 1H), 2.34 (td, *J* = 7.2, 1.3 Hz, 2H), 2.03 – 1.74 (m, 2H), 1.66 (dq, *J* = 8.0, 7.0 Hz, 2H).

^{13}C NMR (101 MHz, CDCl3) δ 201.36, 167.55 (d, $^{2}J_{C–F}$ = 17.8 Hz), 136.54, 129.17, 125.09, 120.01, 91.75 (d, $^{1}J_{C–F}$ = 187.8 Hz), 43.21, 31.64 (d, $^{2}J_{C–F}$ = 20.2 Hz), 17.12 (d, $^{3}J_{C–F}$ = 2.8 Hz).

^{19}F{H} NMR (376 MHz, $CDCl_3$) δ -188.33.

^{19}F NMR (376 MHz, $CDCl_3$) δ -188.07 – -188.54 (m).

HRMS (ESI/QTOF) m/z: $[M + Na]^+$ Calcd for $C_{12}H_{14}FNNaO_2^+$ 246.0906; Found 246.0913.

$[\alpha]_D^{23}$ = -14.4 (c = 0.33 in $CHCl_3$).

FTIR (neat): $\tilde{\nu}$ = 2923, 1718, 1674, 1600, 1538, 1445, 1081, 755, 693 cm^{-1}.

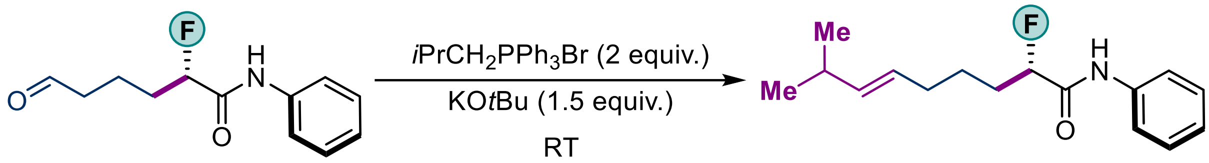

5cb-1 **17**

Potassium *tert*-butoxide (19.8 mg, 0.18 mmol, 1.5 equiv.) was added in a solution of isobutyltriphenylphosphonium bromide (94.1 mg, 0.24 mmol, 2 equiv.) in anhydrous THF (0.2 mL) under nitrogen. The reaction mixture was heated at 50 °C for 30 minutes, and then it was cooled down to RT. A solution of (*S*)-2-fluoro-6-oxo-*N*-phenylhexanamide (22.3 mg, 0.12 mmol, 1 equiv.) in anhydrous THF was added dropwise. The resulting mixture was kept stirring for 30 minutes to 1 h and the reaction was monitored by TLC. The reaction was quenched by addition of sat. aq NH_4Cl. The resulting mixture was extracted with EtOAc (3 x 10 mL). The combined organic layer was dried over Na_2SO_4, filtered, and concentrated under reduced pressure. Automated flash column chromatography (10 g SiO_2, gradient elution: hexane to 10% EtOAc in hexane) afforded the desired product (-) **17** as a colourless oil (25 mg, 80% yield). 5% defluorinated side product was observed.

^{1}H NMR (400 MHz, $CDCl_3$) δ 7.90 (s, 1H), 7.53 – 7.42 (m, 2H), 7.32 – 7.21 (m, 2H), 7.15 – 7.01 (m, 1H), 5.21 – 5.07 (m, 2H), 4.95 (ddd, *J* = 50.0, 7.6, 3.7 Hz, 1H), 2.50 (ddt, *J* = 13.4, 8.3, 6.7 Hz, 1H), 2.05 (qd, *J* = 6.6, 2.7 Hz, 2H), 2.00 – 1.77 (m, 2H), 1.56 – 1.44 (m, 2H), 0.87 (d, *J* = 6.6 Hz, 6H).

^{13}C NMR (101 MHz, $CDCl_3$) δ 168.03 (d, $^{2}J_{C–F}$ = 18.2 Hz), 138.52, 136.69, 129.14, 126.14, 124.95, 119.97, 92.16 (d, $^{1}J_{C–F}$ = 187.0 Hz), 32.00 (d, $^{2}J_{C–F}$ = 20.2 Hz), 26.76, 26.51, 24.64 (d, $^{3}J_{C–F}$ = 2.8 Hz), 23.17.

^{19}F{H} NMR (376 MHz, $CDCl_3$) δ -187.88.

^{19}F NMR (376 MHz, $CDCl_3$) δ -187.88 (dddd, *J* = 50.0, 30.0, 26.2, 6.9 Hz).

HRMS (ESI/QTOF) m/z: $[M + H]^+$ Calcd for $C_{16}H_{23}FNO^+$ 264.1764; Found 264.1763.

$[\alpha]_D^{23}$ = -18.8 (c = 0.40 in $CHCl_3$).

FTIR (neat): $\tilde{\nu}$ = 2955, 1674, 1601, 1535, 1444, 1211, 753, 692 cm^{-1}.

HPLC: The enantiomeric ratio (95:5) was determined *via* HPLC analysis using a CHIRALCEL® IB column, with hexane:isopropanol = 99:1 at a flow rate 1.0 mL/min detected at 254 nm wavelength. Retention time: t_{major} = 12.8 min and t_{minor} = 27.3 min.

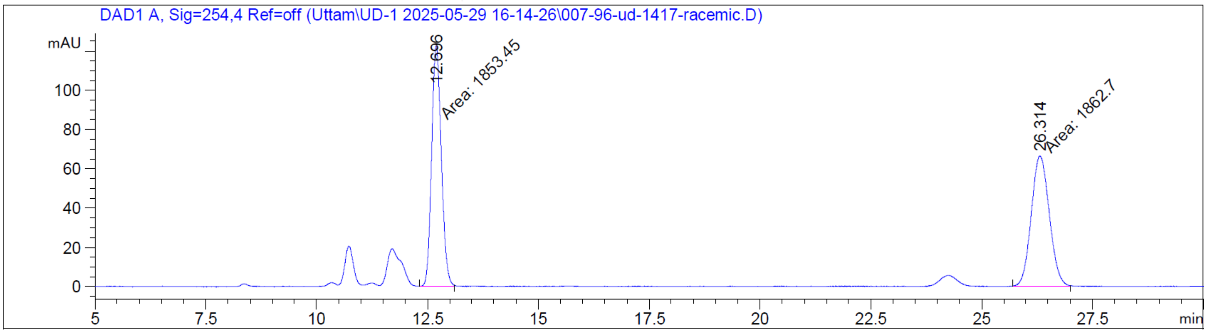

Peak #	RetTime [min]	Type	Width [min]	Area [mAU*s]	Height [mAU]	Area %
1	12.696	MM	0.2526	1853.44885	122.29034	49.8755
2	26.314	MM	0.4692	1862.70410	66.17146	50.1245

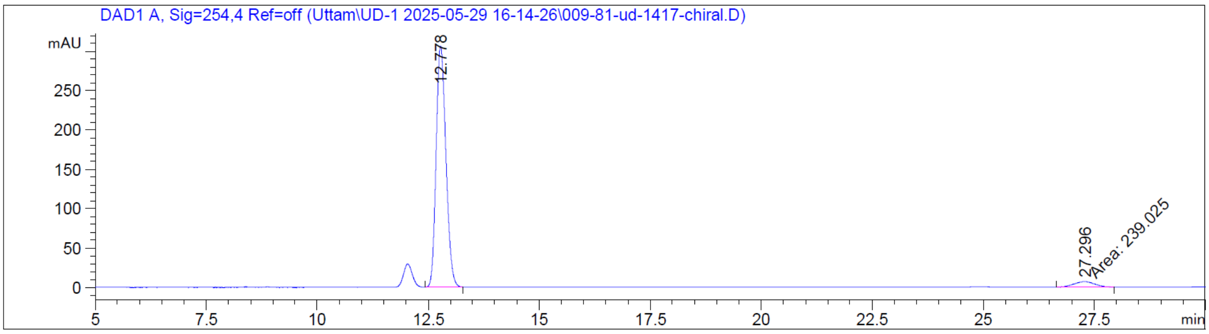

Peak #	RetTime [min]	Type	Width [min]	Area [mAU*s]	Height [mAU]	Area %
1	12.778	VV	0.2152	4692.86816	306.41354	95.1535
2	27.296	MM	0.5600	239.02472	7.11344	4.8465

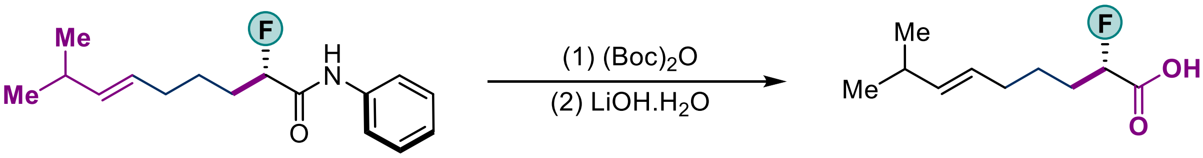

17 **18**

To a solution of (*S,E*)-2-fluoro-8-methyl-*N*-phenylnon-6-enamide **17** (15 mg, 0.057 mmol, 1.0 equiv.), 4-dimethylaminopyridine (0.06 mmol, 1.05 equiv), and triethylamine (0.06 mmol, 1.05 equiv.) in dichloromethane (0.5 mL), di-*tert*-butyl dicarbonate (0.11 mmol, 2.0 equiv) was added. The solution was stirred at room temperature for 12 hours. The mixture was then concentrated, and after a short filtration through silica, the crude product was used in the next step.

To the solution of the crude product in THF (0.5 mL) and H_2O (0.5 mL) was added $LiOH{\cdot}H_2O$ (3.0 equiv.). The reaction mixture was allowed to stir for 2.5 h at 25 °C. The reaction was washed with DCM (3 × 3 mL), the aqueous layer was acidified with 1 N hydrochloric acid and extracted with DCM (3 × 3 mL). The combined organic layers were dried over anhydrous Na_2SO_4, filtered and concentrated under reduced pressure. Automated flash column chromatography (10 g SiO_2, gradient elution: hexane to 80% EtOAc in hexane) afforded the desired product (-) **18** as a colourless oil (10.5 mg, 71% yield, over two steps).

^{1}H NMR (400 MHz, $CDCl_3$) δ 5.23 – 5.07 (m, 2H), 4.90 (d, *J* = 48.5 Hz, 1H), 2.50 (dp, *J* = 8.6, 6.7 Hz, 1H), 2.05 (q, *J* = 7.0 Hz, 2H), 1.97 – 1.78 (m, 2H), 1.56 – 1.44 (m, 2H), 0.87 (d, *J* = 6.7 Hz, 6H).

^{13}C NMR (101 MHz, $CDCl_3$) δ 174.66 (d, $^2J_{C–F}$ = 22.1 Hz), 138.70, 125.88, 88.44 (d, $^1J_{C–F}$ = 183.9 Hz), 31.71 (d, $^2J_{C–F}$ = 20.6 Hz), 26.56, 26.52, 24.52 (d, $^3J_{C–F}$ = 2.3 Hz), 23.15.

^{19}F{H} NMR (376 MHz, $CDCl_3$) δ -191.84.

^{19}F NMR (376 MHz, $CDCl_3$) δ -191.47 – -192.94 (m)

HRMS (ESI/QTOF) m/z: $[M]^-$ Calcd for $C_{10}H_{16}FO_2^-$ 187.1134; Found 187.1133.

$[\alpha]_D^{23}$ = -3.2 (c = 0.33 in $CHCl_3$).

FTIR (neat): $\tilde{\nu}$ = 2956, 1733, 1459, 1232, 1090, 858, 737 cm^{-1}.

For HPLC analysis, **18** was converted back into **17**: A solution of oxalyl chloride (1.1 eq.) in dry DCM (0.5 M) was added slowly to a stirred solution of **18** in DCM (0.5 M) at 0 °C, then 2 drops of DMF were added. Gas evolution was observed and the reaction was allowed to stir at room temperature for 2 additional hours. The reaction mixture was concentrated under reduced pressuree and re-dissolved in THF. Then, aniline (1.1 eq.) and triethylamine (1.2 eq.) were added. After reaction completion (as determined by TLC), the reaction was quenched with saturated NH_4Cl solution and extracted with EtOAc three times. Organic layers were combined and concentrated under reduced pressure. The resulting residue was purified by preparative TLC to yield compound **17**. HPLC analysis was performed of **17**.

HPLC: The enantiomeric ratio (95:5) was determined *via* HPLC analysis using a CHIRALCEL® IB column, with hexane:isopropanol = 99:1 at a flow rate 1.0 mL/min detected at 254 nm wavelength. Retention time: t_{major} = 11.7 min and t_{minor} = 27.1 min.

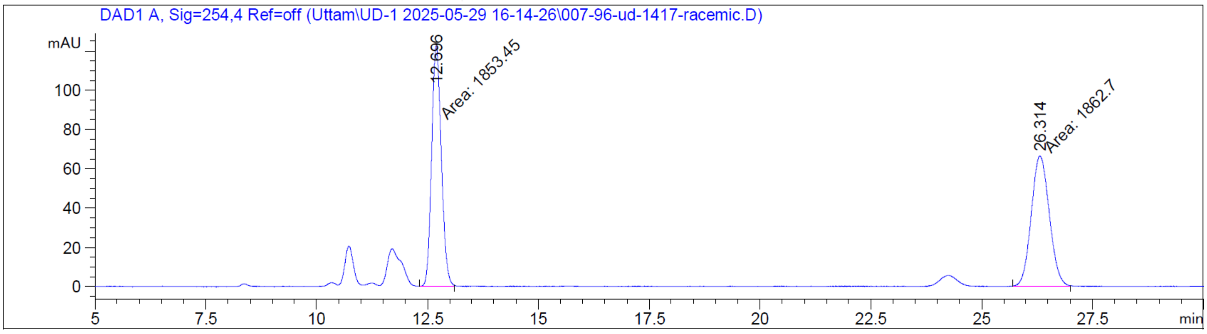

Peak #	RetTime [min]	Type	Width [min]	Area [mAU*s]	Height [mAU]	Area %
1	12.696	MM	0.2526	1853.44885	122.29034	49.8755
2	26.314	MM	0.4692	1862.70410	66.17146	50.1245

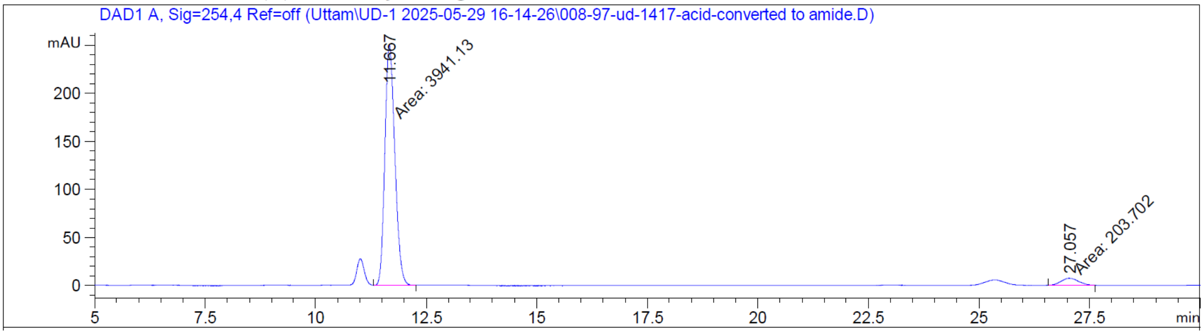

Peak #	RetTime [min]	Type	Width [min]	Area [mAU*s]	Height [mAU]	Area %
1	11.667	MM	0.2630	3941.12842	249.76468	95.0854
2	27.057	MM	0.4569	203.70157	7.43116	4.9146

Figure S2. Unsuccessful Substrates

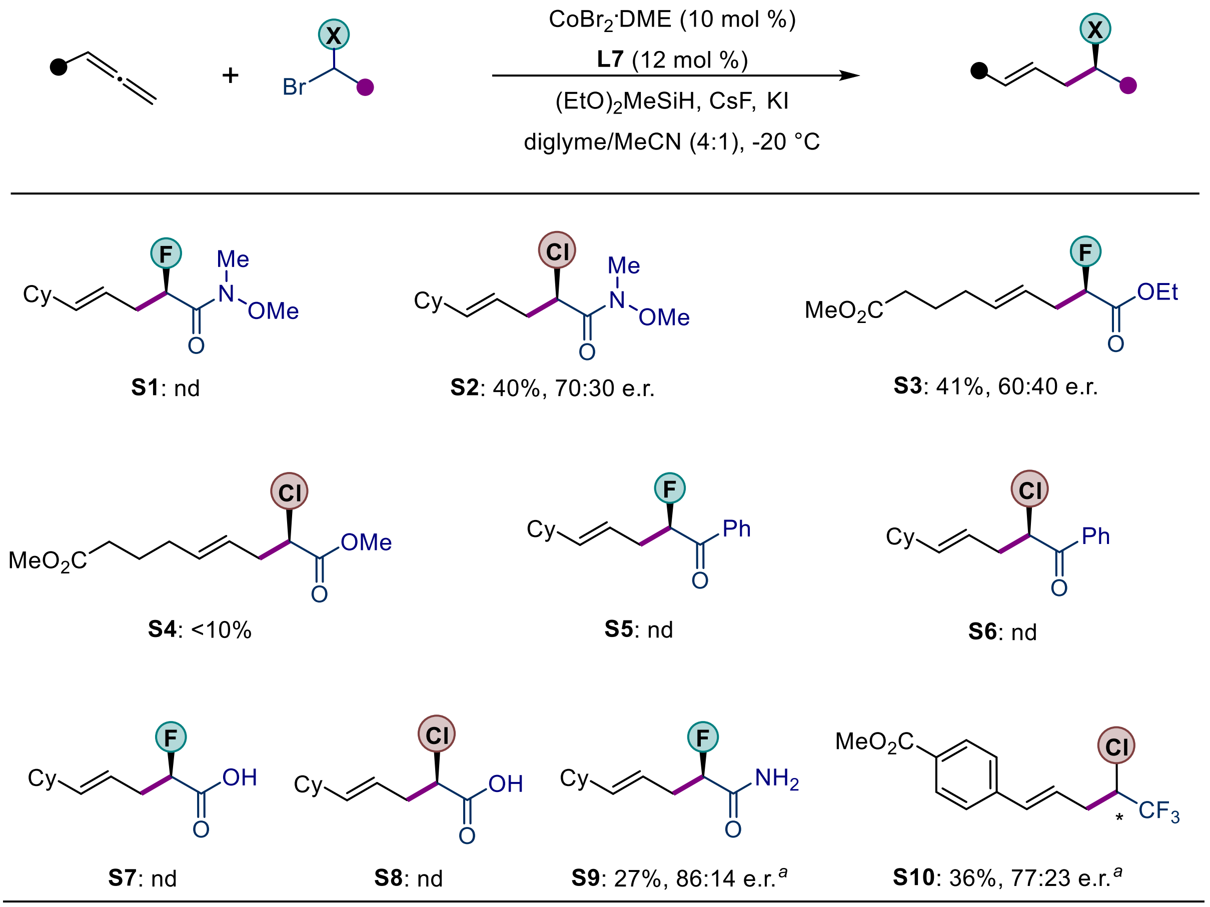

Conditions: $CoBr_2$·DME (10 mol %), **L7** (12 mol %), allene (0.20 mmol), electrophile (0.10 mmol), $(EtO)_2MeSiH$ (0.25 mmol), CsF (0.25 mmol), KI (0.10 mmol) and diglyme/MeCN (0.8:0.2 mL) at -20 °C for 40 h. [a] 0 °C for 20 h.

(*R*,*E*)-2-Chloro-5-cyclohexyl-*N*-methoxy-*N*-methylpent-4-enamide (S2)

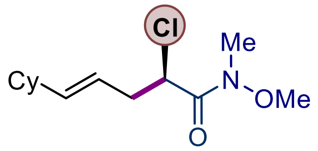

Prepared according to **GP1** with 2-bromo-2-chloro-*N*-methoxy-*N*-methylacetamide (21.6 mg, 0.10 mmol, 1 equiv.) and propa-1,2-dienylcyclohexane (29.1 µL, 0.2 mmol, 2 equiv.). Automated flash column chromatography (10 g SiO_2, gradient elution: hexane to 50% EtOAc in hexane) afforded the desired product (+) **S2** as a colourless oil (10.5 mg, 40%).

^{1}H NMR (400 MHz, $CDCl_3$) δ 5.56 (dd, *J* = 15.5, 6.7 Hz, 1H), 5.42 – 5.20 (m, 1H), 4.73 (bs, 1H), 3.78 (s, 3H), 3.25 (s, 3H), 2.67 (ddt, *J* = 64.7, 14.1, 7.2 Hz, 2H), 1.94 (d, *J* = 7.7 Hz, 1H), 1.82 – 1.59 (m, 5H), 1.26 (dtdd, *J* = 14.2, 10.8, 7.2, 3.2 Hz, 3H), 1.10 – 0.98 (m, 2H).

^{13}C NMR (101 MHz, $CDCl_3$) δ 169.60, 141.43, 121.47, 61.80, 52.28, 40.68, 37.55, 32.88, 32.86, 26.14, 25.98.

HRMS (ESI/QTOF) m/z: $[M + Na]^+$ Calcd for $C_{13}H_{22}ClNNaO_2^+$ 282.1237; Found 282.1234.

$[\alpha]_D^{23}$ = +1.6 (c = 0.33 in $CHCl_3$).

FTIR (neat): $\tilde{\nu}$ = 2924, 1674, 1536, 1448, 1142, 972, 668 cm^{-1}.

HPLC: The enantiomeric ratio (70:30) was determined *via* HPLC analysis using a CHIRALCEL® OD-H column, with hexane:isopropanol = 90:10 at a flow rate 1.0 mL/min detected at 214 nm wavelength. Retention time: t_{major} = 4.3 min and t_{minor} = 5.1 min.

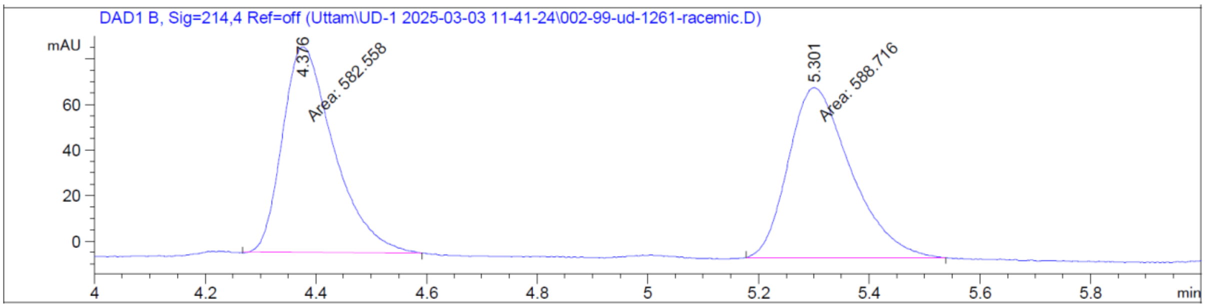

Peak #	RetTime [min]	Type	Width [min]	Area [mAU*s]	Height [mAU]	Area %
1	4.376	MM	0.1078	582.55817	90.05111	49.7371
2	5.301	MM	0.1313	588.71594	74.70275	50.2629

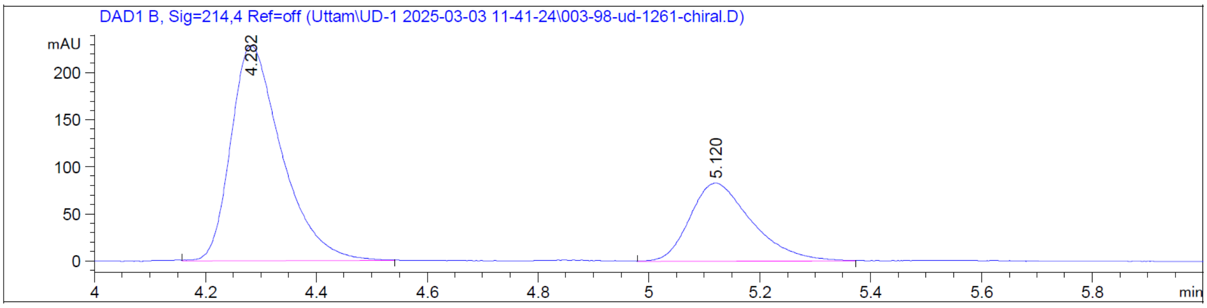

Peak #	RetTime [min]	Type	Width [min]	Area [mAU*s]	Height [mAU]	Area %
1	4.282	VV	0.0956	1466.73474	229.31219	69.6132
2	5.120	BV	0.1051	640.24426	82.93857	30.3868

Dimethyl (*R*,*E*)-2-fluoronon-4-enedioate (S3)

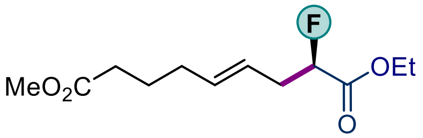

Prepared according to **GP1** with ethyl 2-bromo-2-fluoroacetate (18.5 mg, 0.10 mmol, 1 equiv.) and methyl hepta-5,6-dienoate (28.0 mg, 0.2 mmol, 2 equiv.). Automated flash column chromatography (10 g SiO_2, gradient elution: hexane to 20% EtOAc in hexane) afforded the desired product (+) **S3** as a colourless oil (10.0 mg, 41%).

^{1}H NMR (400 MHz, $CDCl_3$) δ 5.40 (dt, *J* = 13.8, 6.6 Hz, 1H), 5.29 (dt, *J* = 16.0, 6.8 Hz, 1H), 4.74 (ddd, *J* = 48.8, 6.7, 4.8 Hz, 1H), 4.17 – 4.02 (m, 2H), 3.51 (d, *J* = 1.1 Hz, 3H), 2.43 (ddq, *J* = 28.4, 14.9, 6.8 Hz, 2H), 2.14 (t, *J* = 7.5 Hz, 2H), 1.90 (q, *J* = 7.1 Hz, 2H), 1.54 (p, *J* = 7.4 Hz, 2H), 1.15 (td, *J* = 7.1, 1.0 Hz, 3H).

^{13}C NMR (101 MHz, $CDCl_3$) δ 174.01, 169.42 (d, $^2J_{C–F}$ = 23.6 Hz), 134.26, 123.36 (d, $^3J_{C–F}$ = 3.6 Hz), 88.57 (d, $^1J_{C–F}$ = 185.6 Hz), 61.46, 51.50, 35.65 (d, $^2J_{C–F}$ = 21.4 Hz), 33.28, 31.83, 24.32, 14.20.

^{19}F{H} NMR (377 MHz, $CDCl_3$) δ -191.51.

^{19}F NMR (377 MHz, $CDCl_3$) δ -191.51 (dt, *J* = 49.4, 25.1 Hz).

HRMS (ESI/QTOF) m/z: $[M + Na]^+$ Calcd for $C_{12}H_{19}FNaO_4^+$ 269.1165; Found 269.1169.

$[\alpha]_D^{23}$ = +0.9 (c = 0.33 in $CHCl_3$).

FTIR (neat): $\tilde{\nu}$ = 2928, 1760, 1735, 1437, 1199, 1029, 785 cm^{-1}.

HPLC: The enantiomeric ratio (60:40) was determined *via* HPLC analysis using a CHIRALCEL® IB column, with hexane:isopropanol = 99.5:0.5 at a flow rate 0.5 mL/min detected at 210 nm wavelength. Retention time: t_{major} = 16.8 min and t_{minor} = 16.1 min.

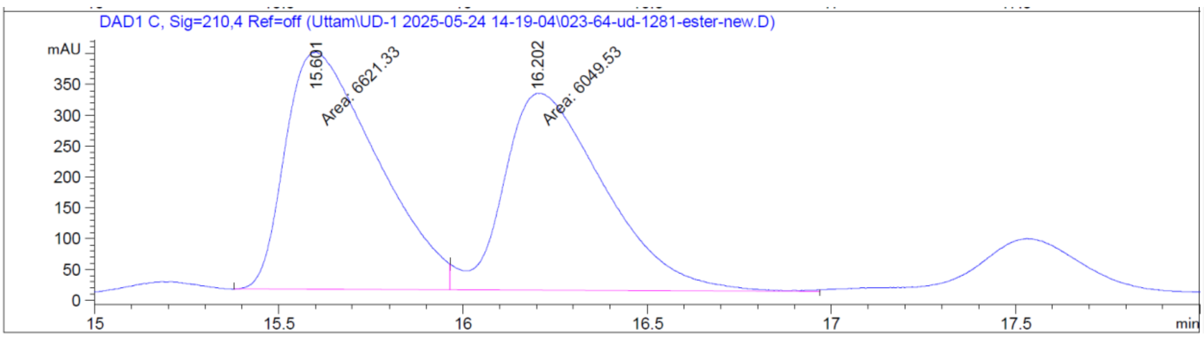

Peak #	RetTime [min]	Type	Width [min]	Area [mAU*s]	Height [mAU]	Area %
1	15.601	MF	0.2873	6621.32959	384.08585	52.2564
2	16.202	FM	0.3159	6049.52832	319.19333	47.7436

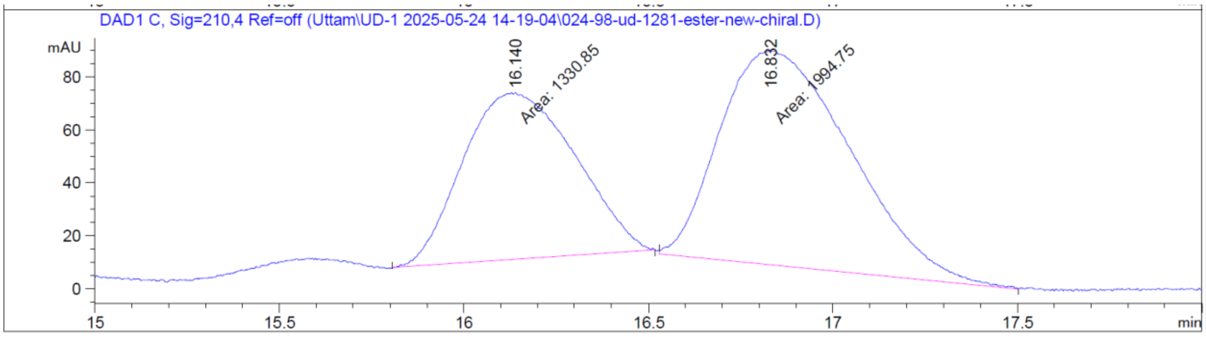

Peak #	RetTime [min]	Type	Width [min]	Area [mAU*s]	Height [mAU]	Area %
1	16.140	MM	0.3527	1330.84766	62.89734	40.0183
2	16.832	MM	0.4106	1994.74878	80.97864	59.9817

(*R*,*E*)-5-Cyclohexyl-2-fluoropent-4-enamide (S9)

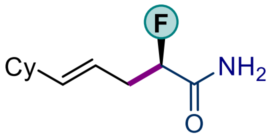

A modified procedure **GP1** was followed with 2-bromo-2-fluoroacetamide (15.6 mg, 0.10 mmol, 1 equiv.) and propa-1,2-dienylcyclohexane (29.1 µL, 0.20 mmol, 2 equiv.) at 0 °C. Automated flash column chromatography (10 g SiO_2, gradient elution: hexane to 50% EtOAc in hexane) afforded the desired product (+) **S9** as a colourless oil (5.4 mg, 27%).

^{1}H NMR (400 MHz, $CDCl_3$) δ 6.18 (s, 1H), 5.77 (s, 1H), 5.49 (dd, J = 15.5, 6.7 Hz, 1H), 5.30 (dtd, J = 15.3, 6.9, 1.2 Hz, 1H), 4.82 (ddd, J = 49.6, 6.9, 3.9 Hz, 1H), 2.73 – 2.33 (m, 2H), 1.87 (tdt, J = 10.7, 6.8, 3.4 Hz, 1H), 1.70 – 1.56 (m, 5H), 1.26 – 0.90 (m, 5H).

^{13}C NMR (101 MHz, $CDCl_3$) δ 172.31 (d, $^{2}J_{C\text{–}F}$ = 20.6 Hz), 141.58, 119.68 (d, $^{3}J_{C\text{–}F}$ = 2.8 Hz), 91.41 (d, $^{1}J_{C\text{–}F}$ = 187.4 Hz), 40.68, 35.39 (d, $^{2}J_{C\text{–}F}$ = 20.2 Hz), 32.91, 32.88, 26.15, 25.99.

^{19}F{H} NMR (377 MHz, $CDCl_3$) δ -187.89.

^{19}F NMR (377 MHz, $CDCl_3$) δ -187.58 – -188.18 (m).

HRMS (ESI/QTOF) m/z: $[M + H]^+$ Calcd for $C_{11}H_{19}FNO^+$ 200.1451; Found 200.1454.

$[\alpha]_D^{23}$ = +18.6 (c = 0.33 in $CHCl_3$).

FTIR (neat): $\tilde{\nu}$ = 3393, 2923, 1656, 1113, 964, 657 cm^{-1}.

HPLC: The enantiomeric ratio (86:14) was determined *via* HPLC analysis using a CHIRALCEL® IB column, with hexane:isopropanol = 90:10 at a flow rate 1.0 mL/min detected at 210 nm wavelength. Retention time: t_{major} = 6.9 min and t_{minor} = 6.2 min.

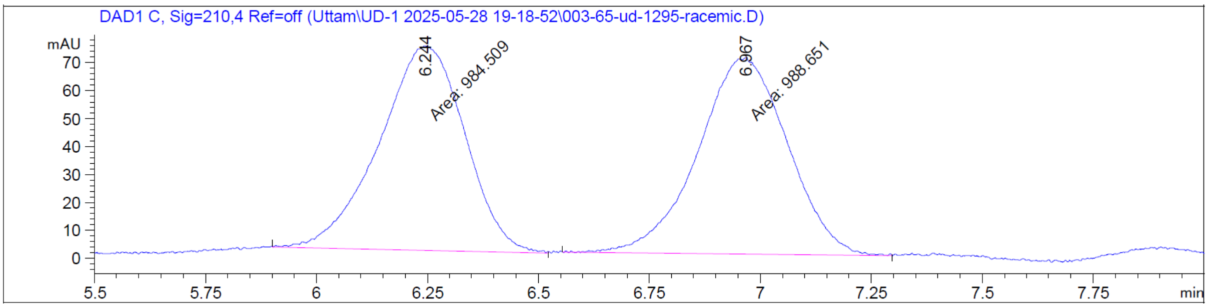

Peak #	RetTime [min]	Type	Width [min]	Area [mAU*s]	Height [mAU]	Area %
1	6.244	MM	0.2237	984.50909	73.35052	49.8950
2	6.967	MM	0.2340	988.65094	70.40954	50.1050

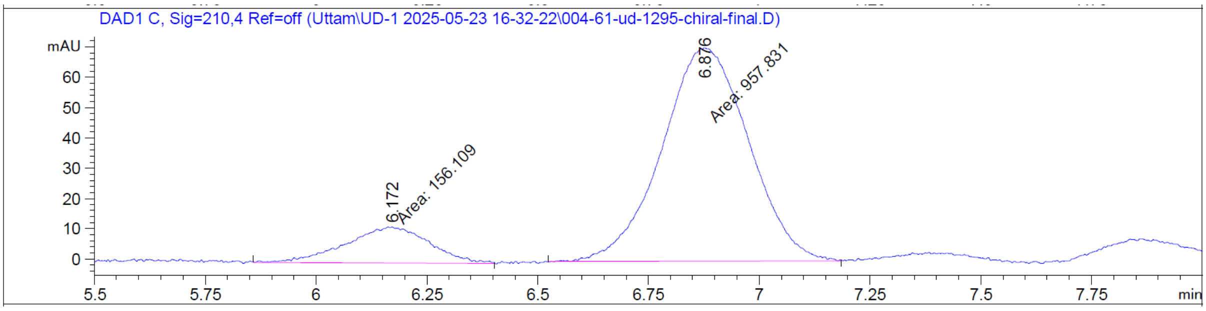

Peak #	RetTime [min]	Type	Width [min]	Area [mAU*s]	Height [mAU]	Area %
1	6.172	MM	0.2177	156.10948	11.95229	14.0142
2	6.876	MM	0.2269	957.83148	70.36507	85.9858

Methyl (*E*)-4-(4-chloro-5,5,5-trifluoropent-1-en-1-yl)benzoate (S10)

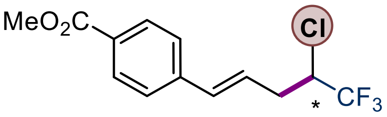

A modified procedure **GP1** was followed with 2-bromo-2-chloro-1,1,1-trifluoroethane (10.5 µL, 0.10 mmol, 1 equiv.) and methyl 4-(propa-1,2-dien-1-yl)benzoate (34.8 mg, 0.20 mmol, 2

equiv.) at 0 °C. Automated flash column chromatography (10 g SiO_2, gradient elution: hexane to 10% EtOAc in hexane) afforded the desired product (-) **S10** as a colourless oil (10.5 mg, 36%).

^{1}H NMR (400 MHz, $CDCl_3$) δ 7.93 (d, J = 8.3 Hz, 2H), 7.36 (d, J = 8.2 Hz, 2H), 6.53 (d, J = 15.8 Hz, 1H), 6.24 (dt, J = 15.2, 7.1 Hz, 1H), 4.20 – 4.06 (m, 1H), 3.85 (s, 3H), 2.95 – 2.82 (m, 1H), 2.68 (dt, J = 15.7, 8.6 Hz, 1H).

13C NMR (101 MHz, CDCl3) δ 166.81, 140.89, 133.86, 130.00, 129.33, 126.25, 125.21, 123.91 (q, $^{1}J_{C–F}$ = 271.9 Hz), 56.81 (q, $^{2}J_{C–F}$ = 32.8 Hz), 52.12, 34.98 (d, $^{3}J_{C–F}$ = 1.9 Hz).

^{19}F{H} NMR (376 MHz, $CDCl_3$) δ -74.35.

^{19}F NMR (376 MHz, $CDCl_3$) δ -74.35 (d, J = 6.8 Hz).

HRMS (nanochip-ESI/LTQ-Orbitrap) m/z: $[M + H]^+$ Calcd for $C_{13}H_{13}ClF_3O_2^+$ 293.0556; Found 293.0549.

$[\alpha]_D^{23}$ = -9.4 (c = 0.3 in $CHCl_3$).

FTIR (neat): $\tilde{\nu}$ = 2923, 1704, 1607, 1438, 1270, 1123, 682 cm^{-1}.

HPLC: The enantiomeric ratio (77:23) was determined *via* HPLC analysis using a CHIRALCEL® OD-H column, with hexane:isopropanol = 99:1 at a flow rate 1.0 mL/min detected at 254 nm wavelength. Retention time: t_{major} = 14.0 min and t_{minor} = 10.4 min.

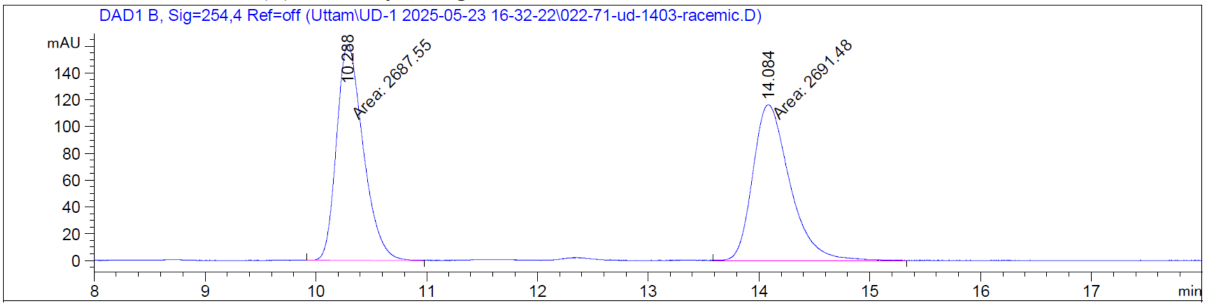

Peak #	RetTime [min]	Type	Width [min]	Area [mAU*s]	Height [mAU]	Area %
1	10.288	MM	0.2780	2687.54688	161.10043	49.9634
2	14.084	MM	0.3858	2691.48169	116.28645	50.0366

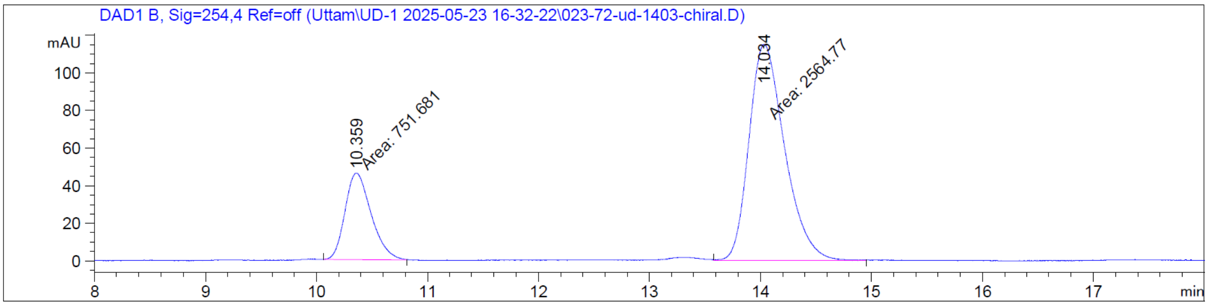

Peak #	RetTime [min]	Type	Width [min]	Area [mAU*s]	Height [mAU]	Area %
1	10.359	MM	0.2718	751.68127	46.08632	22.6652
2	14.034	MM	0.3719	2564.76782	114.92770	77.3348

7. Mechanistic experiments

a) Radical scavenger experiment

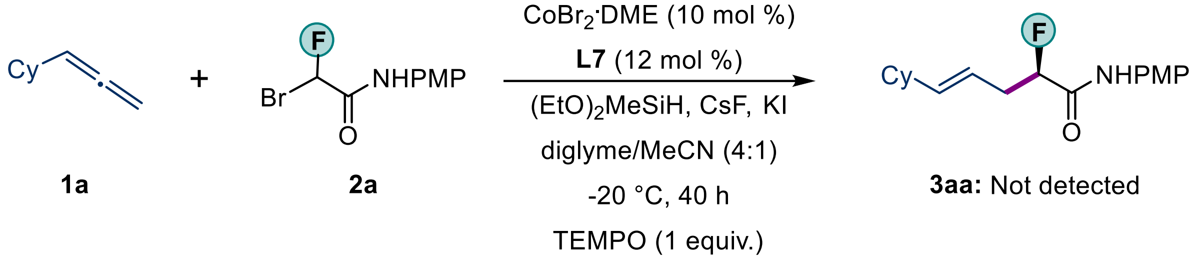

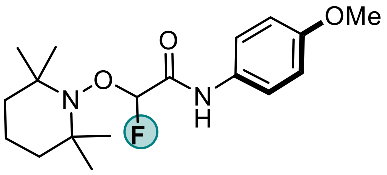

20: detected by HRMS

Analysis by mass spectrometry:
$[M + Na]^+$ m/z Calc.: 361.1903
Found: 361.1904

The reaction was conducted following **GP1**. TEMPO (15.6 mg, 0.10 mmol, 1.0 equiv.) was added after the addition of all other reagents. The hydroalkylation product **3aa** was not detected. TEMPO-adduct **20** was detected by HRMS.

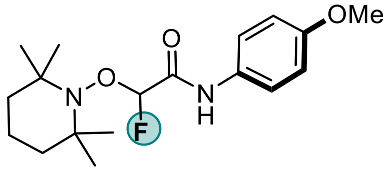

HRMS (ESI/QTOF) *m/z*: $[M + Na]^+$ Calcd for $C_{18}H_{27}FN_2NaO_3^+$ 361.1903; Found 361.1904.

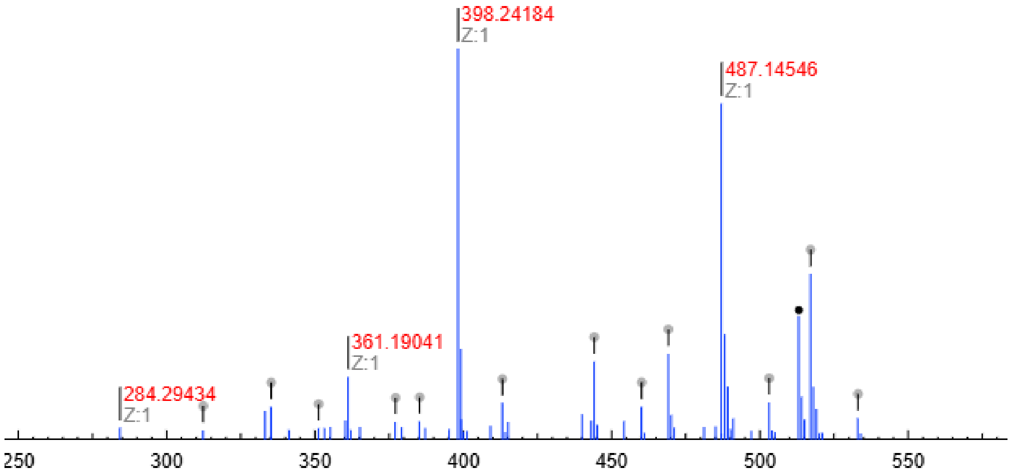

Figure S3. HRMS spectra of **20**.

b) Deuterium labelling experiment

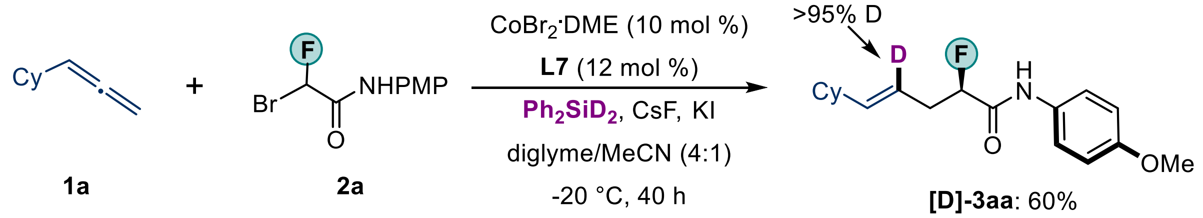

To an oven-dried 10 mL Teflon-screw capped vial was added cobalt(II) dibromo(1,2-dimethoxyethane) (3.09 mg, 0.01 mmol, 0.10 equiv.) and (4*S*,4'*S*)-2,2'-(1,3-bis(4-(trifluoromethyl)phenyl)propane-2,2-diyl)bis(4-phenyl-4,5-dihydrooxazole) (7.47 mg, 0.012 mmol, 0.12 equiv.). The vial was introduced in a nitrogen filled glovebox. A magnetic stir bar (6 x 15 mm) and anhydrous diglyme/MeCN (0.8 mL/0.2 mL) were added and the mixture was stirred for 30 min at room temperature until it became a clear blue solution. Then 2-bromo-2-fluoro-*N*-(4-methoxyphenyl)acetamide (**2a**) (26.2 mg, 0.10 mmol, 1.0 equiv.), propa-1,2-dienylcyclohexane (**1a**) (24.4 mg, 0.20 mmol, 2.0 equiv.), and anhydrous cesium fluoride (38 mg, 0.25 mmol, 2.5 equiv.), potassium iodide (16.6 mg, 0.10 mmol, 1.0 equiv.) were added to it. Then diphenyl(silane-d2) (23 µL, 0.125 mmol, 1.25 equiv.) was added dropwise to it. The vial was then sealed with airtight electrical tapes, removed from the glove box immediately, and stirred for 40 h at -20 °C, maintaining 600 rpm. After that, the reaction was diluted with EtOAc, transferred into a one-necked flask, and the solvents were removed under *vacuum*. Automated flash column chromatography (10 g SiO_2, gradient elution: hexane to 20% EtOAc in hexane) afforded the deuterated product **[D]-3aa** as a yellow oil (18.5 mg, 60%).

^{1}H NMR (400 MHz, $CDCl_3$) δ 7.84 (d, J = 6.4 Hz, 1H), 7.51 – 7.30 (m, 2H), 6.94 – 6.80 (m, 2H), 5.58 (d, J = 6.7 Hz, 1H), 5.00 (ddd, J = 49.7, 6.8, 3.8 Hz, 1H), 3.80 (s, 3H), 2.78 – 2.58 (m, 2H), 1.94 (dddt, J = 14.3, 10.6, 6.5, 3.2 Hz, 1H), 1.76 – 1.53 (m, 5H), 1.32 – 0.94 (m, 5H).

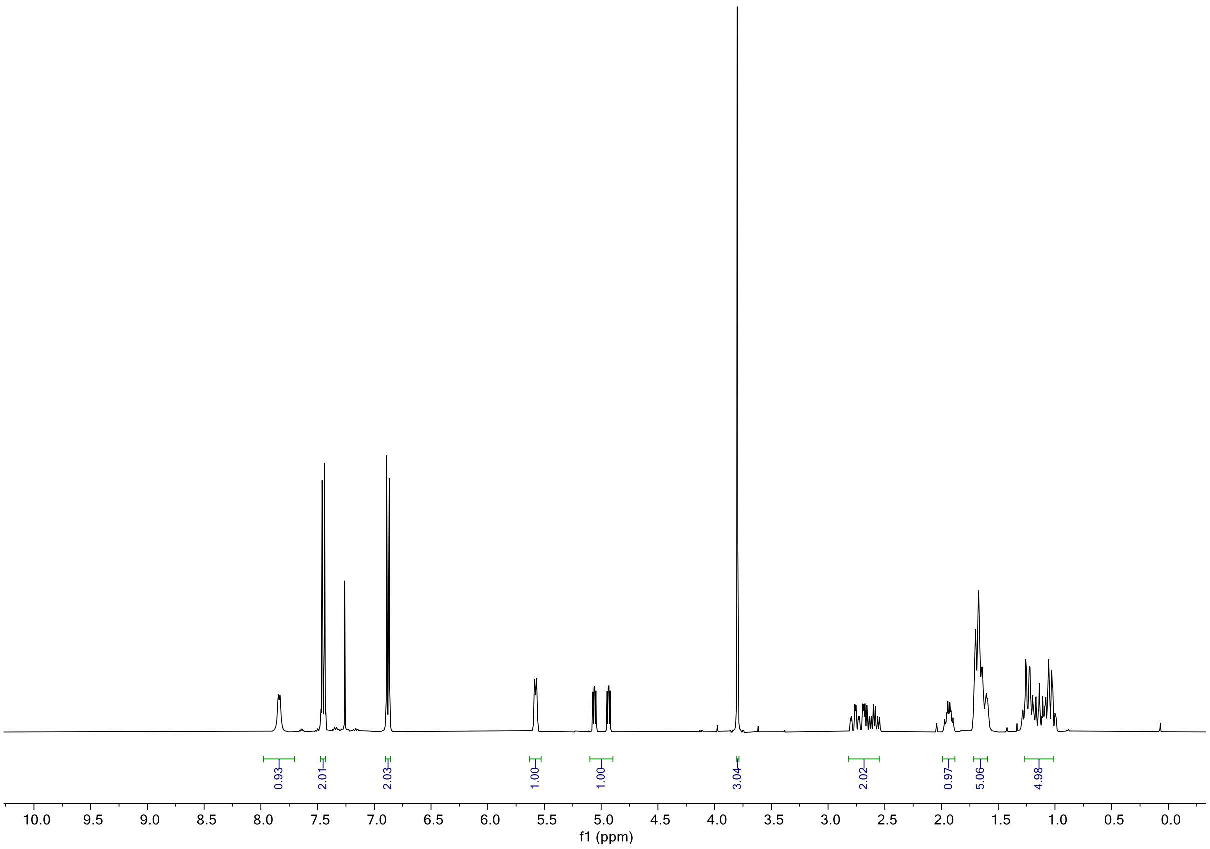

Figure S4. ^{1}H NMR spectra of H/D exchange experiment.

c) Cobalt complex (**21**) synthesis

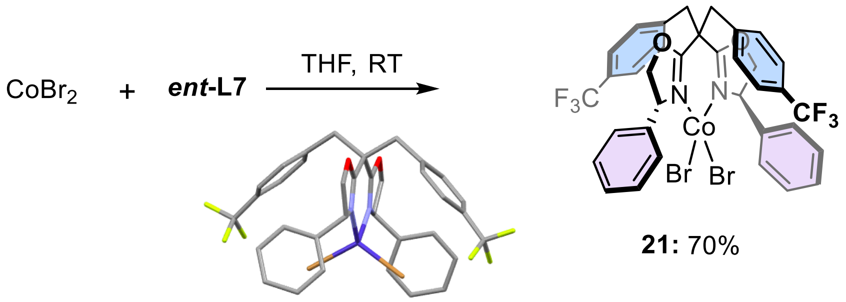

Prepared according to literature procedure:[58] To an oven-dried 10 mL Teflon-screw capped vial was added (4*R*,4'*R*)-2,2'-(1,3-bis(4-(trifluoromethyl)phenyl)propane-2,2-diyl)bis(4-phenyl-4,5-dihydrooxazole) ***ent*-L7** (249 mg, 400 µmol, 1.0 equiv.). The vial was introduced in a nitrogen filled glovebox and a magnetic stir bar (6 x 15 mm) was added. Then cobalt bromide (87.5 mg, 400 µmol, 1.0 equiv.) and anhydrous THF (4.0 mL) were added. The resulting solution was stirred for 16 h at room temperature. Hexane (5.0 mL) was then added, and after 5 minutes of stirring, the complex precipitated. The mixture was allowed to settle for 5 minutes, and the supernatant was decanted. The resulting cobalt-blue solid was washed and dried to yield the cobalt-blue powder **21** (235 mg, 70% yield).

HRMS (ESI/QTOF) m/z: $[M + Na]^+$ Calcd for $C_{35}H_{28}Br_2CoF_6N_2NaO_2^+$ 861.9651; Found 861.9671.

A sample of **21** was recrystallized by vapor diffusion from ether-THF for X-ray crystallography.

Cobalt complex (**21**) activity test:

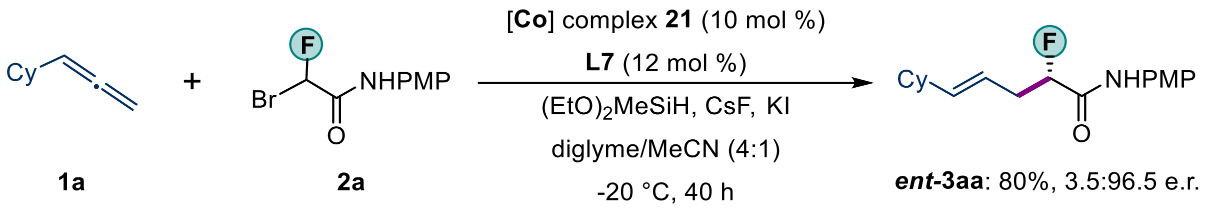

In a nitrogen filled glovebox, to an oven-dried 10 mL Teflon-screw capped vial cobalt complex **21** was added. A magnetic stir bar (6 x 15 mm) and anhydrous diglyme/MeCN (0.8 mL/0.2 mL) were added and the mixture was stirred for 30 min at room temperature until it became a clear blue solution. Then 2-bromo-2-fluoro-*N*-(4-methoxyphenyl)acetamide (**2a**) (26.2 mg, 0.10 mmol, 1.0 equiv.), propa-1,2-dienylcyclohexane (**1a**) (24.4 mg, 0.20 mmol, 2.0 equiv.), and anhydrous cesium fluoride (38 mg, 0.25 mmol, 2.5 equiv.), potassium iodide (16.6 mg, 0.10 mmol, 1.0 equiv.) were added to it. Then $(EtO)_2MeSiH$ (40.5 µL, 0.25 mmol, 0.25 equiv.) was added dropwise to it. The vial was then sealed with airtight electrical tapes, removed from the glove box immediately, and stirred for 40 h at -20 °C, maintaining 600 rpm. After that, the reaction was diluted with EtOAc, transferred into a one-necked flask, and the solvents were removed under *vacuum*. Automated flash column chromatography (10 g SiO_2, gradient elution: hexane to 20% EtOAc in hexane) afforded the desired product ***ent*-3aa** in 80% yield with 3.5:96.5 e.r.

HPLC: The enantiomeric ratio (3.5:96.5) was determined *via* HPLC analysis using a CHIRALCEL® IA column, with hexane:isopropanol = 95:5 at a flow rate 1.0 mL/min detected at 254 nm wavelength. Retention time: t_{major} = 17.5 min and t_{minor} = 13.5 min.

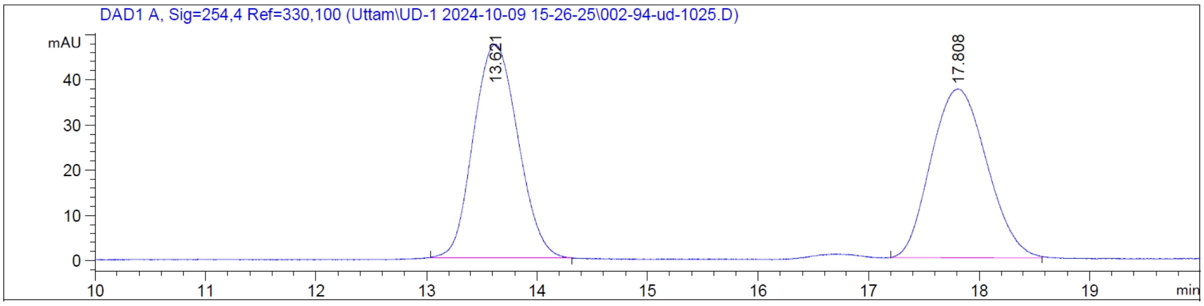

Peak #	RetTime [min]	Type	Width [min]	Area [mAU*s]	Height [mAU]	Area %
1	13.621	VB	0.3293	1322.78088	47.29702	50.2327
2	17.808	BV	0.4112	1310.52637	37.36232	49.7673

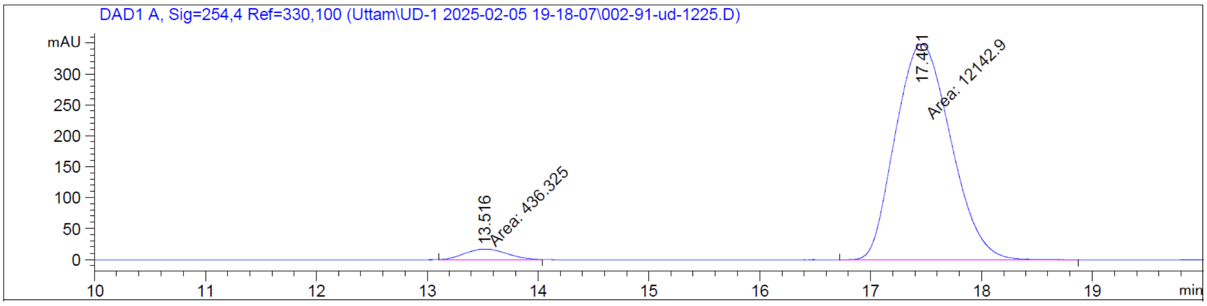

#	[min]		[min]	[mAU*s]	[mAU]	%
1	13.516	MM	0.4344	436.32535	16.74025	3.4686
2	17.461	MM	0.5793	1.21429e4	349.36548	96.5314

d) Non-linear effect study

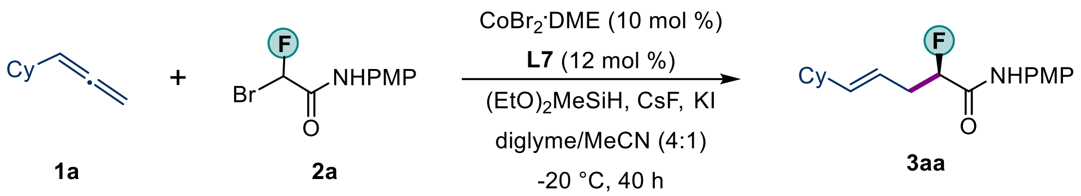

Six parallel independent reactions were conducted following **GP1**, with **1a** and **2a** as coupling partners. The crude mixture was purified by automated flash column chromatography (10 g SiO_2, gradient elution: hexane to 20% EtOAc in hexane). The enantiomeric excess was determined by HPLC with chiral stationary phase.

Table S2. Non-linear effect study.

entry	e.e. of the chiral ligand **L7**	e.r. of the product **3aa**
1	0	0
2	10	53:47
3	30	63:37
4	50	72.5:27.5
5	70	82:18
6	90	90.5:9.5
7	100	96.5:3.5

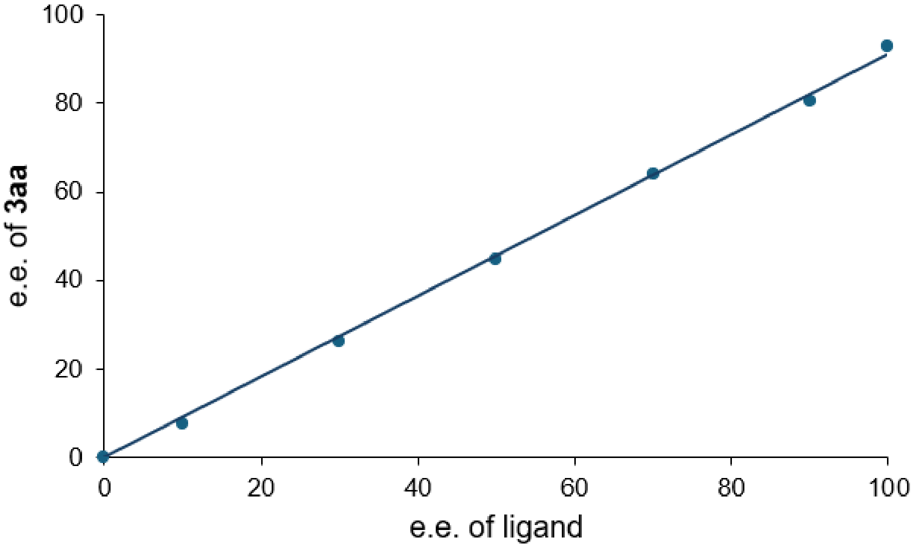

Figure S5. Absence of a non-linear effect.

e) Reaction rates comparison among possible intermediates

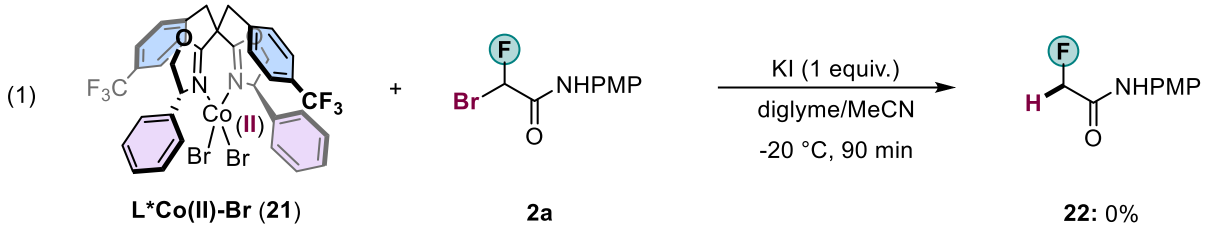

In a nitrogen-filled glovebox, an oven-dried 16 mL Teflon screw-cap vial was charged with L*Co(II)Br_2 (**21**) (25.2 mg, 0.030 mmol, 1.0 equiv), a magnetic stir bar (6 × 15 mm), and anhydrous diglyme/MeCN (0.8 mL:0.2 mL). The mixture was stirred at room temperature for 20 minutes. Subsequently, 2-bromo-2-fluoro-*N*-(4-methoxyphenyl)acetamide (**2a**) (7.86 mg, 0.030 mmol, 1.0 equiv) and potassium iodide (5.0 mg, 0.030 mmol, 1.0 equiv) were added. The vial was sealed with airtight electrical tape, removed from the glovebox, and stirred at –20 °C for 90 minutes at 600 rpm. The reaction was quenched with H_2O (0.1 mL), extracted with EtOAc, and transferred to a one-necked flask. Solvents were removed under reduced pressure. The yield was determined by ^{19}F NMR spectroscopy using $PhCF_3$ as internal standard and no desired product **22** was detected (0% yield).

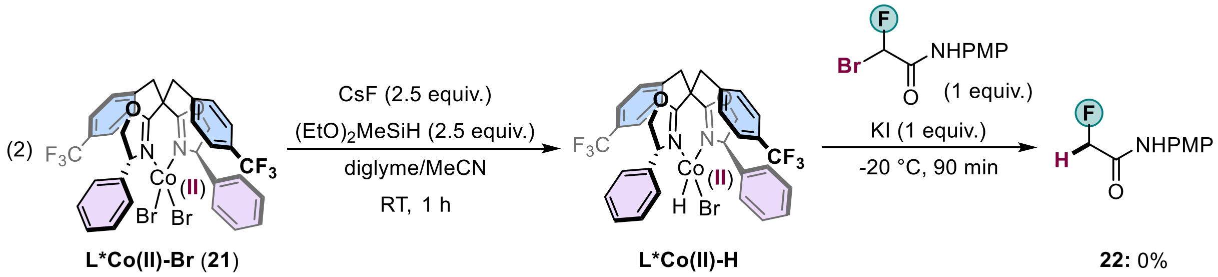

In a nitrogen-filled glovebox, an oven-dried 16 mL Teflon screw-cap vial was charged with L*Co(II)Br_2 (**21**) (25.2 mg, 0.030 mmol, 1.0 equiv), a magnetic stir bar (6 × 15 mm), and anhydrous diglyme/MeCN (0.8 mL:0.2 mL). The solution was stirred at room temperature for 20 minutes, after which cesium fluoride (11.4 mg, 0.075 mmol, 2.5 equiv) was added, followed by the dropwise addition of methyldiethoxysilane (12.1 μL, 0.075 mmol, 2.5 equiv). The resulting mixture was stirred at RT for an additional 1 hour. Subsequently, 2-bromo-2-fluoro-*N*-(4-methoxyphenyl)acetamide (**2a**) (7.86 mg, 0.030 mmol, 1.0 equiv) and potassium iodide (5.0 mg, 0.030 mmol, 1.0 equiv) were added. The vial was sealed with airtight electrical tape, removed from the glovebox, and stirred at –20 °C for 90 min at 600 rpm. The reaction was quenched by the addition of H_2O (0.1 mL), extracted with EtOAc, and transferred to a one-necked flask. The solvents were removed under reduced pressure. The crude product was

analyzed by ^{19}F NMR spectroscopy using $PhCF_3$ as an internal standard, and no desired product **22** was detected (0% yield).

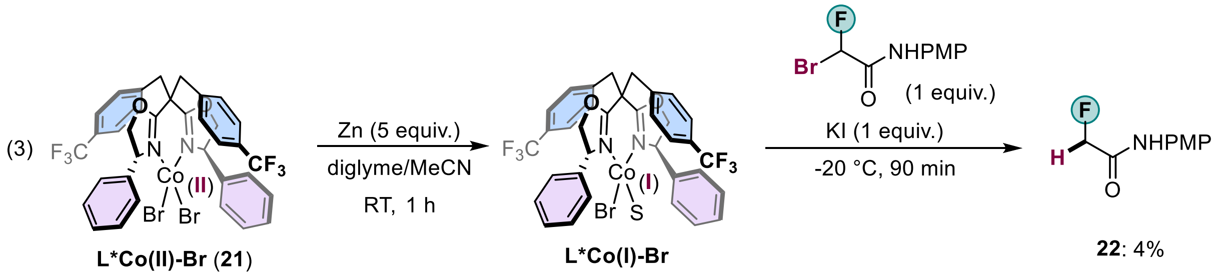

In a nitrogen-filled glovebox, an oven-dried 16 mL Teflon screw-cap vial was charged with L*Co(II)Br_2 (**21**) (25.2 mg, 0.030 mmol, 1.0 equiv), a magnetic stir bar (6 × 15 mm), and anhydrous diglyme/MeCN (0.8 mL:0.2 mL). The solution was stirred at room temperature for 20 minutes, after which zinc (9.81 mg, 0.15 mmol, 5 equiv.) was added and stirred for 1 h. Subsequently, 2-bromo-2-fluoro-*N*-(4-methoxyphenyl)acetamide (**2a**) (7.86 mg, 0.030 mmol, 1.0 equiv) and potassium iodide (5.0 mg, 0.030 mmol, 1.0 equiv) were added. The vial was sealed with airtight electrical tape, removed from the glovebox, and stirred at –20 °C for 90 min at 600 rpm. The reaction was quenched by the addition of H_2O (0.1 mL), extracted with EtOAc, and transferred to a one-necked flask. The solvents were removed under reduced pressure. The crude product was analyzed by ^{19}F NMR spectroscopy using $PhCF_3$ as an internal standard, and the desired product **22** was detected in 4% yield.

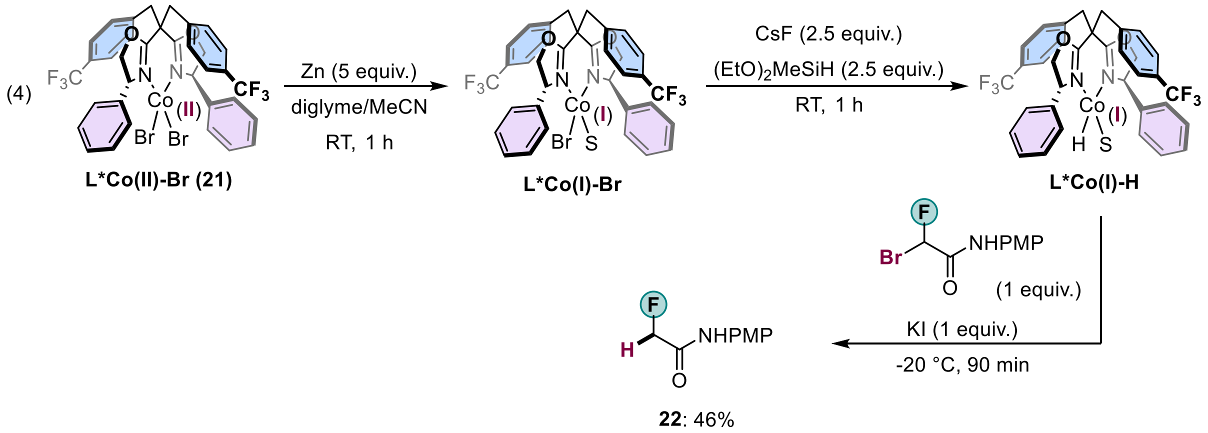

In a nitrogen-filled glovebox, an oven-dried 16 mL Teflon screw-cap vial was charged with L*Co(II)Br_2 (**21**) (25.2 mg, 0.030 mmol, 1.0 equiv), a magnetic stir bar (6 × 15 mm), and anhydrous diglyme/MeCN (0.8 mL:0.2 mL). The solution was stirred at room temperature for 20 minutes, after which zinc (9.81 mg, 0.15 mmol, 5 eq.) was added and stirred for 1 h. Then cesium fluoride (11.4 mg, 0.075 mmol, 2.5 equiv) was added, followed by the dropwise addition of methyldiethoxysilane (12.1 µL, 0.075 mmol, 2.5 equiv). The resulting mixture was stirred at RT for an additional 1 hour. Subsequently, 2-bromo-2-fluoro-*N*-(4-

methoxyphenyl)acetamide (**2a**) (7.86 mg, 0.030 mmol, 1.0 equiv) and potassium iodide (5.0 mg, 0.030 mmol, 1.0 equiv) were added. The vial was sealed with airtight electrical tape, removed from the glovebox, and stirred at –20 °C for 90 min at 600 rpm. The reaction was quenched by the addition of H_2O (0.1 mL), extracted with EtOAc, and transferred to a one-necked flask. The solvents were removed under reduced pressure. The crude product was analyzed by ^{19}F NMR spectroscopy using $PhCF_3$ as an internal standard, and the desired product **22** was detected in 46% yield.

8. Crystallography Details

Compound (+) 3ac:

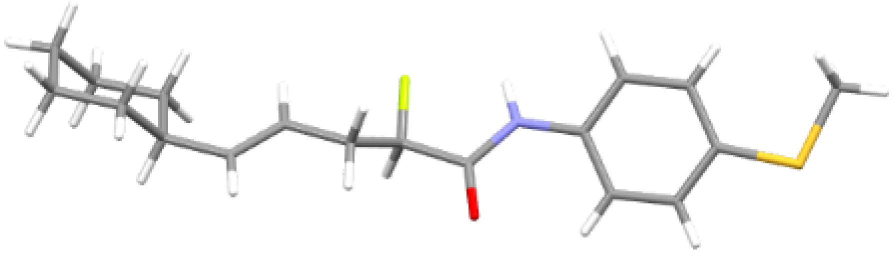

Experimental. Single clear pale colourless prism-shaped crystals of **3ac** were used as supplied. A suitable crystal with dimensions 0.29 × 0.05 × 0.04 mm^3 was selected and mounted on a SuperNova, Dual, Cu at home/near, AtlasS2 diffractometer. The crystal was kept at a steady T = 140.00(10) K during data collection. The structure was solved with the ShelXT (Sheldrick, 2015) solution program using dual methods and by using Olex2 1.5 (Dolomanov et al., 2009) as the graphical interface. The model was refined with ShelXL 2019/3 (Sheldrick, 2015) using full matrix least squares minimisation on $\boldsymbol{F}^2$.

Crystal Data. $C_{18}H_{24}FNOS$, M_r = 321.44, monoclinic, $P2_1$ (No. 4), a = 11.3054(8) Å, b = 5.2963(3) Å, c = 14.1454(8) Å, β = 98.137(6)°, α = γ = 90°, V = 838.45(9) Å^3, T = 140.00(10) K, Z = 2, Z' = 1, μ(Cu K$_\alpha$) = 1.808, 6242 reflections measured, 3243 unique (R_{int} = 0.0347) which were used in all calculations. The final wR_2 was 0.1150 (all data) and R_1 was 0.0438 (I≥2 σ(I)).

**Compound**	**3ac**
Formula	$C_{18}H_{24}FNOS$
$D_{calc.}$/ g cm^{-3}	1.273
μ/mm^{-1}	1.808
Formula Weight	321.44
Colour	clear pale colourless
Shape	prism
Size/mm^3	0.29×0.05×0.04
T/K	140.00(10)
Crystal System	monoclinic
Flack Parameter	0.003(17)
Hooft Parameter	0.003(17)
Space Group	$P2_1$
a/Å	11.3054(8)
b/Å	5.2963(3)
c/Å	14.1454(8)
α/°	90
β/°	98.137(6)
γ/°	90
V/Å^3	838.45(9)
Z	2
Z'	1
Wavelength/Å	1.54184
Radiation type	Cu K□
Θ_{min}/°	3.156
Θ_{max}/°	73.174
Measured Refl's.	6242
Indep't Refl's	3243
Refl's I≥2 σ(I)	2901
R_{int}	0.0347
Parameters	200
Restraints	1
Largest Peak	0.331
Deepest Hole	-0.247
GooF	1.023
wR_2 (all data)	0.1150
wR_2	0.1091
R_1 (all data)	0.0506
R_1	0.0438
**CCDC**	**2425950**

Structure Quality Indicators

**Reflections:**	d min (CuKα) 2Θ=146.3° 0.81	I/σ(I) 20.6	Rint m=1.93 3.47%	Full 135.4° 99% to 146.3° 100
**Refinement:**	Shift 0.000	Max Peak 0.3	Min Peak -0.2	GooF 1.023
				Hooft .003(17)

A clear pale colourless prism-shaped crystal with dimensions 0.29 × 0.05 × 0.04 mm^3 was mounted. Data were collected using a SuperNova, Dual, Cu at home/near, AtlasS2 diffractometer operating at T = 140.00(10) K.

Data were measured using ω scans with Cu K_α radiation. The diffraction pattern was indexed and the total number of runs and images was based on the strategy calculation from the program CrysAlisPro system (CCD 43.143a 64-bit (release 25-10-2024)). The maximum resolution that was achieved was Θ = 73.174° (0.81 Å).

The unit cell was refined using CrysAlisPro 1.171.43.143a (Rigaku OD, 2024) on 3443 reflections, 55% of the observed reflections.

Data reduction, scaling and absorption corrections were performed using CrysAlisPro 1.171.43.143a (Rigaku OD, 2024). The final completeness is 100.00 % out to 73.174° in Θ. An analytical absorption correction was performed using CrysAlisPro 1.171.43.143a (Rigaku Oxford Diffraction, 2024). The analytical numeric absorption correction was done using a multifaceted crystal model based on expressions derived by R.C. Clark & J.S. Reid. (Clark, R. C. & Reid, J. S. (1995). Acta Cryst. A51, 887-897). The empirical absorption correction was done using spherical harmonics, implemented in SCALE3 ABSPACK scaling algorithm. The absorption coefficient μ of this crystal is 1.808 mm^{-1} at this wavelength (λ = 1.54184Å) and the minimum and maximum transmissions are 0.766 and 0.940.

The structure was solved and the space group $P2_1$ (# 4) determined by the ShelXT (Sheldrick, 2015) structure solution program using dual methods and refined by full matrix least squares minimisation on $\boldsymbol{F}^2$ using version 2019/3 of ShelXL (Sheldrick, 2015). All non-hydrogen atoms were refined anisotropically. Hydrogen atom positions were calculated geometrically and refined using the riding model.

There is a single formula unit in the asymmetric unit, which is represented by the reported sum formula. In other words: Z is 2 and Z' is 1. The moiety formula is C18 H24 F N O S.

The Flack parameter was refined to 0.003(17). Determination of absolute structure using Bayesian statistics on Bijvoet differences using the Olex2 results in 0.003(17). The chiral atoms in this structure are: C9(R). Note: The Flack parameter is used to determine chirality of the crystal studied, the value should be near 0, a value of 1 means that the stereochemistry is wrong and the model should be inverted. A value of 0.5 means that the crystal consists of a racemic mixture of the two enantiomers.

CCDC- 2425950 contains the supplementary crystallographic data for (+) **3ac**. These data can be obtained free of charge from The Cambridge Crystallographic Data Centre via www.ccdc.cam.ac.uk/data_request/cif.

Compound (+) 5ah:

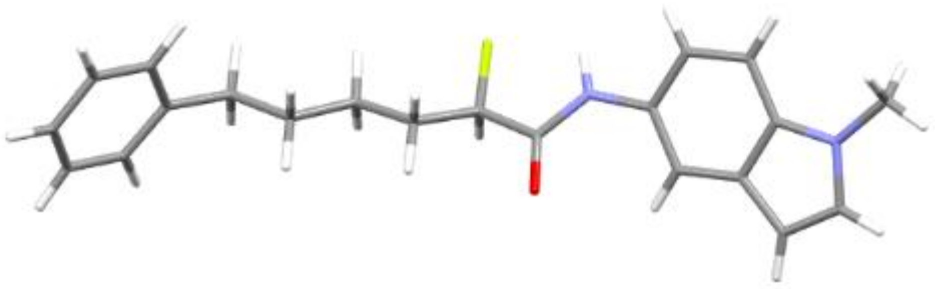

Experimental. Single clear pale colourless needle-shaped crystals of **5ah** were used as supplied. A suitable crystal with dimensions 0.31 × 0.04 × 0.03 mm^3 was selected and mounted on a XtaLAB Synergy R, DW system, HyPix-Arc 150 diffractometer. The crystal was kept at a steady T = 140.00(10) K during data collection. The structure was solved with the ShelXT (Sheldrick, 2015) solution program using dual methods and by using Olex2 1.5 (Dolomanov et al., 2009) as the graphical interface. The model was refined with ShelXL 2019/3 (Sheldrick, 2015) using full matrix least squares minimisation on $\boldsymbol{F^2}$.

Crystal Data. $C_{21}H_{23}FN_2O$, M_r = 338.41, monoclinic, C2 (No. 5), a = 26.2990(4) Å, b = 5.48156(8) Å, c = 12.9130(2) Å, β = 92.9719(14)°, $\alpha = \gamma$ = 90°, V = 1859.03(5) Å^3, T = 140.00(10) K, Z = 4, Z' = 1, μ(Cu K$_\alpha$) = 0.656, 19902 reflections measured, 3790 unique (R_{int} = 0.0272) which were used in all calculations. The final wR_2 was 0.0868 (all data) and R_1 was 0.0349 (I≥2 σ(I)).

**Compound**	**5ah**
Formula	$C_{21}H_{23}FN_2O$
$D_{calc.}$/ g cm^{-3}	1.209
μ/mm^{-1}	0.656
Formula Weight	338.41
Colour	clear pale colourless
Shape	needle
Size/mm^3	0.31×0.04×0.03
T/K	140.00(10)
Crystal System	monoclinic
Flack Parameter	-0.03(8)
Hooft Parameter	-0.03(8)
Space Group	$C2$
a/Å	26.2990(4)
b/Å	5.48156(8)
c/Å	12.9130(2)
α/°	90
β/°	92.9719(14)
γ/°	90
V/Å^3	1859.03(5)
Z	4
Z'	1
Wavelength/Å	1.54184
Radiation type	Cu K□
Θ_{min}/°	3.366
Θ_{max}/°	75.557
Measured Refl's.	19902
Indep't Refl's	3790
Refl's I≥2 σ(I)	3513
R_{int}	0.0272
Parameters	319
Restraints	1
Largest Peak	0.141
Deepest Hole	-0.129
GooF	1.022
wR_2 (all data)	0.0868
wR_2	0.0850
R_1 (all data)	0.0389
R_1	0.0349
**CCDC**	**2425951**

Structure Quality Indicators

**Reflections:**	d min (CuKα) 2Θ=151.1° 0.80	I/σ(I) 40.4	Rint m=5.25 2.72%	Full 135.4° 99% to 151.1° 100	
**Refinement:**	Shift 0.000	Max Peak 0.1	Min Peak -0.1	GooF 1.022	Hooft -.03(8)

A clear pale colourless needle-shaped crystal with dimensions 0.31 × 0.04 × 0.03 mm^3 was mounted. Data were collected using a XtaLAB Synergy R, DW system, HyPix-Arc 150 diffractometer operating at T = 140.00(10) K.

Data were measured using ω scans with Cu K_α radiation. The diffraction pattern was indexed and the total number of runs and images was based on the strategy calculation from the program CrysAlisPro system (CCD 44.91a 64-bit (release 23-01-2025)). The maximum resolution that was achieved was Θ = 75.557° (0.80 Å).

The unit cell was refined using CrysAlisPro 1.171.44.92a (Rigaku OD, 2025) on 7564 reflections, 38% of the observed reflections.

Data reduction, scaling and absorption corrections were performed using CrysAlisPro 1.171.44.92a (Rigaku OD, 2025). The final completeness is 100.00 % out to 75.557° in Θ. An analytical absorption correction was performed using CrysAlisPro 1.171.44.92a (Rigaku Oxford Diffraction, 2025). The analytical numeric absorption correction was done using a multifaceted crystal model based on expressions derived by R.C. Clark & J.S. Reid. (Clark, R. C. & Reid, J. S. (1995). Acta Cryst. A51, 887-897). The empirical absorption correction was done using spherical harmonics, implemented in SCALE3 ABSPACK scaling algorithm. The absorption coefficient μ of this crystal is 0.656 mm^{-1} at this wavelength (λ = 1.54184Å) and the minimum and maximum transmissions are 0.880 and 0.985.

The structure was solved and the space group $C2$ (# 5) determined by the ShelXT (Sheldrick, 2015) structure solution program using dual methods and refined by full matrix least squares minimisation on $\boldsymbol{F}^2$ using version 2019/3 of ShelXL (Sheldrick, 2015). All non-hydrogen atoms were refined anisotropically. Hydrogen atom positions were calculated geometrically and refined freely.

There is a single formula unit in the asymmetric unit, which is represented by the reported sum formula. In other words: Z is 4 and Z' is 1. The moiety formula is C21 H23 F N2 O.

The Flack parameter was refined to -0.03(8). Determination of absolute structure using Bayesian statistics on Bijvoet differences using the Olex2 results in -0.03(8). The chiral atoms in this structure are: C11(R). Note: The Flack parameter is used to determine chirality of the crystal studied, the value should be near 0, a value of 1 means that the stereochemistry is wrong and the model should be inverted. A value of 0.5 means that the crystal consists of a racemic mixture of the two enantiomers.

CCDC- 2425951 contains the supplementary crystallographic data for (+) **5ah**. These data can be obtained free of charge from The Cambridge Crystallographic Data Centre via www.ccdc.cam.ac.uk/data_request/cif.

Compound 21:

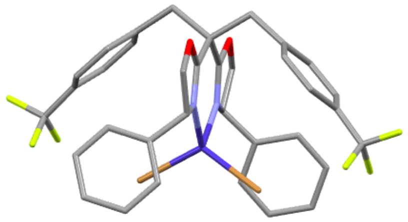

Experimental. Single clear light blue needle-shaped crystals of **21** were used as supplied. A suitable crystal with dimensions 0.22 × 0.01 × 0.01 mm^3 was selected and mounted on an XtaLAB Synergy R, DW system, HyPix-Arc 150 diffractometer. The crystal was kept at a steady T = 139.99(10) K during data collection. The structure was solved with the ShelXT 2018/2 (Sheldrick, 2015) solution program using dual methods and by using Olex2 1.5 (Dolomanov et al., 2009) as the graphical interface. The model was refined with ShelXL 2019/3 (Sheldrick, 2015) using full matrix least squares minimisation on $\boldsymbol{F}^2$.

Crystal Data. $C_{35}H_{28}Br_2CoF_6N_2O_2$, M_r = 841.34, monoclinic, $I2$ (No. 5), a = 16.0353(4) Å, b = 14.0547(4) Å, c = 22.7546(6) Å, β = 97.484(3)°, $\alpha = \gamma$ = 90°, V = 5084.6(2) Å^3, T = 139.99(10) K, Z = 6, Z' = 1.5, μ(Cu K$_\alpha$) = 7.329, 13743 reflections measured, 4304 unique (R_{int} = 0.0432) which were used in all calculations. The final wR_2 was 0.2199 (all data) and R_1 was 0.0816 (I≥2σ(I)).

**Compound**	**21**
Formula	$C_{35}H_{28}Br_2CoF_6N_2O_2$
$D_{calc.}$/ g cm^{-3}	1.649
μ/mm^{-1}	7.329
Formula Weight	841.34
Colour	clear light blue
Shape	needle-shaped
Size/mm^3	0.22×0.01×0.01
T/K	139.99(10)
Crystal System	monoclinic
Flack Parameter	-0.018(5)
Space Group	$I2$
a/Å	16.0353(4)
b/Å	14.0547(4)
c/Å	22.7546(6)
α/°	90
β/°	97.484(3)
γ/°	90
V/Å^3	5084.6(2)
Z	6
Z'	1.5
Wavelength/Å	1.54184
Radiation type	Cu$K\alpha$
Θ_{min}/°	3.185
Θ_{max}/°	51.421
Measured Refl's.	13743
Indep't Refl's	4304
Refl's I≥2σ(I)	3241
R_{int}	0.0432
Parameters	578
Restraints	1055
Largest Peak/e Å^{-3}	1.220
Deepest Hole/e Å^{-3}	-1.264
GooF	1.038
wR_2 (all data)	0.2199
wR_2	0.1995
R_1 (all data)	0.1094
R_1	0.0816
CCDC number	**2440842**

Structure Quality Indicators

**Reflections:**	d min (CuKα) 2Θ=102.8° 0.99	I/σ(I) 14.3	Rint m=3.19 4.32%	Full 102.8° 97.8	
**Refinement:**	Shift 0.000	Max Peak 1.2	Min Peak -1.3	GooF 1.038	Hooft -.018(5)

A clear light blue needle-shaped crystal with dimensions 0.22 × 0.01 × 0.01 mm^3 was mounted. Data were collected using an XtaLAB Synergy R, DW system, HyPix-Arc 150 diffractometer operating at T = 139.99(10) K.

Data were measured using ω scans with CuK_{α} radiation. The diffraction pattern was indexed and the total number of runs and images was based on the strategy calculation from the program CrysAlisPro system (CCD 44.93a 64-bit (release 10-02-2025)). The maximum resolution achieved was Θ = 51.421° (0.99 Å).

The unit cell was refined using CrysAlisPro 1.171.44.93a (Rigaku OD, 2025) on 5159 reflections, 38% of the observed reflections.

Data reduction, scaling and absorption corrections were performed using CrysAlisPro 1.171.44.93a (Rigaku OD, 2025). The final completeness is 97.80 % out to 51.421° in Θ. A Gaussian absorption correction was performed using CrysAlisPro 1.171.44.93a (Rigaku Oxford Diffraction, 2025) Numerical absorption correction based on Gaussian integration over a multifaceted crystal model. Empirical absorption correction using spherical harmonics as implemented in SCALE3 ABSPACK scaling algorithm. The absorption coefficient μ of this material is 7.329 mm^{-1} at this wavelength (λ = 1.54184Å) and the minimum and maximum transmissions are 0.391 and 1.000.

The structure was solved in the space group $I2$ (# 5) by the ShelXT 2018/2 (Sheldrick, 2015) structure solution program using dual methods and refined by full matrix least squares minimisation on $\boldsymbol{F}^2$ using version 2019/3 of ShelXL 2019/3 (Sheldrick, 2015). All non-hydrogen atoms were refined anisotropically. Hydrogen atom positions were calculated geometrically and refined using the riding model.

The value of Z' is 1.5. The moiety formula is C35 H28 Br2 Co F6 N2 O2.

The Flack parameter was refined to -0.018(5). Determination of absolute structure using Bayesian statistics on Bijvoet differences using the Olex2 results in None. The chiral atoms in this structure are: C1(R), C7(R), C38(R). Note: The Flack parameter is used to determine chirality of the crystal studied, the value should be near 0, a value of 1 means that the stereochemistry is wrong, and the model should be inverted. A value of 0.5 means that the crystal consists of a racemic mixture of the two enantiomers.

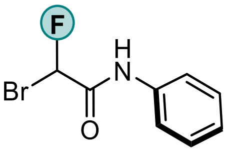

2b
($CDCl_3$, ^1H NMR)

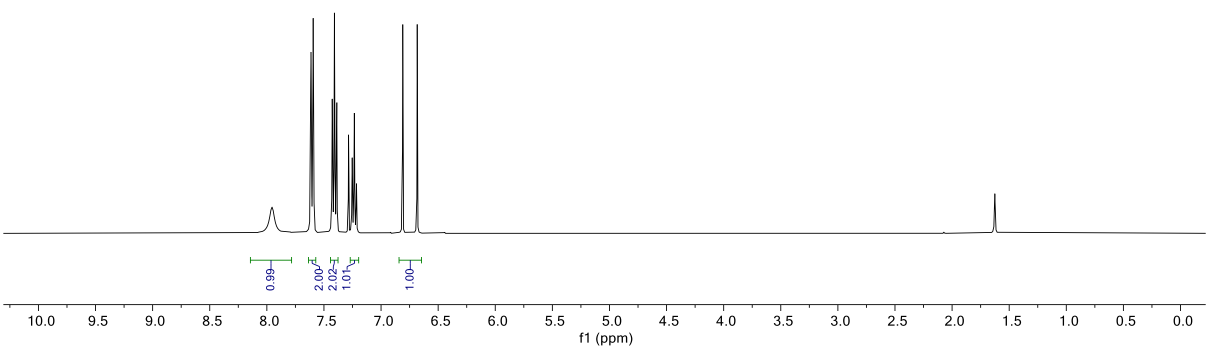

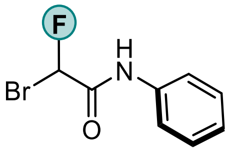

2b
($CDCl_3$, ^{13}C NMR)

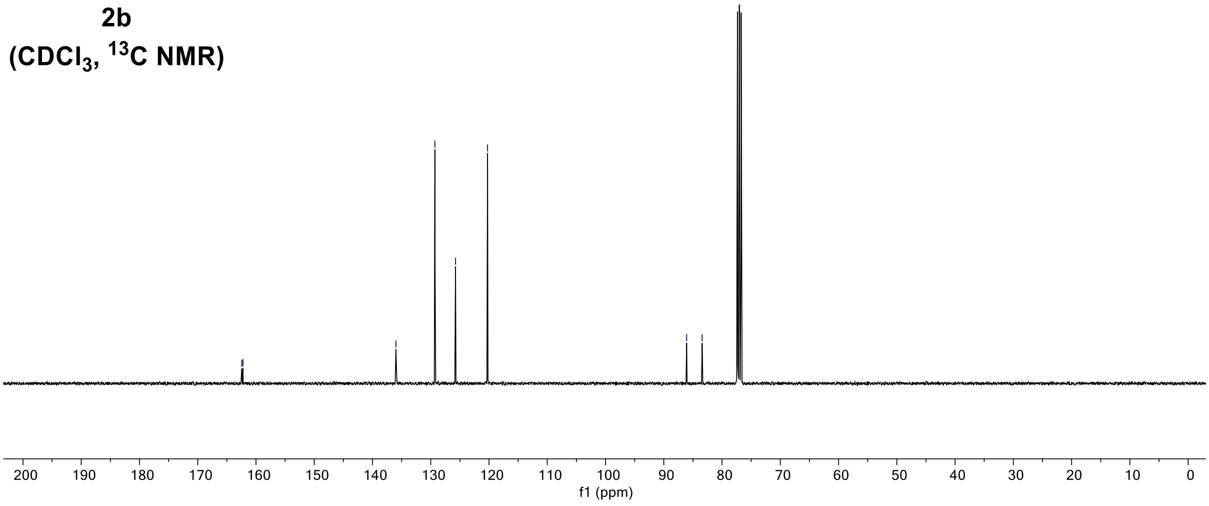

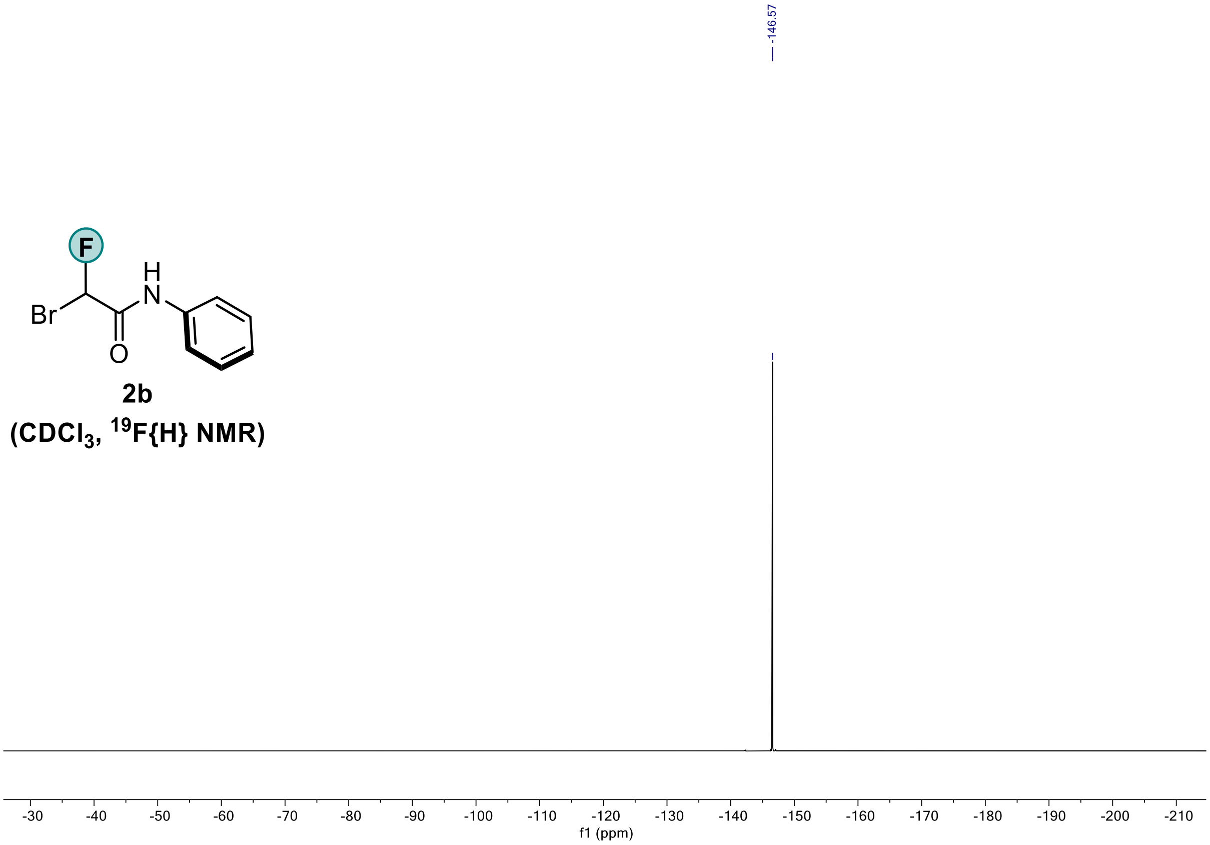

2b
($CDCl_3$, $^{19}F\{H\}$ NMR)

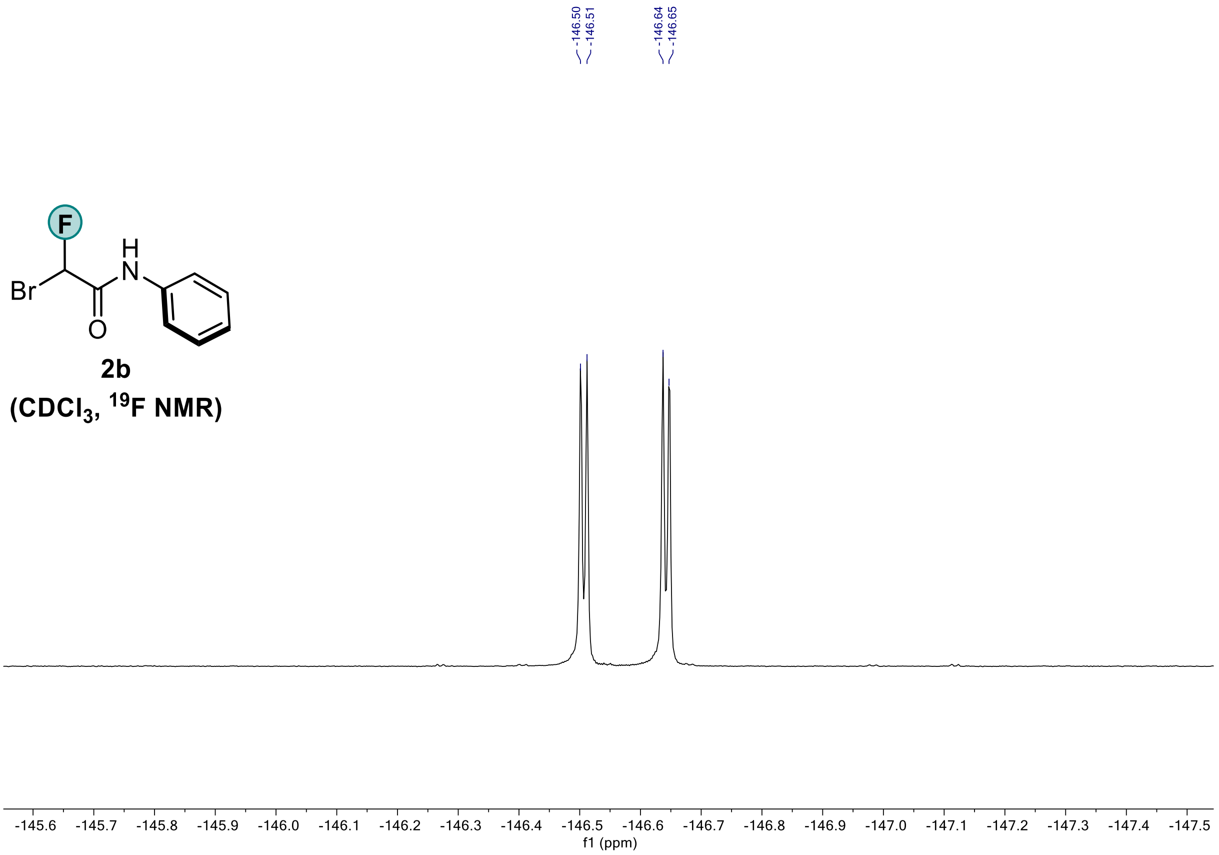

2b
($CDCl_3$, ^{19}F NMR)

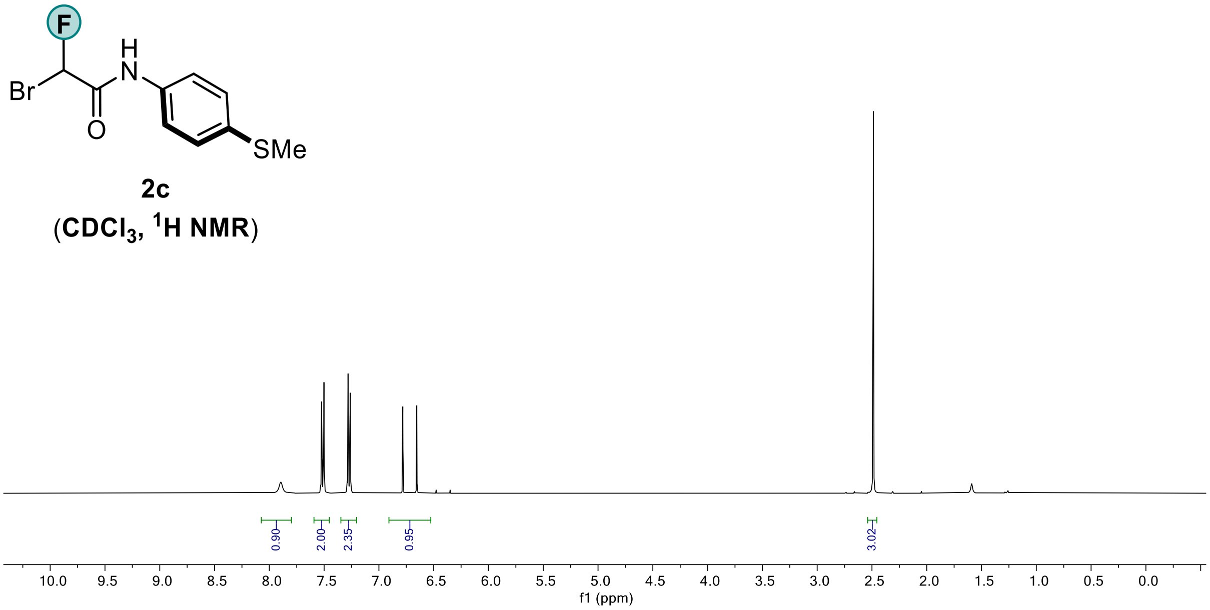

2c
(**CDCl$_3$, ^{1}H NMR**)

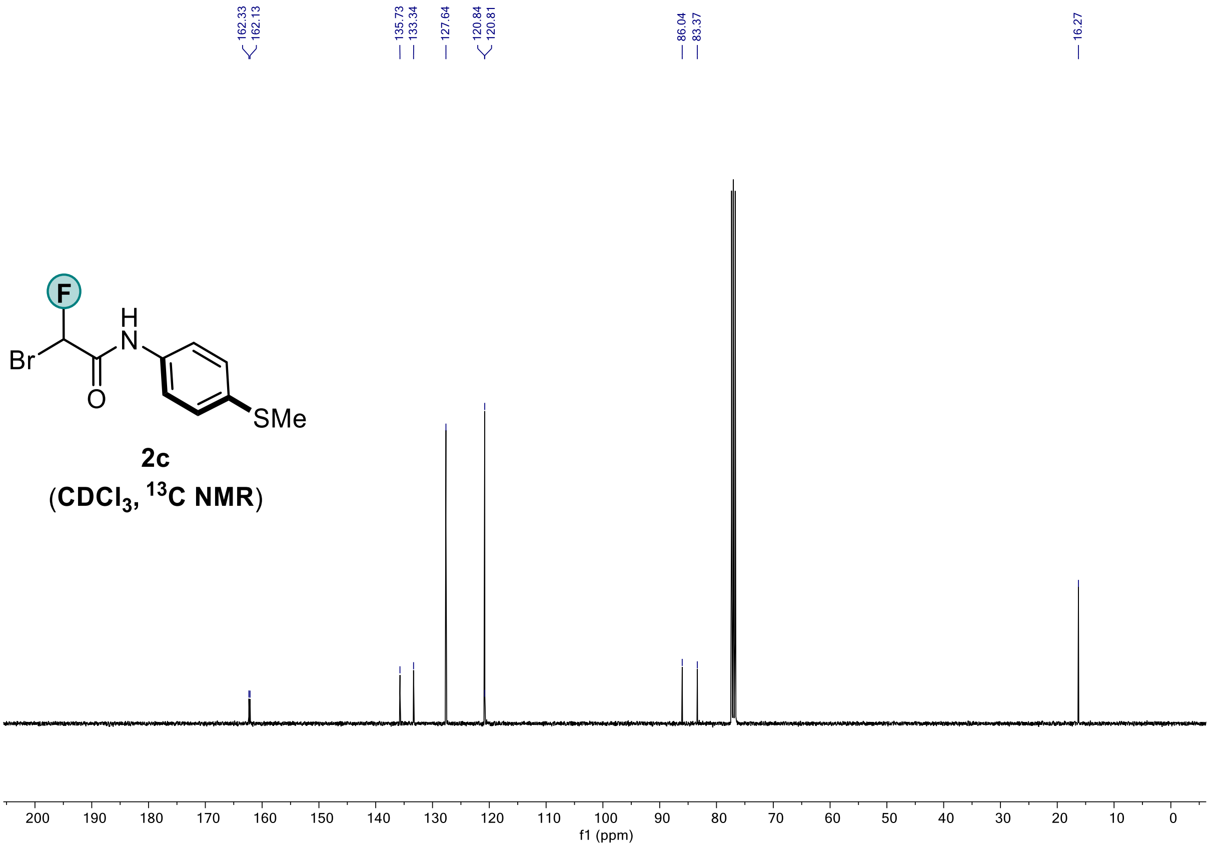

2c
(**CDCl$_3$, ^{13}C NMR**)

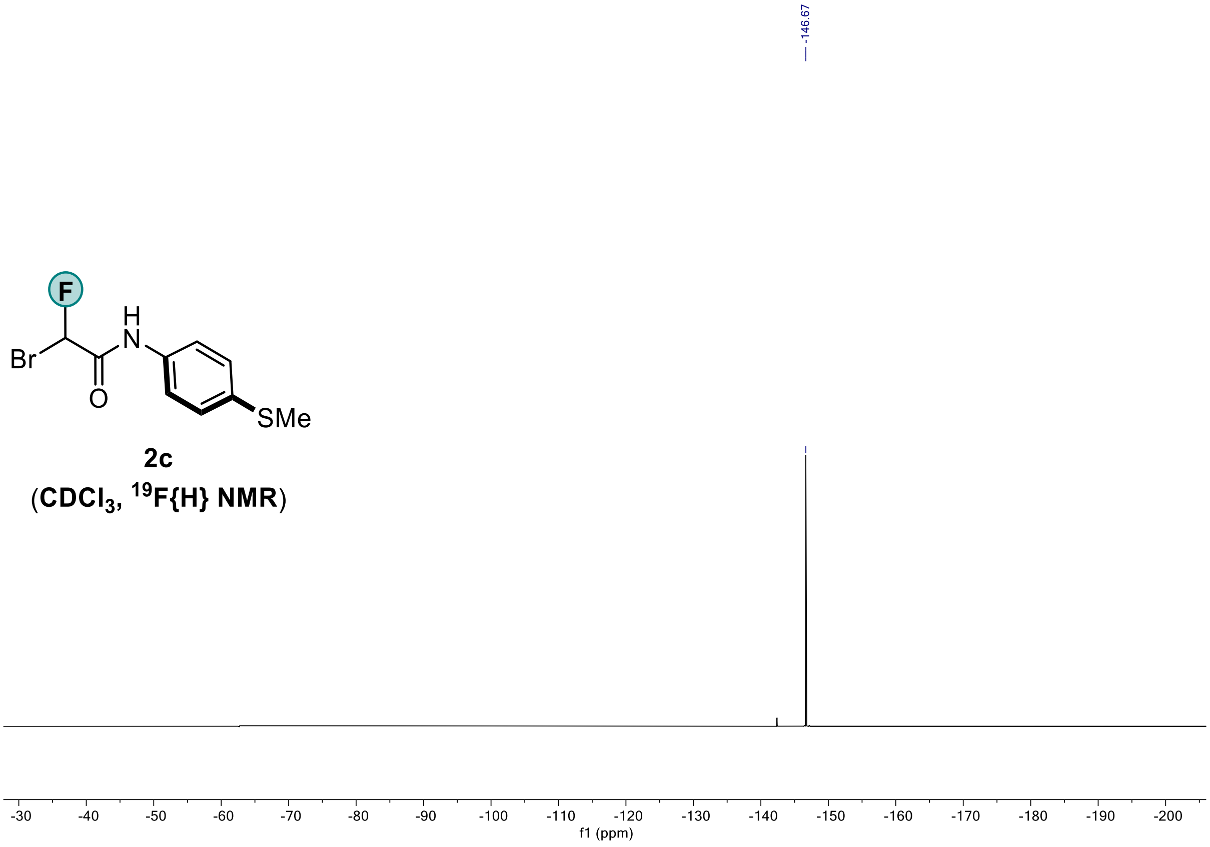
-146.67
2c
(CDCl3, 19F{H} NMR)
-30 -40 -50 -60 -70 -80 -90 -100 -110 -120 -130 -140 -150 -160 -170 -180 -190 -200
f1 (ppm)

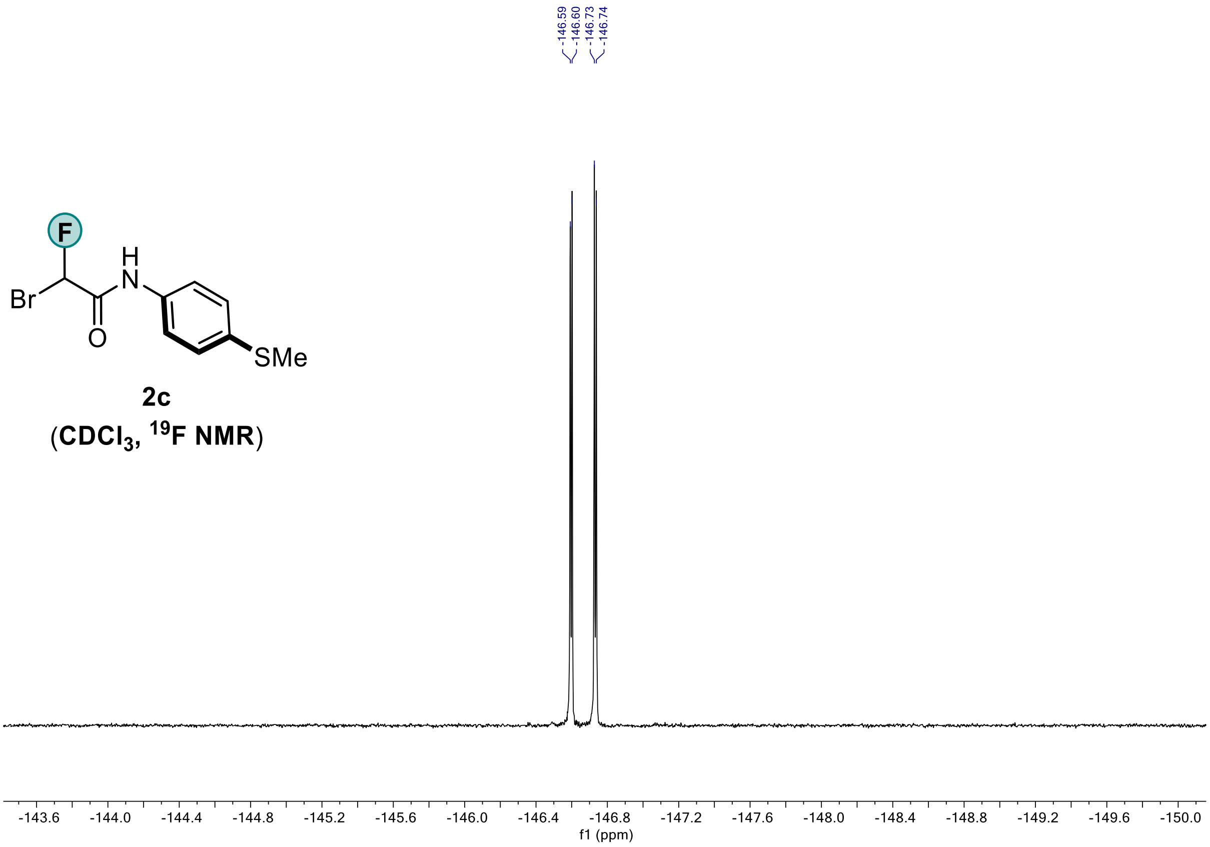
-146.59
-146.60
-146.73
-146.74
2c
(CDCl3, 19F NMR)
-143.6 -144.0 -144.4 -144.8 -145.2 -145.6 -146.0 -146.4 -146.8 -147.2 -147.6 -148.0 -148.4 -148.8 -149.2 -149.6 -150.0
f1 (ppm)

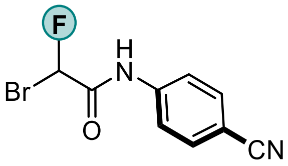

2d
(**CDCl$_3$, ^{1}H NMR**)

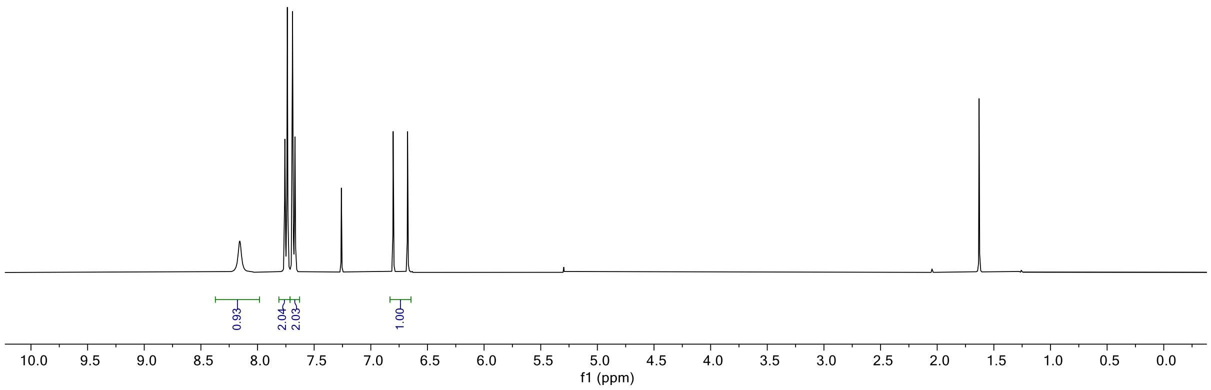

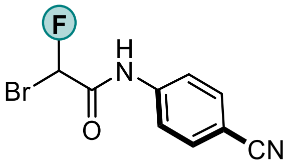

2d
(**CDCl$_3$, ^{13}C NMR**)

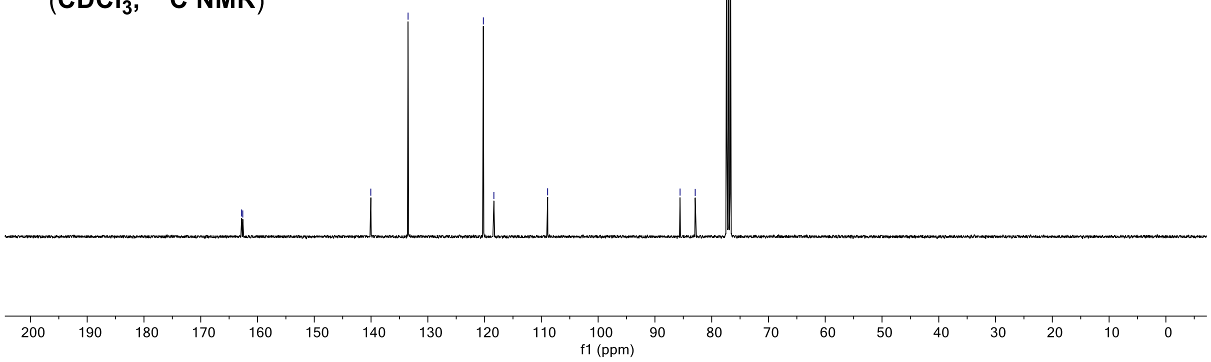

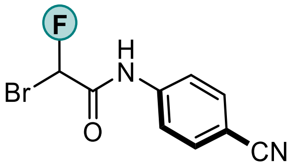

2d
($CDCl_3$, $^{19}F\{H\}$ NMR)

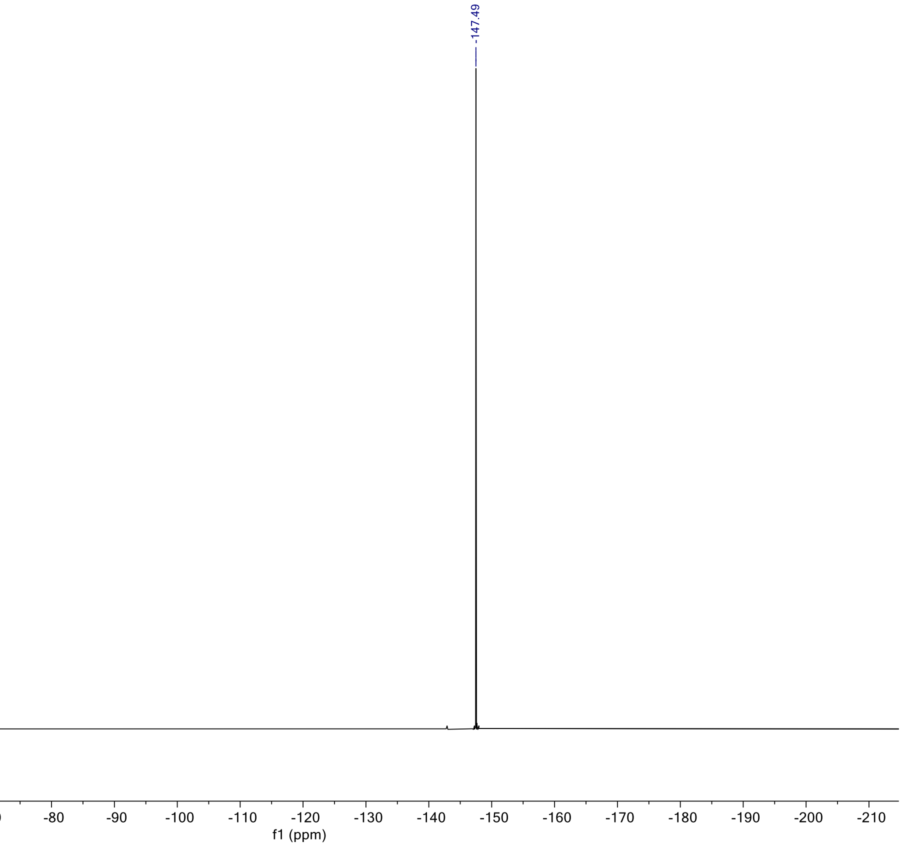

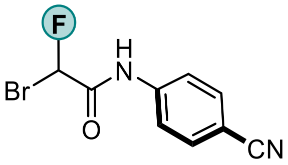

2d
($CDCl_3$, ^{19}F NMR)

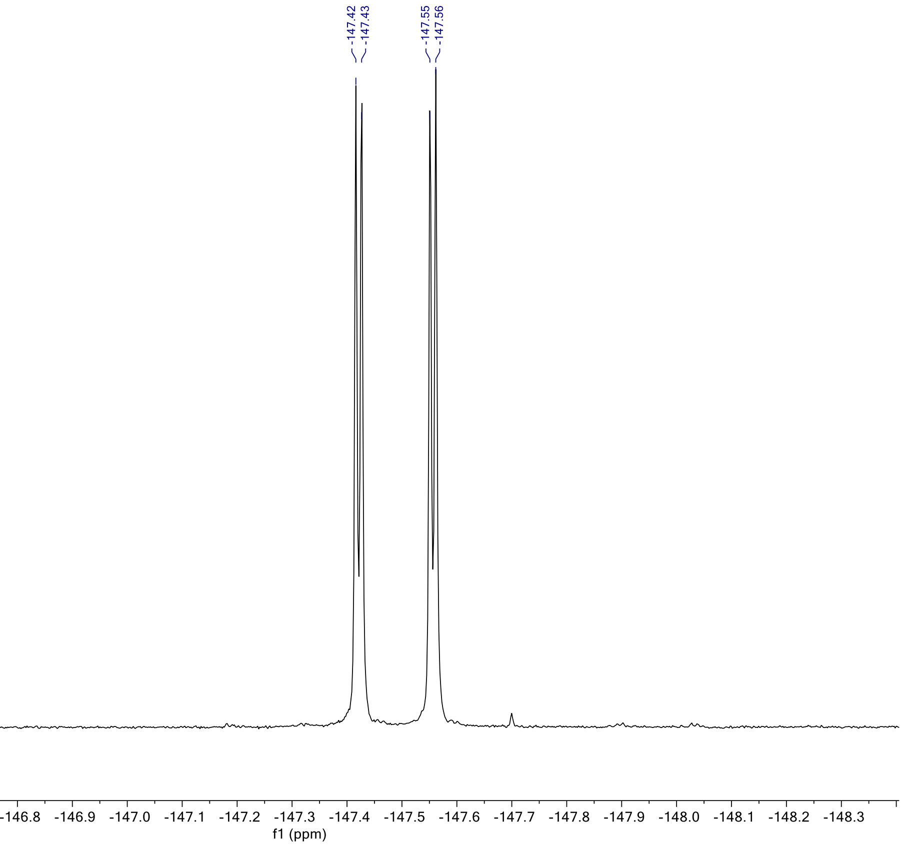

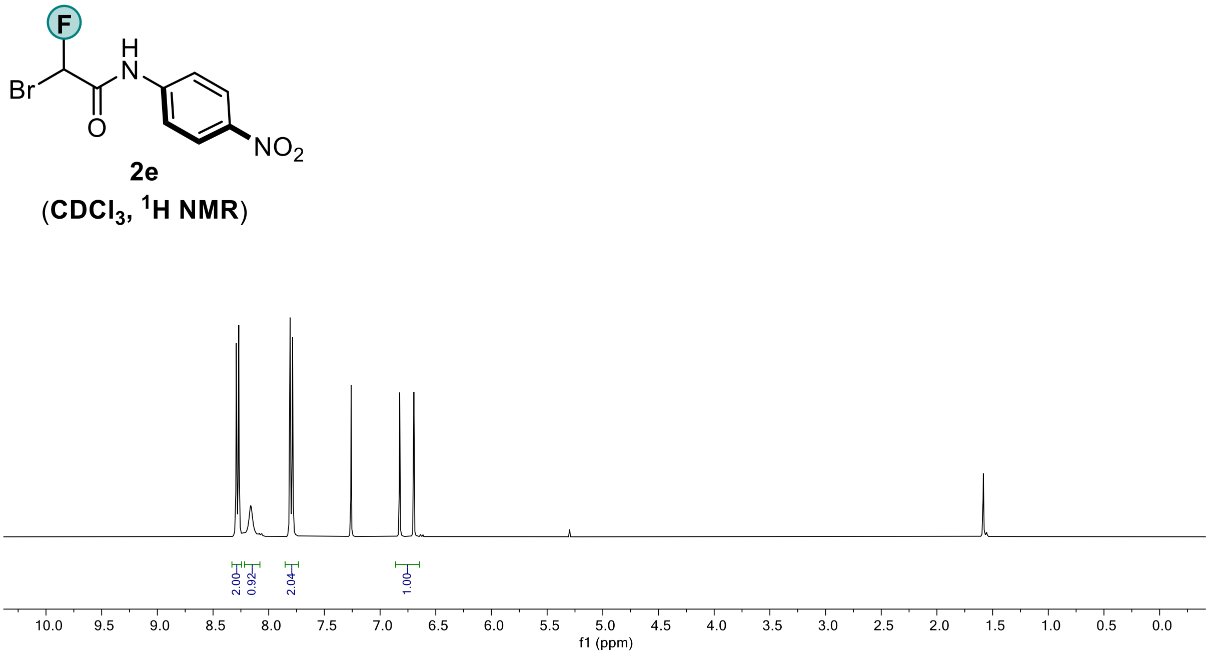
F
H
N
Br
O
NO2
2e
(CDCl3, 1H NMR)
2.00
0.92
2.04
1.00
10.0
9.5
9.0
8.5
8.0
7.5
7.0
6.5
6.0
5.5
5.0
4.5
4.0
3.5
3.0
2.5
2.0
1.5
1.0
0.5
0.0
f1 (ppm)

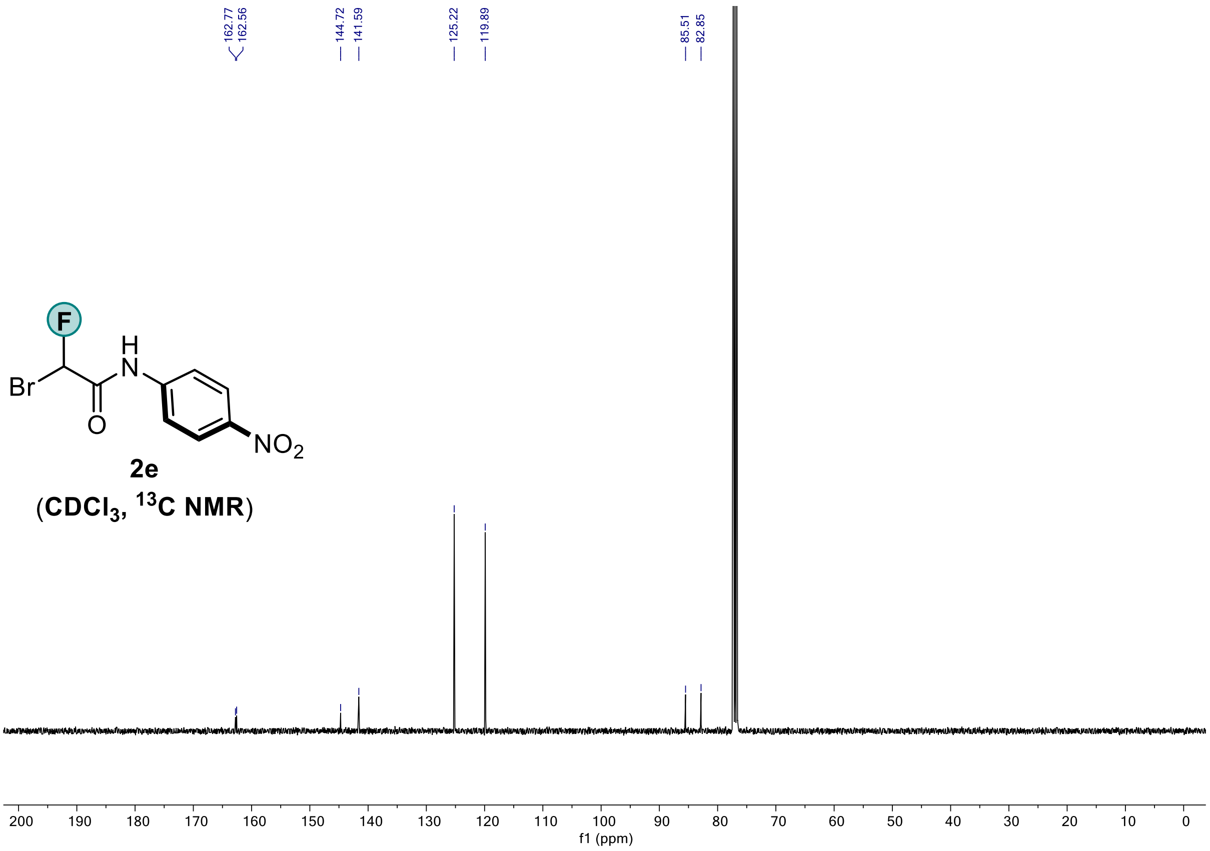
162.77
162.56
144.72
141.59
125.22
119.89
85.51
82.85
F
H
N
Br
O
NO2
2e
(CDCl3, 13C NMR)
200
190
180
170
160
150
140
130
120
110
100
90
80
70
60
50
40
30
20
10
0
f1 (ppm)

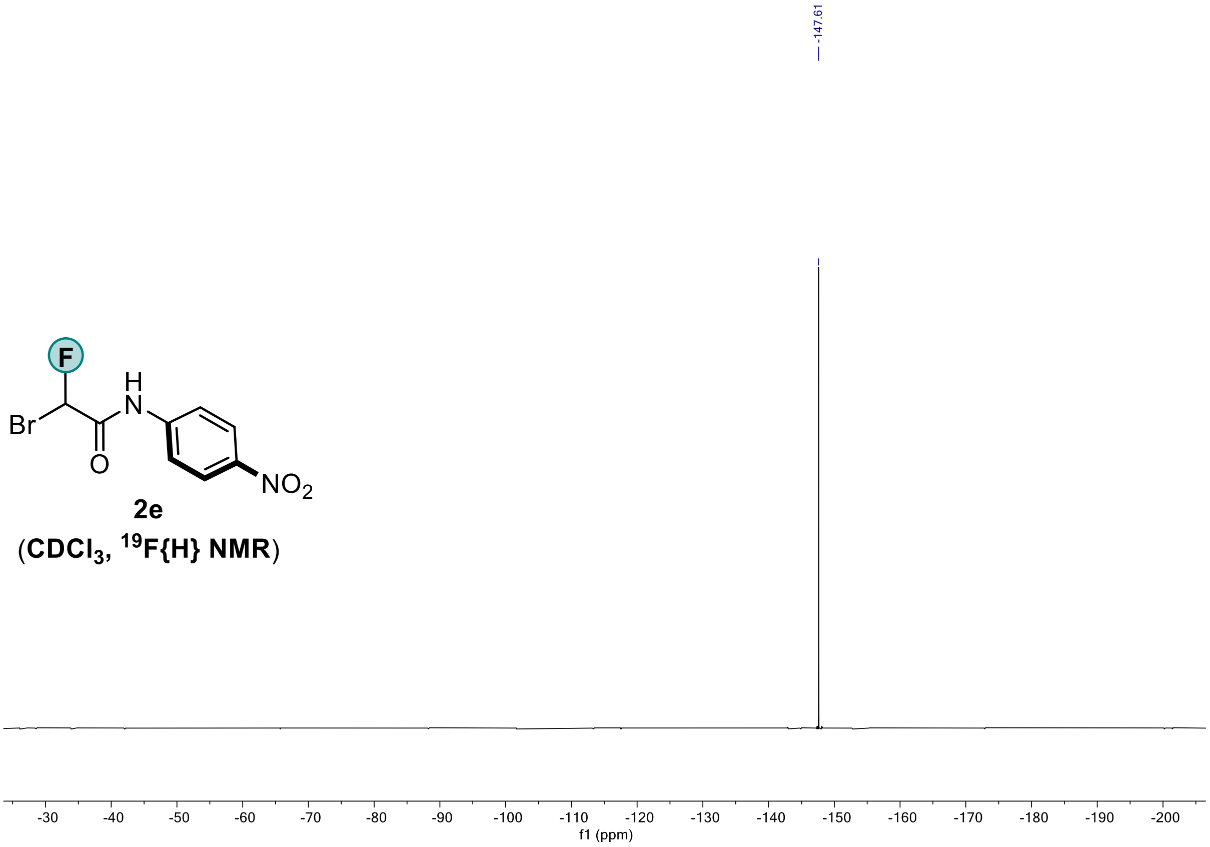
-147.61
Br
H
N
F
O
NO2
2e
(CDCl3, 19F{H} NMR)
-30 -40 -50 -60 -70 -80 -90 -100 -110 -120 -130 -140 -150 -160 -170 -180 -190 -200
f1 (ppm)

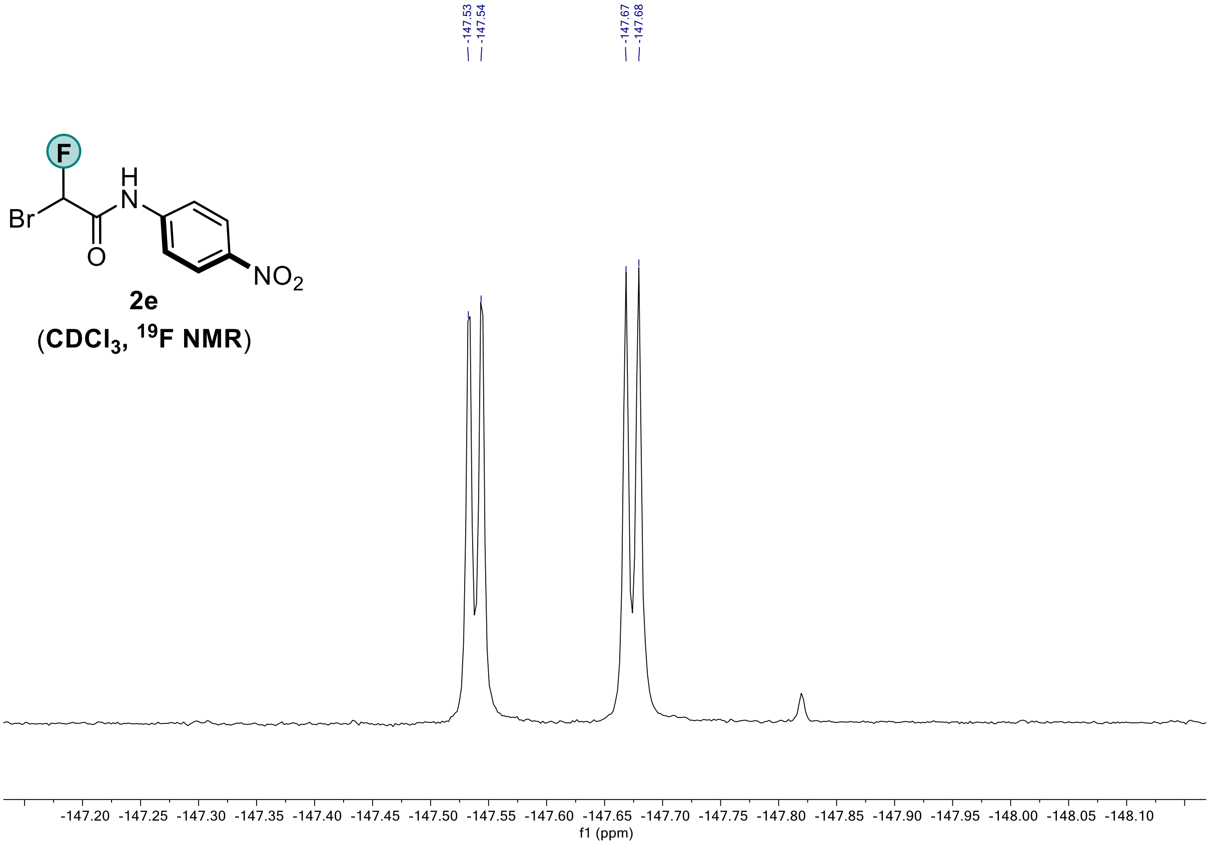
-147.53
-147.54
-147.67
-147.68
Br
H
N
F
O
NO2
2e
(CDCl3, 19F NMR)
-147.20 -147.25 -147.30 -147.35 -147.40 -147.45 -147.50 -147.55 -147.60 -147.65 -147.70 -147.75 -147.80 -147.85 -147.90 -147.95 -148.00 -148.05 -148.10
f1 (ppm)

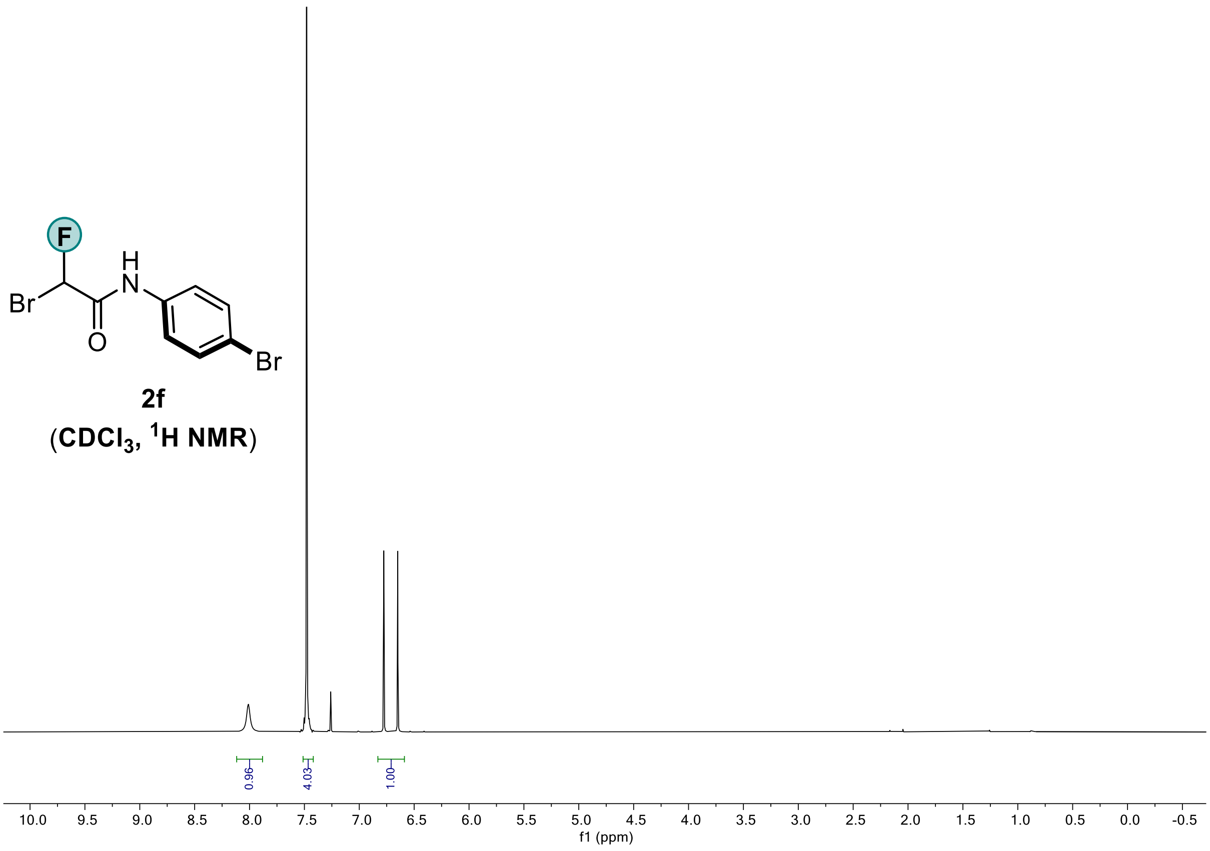
F
H
N
Br
O
Br
2f
(CDCl3, 1H NMR)
0.96
4.03
1.00
10.0 9.5 9.0 8.5 8.0 7.5 7.0 6.5 6.0 5.5 5.0 4.5 4.0 3.5 3.0 2.5 2.0 1.5 1.0 0.5 0.0 -0.5
f1 (ppm)

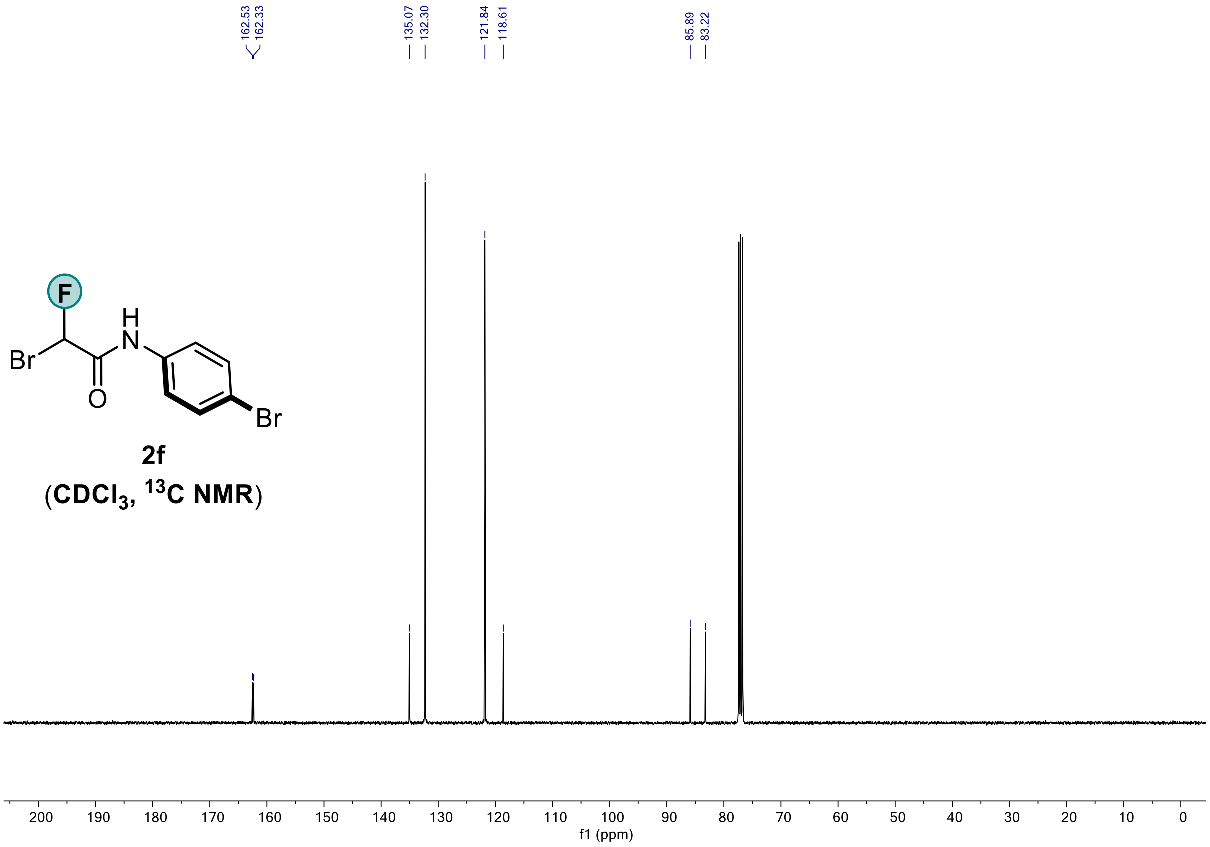
162.53
162.33
135.07
132.30
121.84
118.61
85.89
83.22
F
H
N
Br
O
Br
2f
(CDCl3, 13C NMR)
200 190 180 170 160 150 140 130 120 110 100 90 80 70 60 50 40 30 20 10 0
f1 (ppm)

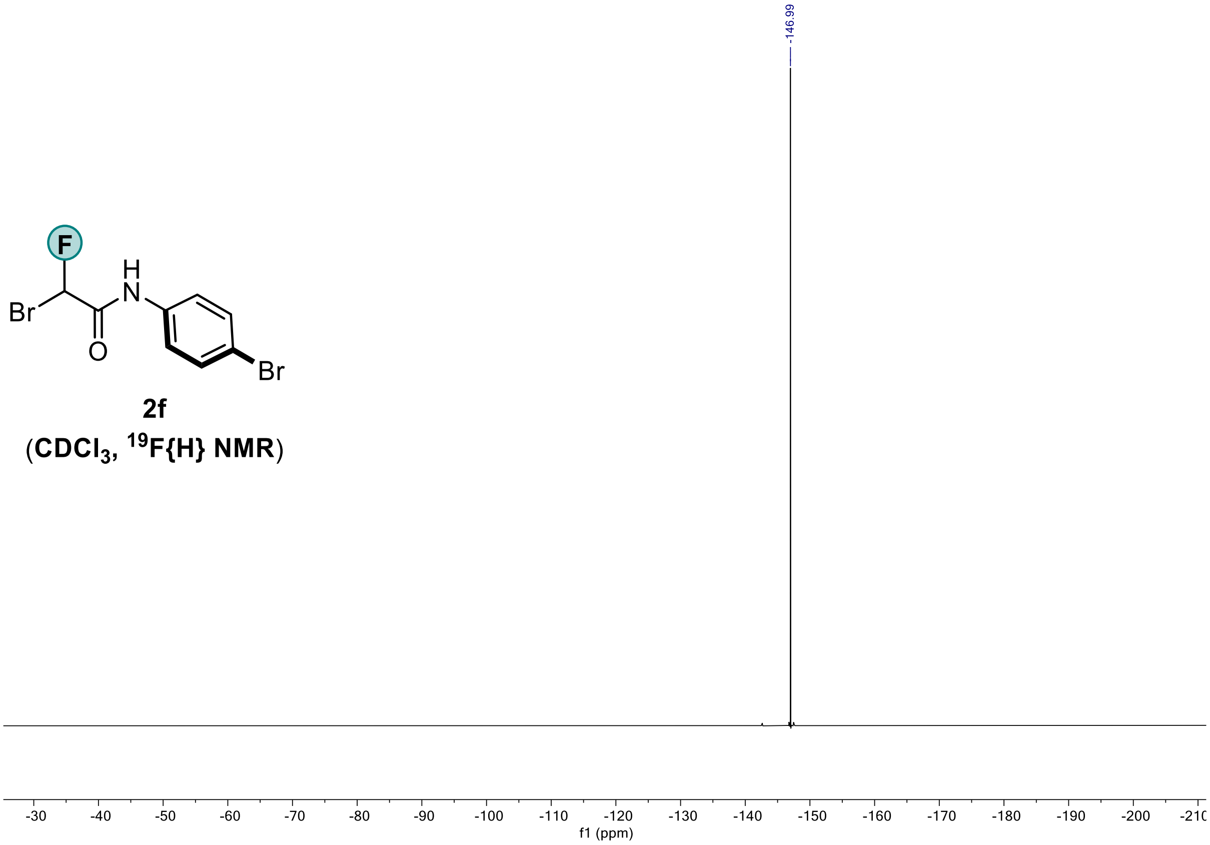
F
H
N
Br
O
Br
2f
(CDCl$_3$, ^{19}F{H} NMR)
-146.99
-30 -40 -50 -60 -70 -80 -90 -100 -110 -120 -130 -140 -150 -160 -170 -180 -190 -200 -210
f1 (ppm)

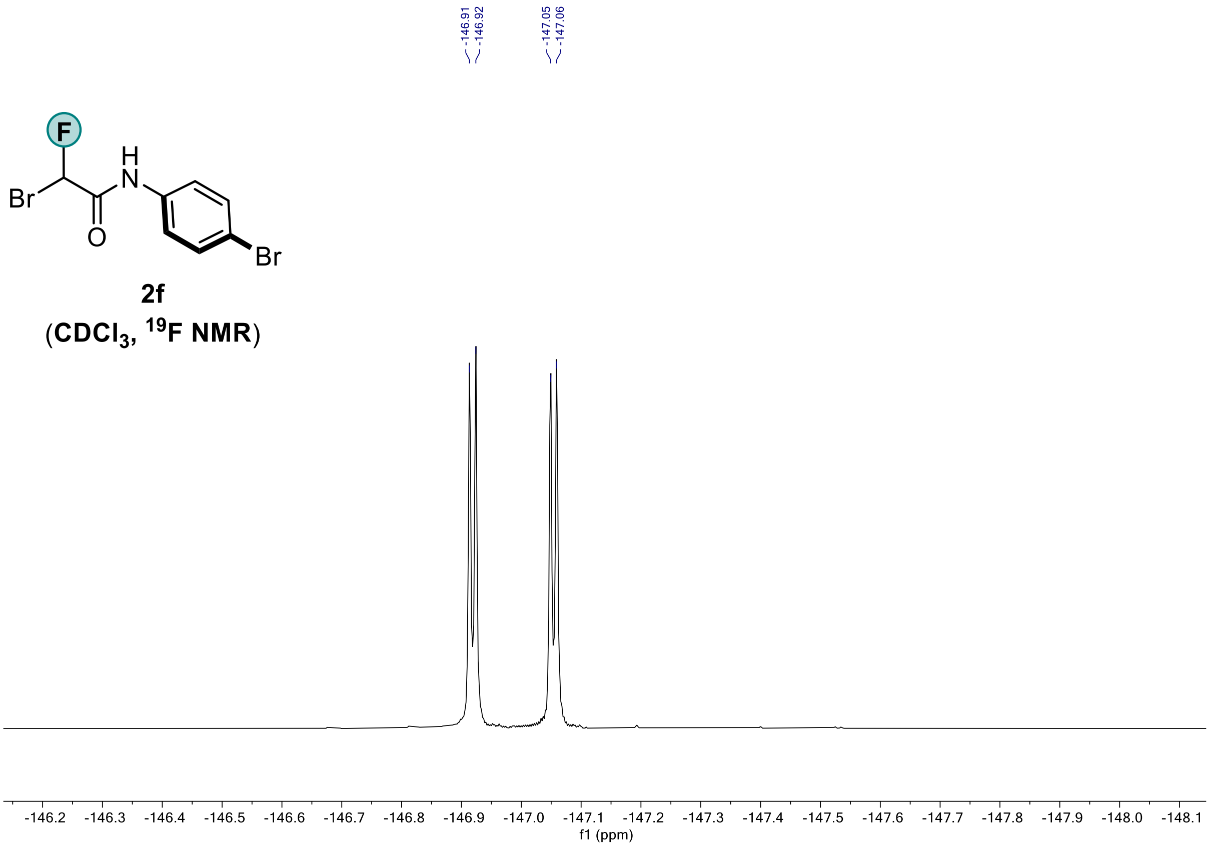
-146.91
-146.92
-147.05
-147.06
F
H
N
Br
O
Br
2f
(CDCl$_3$, ^{19}F NMR)
-146.2 -146.3 -146.4 -146.5 -146.6 -146.7 -146.8 -146.9 -147.0 -147.1 -147.2 -147.3 -147.4 -147.5 -147.6 -147.7 -147.8 -147.9 -148.0 -148.1
f1 (ppm)

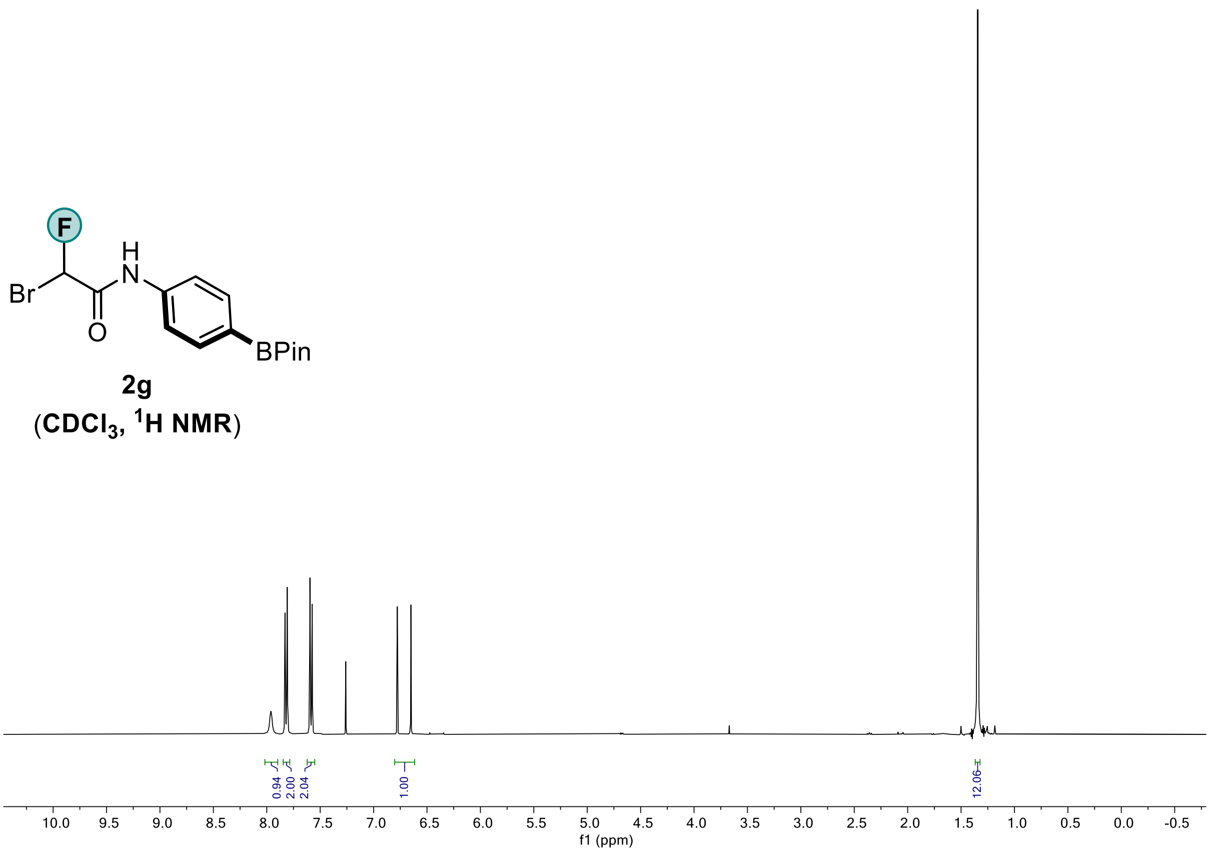

F
H
N
Br
O
BPin
2g
(CDCl3, 1H NMR)
0.94
2.00
2.04
1.00
12.06
10.0 9.5 9.0 8.5 8.0 7.5 7.0 6.5 6.0 5.5 5.0 4.5 4.0 3.5 3.0 2.5 2.0 1.5 1.0 0.5 0.0 -0.5
f1 (ppm)

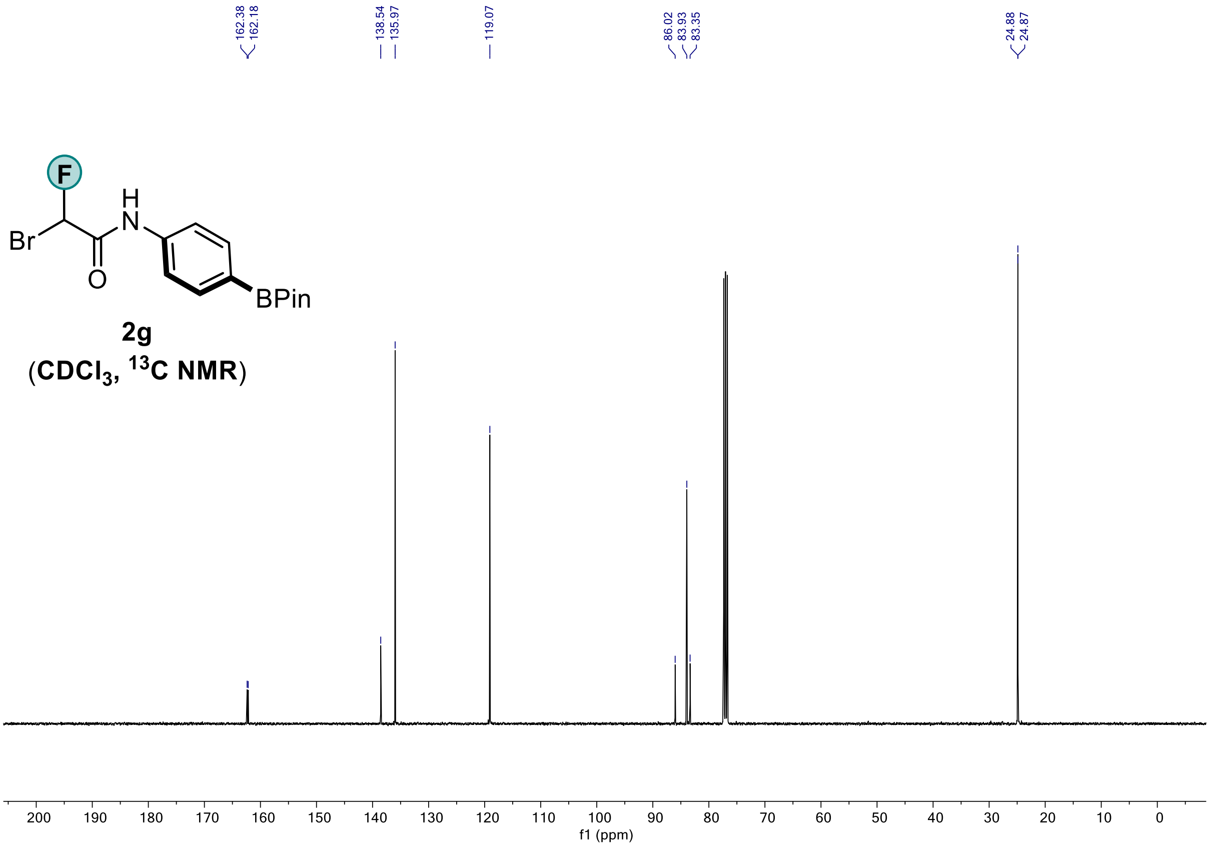

162.38
162.18
138.54
135.97
119.07
86.02
83.93
83.35
24.88
24.87
F
H
N
Br
O
BPin
2g
(CDCl3, 13C NMR)
200 190 180 170 160 150 140 130 120 110 100 90 80 70 60 50 40 30 20 10 0
f1 (ppm)

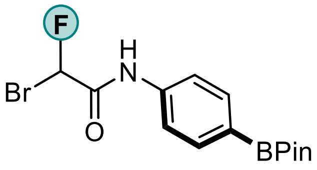

2g
(**CDCl$_3$, ^{19}F{H} NMR**)

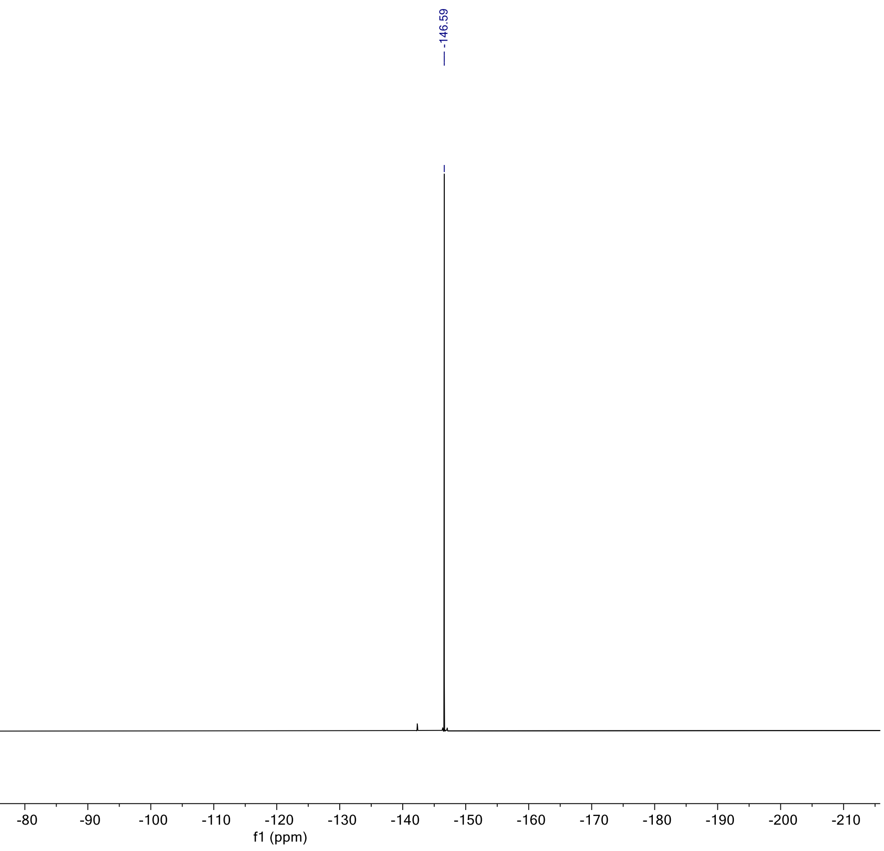

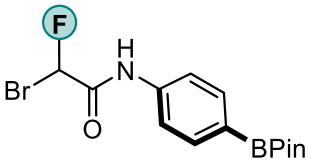

2g
(**CDCl$_3$, ^{19}F NMR**)

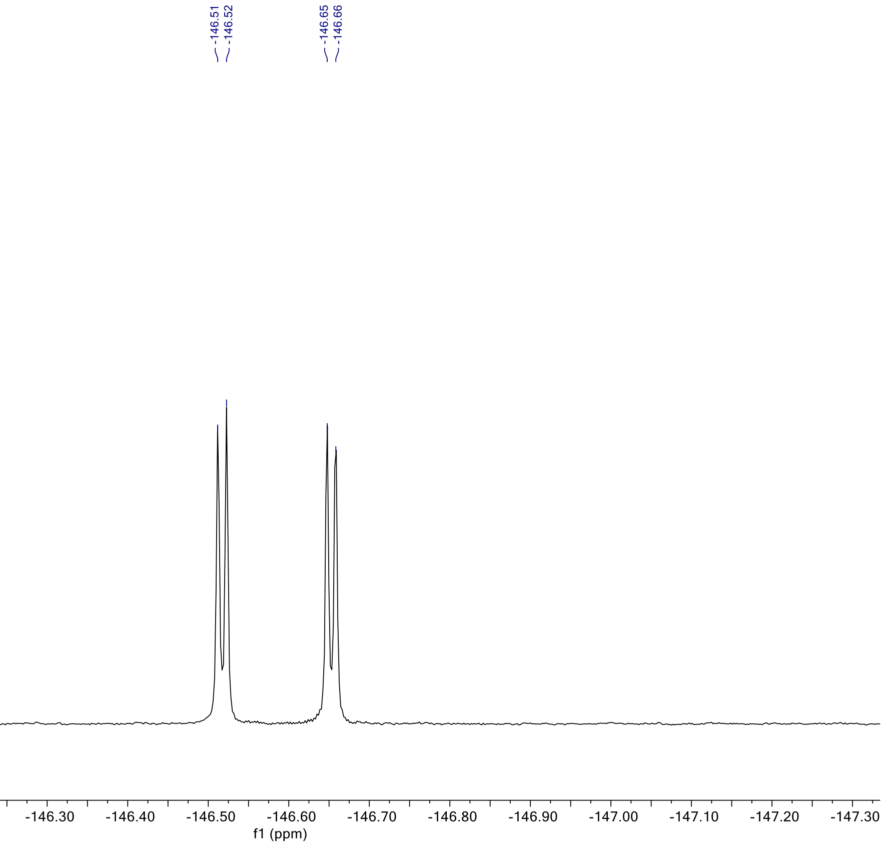

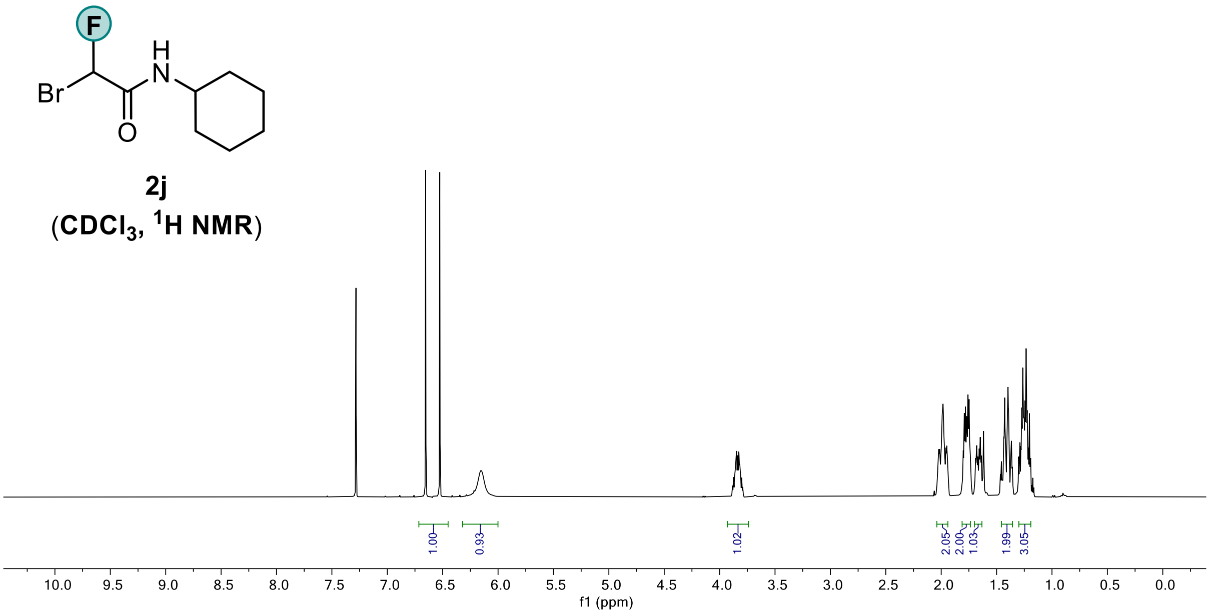

2j
(**$CDCl_3$, ^{1}H NMR**)

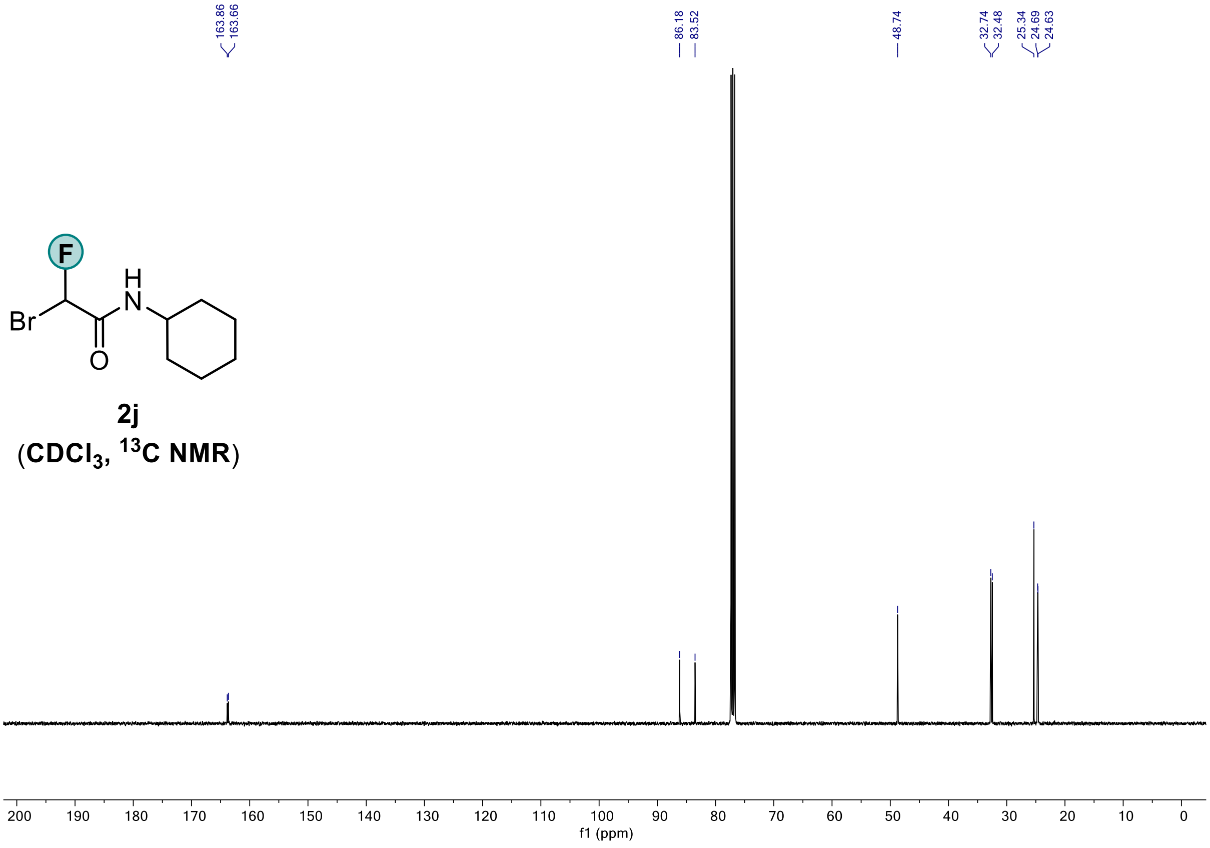

2j
(**$CDCl_3$, ^{13}C NMR**)

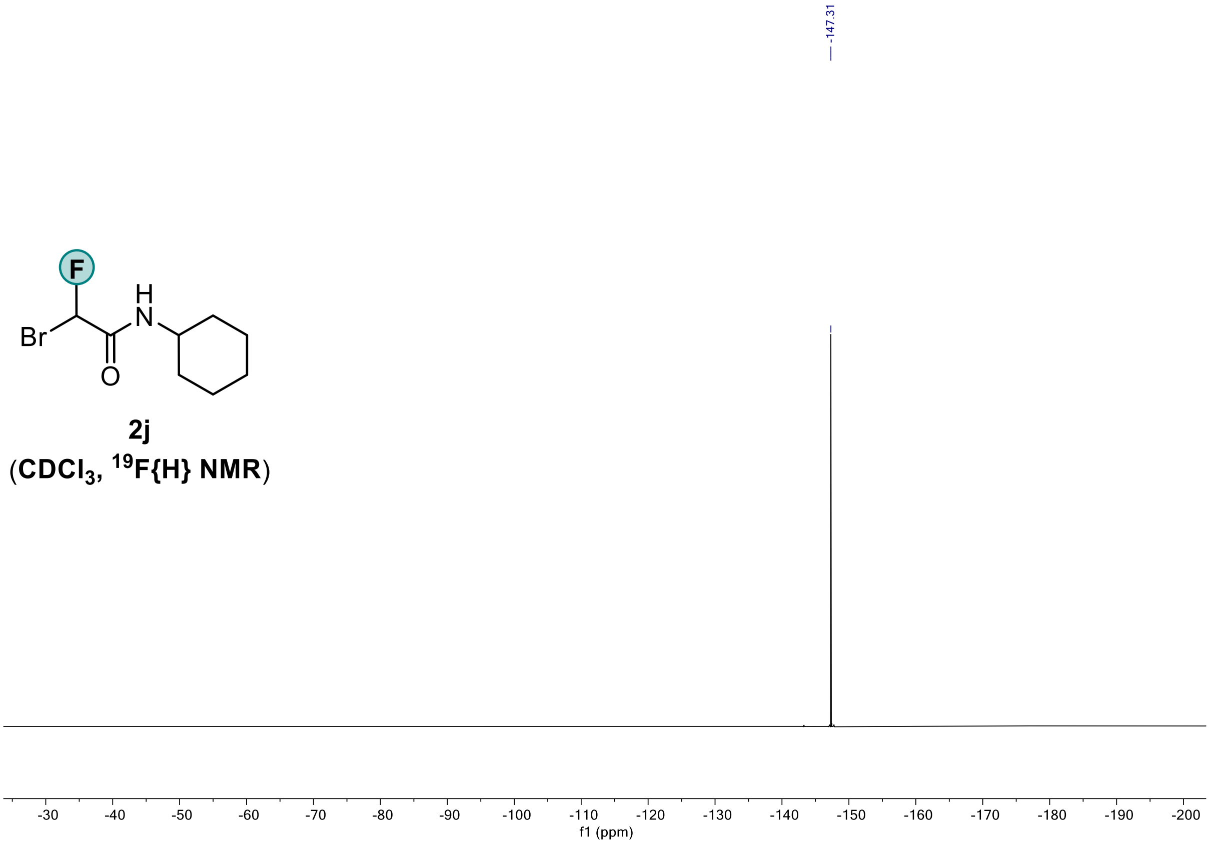
-147.31
F
Br
H
N
O
2j
(CDCl$_3$, ^{19}F{H} NMR)
-30 -40 -50 -60 -70 -80 -90 -100 -110 -120 -130 -140 -150 -160 -170 -180 -190 -200
f1 (ppm)

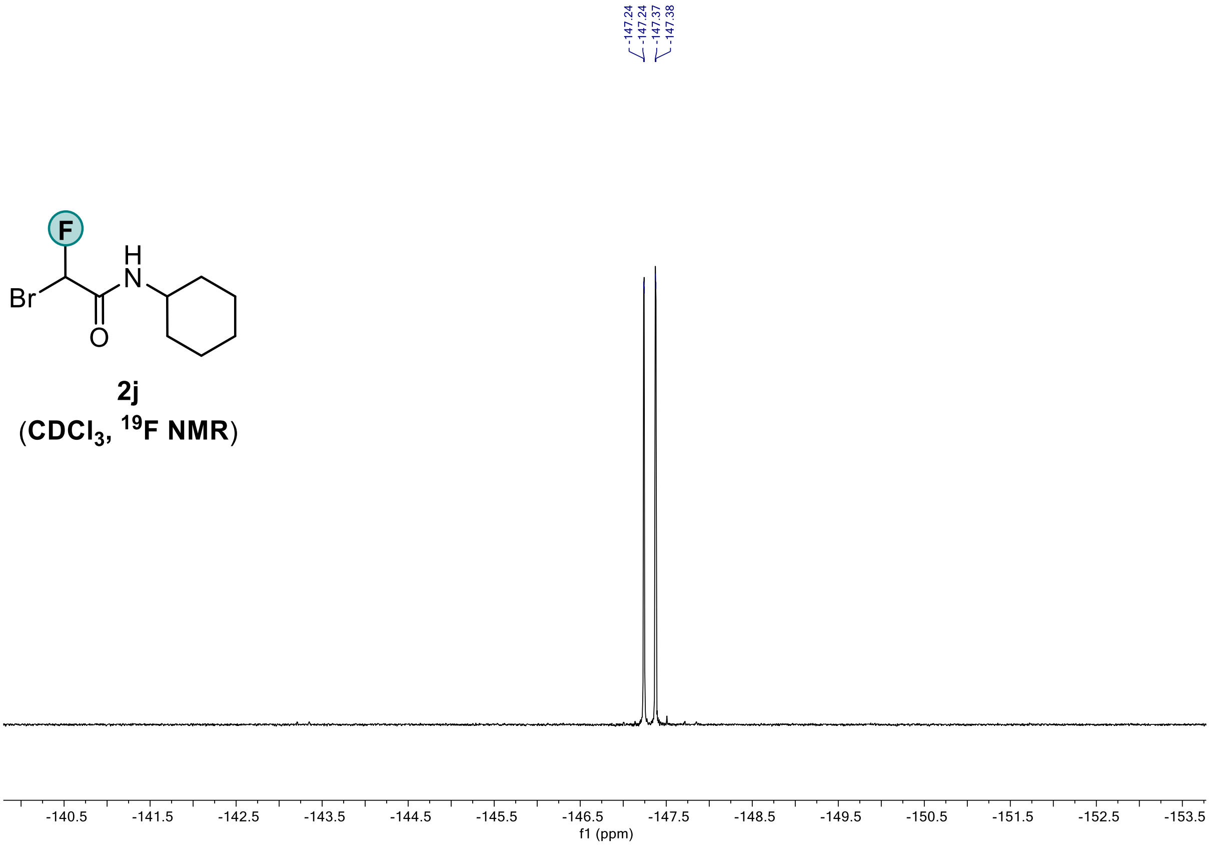
-147.24
-147.24
-147.37
-147.38
F
Br
H
N
O
2j
(CDCl$_3$, ^{19}F NMR)
-140.5 -141.5 -142.5 -143.5 -144.5 -145.5 -146.5 -147.5 -148.5 -149.5 -150.5 -151.5 -152.5 -153.5
f1 (ppm)

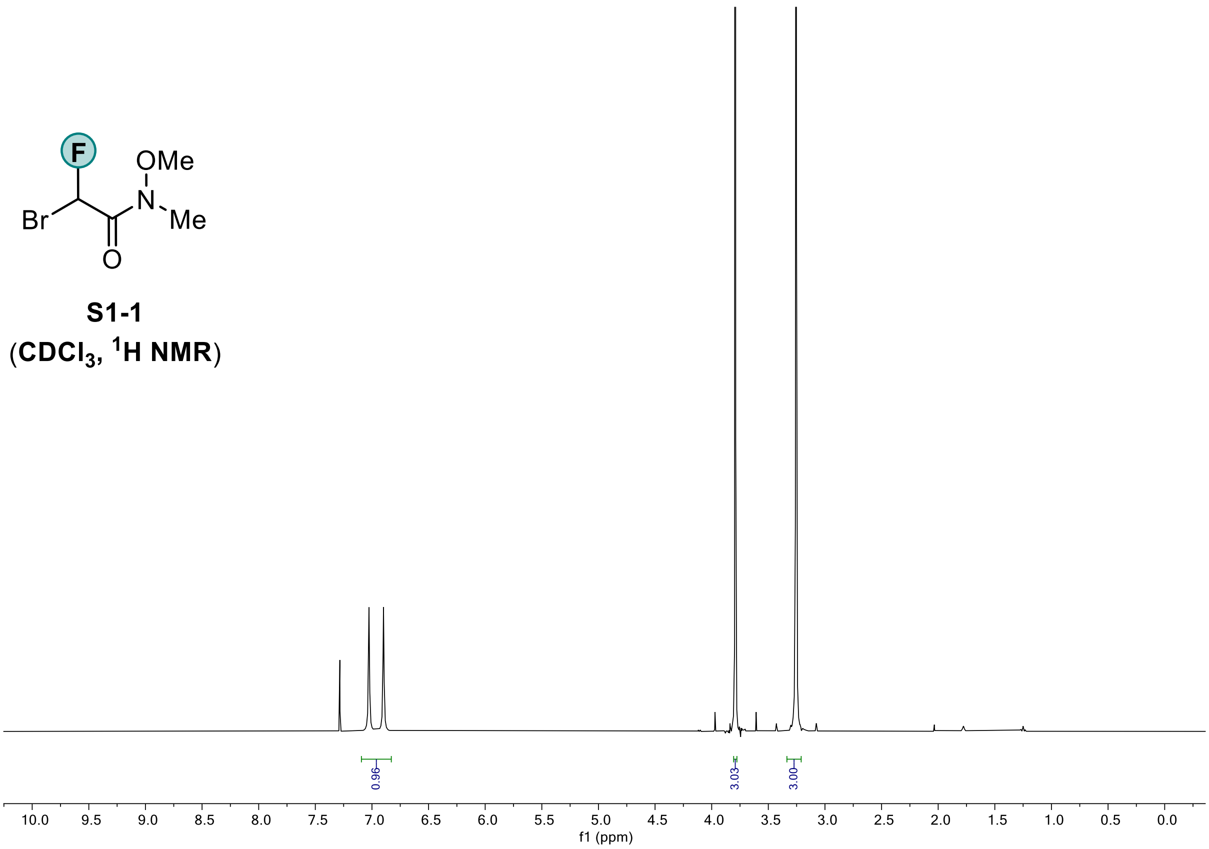

S1-1
(CDCl$_3$, ^{1}H NMR)

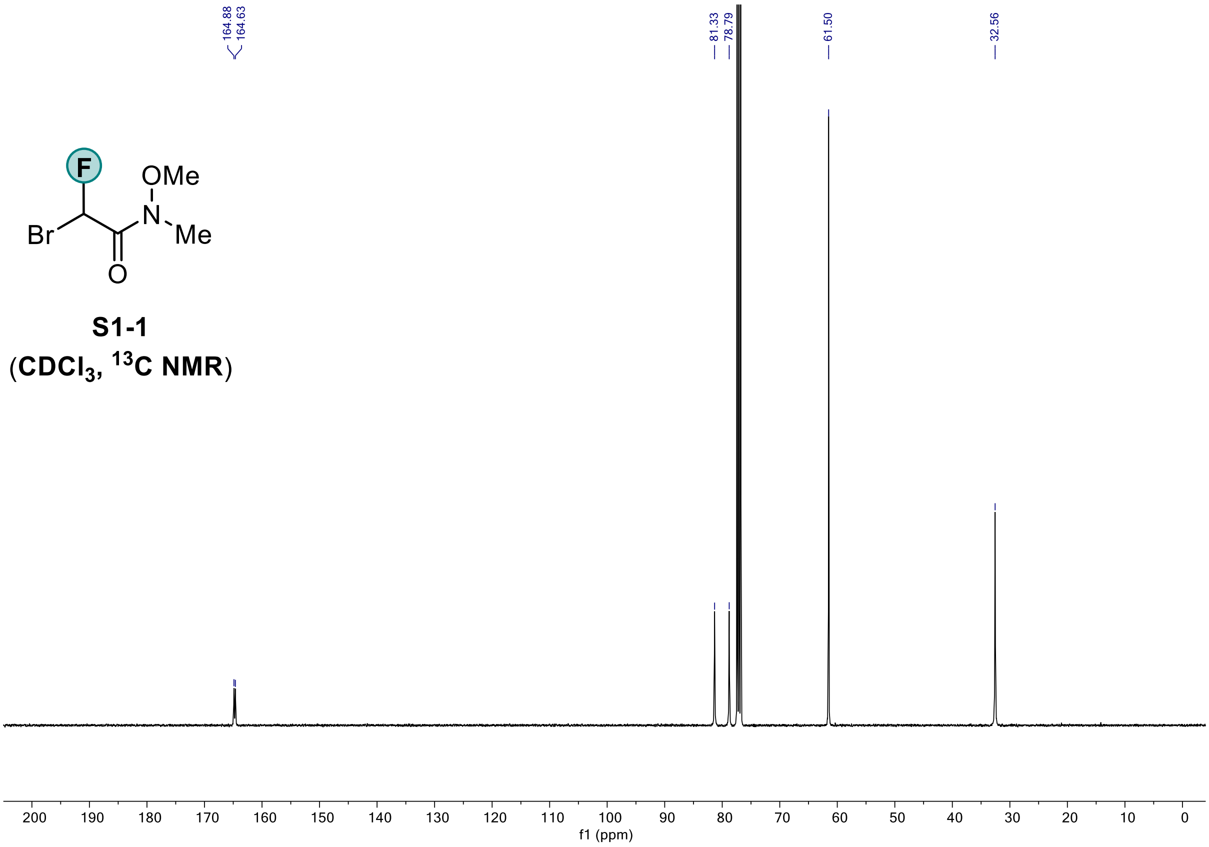

S1-1
(CDCl$_3$, ^{13}C NMR)

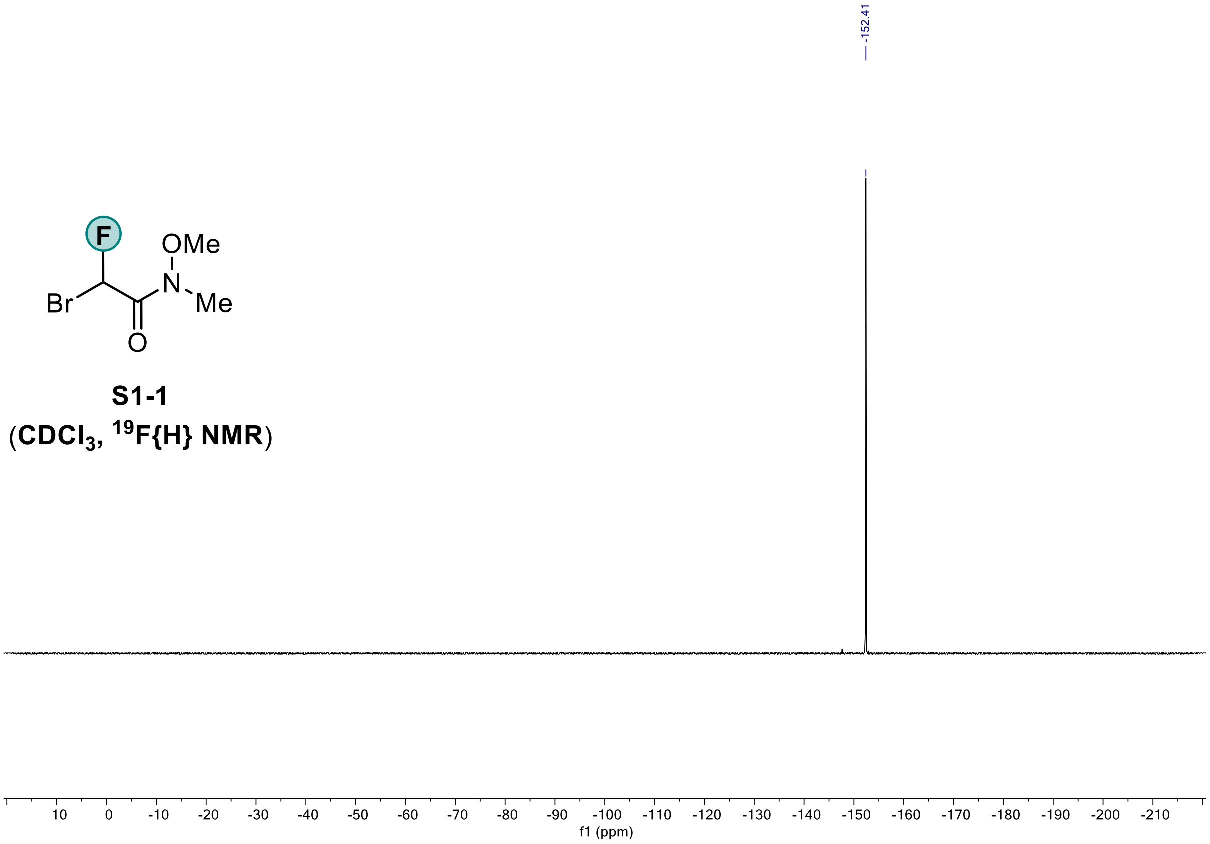

S1-1
(**$CDCl_3$, $^{19}F\{H\}$ NMR**)

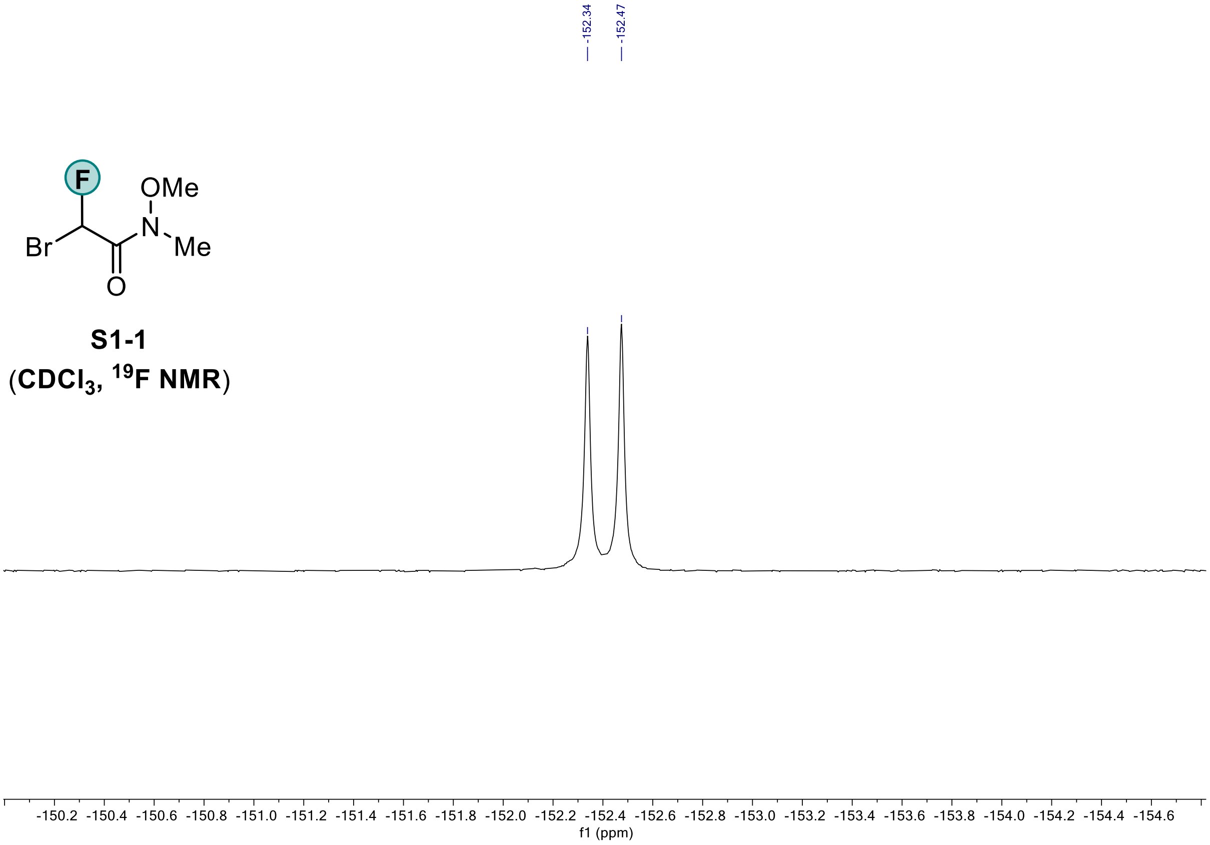

S1-1
(**$CDCl_3$, ^{19}F NMR**)

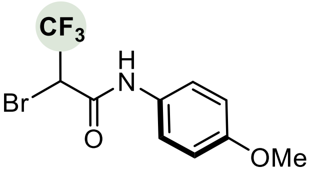

6a
(**CDCl$_3$, ^{1}H NMR**)

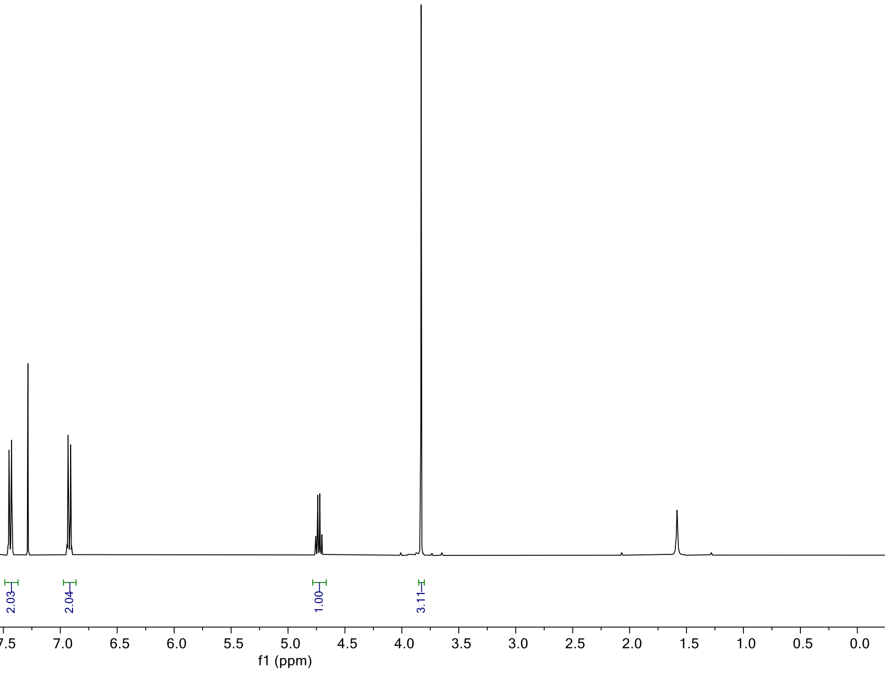

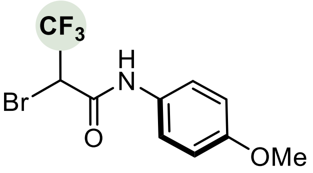

6a
(**CDCl$_3$, ^{13}C NMR**)

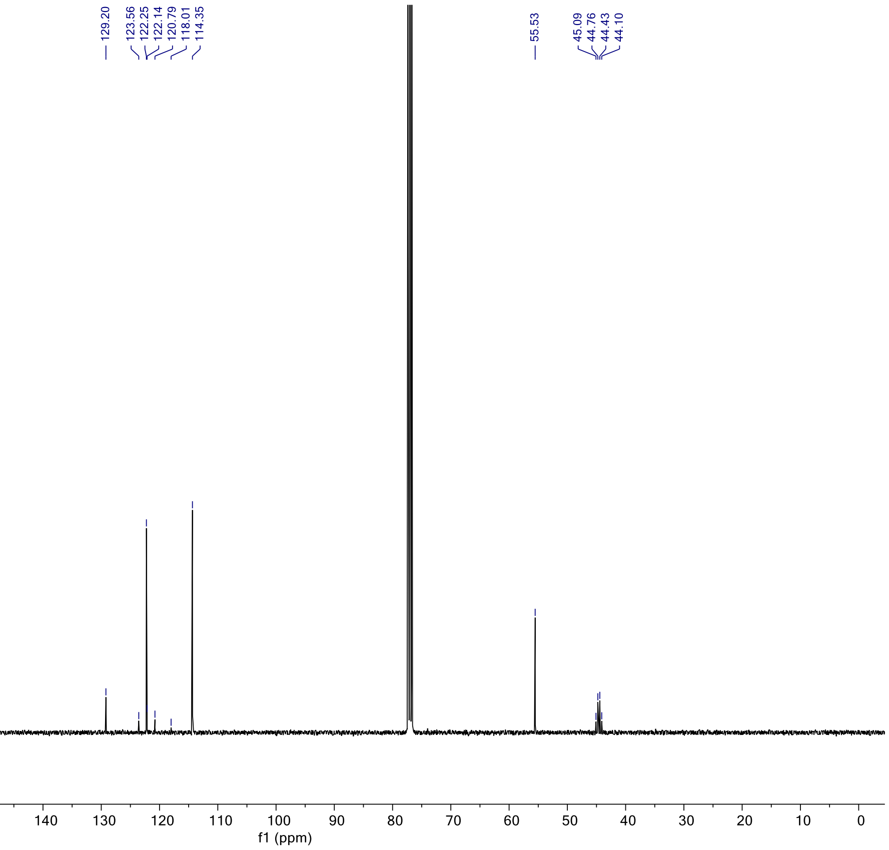

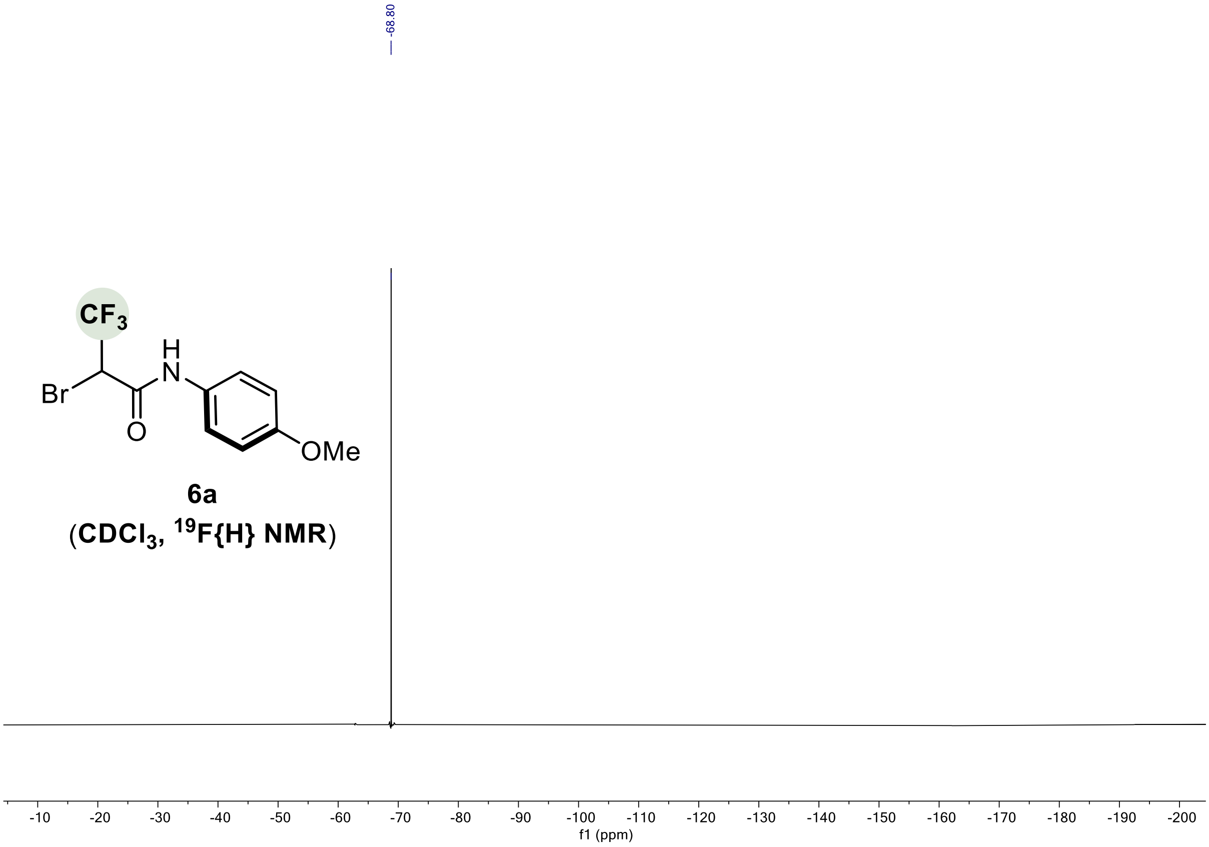

6a
(**$CDCl_3$, $^{19}F\{H\}$ NMR**)

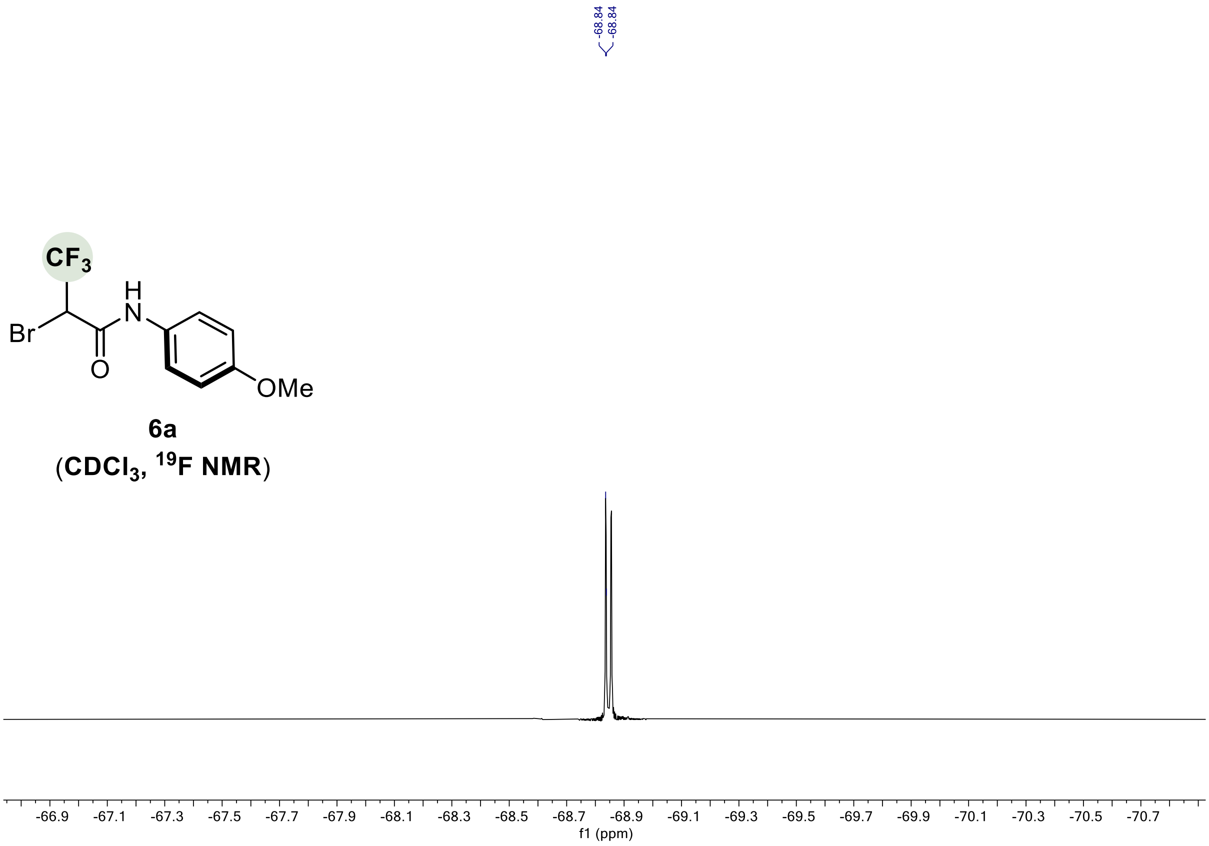

6a
(**$CDCl_3$, ^{19}F NMR**)

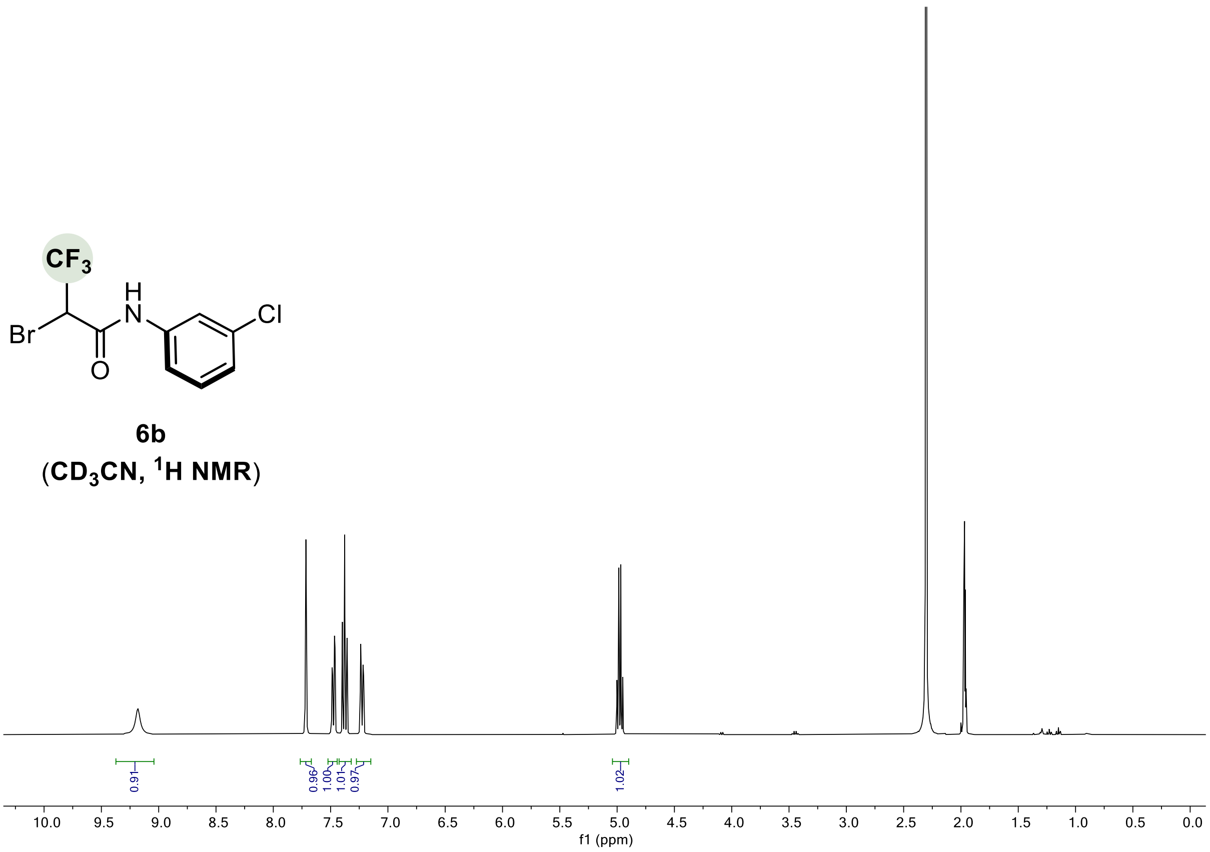
CF3
H
N
Br
O
Cl
6b
(CD3CN, 1H NMR)
0.91
0.96
1.00
1.01
0.97
1.02
10.0 9.5 9.0 8.5 8.0 7.5 7.0 6.5 6.0 5.5 5.0 4.5 4.0 3.5 3.0 2.5 2.0 1.5 1.0 0.5 0.0
f1 (ppm)

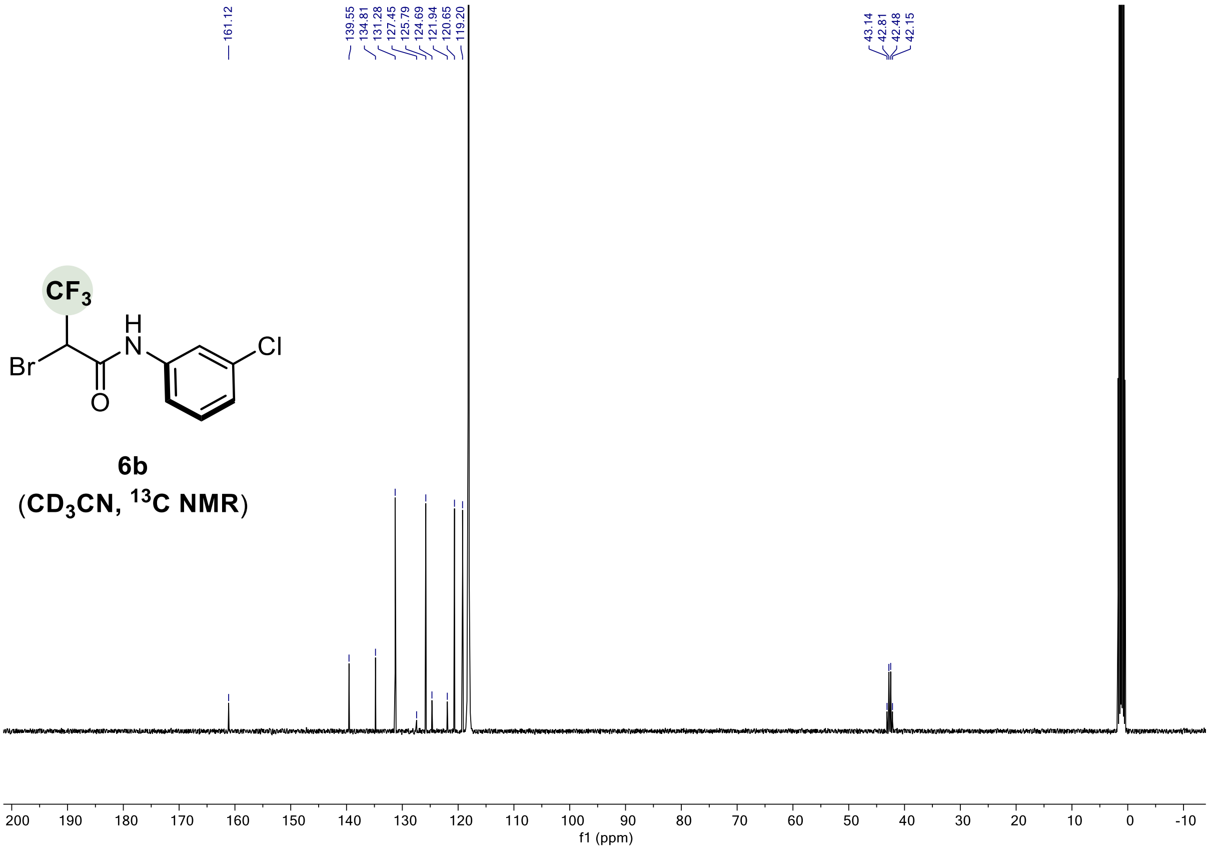
161.12
139.55
134.81
131.28
127.45
125.79
124.69
121.94
120.65
119.20
43.14
42.81
42.48
42.15
CF3
H
N
Br
O
Cl
6b
(CD3CN, 13C NMR)
200 190 180 170 160 150 140 130 120 110 100 90 80 70 60 50 40 30 20 10 0 -10
f1 (ppm)

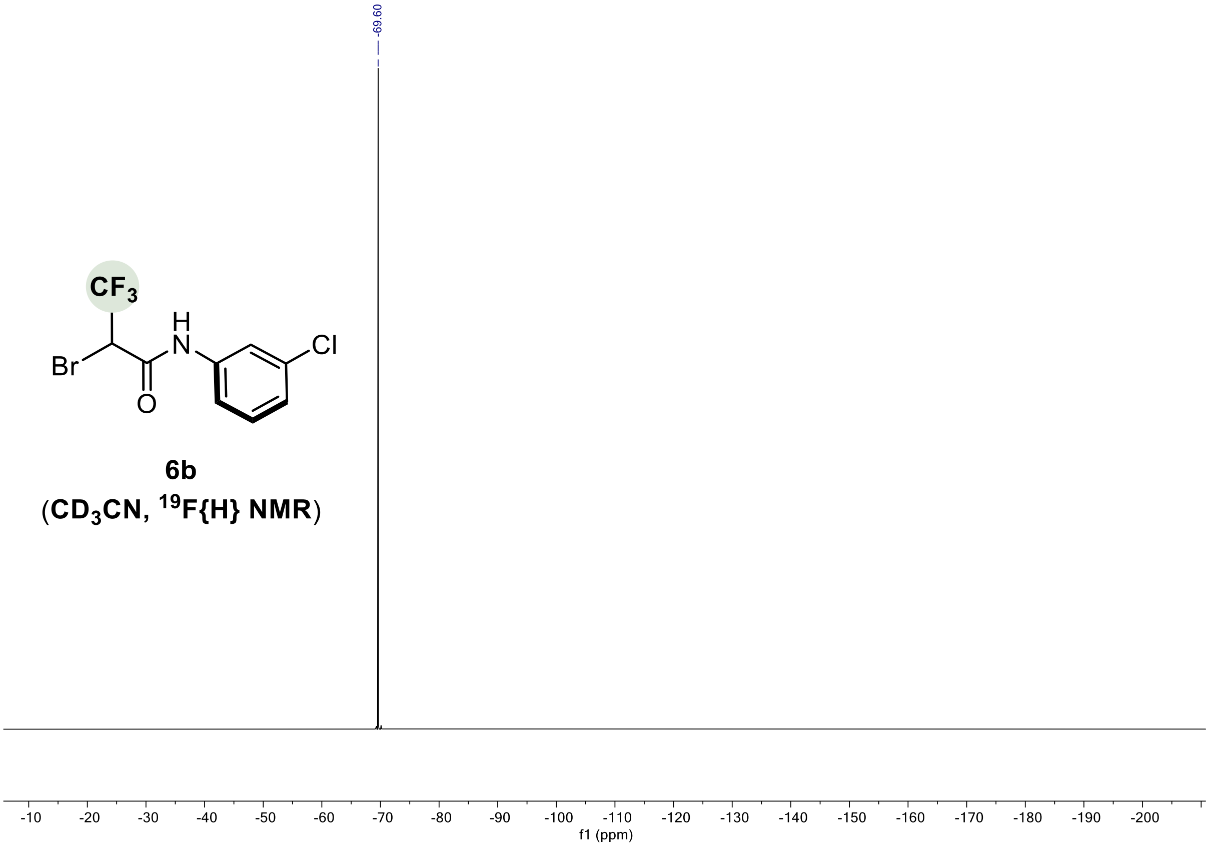

6b
(**CD_3CN, $^{19}F\{H\}$ NMR**)

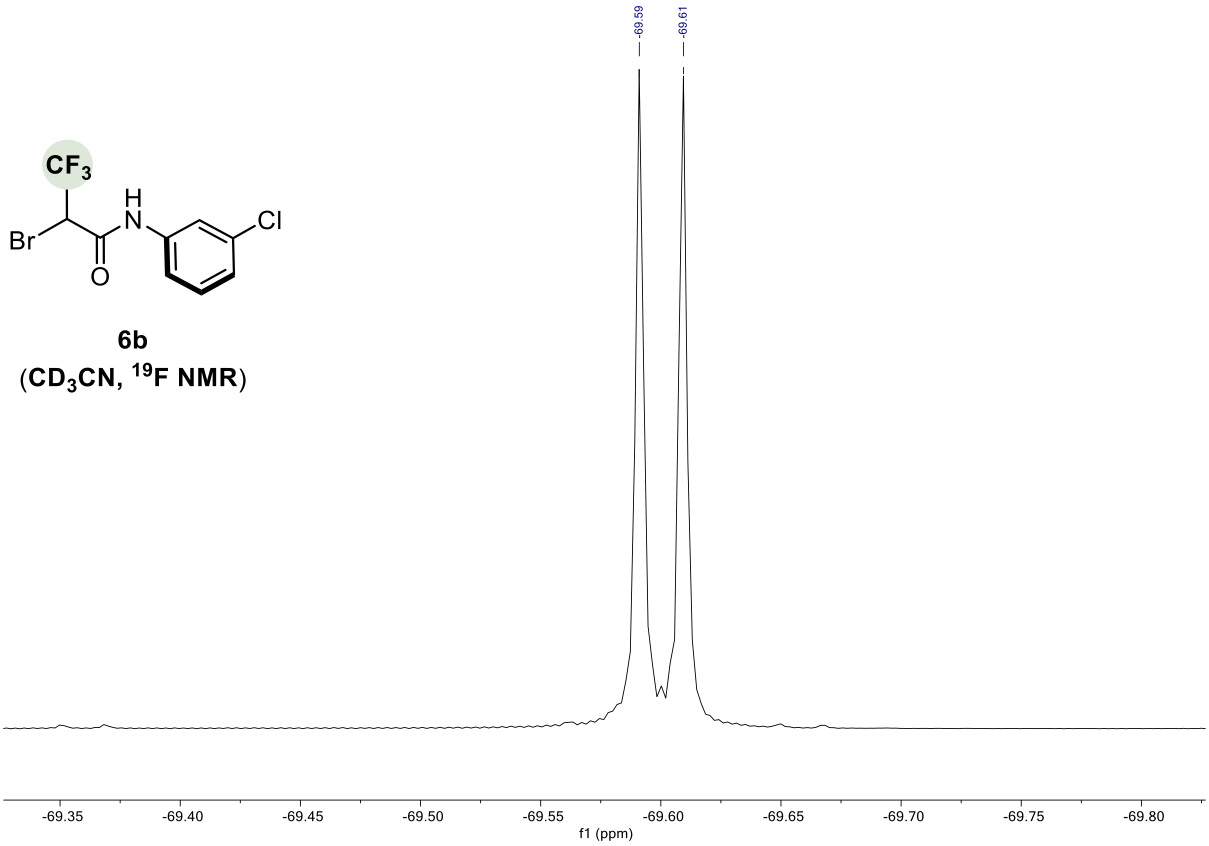

6b
(**CD_3CN, ^{19}F NMR**)

6c
(CD_3CN, ^1H NMR)

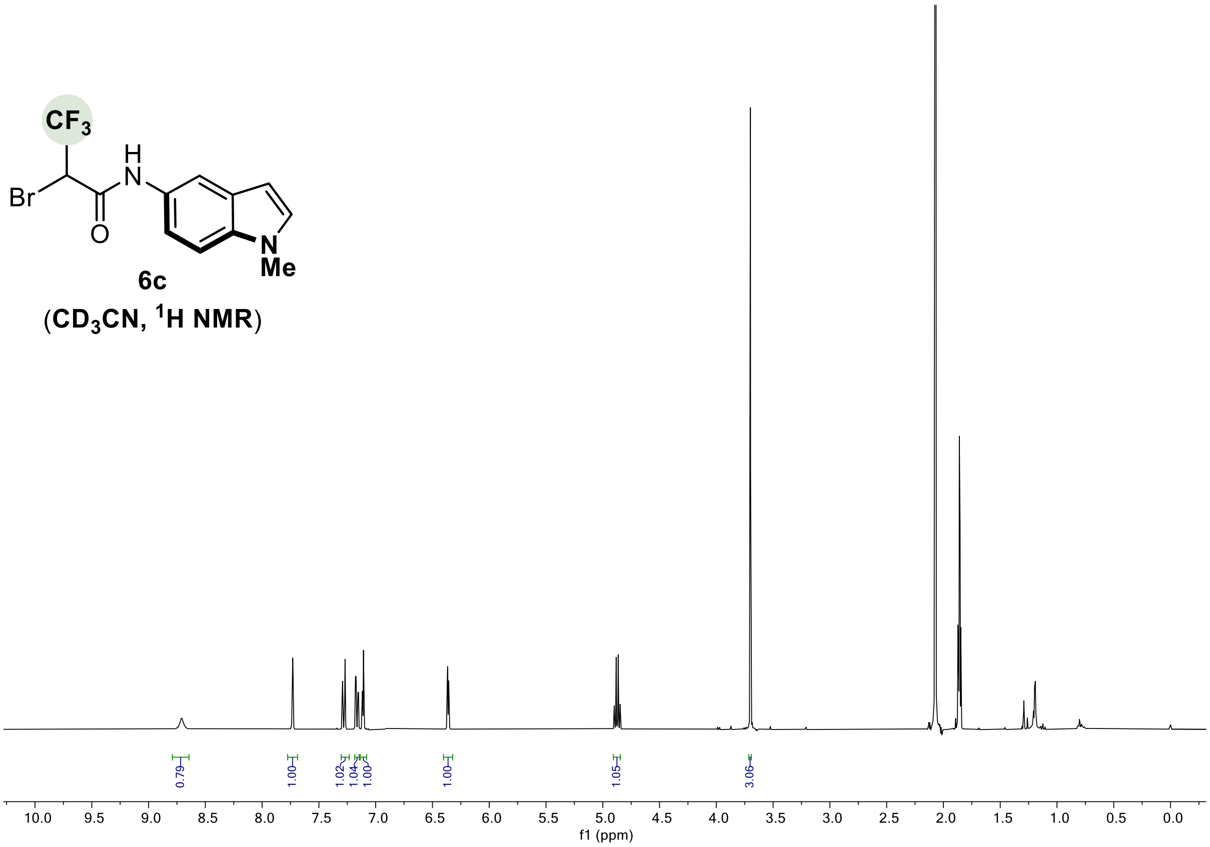

6c
(CD_3CN, ^{13}C NMR)

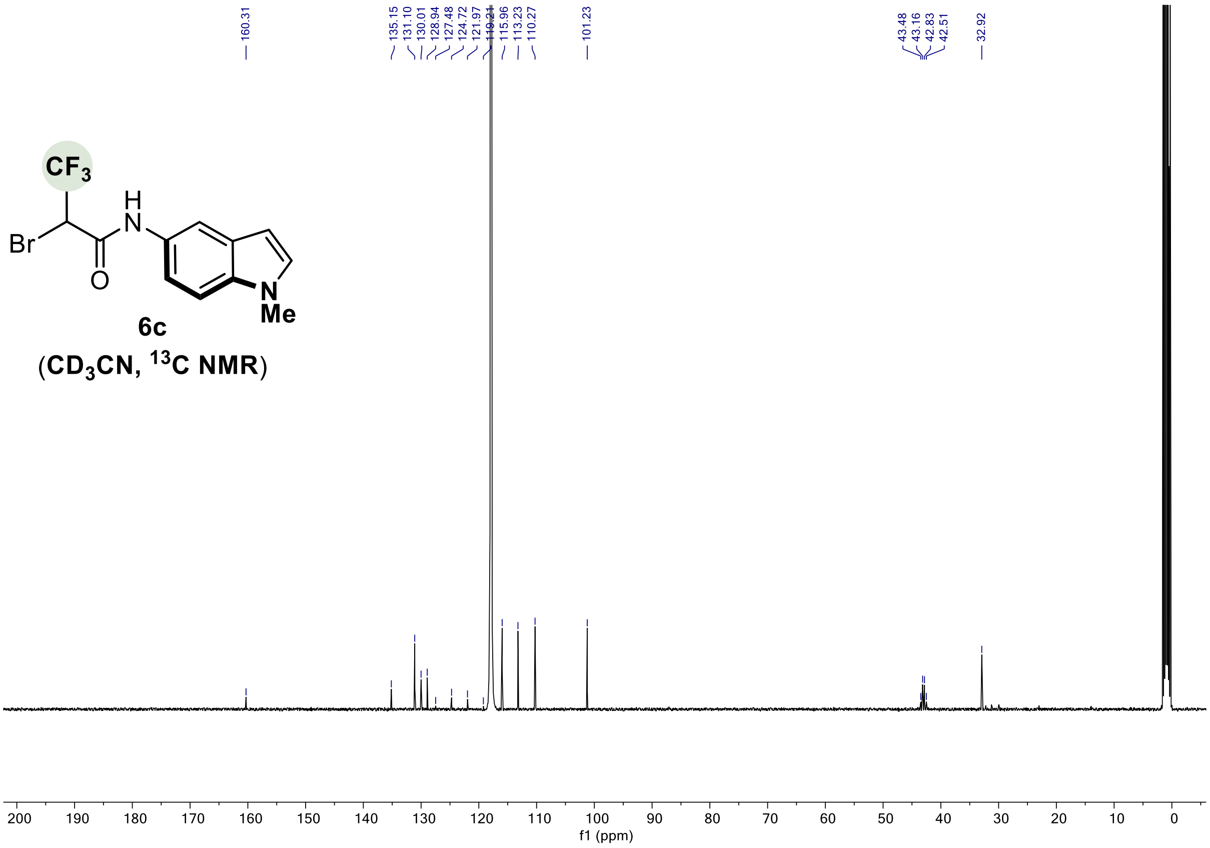

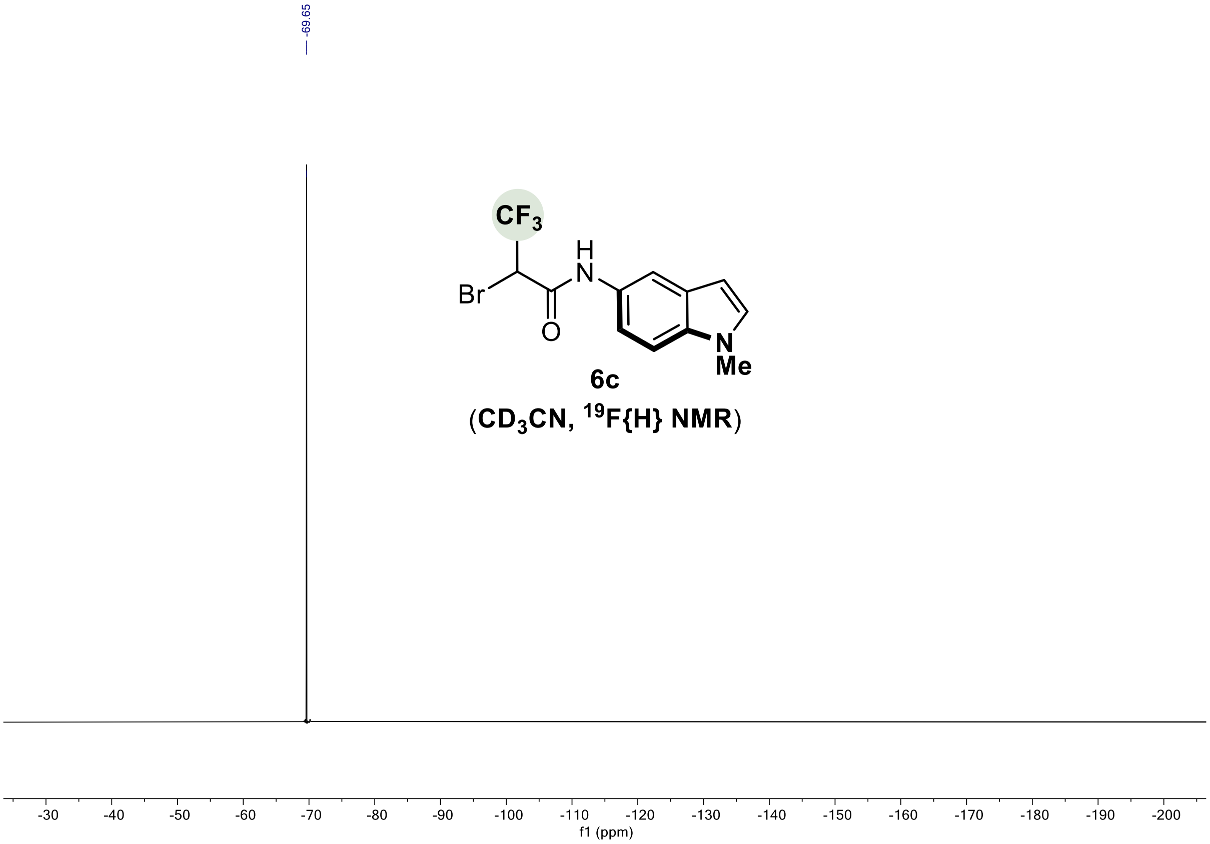
-69.65
CF3
H
N
Br
O
N
Me
6c
(CD3CN, 19F{H} NMR)
-30 -40 -50 -60 -70 -80 -90 -100 -110 -120 -130 -140 -150 -160 -170 -180 -190 -200
f1 (ppm)

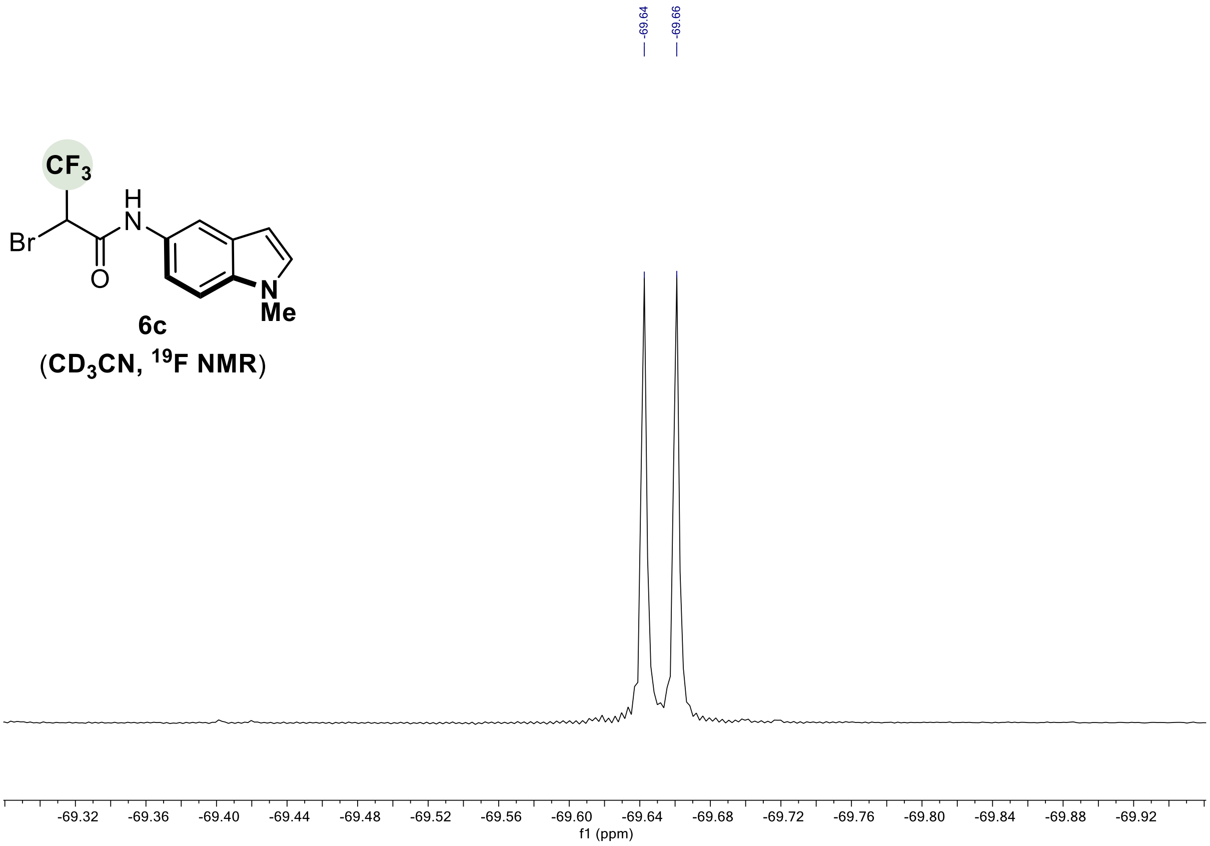
-69.64
-69.66
CF3
H
N
Br
O
N
Me
6c
(CD3CN, 19F NMR)
-69.32 -69.36 -69.40 -69.44 -69.48 -69.52 -69.56 -69.60 -69.64 -69.68 -69.72 -69.76 -69.80 -69.84 -69.88 -69.92
f1 (ppm)

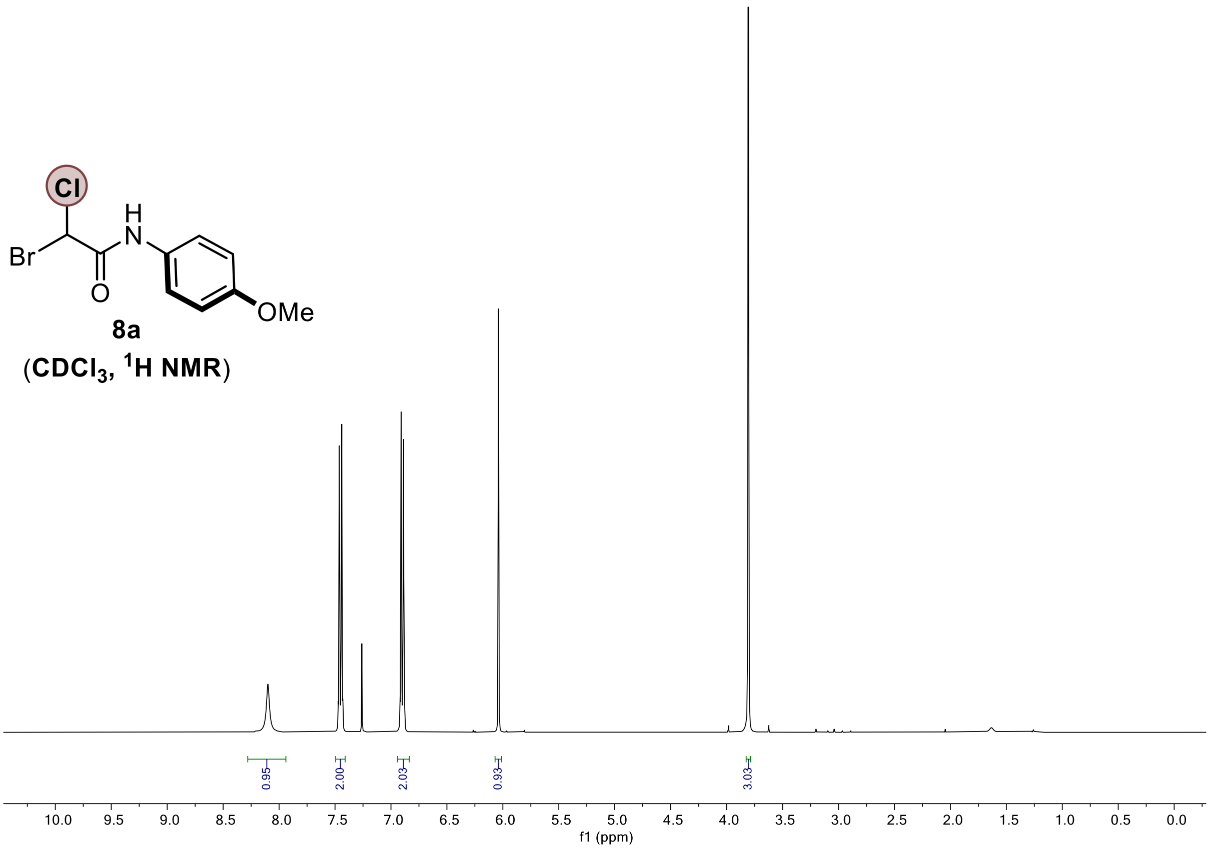
Cl
H
N
Br
O
OMe
8a
(CDCl$_3$, ^{1}H NMR)
0.95
2.00
2.03
0.93
3.03
10.0 9.5 9.0 8.5 8.0 7.5 7.0 6.5 6.0 5.5 5.0 4.5 4.0 3.5 3.0 2.5 2.0 1.5 1.0 0.5 0.0
f1 (ppm)

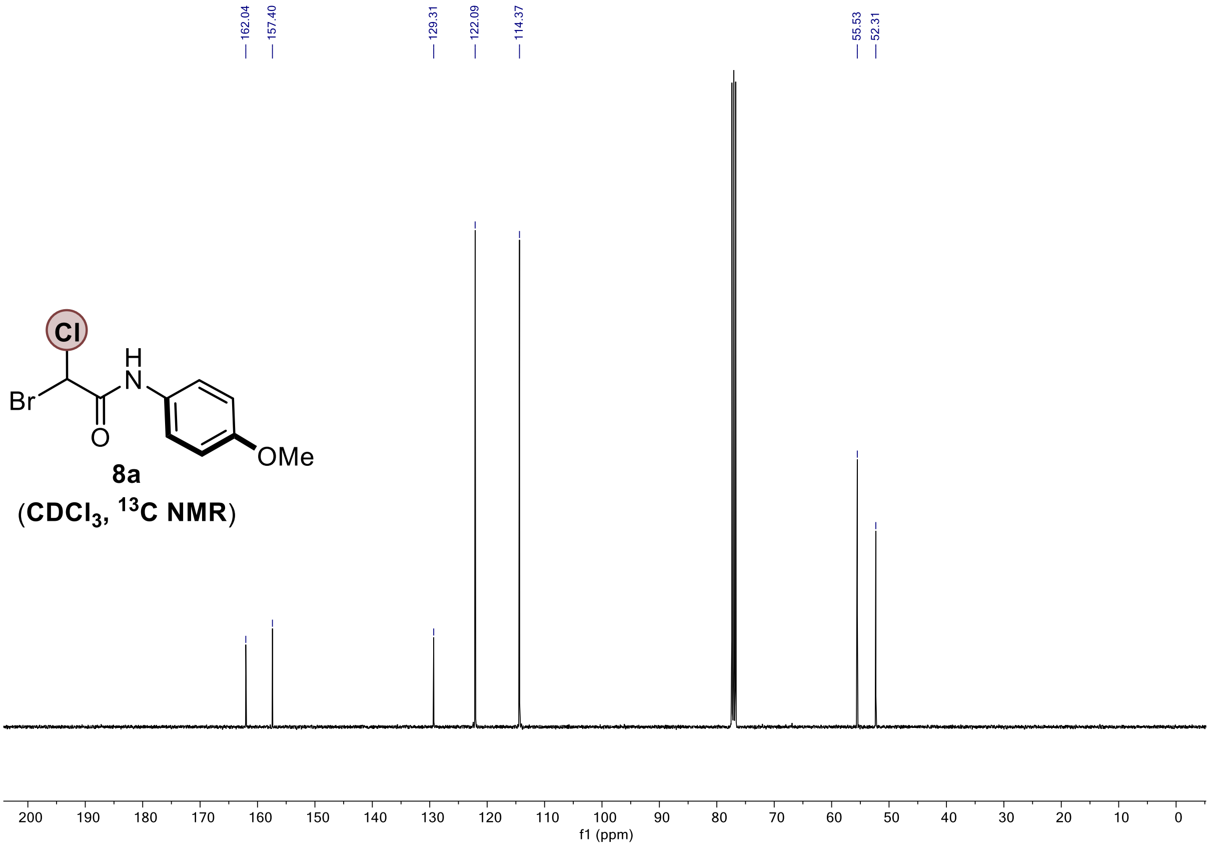
162.04
157.40
129.31
122.09
114.37
55.53
52.31
Cl
H
N
Br
O
OMe
8a
(CDCl$_3$, ^{13}C NMR)
200 190 180 170 160 150 140 130 120 110 100 90 80 70 60 50 40 30 20 10 0
f1 (ppm)

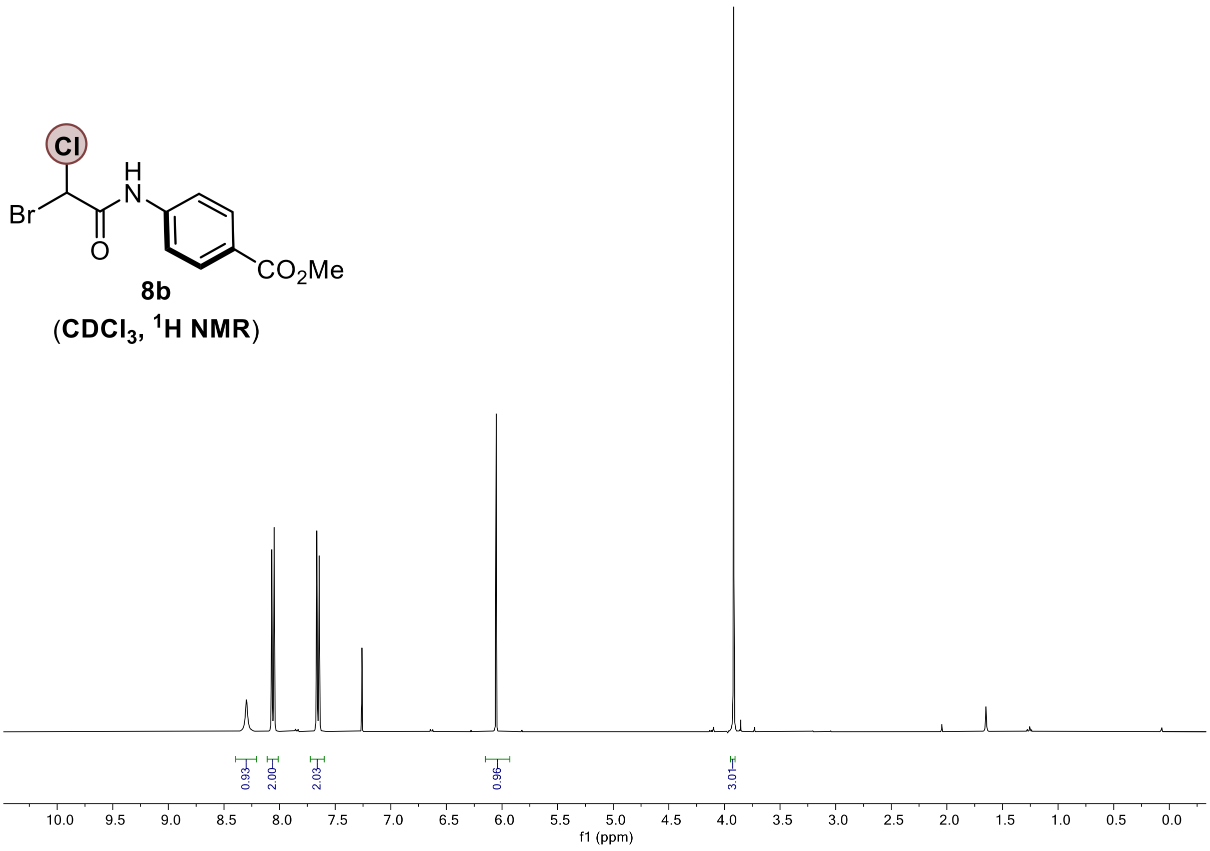
Cl
H
N
Br
O
CO2Me
8b
(CDCl3, 1H NMR)
0.93
2.00
2.03
0.96
3.01
10.0 9.5 9.0 8.5 8.0 7.5 7.0 6.5 6.0 5.5 5.0 4.5 4.0 3.5 3.0 2.5 2.0 1.5 1.0 0.5 0.0
f1 (ppm)

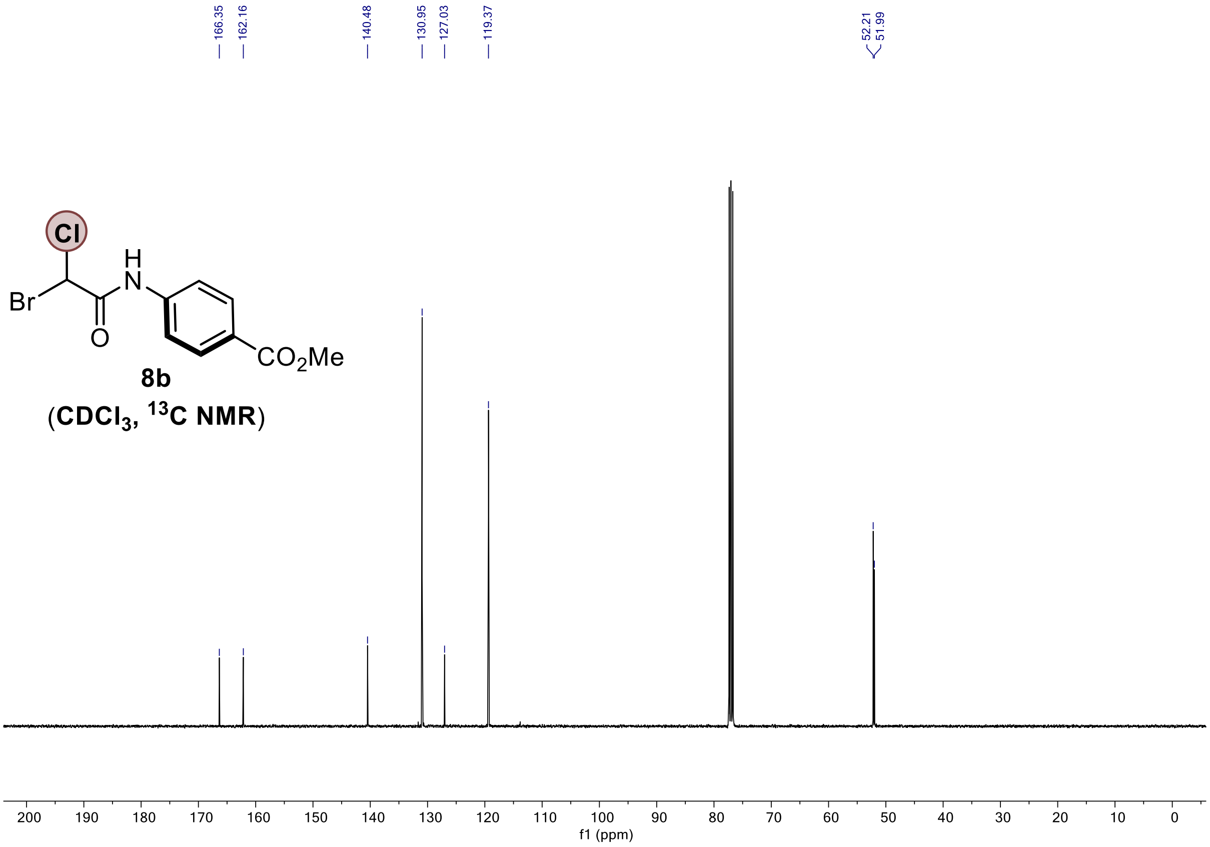
166.35
162.16
140.48
130.95
127.03
119.37
52.21
51.99
Cl
H
N
Br
O
CO2Me
8b
(CDCl3, 13C NMR)
200 190 180 170 160 150 140 130 120 110 100 90 80 70 60 50 40 30 20 10 0
f1 (ppm)

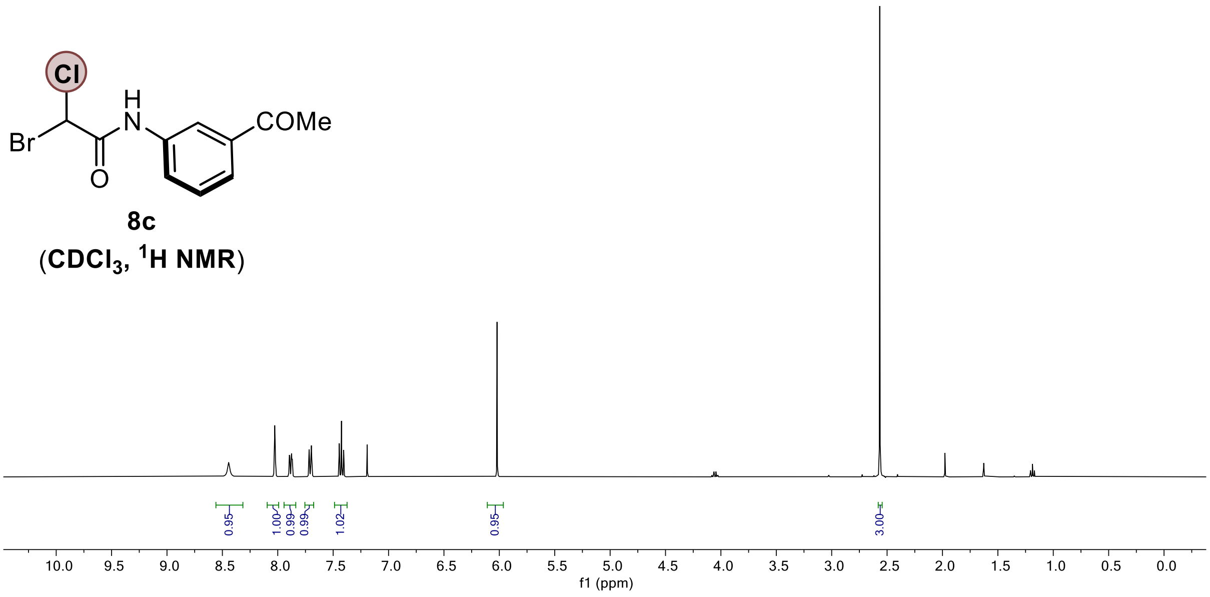
Cl
H
N
COMe
Br
O
8c
(CDCl3, 1H NMR)
0.95
1.00
0.99
0.99
1.02
0.95
3.00
10.0 9.5 9.0 8.5 8.0 7.5 7.0 6.5 6.0 5.5 5.0 4.5 4.0 3.5 3.0 2.5 2.0 1.5 1.0 0.5 0.0
f1 (ppm)

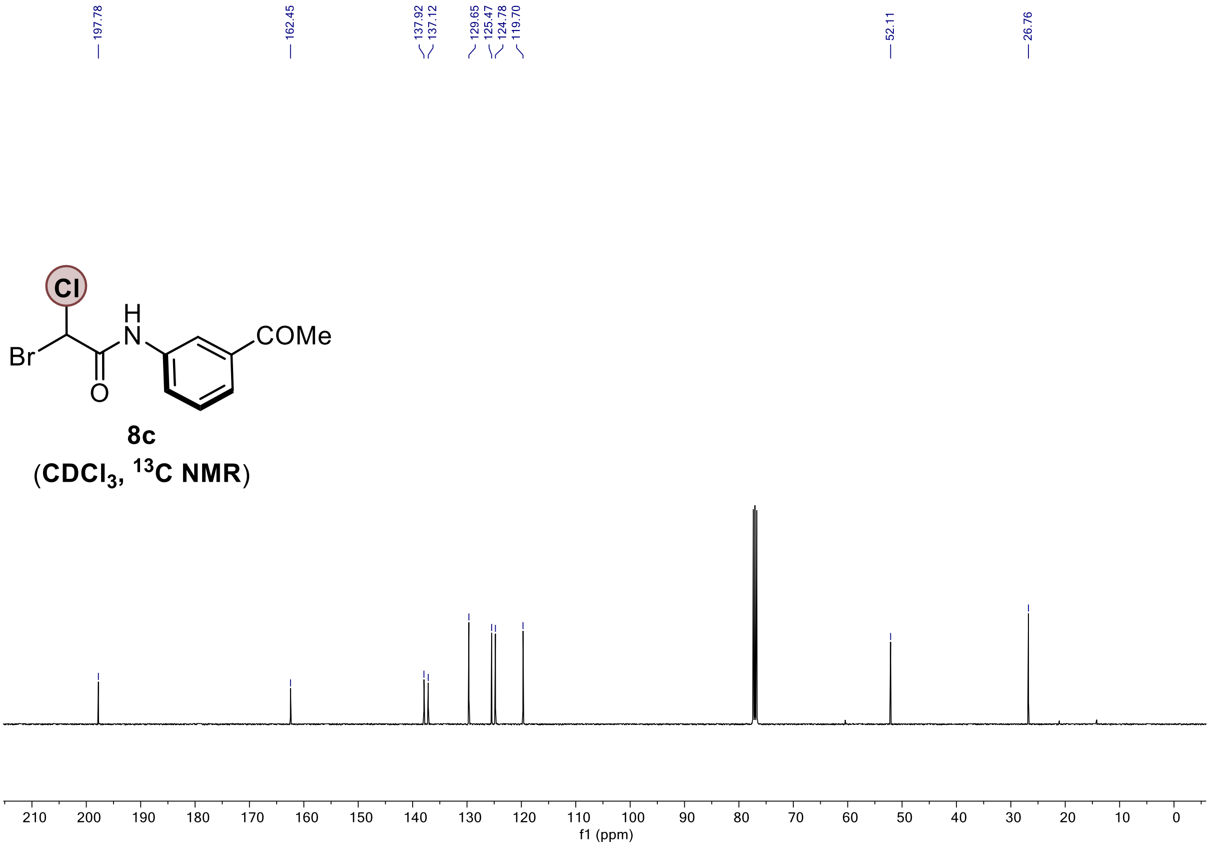
197.78
162.45
137.92
137.12
129.65
125.47
124.78
119.70
52.11
26.76
Cl
H
N
COMe
Br
O
8c
(CDCl3, 13C NMR)
210 200 190 180 170 160 150 140 130 120 110 100 90 80 70 60 50 40 30 20 10 0
f1 (ppm)

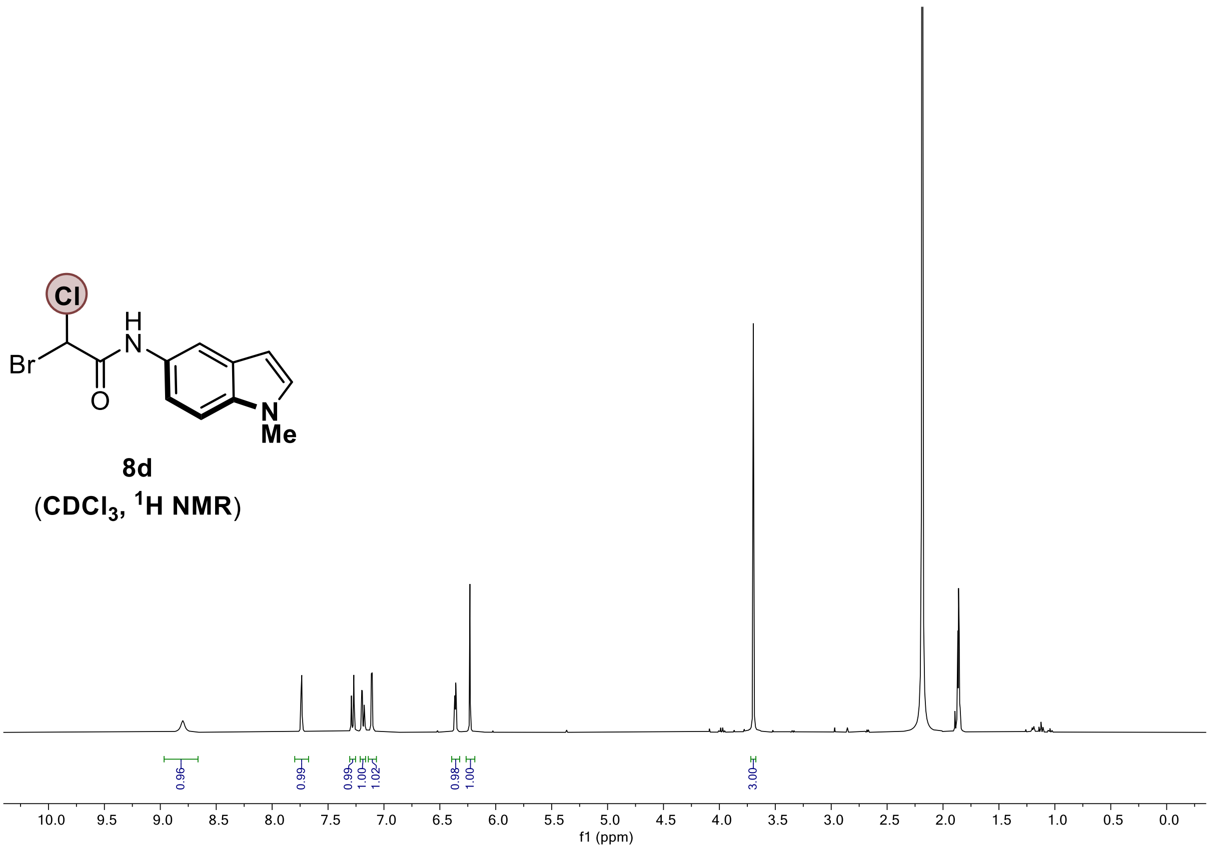

8d
(**CDCl$_3$, ^{1}H NMR**)

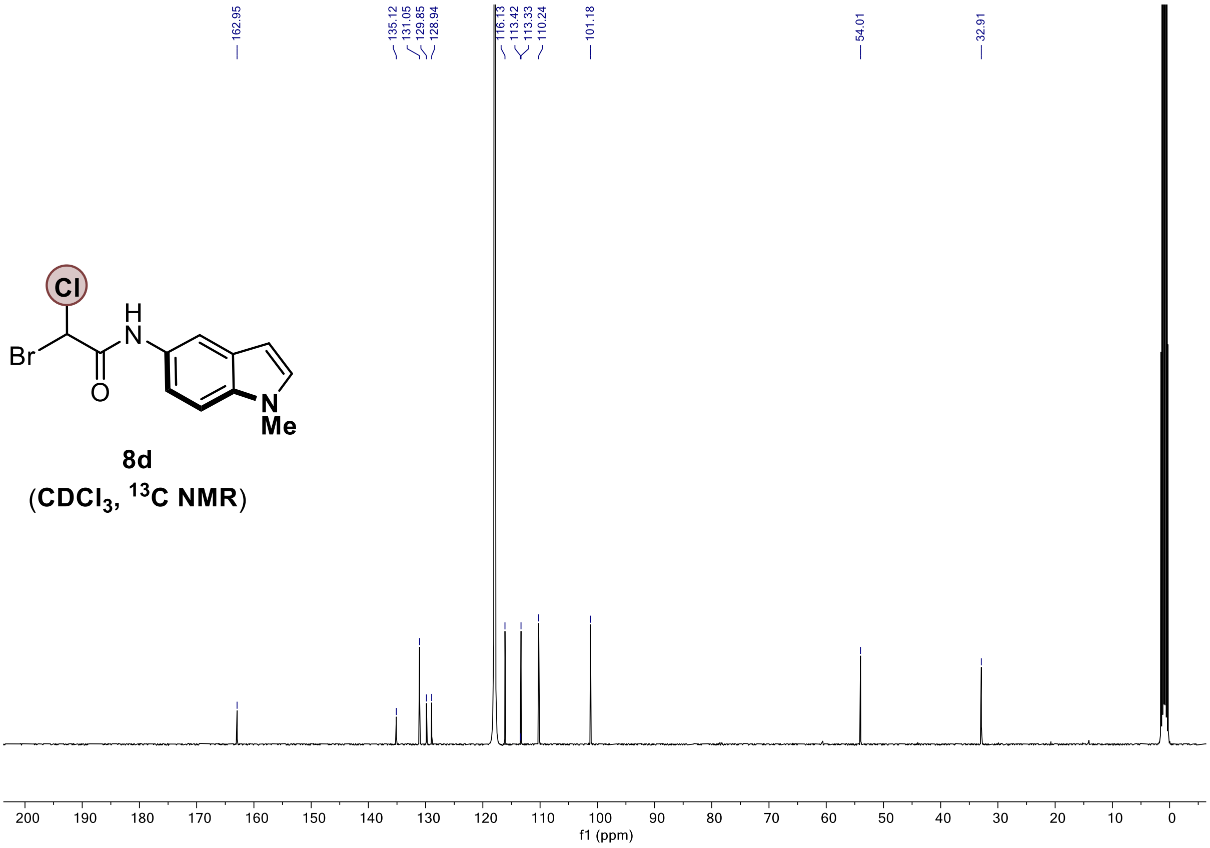

8d
(**CDCl$_3$, ^{13}C NMR**)

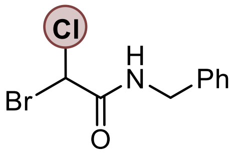

8e

(**CDCl$_3$, ^{1}H NMR**)

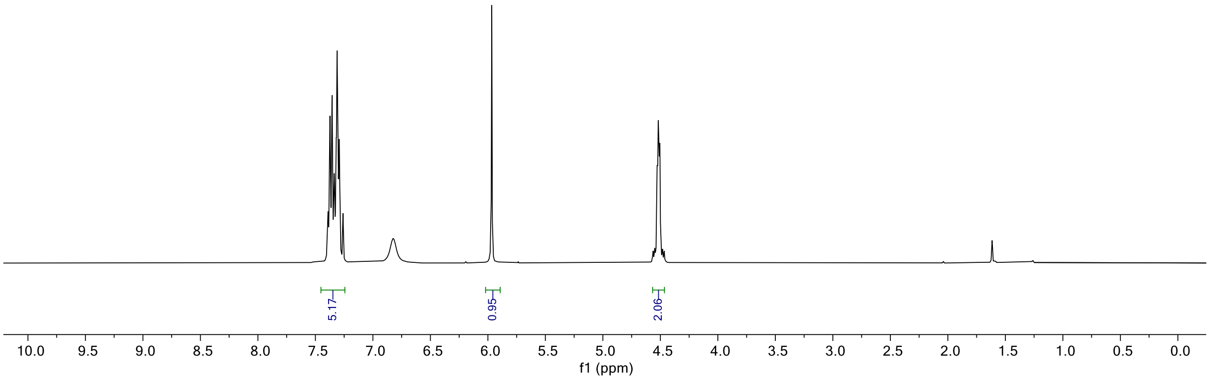

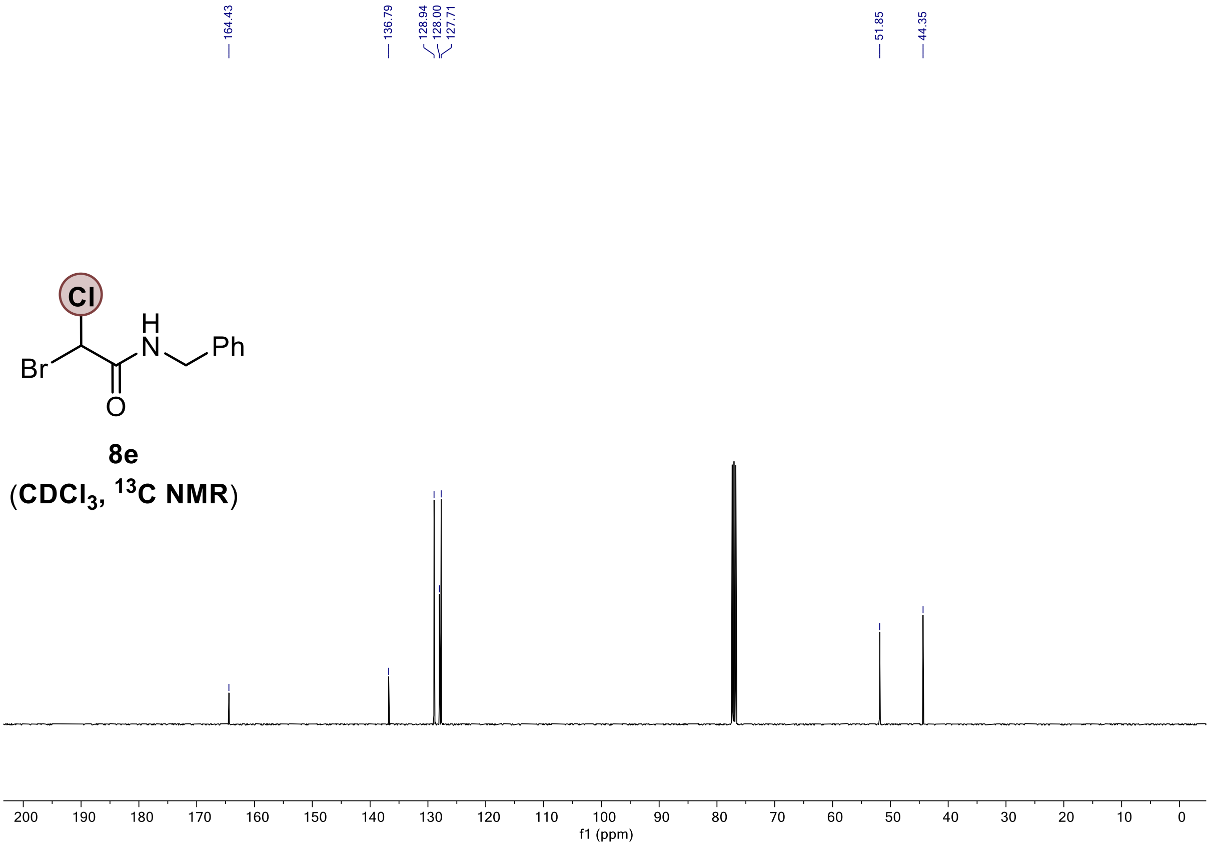

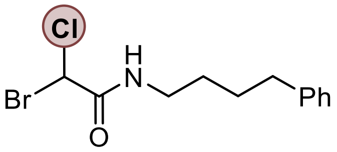

8f
($CDCl_3$, ^{1}H NMR)

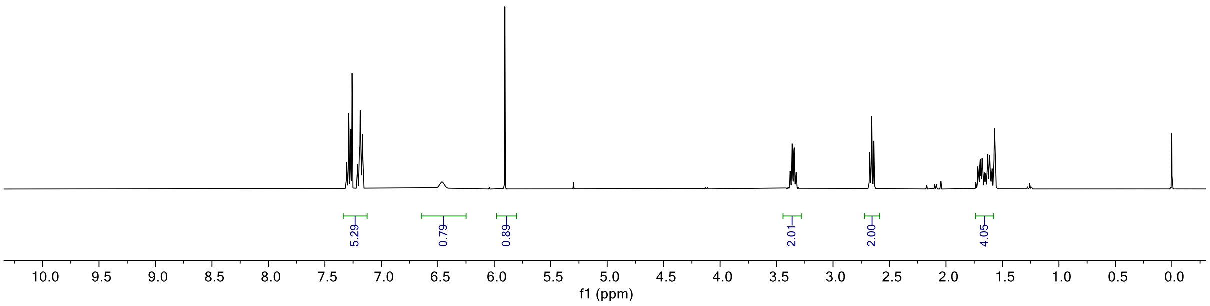

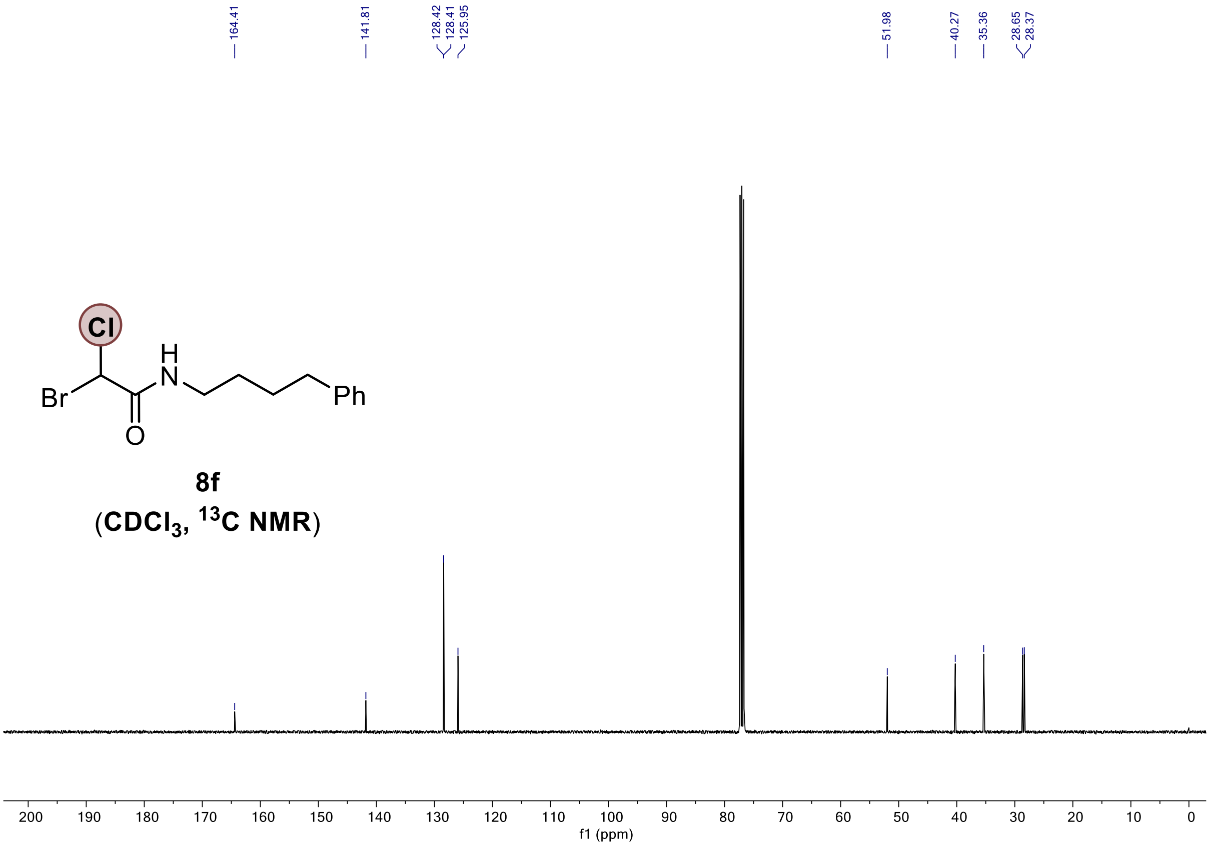

8f
($CDCl_3$, ^{13}C NMR)

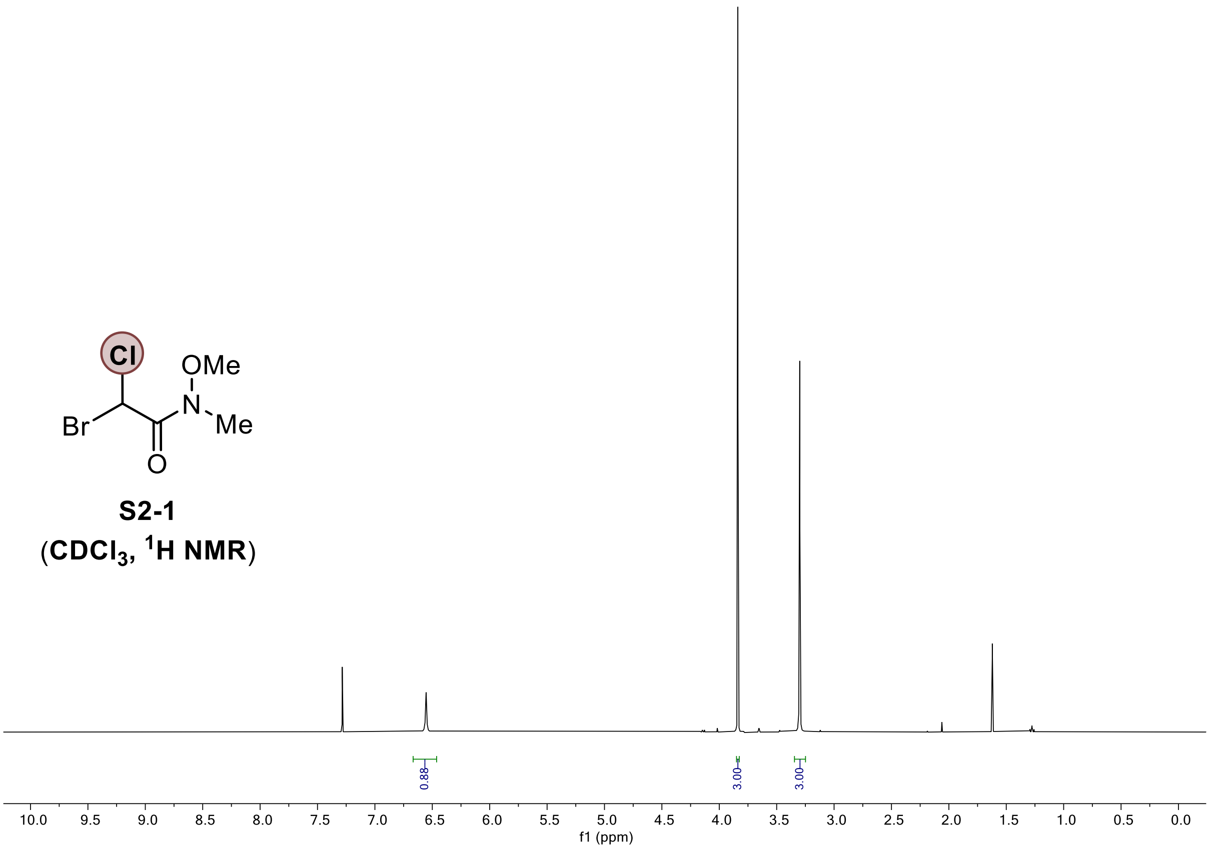

S2-1
(CDCl$_3$, ^{1}H NMR)

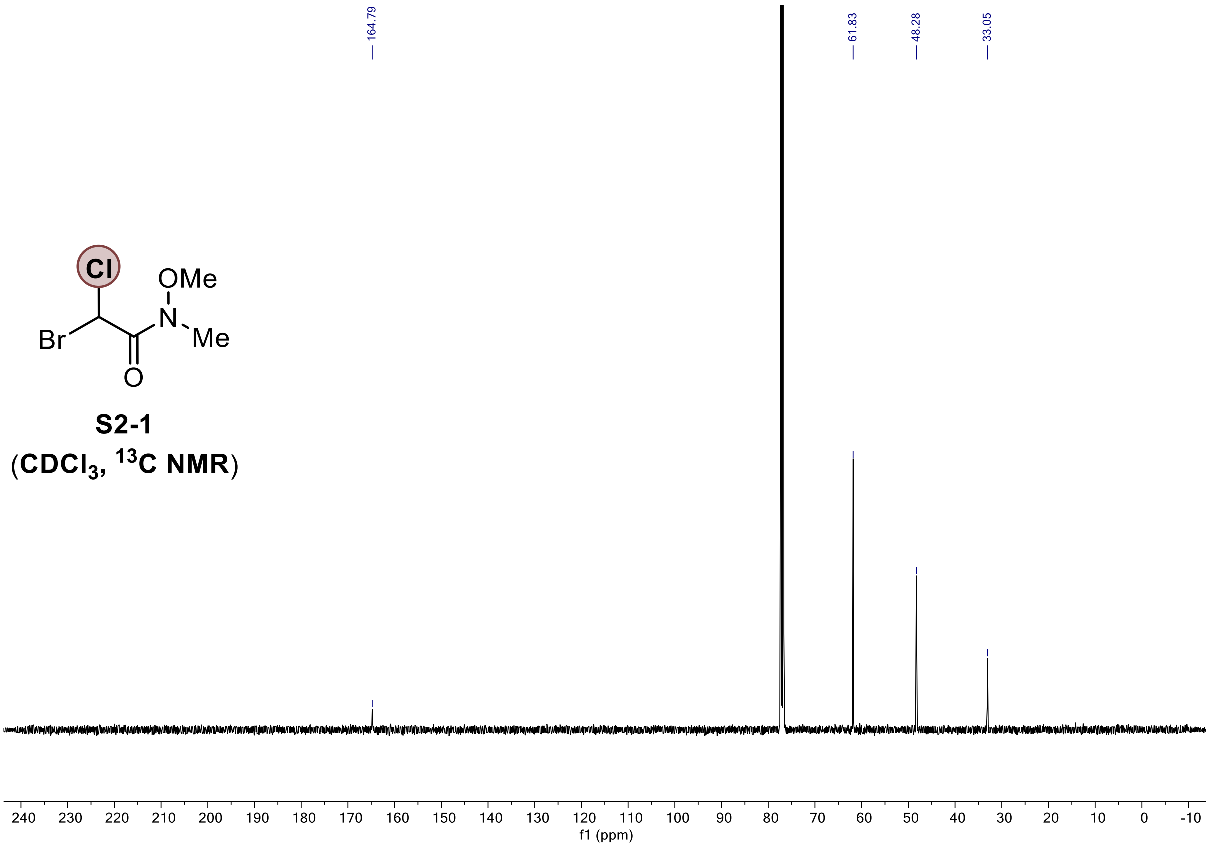

S2-1
(CDCl$_3$, ^{13}C NMR)

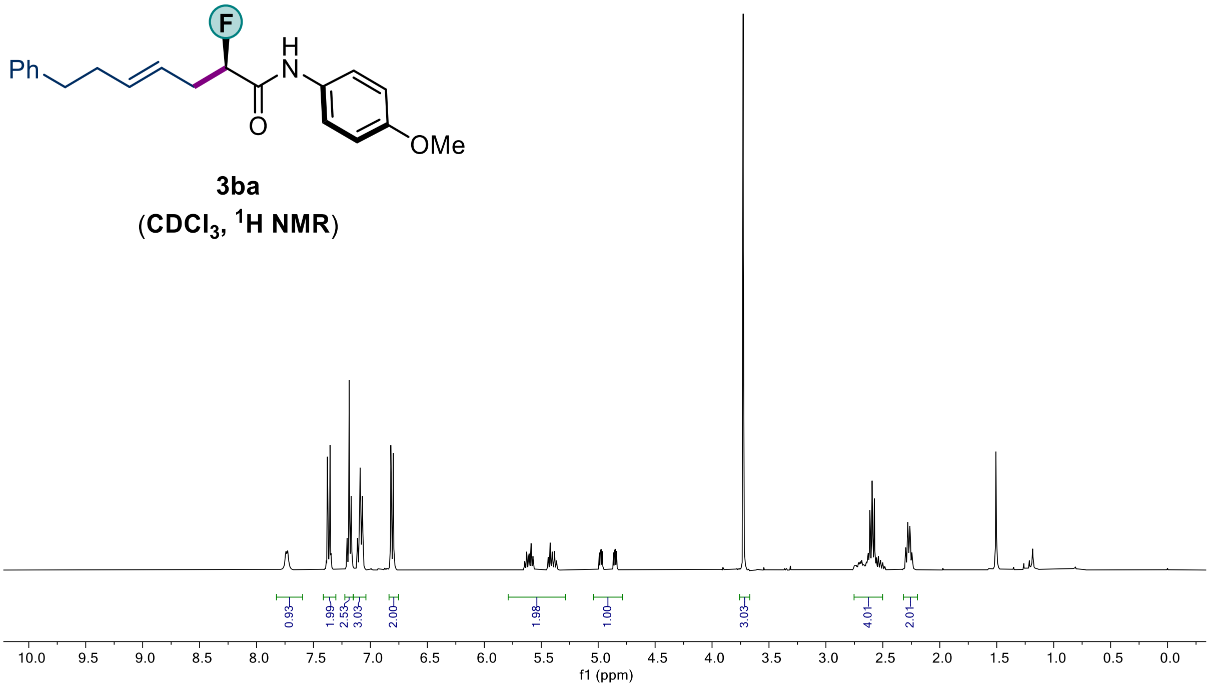

3ba
(CDCl3, 1H NMR)
f1 (ppm)

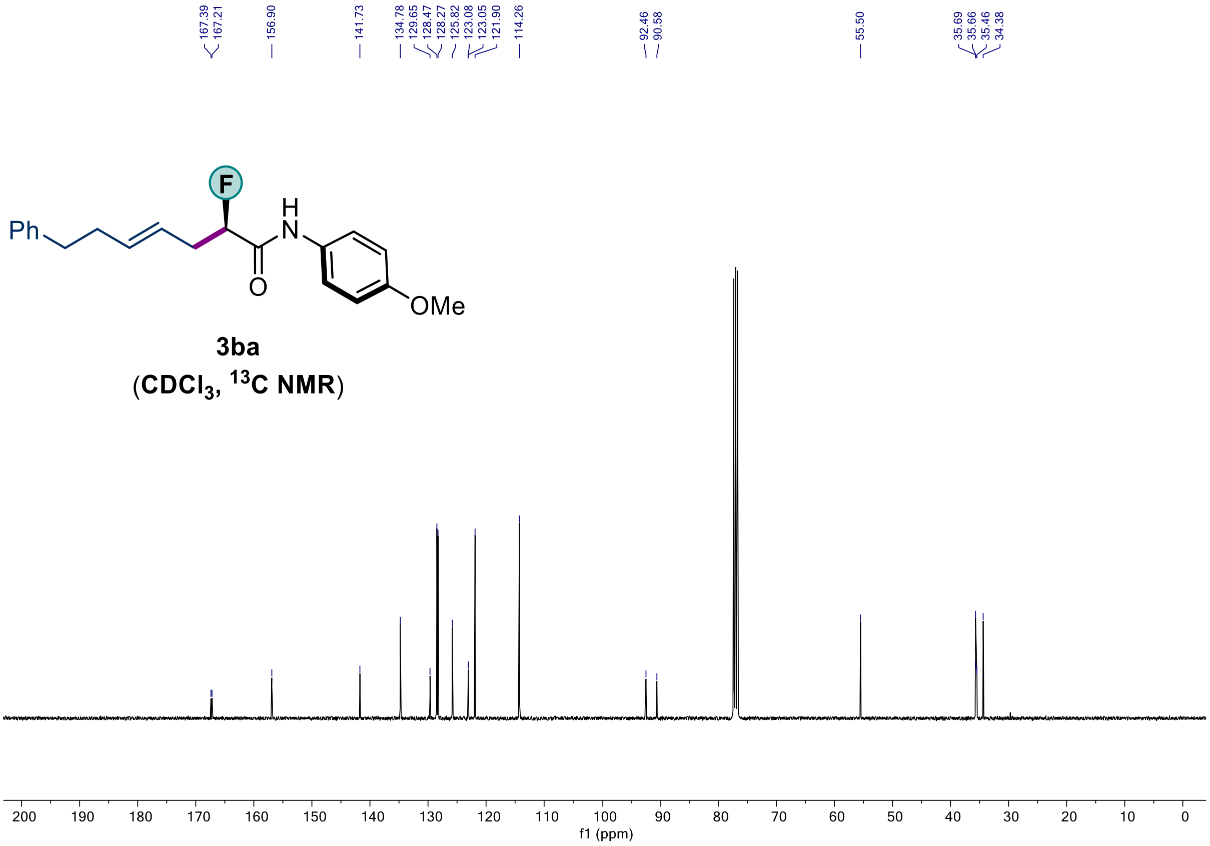

167.39
167.21
156.90
141.73
134.78
129.65
128.47
128.27
125.82
123.08
123.05
121.90
114.26
92.46
90.58
55.50
35.69
35.66
35.46
34.38
3ba
(CDCl3, 13C NMR)
f1 (ppm)

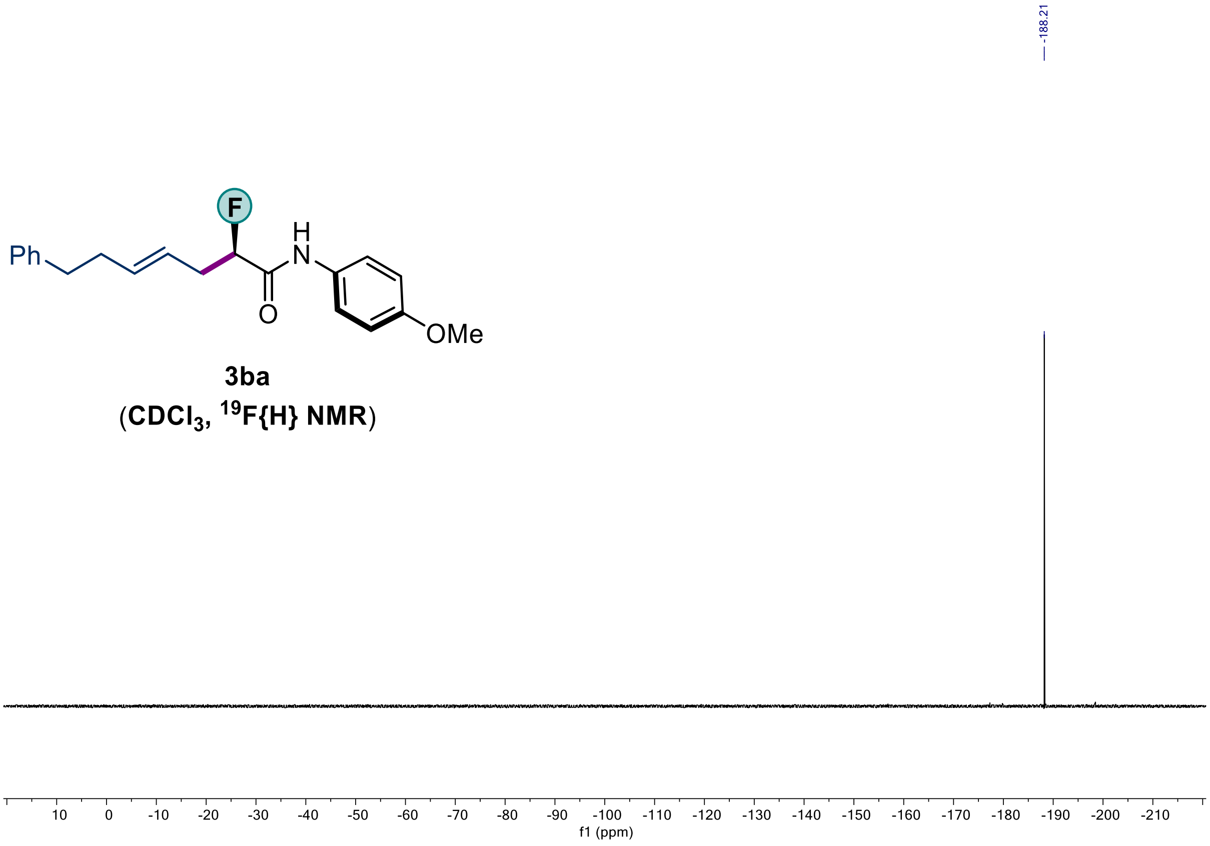

-188.21
Ph
F
H
N
O
OMe
3ba
(CDCl$_3$, ^{19}F{H} NMR)
10 0 -10 -20 -30 -40 -50 -60 -70 -80 -90 -100 -110 -120 -130 -140 -150 -160 -170 -180 -190 -200 -210
f1 (ppm)

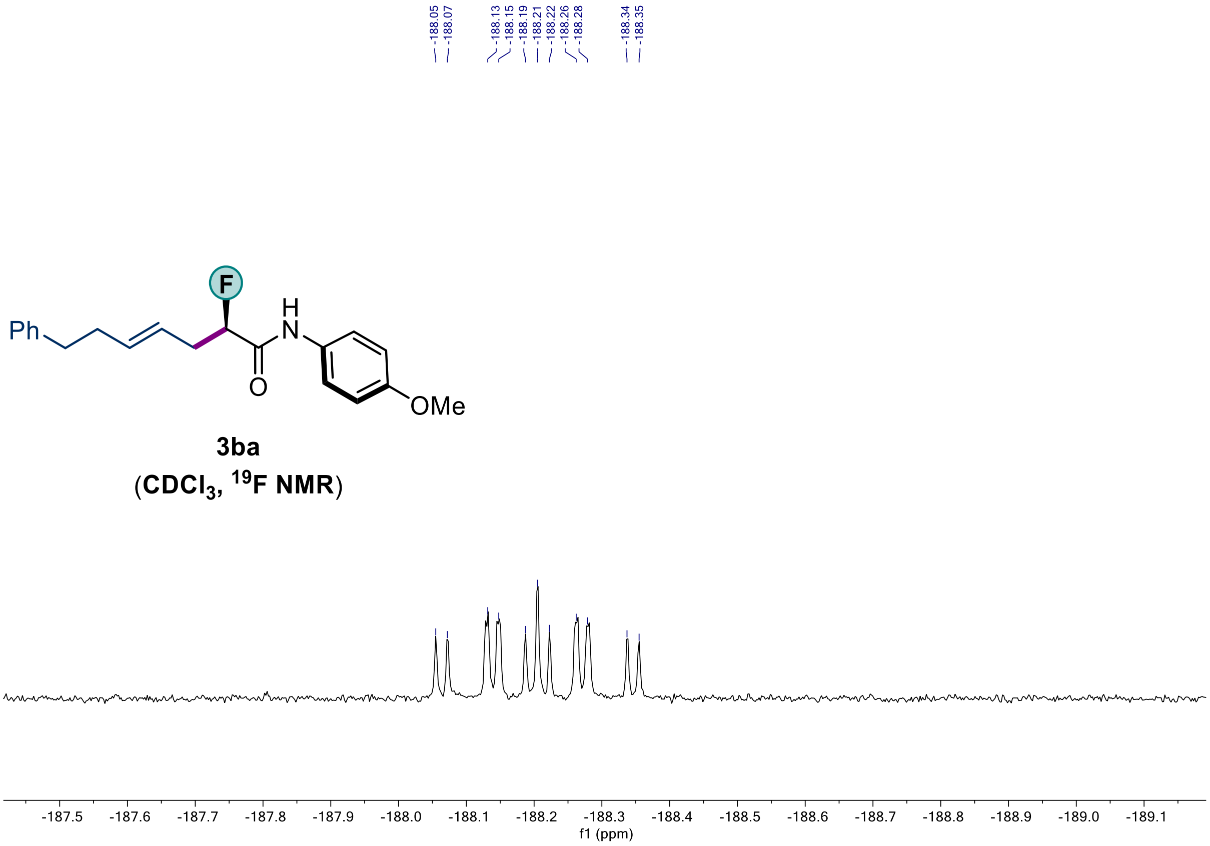

-188.05
-188.07
-188.13
-188.15
-188.19
-188.21
-188.22
-188.26
-188.28
-188.34
-188.35
Ph
F
H
N
O
OMe
3ba
(CDCl$_3$, ^{19}F NMR)
-187.5 -187.6 -187.7 -187.8 -187.9 -188.0 -188.1 -188.2 -188.3 -188.4 -188.5 -188.6 -188.7 -188.8 -188.9 -189.0 -189.1
f1 (ppm)

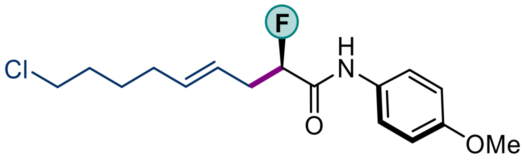

3ca
(**$CDCl_3$, ^{1}H NMR**)

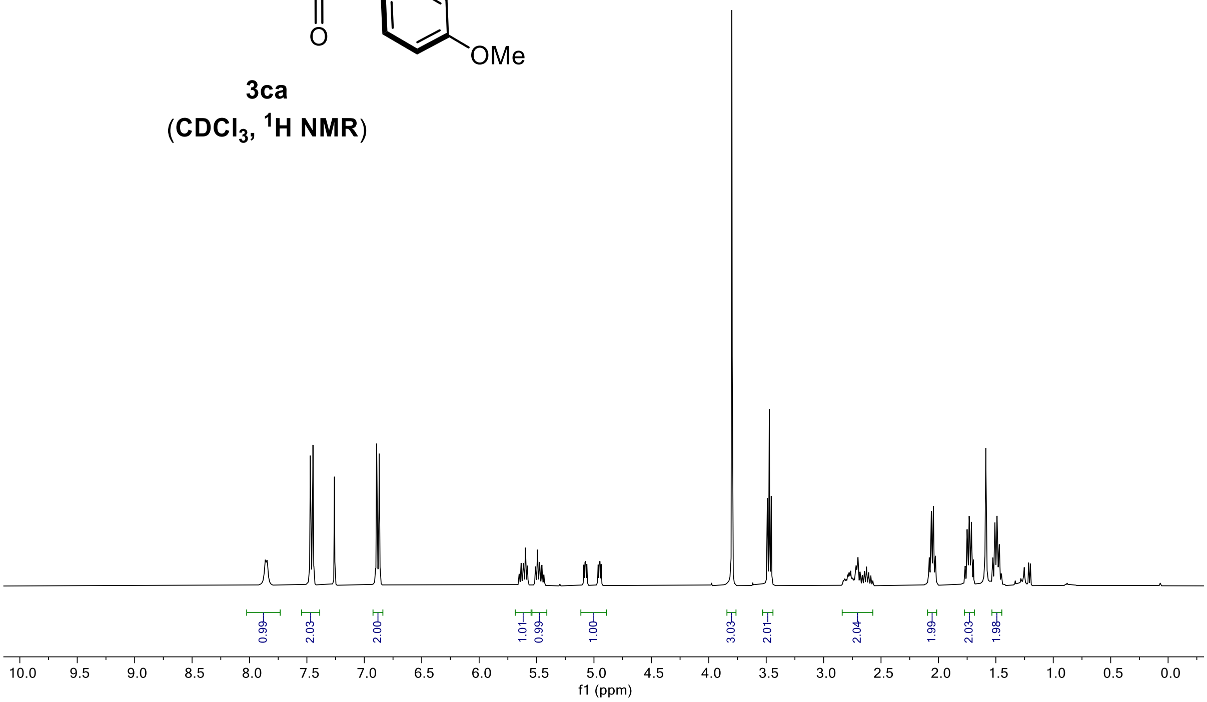

3ca
(**$CDCl_3$, ^{13}C NMR**)

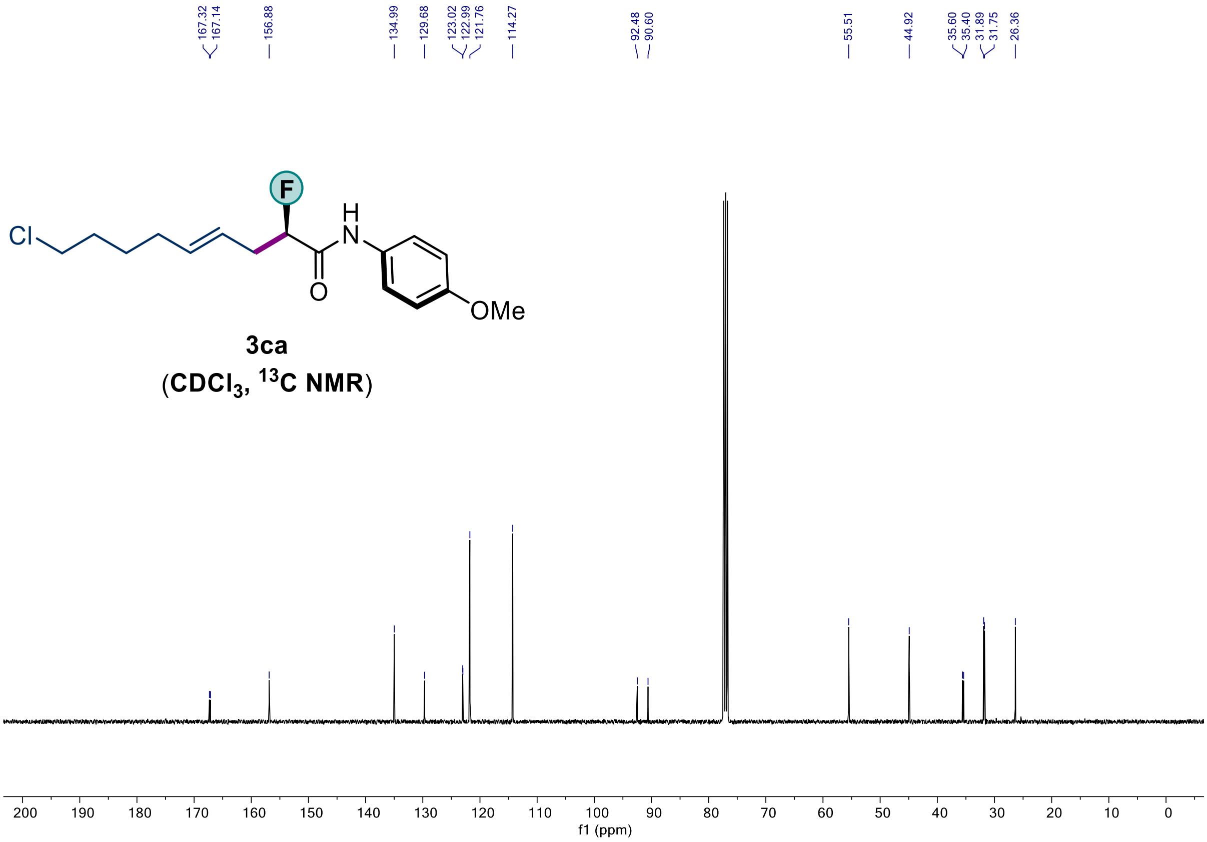

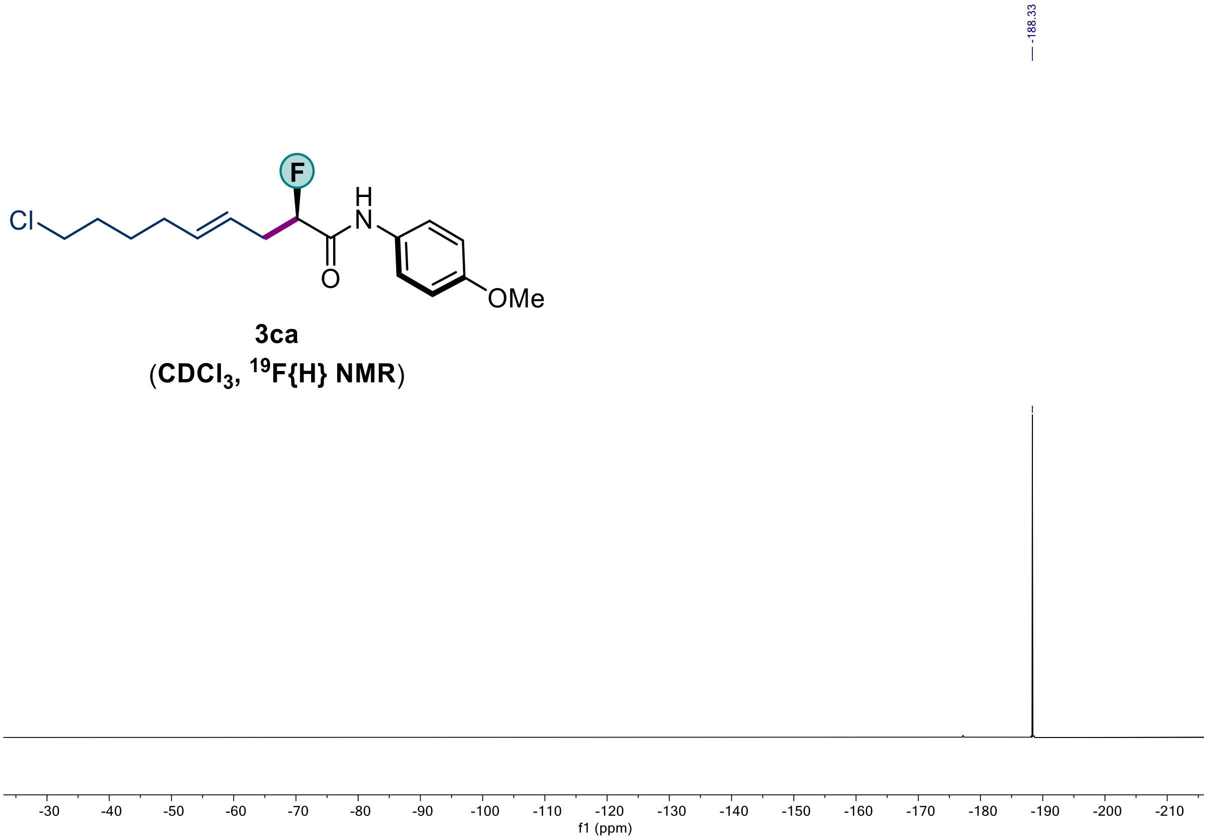
-188.33
Cl
F
H
N
O
OMe
3ca
(CDCl3, 19F{H} NMR)
-30 -40 -50 -60 -70 -80 -90 -100 -110 -120 -130 -140 -150 -160 -170 -180 -190 -200 -210
f1 (ppm)

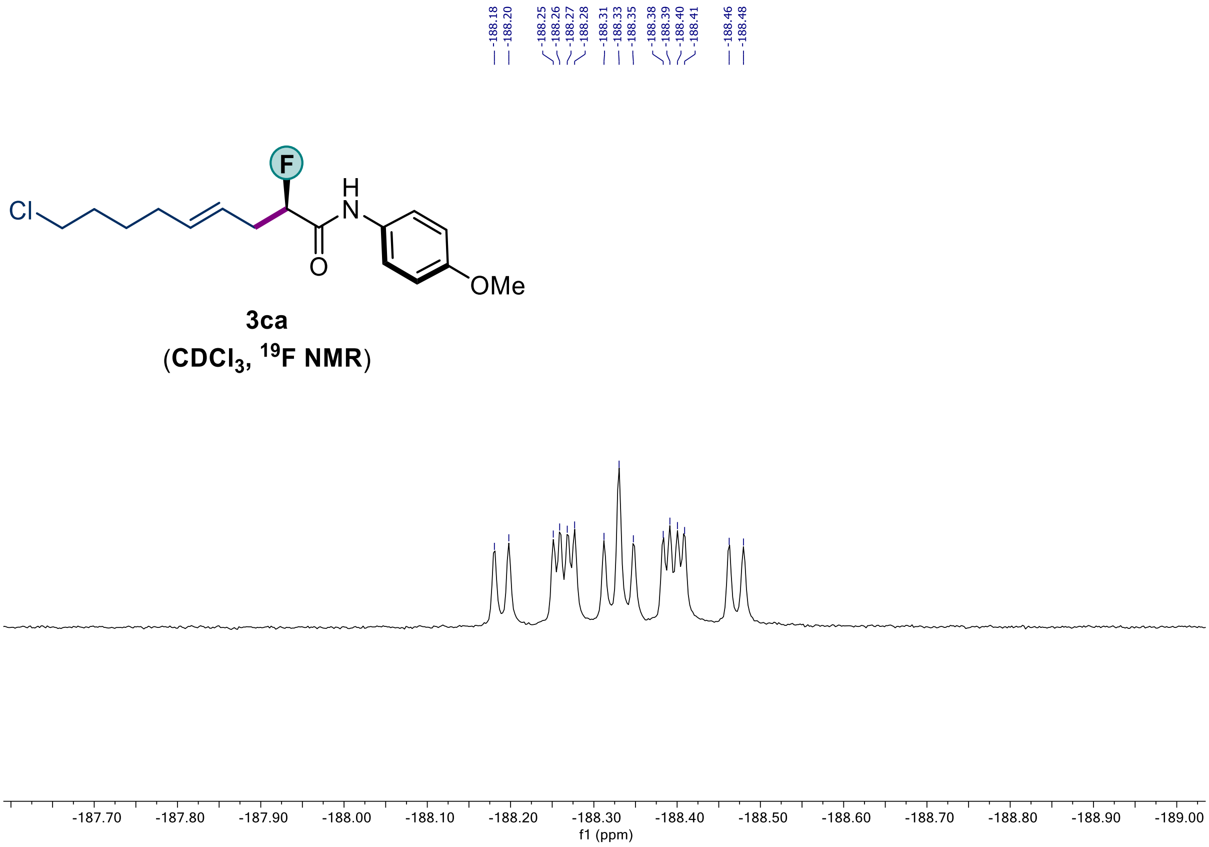
-188.18
-188.20
-188.25
-188.26
-188.27
-188.28
-188.31
-188.33
-188.35
-188.38
-188.39
-188.40
-188.41
-188.46
-188.48
Cl
F
H
N
O
OMe
3ca
(CDCl3, 19F NMR)
-187.70 -187.80 -187.90 -188.00 -188.10 -188.20 -188.30 -188.40 -188.50 -188.60 -188.70 -188.80 -188.90 -189.00
f1 (ppm)

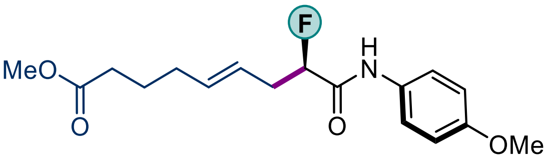

3da
($CDCl_3$, ^{1}H NMR)

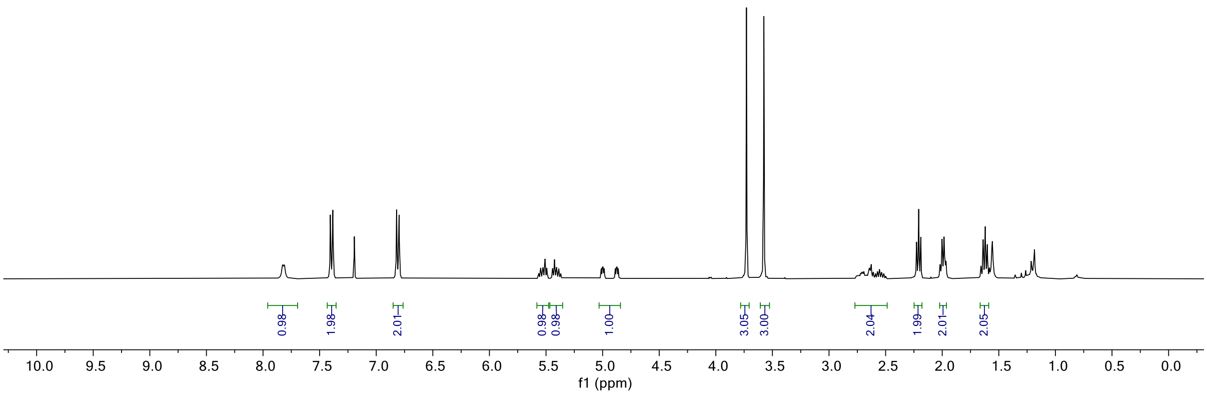

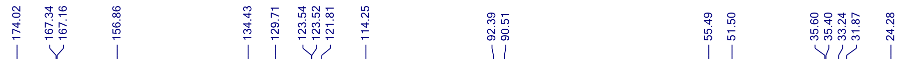

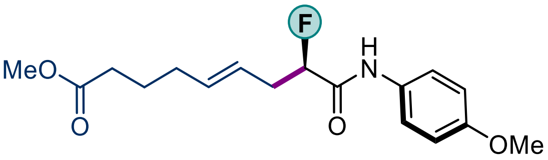

3da
($CDCl_3$, ^{13}C NMR)

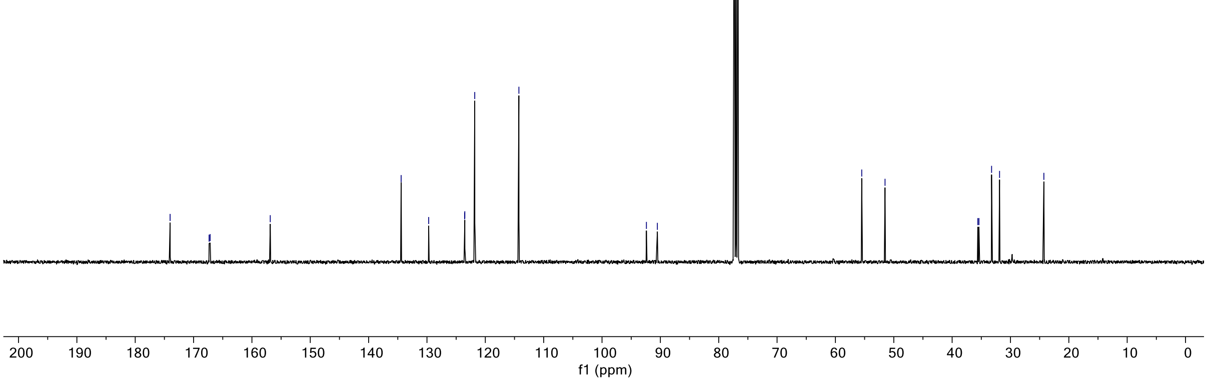

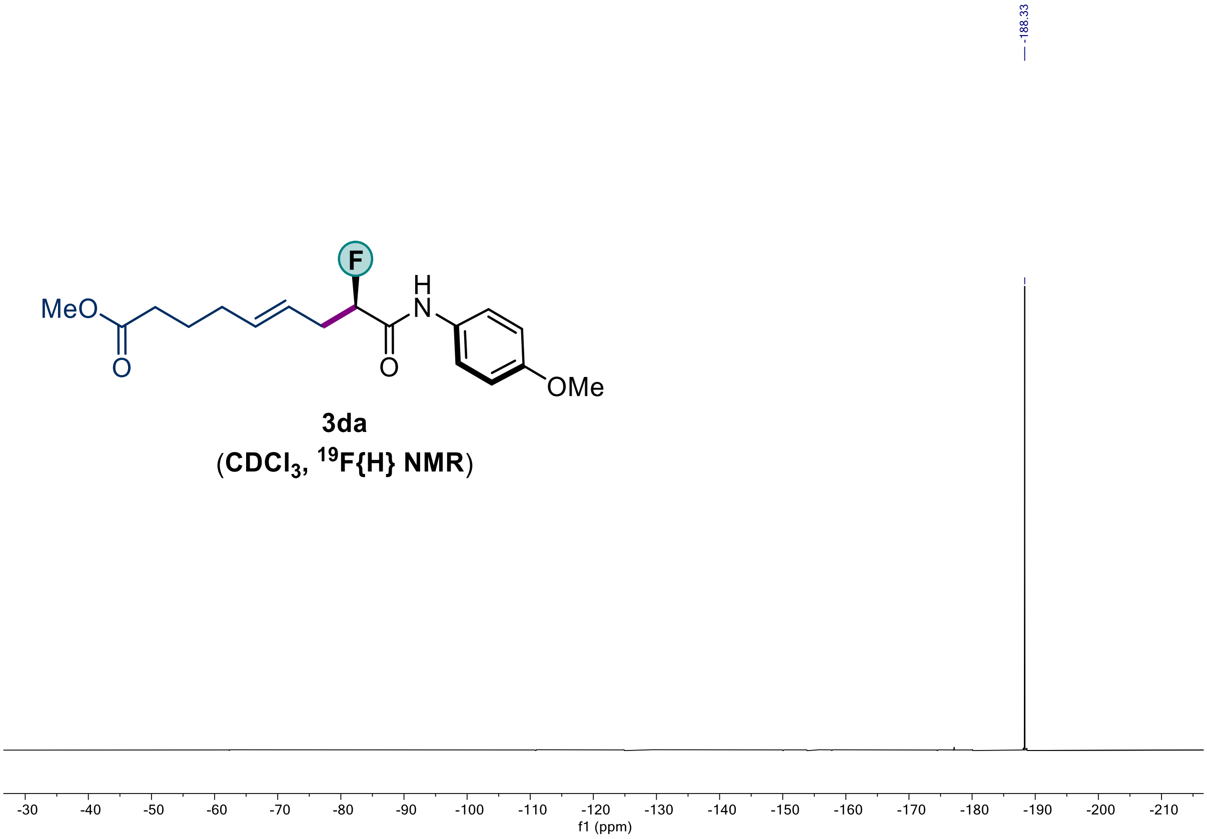
-188.33
MeO
O
F
H
N
O
OMe
3da
(CDCl3, 19F{H} NMR)
-30 -40 -50 -60 -70 -80 -90 -100 -110 -120 -130 -140 -150 -160 -170 -180 -190 -200 -210
f1 (ppm)

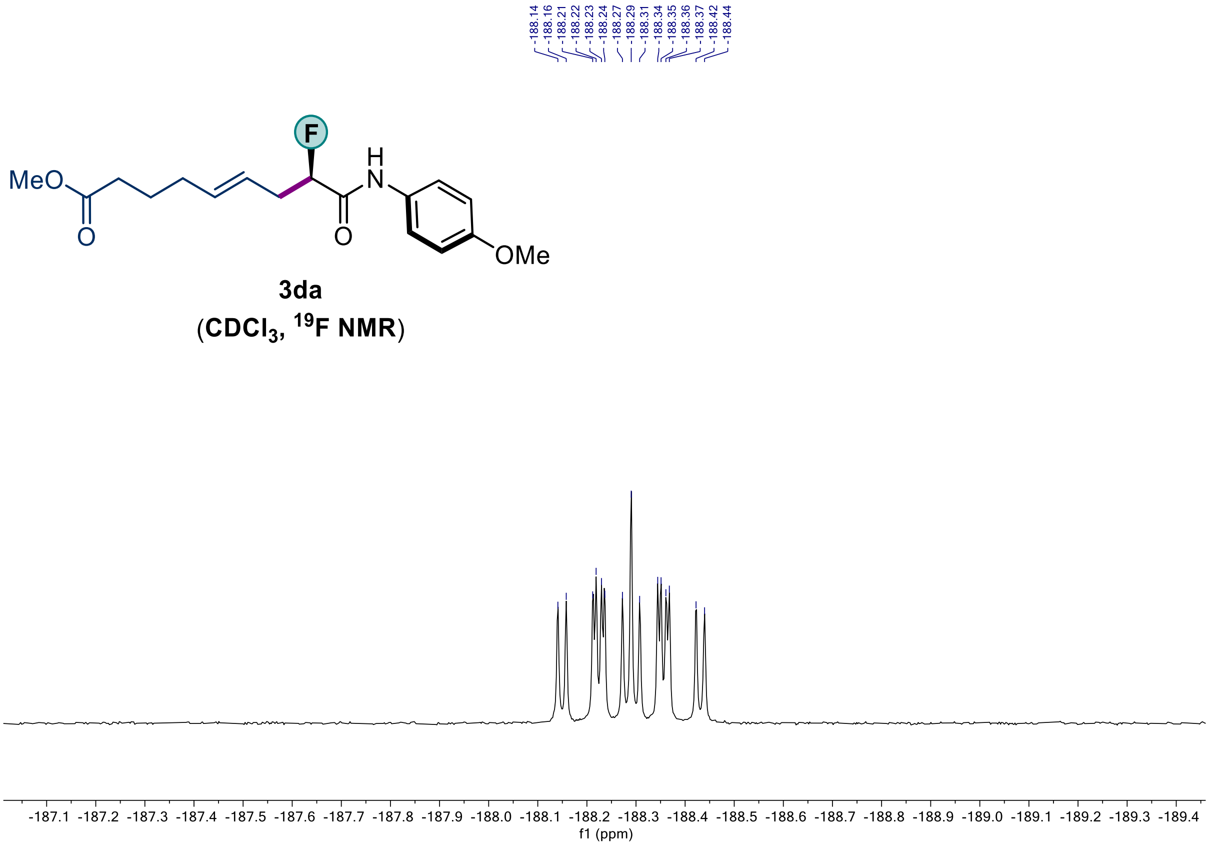
-188.14
-188.16
-188.21
-188.22
-188.23
-188.24
-188.27
-188.29
-188.31
-188.34
-188.35
-188.36
-188.37
-188.42
-188.44
MeO
O
F
H
N
O
OMe
3da
(CDCl3, 19F NMR)
-187.1 -187.2 -187.3 -187.4 -187.5 -187.6 -187.7 -187.8 -187.9 -188.0 -188.1 -188.2 -188.3 -188.4 -188.5 -188.6 -188.7 -188.8 -188.9 -189.0 -189.1 -189.2 -189.3 -189.4
f1 (ppm)

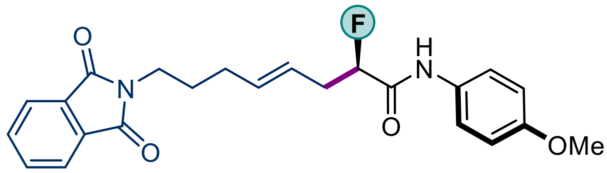

3ea
($CDCl_3$, ^1H NMR)

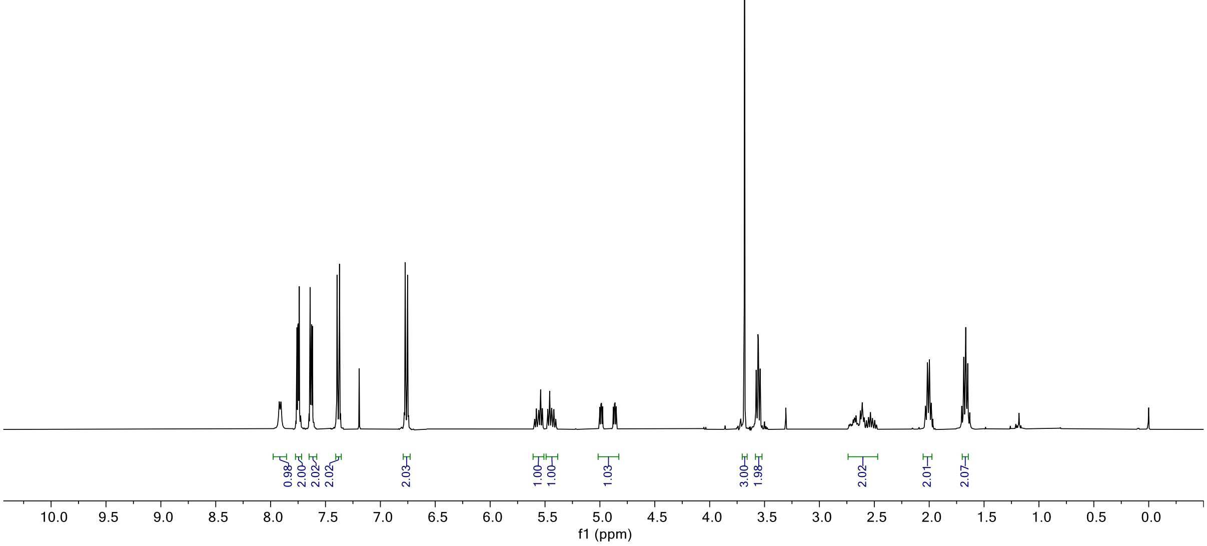

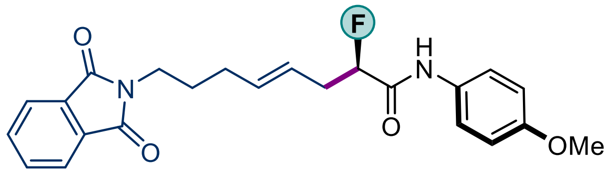

3ea
($CDCl_3$, ^{13}C NMR)

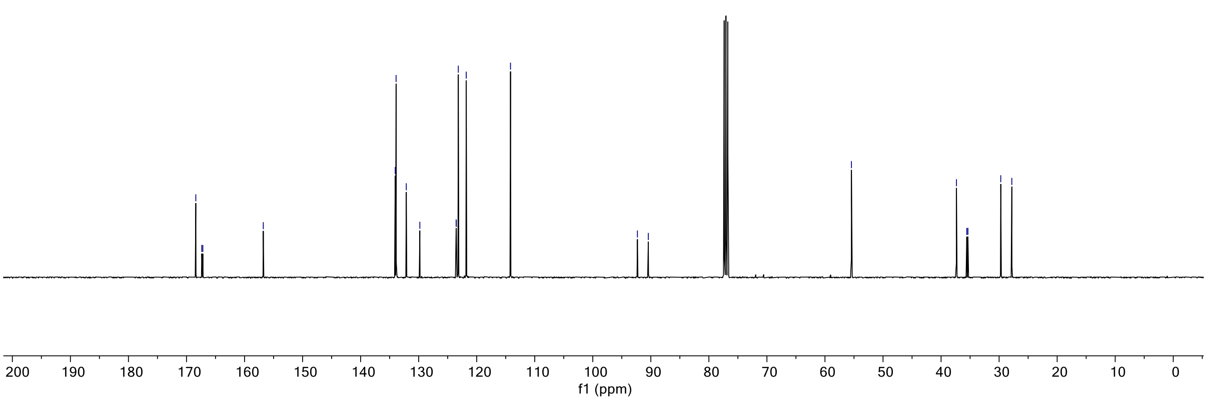

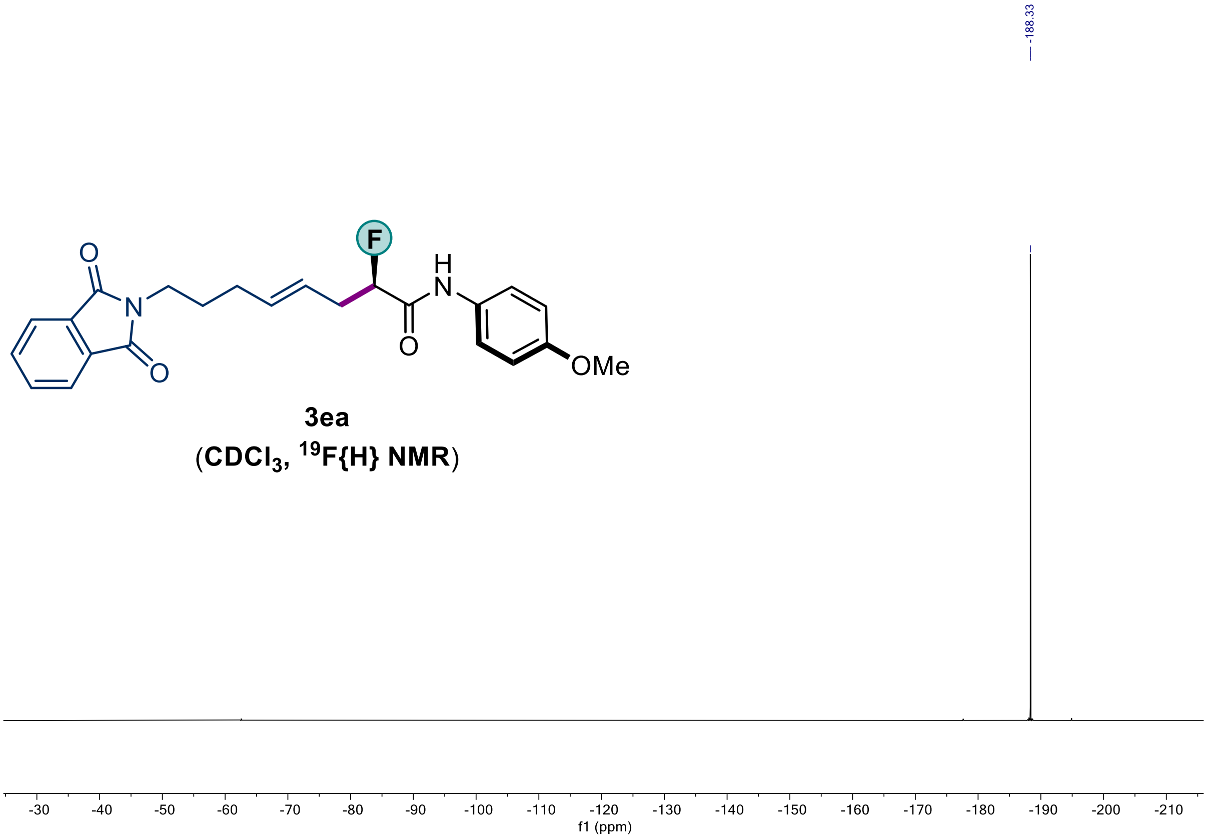

3ea
(**CDCl$_3$, ^{19}F{H} NMR**)

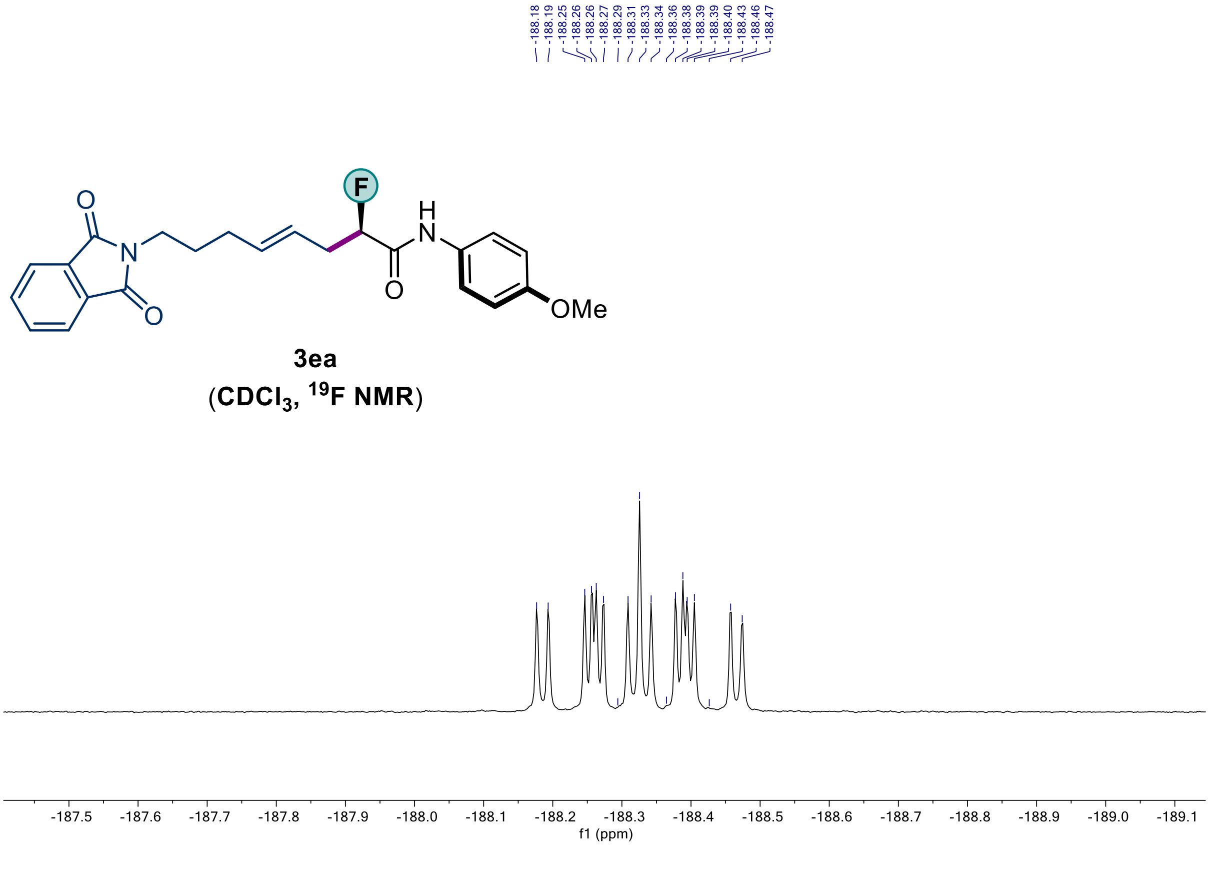

3ea
(**CDCl$_3$, ^{19}F NMR**)

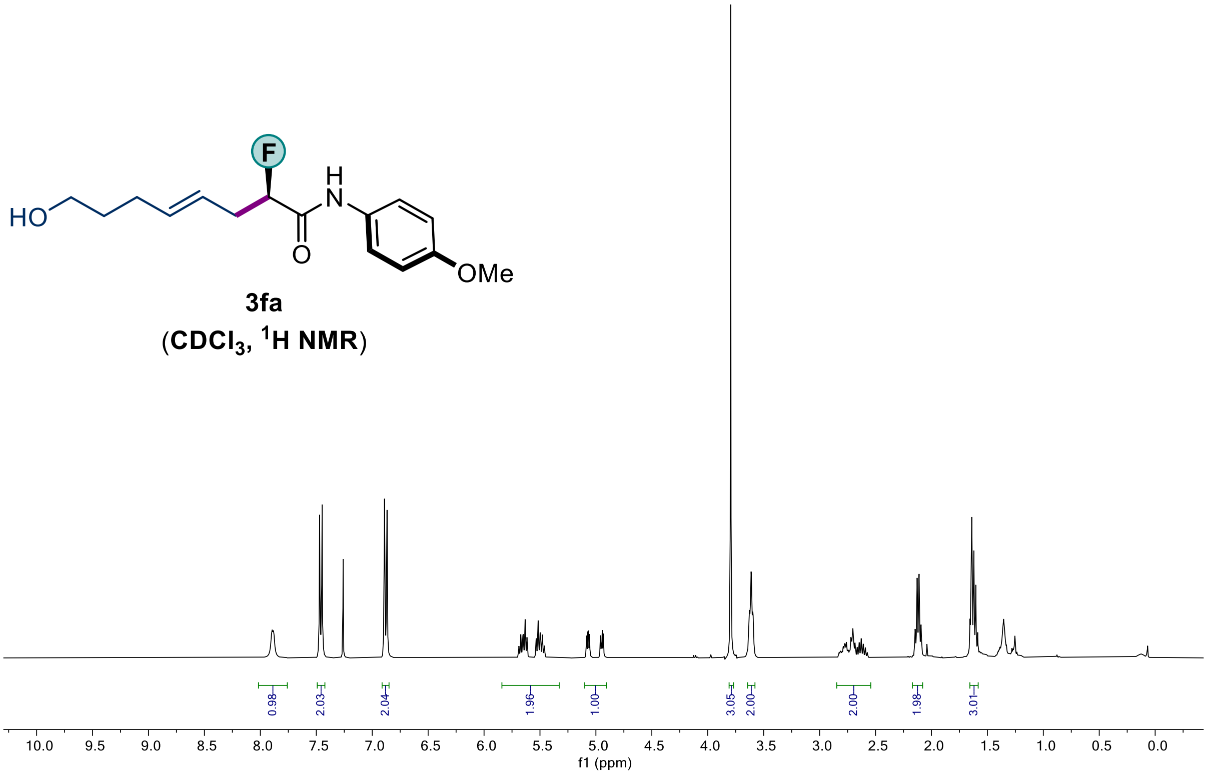
F
H
N
HO
O
OMe
3fa
(CDCl3, 1H NMR)
0.98
2.03
2.04
1.96
1.00
3.05
2.00
2.00
1.98
3.01
10.0 9.5 9.0 8.5 8.0 7.5 7.0 6.5 6.0 5.5 5.0 4.5 4.0 3.5 3.0 2.5 2.0 1.5 1.0 0.5 0.0
f1 (ppm)

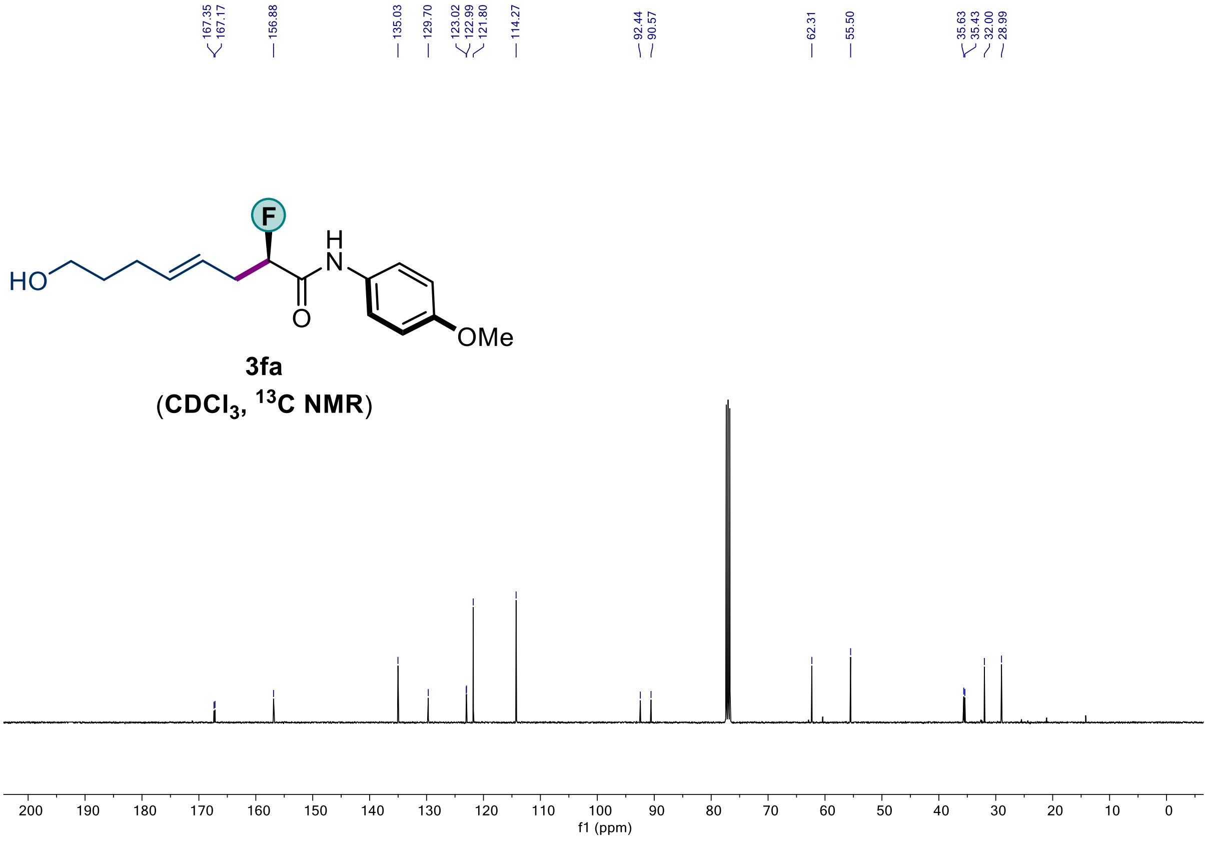
167.35
167.17
156.88
135.03
129.70
123.02
122.99
121.80
114.27
92.44
90.57
62.31
55.50
35.63
35.43
32.00
28.99
F
H
N
HO
O
OMe
3fa
(CDCl3, 13C NMR)
200 190 180 170 160 150 140 130 120 110 100 90 80 70 60 50 40 30 20 10 0
f1 (ppm)

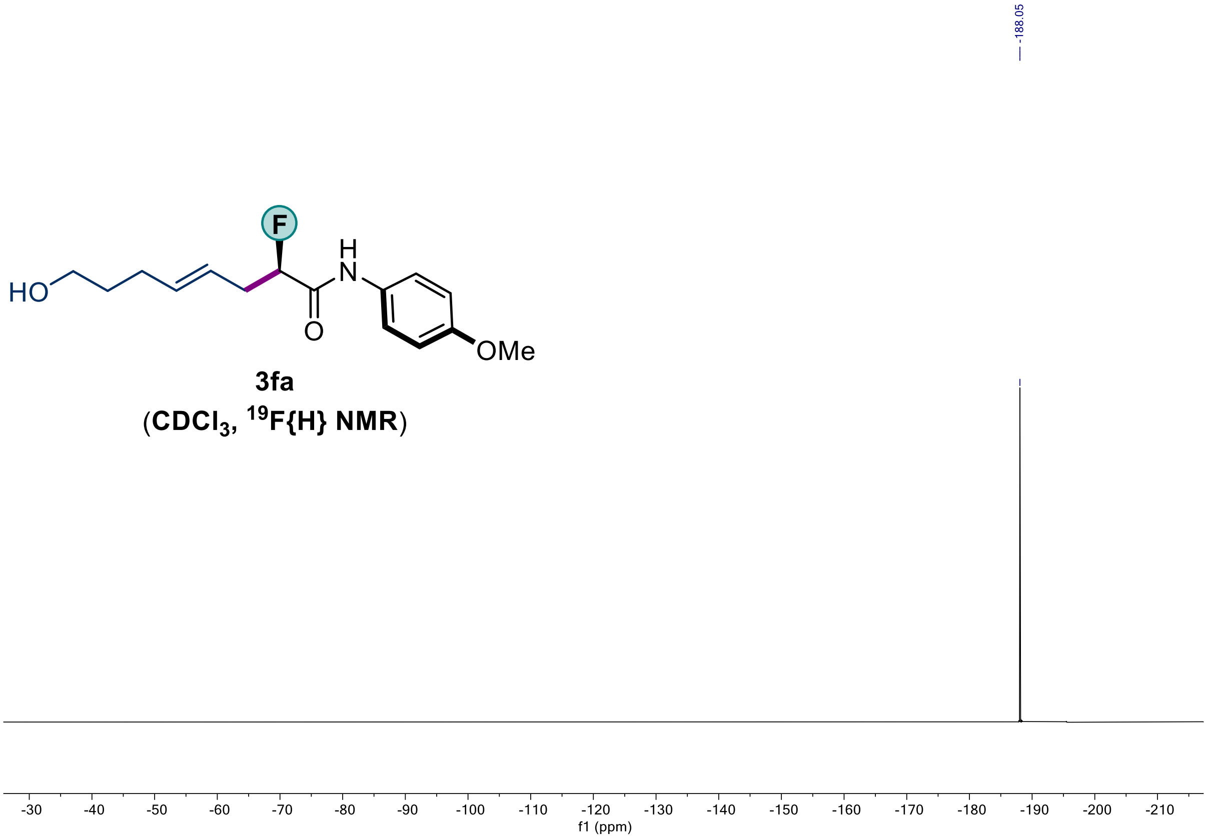
-188.05
HO
F
H
N
O
OMe
3fa
(CDCl3, 19F{H} NMR)
-30 -40 -50 -60 -70 -80 -90 -100 -110 -120 -130 -140 -150 -160 -170 -180 -190 -200 -210
f1 (ppm)

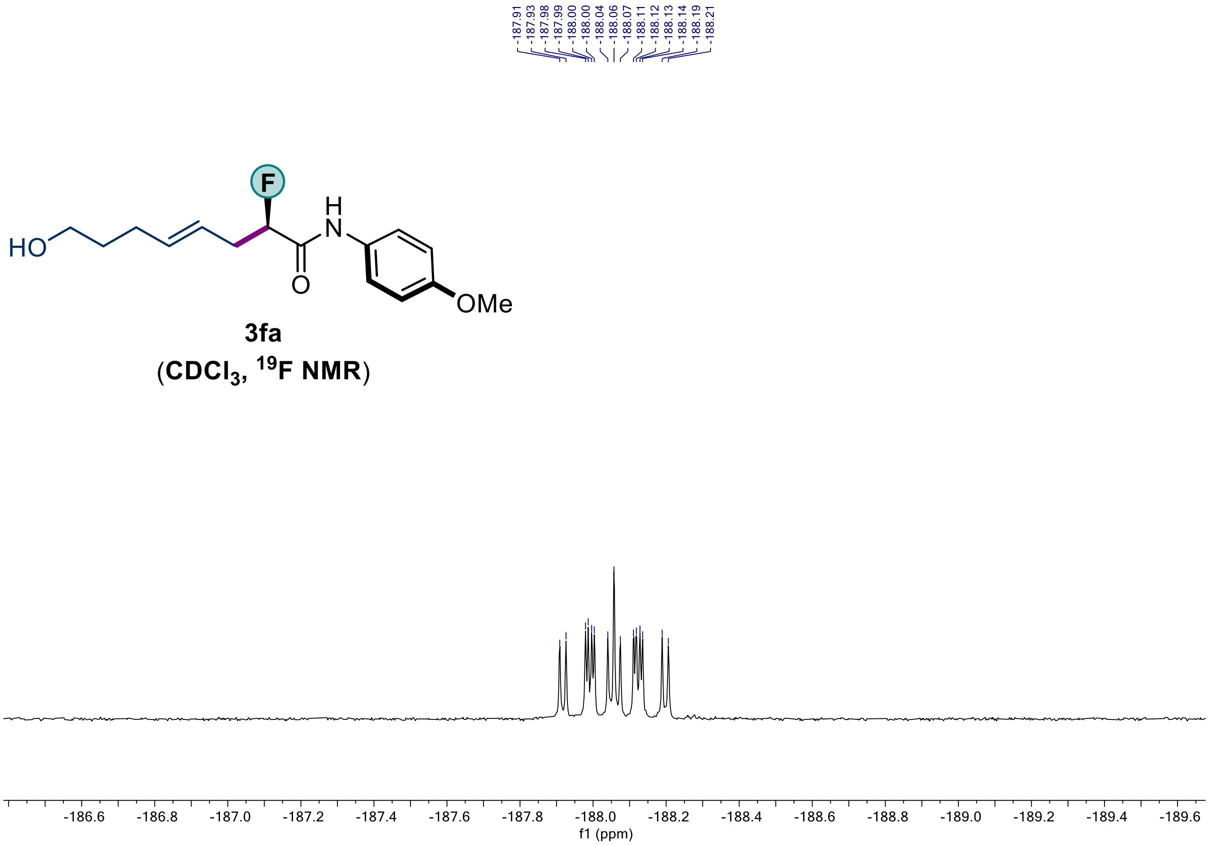
-187.91
-187.93
-187.98
-187.99
-188.00
-188.04
-188.06
-188.07
-188.11
-188.12
-188.13
-188.14
-188.19
-188.21
HO
F
H
N
O
OMe
3fa
(CDCl3, 19F NMR)
-186.6 -186.8 -187.0 -187.2 -187.4 -187.6 -187.8 -188.0 -188.2 -188.4 -188.6 -188.8 -189.0 -189.2 -189.4 -189.6
f1 (ppm)

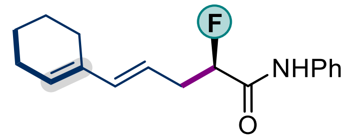

3gb
($CDCl_3$, ^1H NMR)

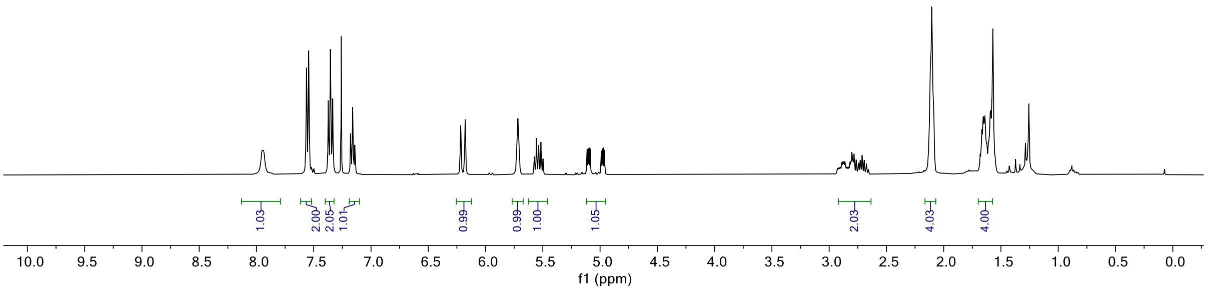

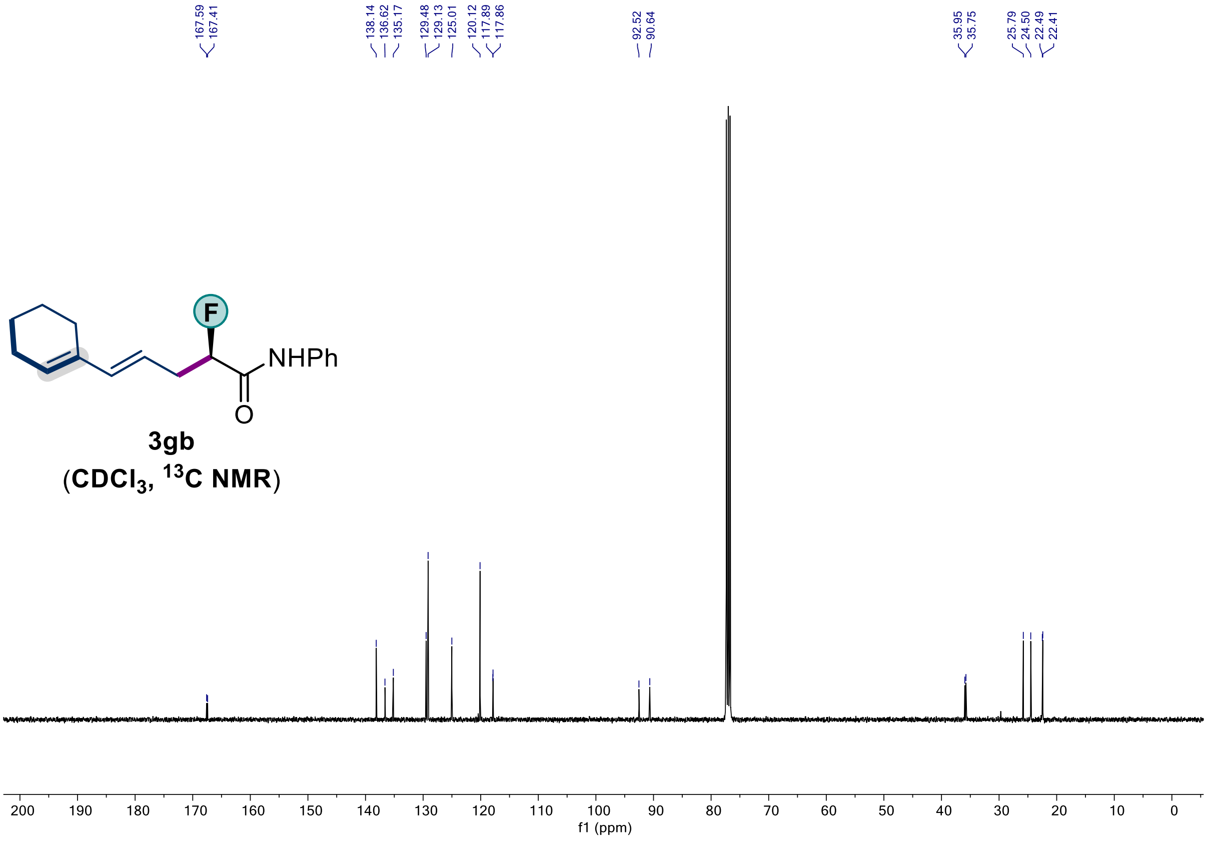

3gb
($CDCl_3$, ^{13}C NMR)

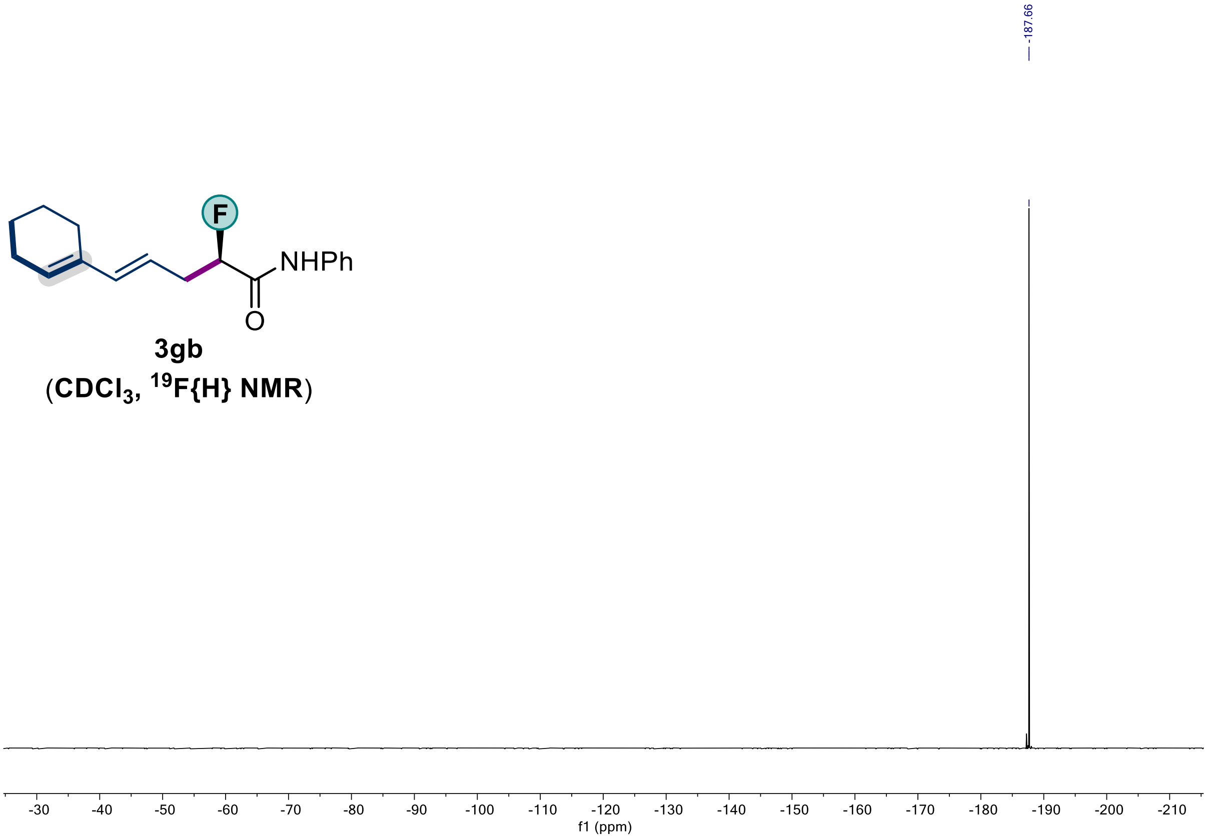

3gb
($CDCl_3$, $^{19}F\{H\}$ NMR)

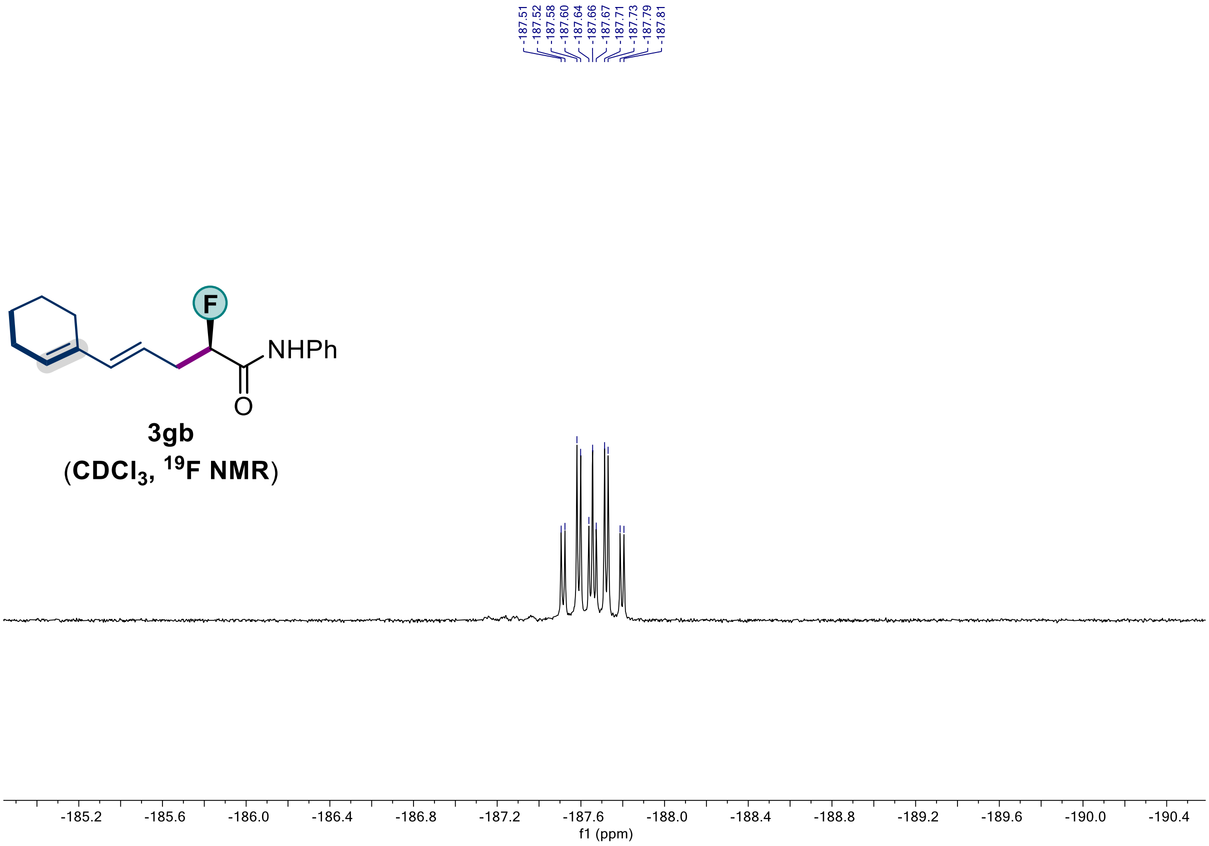

3gb
($CDCl_3$, ^{19}F NMR)

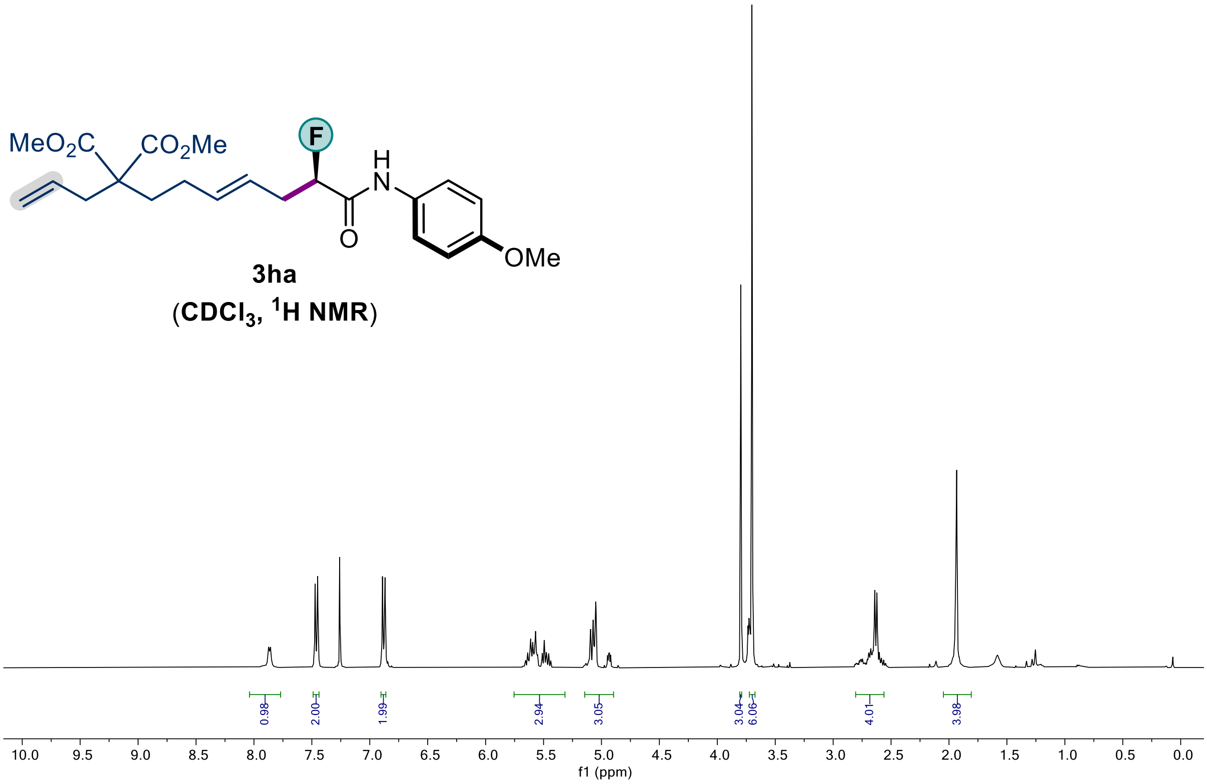
MeO2C
CO2Me
F
H
N
O
OMe
3ha
(CDCl3, 1H NMR)
0.98
2.00
1.99
2.94
3.05
3.04
6.06
4.01
3.98
10.0 9.5 9.0 8.5 8.0 7.5 7.0 6.5 6.0 5.5 5.0 4.5 4.0 3.5 3.0 2.5 2.0 1.5 1.0 0.5 0.0
f1 (ppm)

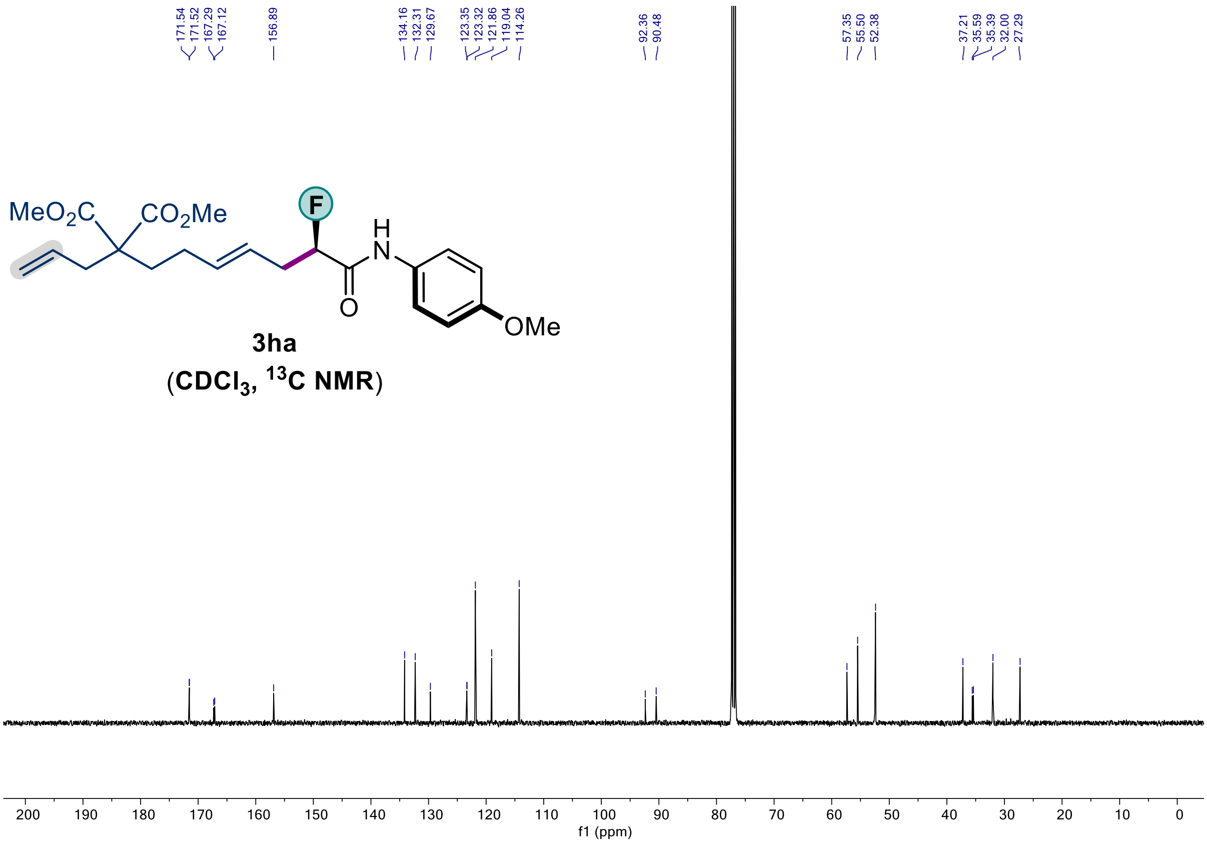
171.54
171.52
167.29
167.12
156.89
134.16
132.31
129.67
123.35
123.32
121.86
119.04
114.26
92.36
90.48
57.35
55.50
52.38
37.21
35.59
35.39
32.00
27.29
MeO2C
CO2Me
F
H
N
O
OMe
3ha
(CDCl3, 13C NMR)
200 190 180 170 160 150 140 130 120 110 100 90 80 70 60 50 40 30 20 10 0
f1 (ppm)

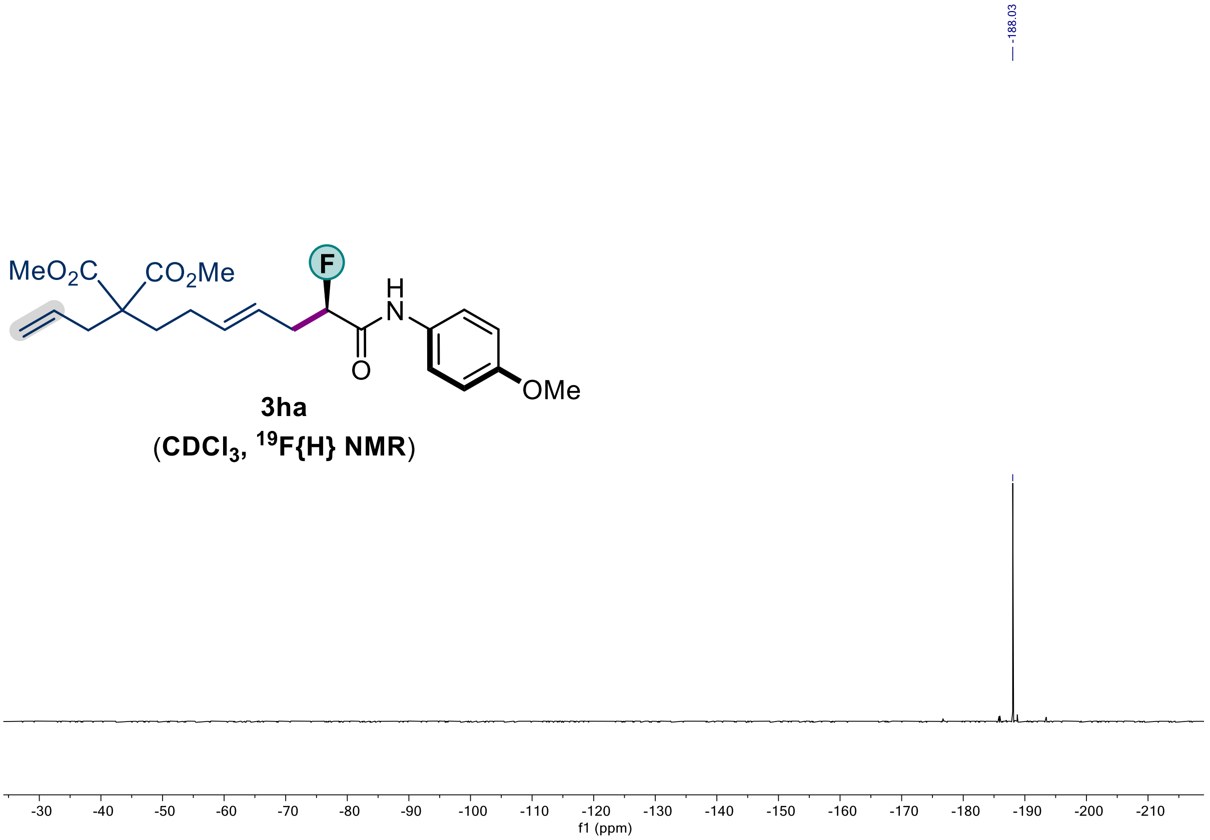
MeO2C
CO2Me
F
H
N
O
OMe
3ha
(CDCl3, 19F{H} NMR)
-188.03
-30 -40 -50 -60 -70 -80 -90 -100 -110 -120 -130 -140 -150 -160 -170 -180 -190 -200 -210
f1 (ppm)

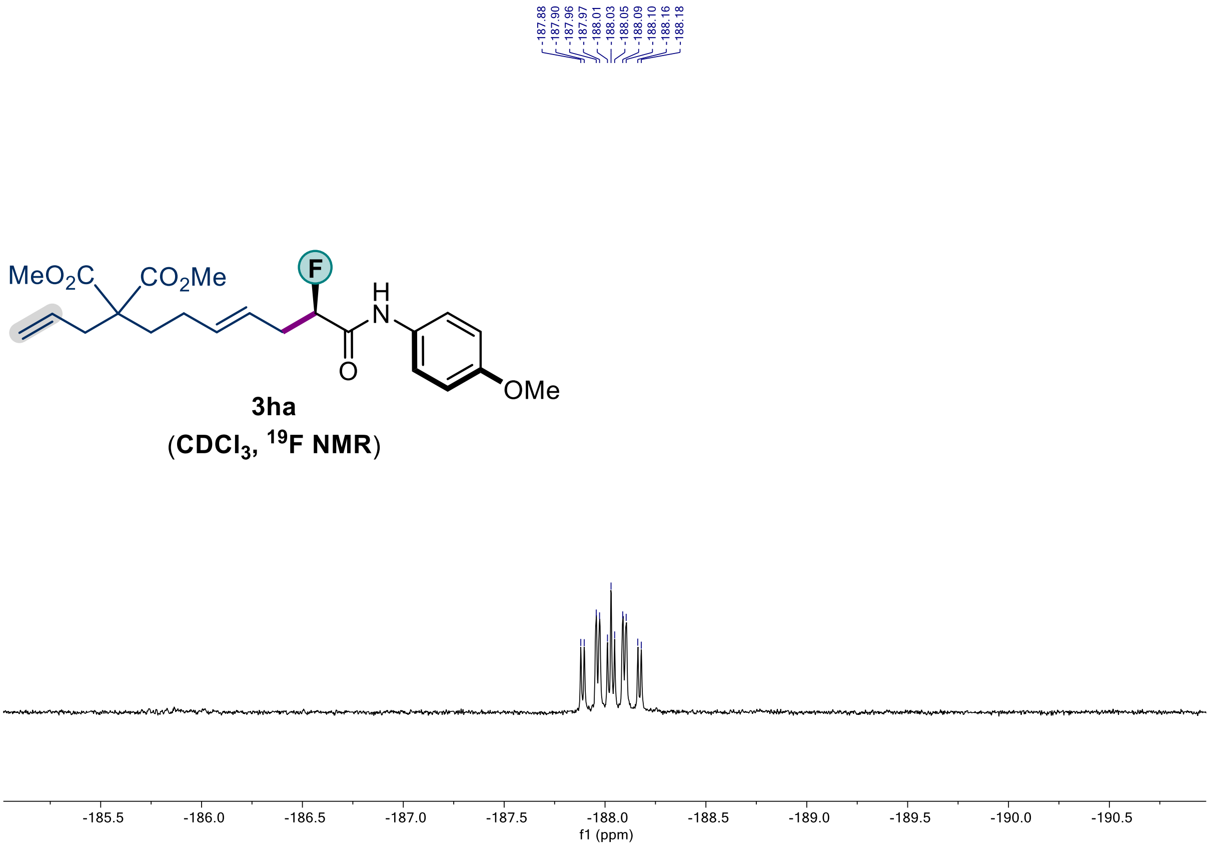
-187.88
-187.90
-187.96
-187.97
-188.01
-188.03
-188.05
-188.09
-188.10
-188.16
-188.18
MeO2C
CO2Me
F
H
N
O
OMe
3ha
(CDCl3, 19F NMR)
-185.5 -186.0 -186.5 -187.0 -187.5 -188.0 -188.5 -189.0 -189.5 -190.0 -190.5
f1 (ppm)

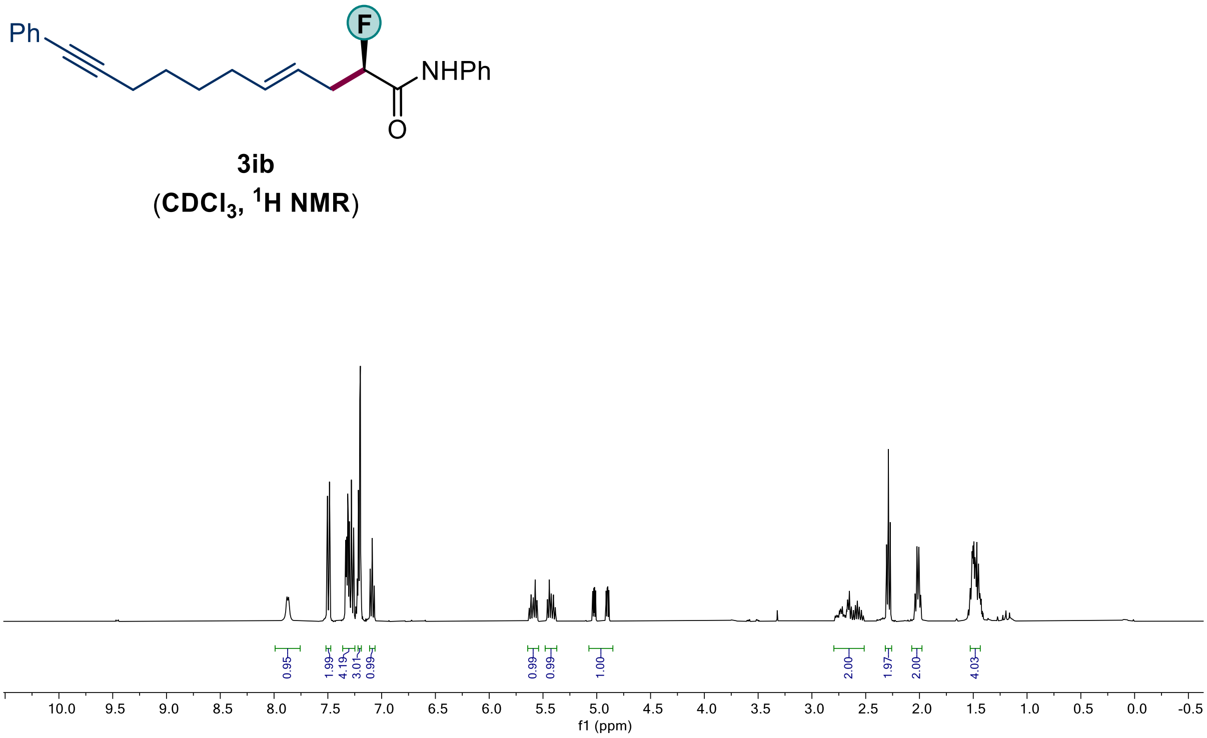
Ph
F
NHPh
O
3ib
(CDCl3, 1H NMR)
0.95
1.99
4.19
3.01
0.99
0.99
0.99
1.00
2.00
1.97
2.00
4.03
f1 (ppm)

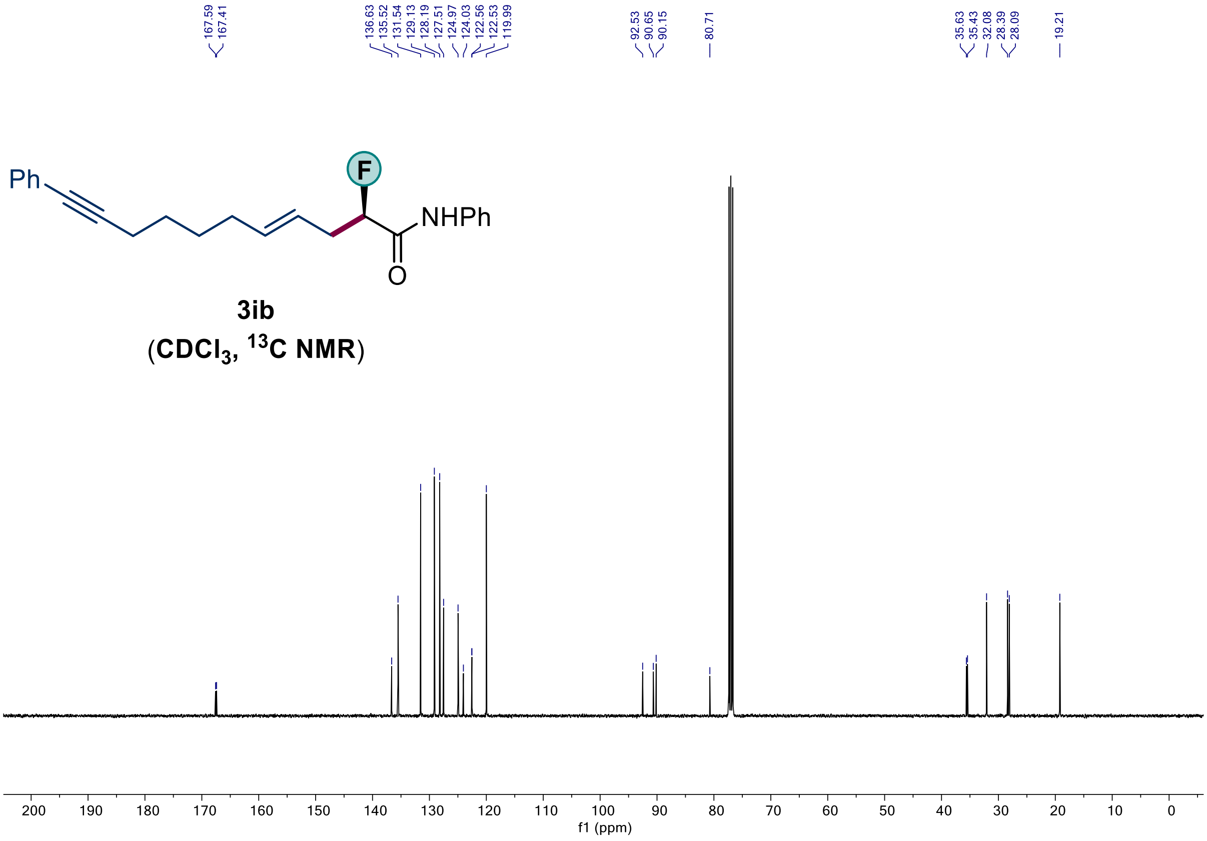
167.59
167.41
136.63
135.52
131.54
129.13
128.19
127.51
124.97
124.03
122.56
122.53
119.99
92.53
90.65
90.15
80.71
35.63
35.43
32.08
28.39
28.09
19.21
Ph
F
NHPh
O
3ib
(CDCl3, 13C NMR)
f1 (ppm)

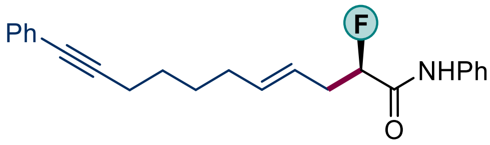

3ib
(**$CDCl_3$, $^{19}F\{1H\}$ NMR**)

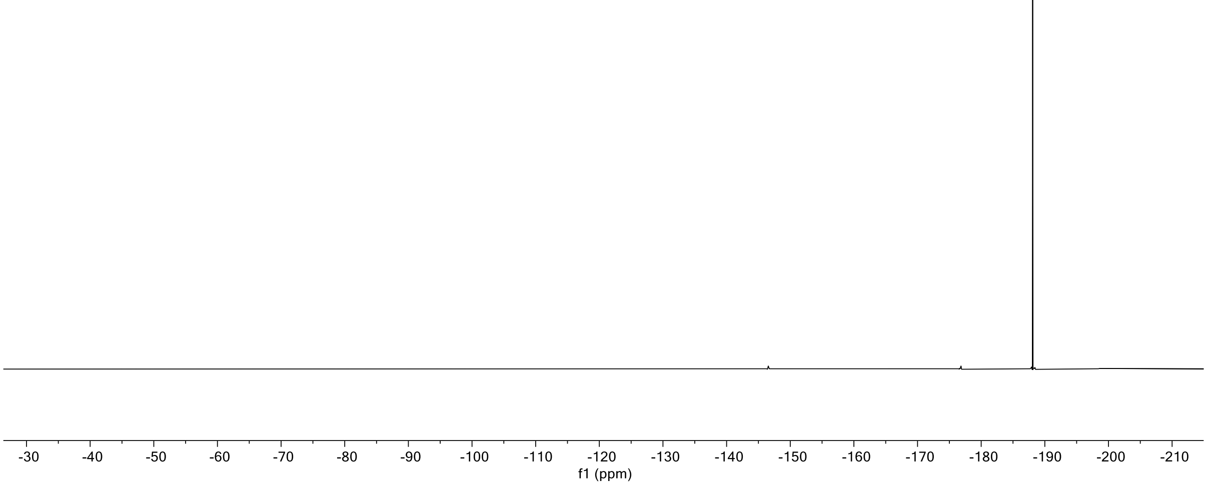

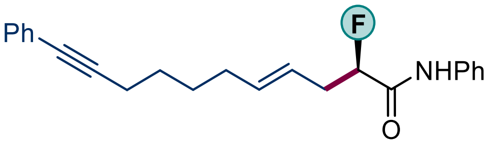

3ib
(**$CDCl_3$, ^{19}F NMR**)

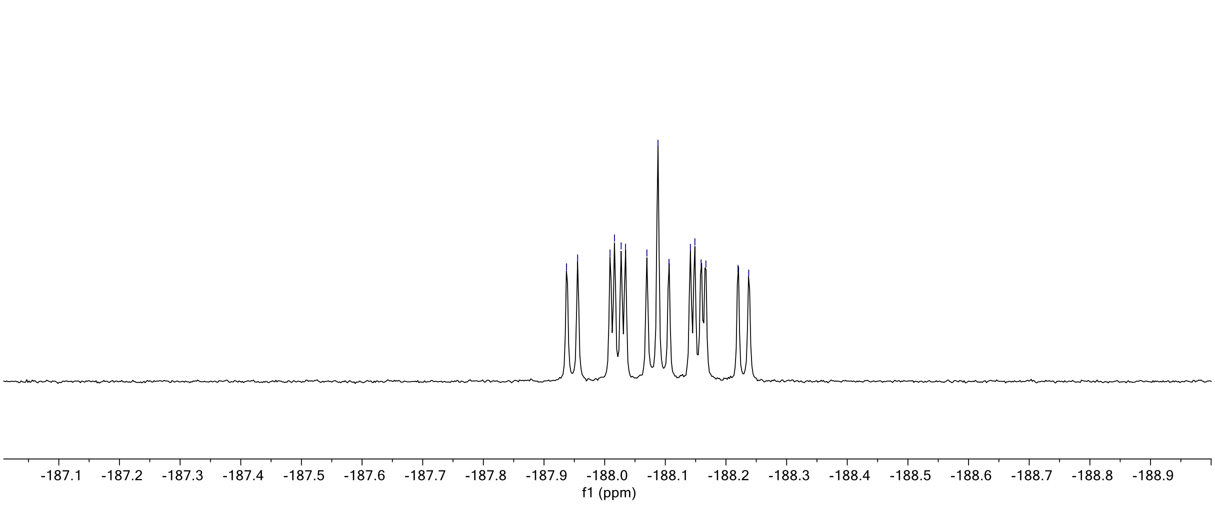

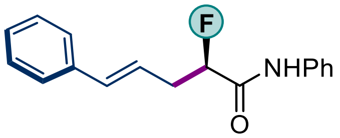

3jb
(**$CDCl_3$, ^{1}H NMR**)

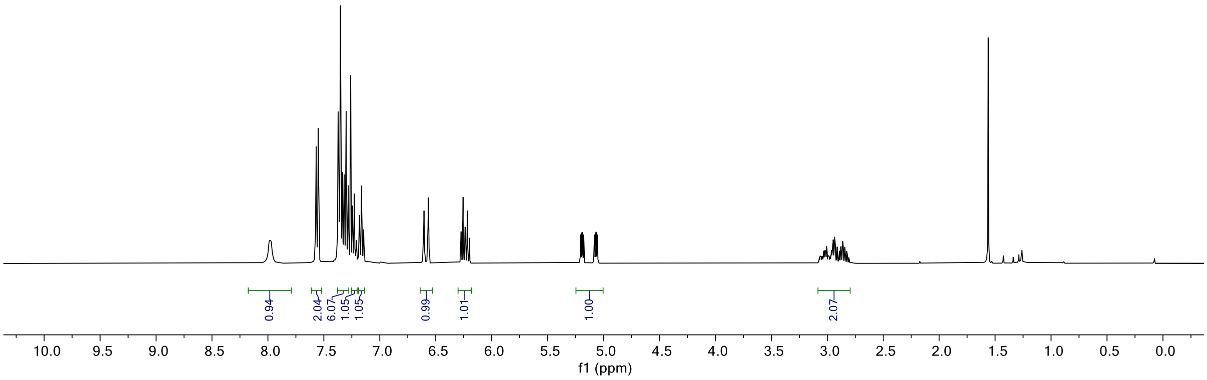

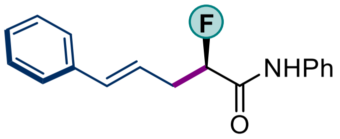

3jb
(**$CDCl_3$, ^{13}C NMR**)

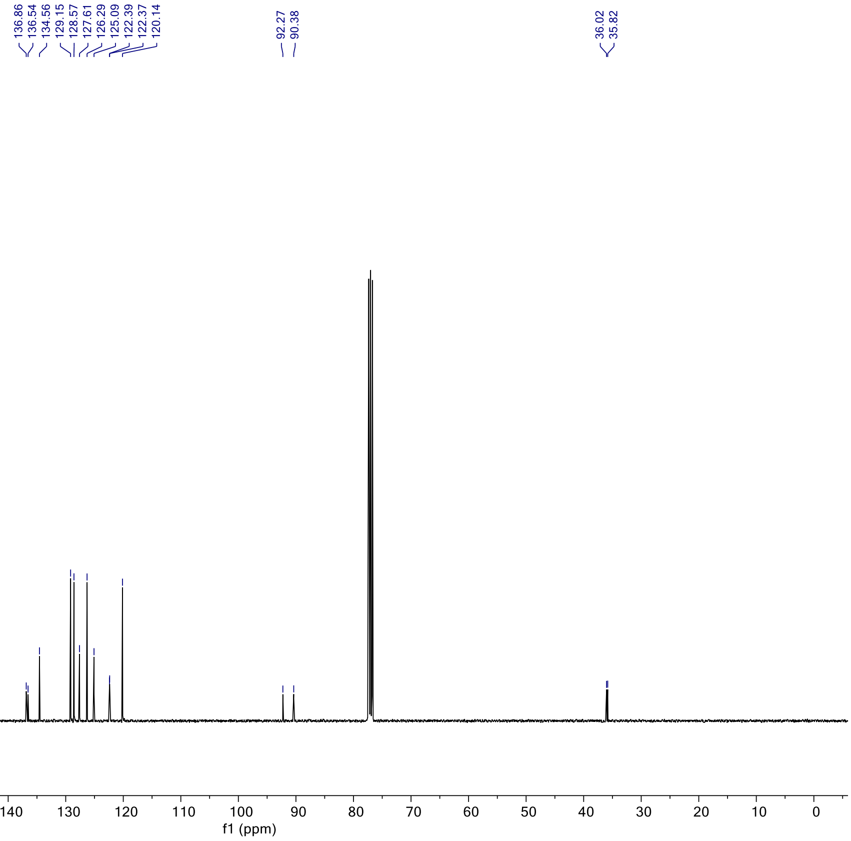

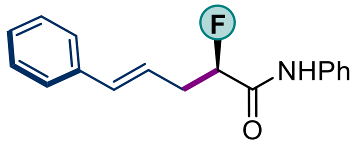

3jb
(**CDCl$_3$, ^{19}F{H} NMR**)

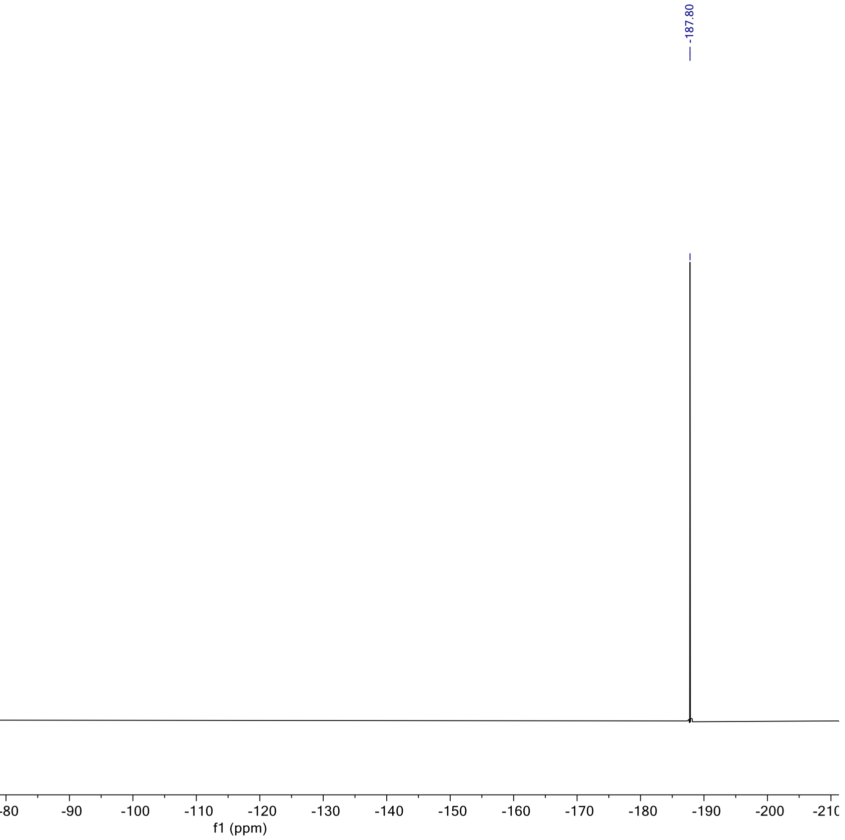

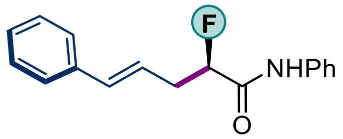

3jb
(**CDCl$_3$, ^{19}F NMR**)

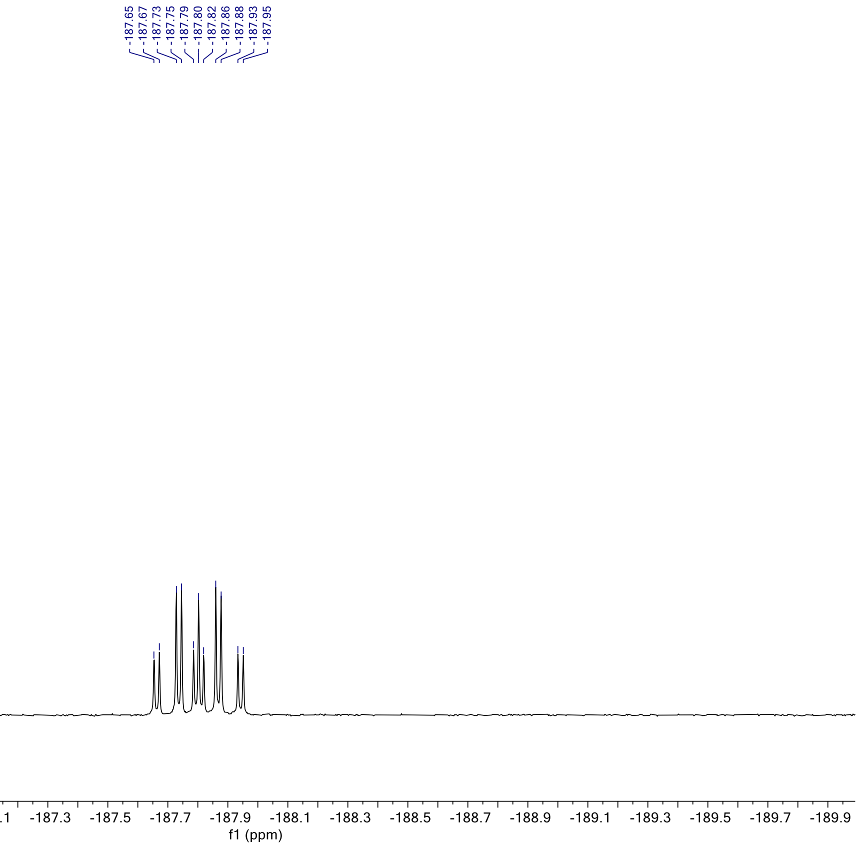

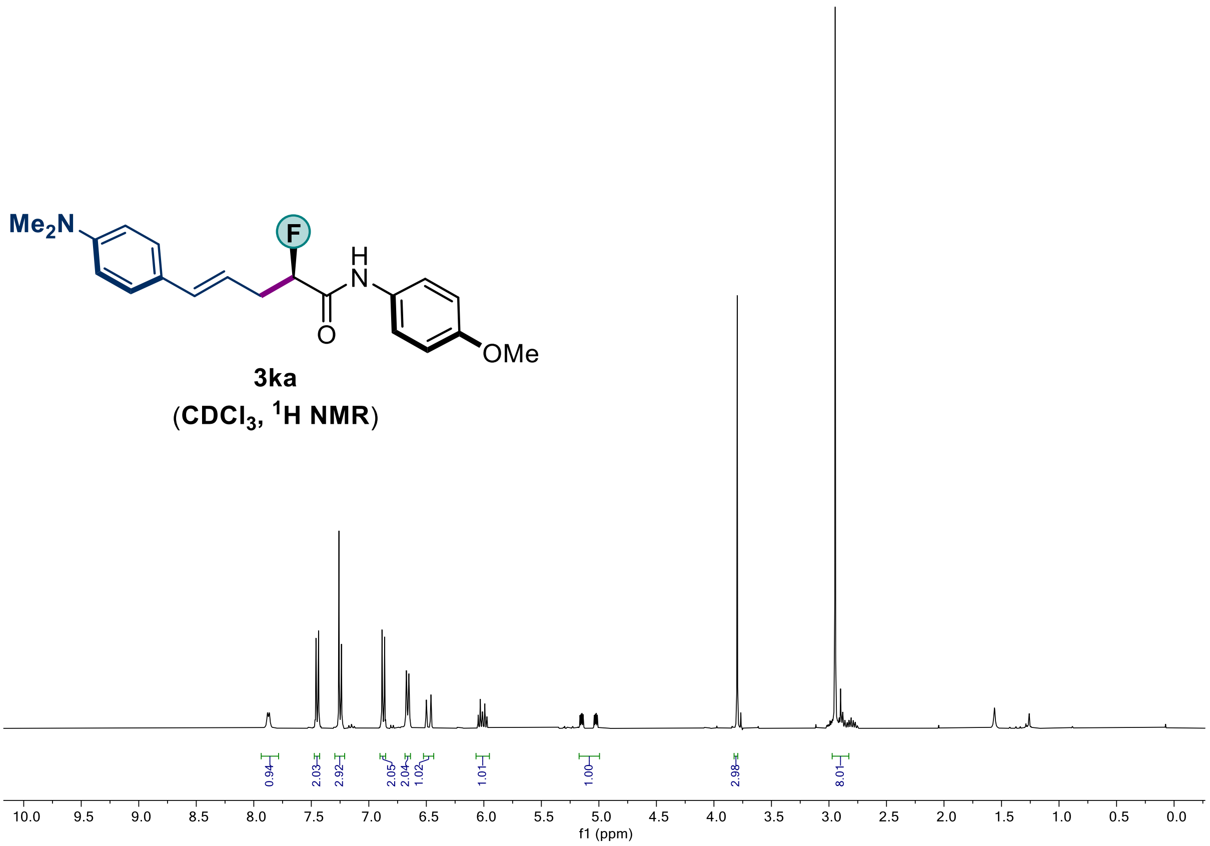
Me2N
F
H
N
O
OMe
3ka
(CDCl3, 1H NMR)
0.94
2.03
2.92
2.05
2.04
1.02
1.01
1.00
2.98
8.01
10.0 9.5 9.0 8.5 8.0 7.5 7.0 6.5 6.0 5.5 5.0 4.5 4.0 3.5 3.0 2.5 2.0 1.5 1.0 0.5 0.0
f1 (ppm)

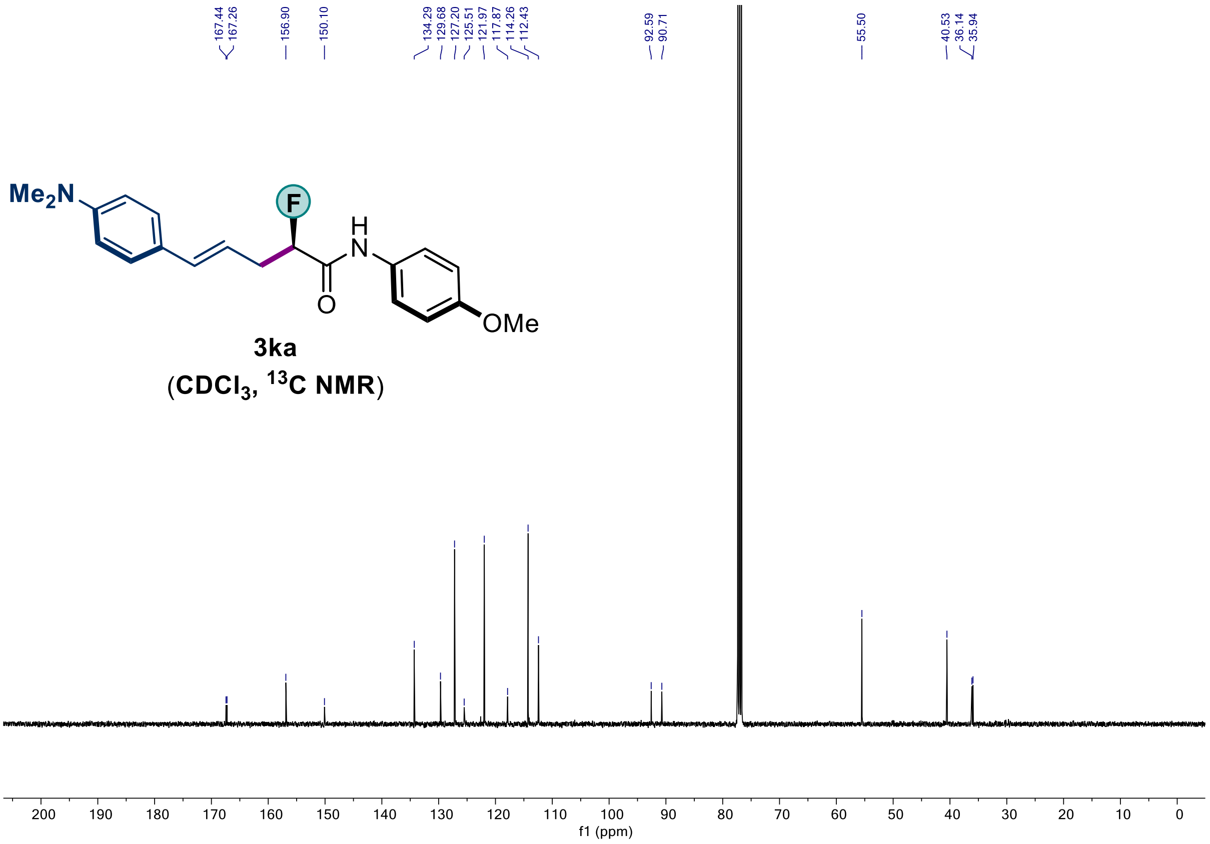
167.44
167.26
156.90
150.10
134.29
129.68
127.20
125.51
121.97
117.87
114.26
112.43
92.59
90.71
55.50
40.53
36.14
35.94
Me2N
F
H
N
O
OMe
3ka
(CDCl3, 13C NMR)
200 190 180 170 160 150 140 130 120 110 100 90 80 70 60 50 40 30 20 10 0
f1 (ppm)

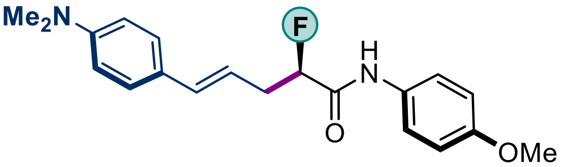

3ka
(**CDCl$_3$, ^{19}F{H} NMR**)

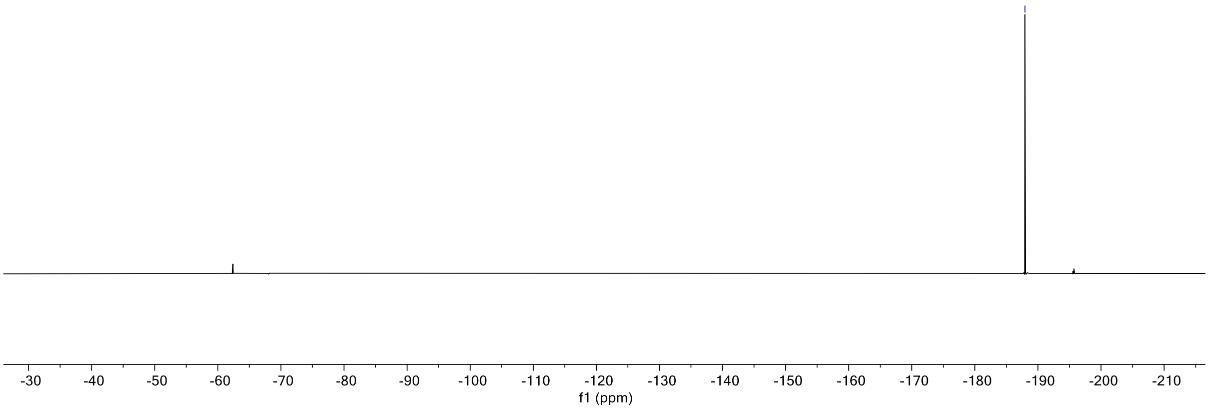

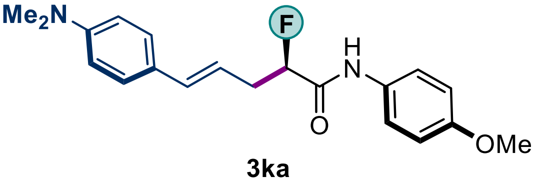

3ka
(**CDCl$_3$, ^{19}F NMR**)

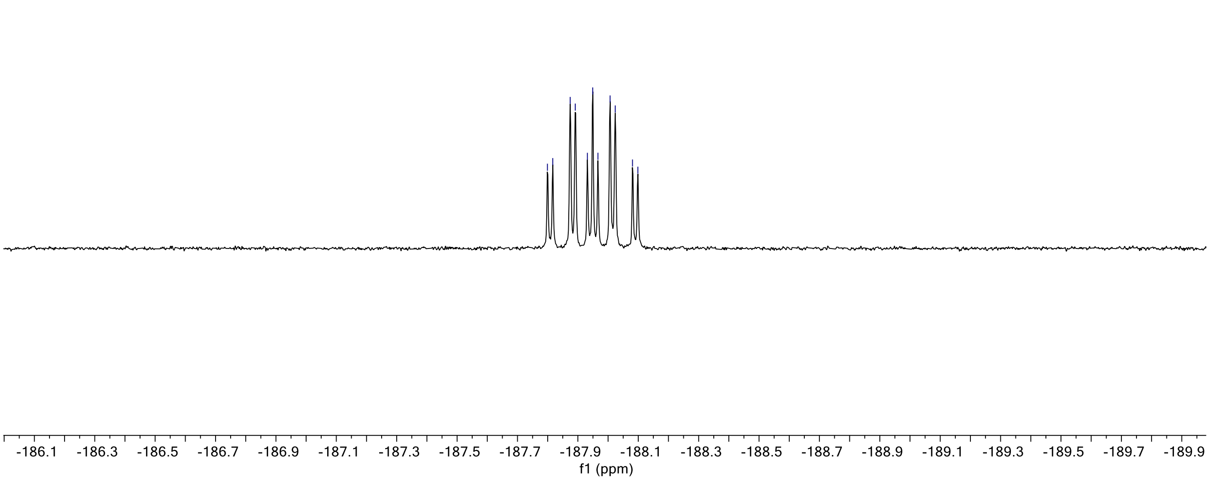

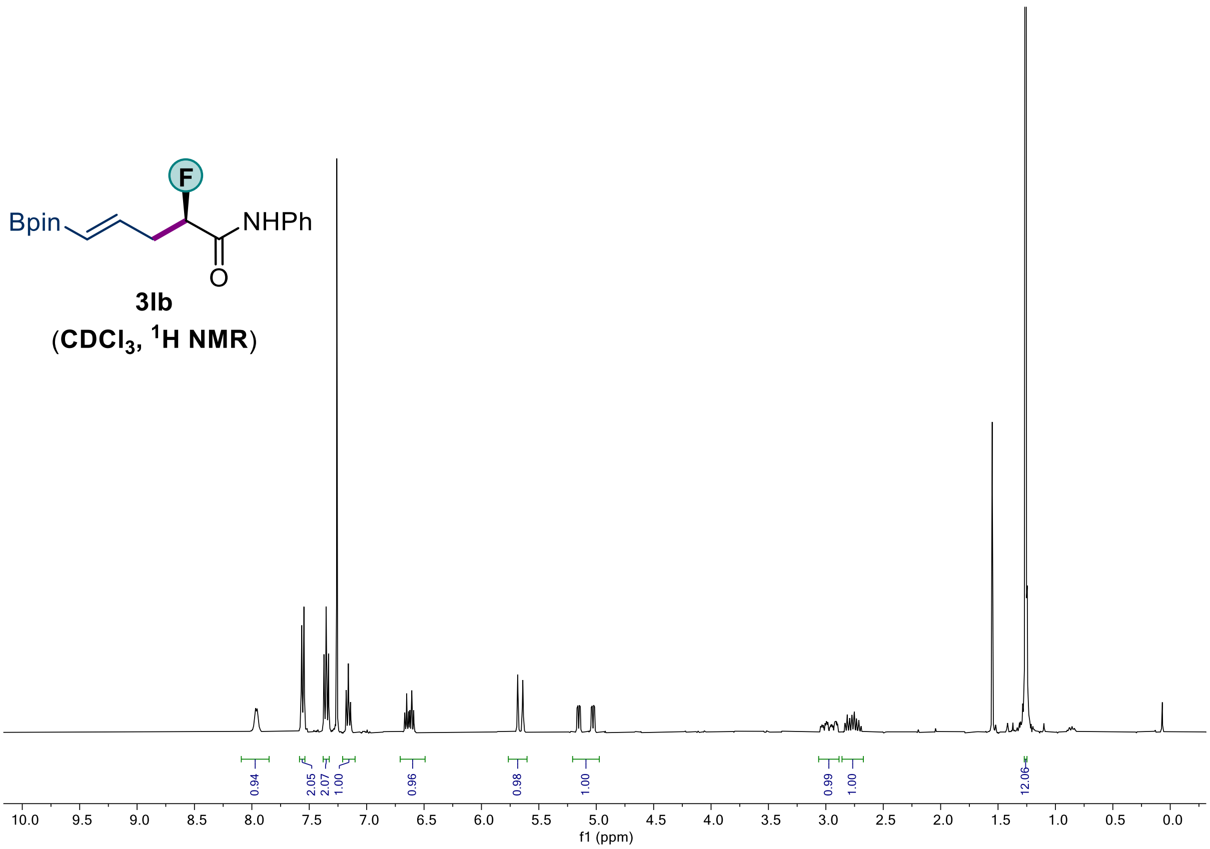
Bpin
F
NHPh
O
3lb
(CDCl3, 1H NMR)
0.94
2.05
2.07
1.00
0.96
0.98
1.00
0.99
1.00
12.06
10.0 9.5 9.0 8.5 8.0 7.5 7.0 6.5 6.0 5.5 5.0 4.5 4.0 3.5 3.0 2.5 2.0 1.5 1.0 0.5 0.0
f1 (ppm)

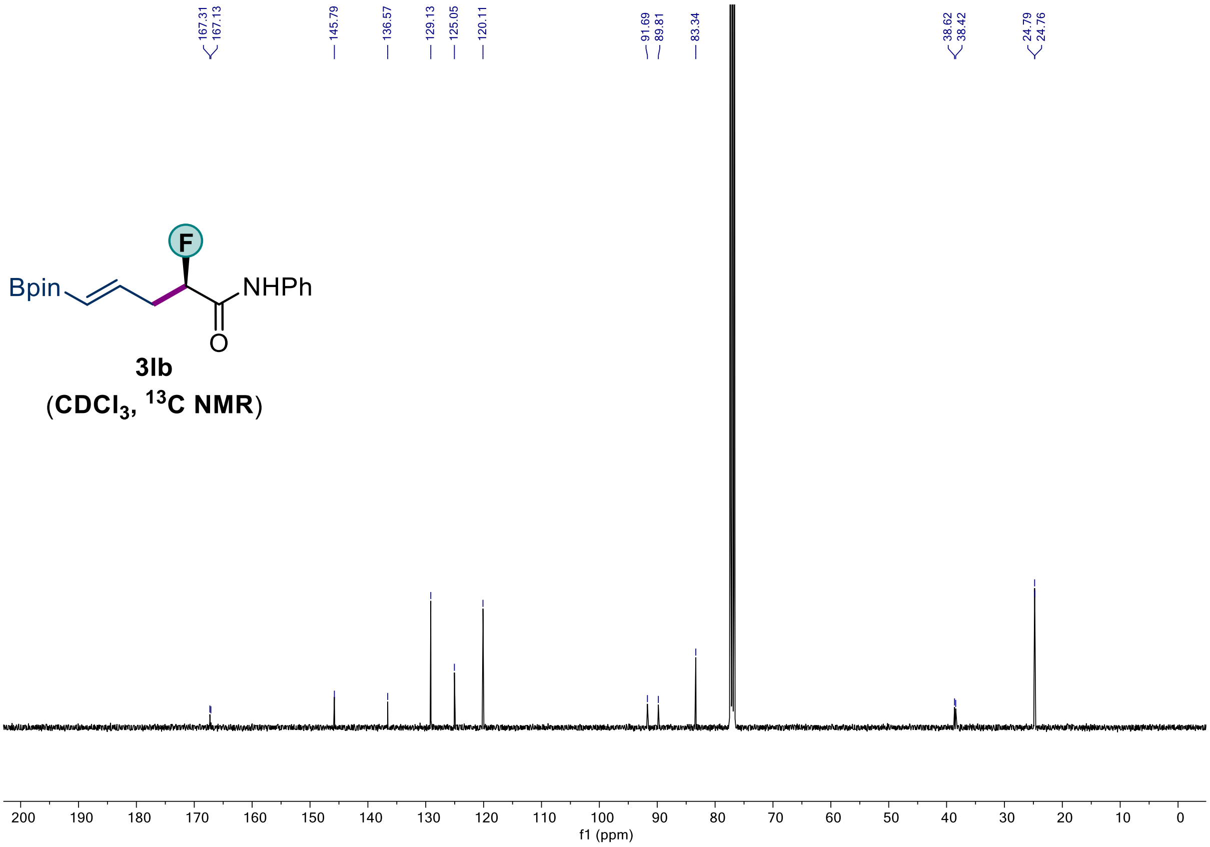
167.31
167.13
145.79
136.57
129.13
125.05
120.11
91.69
89.81
83.34
38.62
38.42
24.79
24.76
Bpin
F
NHPh
O
3lb
(CDCl3, 13C NMR)
200 190 180 170 160 150 140 130 120 110 100 90 80 70 60 50 40 30 20 10 0
f1 (ppm)

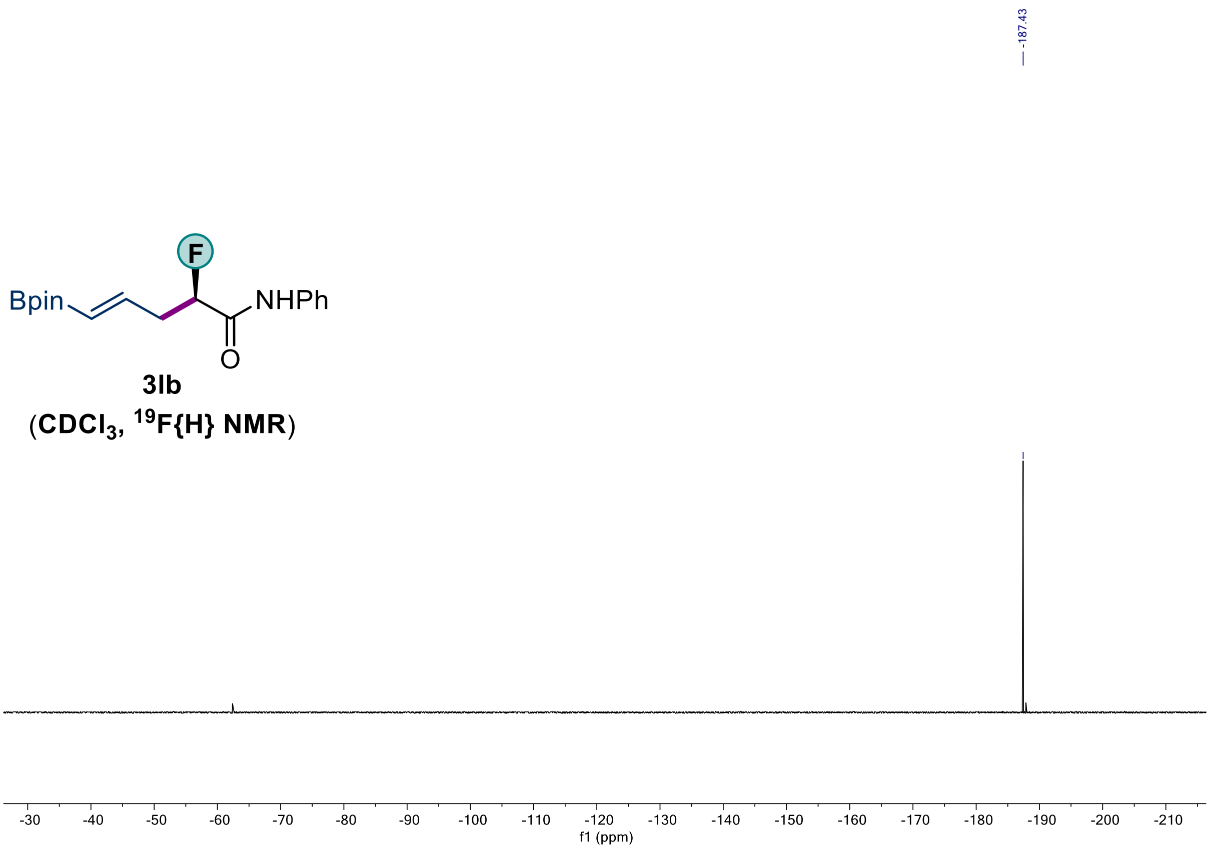

-187.43
F
Bpin
NHPh
O
3lb
(CDCl3, 19F{H} NMR)
-30 -40 -50 -60 -70 -80 -90 -100 -110 -120 -130 -140 -150 -160 -170 -180 -190 -200 -210
f1 (ppm)

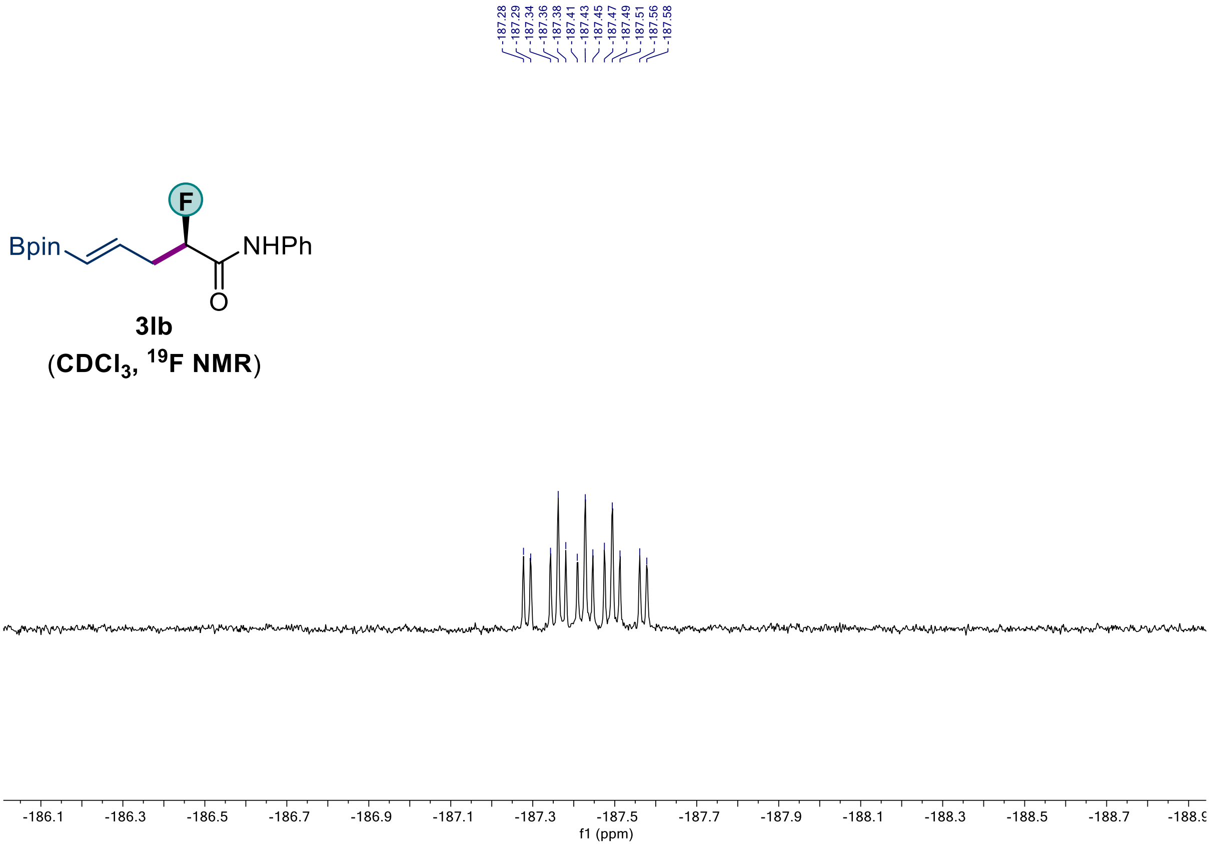

-187.28
-187.29
-187.34
-187.36
-187.38
-187.41
-187.43
-187.45
-187.47
-187.49
-187.51
-187.56
-187.58
F
Bpin
NHPh
O
3lb
(CDCl3, 19F NMR)
-186.1 -186.3 -186.5 -186.7 -186.9 -187.1 -187.3 -187.5 -187.7 -187.9 -188.1 -188.3 -188.5 -188.7
f1 (ppm)

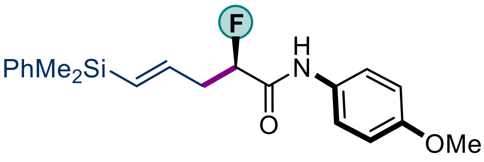

3ma

(CDCl$_3$, ^{1}H NMR)

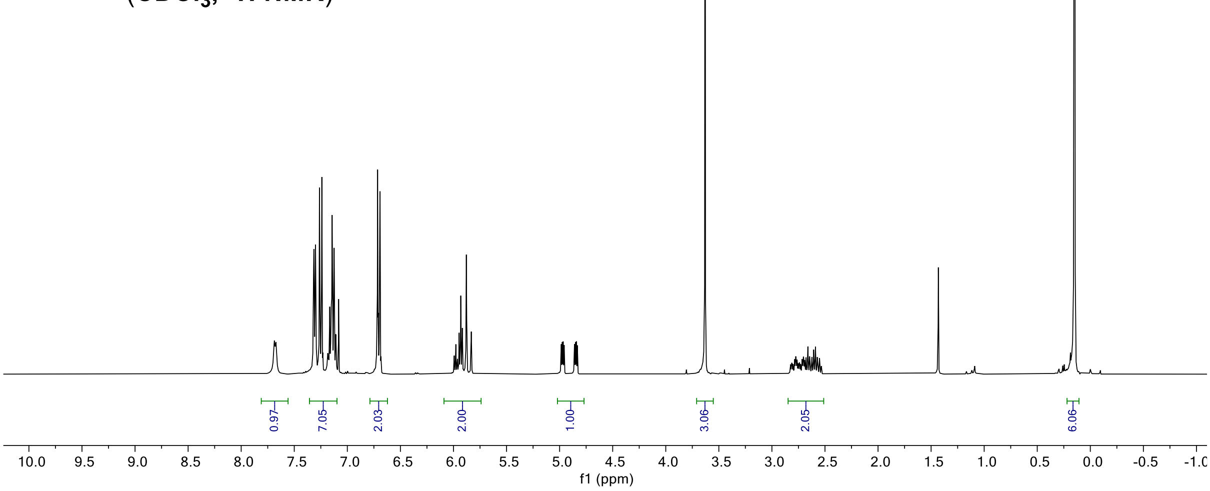

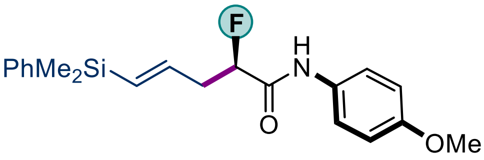

3ma

(CDCl$_3$, ^{13}C NMR)

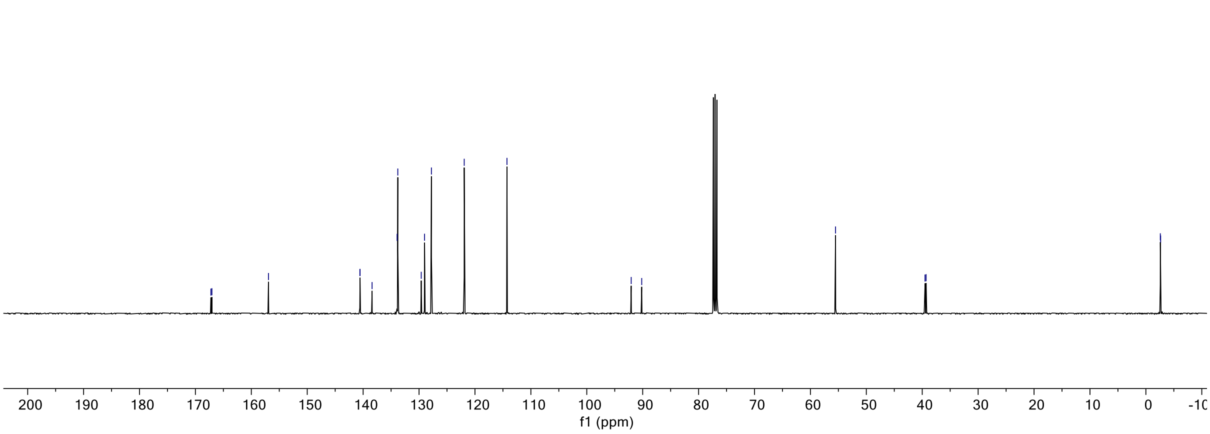

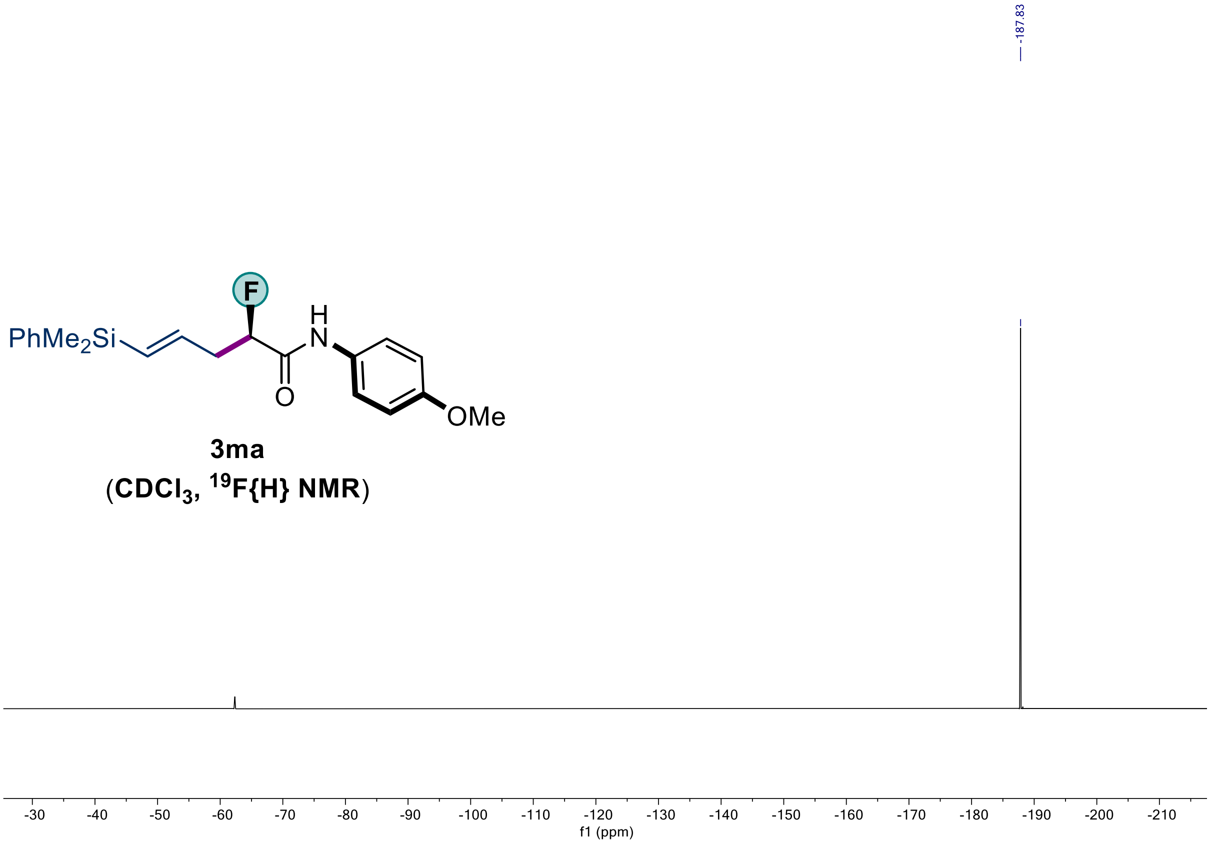
-187.83
PhMe2Si
F
H
N
O
OMe
3ma
(CDCl3, 19F{H} NMR)
-30
-40
-50
-60
-70
-80
-90
-100
-110
-120
-130
-140
-150
-160
-170
-180
-190
-200
-210
f1 (ppm)

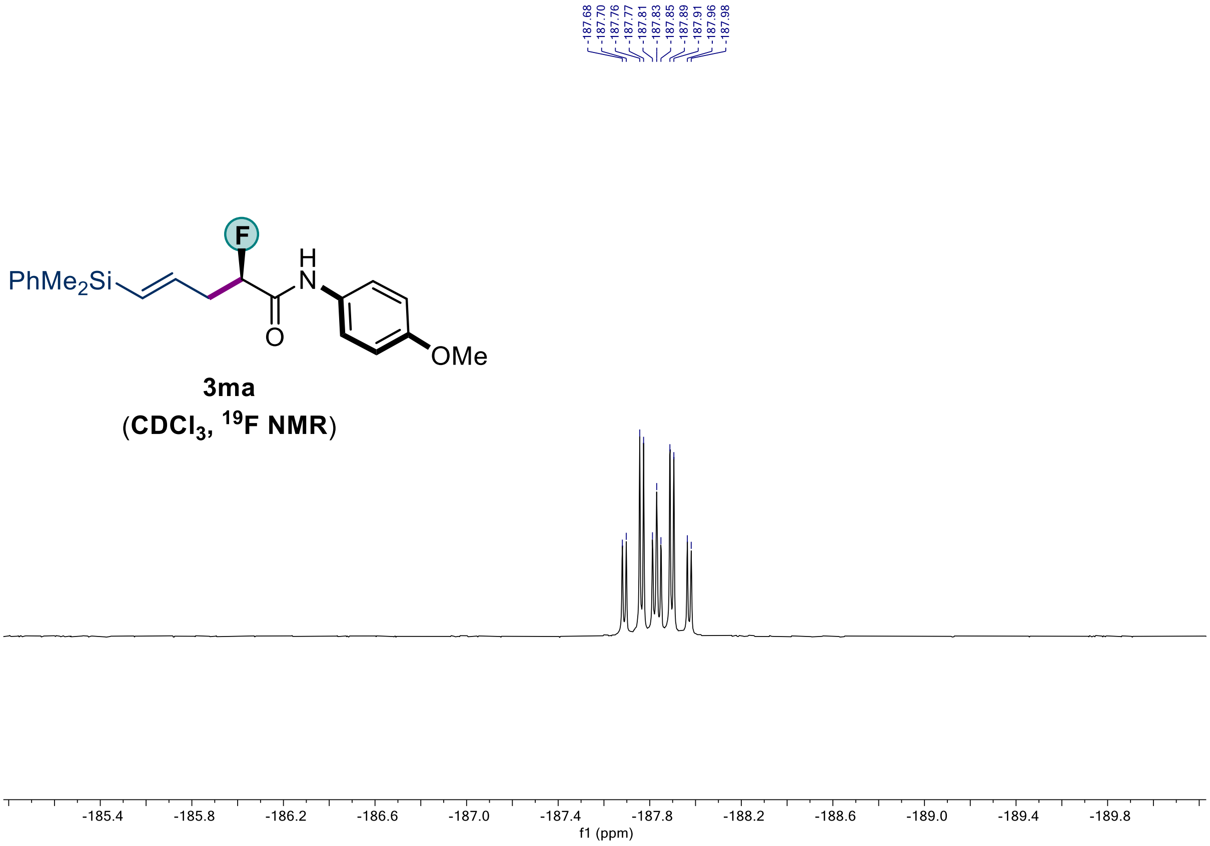
-187.68
-187.70
-187.76
-187.77
-187.81
-187.83
-187.85
-187.89
-187.91
-187.96
-187.98
PhMe2Si
F
H
N
O
OMe
3ma
(CDCl3, 19F NMR)
-185.4
-185.8
-186.2
-186.6
-187.0
-187.4
-187.8
-188.2
-188.6
-189.0
-189.4
-189.8
f1 (ppm)

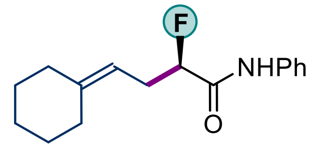

3nb
($CDCl_3$, ^1H NMR)

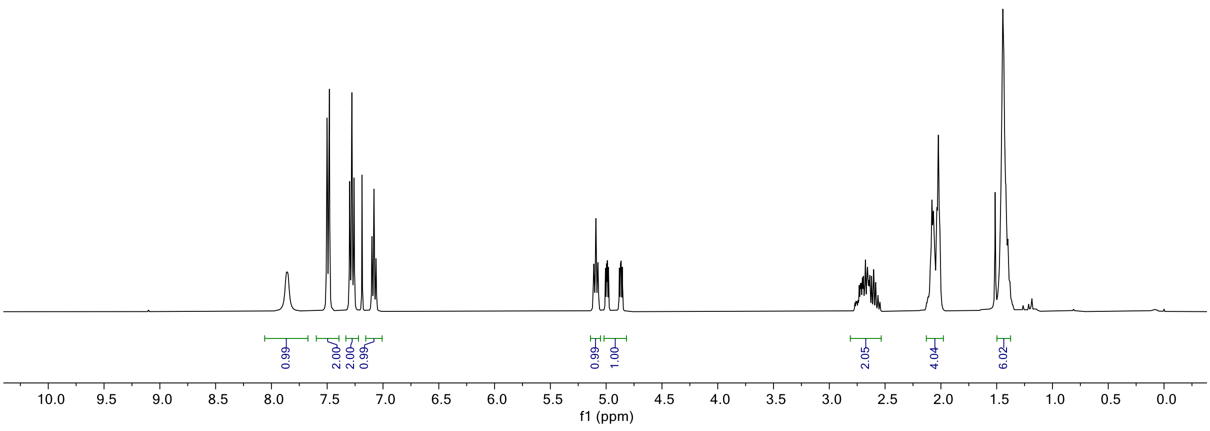

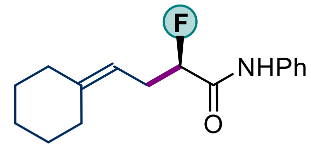

3nb
($CDCl_3$, ^{13}C NMR)

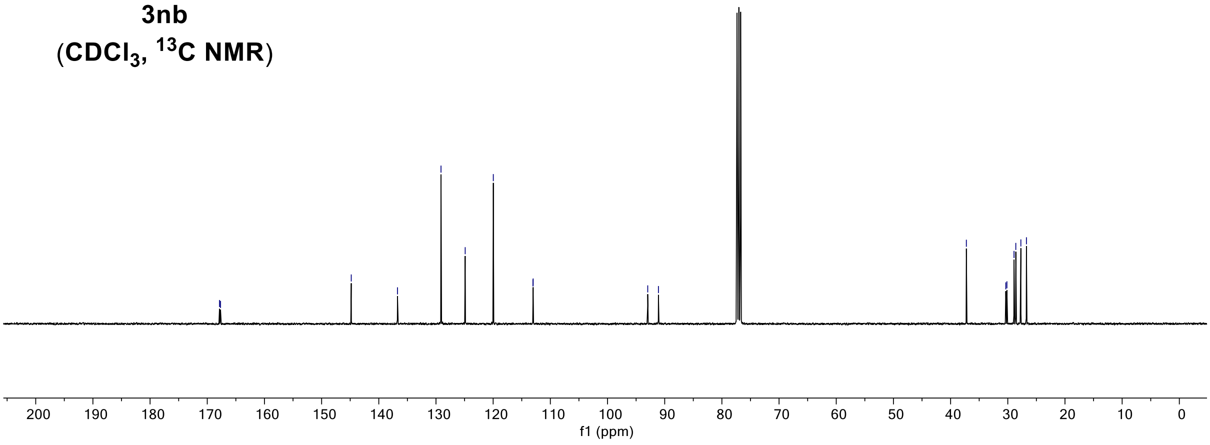

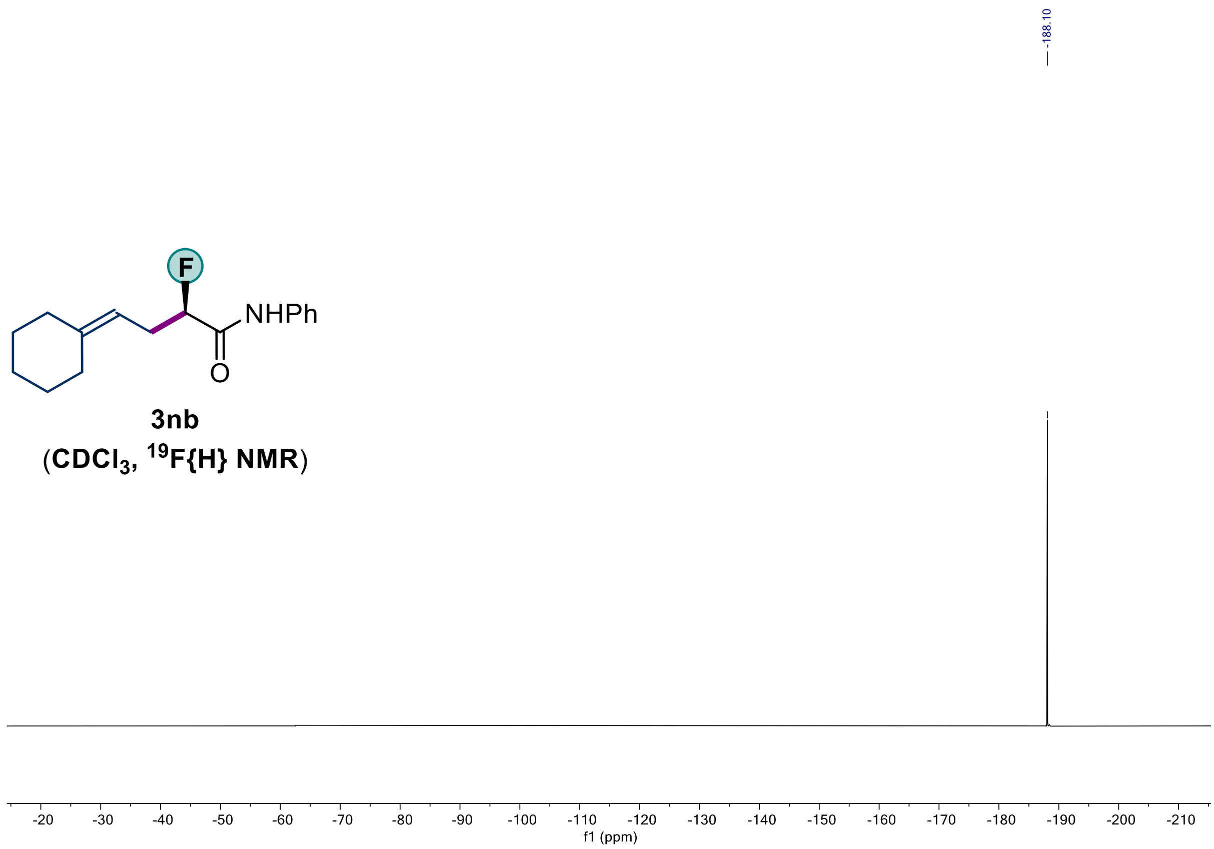
-188.10
NHPh
O
F
3nb
(CDCl3, 19F{H} NMR)
-20 -30 -40 -50 -60 -70 -80 -90 -100 -110 -120 -130 -140 -150 -160 -170 -180 -190 -200 -210
f1 (ppm)

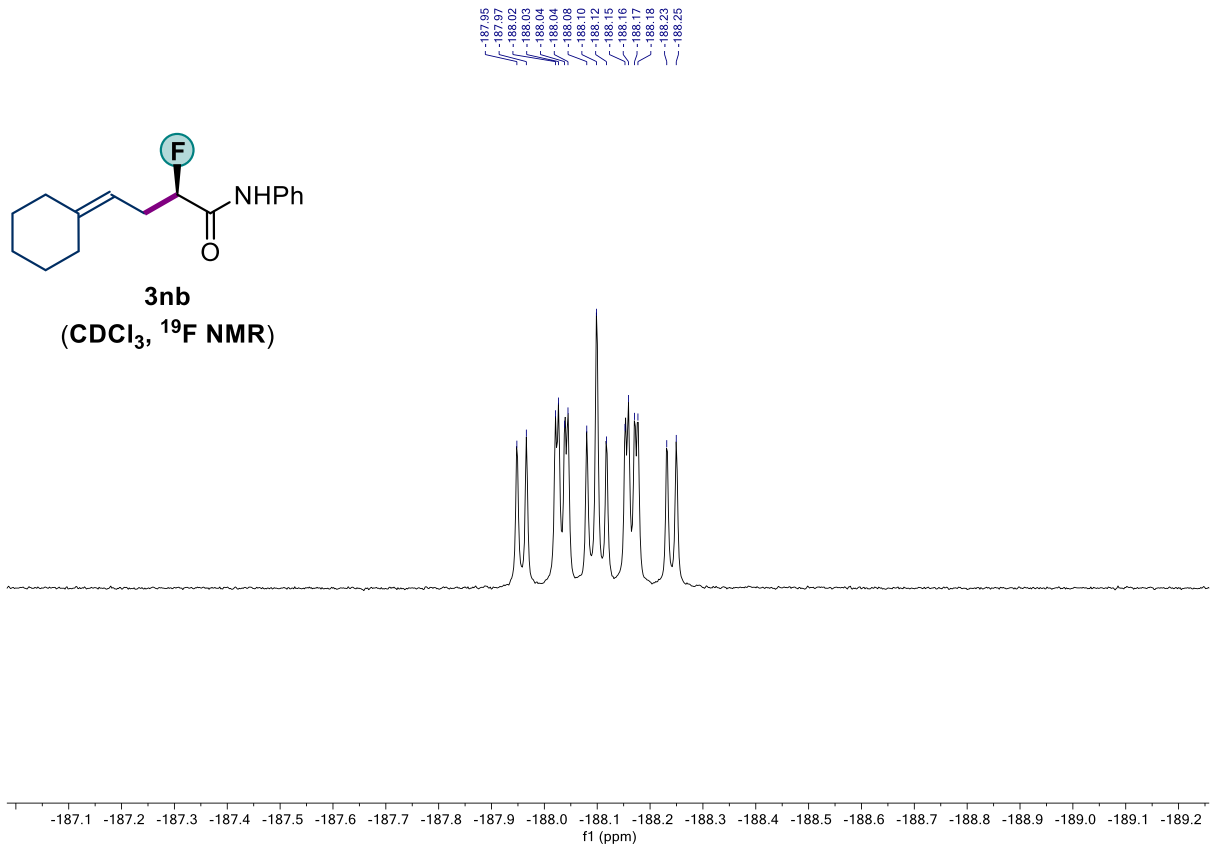
-187.95
-187.97
-188.02
-188.03
-188.04
-188.04
-188.08
-188.10
-188.12
-188.15
-188.16
-188.17
-188.18
-188.23
-188.25
F
NHPh
O
3nb
(CDCl3, 19F NMR)
-187.1 -187.2 -187.3 -187.4 -187.5 -187.6 -187.7 -187.8 -187.9 -188.0 -188.1 -188.2 -188.3 -188.4 -188.5 -188.6 -188.7 -188.8 -188.9 -189.0 -189.1 -189.2
f1 (ppm)

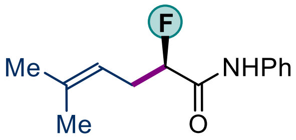

3ob
(**$CDCl_3$, ^{1}H NMR**)

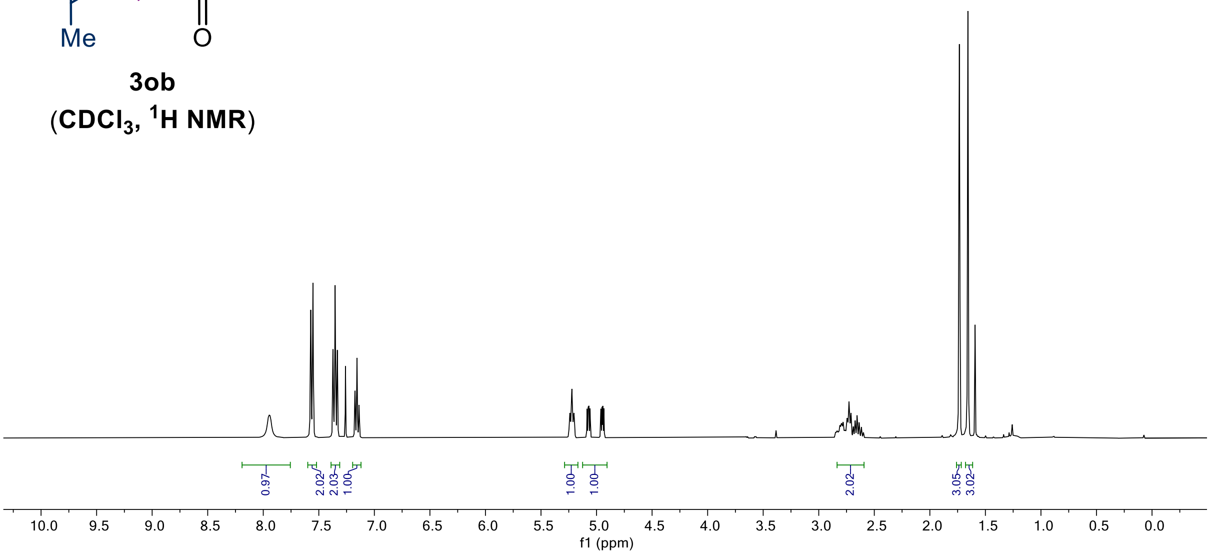

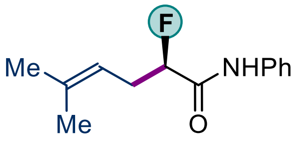

3ob
(**$CDCl_3$, ^{13}C NMR**)

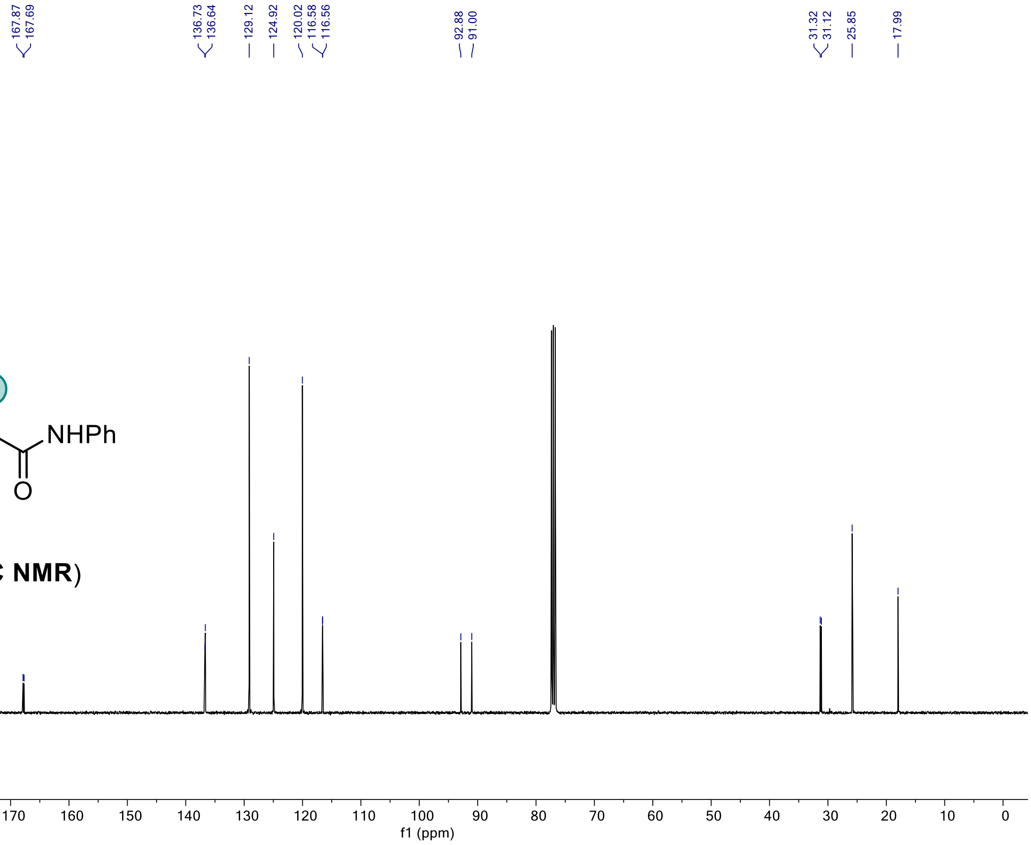

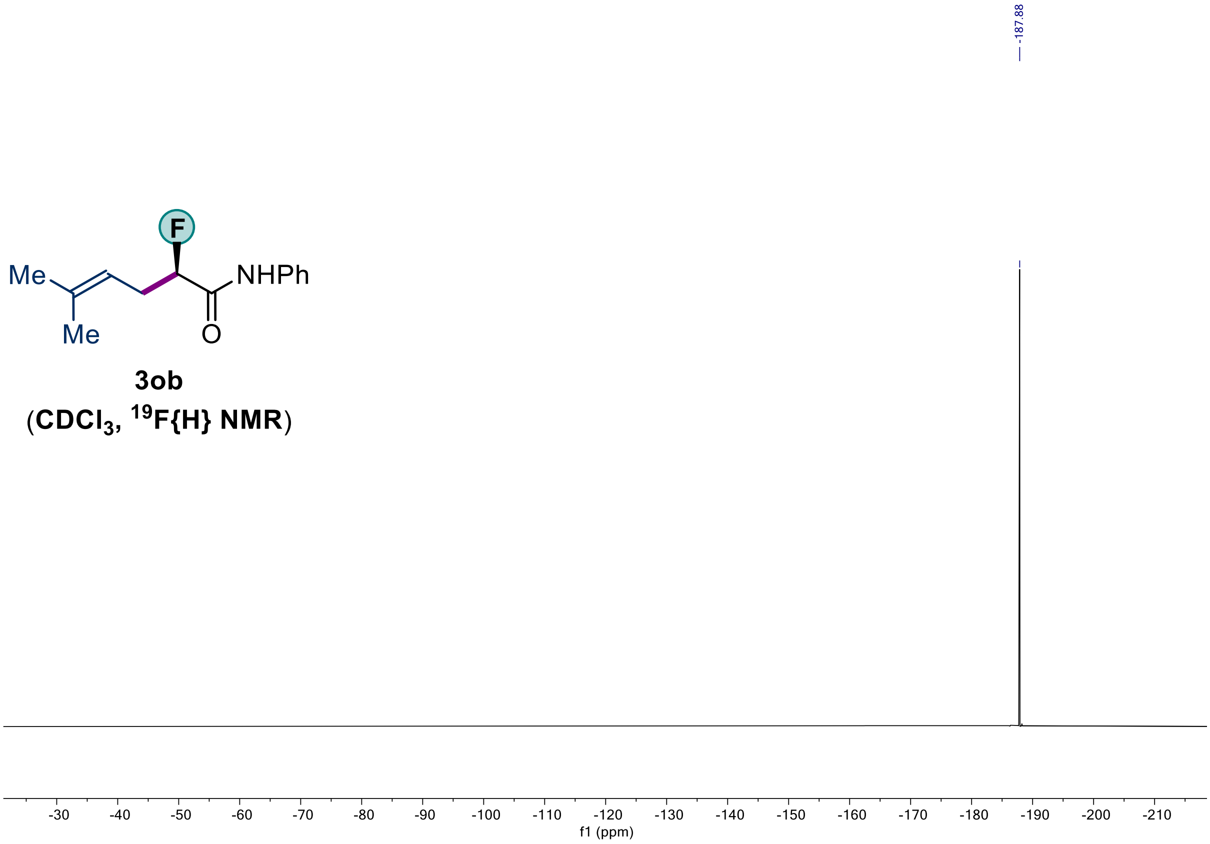

3ob
(**CDCl$_3$, ^{19}F{H} NMR**)

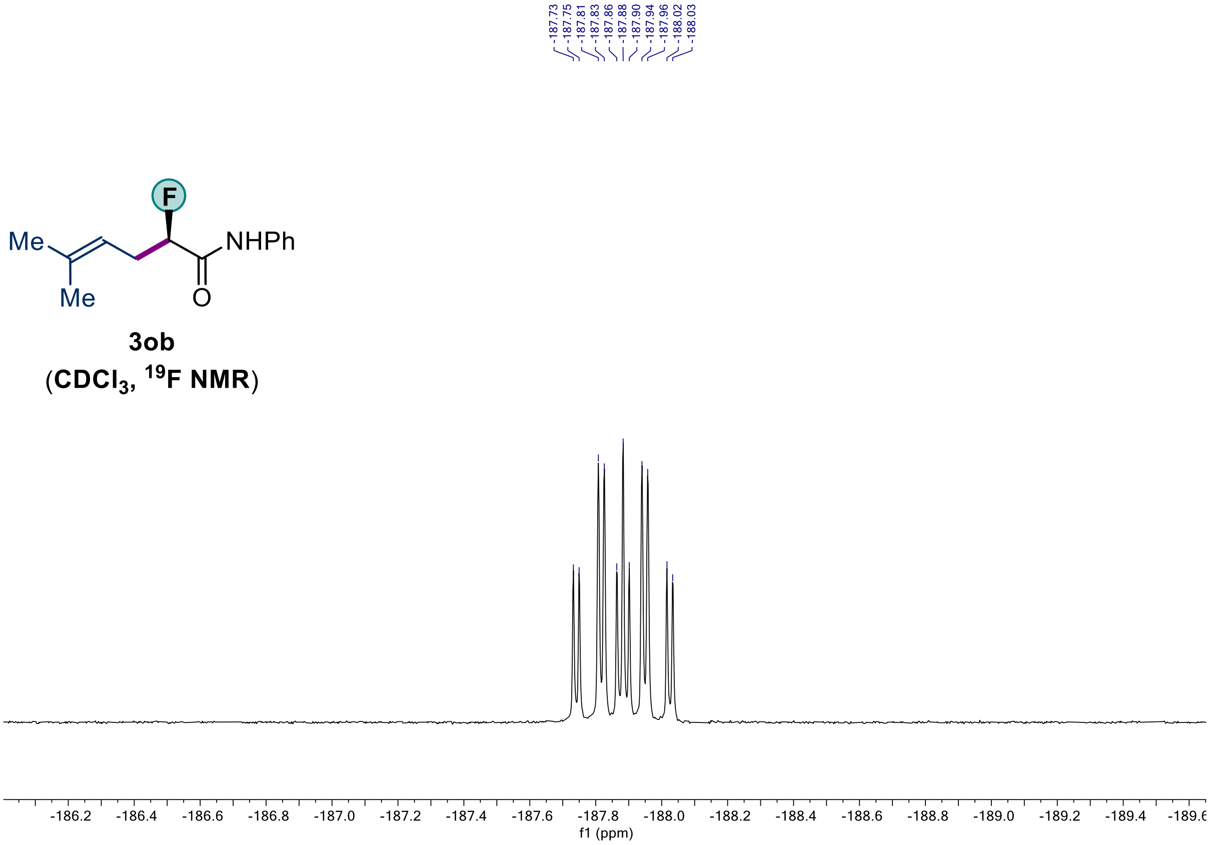

3ob
(**CDCl$_3$, ^{19}F NMR**)

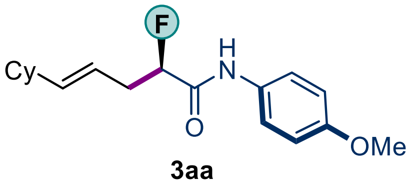

3aa
($CDCl_3$, ^1H NMR)

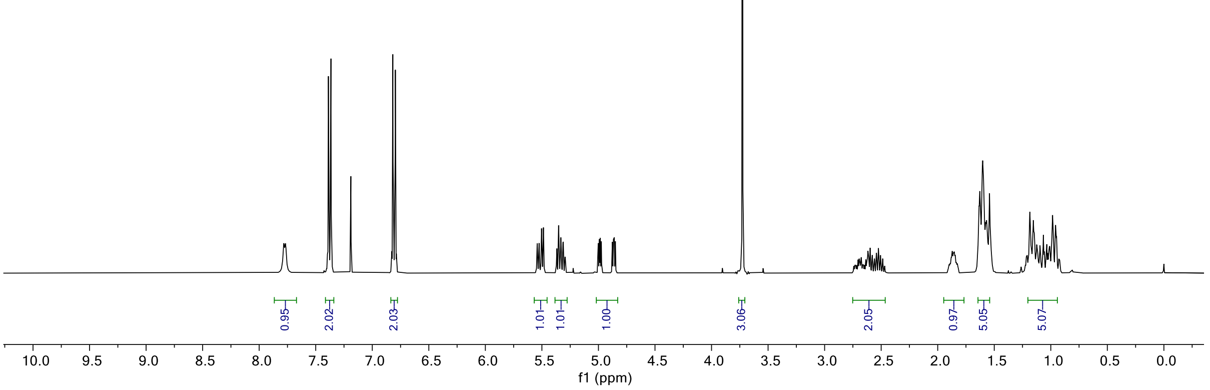

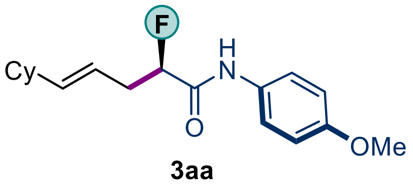

3aa
($CDCl_3$, ^{13}C NMR)

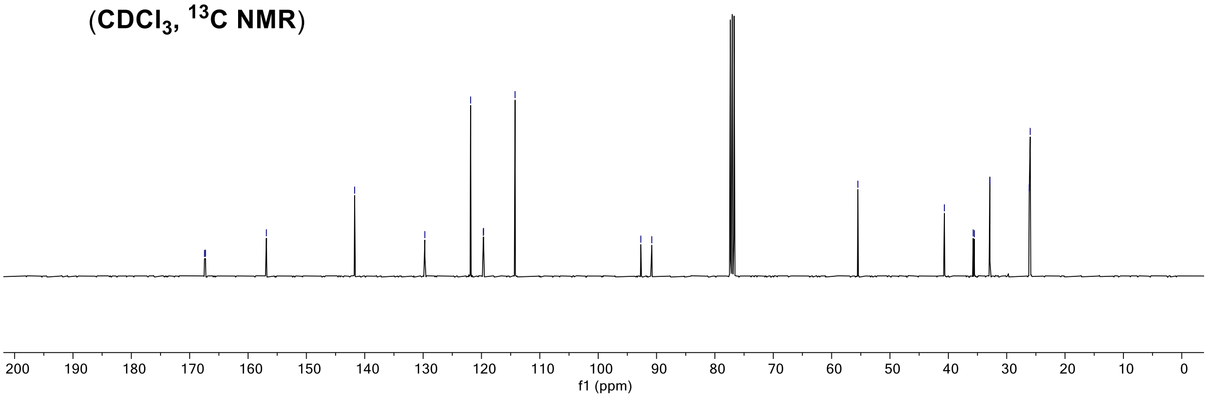

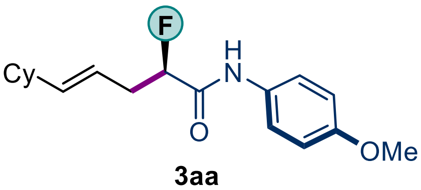

3aa

(**$CDCl_3$, $^{19}F\{H\}$ NMR**)

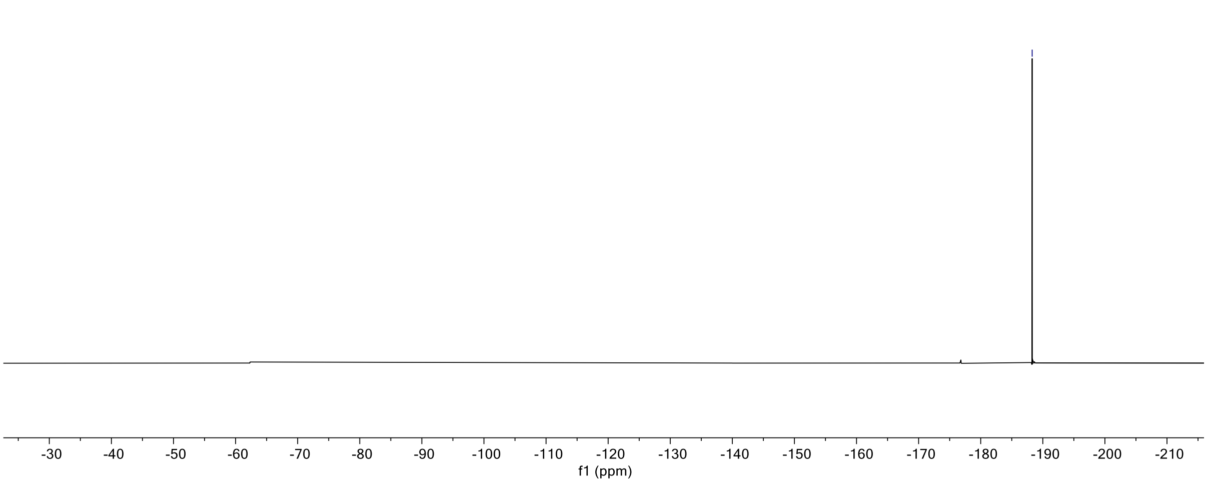

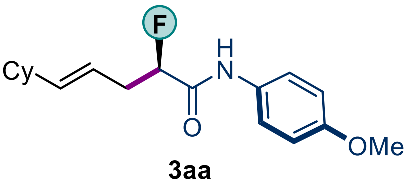

3aa

(**$CDCl_3$, ^{19}F NMR**)

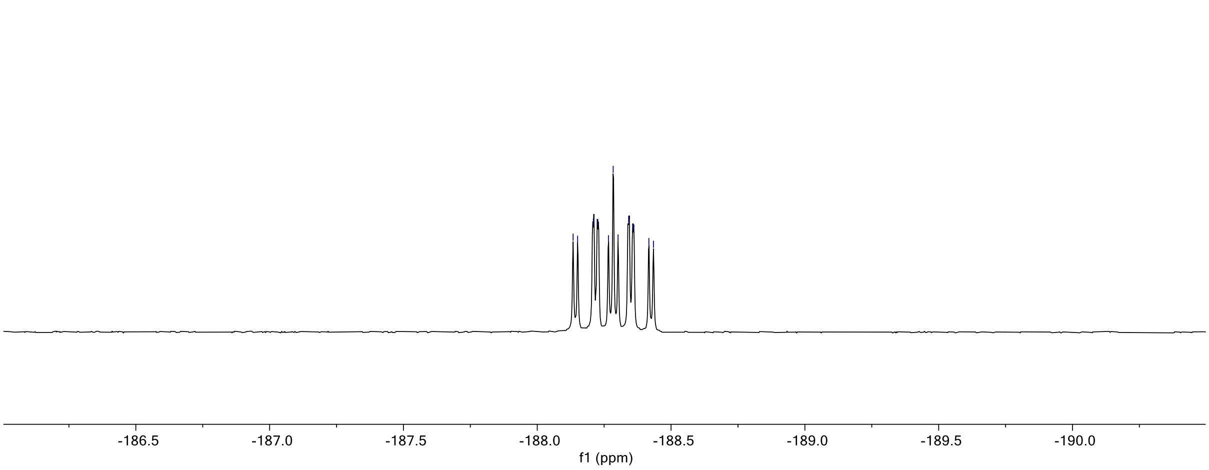

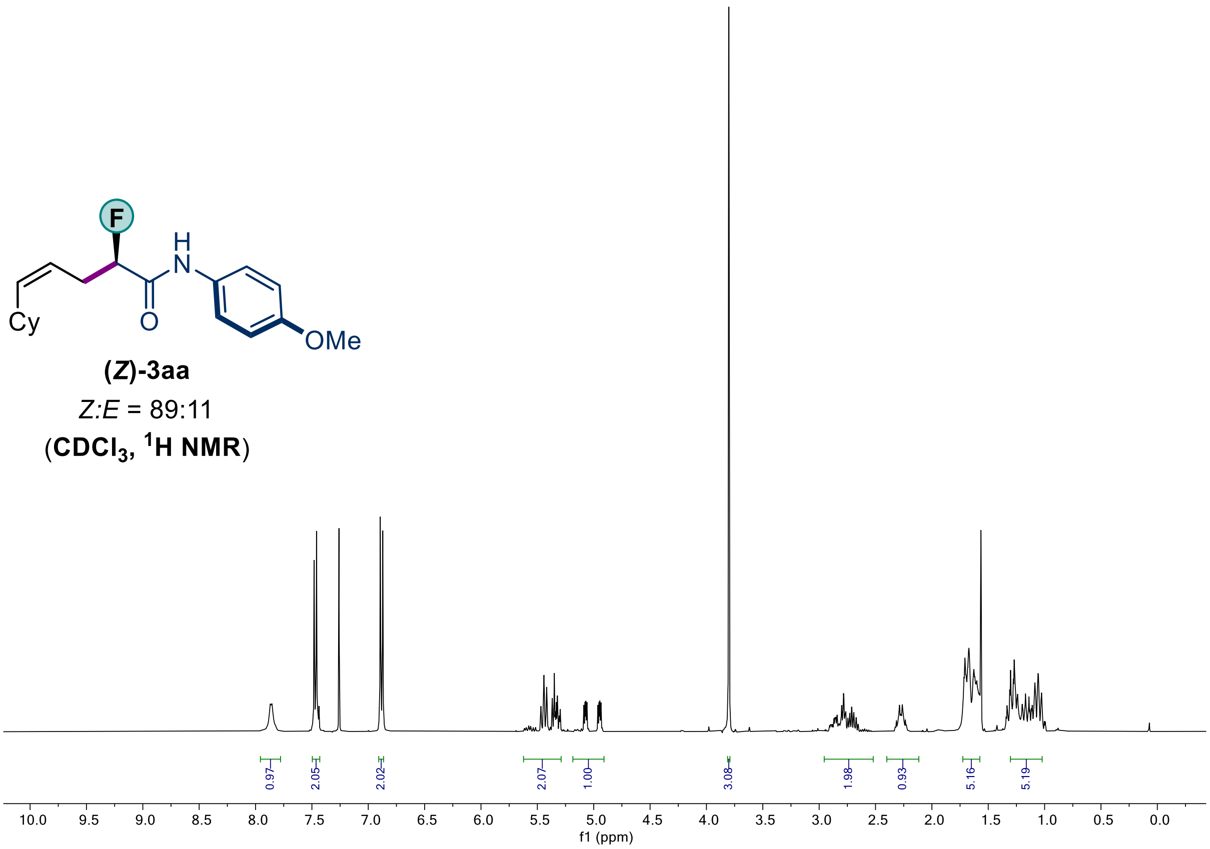
F
H
N
Cy
O
OMe
(*Z*)-3aa
Z:*E* = 89:11
($CDCl_3$, ^1H NMR)
0.97
2.05
2.02
2.07
1.00
3.08
1.98
0.93
5.16
5.19
10.0 9.5 9.0 8.5 8.0 7.5 7.0 6.5 6.0 5.5 5.0 4.5 4.0 3.5 3.0 2.5 2.0 1.5 1.0 0.5 0.0
f1 (ppm)

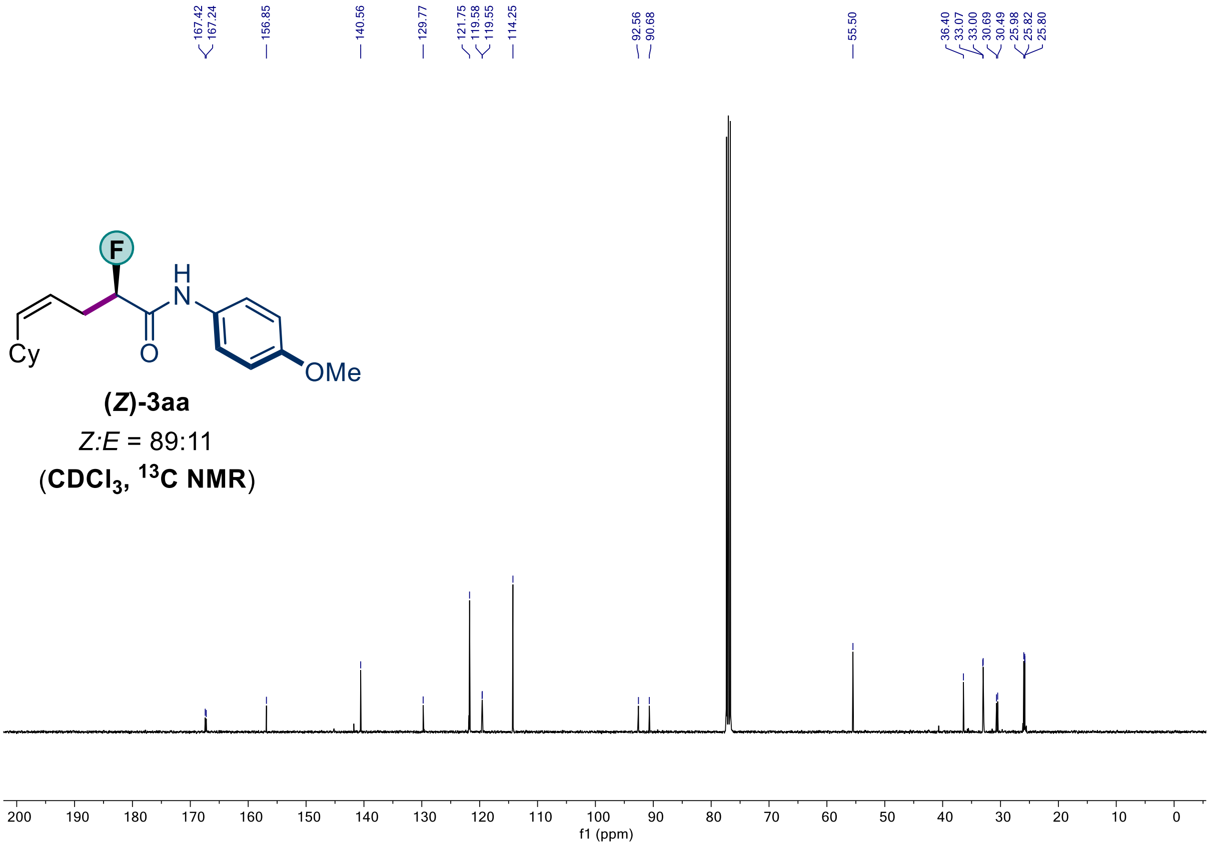
167.42
167.24
156.85
140.56
129.77
121.75
119.58
119.55
114.25
92.56
90.68
55.50
36.40
33.07
33.00
30.69
30.49
25.98
25.82
25.80
F
H
N
Cy
O
OMe
(*Z*)-3aa
Z:*E* = 89:11
($CDCl_3$, ^{13}C NMR)
200 190 180 170 160 150 140 130 120 110 100 90 80 70 60 50 40 30 20 10 0
f1 (ppm)

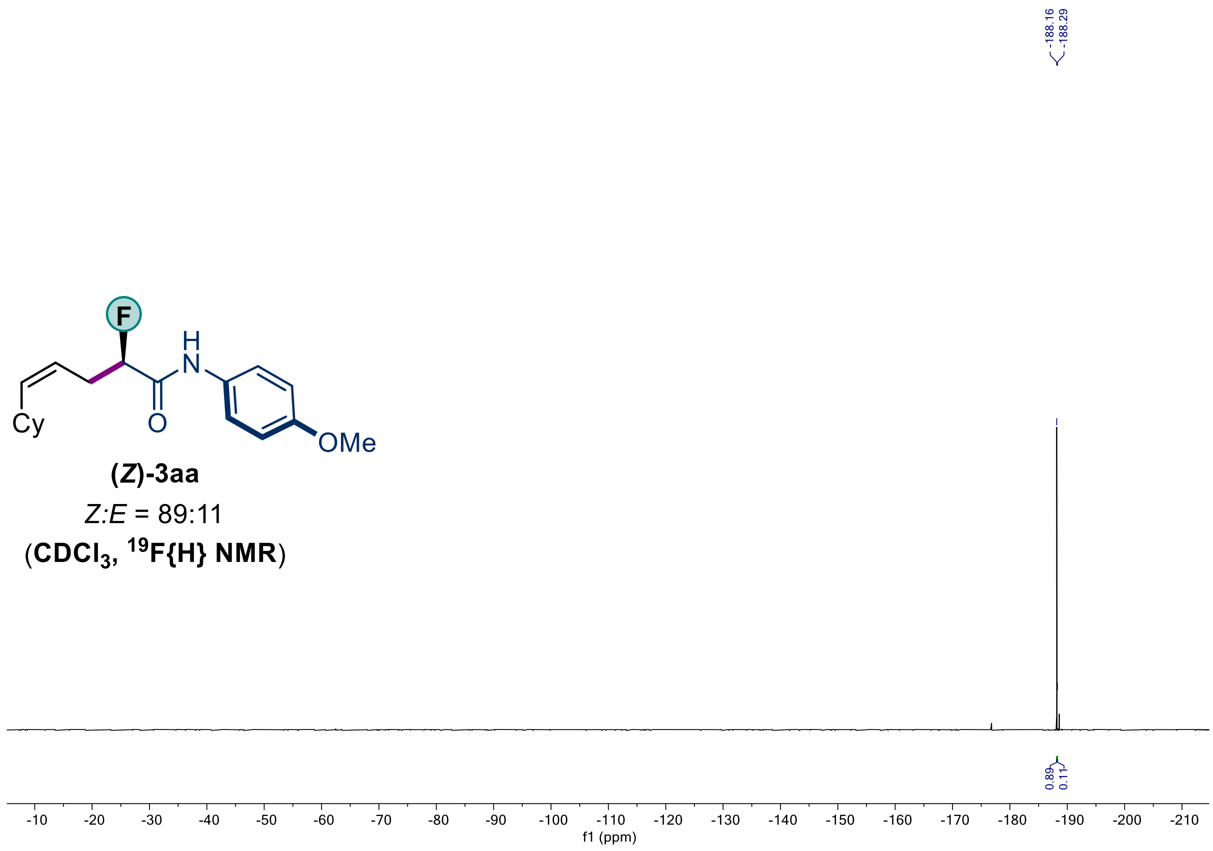

-188.16
-188.29
F
H
N
Cy
O
OMe
(Z)-3aa
Z:E = 89:11
(CDCl3, 19F{H} NMR)
0.89
0.11
-10 -20 -30 -40 -50 -60 -70 -80 -90 -100 -110 -120 -130 -140 -150 -160 -170 -180 -190 -200 -210
f1 (ppm)

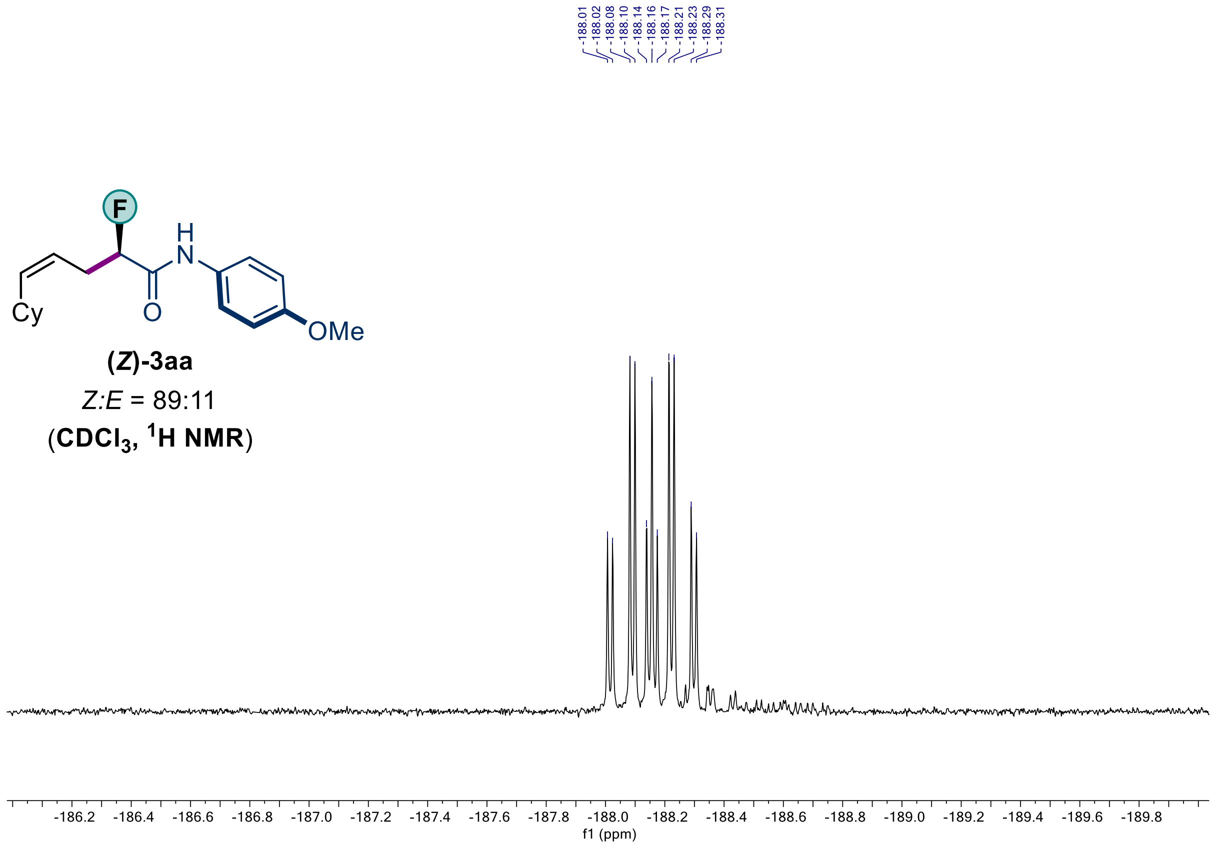

-188.01
-188.02
-188.08
-188.10
-188.14
-188.16
-188.17
-188.21
-188.23
-188.29
-188.31
F
H
N
Cy
O
OMe
(Z)-3aa
Z:E = 89:11
(CDCl3, 1H NMR)
-186.2 -186.4 -186.6 -186.8 -187.0 -187.2 -187.4 -187.6 -187.8 -188.0 -188.2 -188.4 -188.6 -188.8 -189.0 -189.2 -189.4 -189.6 -189.8
f1 (ppm)

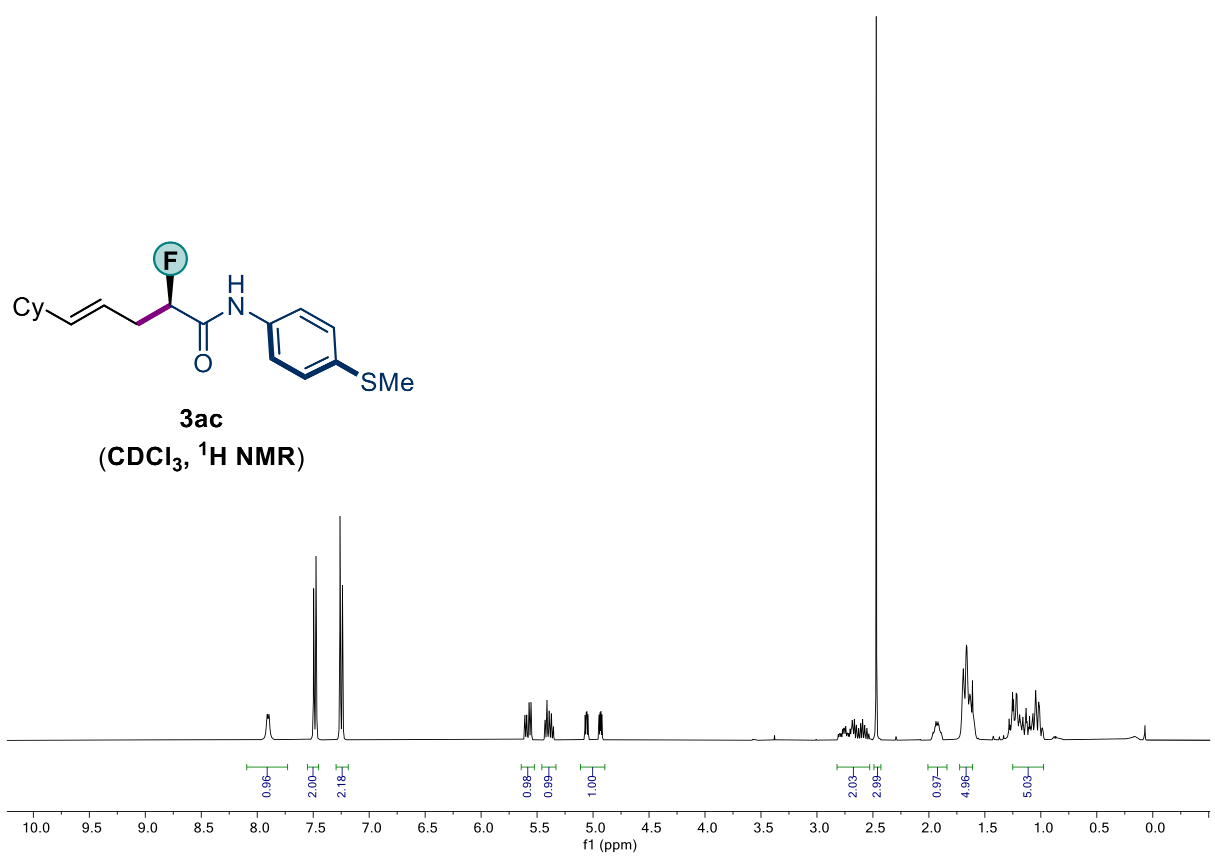
Cy
F
H
N
O
SMe
3ac
(CDCl3, 1H NMR)
0.96
2.00
2.18
0.98
0.99
1.00
2.03
2.99
0.97
4.96
5.03
10.0 9.5 9.0 8.5 8.0 7.5 7.0 6.5 6.0 5.5 5.0 4.5 4.0 3.5 3.0 2.5 2.0 1.5 1.0 0.5 0.0
f1 (ppm)

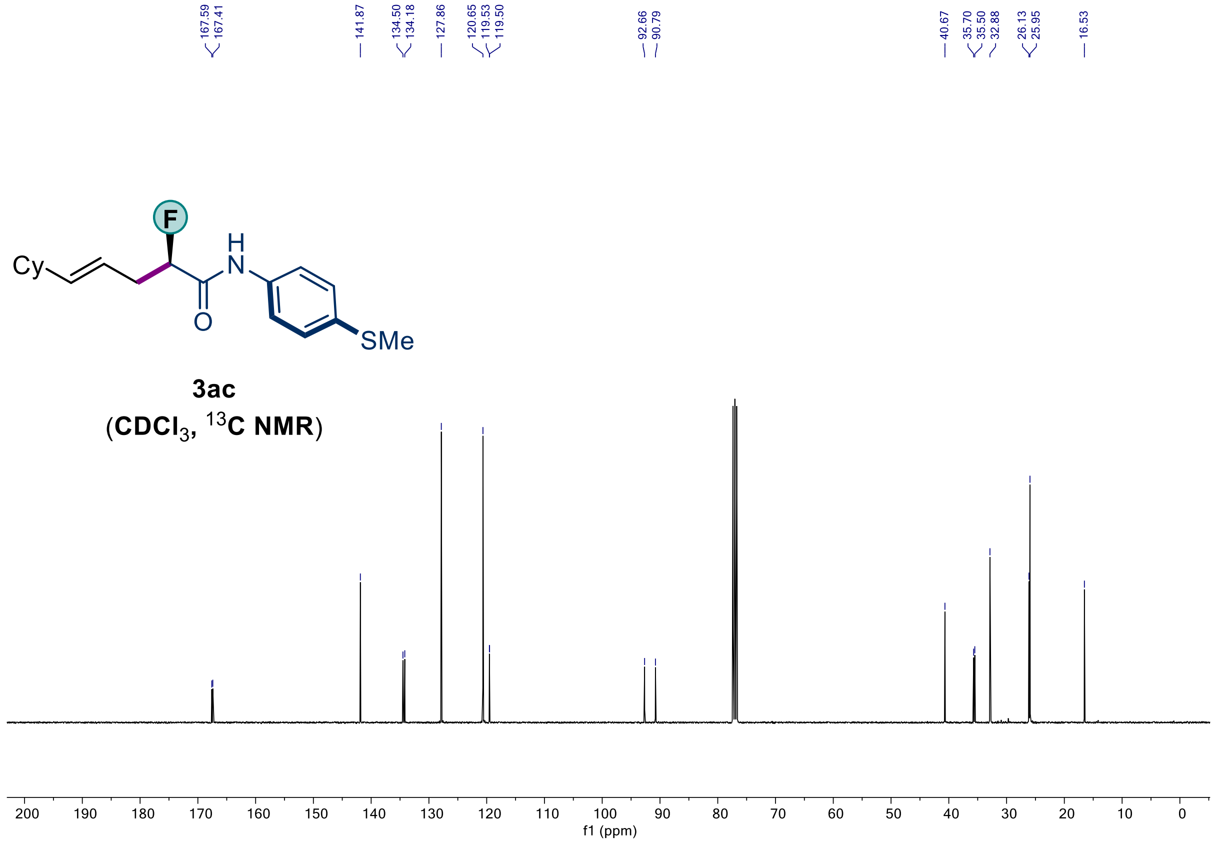
167.59
167.41
141.87
134.50
134.18
127.86
120.65
119.53
119.50
92.66
90.79
40.67
35.70
35.50
32.88
26.13
25.95
16.53
Cy
F
H
N
O
SMe
3ac
(CDCl3, 13C NMR)
200 190 180 170 160 150 140 130 120 110 100 90 80 70 60 50 40 30 20 10 0
f1 (ppm)

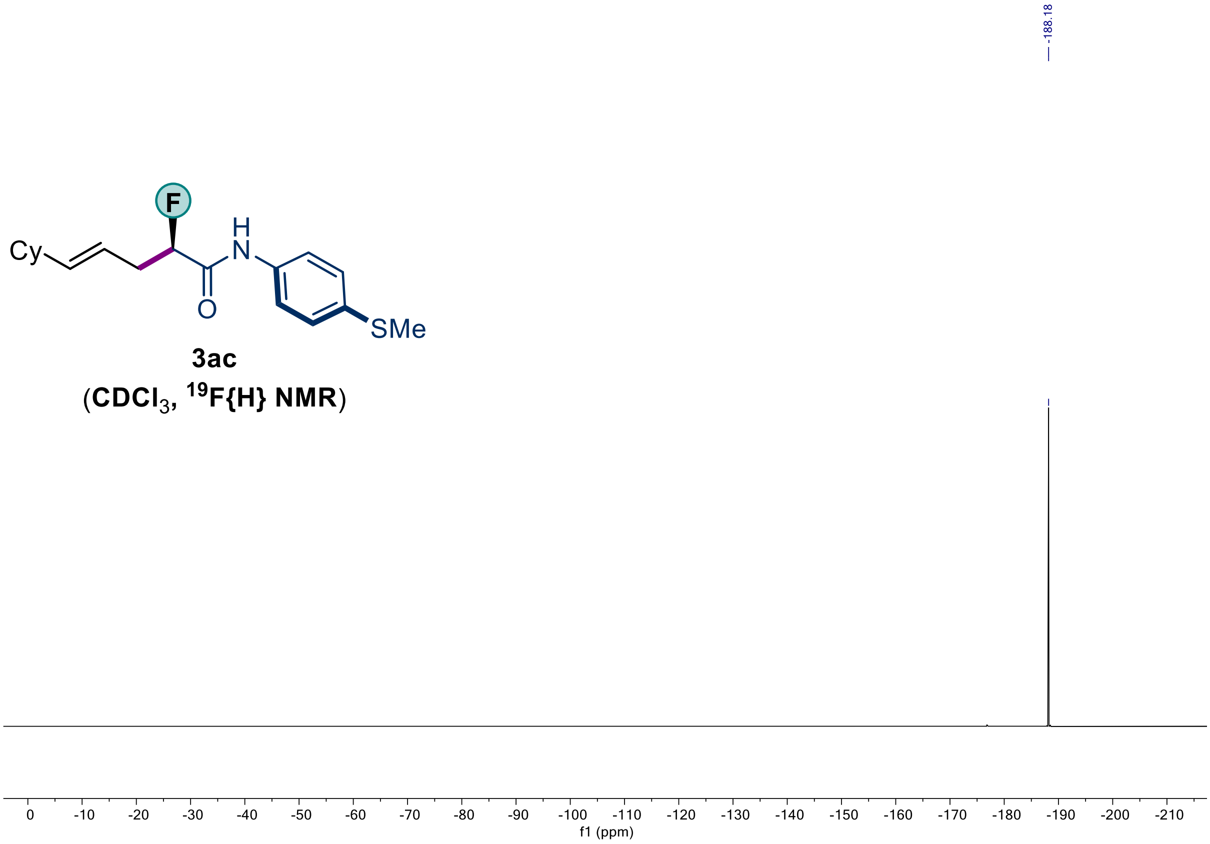

3ac
(**CDCl$_3$, ^{19}F{H} NMR**)

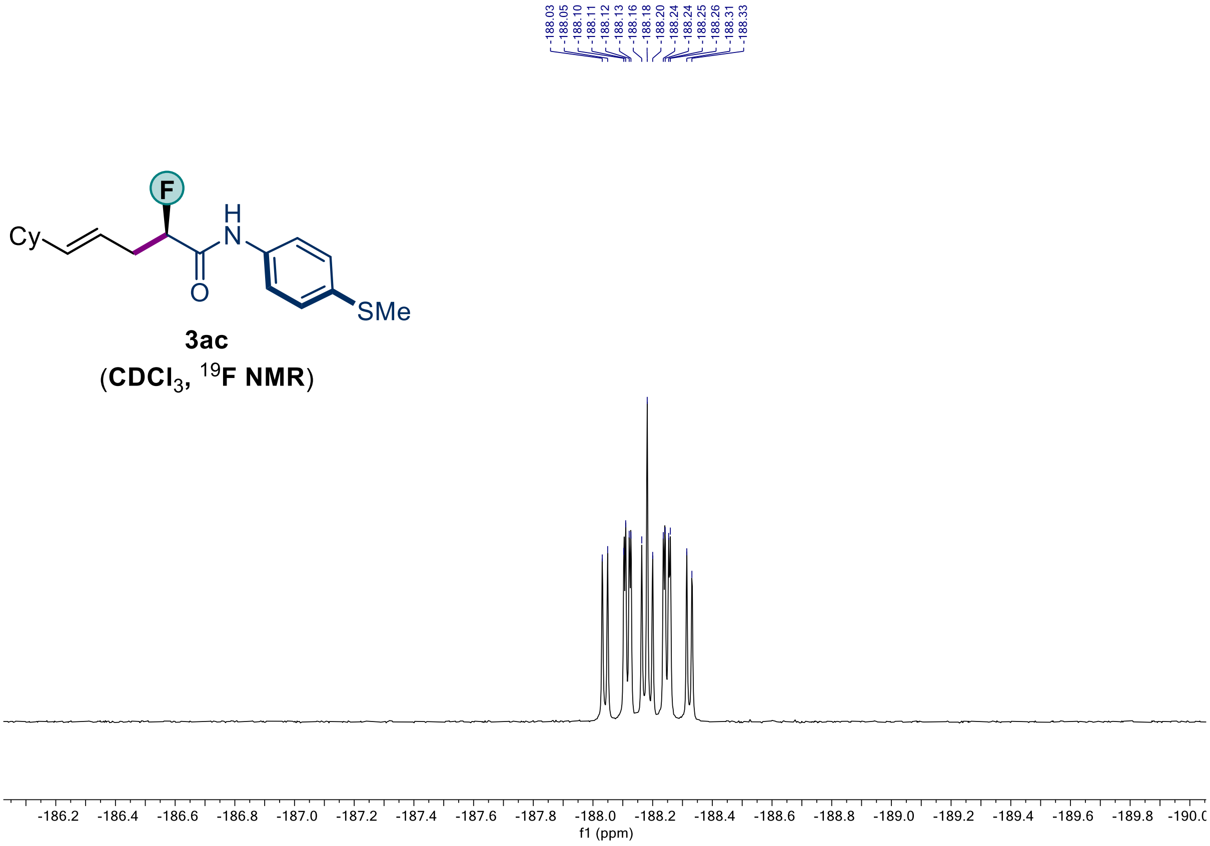

3ac
(**CDCl$_3$, ^{19}F NMR**)

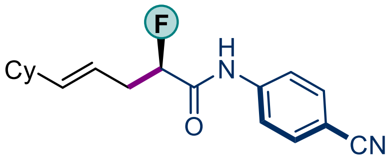

3ad
(**CDCl$_3$, ^{1}H NMR**)

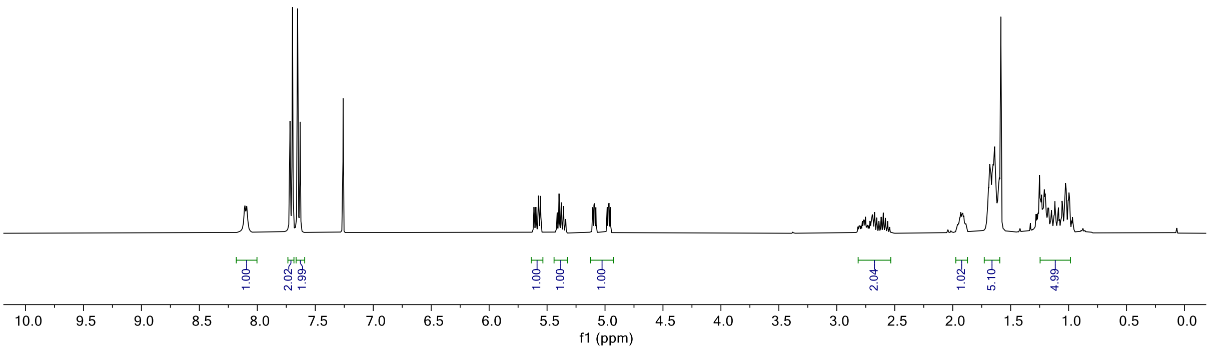

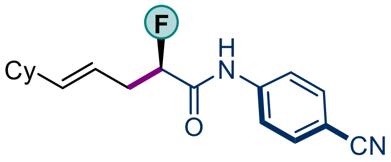

3ad
(**CDCl$_3$, ^{13}C NMR**)

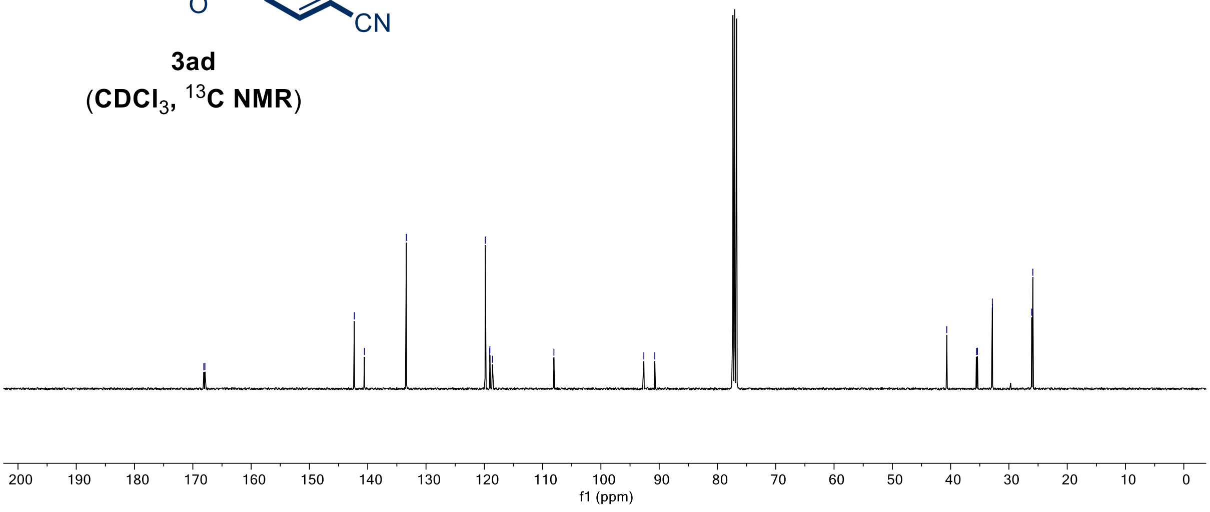

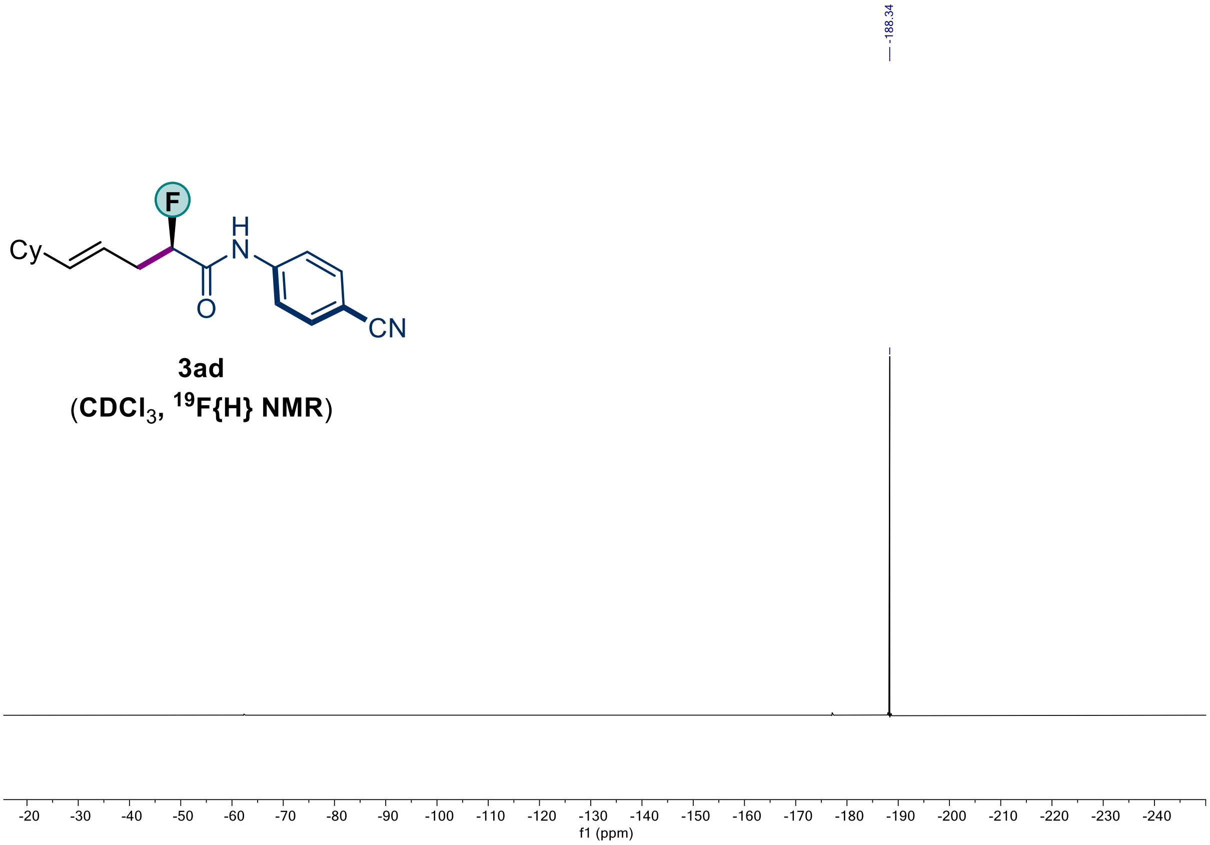

3ad
(**CDCl$_3$, ^{19}F{H} NMR**)

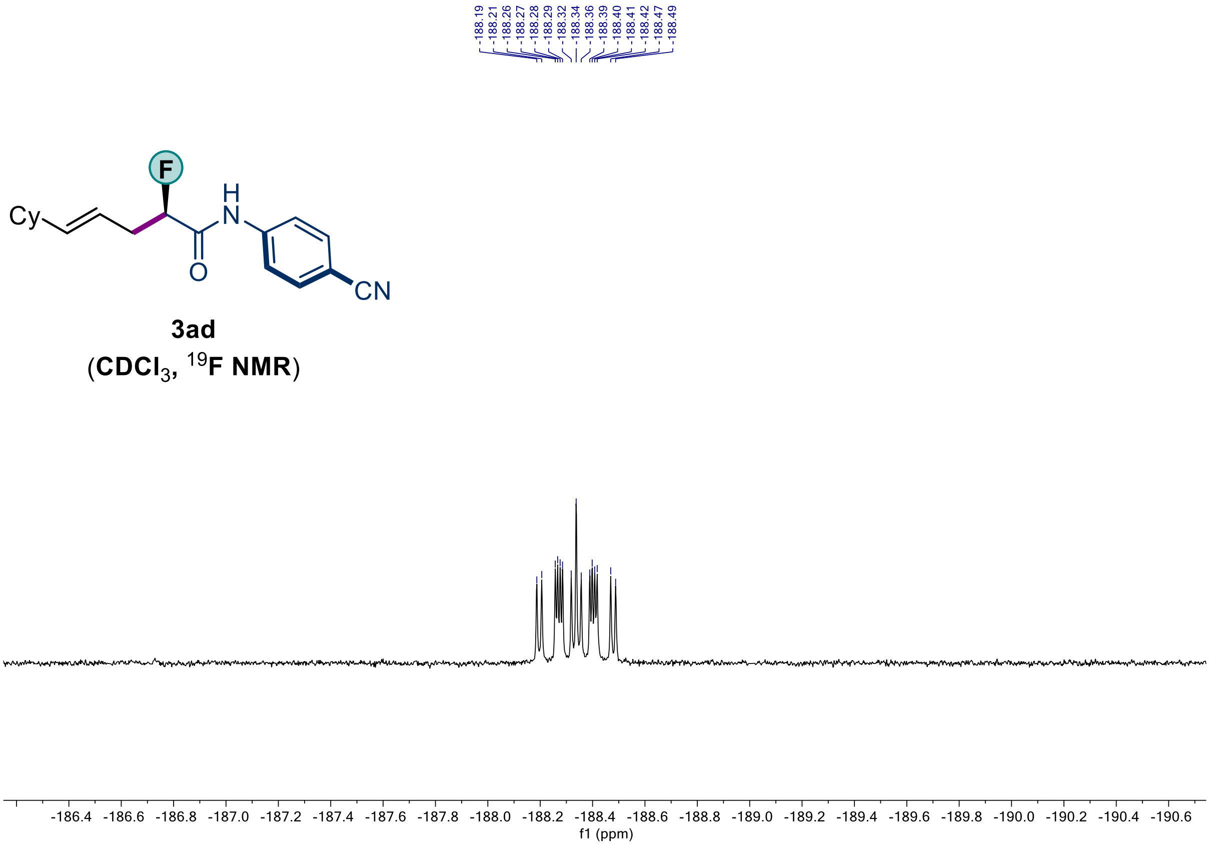

3ad
(**CDCl$_3$, ^{19}F NMR**)

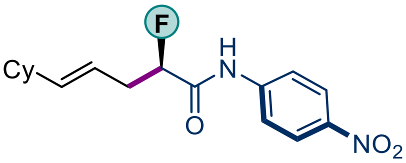

3ae
(**$CDCl_3$, ^{1}H NMR**)

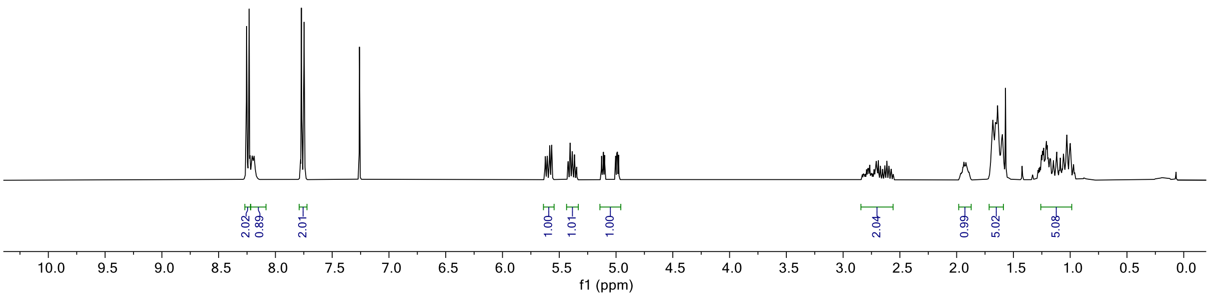

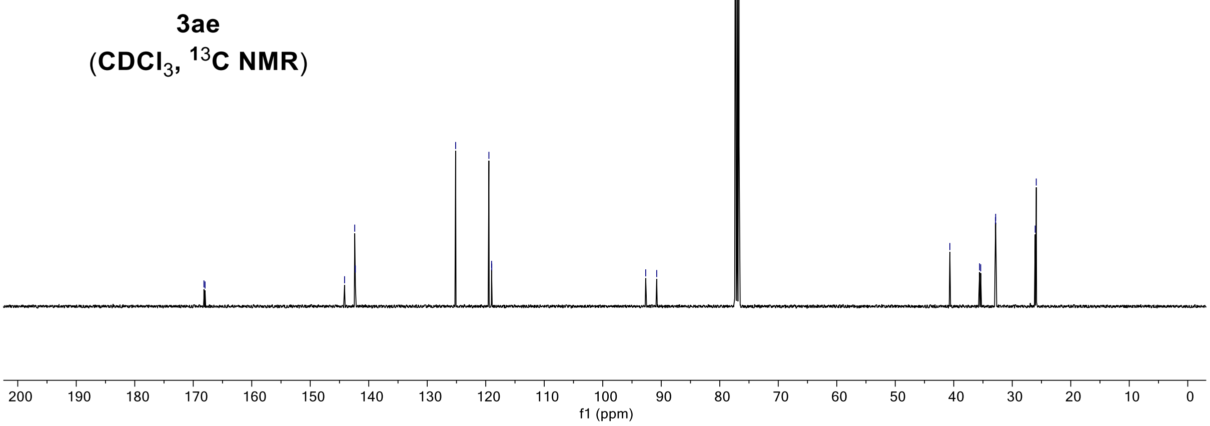

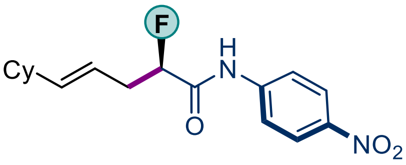

3ae
(**$CDCl_3$, ^{13}C NMR**)

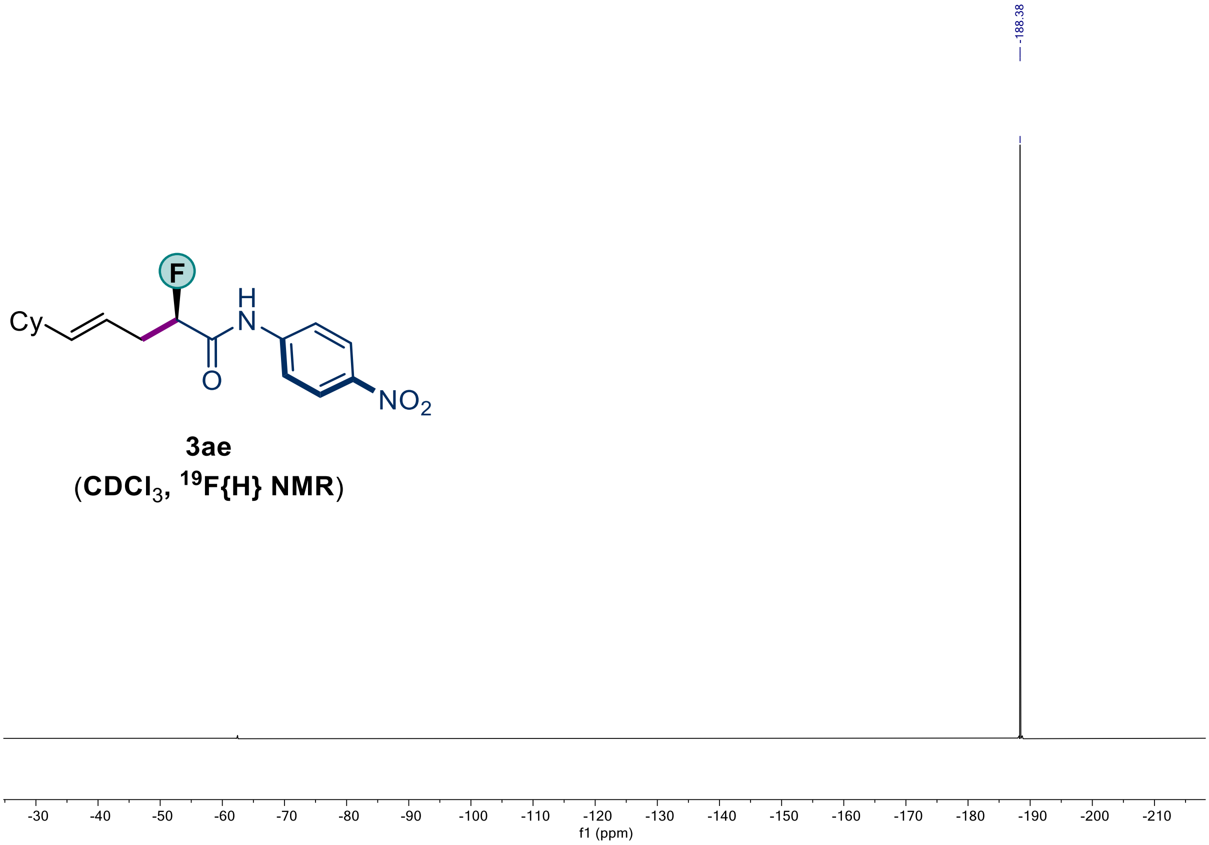
-188.38
Cy
F
H
N
O
NO2
3ae
(CDCl3, 19F{H} NMR)
-30 -40 -50 -60 -70 -80 -90 -100 -110 -120 -130 -140 -150 -160 -170 -180 -190 -200 -210
f1 (ppm)

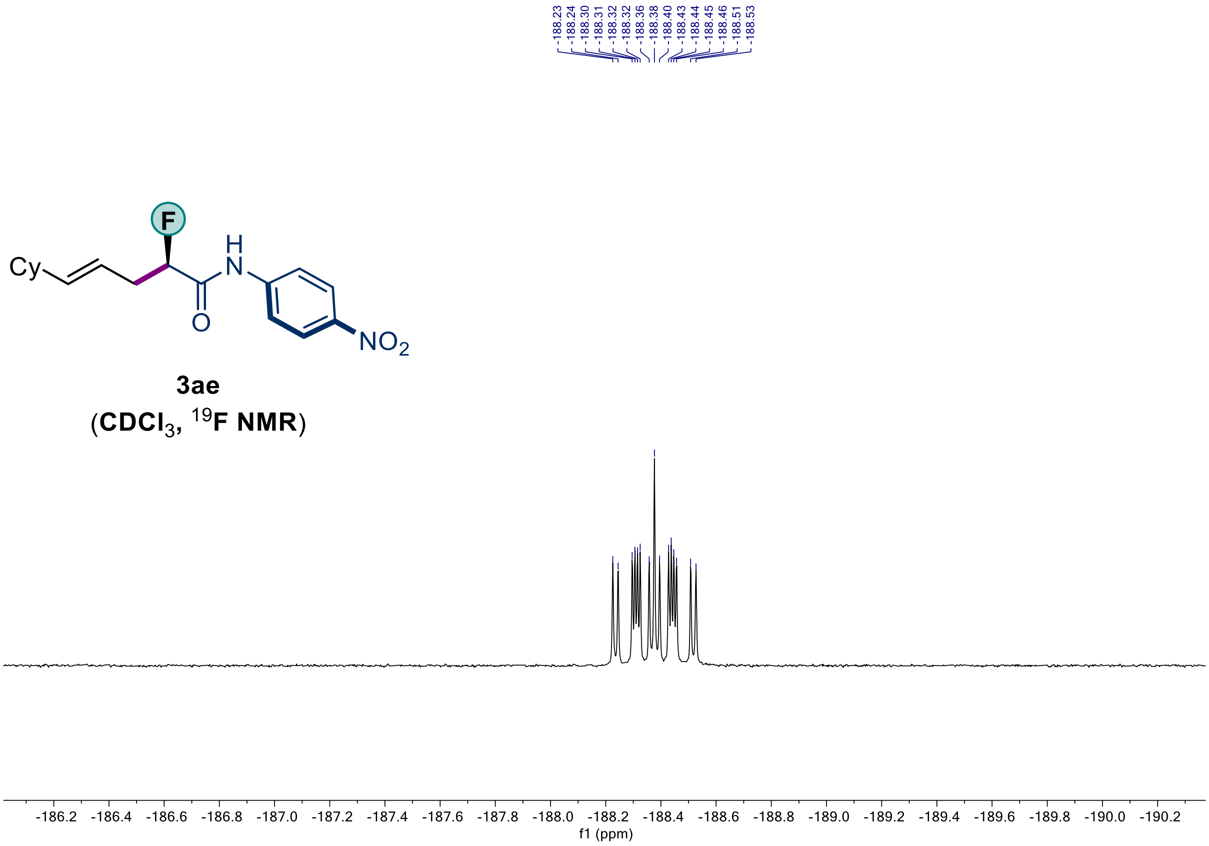
-188.23
-188.24
-188.30
-188.31
-188.32
-188.32
-188.36
-188.38
-188.40
-188.43
-188.44
-188.45
-188.46
-188.51
-188.53
Cy
F
H
N
O
NO2
3ae
(CDCl3, 19F NMR)
-186.2 -186.4 -186.6 -186.8 -187.0 -187.2 -187.4 -187.6 -187.8 -188.0 -188.2 -188.4 -188.6 -188.8 -189.0 -189.2 -189.4 -189.6 -189.8 -190.0 -190.2
f1 (ppm)

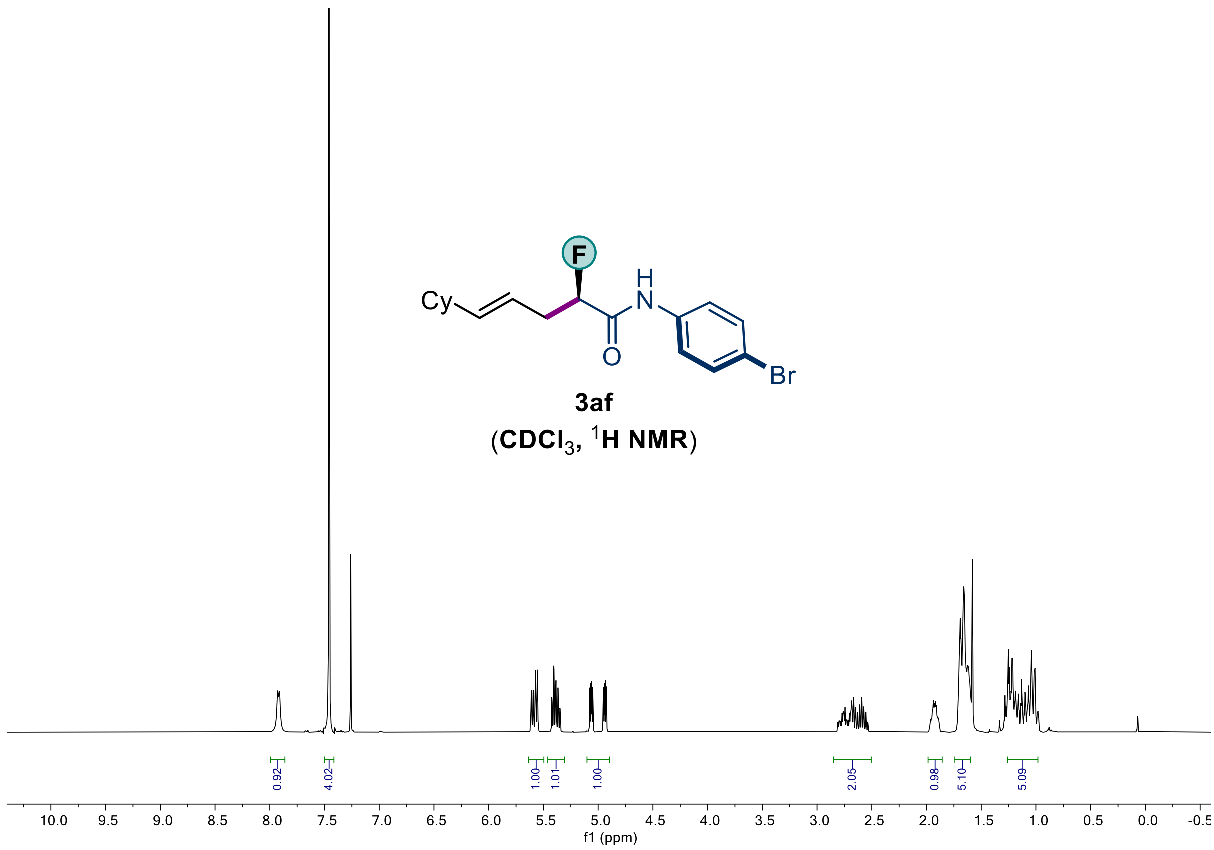
Cy
F
H
N
O
Br
3af
(CDCl3, 1H NMR)
0.92
4.02
1.00
1.01
1.00
2.05
0.98
5.10
5.09
f1 (ppm)

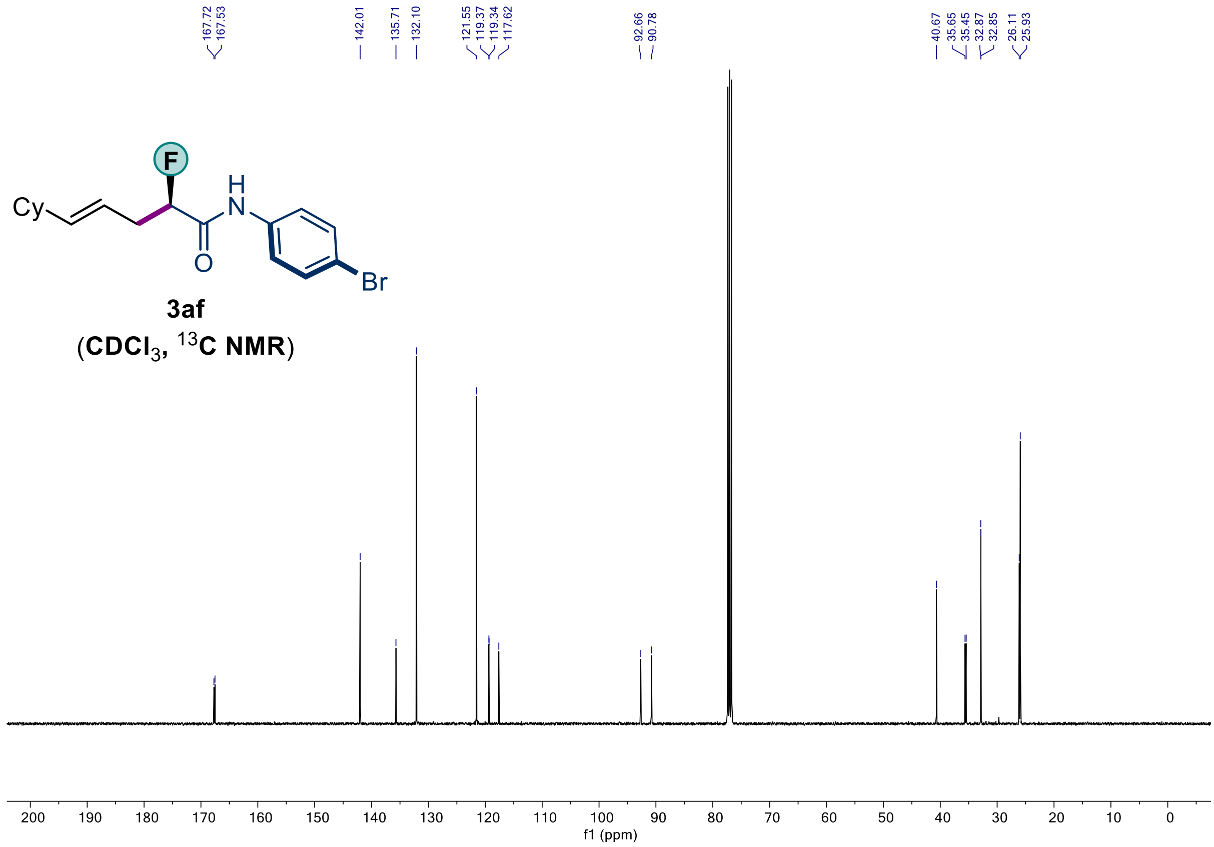
167.72
167.53
142.01
135.71
132.10
121.55
119.37
119.34
117.62
92.66
90.78
40.67
35.65
35.45
32.87
32.85
26.11
25.93
Cy
F
H
N
O
Br
3af
(CDCl3, 13C NMR)
f1 (ppm)

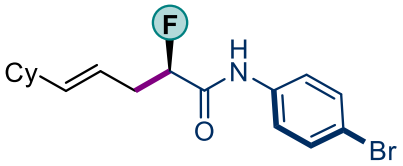

3af

(**CDCl$_3$**, ^{19}**F{H} NMR**)

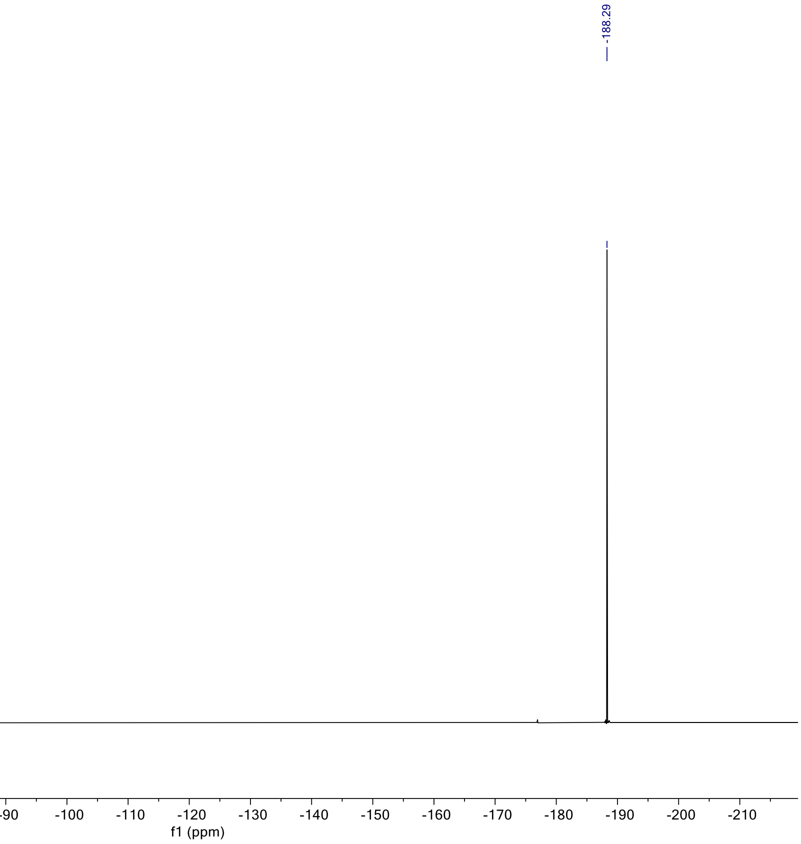

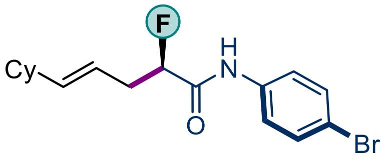

3af

(**CDCl$_3$**, ^{19}**F NMR**)

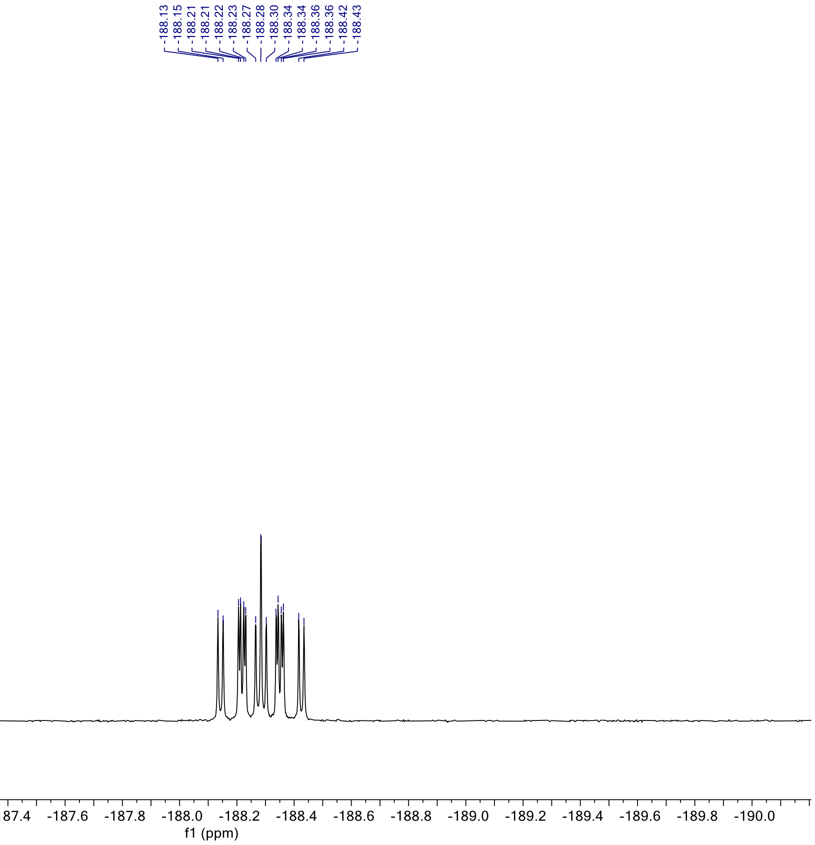

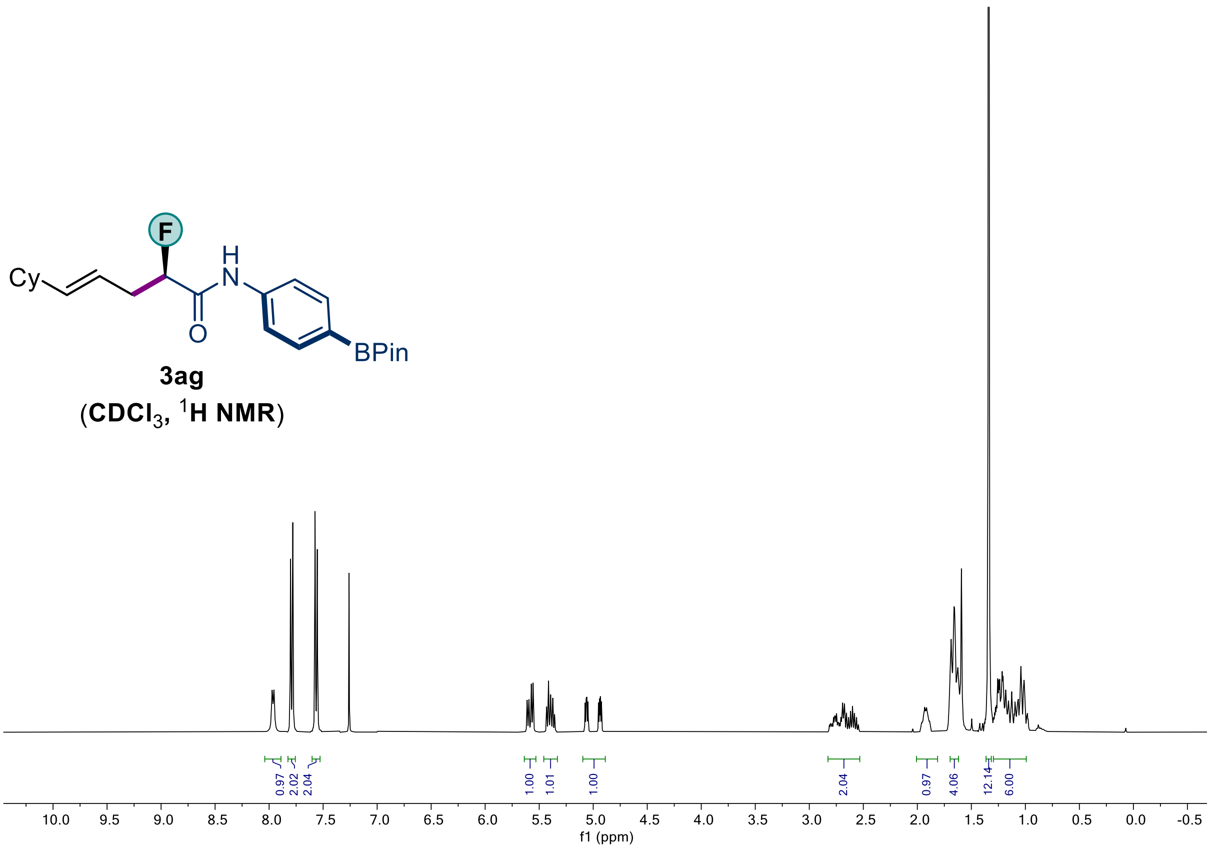
Cy
F
H
N
O
BPin
3ag
(CDCl3, 1H NMR)
0.97
2.02
2.04
1.00
1.01
1.00
2.04
0.97
4.06
12.14
6.00
10.0 9.5 9.0 8.5 8.0 7.5 7.0 6.5 6.0 5.5 5.0 4.5 4.0 3.5 3.0 2.5 2.0 1.5 1.0 0.5 0.0 -0.5
f1 (ppm)

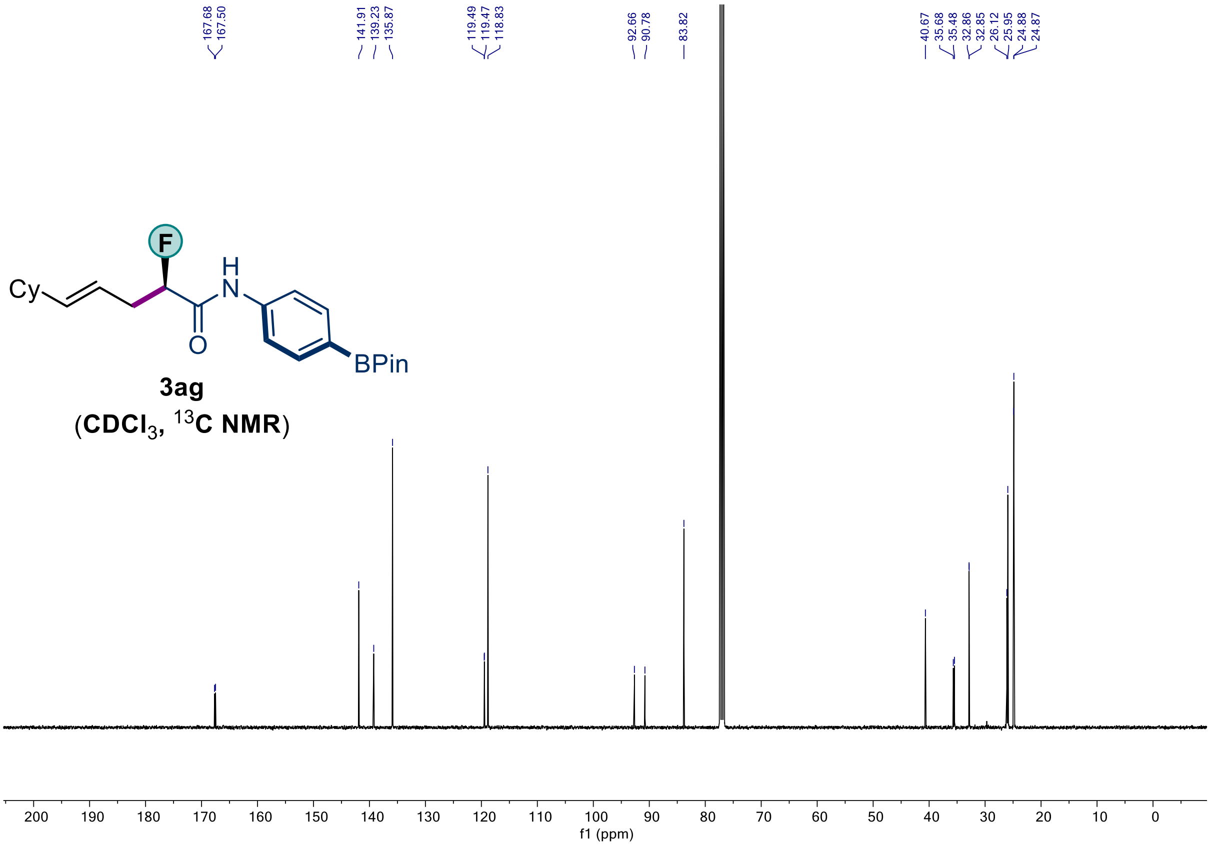
167.68
167.50
141.91
139.23
135.87
119.49
119.47
118.83
92.66
90.78
83.82
40.67
35.68
35.48
32.86
32.85
26.12
25.95
24.88
24.87
Cy
F
H
N
O
BPin
3ag
(CDCl3, 13C NMR)
200 190 180 170 160 150 140 130 120 110 100 90 80 70 60 50 40 30 20 10 0
f1 (ppm)

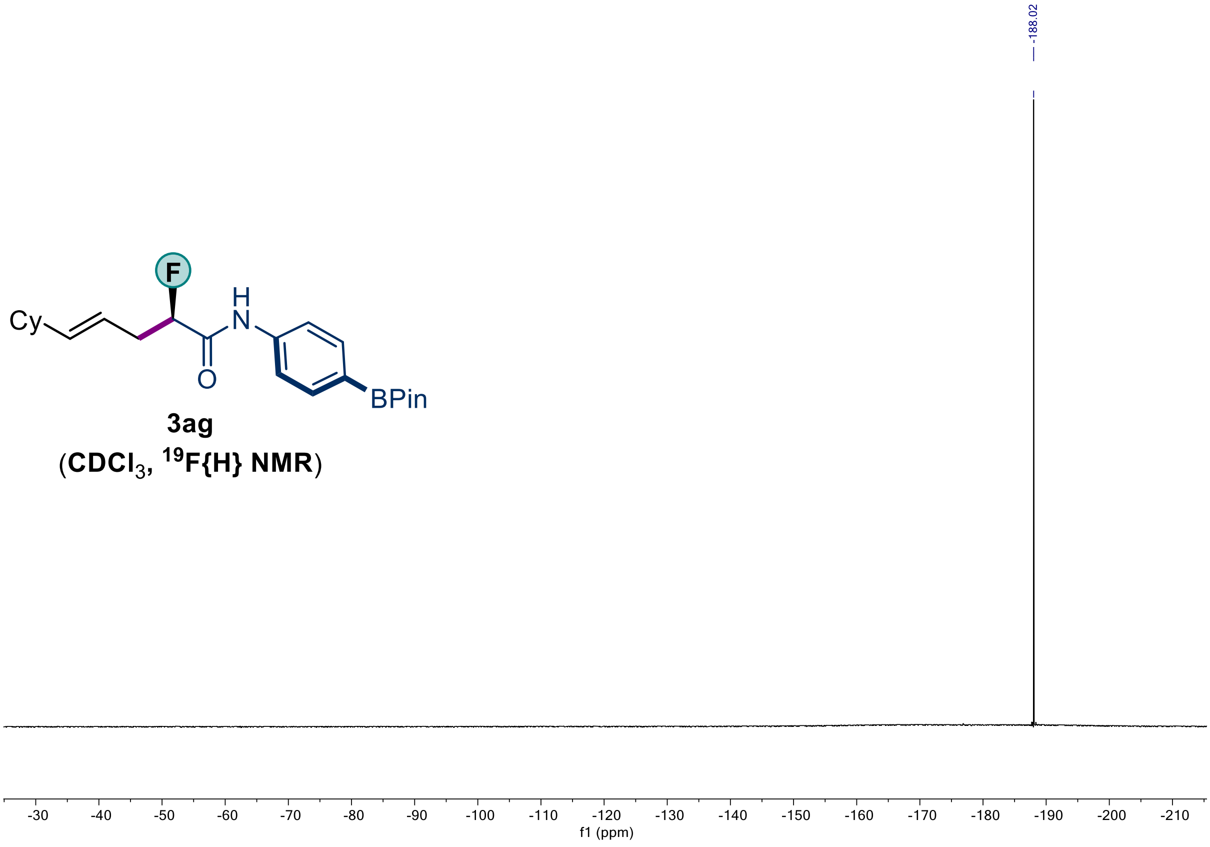
-188.02
Cy
F
H
N
O
BPin
3ag
(CDCl3, 19F{H} NMR)
-30 -40 -50 -60 -70 -80 -90 -100 -110 -120 -130 -140 -150 -160 -170 -180 -190 -200 -210
f1 (ppm)

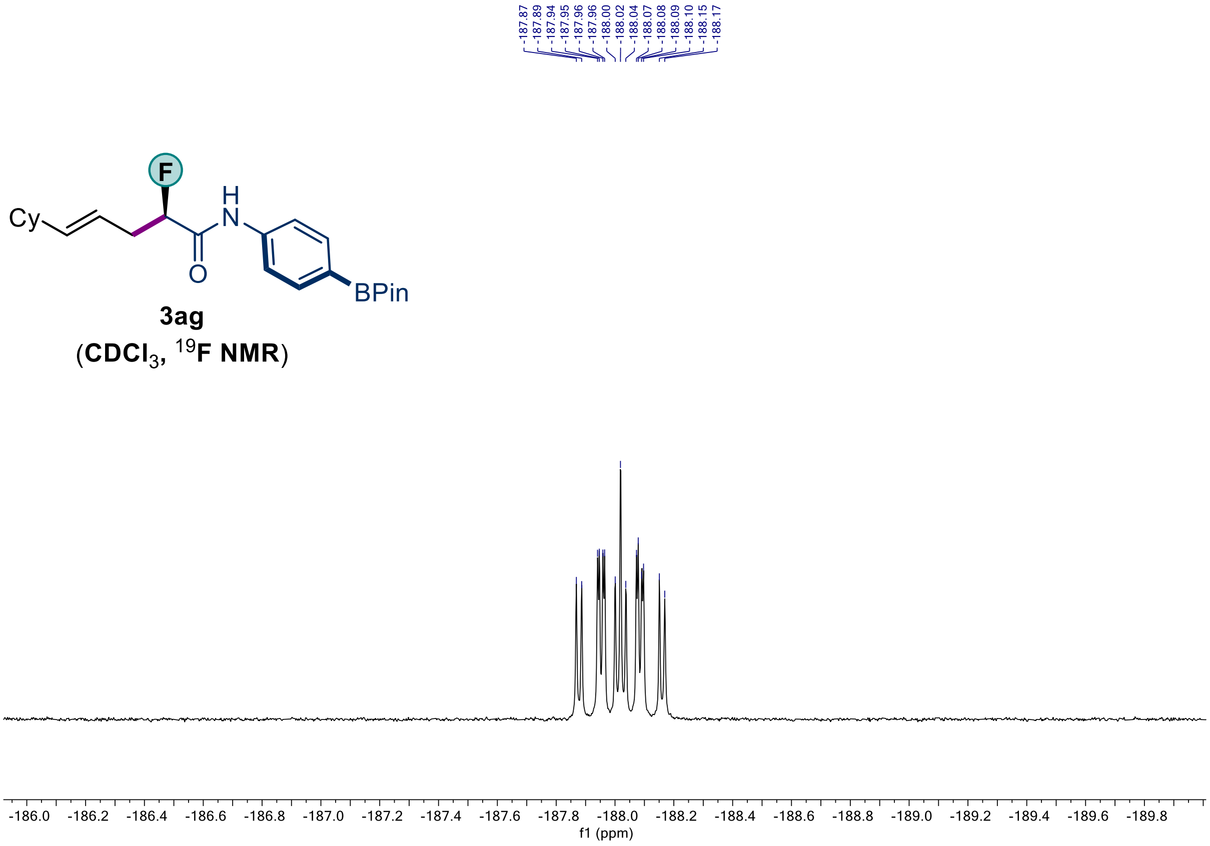
-187.87
-187.89
-187.94
-187.95
-187.96
-187.96
-188.00
-188.02
-188.04
-188.07
-188.08
-188.09
-188.10
-188.15
-188.17
Cy
F
H
N
O
BPin
3ag
(CDCl3, 19F NMR)
-186.0 -186.2 -186.4 -186.6 -186.8 -187.0 -187.2 -187.4 -187.6 -187.8 -188.0 -188.2 -188.4 -188.6 -188.8 -189.0 -189.2 -189.4 -189.6 -189.8
f1 (ppm)

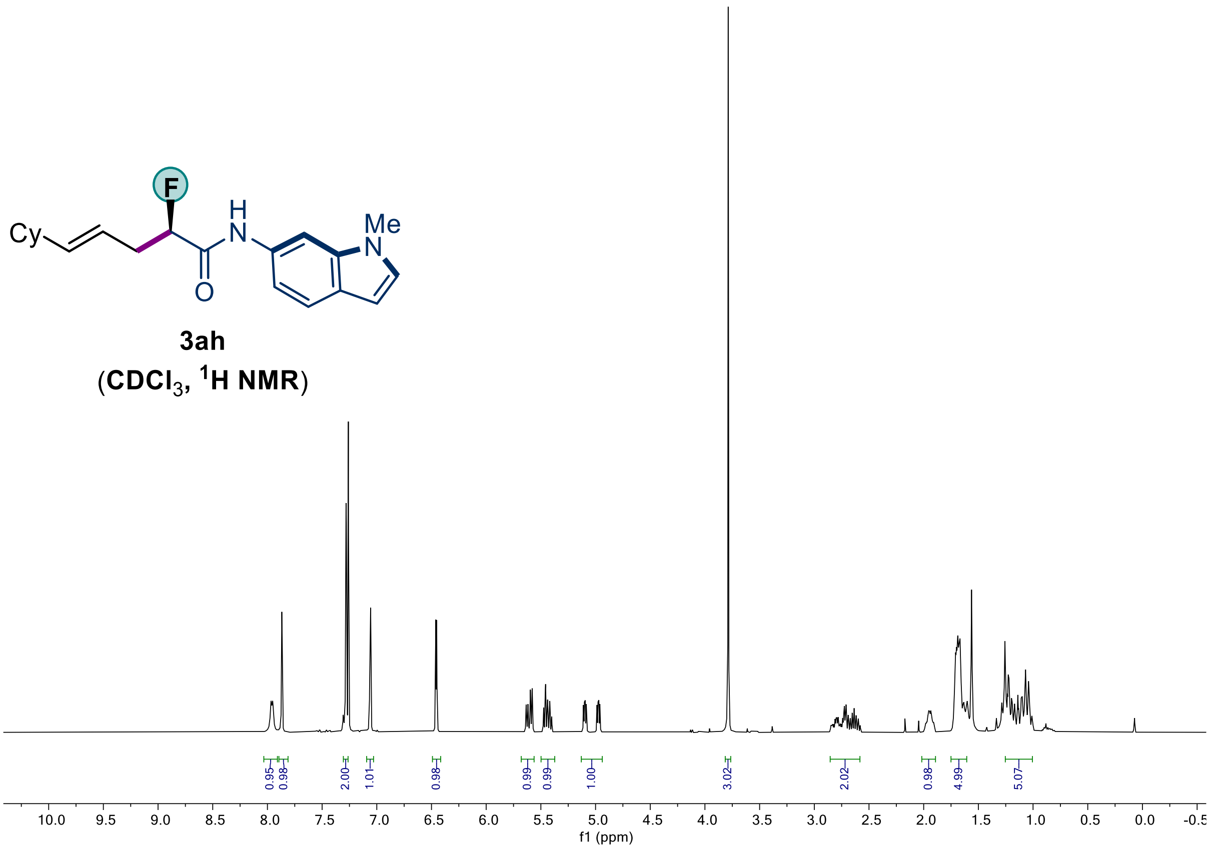
Cy
F
H
N
Me
N
O
3ah
(CDCl3, 1H NMR)
0.95
0.98
2.00
1.01
0.98
0.99
0.99
1.00
3.02
2.02
0.98
4.99
5.07
f1 (ppm)

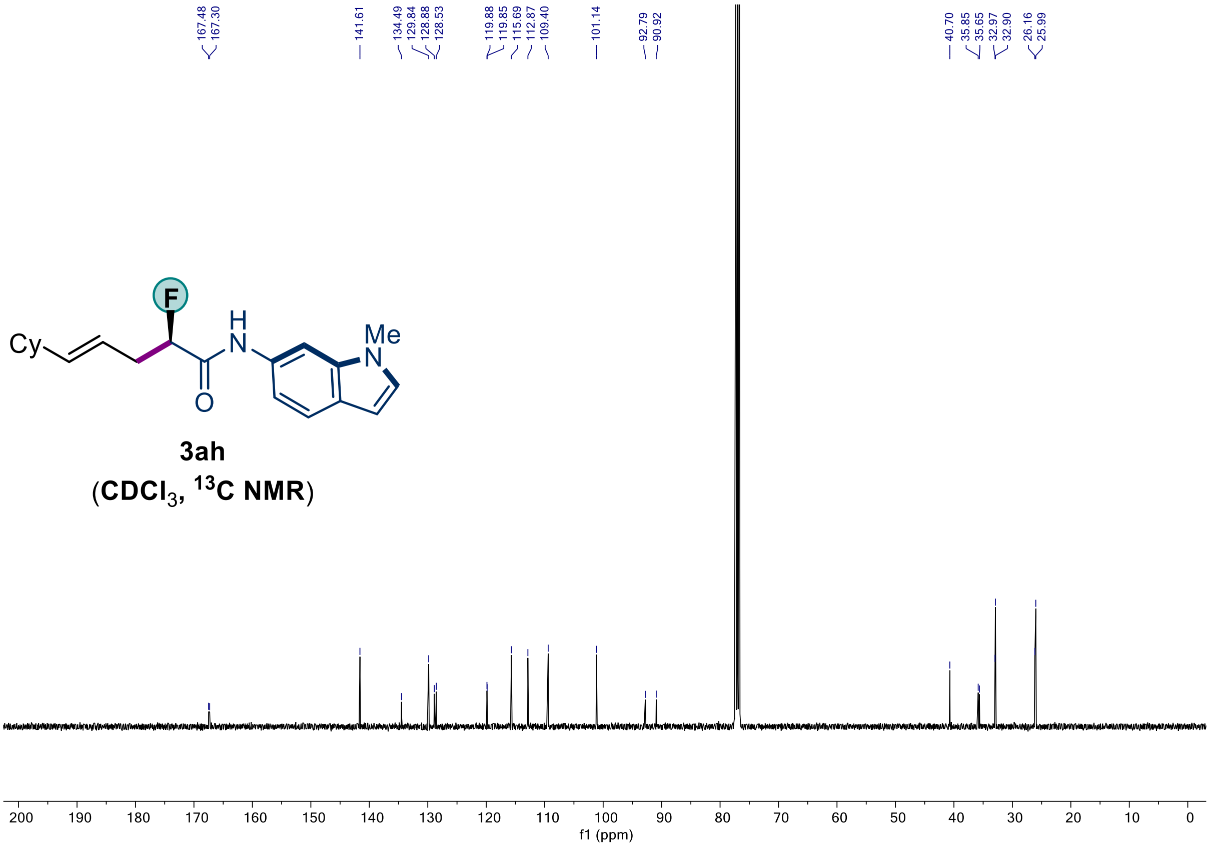
167.48
167.30
141.61
134.49
129.84
128.88
128.53
119.88
119.85
115.69
112.87
109.40
101.14
92.79
90.92
40.70
35.85
35.65
32.97
32.90
26.16
25.99
Cy
F
H
N
Me
N
O
3ah
(CDCl3, 13C NMR)
f1 (ppm)

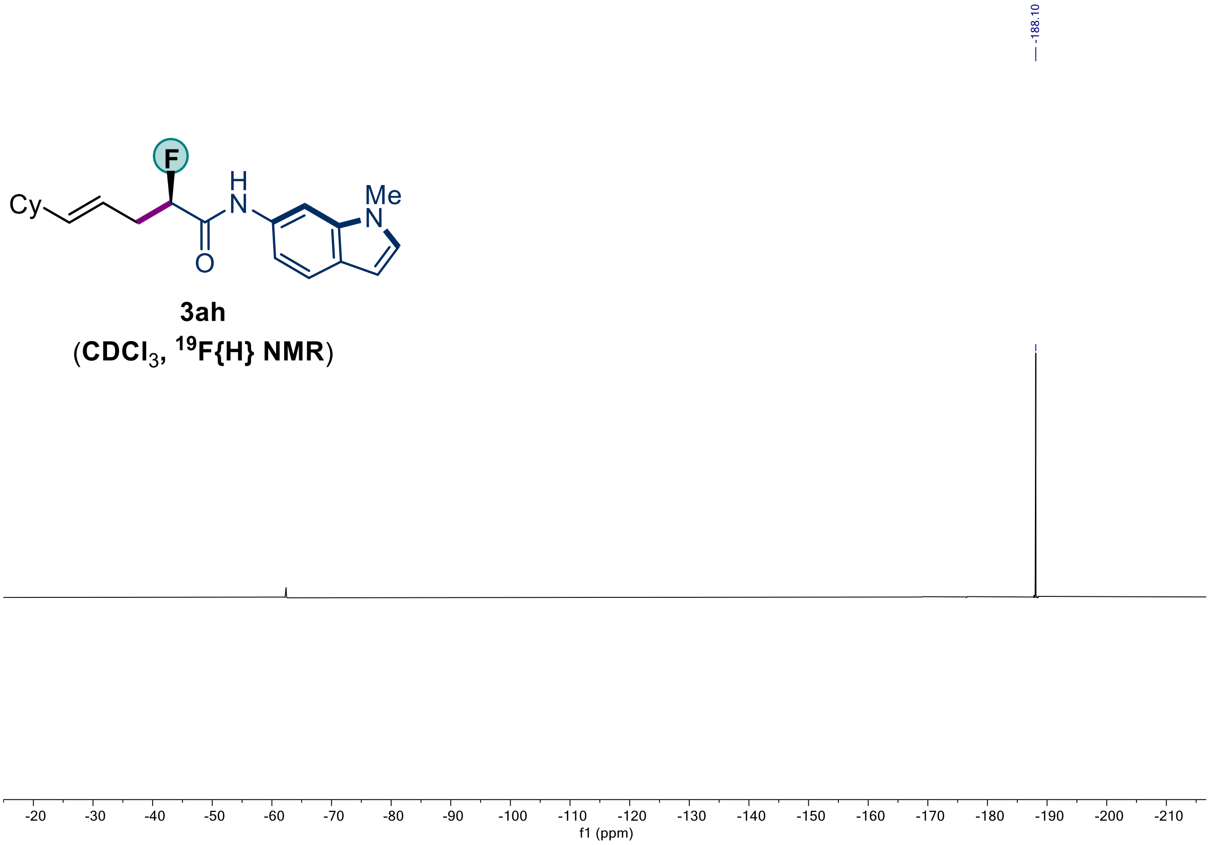
-188.10
Cy
F
H
N
Me
N
O
3ah
(CDCl3, 19F{H} NMR)
-20 -30 -40 -50 -60 -70 -80 -90 -100 -110 -120 -130 -140 -150 -160 -170 -180 -190 -200 -210
f1 (ppm)

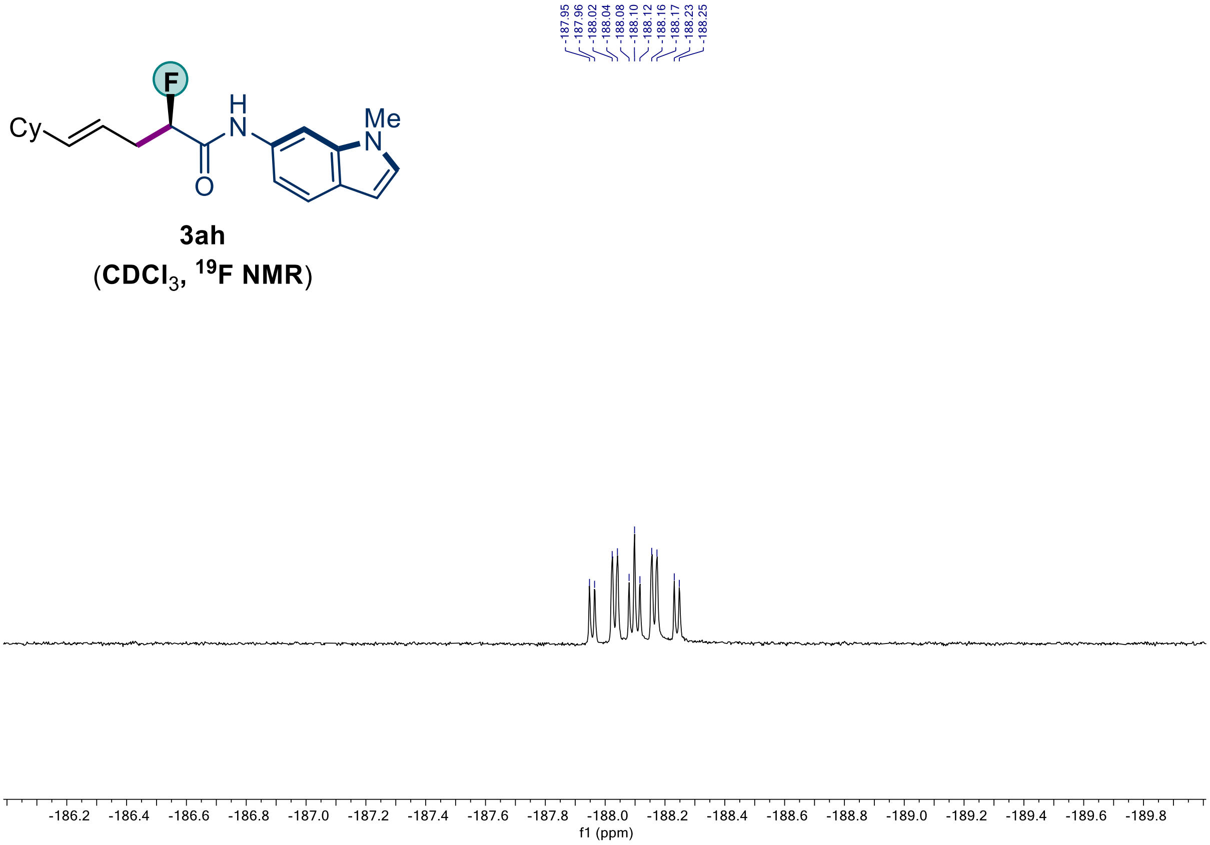
-187.95
-187.96
-188.02
-188.04
-188.08
-188.10
-188.12
-188.16
-188.17
-188.23
-188.25
Cy
F
H
N
Me
N
O
3ah
(CDCl3, 19F NMR)
-186.2 -186.4 -186.6 -186.8 -187.0 -187.2 -187.4 -187.6 -187.8 -188.0 -188.2 -188.4 -188.6 -188.8 -189.0 -189.2 -189.4 -189.6 -189.8
f1 (ppm)

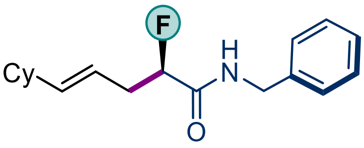

3ai
($CDCl_3$, ^1H NMR)

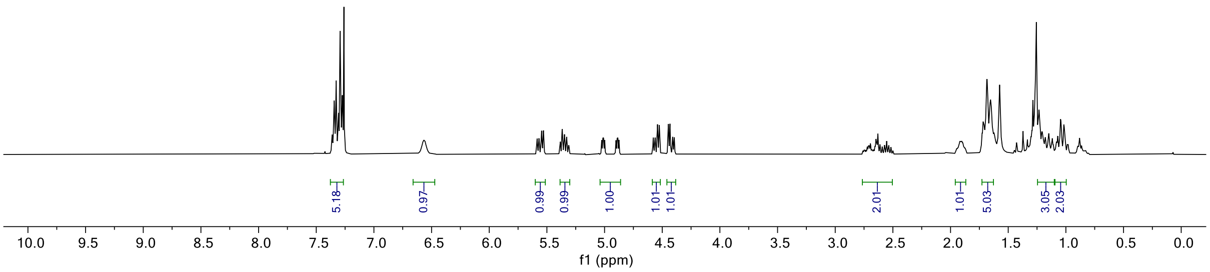

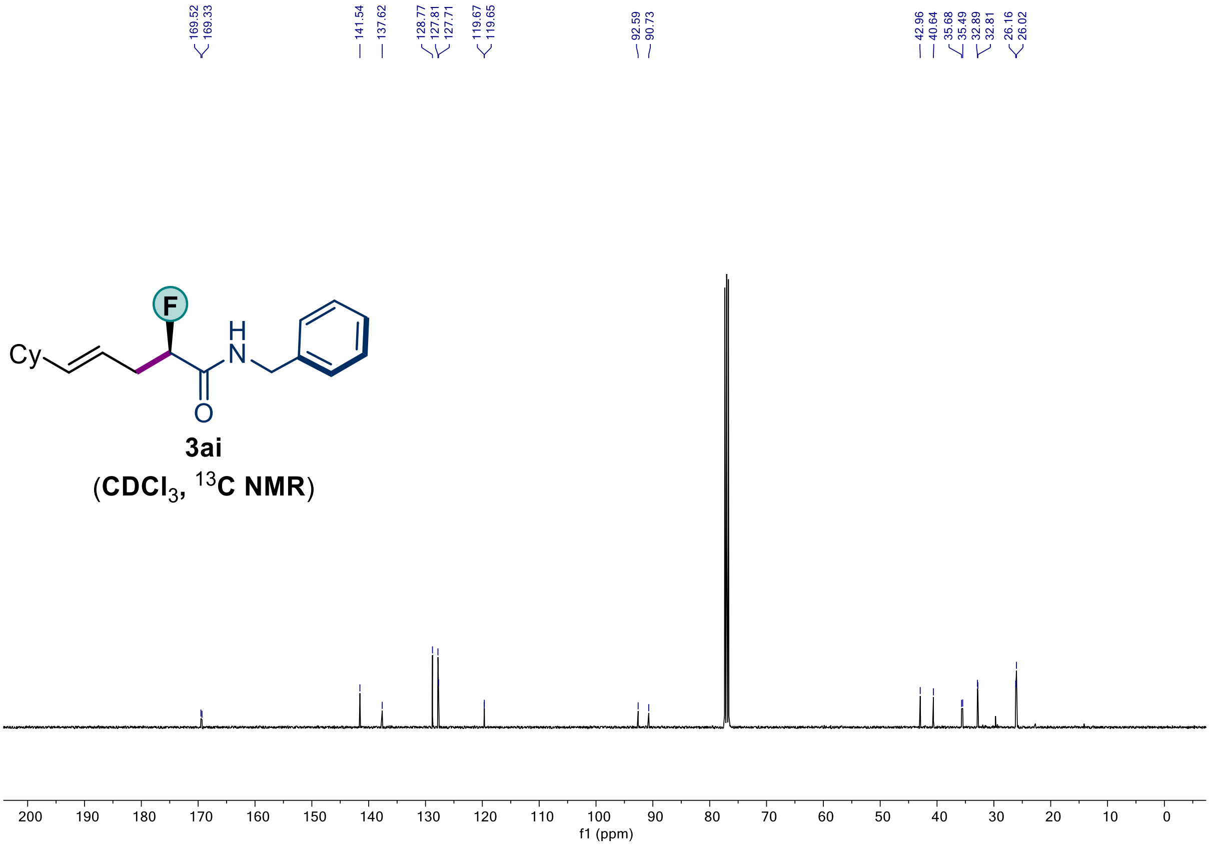

3ai
($CDCl_3$, ^{13}C NMR)

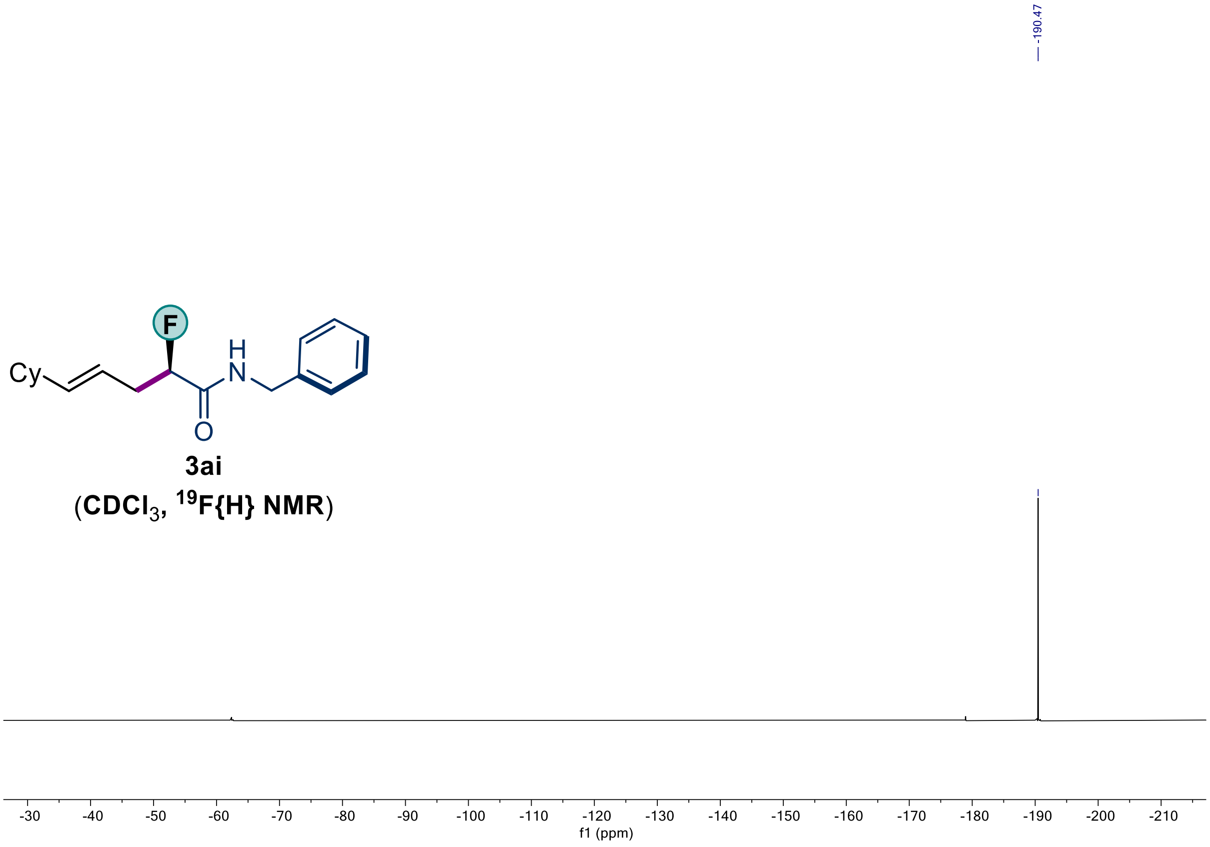

3ai
($CDCl_3$, $^{19}F\{H\}$ NMR)

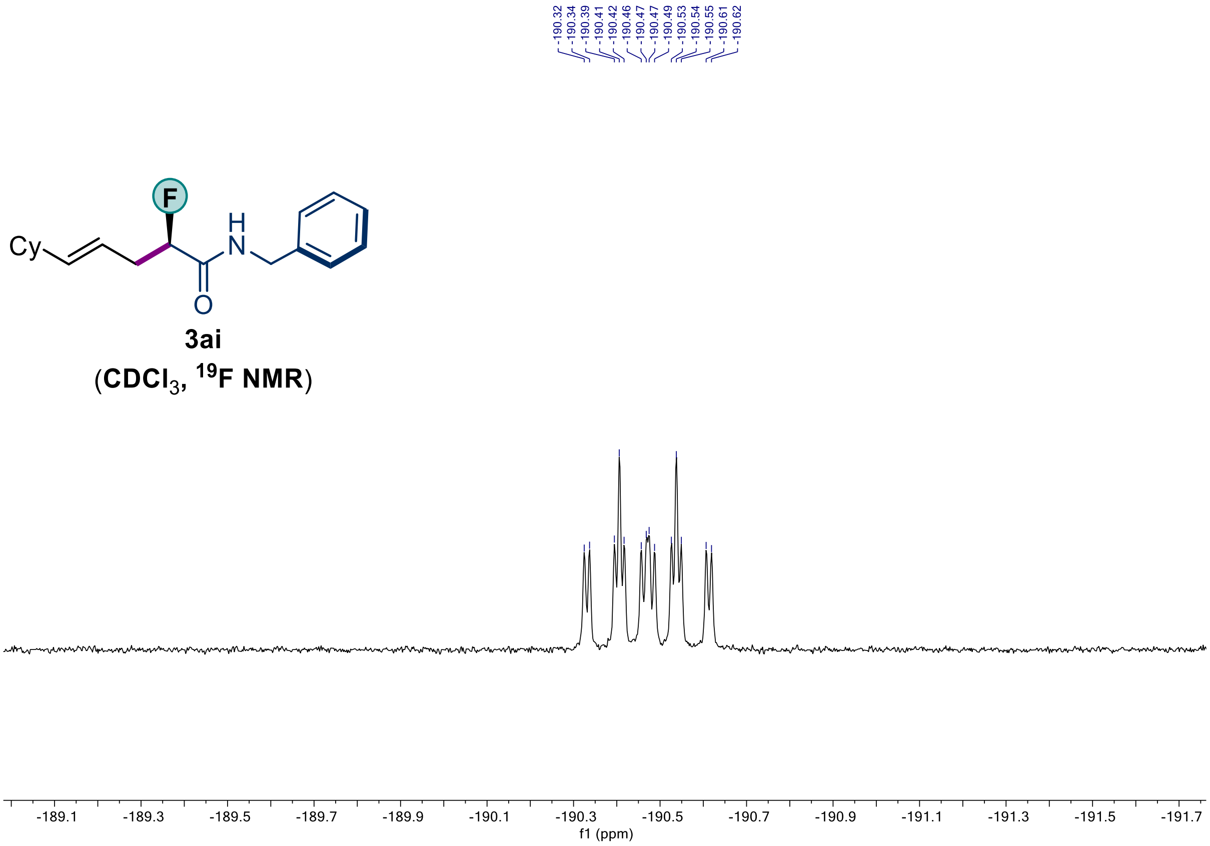

3ai
($CDCl_3$, ^{19}F NMR)

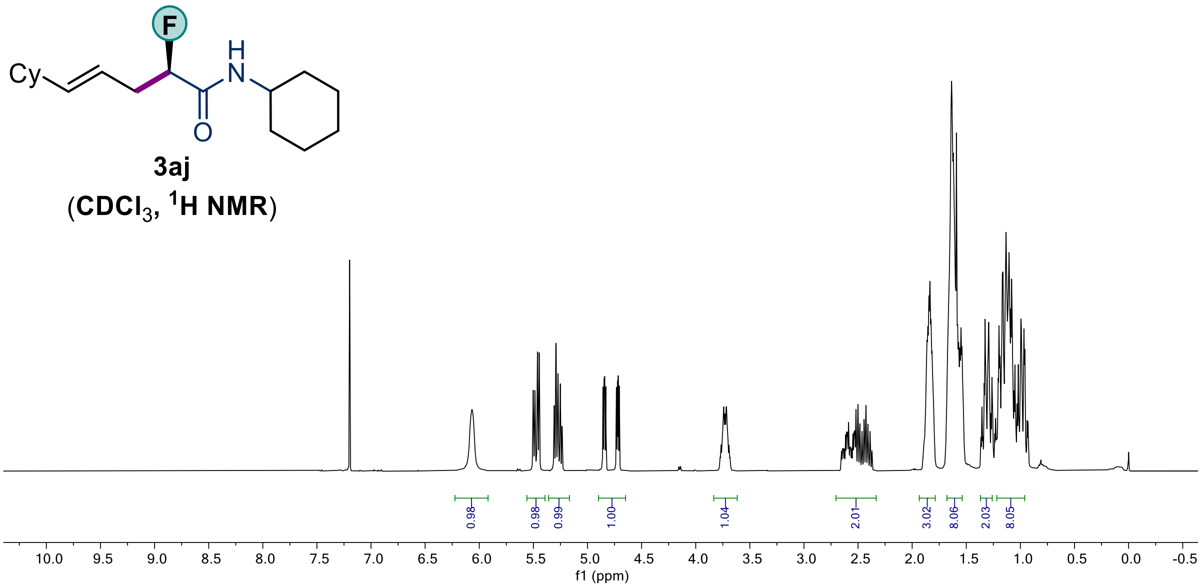
Cy
F
H
N
O
3aj
(CDCl3, 1H NMR)
0.98
0.98
0.99
1.00
1.04
2.01
3.02
8.06
2.03
8.05
f1 (ppm)

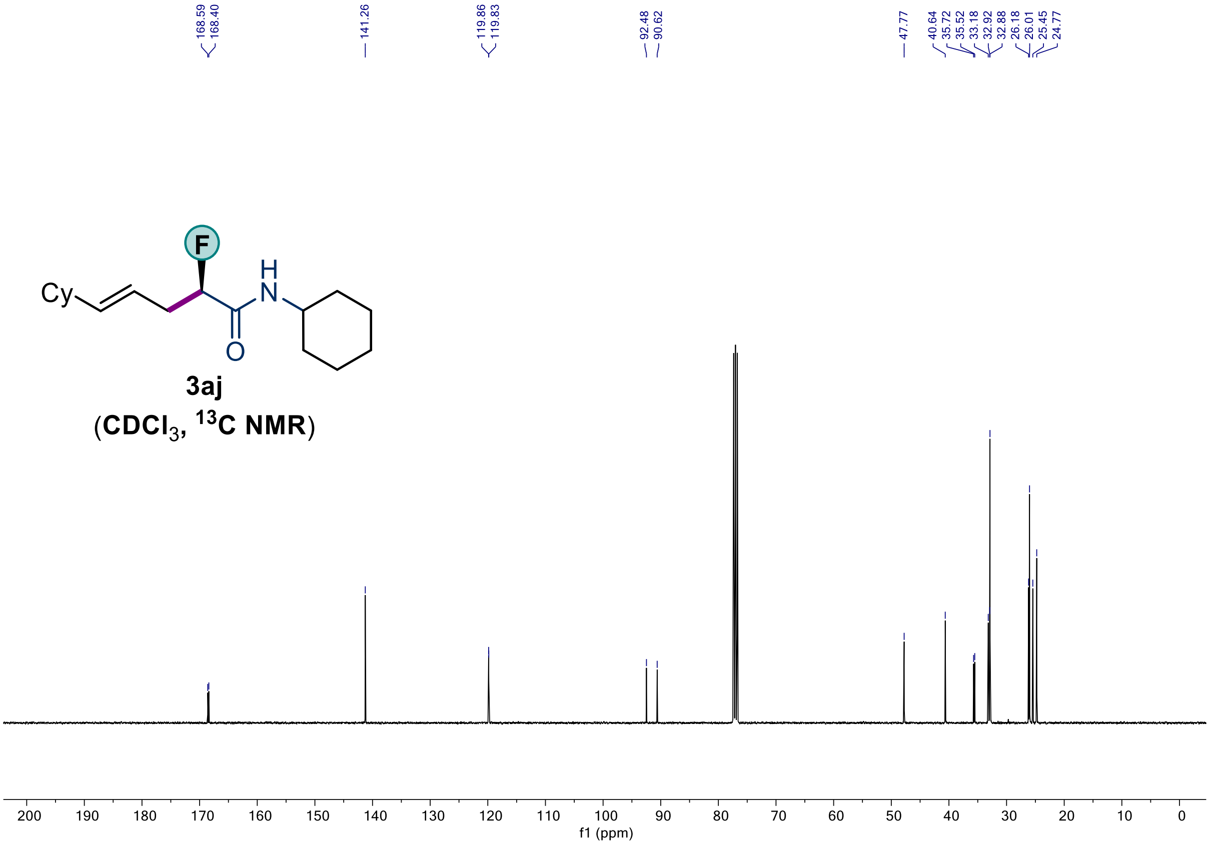
168.59
168.40
141.26
119.86
119.83
92.48
90.62
47.77
40.64
35.72
35.52
33.18
32.92
32.88
26.18
26.01
25.45
24.77
Cy
F
H
N
O
3aj
(CDCl3, 13C NMR)
f1 (ppm)

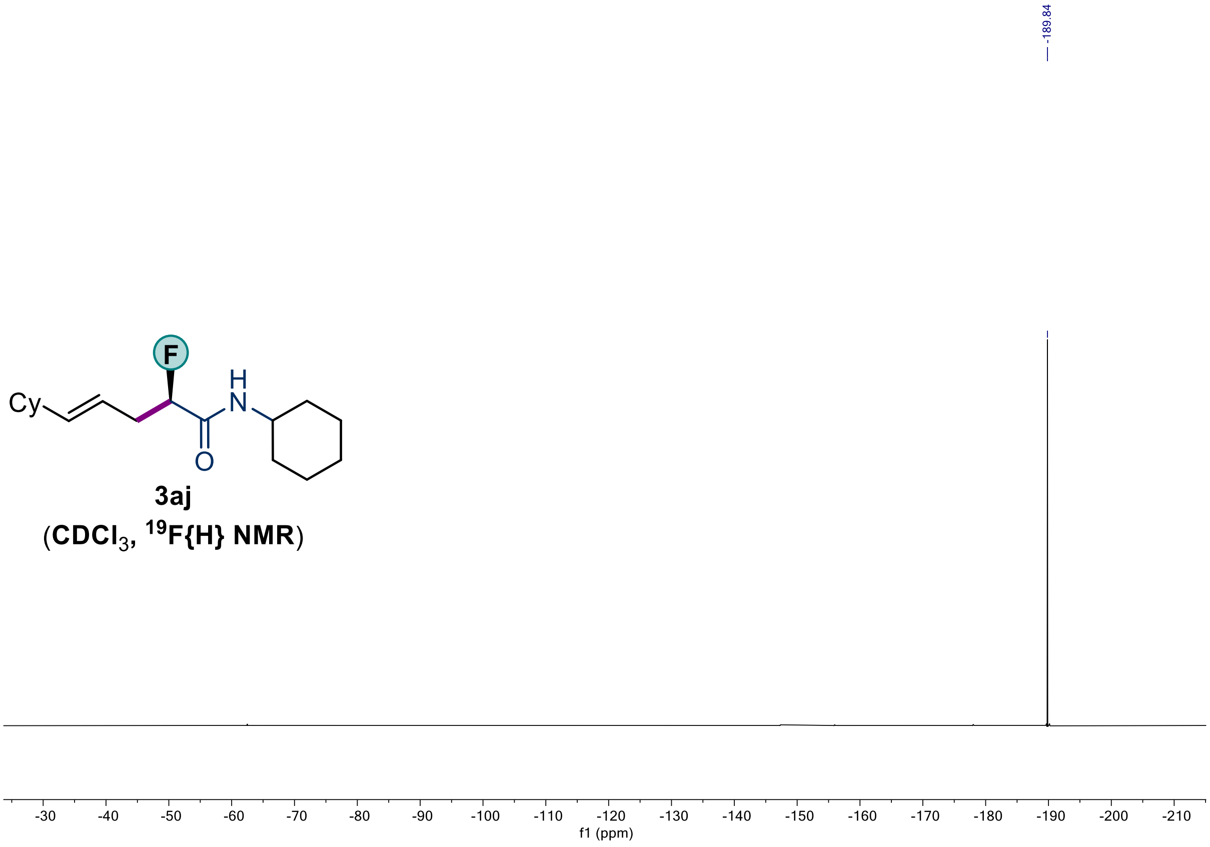

3aj
(**CDCl$_3$, ^{19}F{H} NMR**)

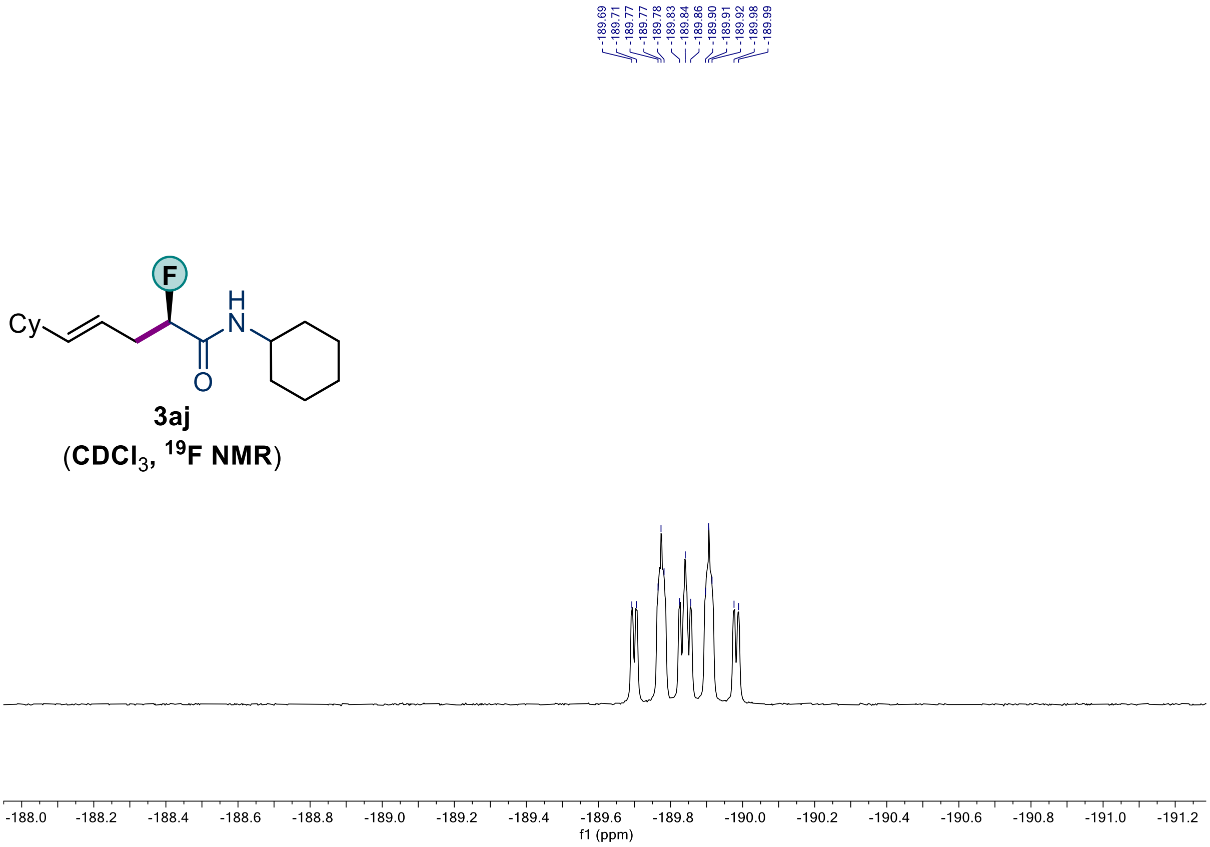

3aj
(**CDCl$_3$, ^{19}F NMR**)

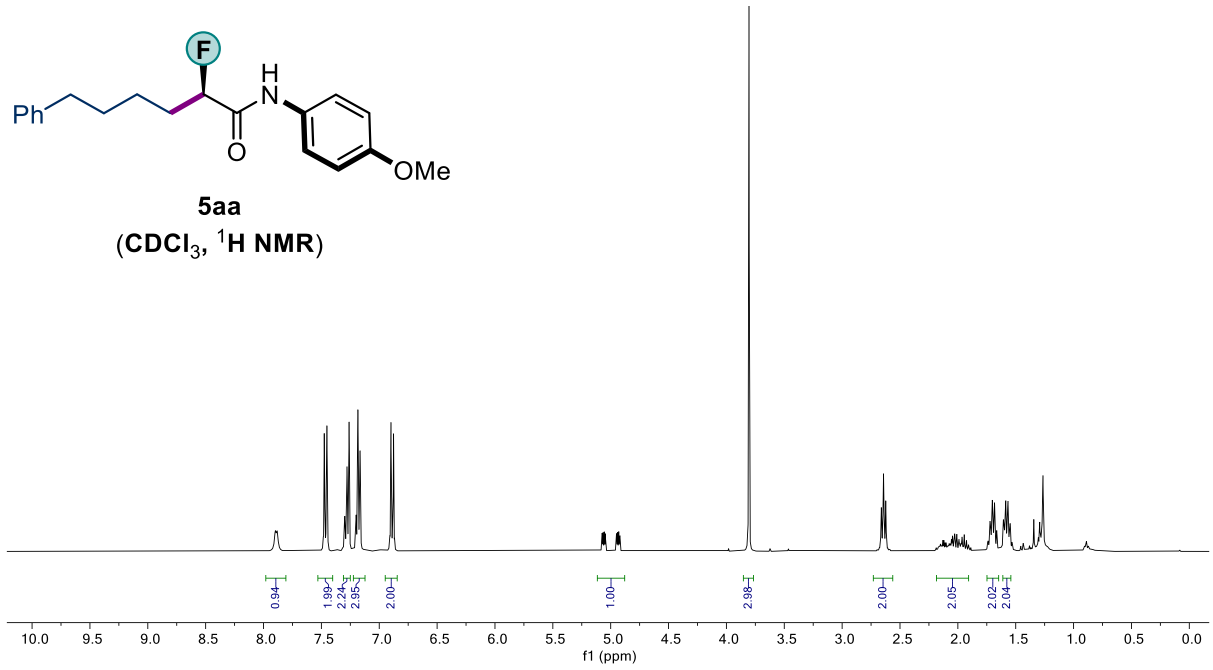

5aa

(**CDCl$_3$, ^{1}H NMR**)

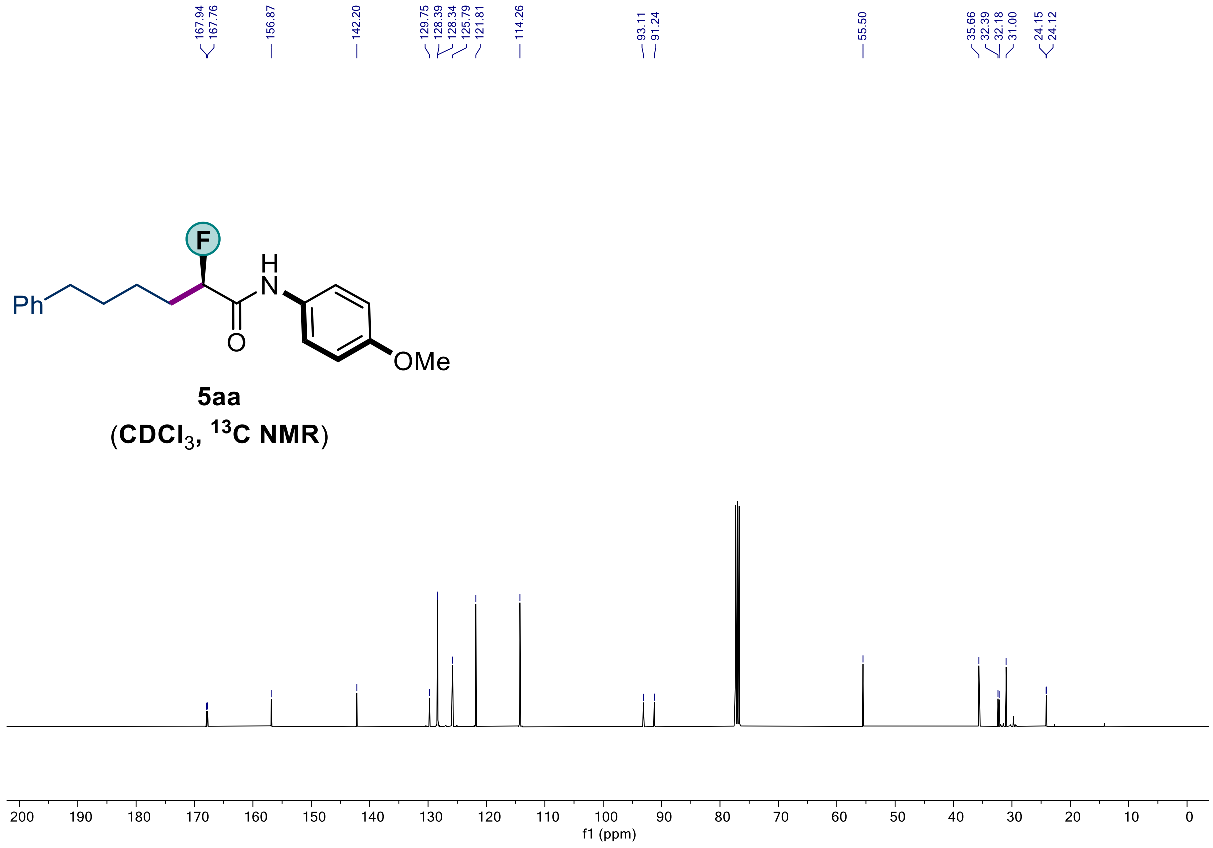

5aa

(**CDCl$_3$, ^{13}C NMR**)

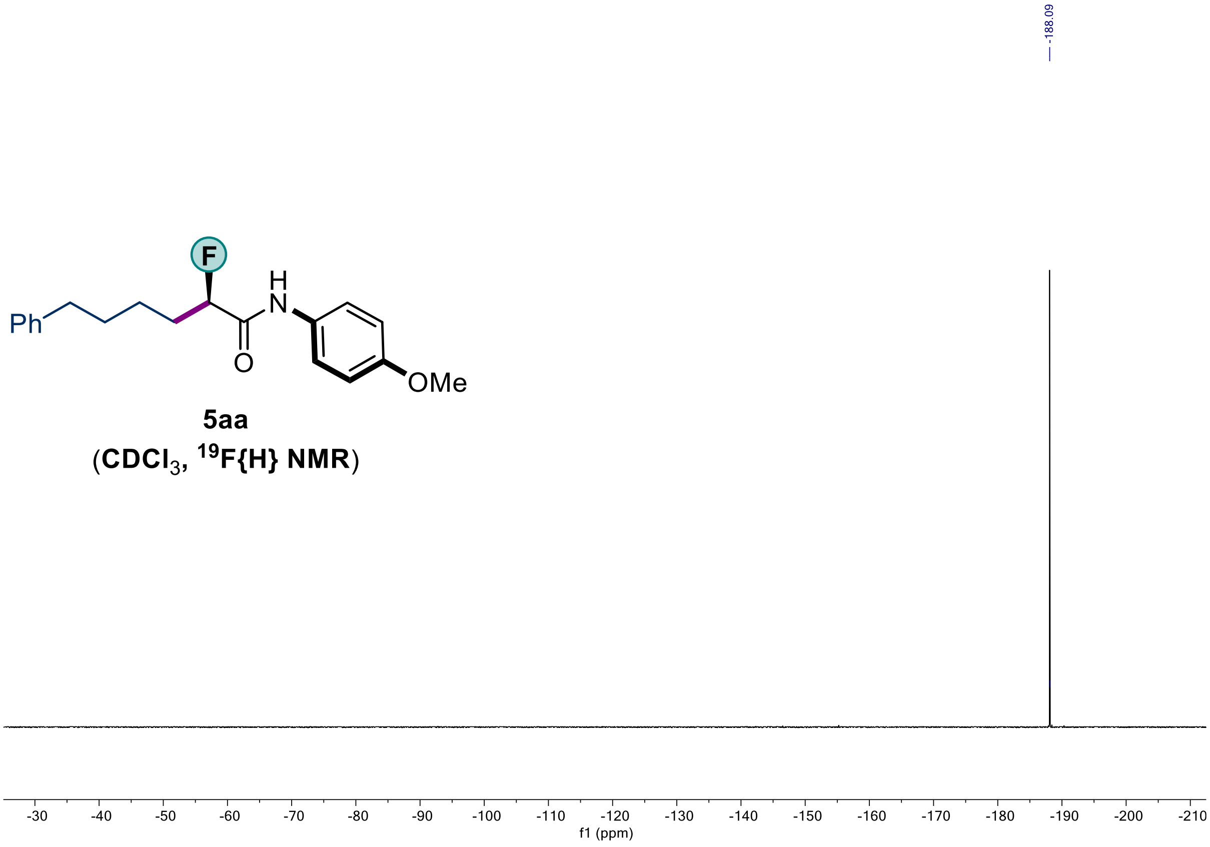

5aa
(**CDCl$_3$, ^{19}F{H} NMR**)

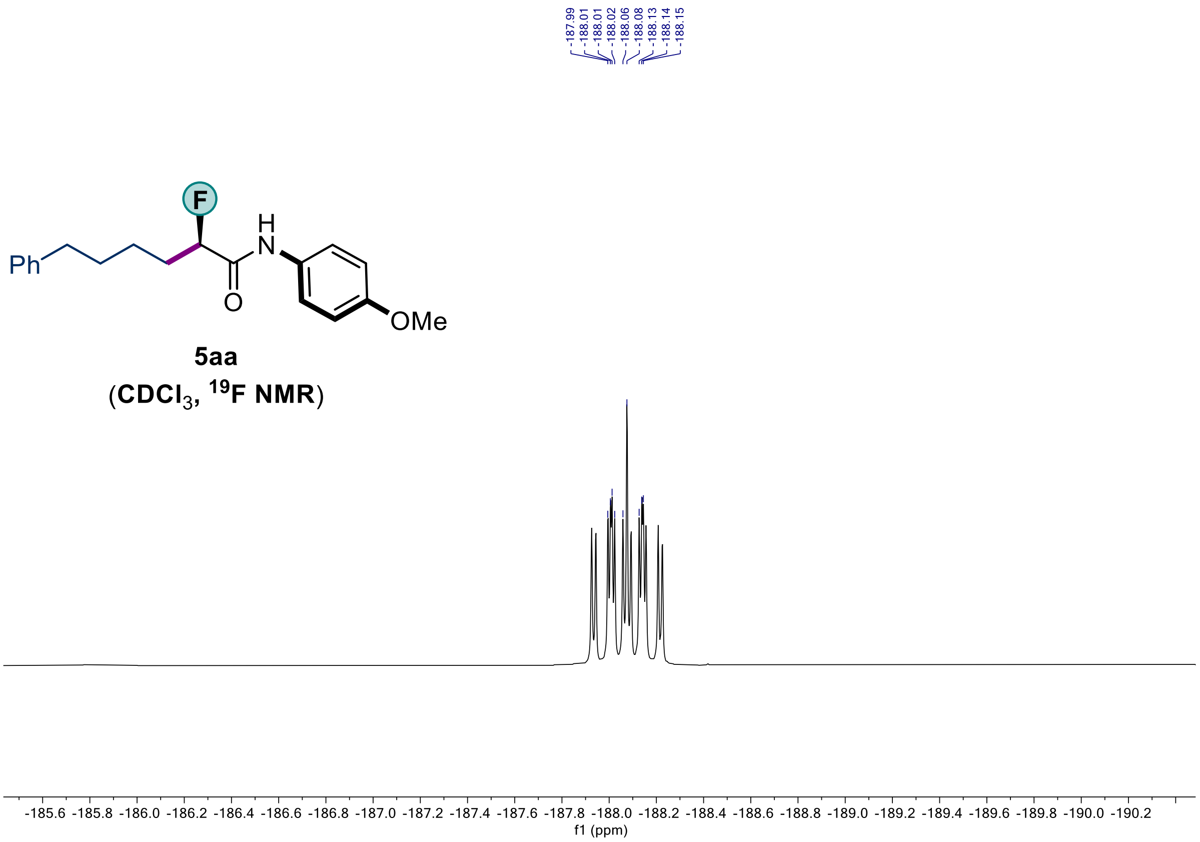

5aa
(**CDCl$_3$, ^{19}F NMR**)

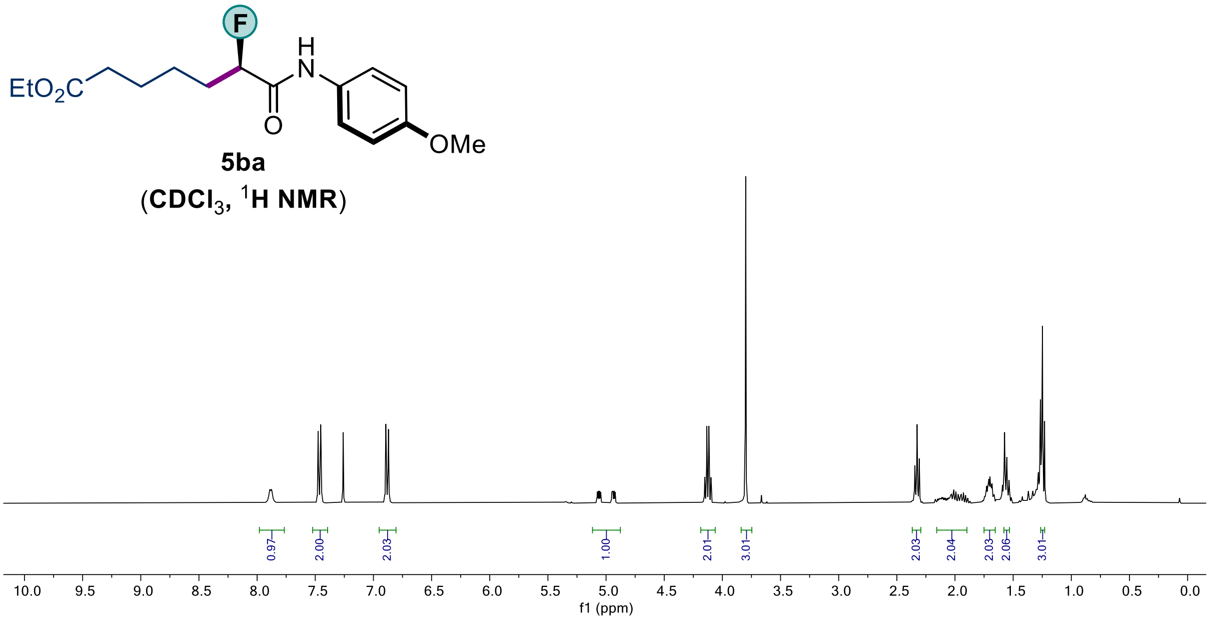
F
EtO2C
H
N
O
OMe
5ba
(CDCl3, 1H NMR)
0.97
2.00
2.03
1.00
2.01
3.01
2.03
2.04
2.03
2.06
3.01
10.0 9.5 9.0 8.5 8.0 7.5 7.0 6.5 6.0 5.5 5.0 4.5 4.0 3.5 3.0 2.5 2.0 1.5 1.0 0.5 0.0
f1 (ppm)

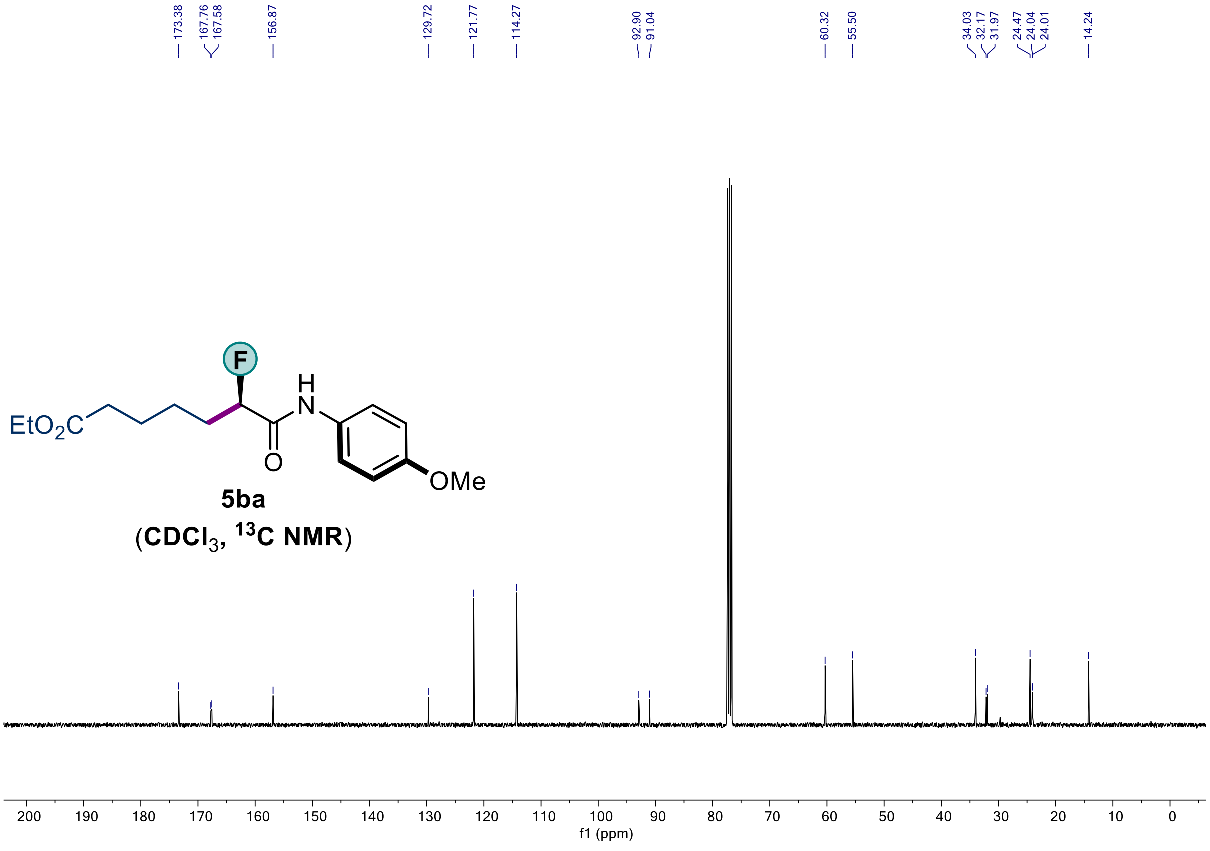
173.38
167.76
167.58
156.87
129.72
121.77
114.27
92.90
91.04
60.32
55.50
34.03
32.17
31.97
24.47
24.04
24.01
14.24
F
EtO2C
H
N
O
OMe
5ba
(CDCl3, 13C NMR)
200 190 180 170 160 150 140 130 120 110 100 90 80 70 60 50 40 30 20 10 0
f1 (ppm)

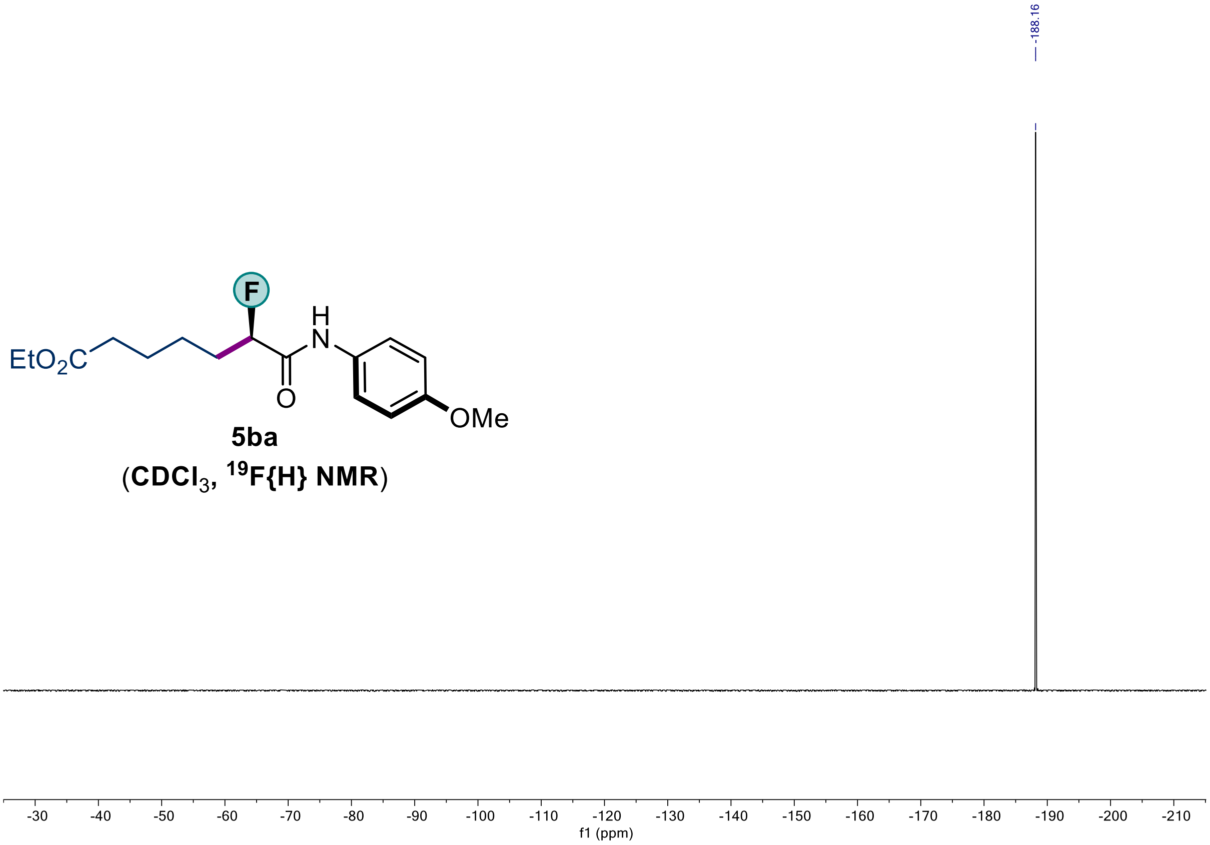
-188.16
EtO2C
F
H
N
O
OMe
5ba
(CDCl3, 19F{H} NMR)
f1 (ppm)

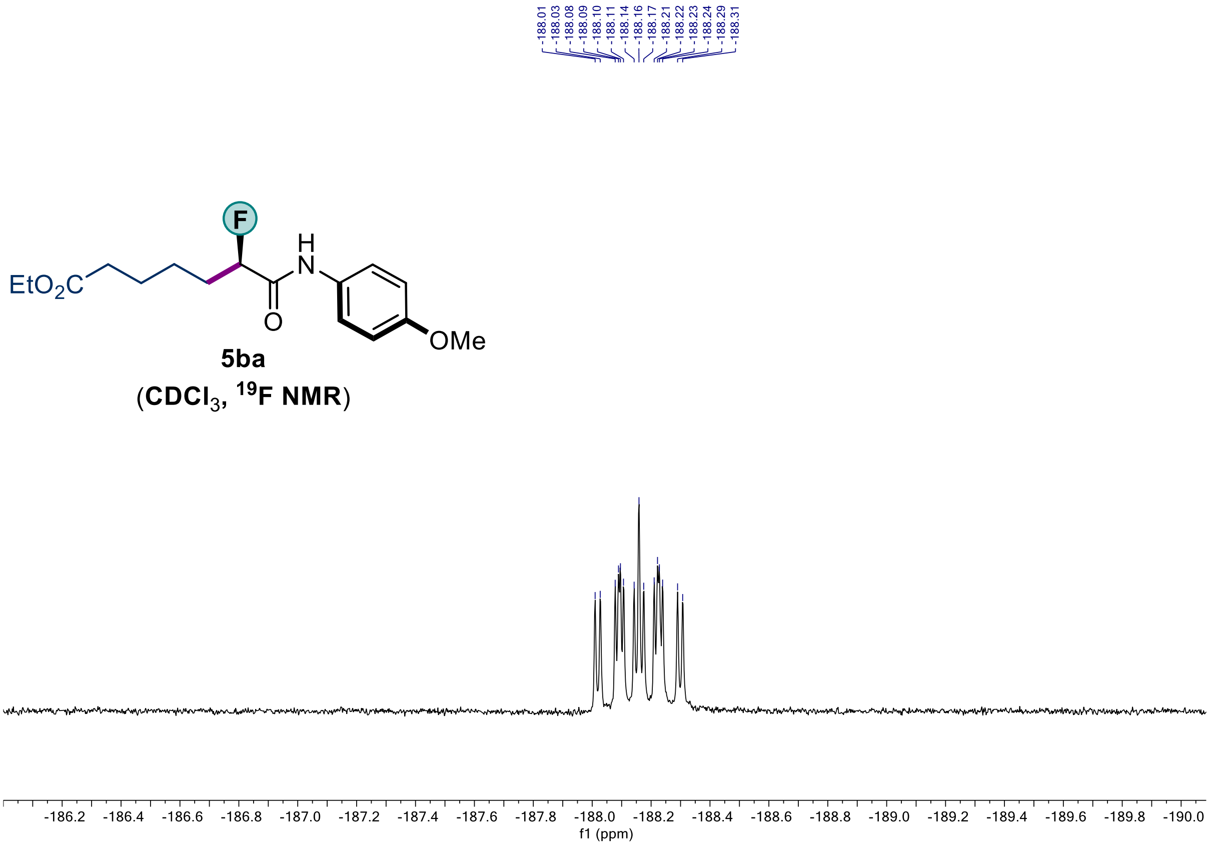
-188.01
-188.03
-188.08
-188.09
-188.10
-188.11
-188.14
-188.16
-188.17
-188.21
-188.22
-188.23
-188.24
-188.29
-188.31
EtO2C
F
H
N
O
OMe
5ba
(CDCl3, 19F NMR)
f1 (ppm)

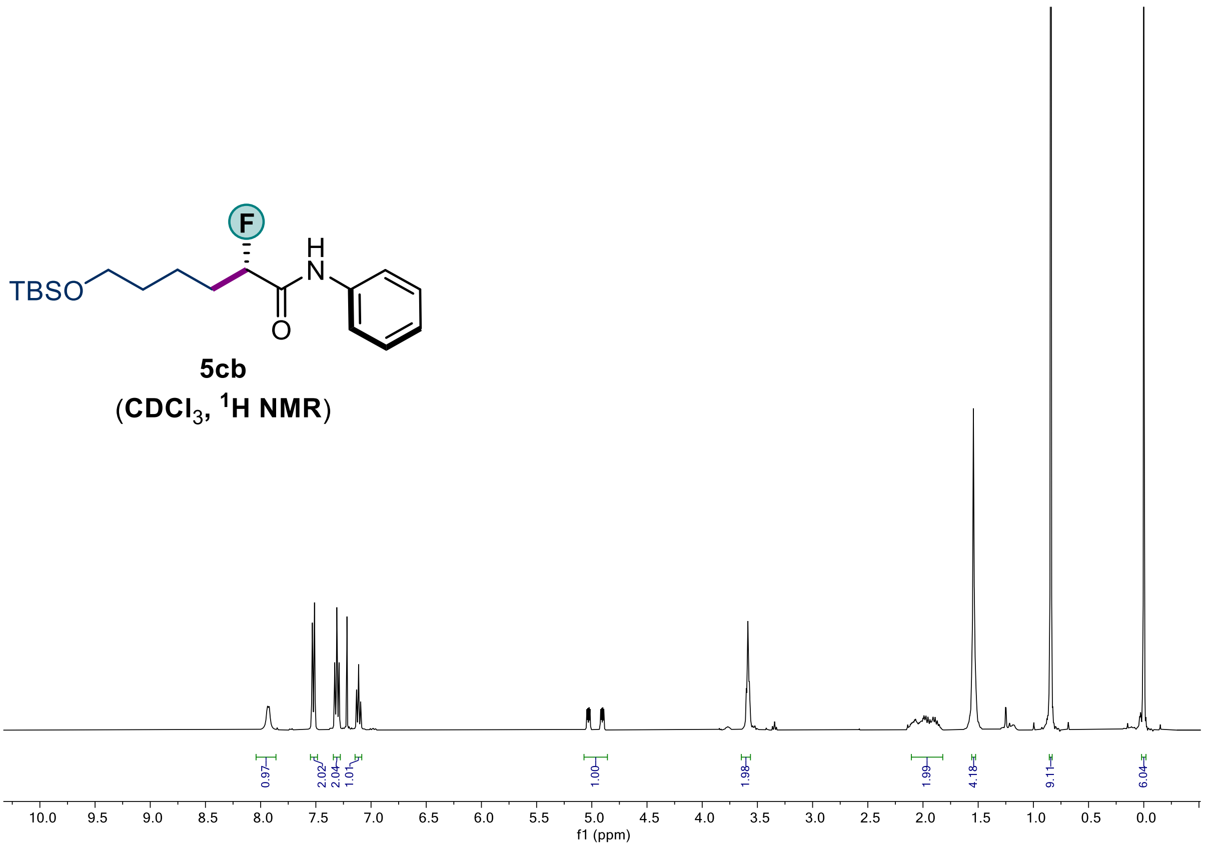
TBSO
F
H
N
O
5cb
(CDCl3, 1H NMR)
0.97
2.02
2.04
1.01
1.00
1.98
1.99
4.18
9.11
6.04
10.0 9.5 9.0 8.5 8.0 7.5 7.0 6.5 6.0 5.5 5.0 4.5 4.0 3.5 3.0 2.5 2.0 1.5 1.0 0.5 0.0
f1 (ppm)

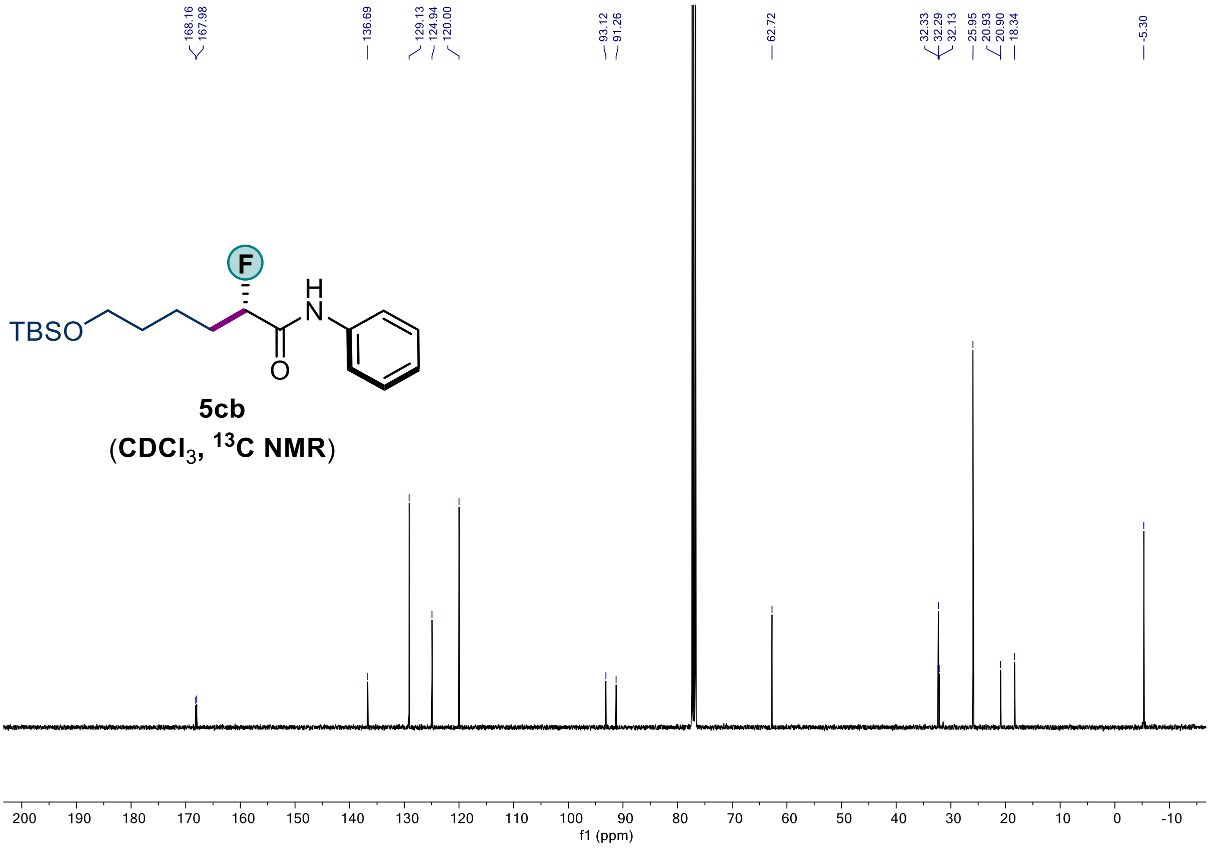
168.16
167.98
136.69
129.13
124.94
120.00
93.12
91.26
62.72
32.33
32.29
32.13
25.95
20.93
20.90
18.34
-5.30
TBSO
F
H
N
O
5cb
(CDCl3, 13C NMR)
200 190 180 170 160 150 140 130 120 110 100 90 80 70 60 50 40 30 20 10 0 -10
f1 (ppm)

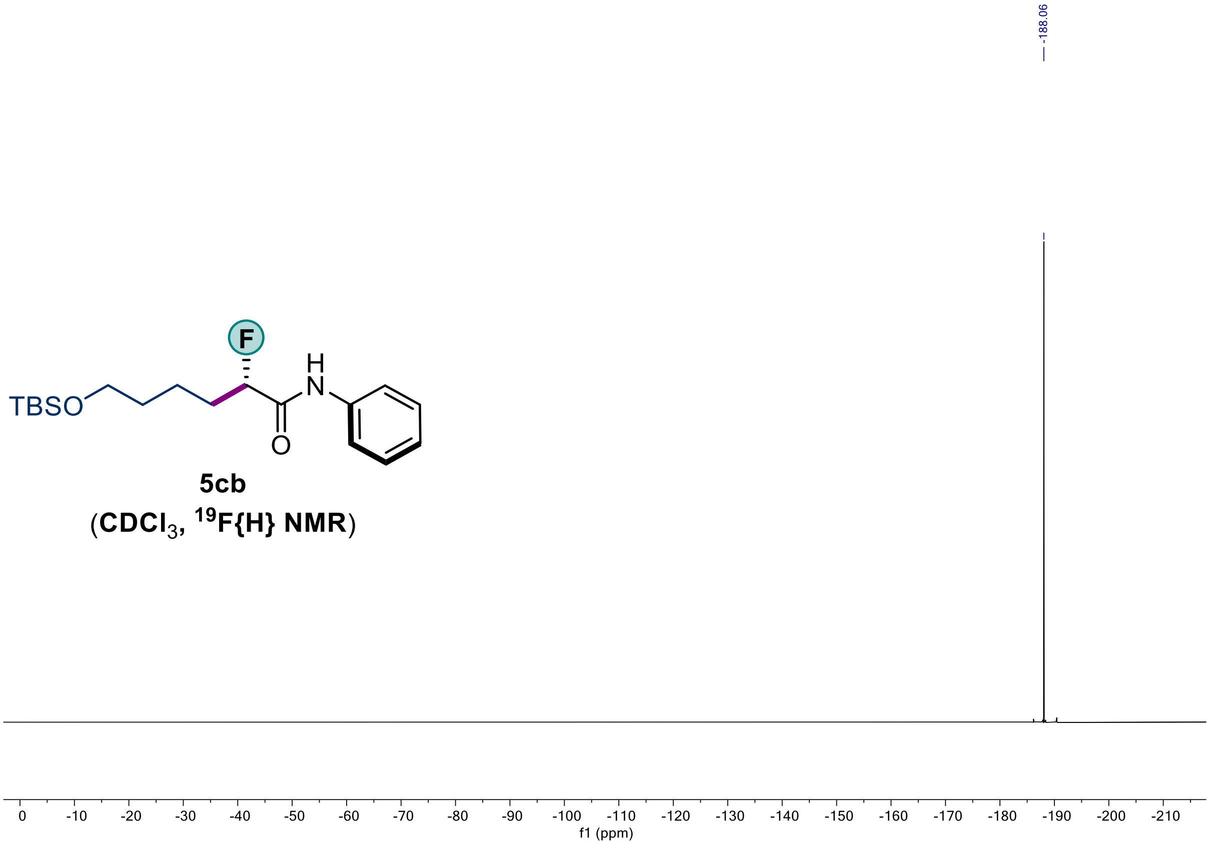
-188.06
TBSO
F
H
N
O
5cb
(CDCl$_3$, ^{19}F{H} NMR)
0 -10 -20 -30 -40 -50 -60 -70 -80 -90 -100 -110 -120 -130 -140 -150 -160 -170 -180 -190 -200 -210
f1 (ppm)

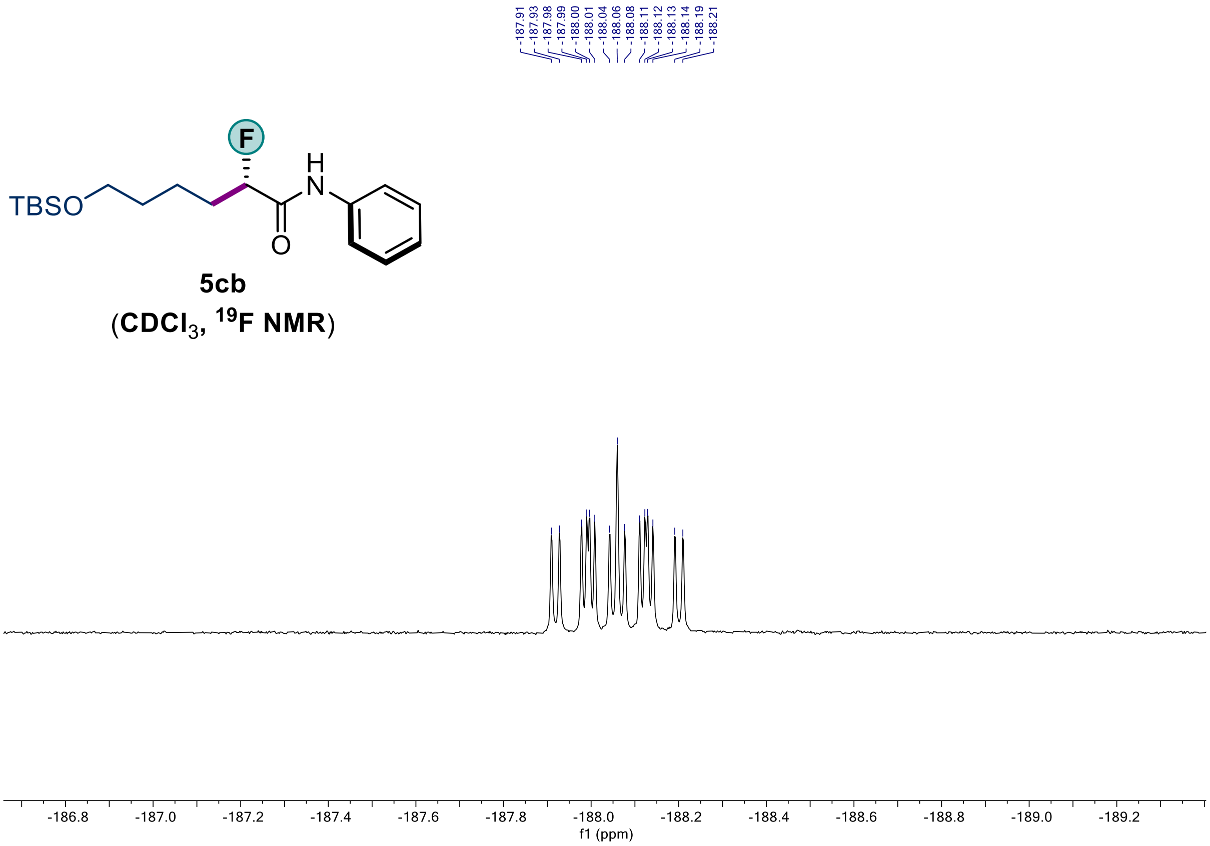
-187.91 -187.93 -187.98 -187.99 -188.00 -188.01 -188.04 -188.06 -188.08 -188.11 -188.12 -188.13 -188.14 -188.19 -188.21
TBSO
F
H
N
O
5cb
(CDCl$_3$, ^{19}F NMR)
-186.8 -187.0 -187.2 -187.4 -187.6 -187.8 -188.0 -188.2 -188.4 -188.6 -188.8 -189.0 -189.2
f1 (ppm)

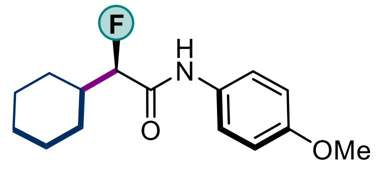

5da

(CDCl$_3$, ^{1}H NMR)

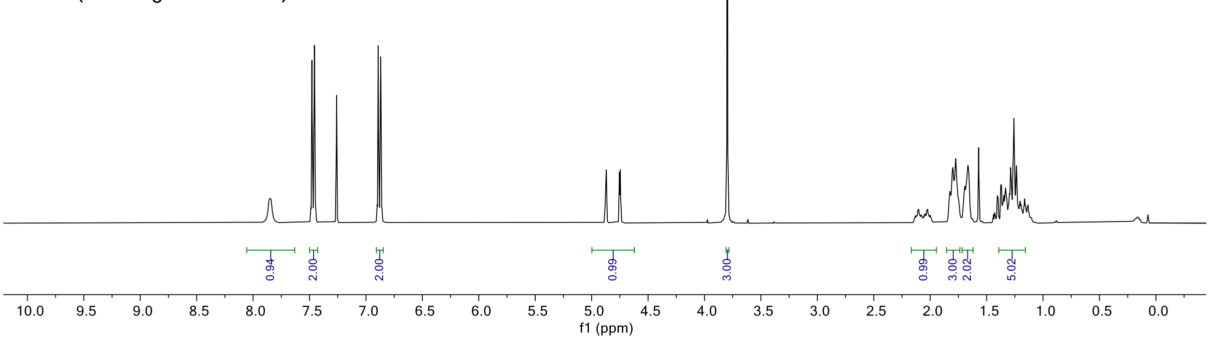

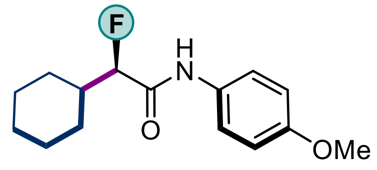

5da

(CDCl$_3$, ^{13}C NMR)

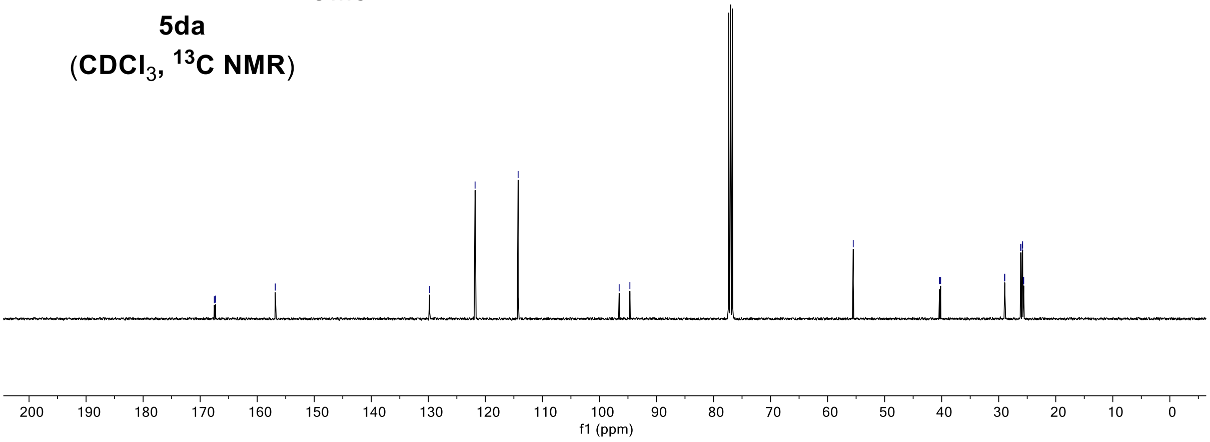

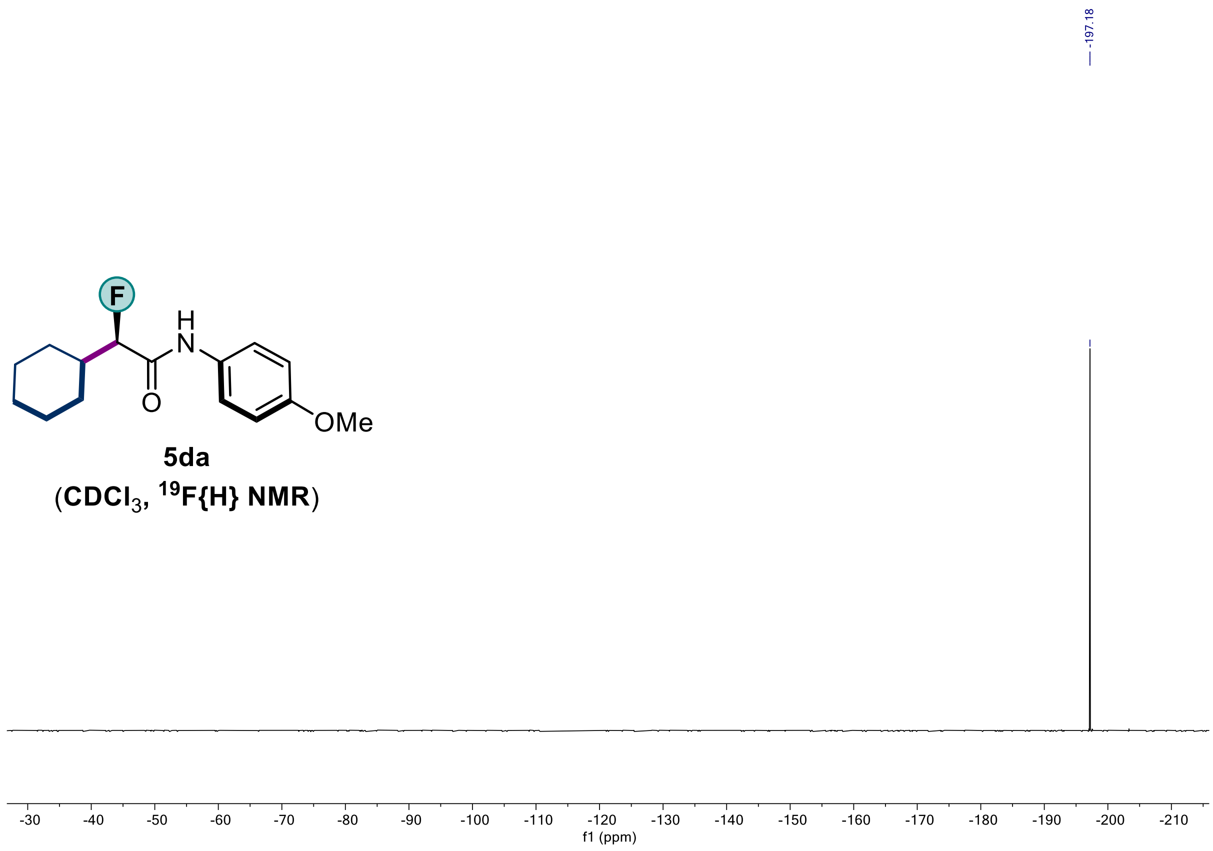
-197.18
5da
(CDCl3, 19F{H} NMR)
OMe
-30 -40 -50 -60 -70 -80 -90 -100 -110 -120 -130 -140 -150 -160 -170 -180 -190 -200 -210
f1 (ppm)

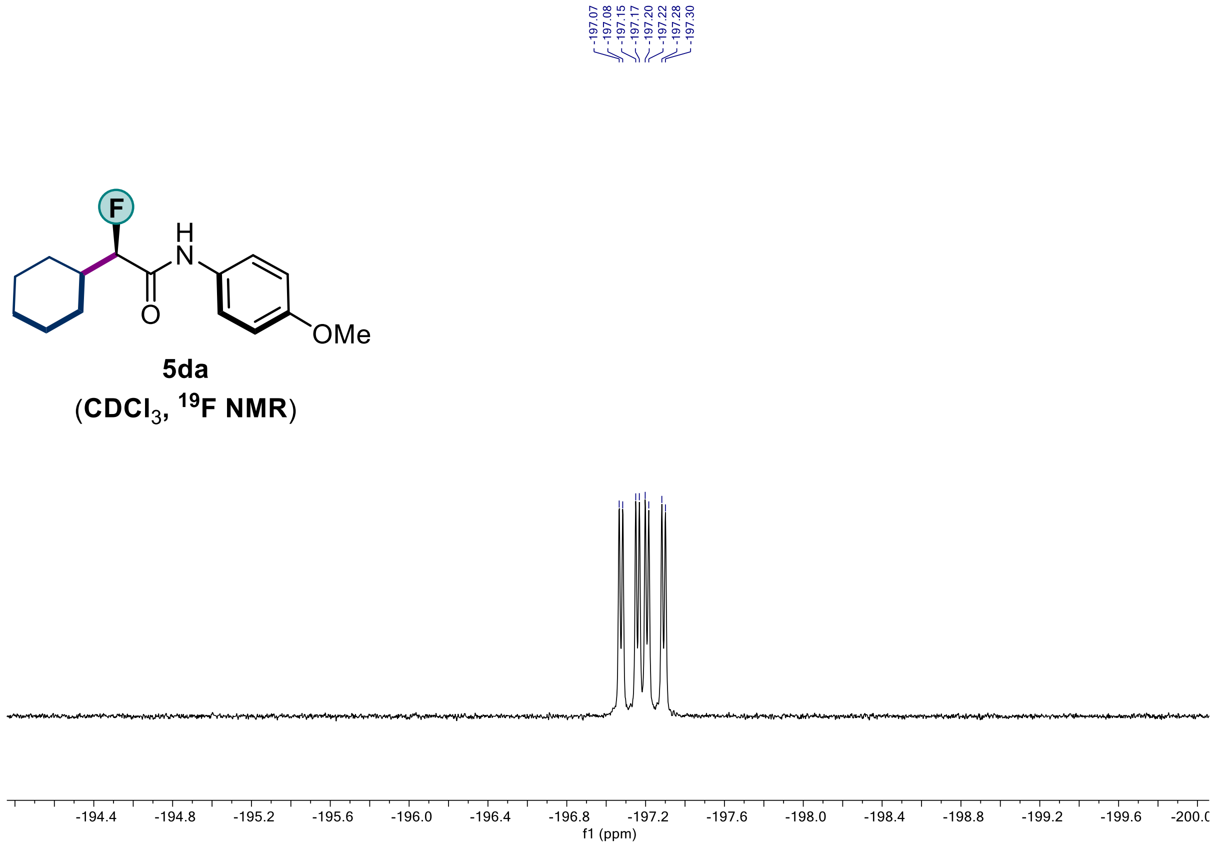
-197.07
-197.08
-197.15
-197.17
-197.20
-197.22
-197.28
-197.30
5da
(CDCl3, 19F NMR)
OMe
-194.4 -194.8 -195.2 -195.6 -196.0 -196.4 -196.8 -197.2 -197.6 -198.0 -198.4 -198.8 -199.2 -199.6
f1 (ppm)

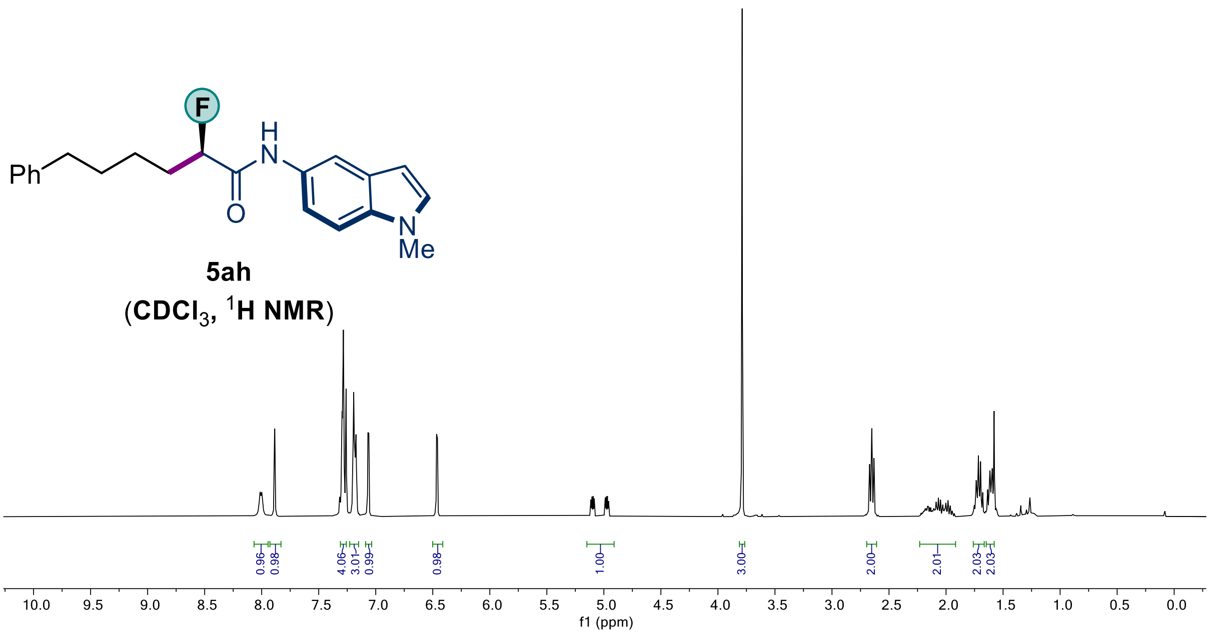

5ah
($CDCl_3$, ^1H **NMR**)

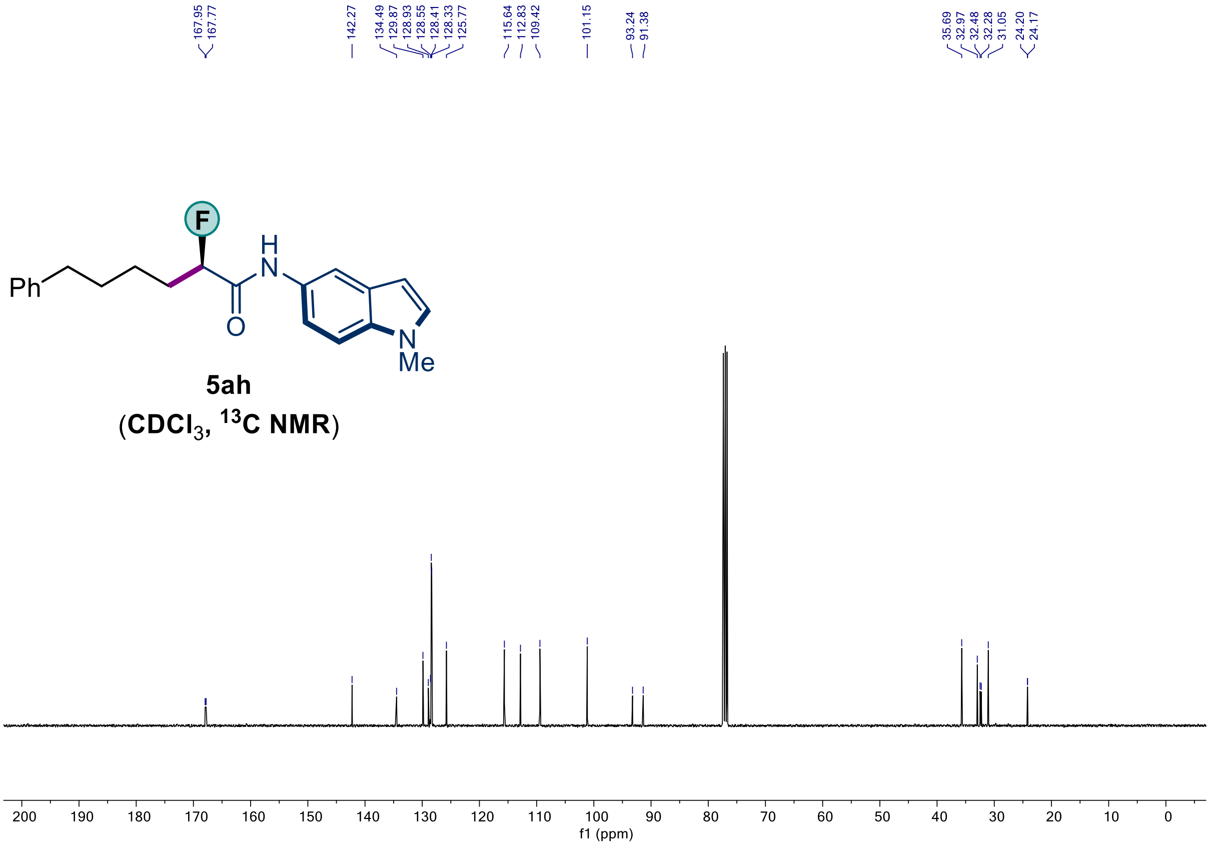

5ah
($CDCl_3$, ^{13}C **NMR**)

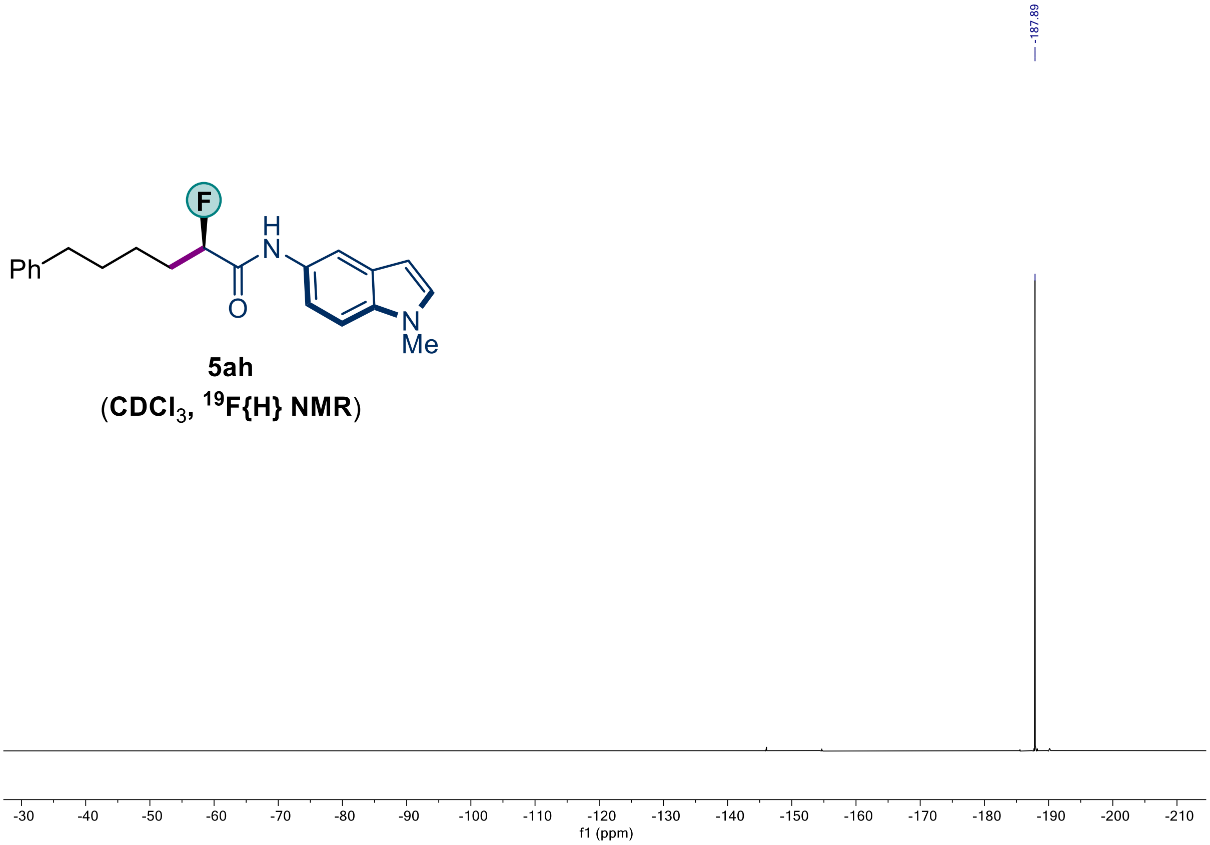

5ah
(CDCl$_3$, ^{19}F{H} NMR)

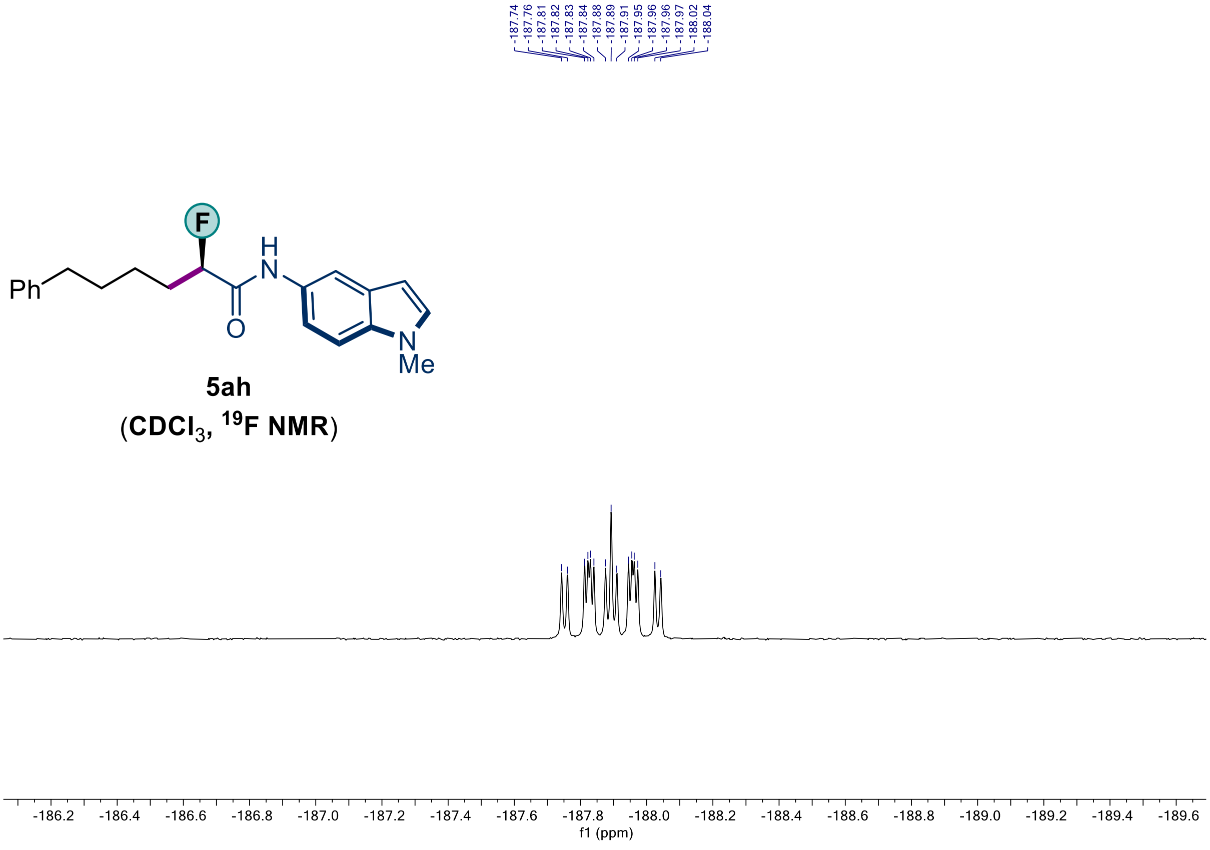

5ah
(CDCl$_3$, ^{19}F NMR)

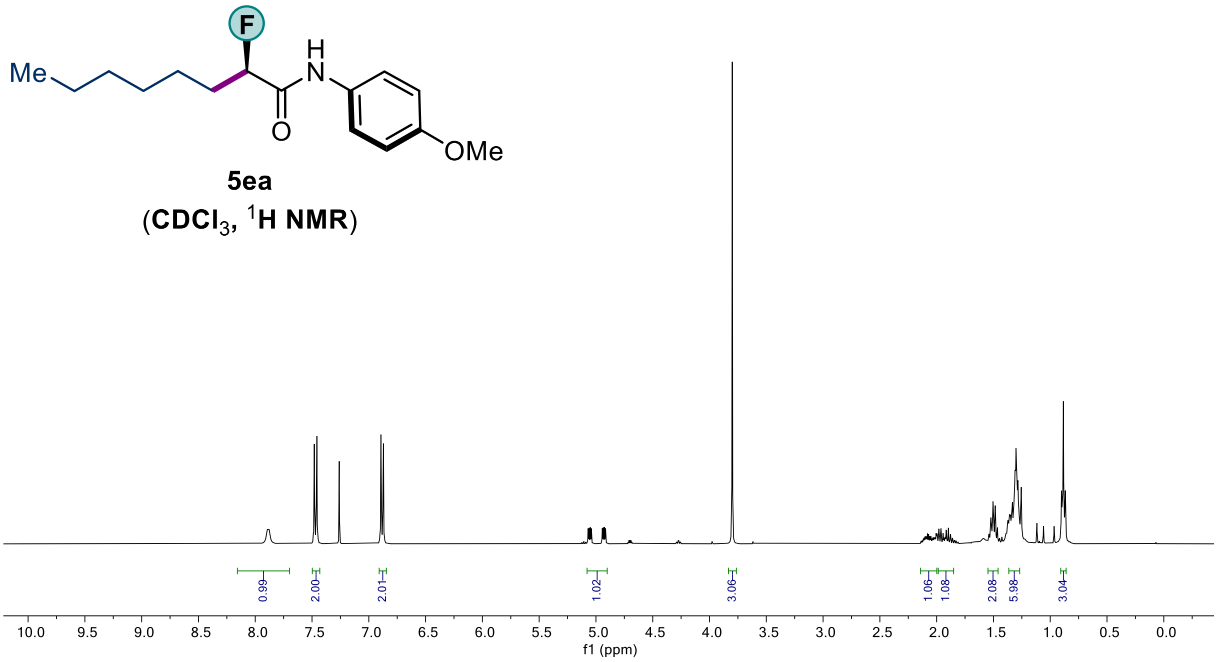

5ea
(CDCl$_3$, ^{1}H NMR)

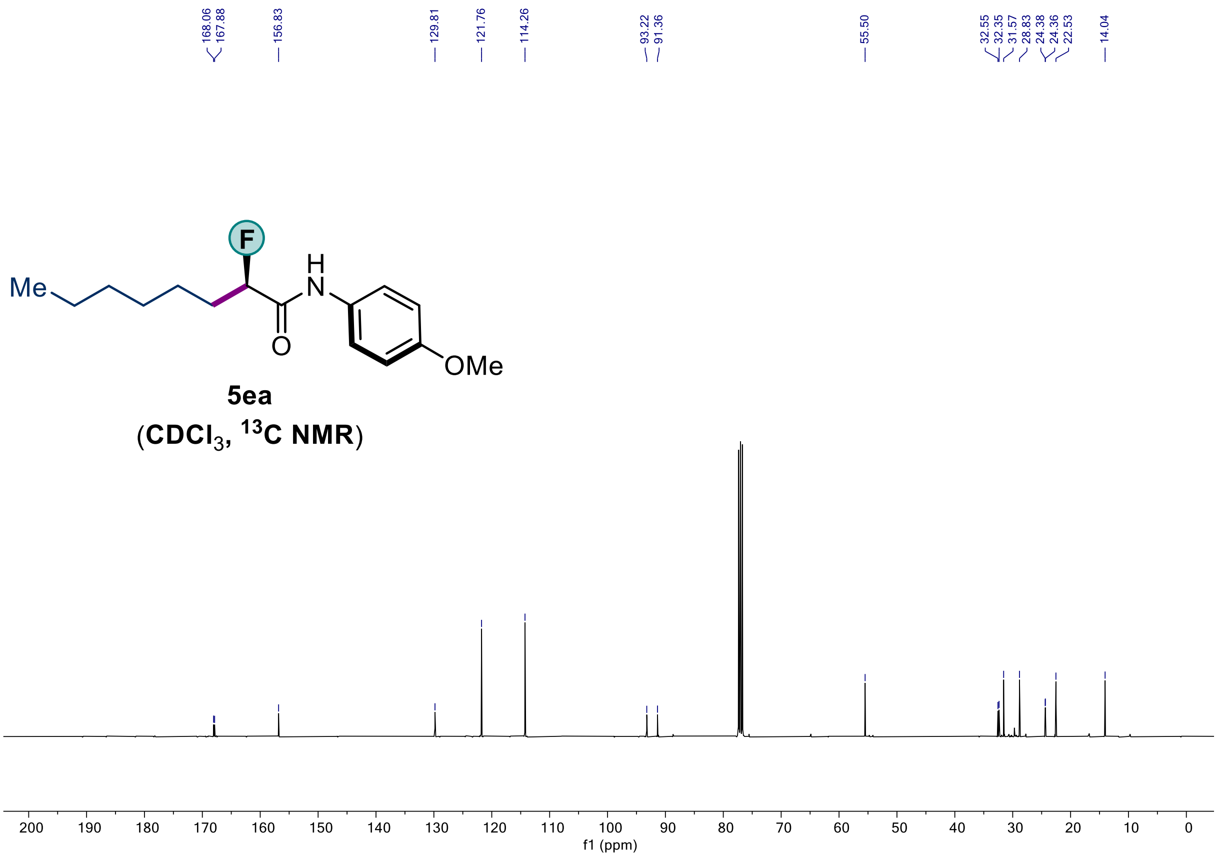

5ea
(CDCl$_3$, ^{13}C NMR)

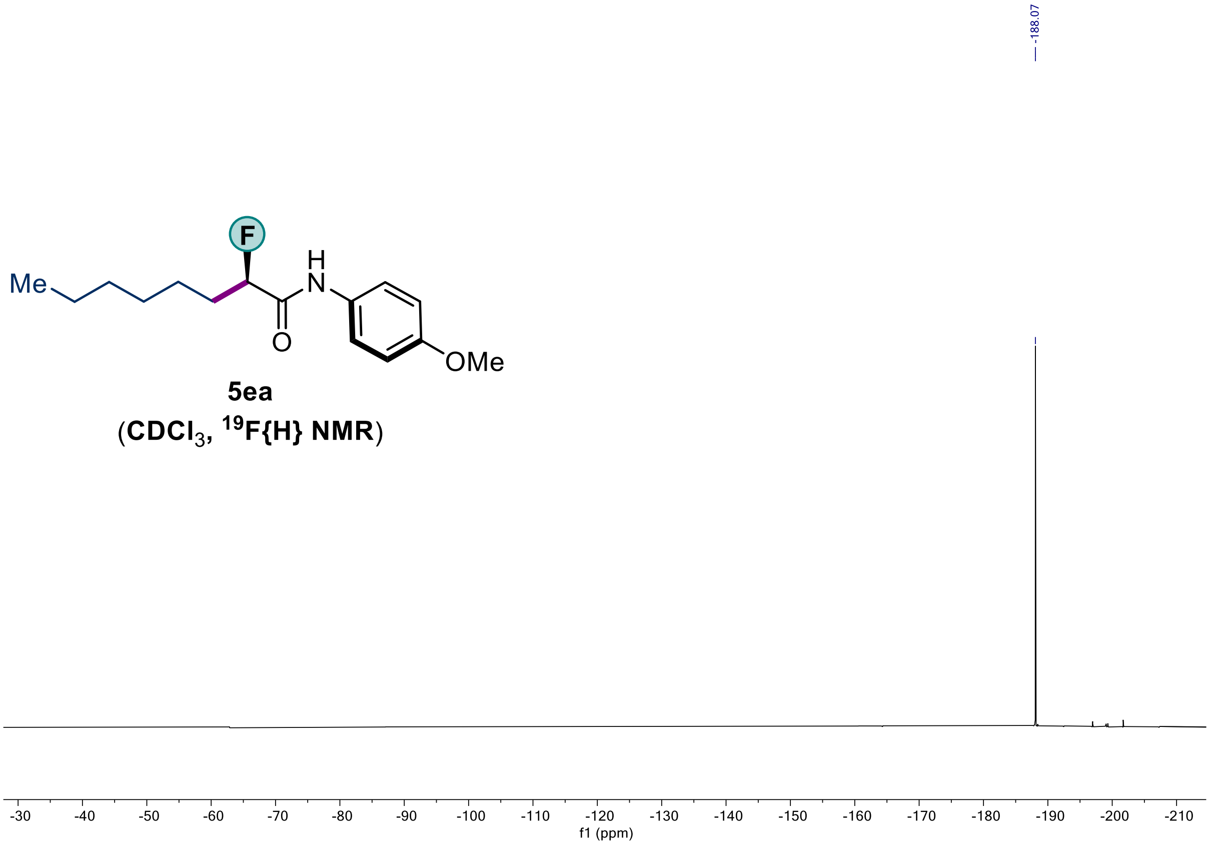
-188.07
Me
F
H
N
O
OMe
5ea
(CDCl3, 19F{H} NMR)
-30 -40 -50 -60 -70 -80 -90 -100 -110 -120 -130 -140 -150 -160 -170 -180 -190 -200 -210
f1 (ppm)

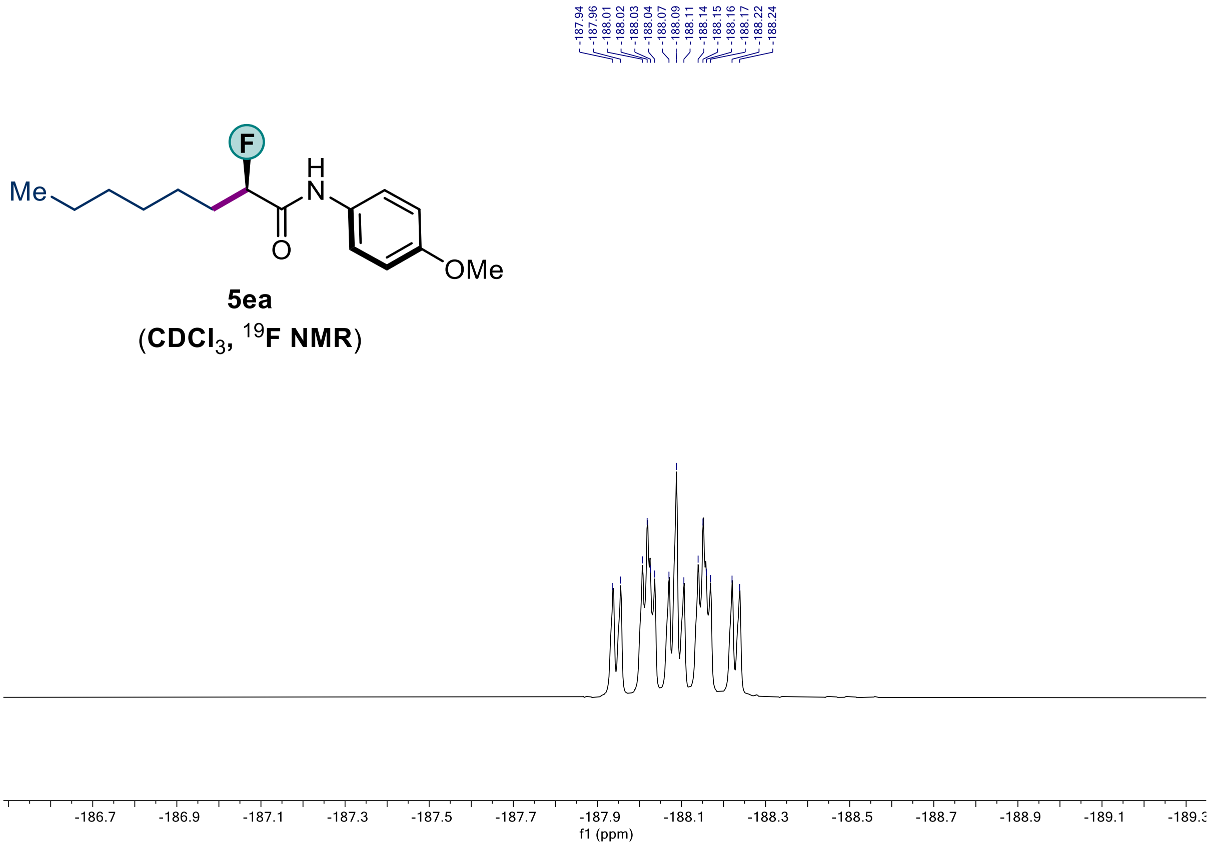
-187.94
-187.96
-188.01
-188.02
-188.03
-188.04
-188.07
-188.09
-188.11
-188.14
-188.15
-188.16
-188.17
-188.22
-188.24
Me
F
H
N
O
OMe
5ea
(CDCl3, 19F NMR)
-186.7 -186.9 -187.1 -187.3 -187.5 -187.7 -187.9 -188.1 -188.3 -188.5 -188.7 -188.9 -189.1
f1 (ppm)

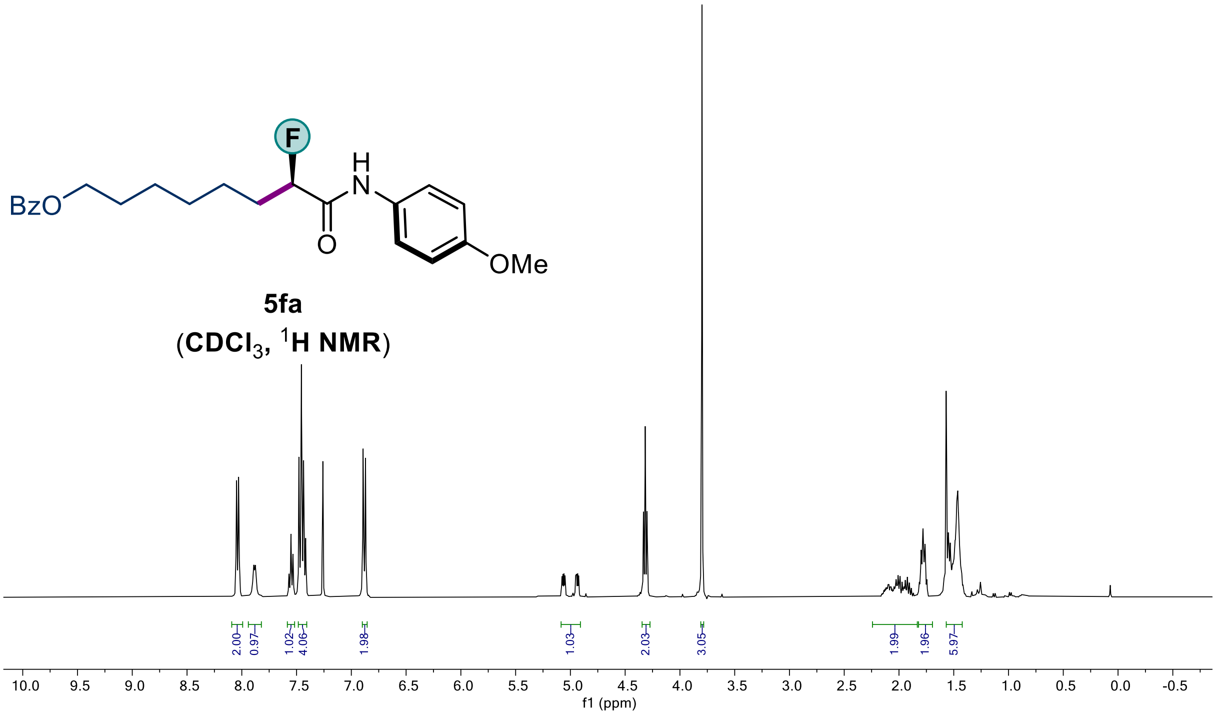

BzO
F
H
N
O
OMe
5fa
(CDCl3, 1H NMR)
2.00 0.97 1.02 4.06 1.98 1.03 2.03 3.05 1.99 1.96 5.97
10.0 9.5 9.0 8.5 8.0 7.5 7.0 6.5 6.0 5.5 5.0 4.5 4.0 3.5 3.0 2.5 2.0 1.5 1.0 0.5 0.0 -0.5
f1 (ppm)

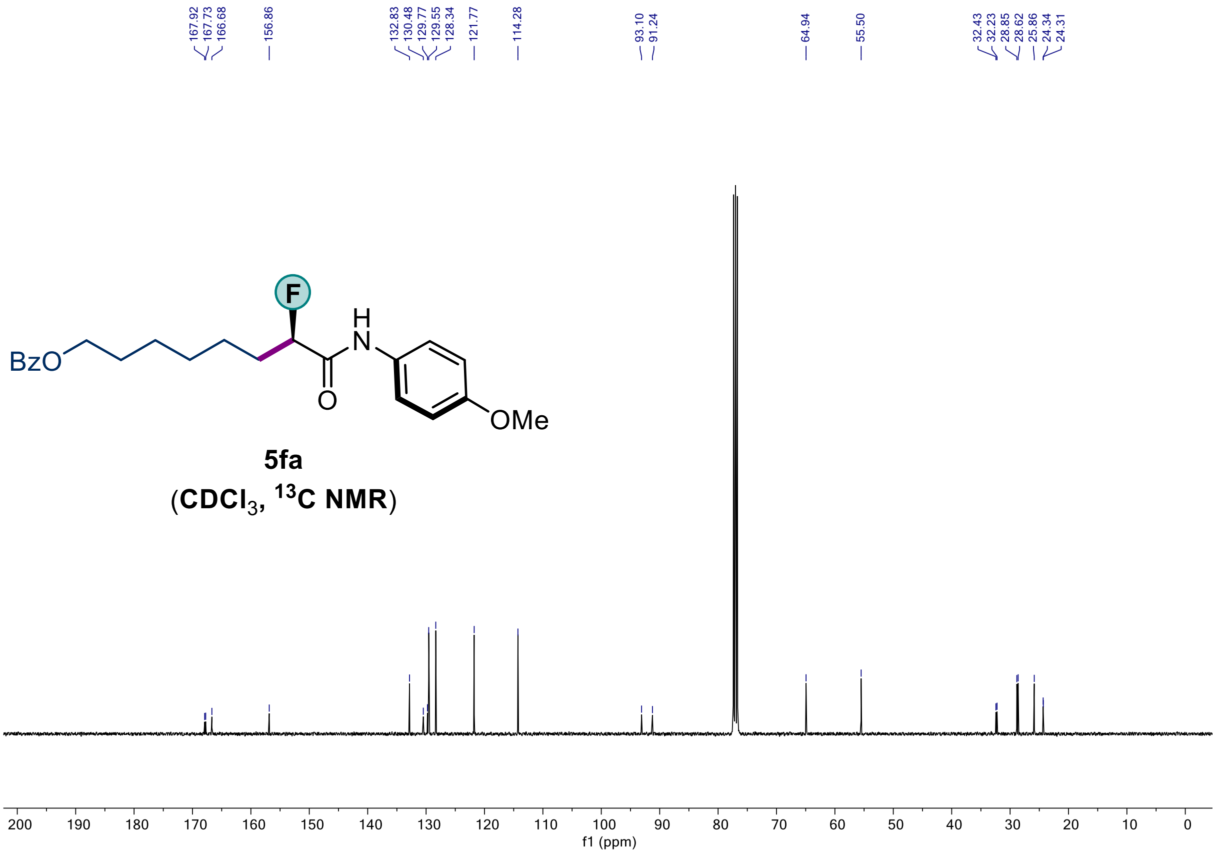

167.92 167.73 166.68 156.86 132.83 130.48 129.77 129.55 128.34 121.77 114.28 93.10 91.24 64.94 55.50 32.43 32.23 28.85 28.62 25.86 24.34 24.31
BzO
F
H
N
O
OMe
5fa
(CDCl3, 13C NMR)
200 190 180 170 160 150 140 130 120 110 100 90 80 70 60 50 40 30 20 10 0
f1 (ppm)

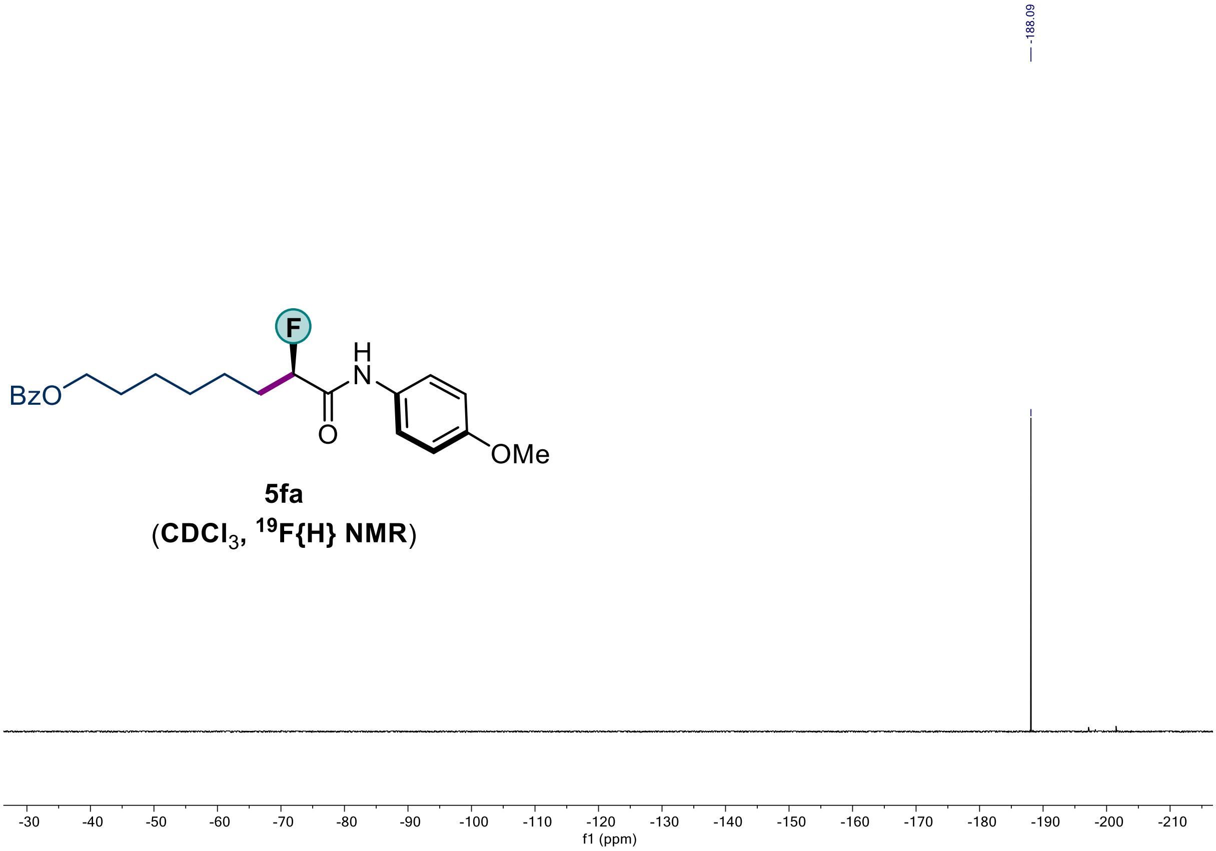
-188.09
BzO
F
H
N
O
OMe
5fa
(CDCl3, 19F{H} NMR)
-30 -40 -50 -60 -70 -80 -90 -100 -110 -120 -130 -140 -150 -160 -170 -180 -190 -200 -210
f1 (ppm)

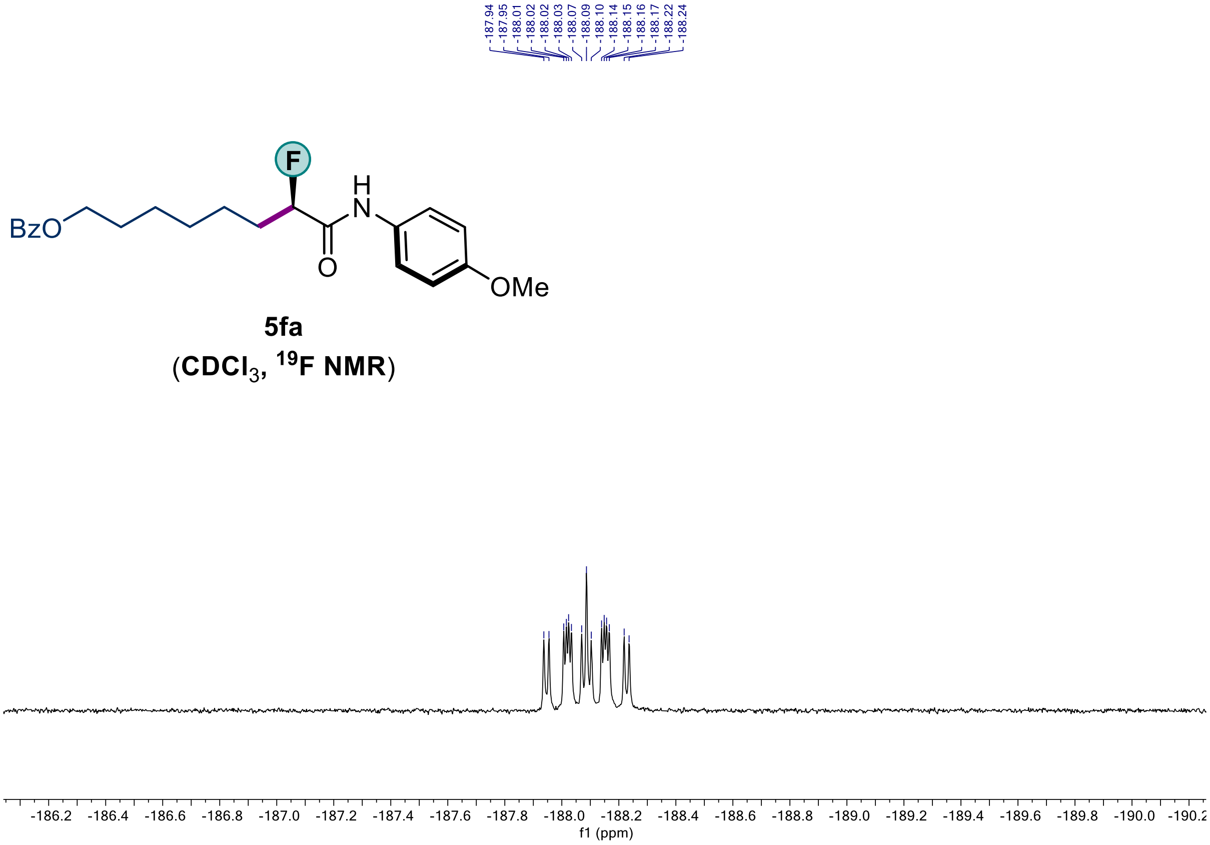
-187.94
-187.95
-188.01
-188.02
-188.03
-188.07
-188.09
-188.10
-188.14
-188.15
-188.16
-188.17
-188.22
-188.24
BzO
F
H
N
O
OMe
5fa
(CDCl3, 19F NMR)
-186.2 -186.4 -186.6 -186.8 -187.0 -187.2 -187.4 -187.6 -187.8 -188.0 -188.2 -188.4 -188.6 -188.8 -189.0 -189.2 -189.4 -189.6 -189.8 -190.0
f1 (ppm)

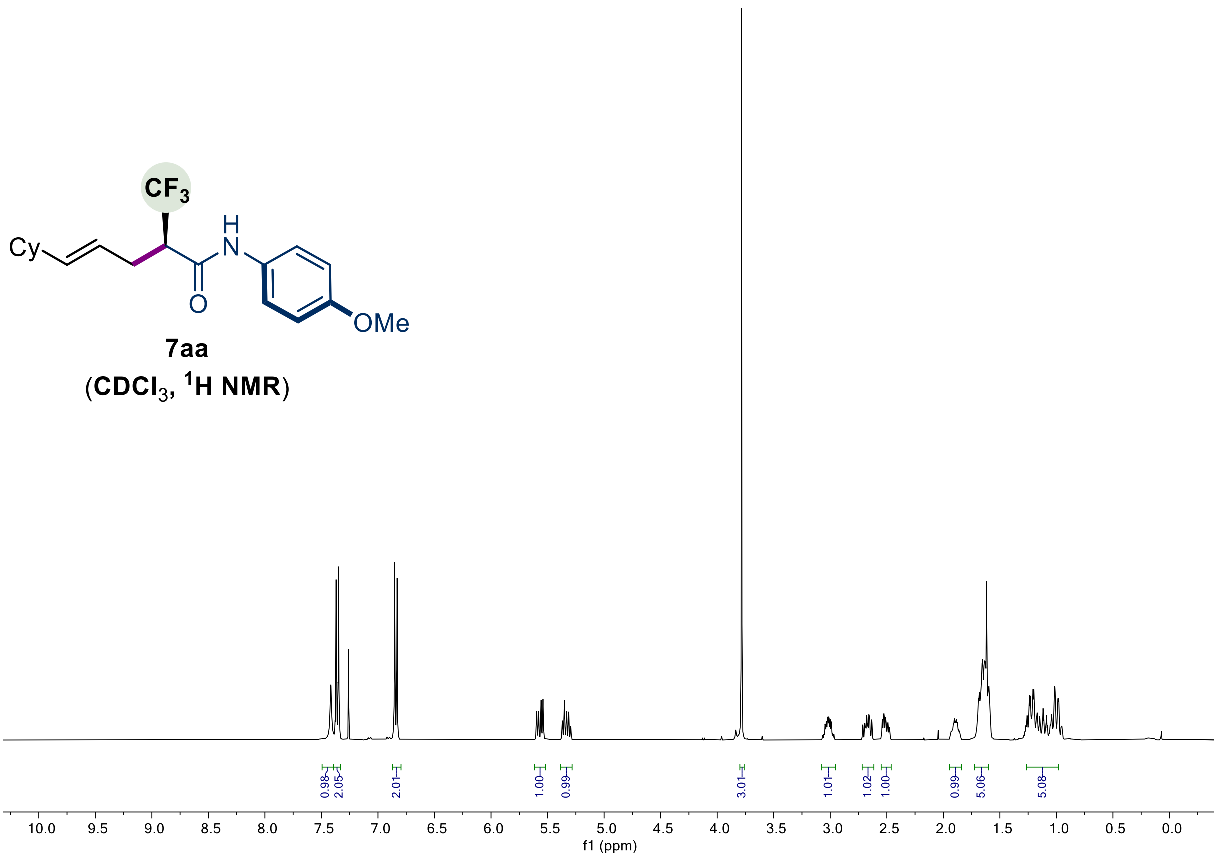

7aa
(CDCl$_3$, ^{1}H NMR)

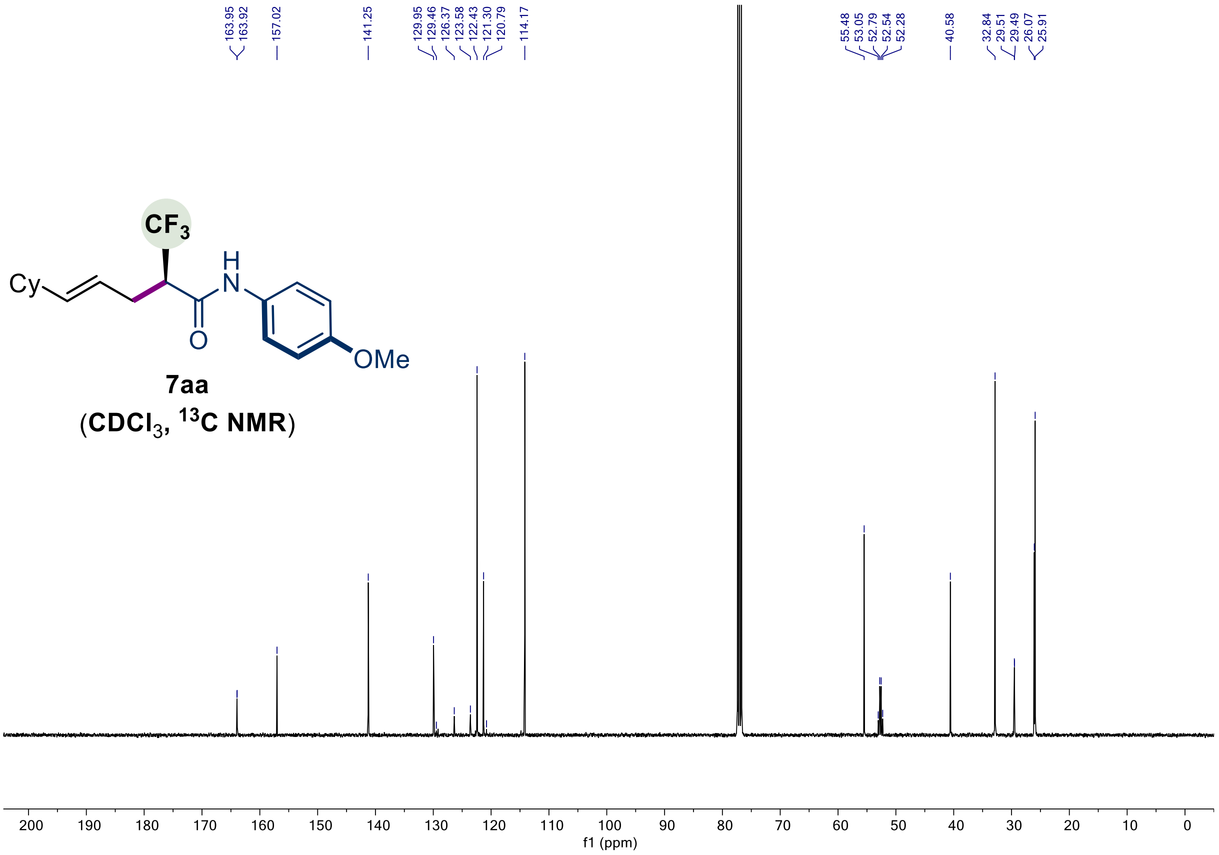

7aa
(CDCl$_3$, ^{13}C NMR)

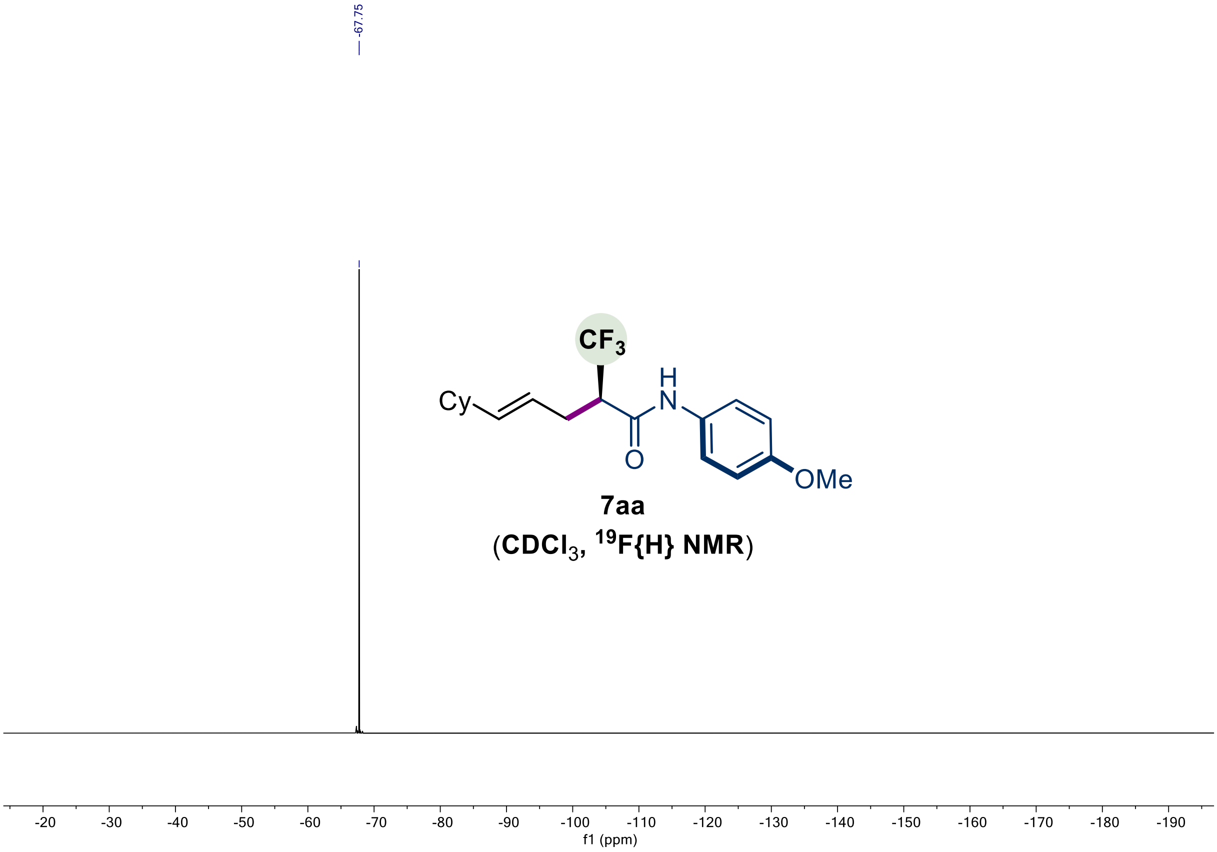
-67.75
CF3
H
N
Cy
O
OMe
7aa
(CDCl3, 19F{H} NMR)
-20
-30
-40
-50
-60
-70
-80
-90
-100
-110
-120
-130
-140
-150
-160
-170
-180
-190
f1 (ppm)

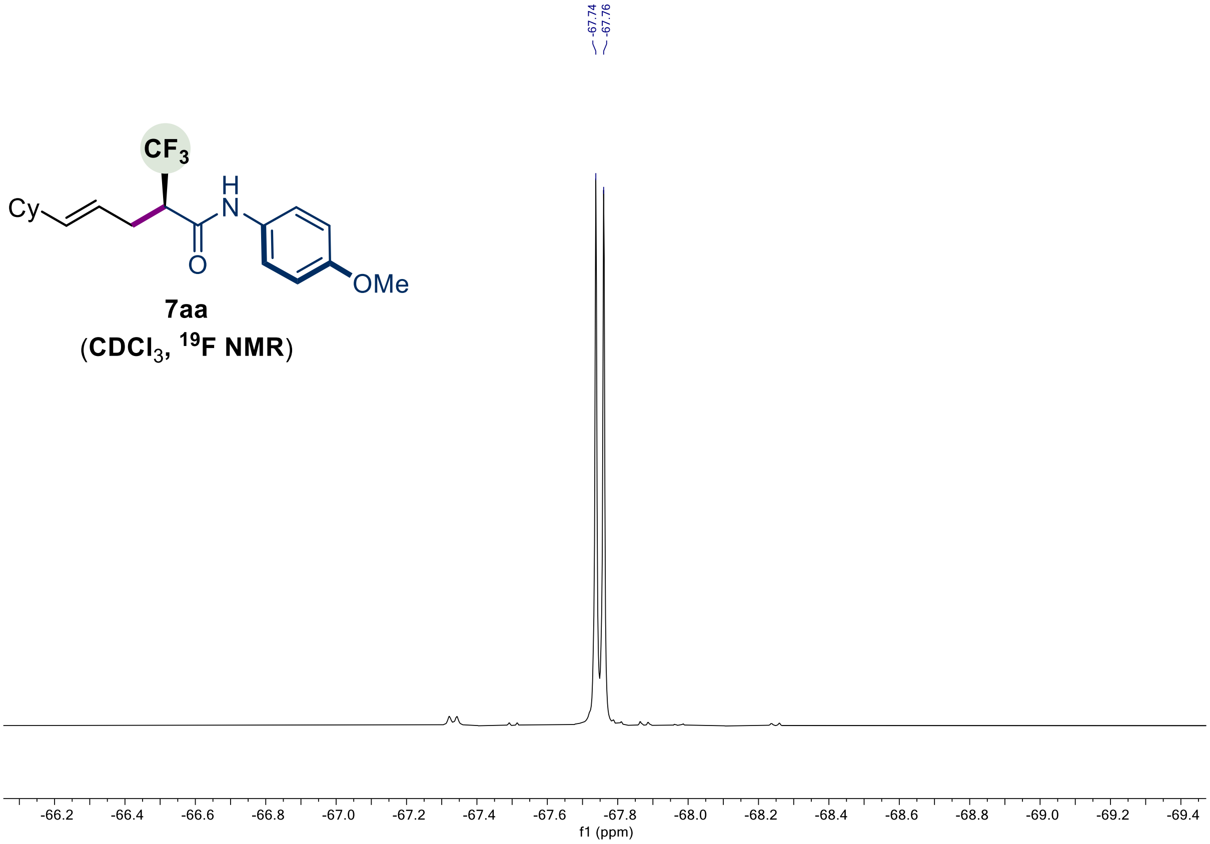
-67.74
-67.76
CF3
H
N
Cy
O
OMe
7aa
(CDCl3, 19F NMR)
-66.2
-66.4
-66.6
-66.8
-67.0
-67.2
-67.4
-67.6
-67.8
-68.0
-68.2
-68.4
-68.6
-68.8
-69.0
-69.2
-69.4
f1 (ppm)

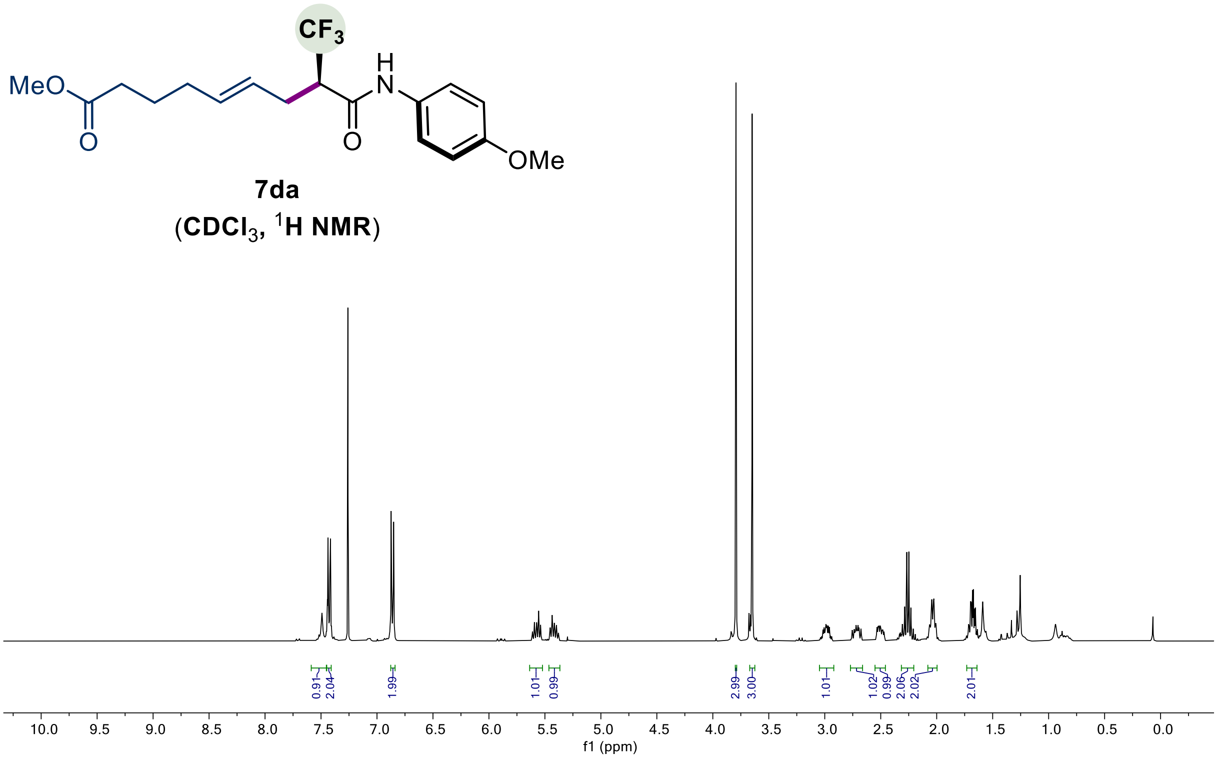

CF3
MeO
O
H
N
O
OMe
7da
(CDCl3, 1H NMR)
0.91
2.04
1.99
1.01
0.99
2.99
3.00
1.01
1.02
0.99
2.06
2.02
2.01
10.0 9.5 9.0 8.5 8.0 7.5 7.0 6.5 6.0 5.5 5.0 4.5 4.0 3.5 3.0 2.5 2.0 1.5 1.0 0.5 0.0
f1 (ppm)

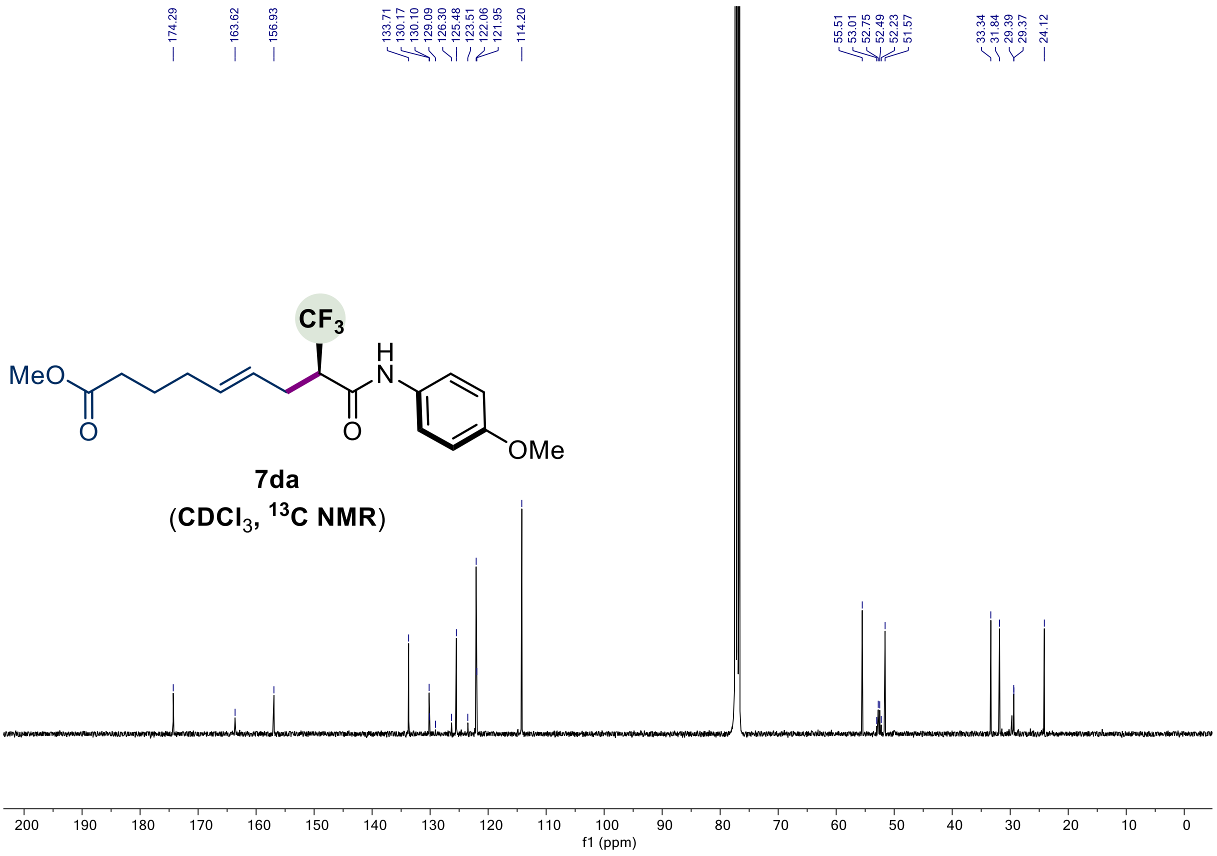

174.29
163.62
156.93
133.71
130.17
130.10
129.09
126.30
125.48
123.51
122.06
121.95
114.20
55.51
53.01
52.75
52.49
52.23
51.57
33.34
31.84
29.39
29.37
24.12
CF3
MeO
O
H
N
O
OMe
7da
(CDCl3, 13C NMR)
200 190 180 170 160 150 140 130 120 110 100 90 80 70 60 50 40 30 20 10 0
f1 (ppm)

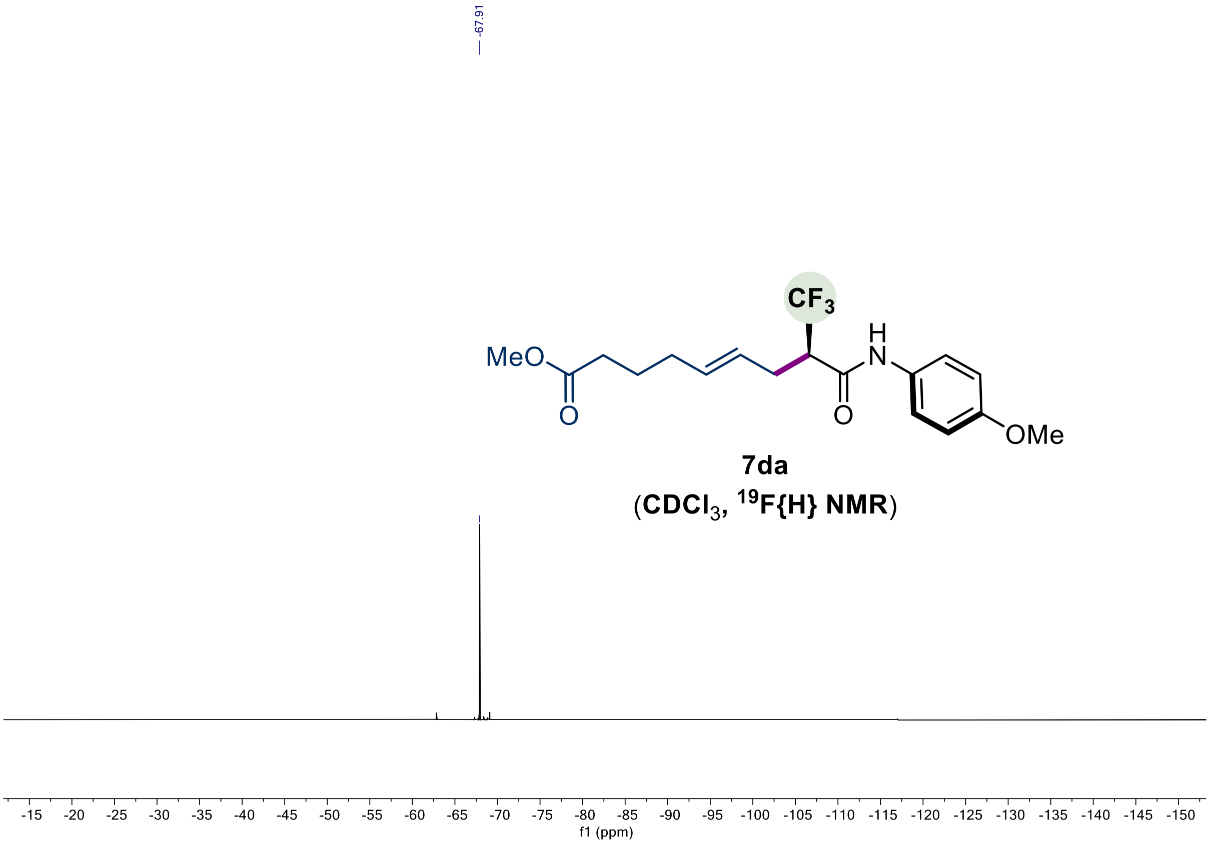
-67.91
MeO
O
CF3
H
N
O
OMe
7da
(CDCl3, 19F{H} NMR)
-15 -20 -25 -30 -35 -40 -45 -50 -55 -60 -65 -70 -75 -80 -85 -90 -95 -100 -105 -110 -115 -120 -125 -130 -135 -140 -145 -150
f1 (ppm)

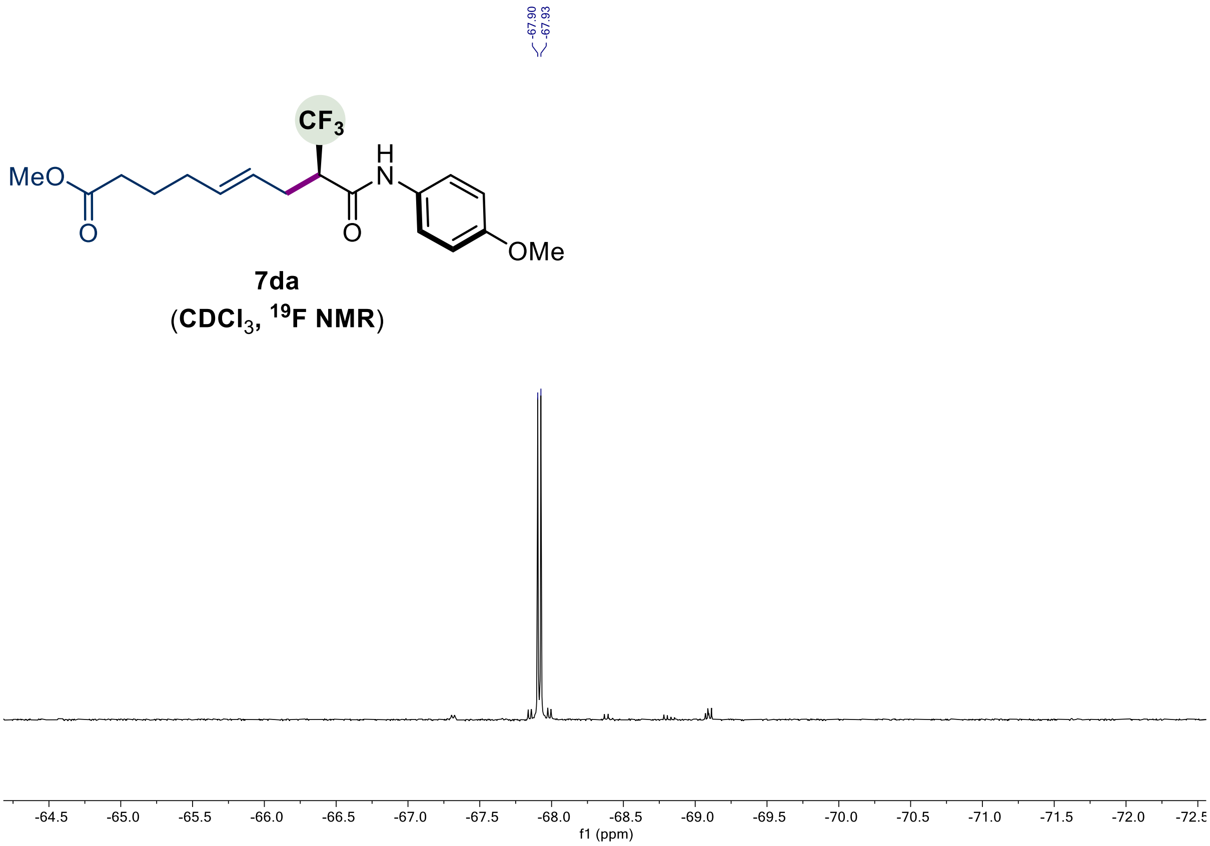
-67.90
-67.93
MeO
O
CF3
H
N
O
OMe
7da
(CDCl3, 19F NMR)
-64.5 -65.0 -65.5 -66.0 -66.5 -67.0 -67.5 -68.0 -68.5 -69.0 -69.5 -70.0 -70.5 -71.0 -71.5 -72.0 -72.5
f1 (ppm)

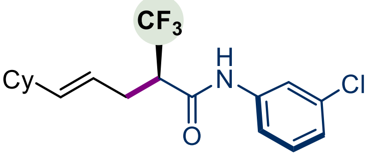

7ab
(**CDCl$_3$**, **^{1}H NMR**)

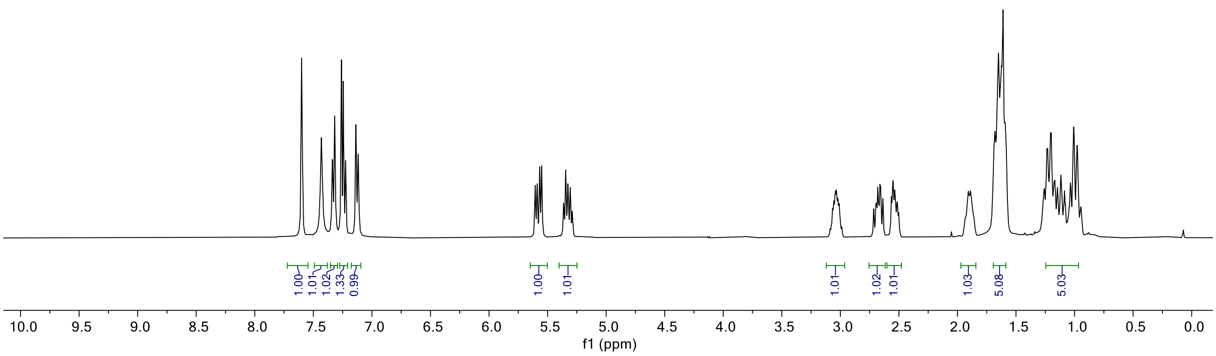

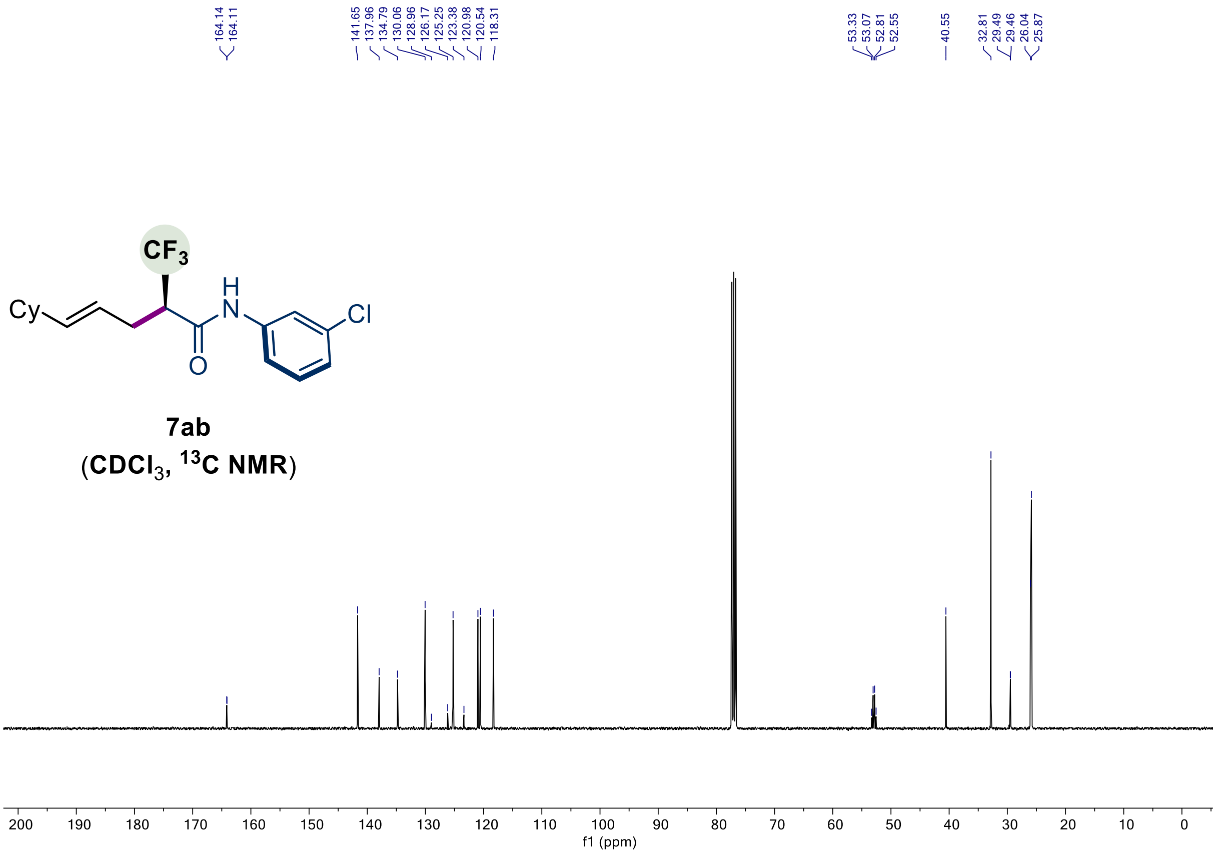

7ab
(**CDCl$_3$**, **^{13}C NMR**)

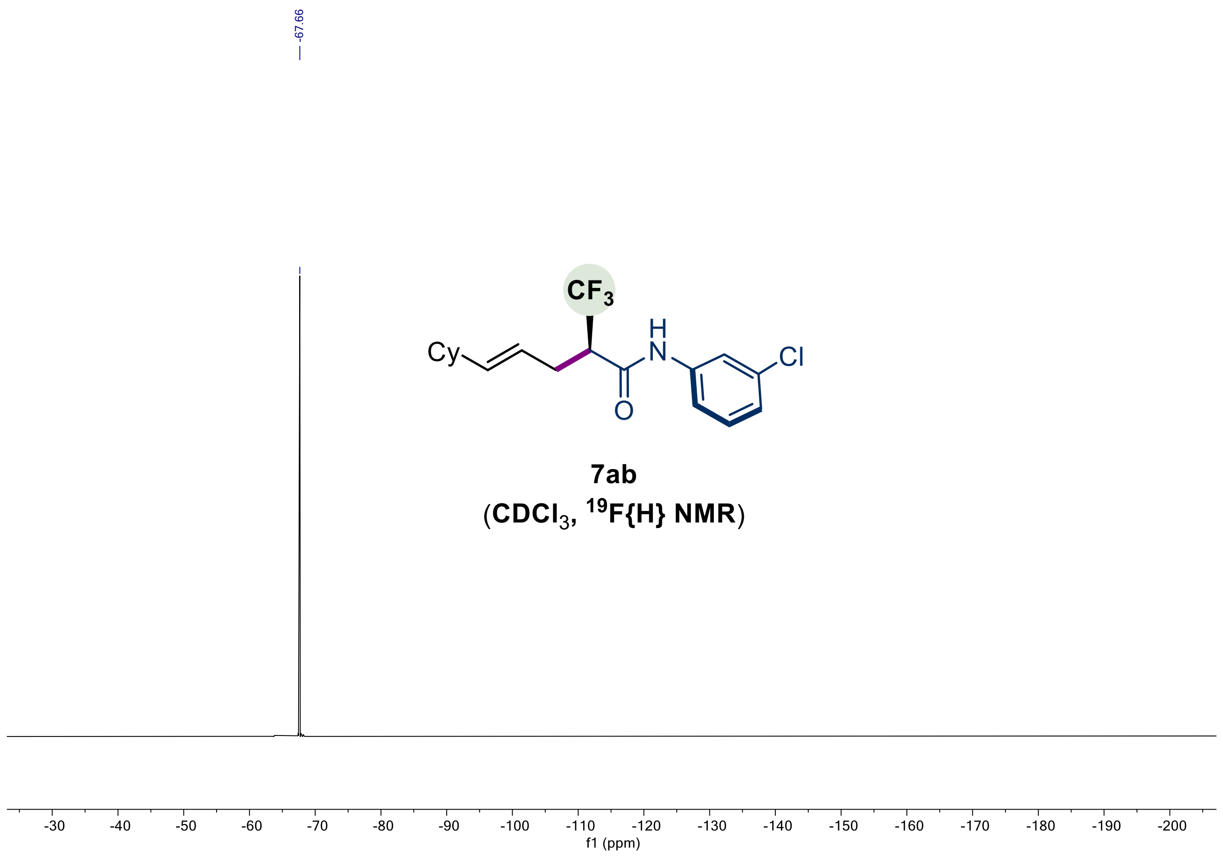
-67.66
Cy
CF3
H
N
Cl
O
7ab
(CDCl3, 19F{H} NMR)
-30 -40 -50 -60 -70 -80 -90 -100 -110 -120 -130 -140 -150 -160 -170 -180 -190 -200
f1 (ppm)

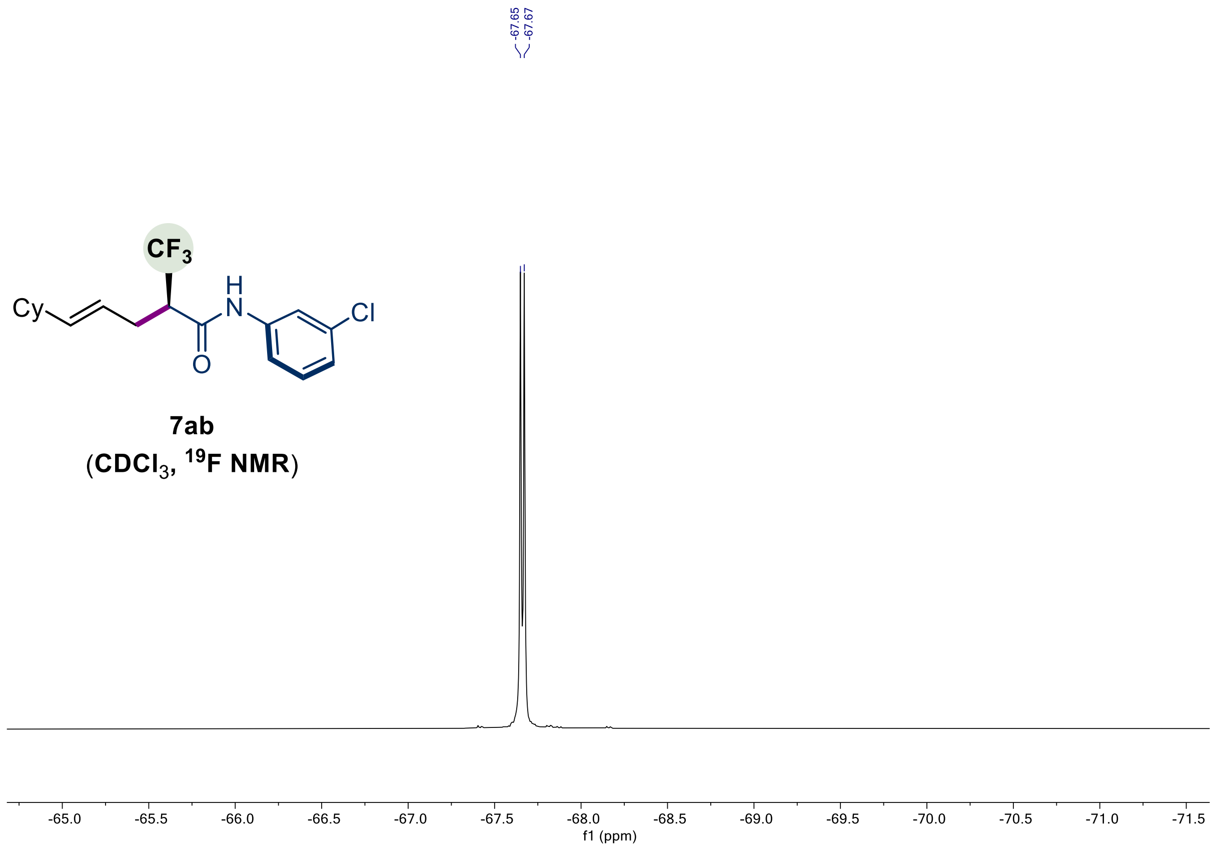
-67.65
-67.67
Cy
CF3
H
N
Cl
O
7ab
(CDCl3, 19F NMR)
-65.0 -65.5 -66.0 -66.5 -67.0 -67.5 -68.0 -68.5 -69.0 -69.5 -70.0 -70.5 -71.0 -71.5
f1 (ppm)

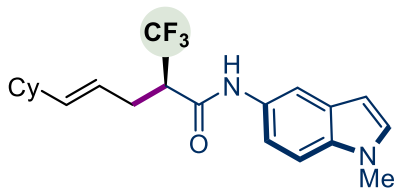

7ac
(CDCl$_3$, ^{1}H NMR)

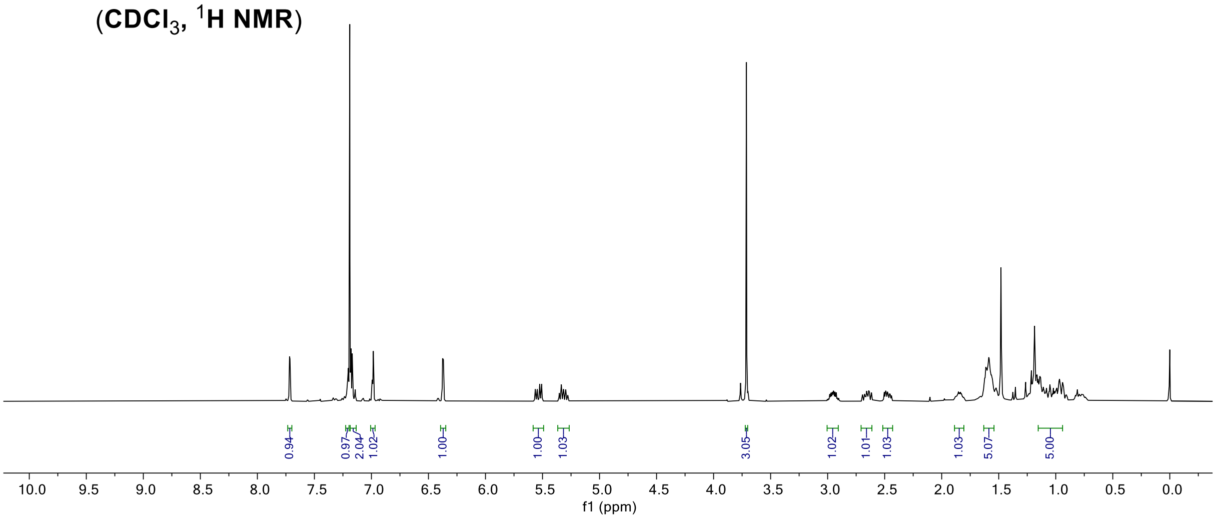

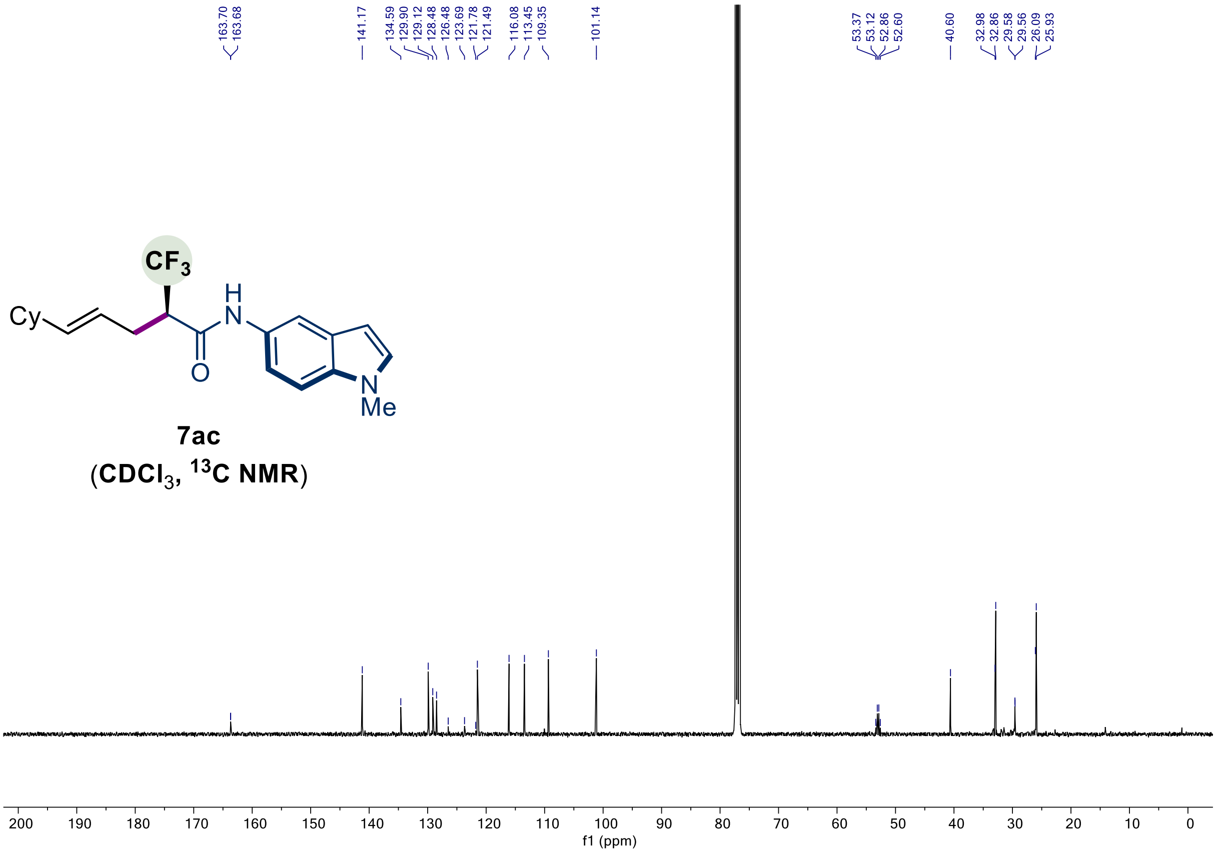

7ac
(CDCl$_3$, ^{13}C NMR)

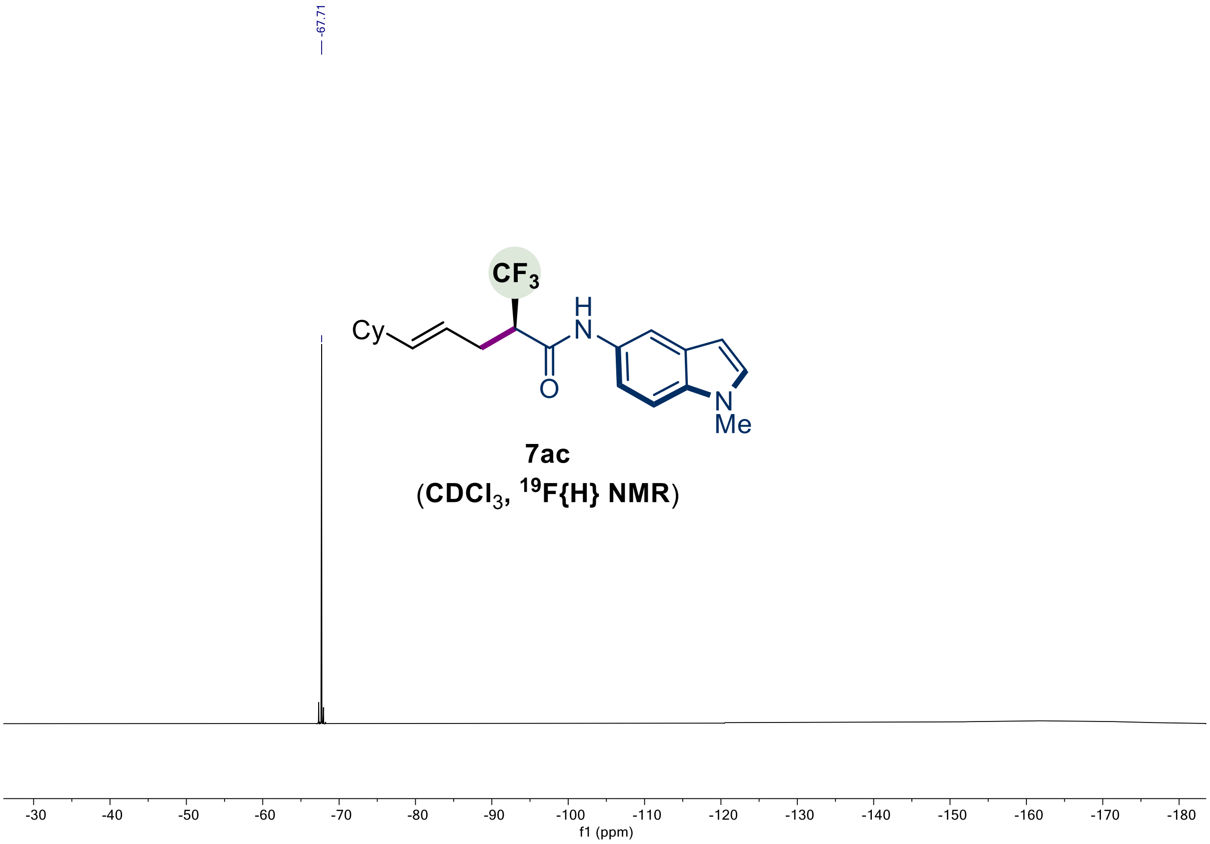
-67.71
CF3
Cy
H
N
O
N
Me
7ac
(CDCl3, 19F{H} NMR)
-30 -40 -50 -60 -70 -80 -90 -100 -110 -120 -130 -140 -150 -160 -170 -180
f1 (ppm)

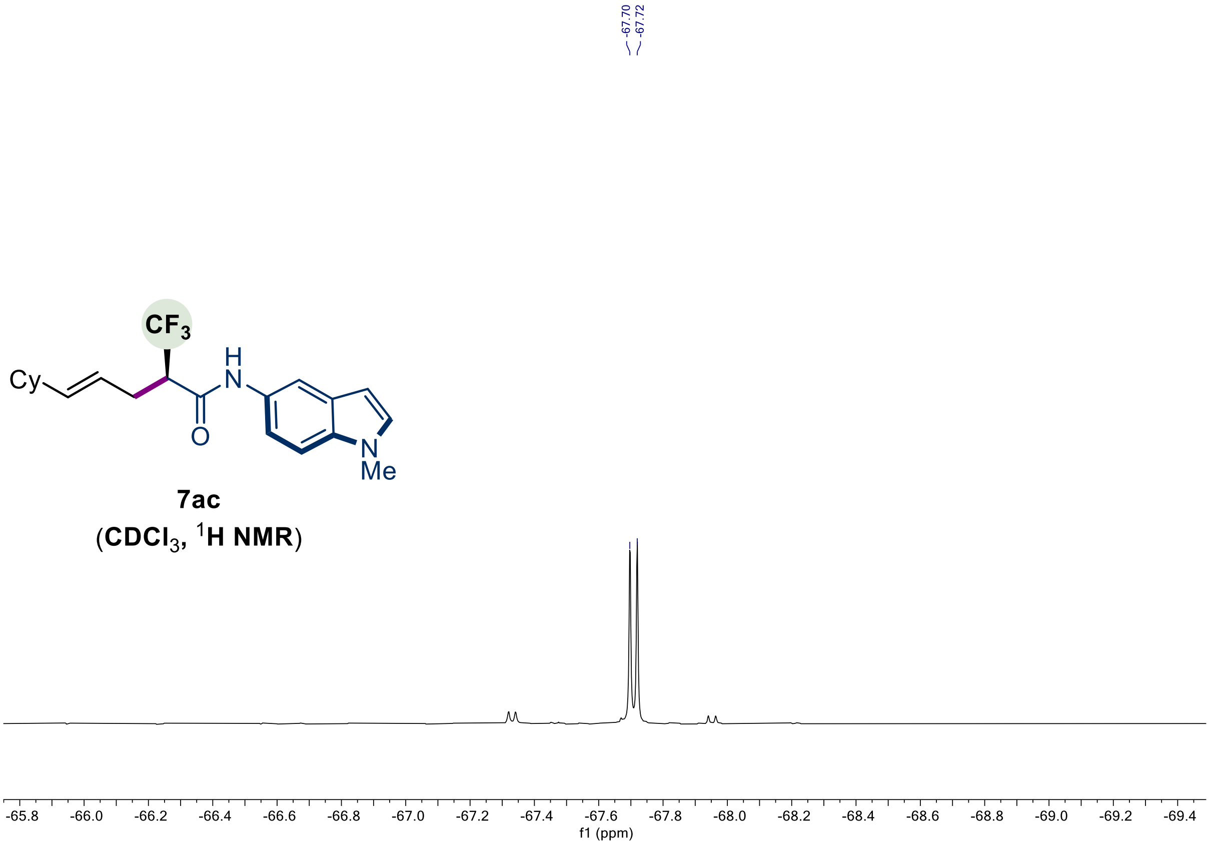
-67.70
-67.72
CF3
Cy
H
N
O
N
Me
7ac
(CDCl3, 1H NMR)
-65.8 -66.0 -66.2 -66.4 -66.6 -66.8 -67.0 -67.2 -67.4 -67.6 -67.8 -68.0 -68.2 -68.4 -68.6 -68.8 -69.0 -69.2 -69.4
f1 (ppm)

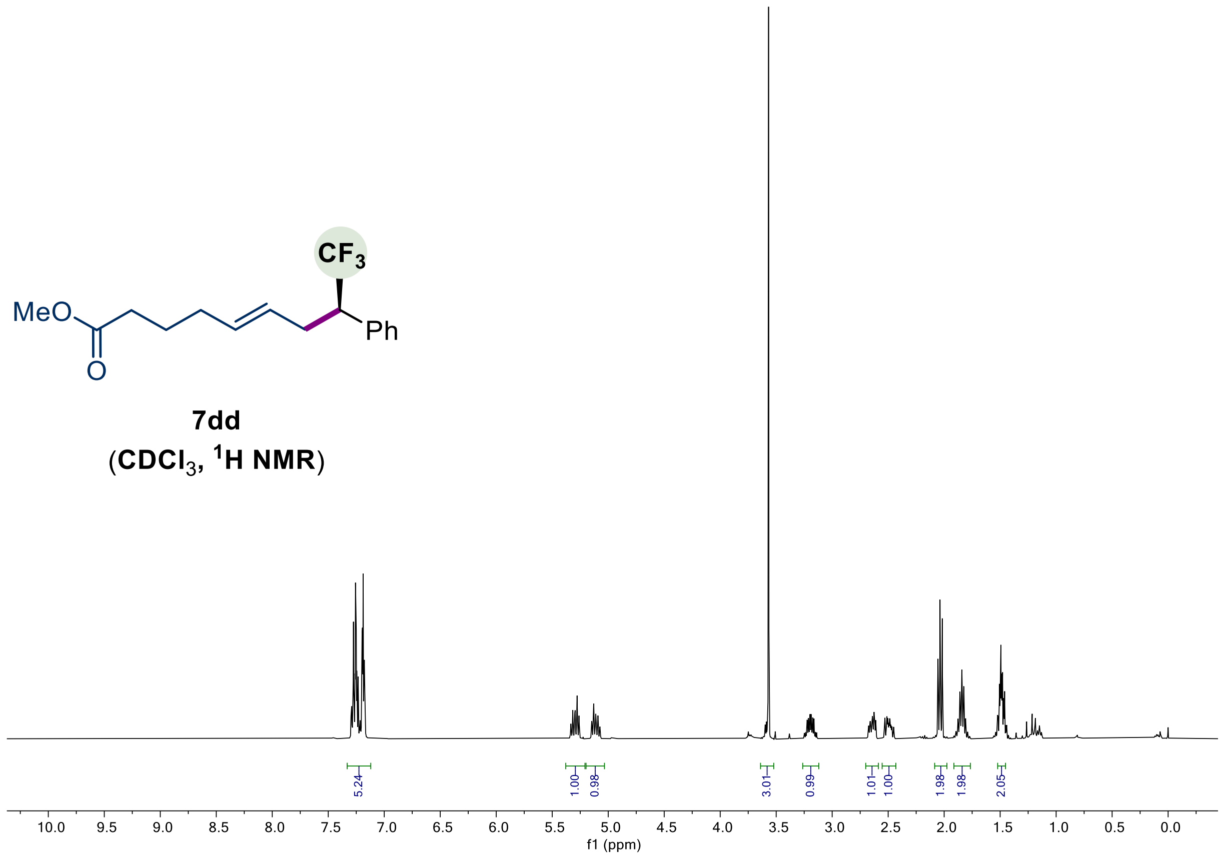
CF3
MeO
O
Ph
7dd
(CDCl3, 1H NMR)
5.24
1.00
0.98
3.01
0.99
1.01
1.00
1.98
1.98
2.05
10.0 9.5 9.0 8.5 8.0 7.5 7.0 6.5 6.0 5.5 5.0 4.5 4.0 3.5 3.0 2.5 2.0 1.5 1.0 0.5 0.0
f1 (ppm)

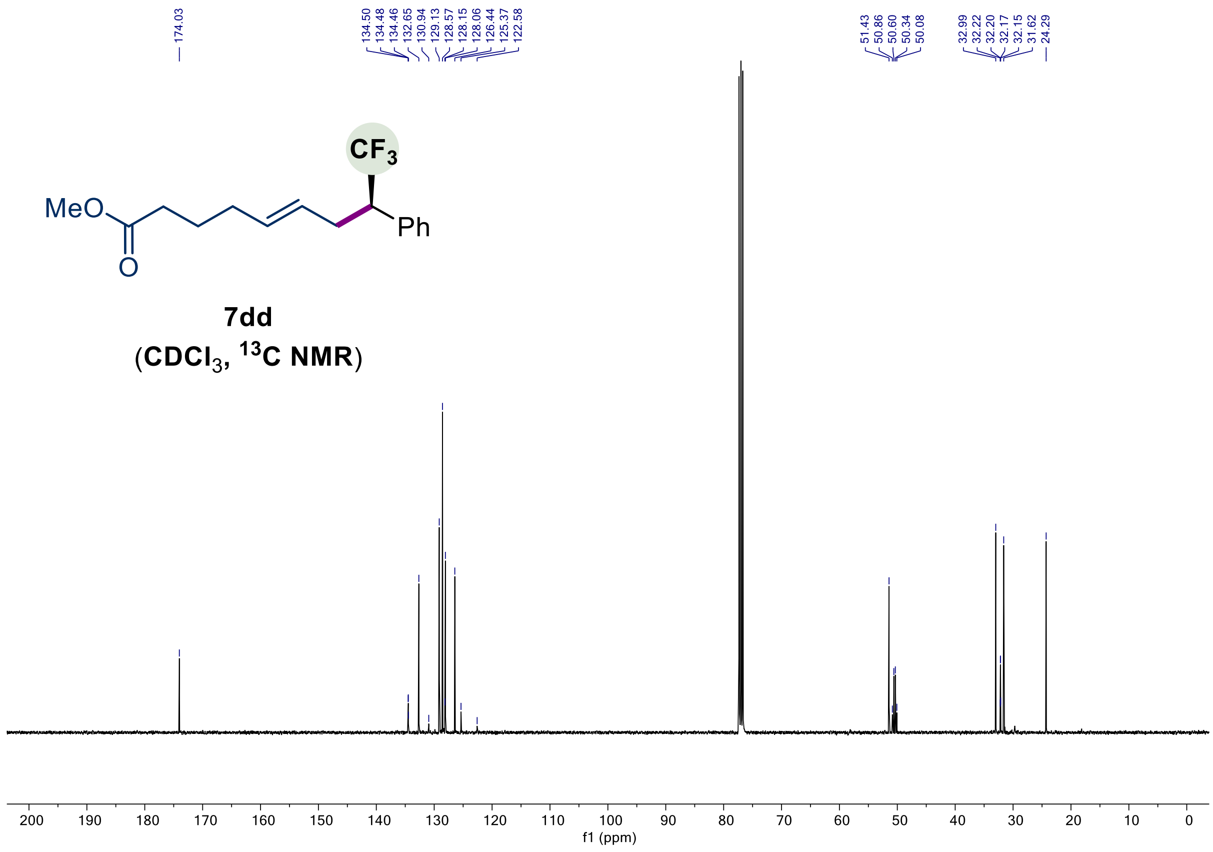
174.03
134.50
134.48
134.46
132.65
130.94
129.13
128.57
128.15
128.06
126.44
125.37
122.58
51.43
50.86
50.60
50.34
50.08
32.99
32.22
32.20
32.17
32.15
31.62
24.29
CF3
MeO
O
Ph
7dd
(CDCl3, 13C NMR)
200 190 180 170 160 150 140 130 120 110 100 90 80 70 60 50 40 30 20 10 0
f1 (ppm)

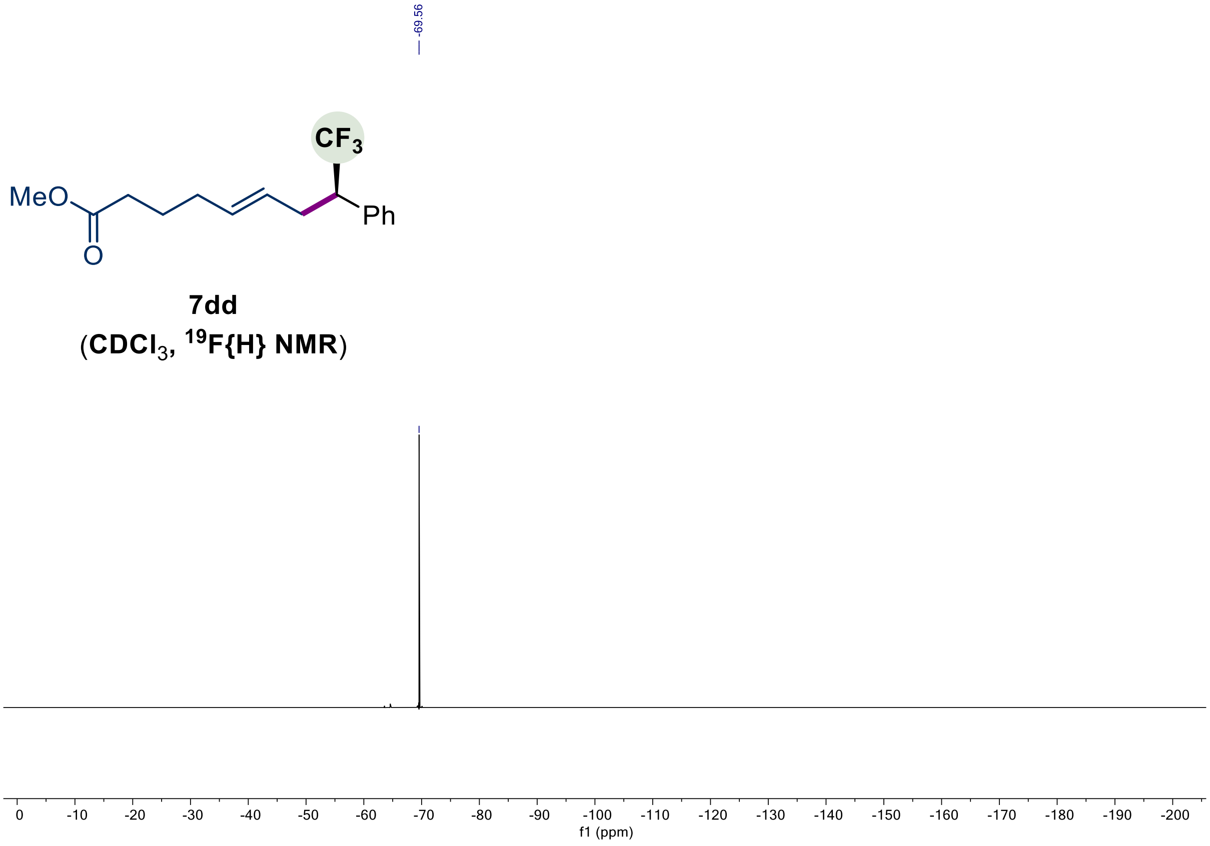

7dd
(**$CDCl_3$, ^{19}F{H} NMR**)

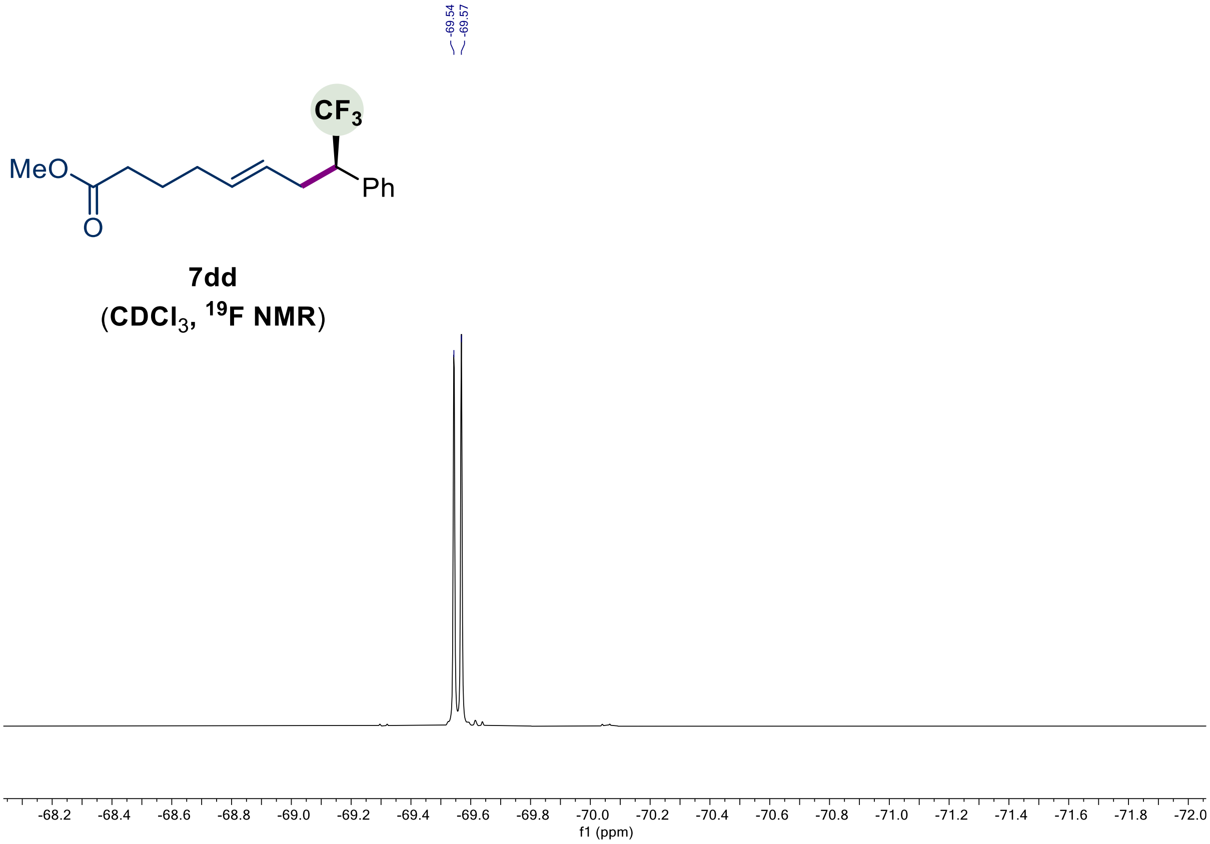

7dd
(**$CDCl_3$, ^{19}F NMR**)

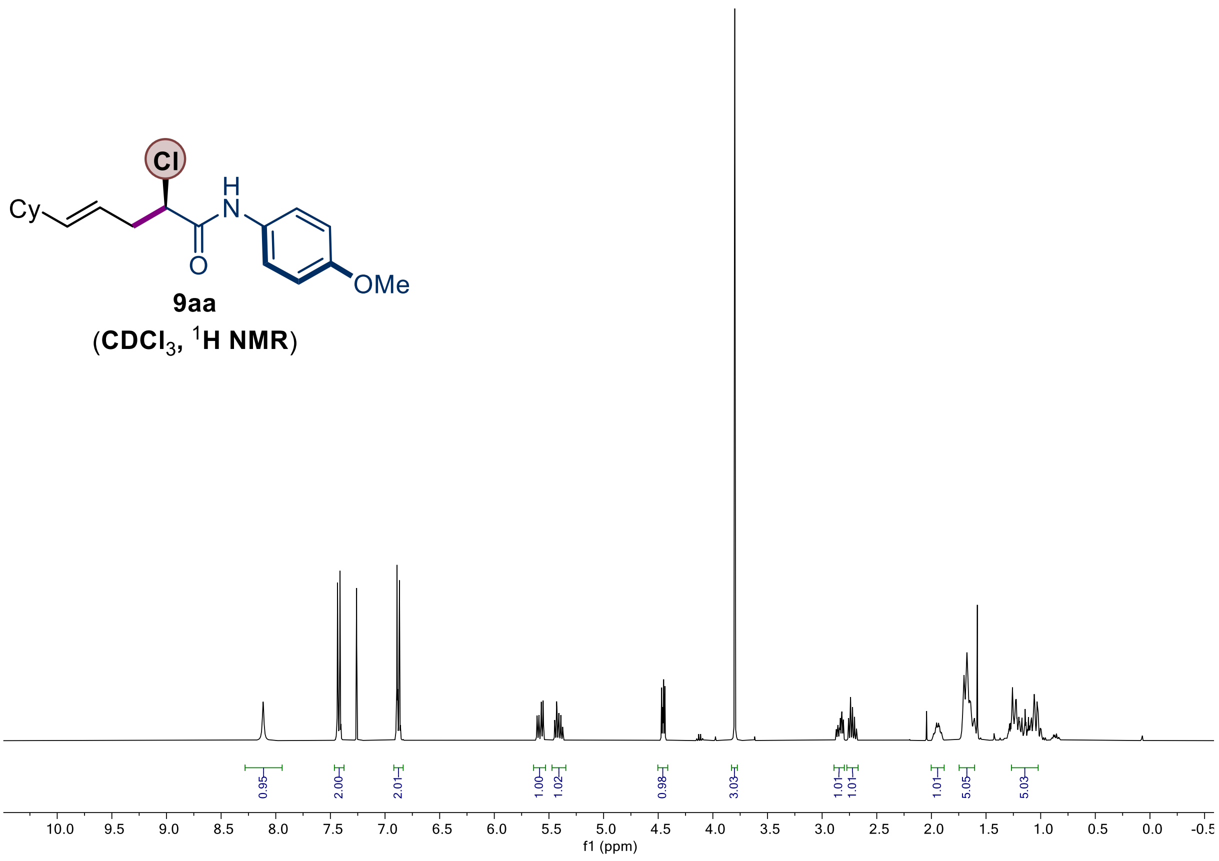
Cl
Cy
H
N
O
OMe
9aa
(CDCl$_3$, ^{1}H NMR)
0.95
2.00
2.01
1.00
1.02
0.98
3.03
1.01
1.01
1.01
5.05
5.03
10.0 9.5 9.0 8.5 8.0 7.5 7.0 6.5 6.0 5.5 5.0 4.5 4.0 3.5 3.0 2.5 2.0 1.5 1.0 0.5 0.0 -0.5
f1 (ppm)

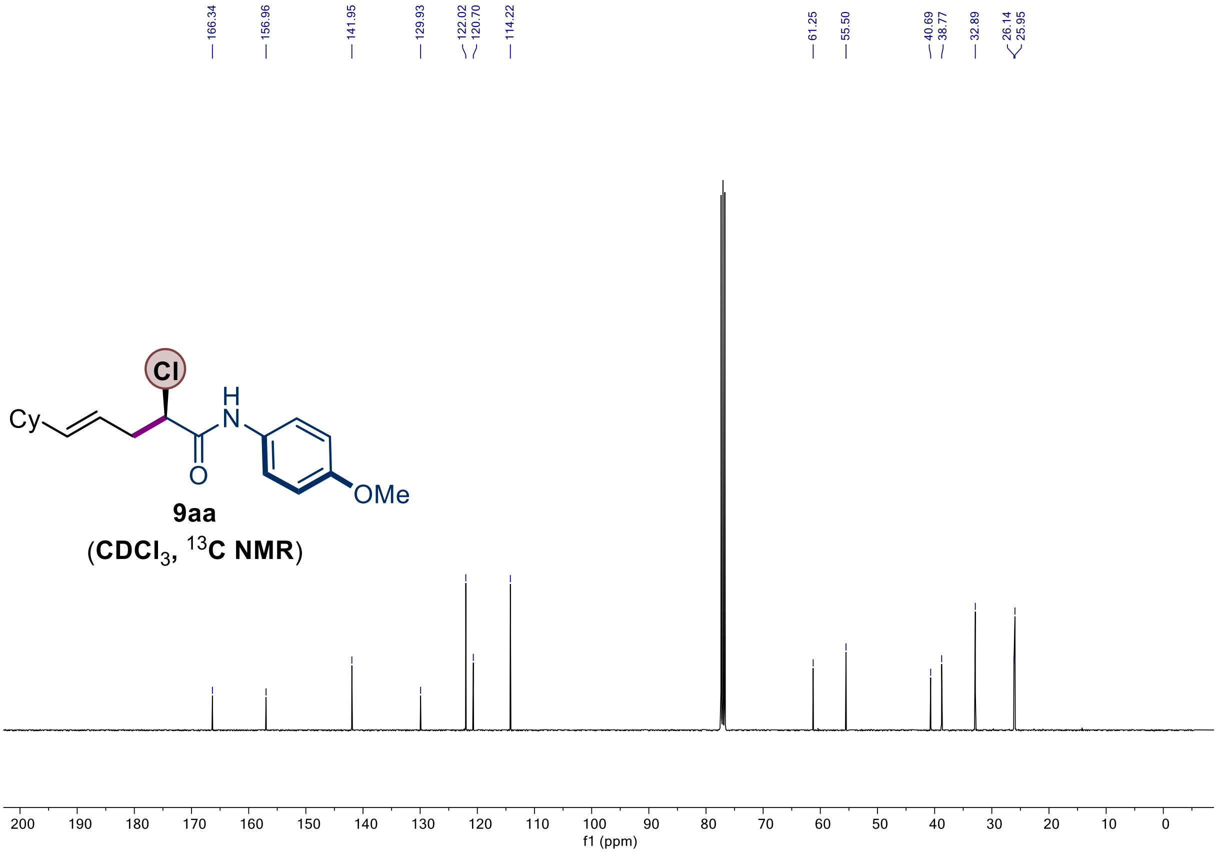
166.34
156.96
141.95
129.93
122.02
120.70
114.22
61.25
55.50
40.69
38.77
32.89
26.14
25.95
Cl
Cy
H
N
O
OMe
9aa
(CDCl$_3$, ^{13}C NMR)
200 190 180 170 160 150 140 130 120 110 100 90 80 70 60 50 40 30 20 10 0
f1 (ppm)

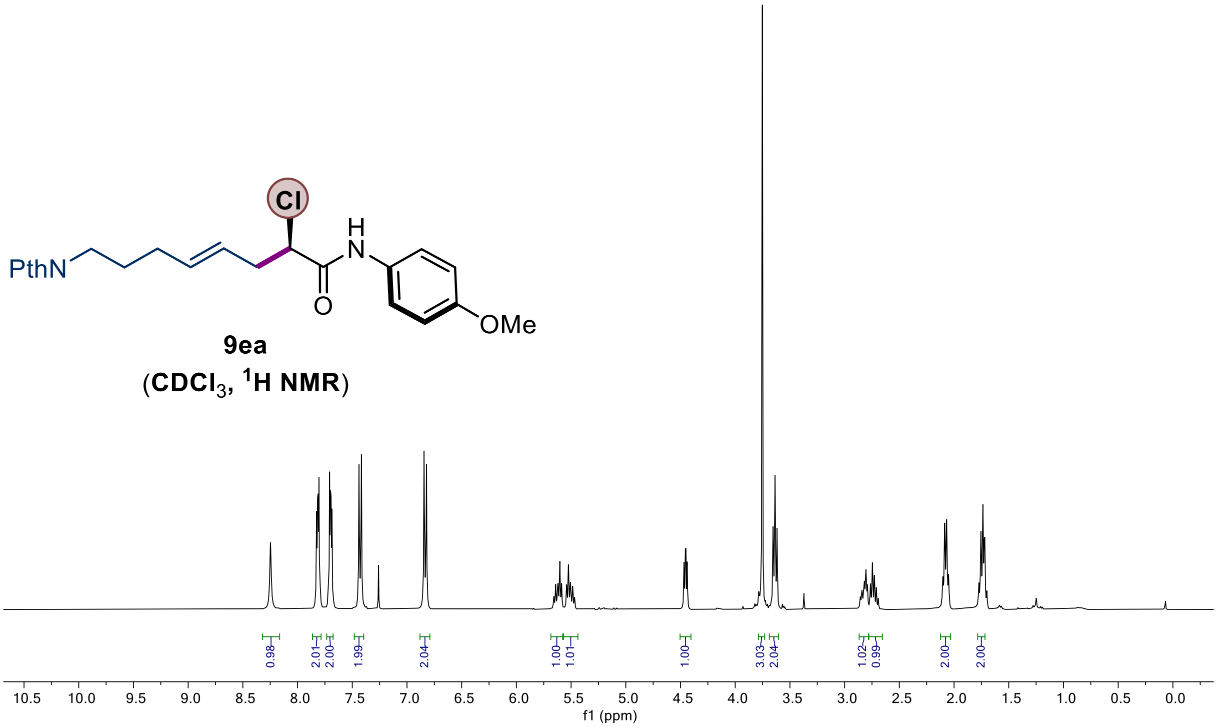

PthN
Cl
H
N
O
OMe
9ea
(CDCl$_3$, ^{1}H NMR)
0.98
2.01
2.00
1.99
2.04
1.00
1.01
1.00
3.03
2.04
1.02
0.99
2.00
2.00
10.5 10.0 9.5 9.0 8.5 8.0 7.5 7.0 6.5 6.0 5.5 5.0 4.5 4.0 3.5 3.0 2.5 2.0 1.5 1.0 0.5 0.0
f1 (ppm)

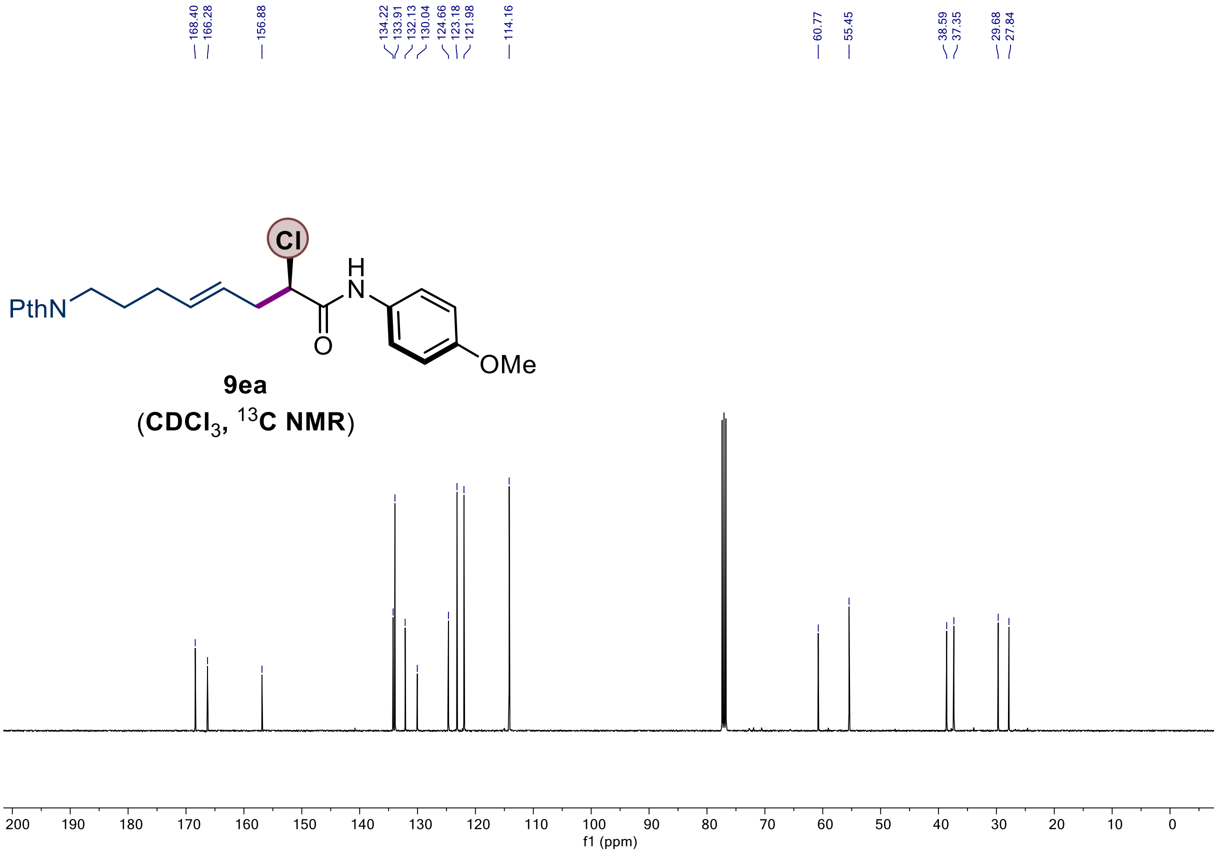

168.40
166.28
156.88
134.22
133.91
132.13
130.04
124.66
123.18
121.98
114.16
60.77
55.45
38.59
37.35
29.68
27.84
PthN
Cl
H
N
O
OMe
9ea
(CDCl$_3$, ^{13}C NMR)
200 190 180 170 160 150 140 130 120 110 100 90 80 70 60 50 40 30 20 10 0
f1 (ppm)

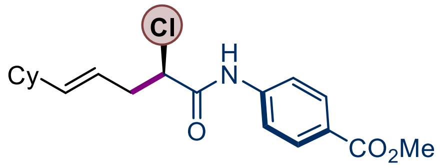

9ab
($CDCl_3$, ^{1}H NMR)

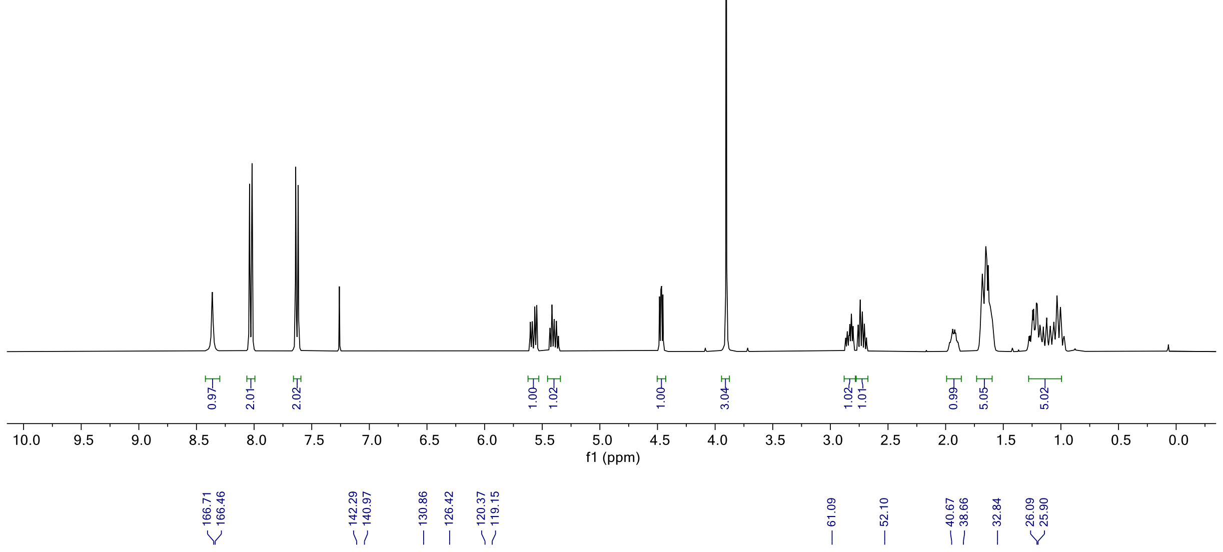

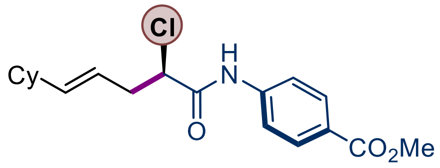

9ab
($CDCl_3$, ^{13}C NMR)

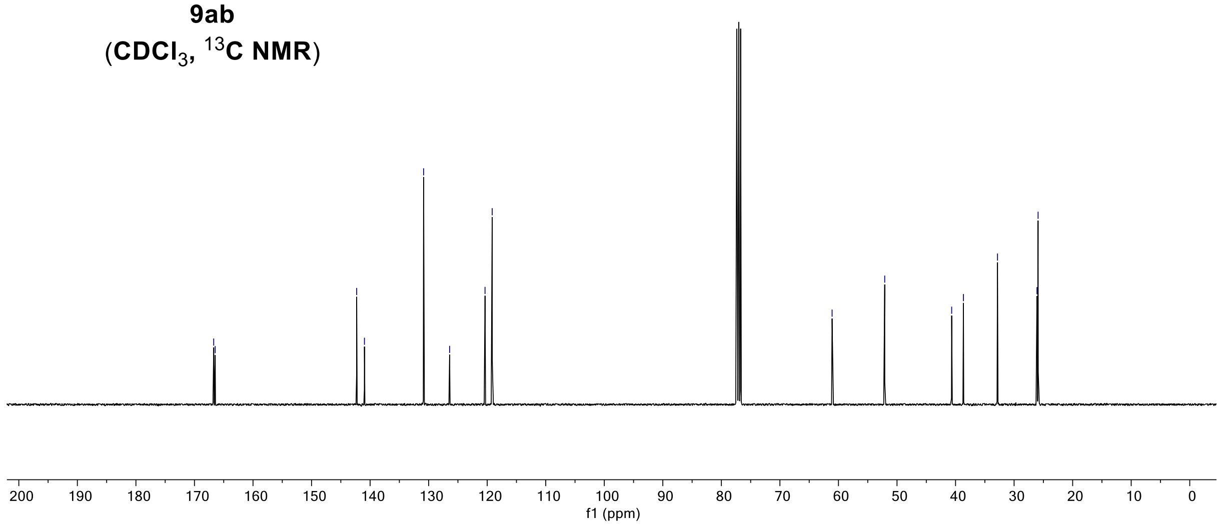

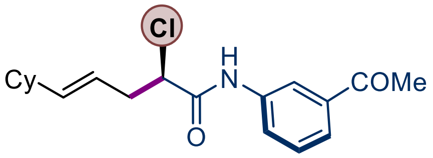

9ac
($CDCl_3$, ^1H NMR)

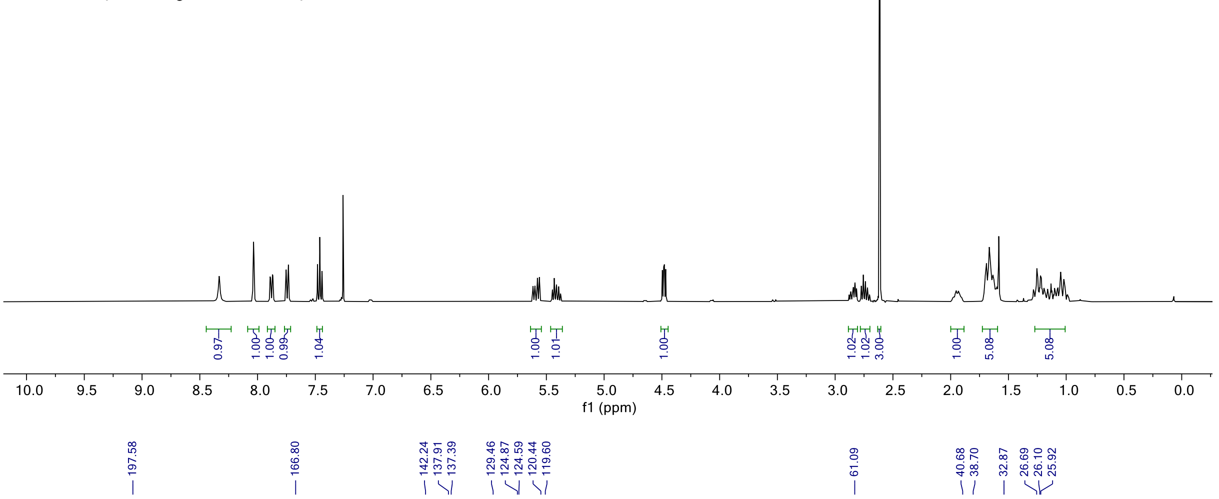

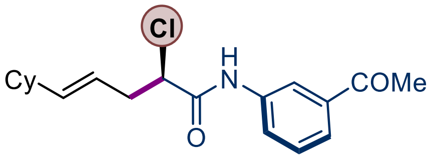

9ac
($CDCl_3$, ^{13}C NMR)

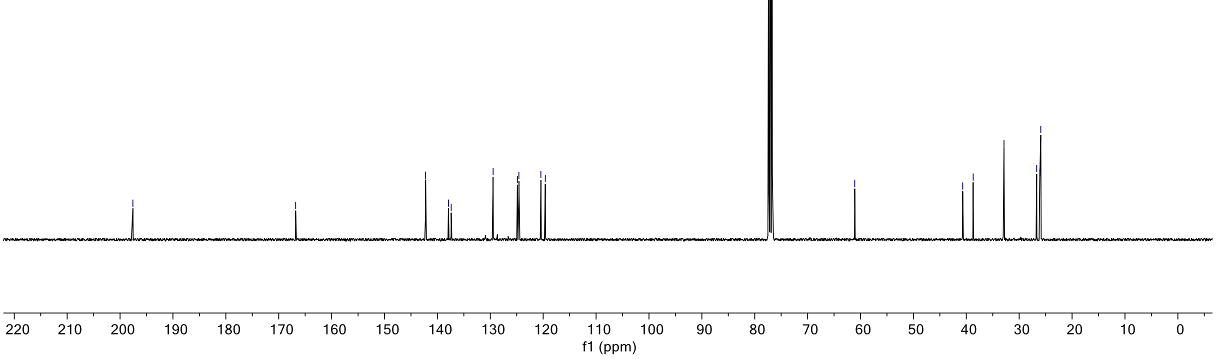

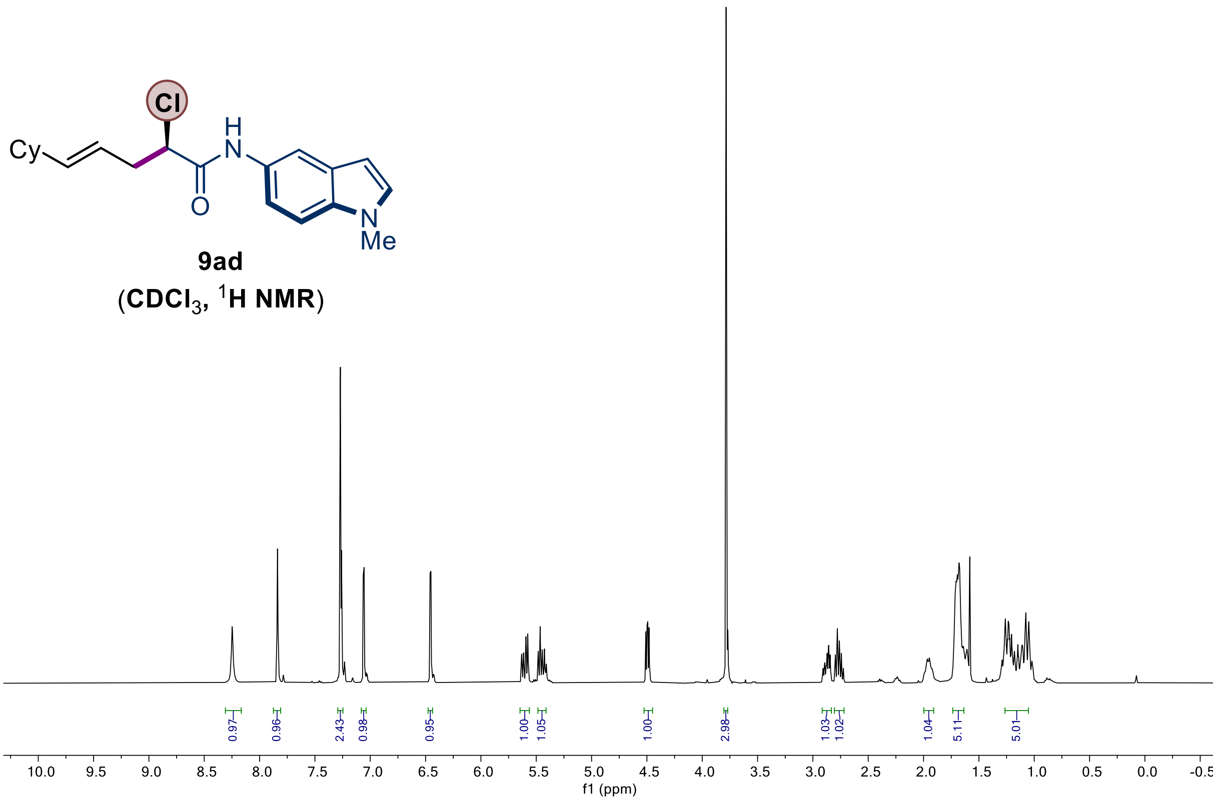
Cl
H
N
Cy
O
N
Me
9ad
(CDCl3, 1H NMR)
0.97
0.96
2.43
0.98
0.95
1.00
1.05
1.00
2.98
1.03
1.02
1.04
5.11
5.01
10.0 9.5 9.0 8.5 8.0 7.5 7.0 6.5 6.0 5.5 5.0 4.5 4.0 3.5 3.0 2.5 2.0 1.5 1.0 0.5 0.0 -0.5
f1 (ppm)

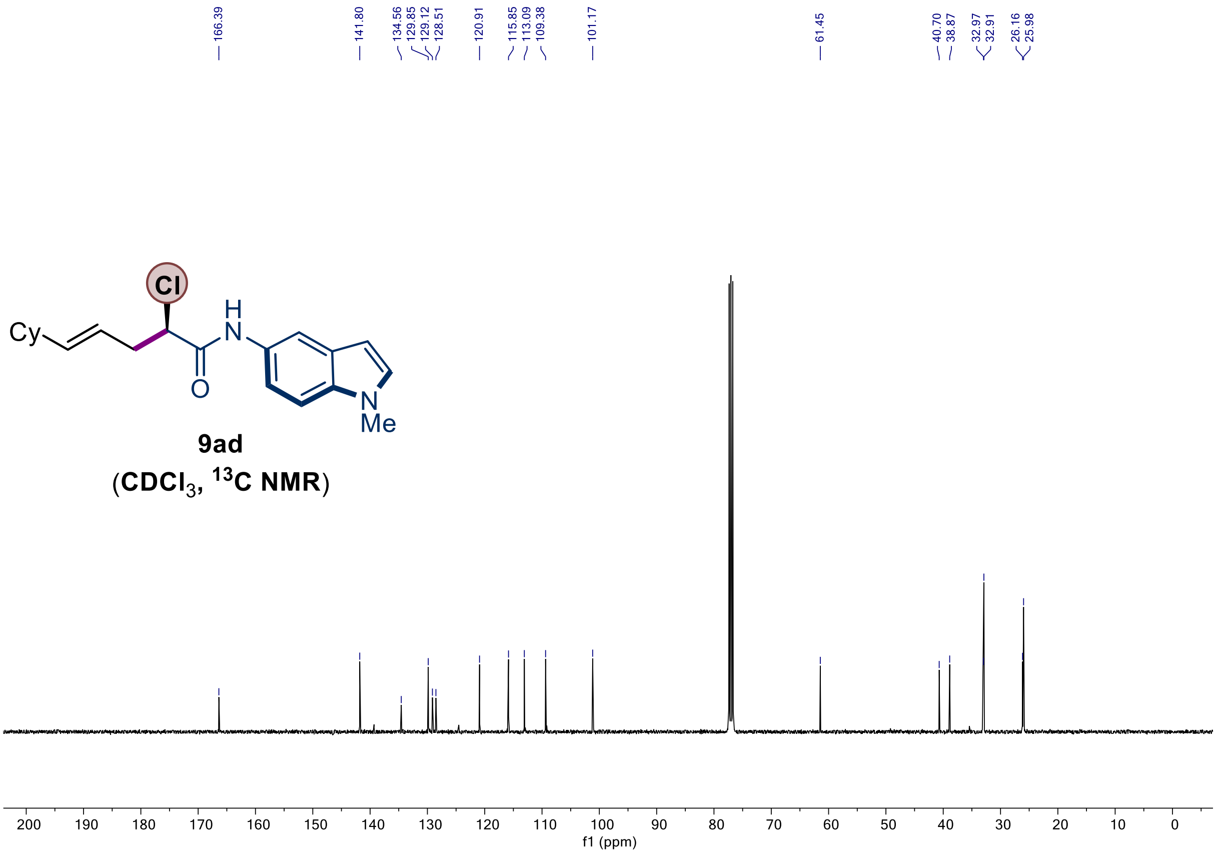
166.39
141.80
134.56
129.85
129.12
128.51
120.91
115.85
113.09
109.38
101.17
61.45
40.70
38.87
32.97
32.91
26.16
25.98
Cl
H
N
Cy
O
N
Me
9ad
(CDCl3, 13C NMR)
200 190 180 170 160 150 140 130 120 110 100 90 80 70 60 50 40 30 20 10 0
f1 (ppm)

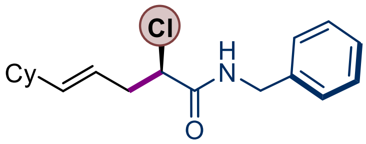

9ae
(CDCl$_3$, ^{1}H NMR)

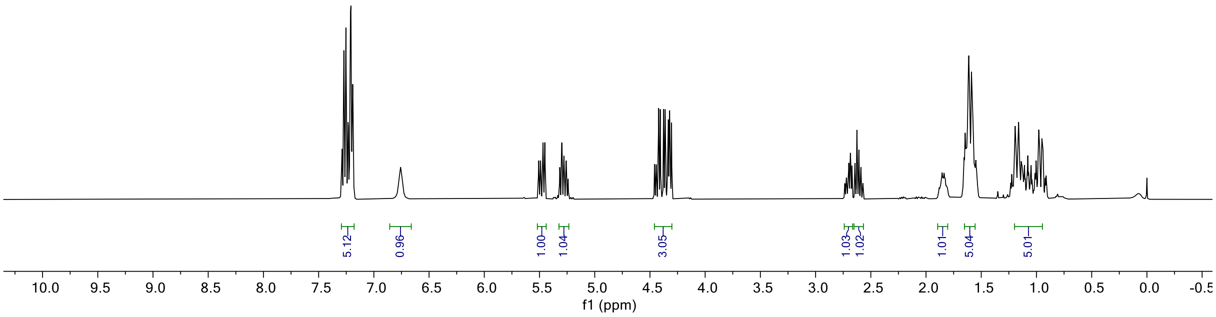

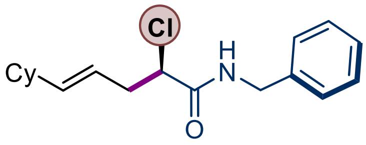

9ae
(CDCl$_3$, ^{13}C NMR)

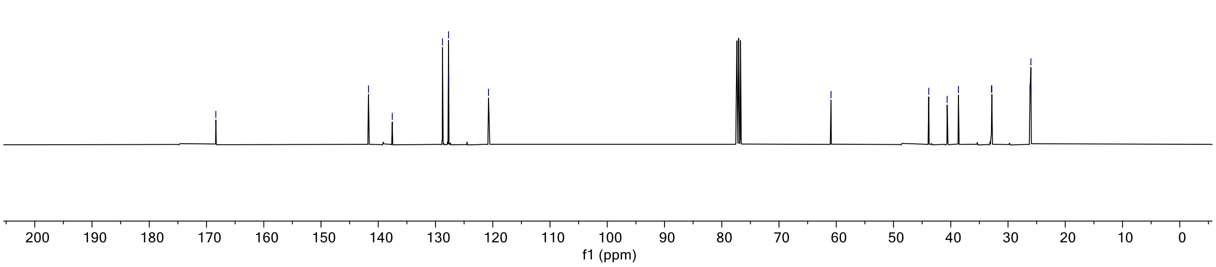

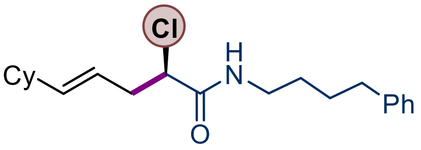

9af
($CDCl_3$, ^{1}H NMR)

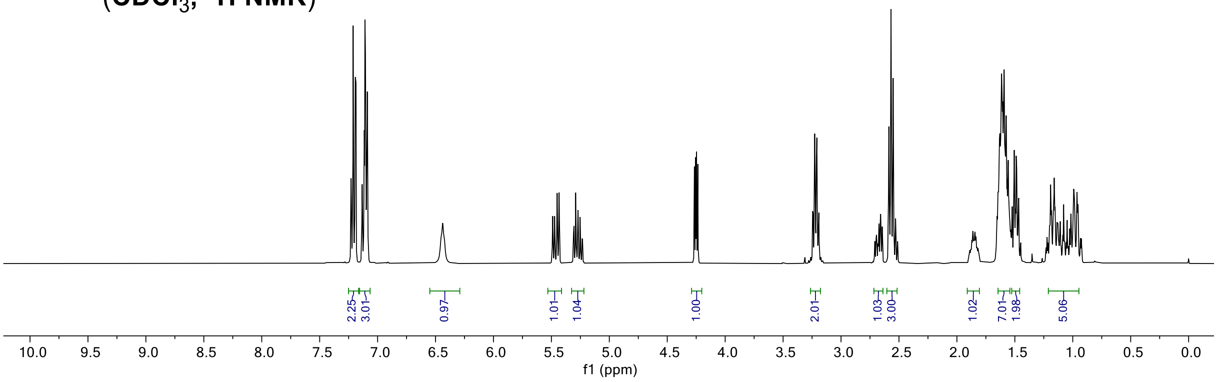

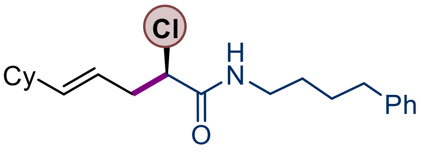

9af
(CD_2Cl_2, ^{13}C NMR)

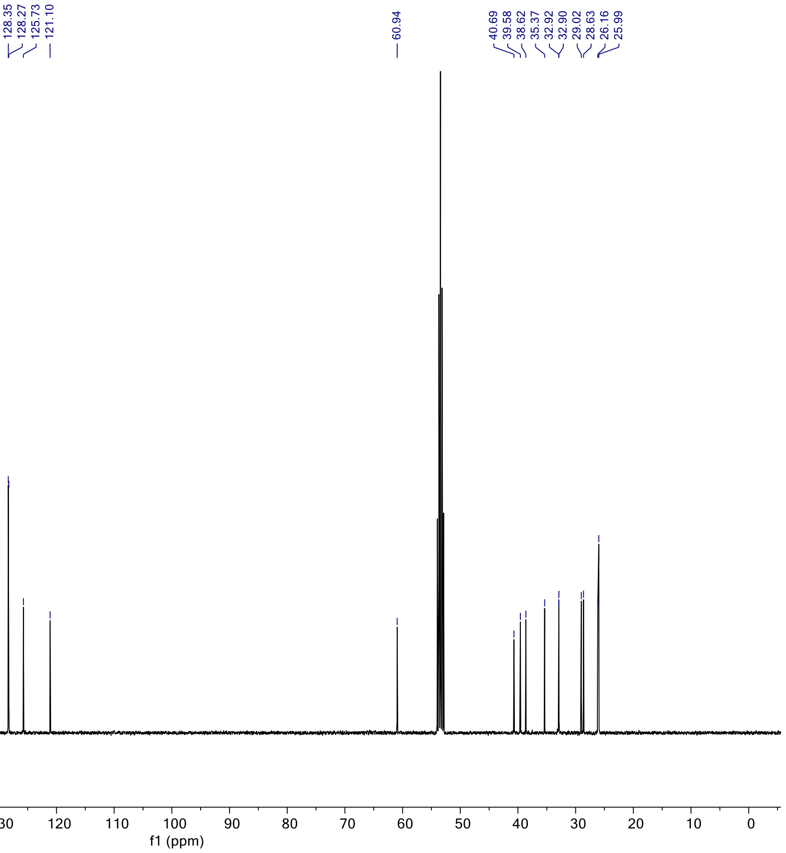

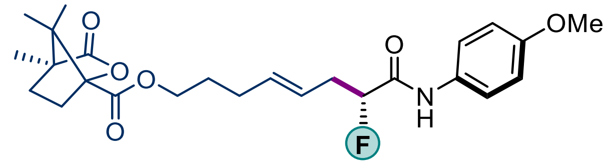

10
(**CDCl$_3$, ^{1}H NMR**)

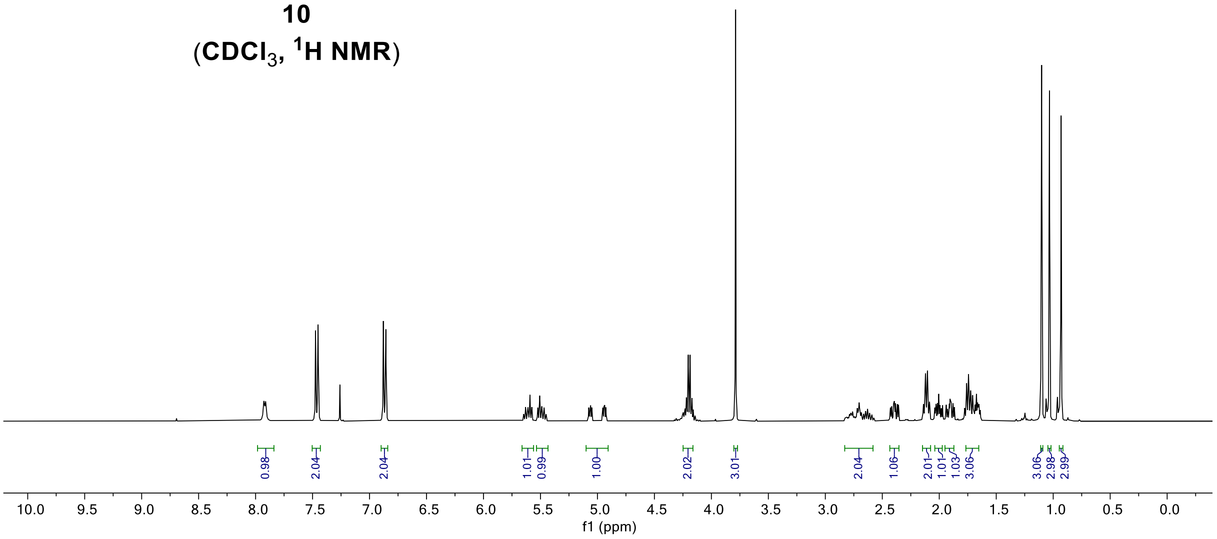

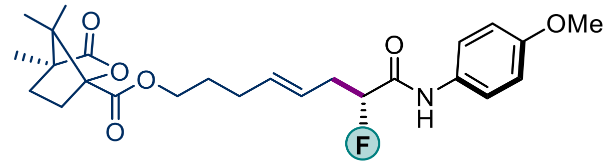

10
(**CDCl$_3$, ^{13}C NMR**)

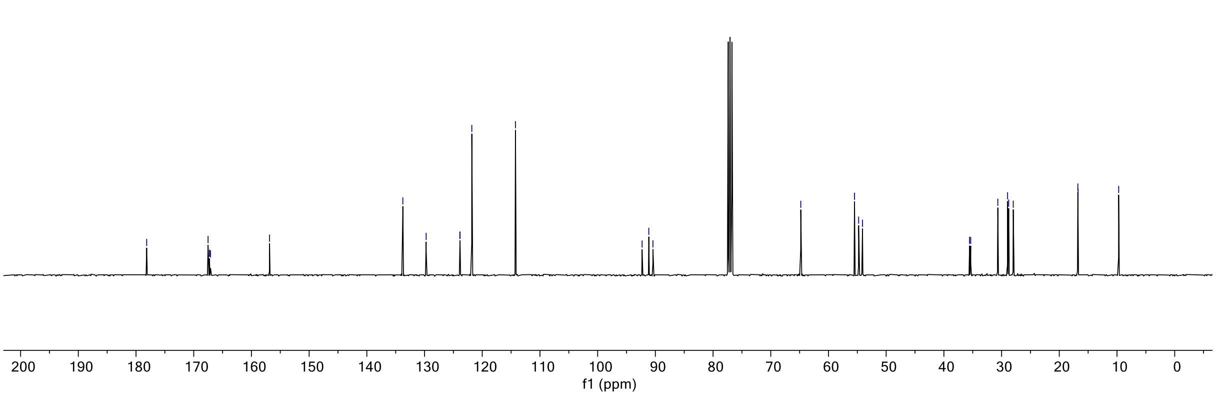

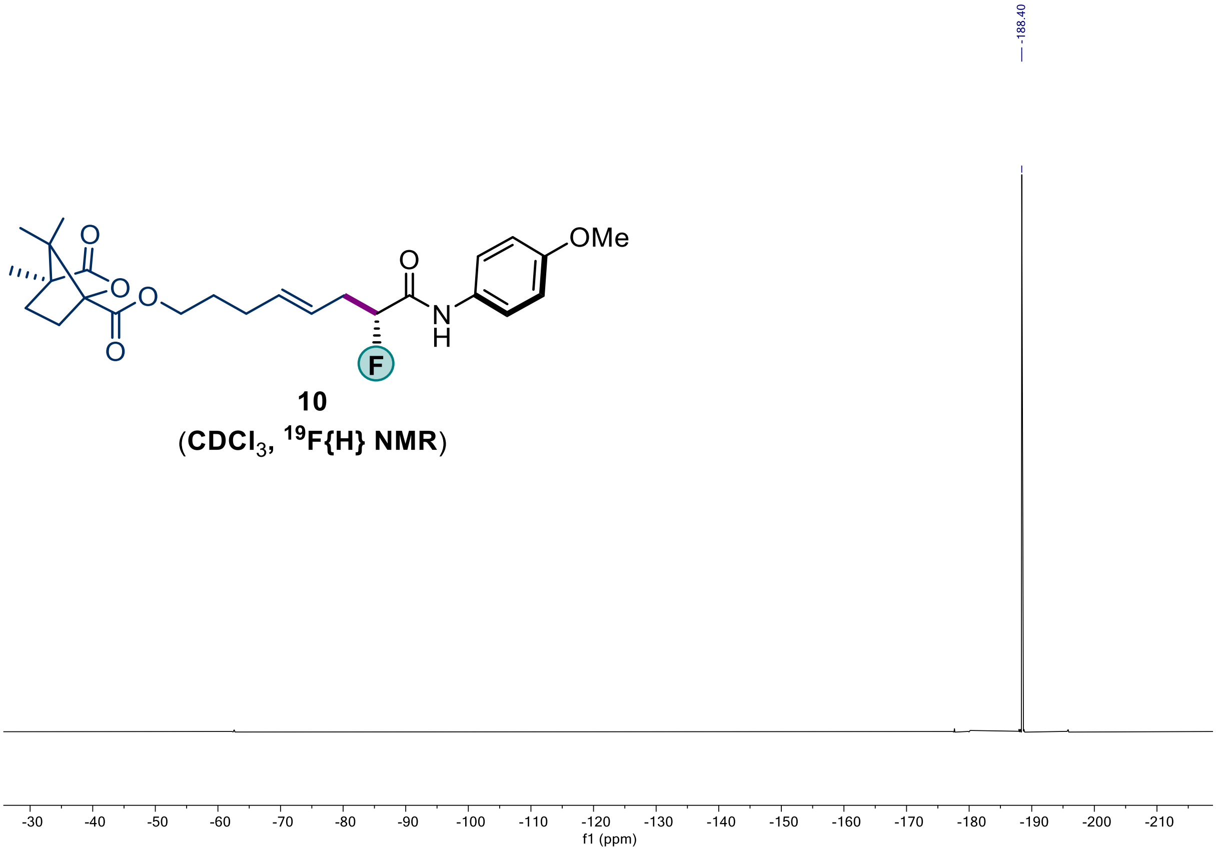

10
(**$CDCl_3$, ^{19}F{H} NMR**)

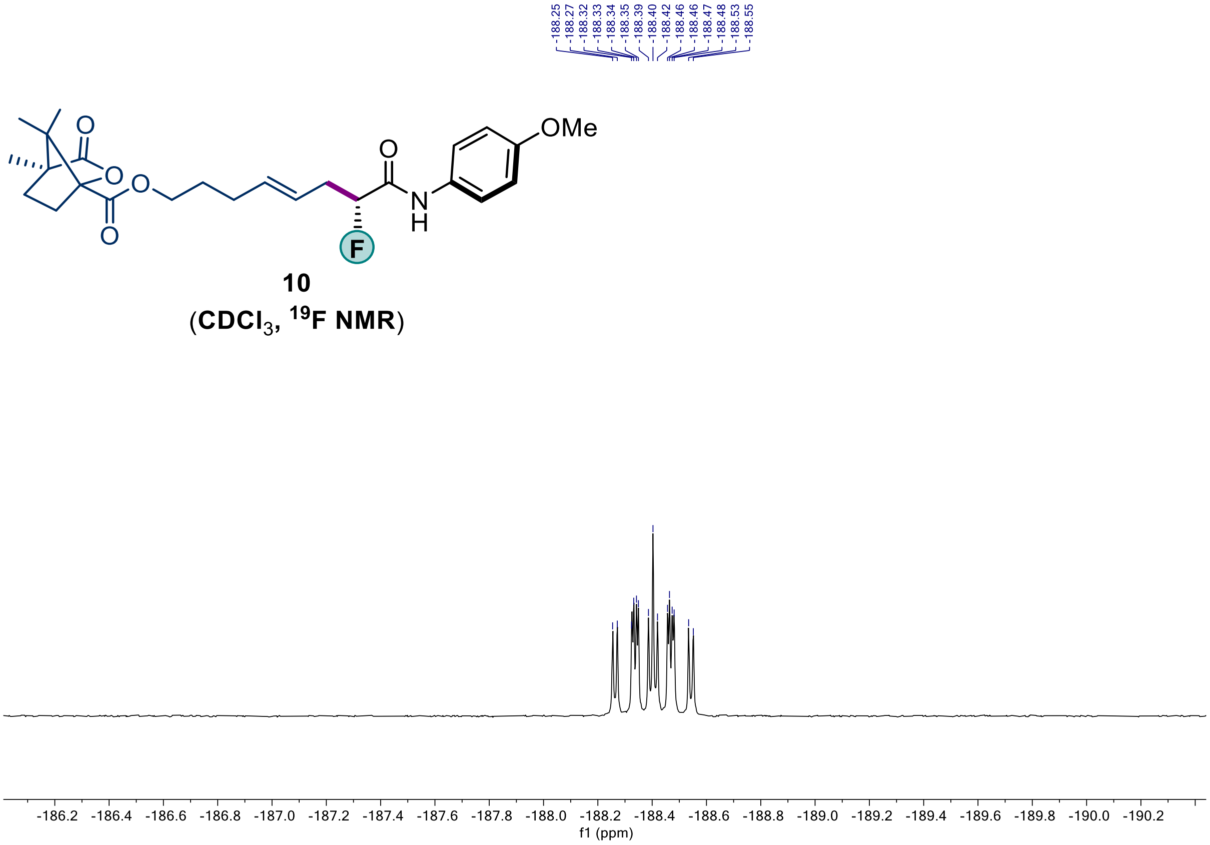

10
(**$CDCl_3$, ^{19}F NMR**)

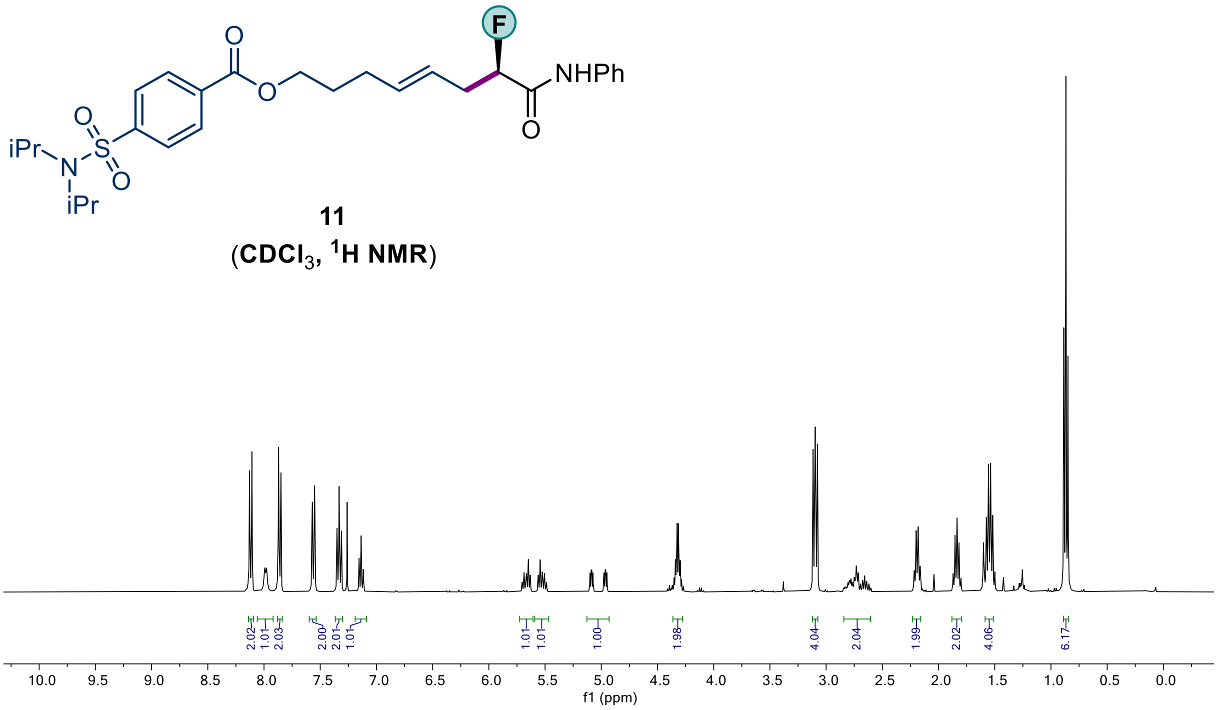

NHPh
11
(CDCl$_3$, ^{1}H NMR)
f1 (ppm)

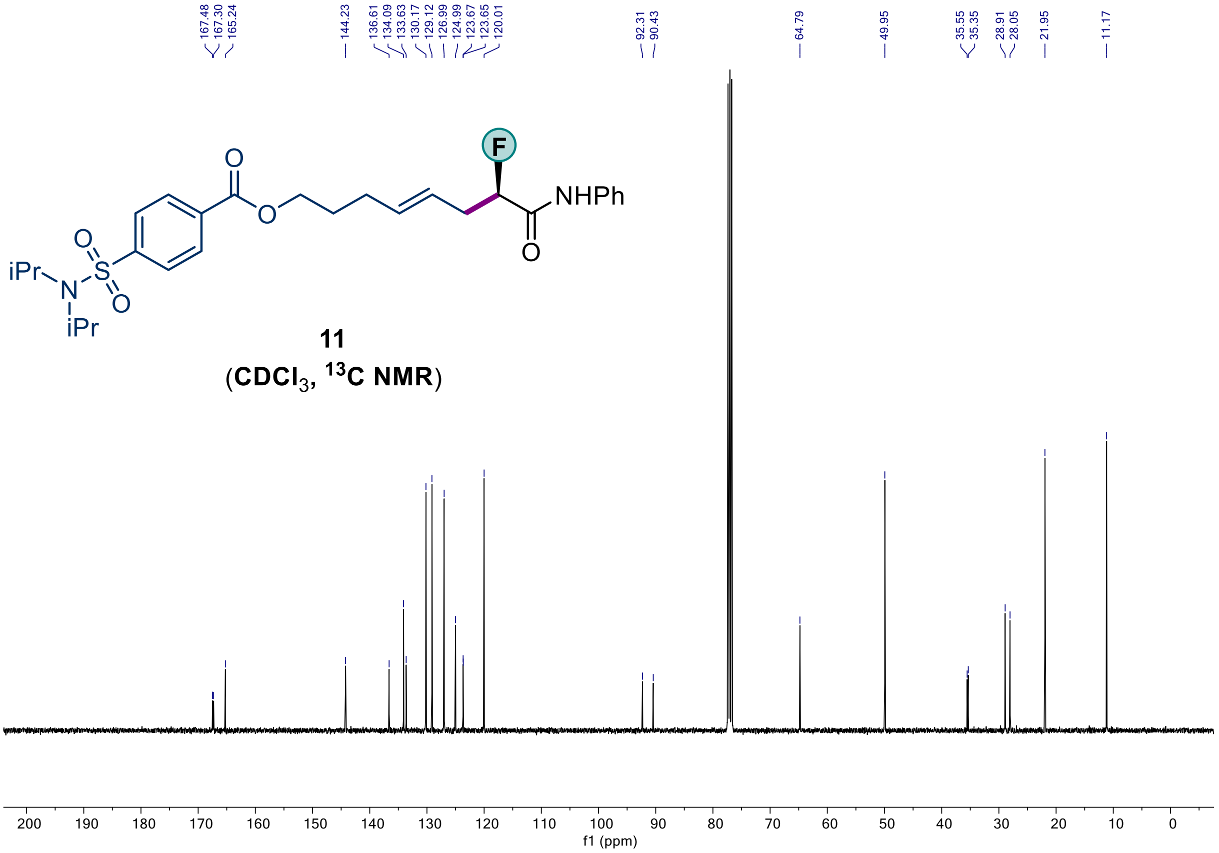

167.48
167.30
165.24
144.23
136.61
134.09
133.63
130.17
129.12
126.99
124.99
123.67
123.65
120.01
92.31
90.43
64.79
49.95
35.55
35.35
28.91
28.05
21.95
11.17
NHPh
11
(CDCl$_3$, ^{13}C NMR)
f1 (ppm)

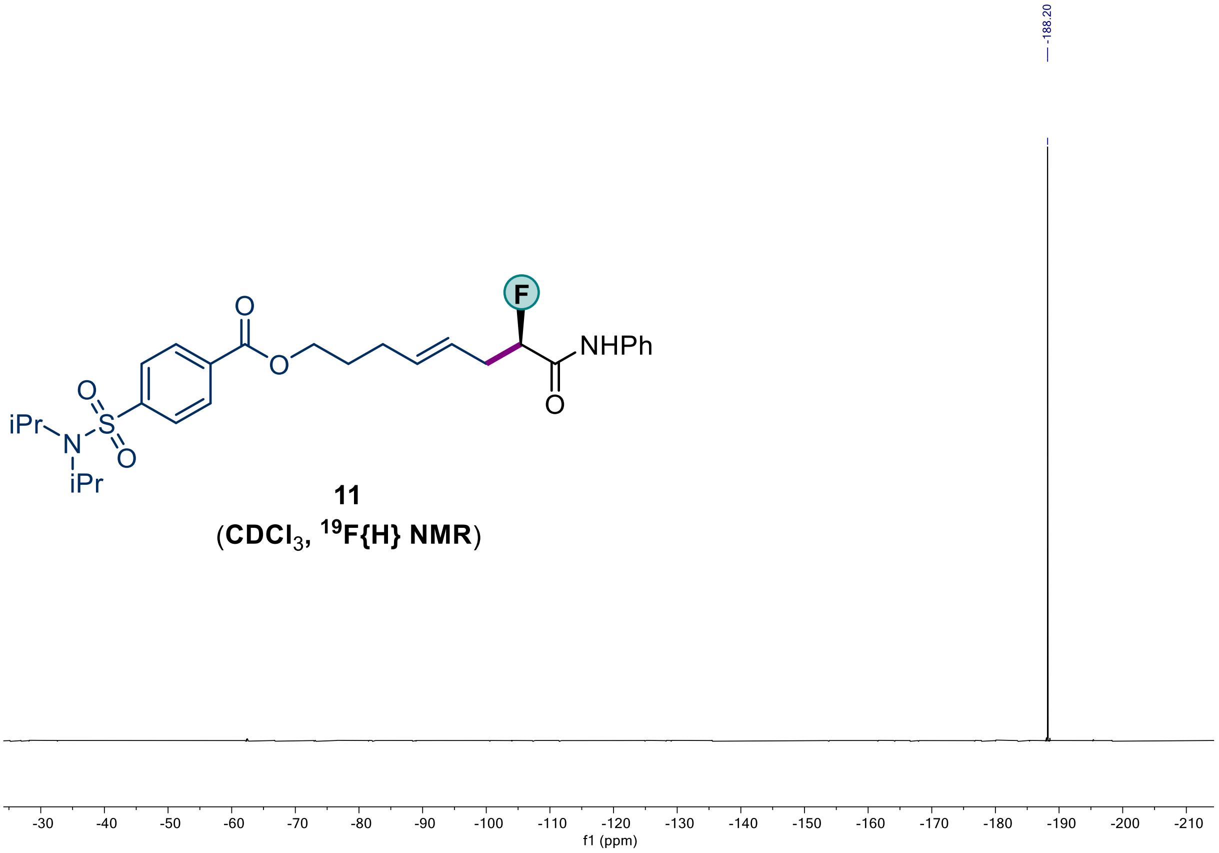
-188.20
NHPh
iPr
iPr
11
(CDCl$_3$, ^{19}F{H} NMR)
-30 -40 -50 -60 -70 -80 -90 -100 -110 -120 -130 -140 -150 -160 -170 -180 -190 -200 -210
f1 (ppm)

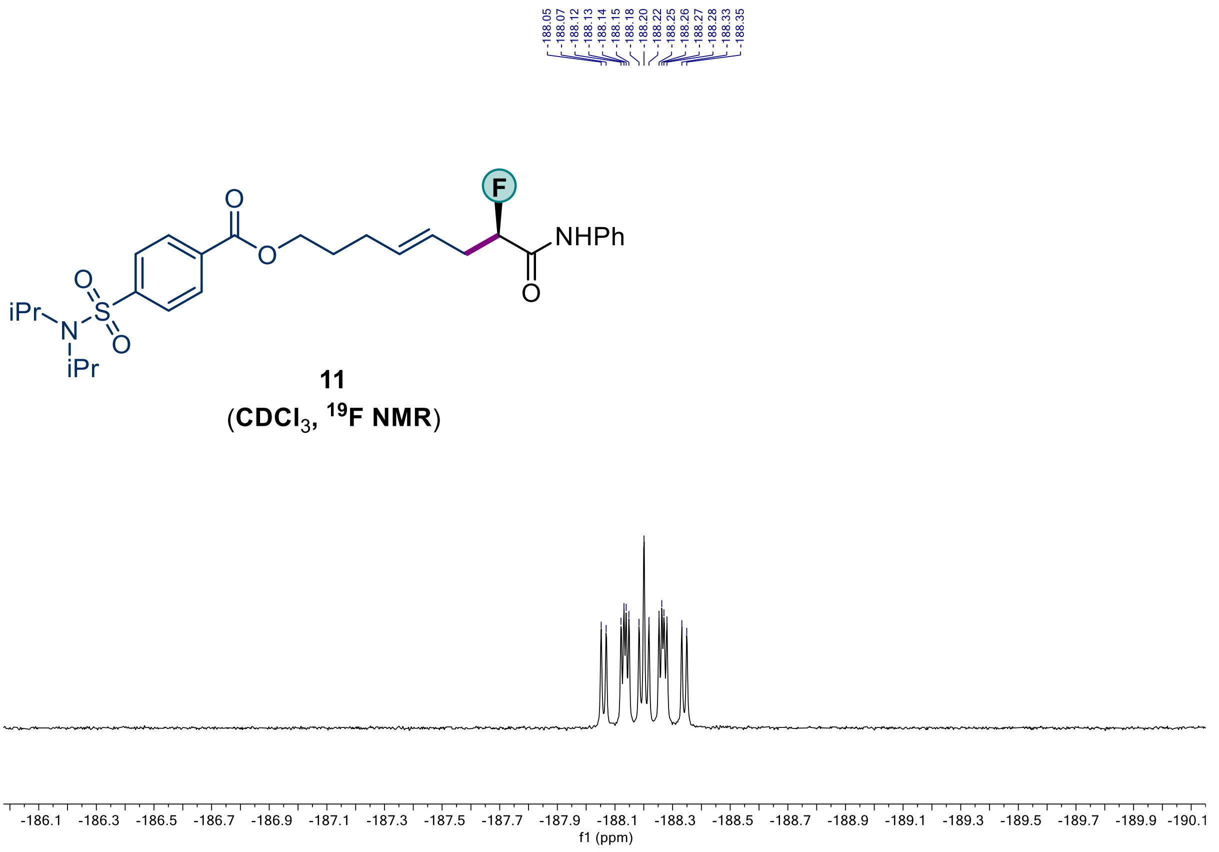
-188.05
-188.07
-188.12
-188.13
-188.14
-188.15
-188.18
-188.20
-188.22
-188.25
-188.26
-188.27
-188.28
-188.33
-188.35
NHPh
iPr
iPr
11
(CDCl$_3$, ^{19}F NMR)
-186.1 -186.3 -186.5 -186.7 -186.9 -187.1 -187.3 -187.5 -187.7 -187.9 -188.1 -188.3 -188.5 -188.7 -188.9 -189.1 -189.3 -189.5 -189.7 -189.9 -190.1
f1 (ppm)

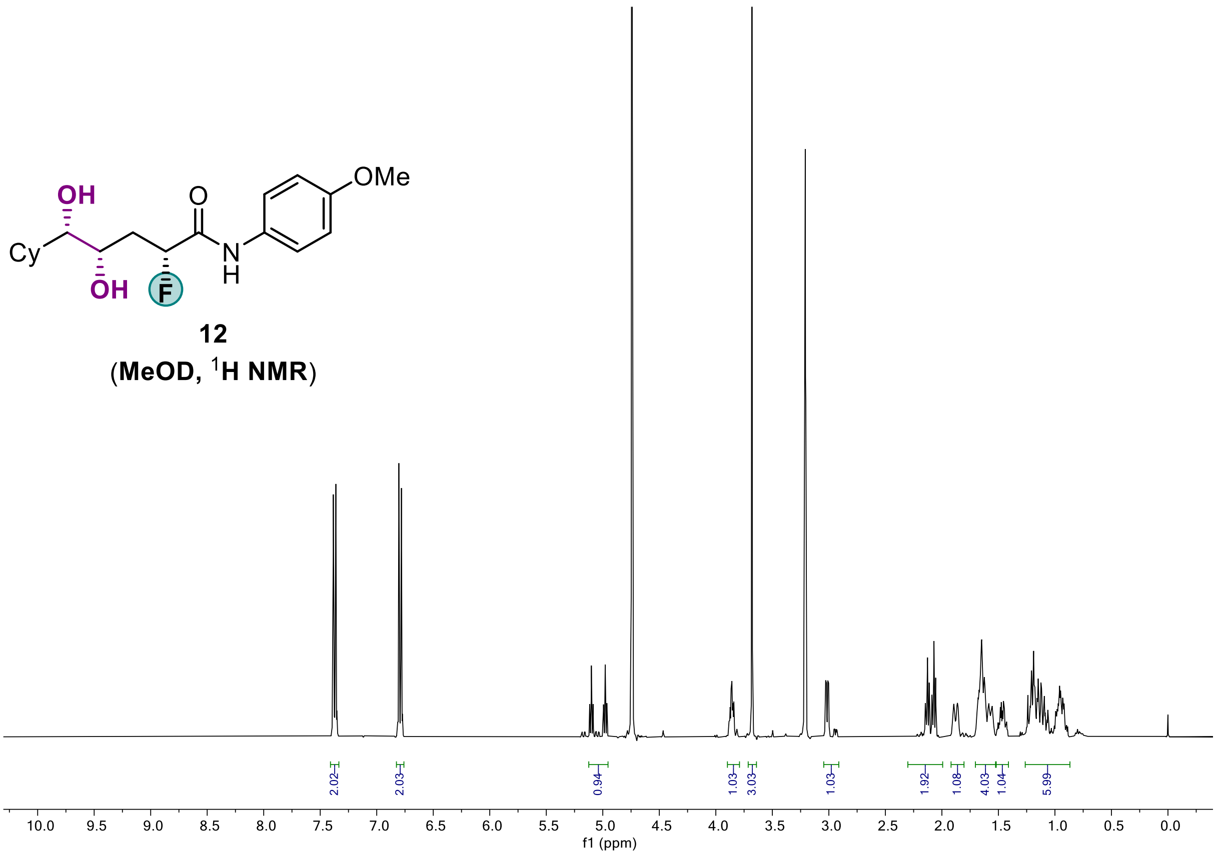
OH
Cy
OH
F
O
N
H
OMe
12
(MeOD, 1H NMR)
2.02
2.03
0.94
1.03
3.03
1.03
1.92
1.08
4.03
1.04
5.99
f1 (ppm)

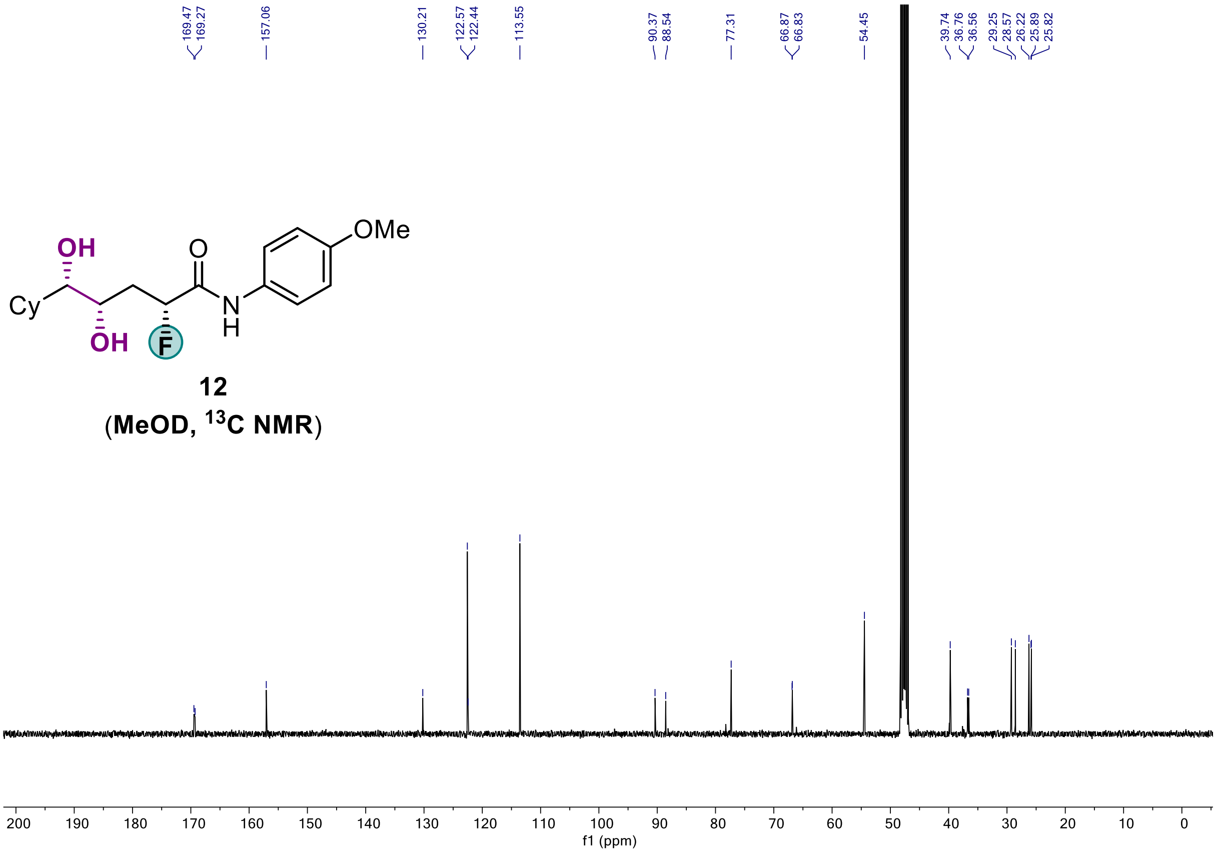
169.47
169.27
157.06
130.21
122.57
122.44
113.55
90.37
88.54
77.31
66.87
66.83
54.45
39.74
36.76
36.56
29.25
28.57
26.22
25.89
25.82
OH
Cy
OH
F
O
N
H
OMe
12
(MeOD, 13C NMR)
f1 (ppm)

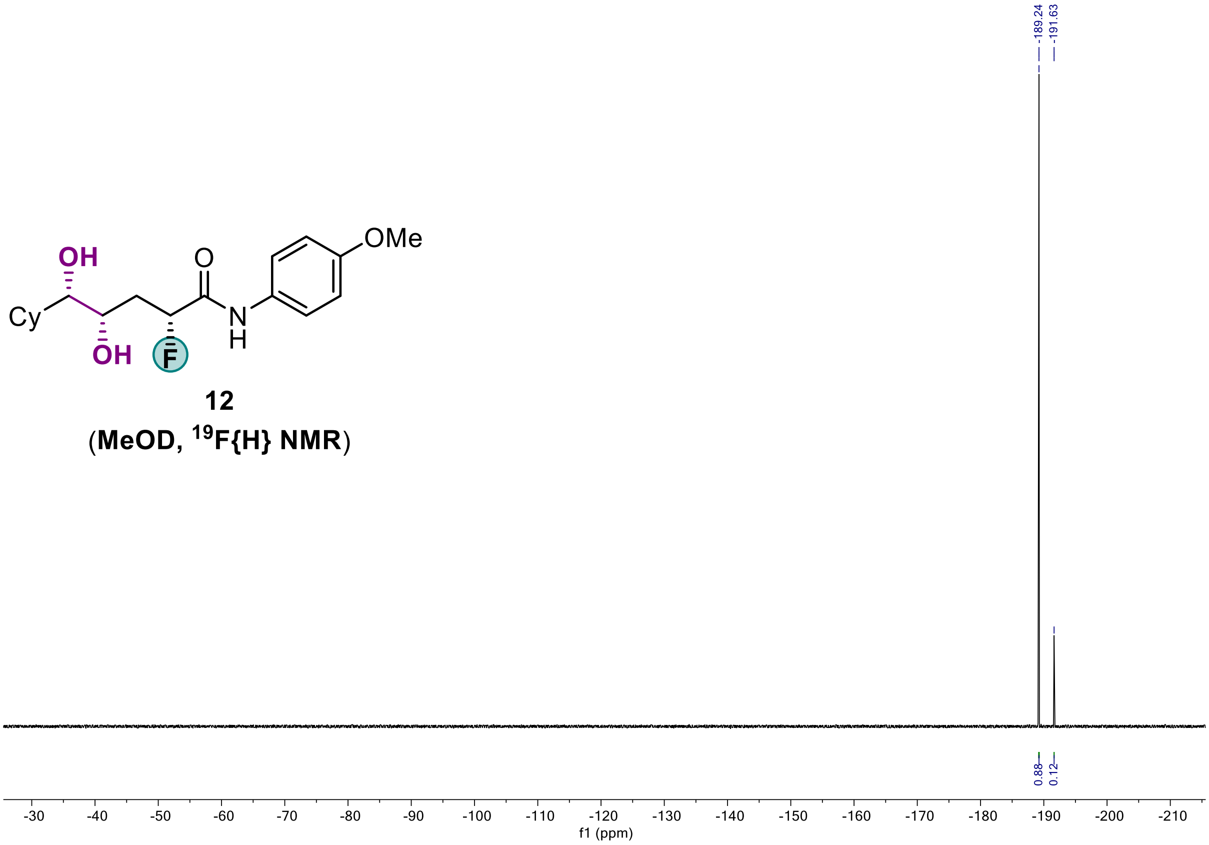
OH
OMe
O
Cy
N
H
OH
F
12
(MeOD, 19F{H} NMR)
-189.24
-191.63
0.88
0.12
f1 (ppm)

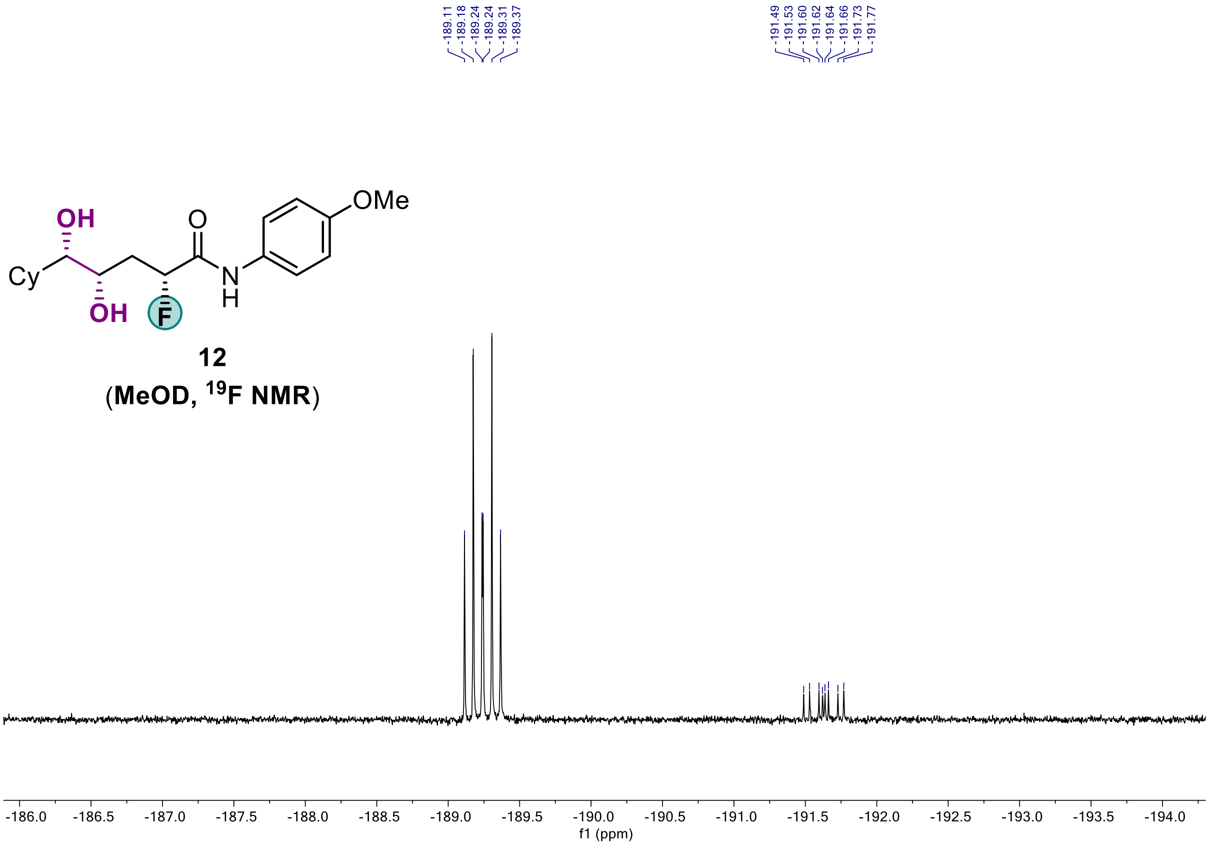
-189.11
-189.18
-189.24
-189.24
-189.31
-189.37
-191.49
-191.53
-191.60
-191.62
-191.64
-191.66
-191.73
-191.77
OH
OMe
O
Cy
N
H
OH
F
12
(MeOD, 19F NMR)
f1 (ppm)

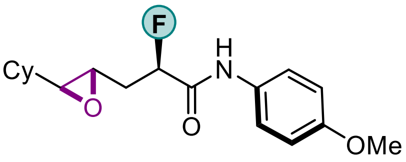

13
(**CDCl$_3$, ^{1}H NMR**)

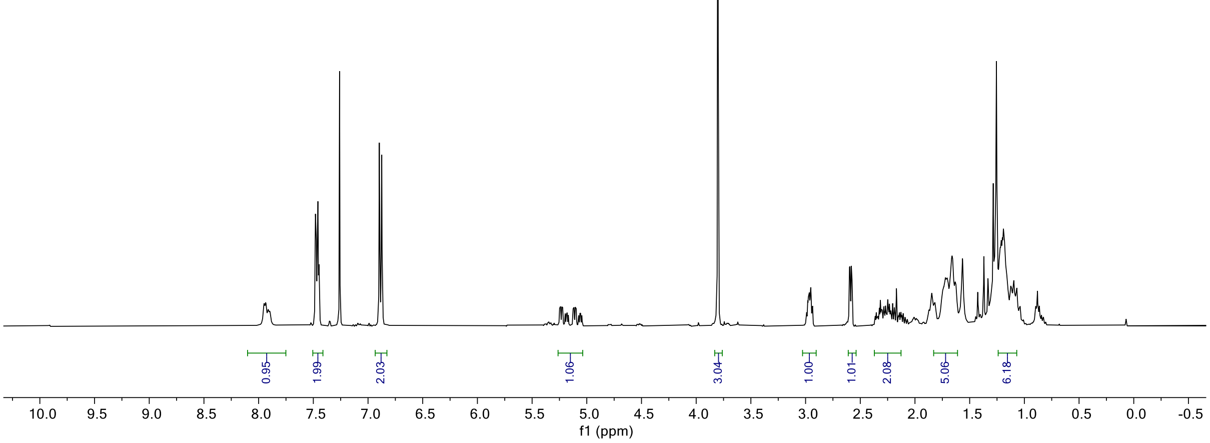

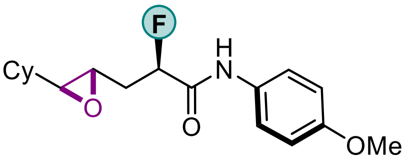

13
(**CDCl$_3$, ^{13}C NMR**)

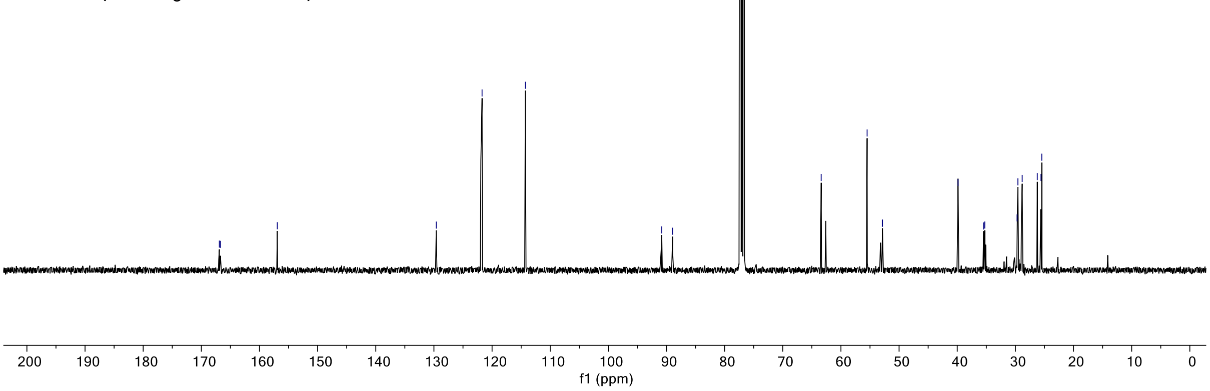

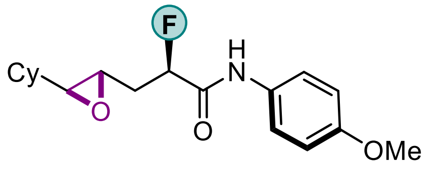

13

(**CDCl$_3$, ^{19}F{H} NMR**)

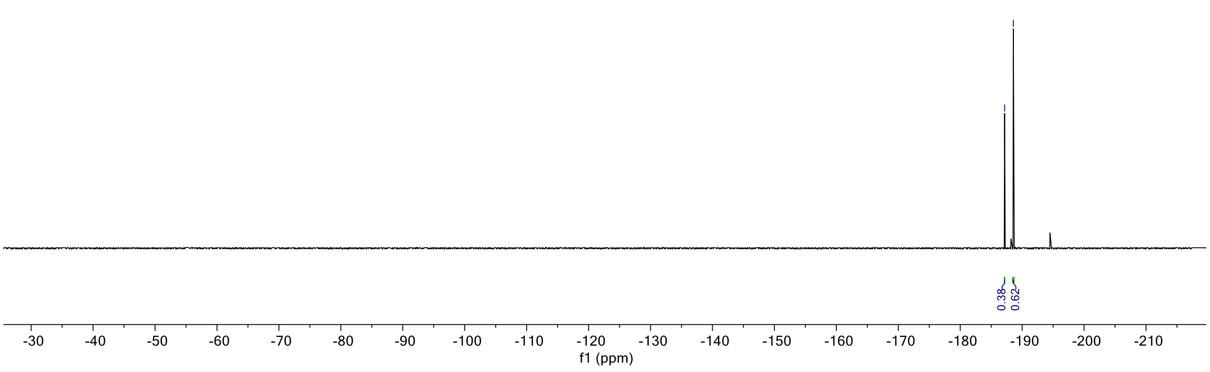

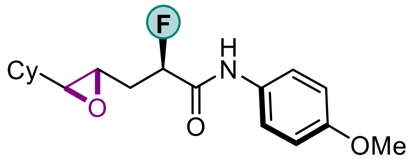

13

(**CDCl$_3$, ^{19}F NMR**)

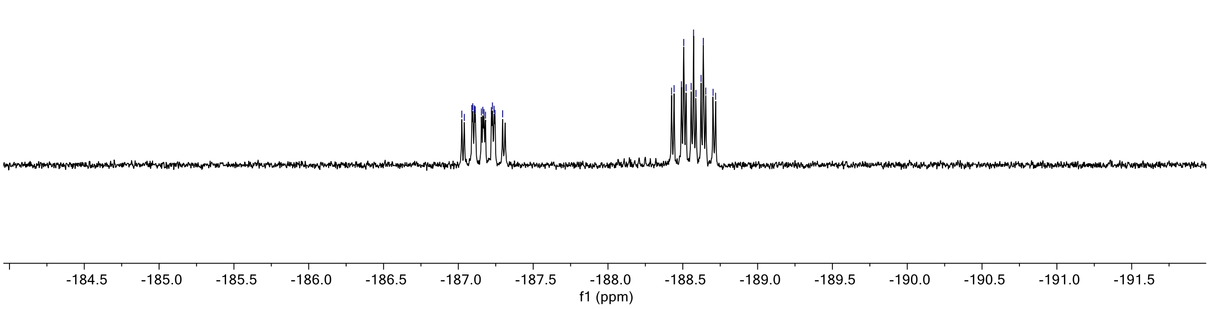

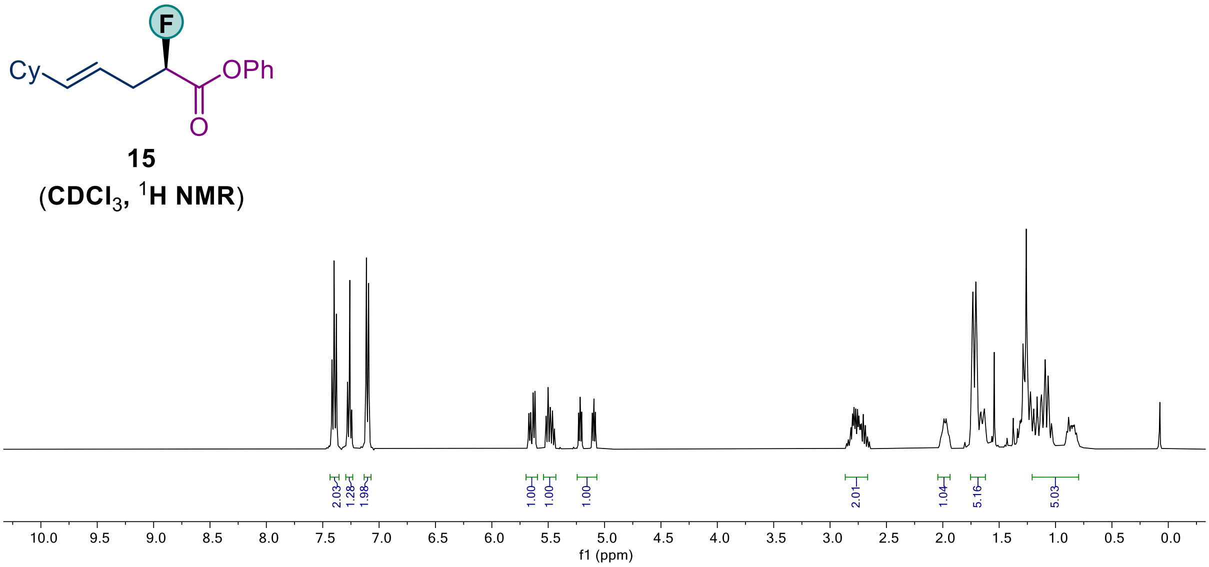

15

(**CDCl$_3$, ^{1}H NMR**)

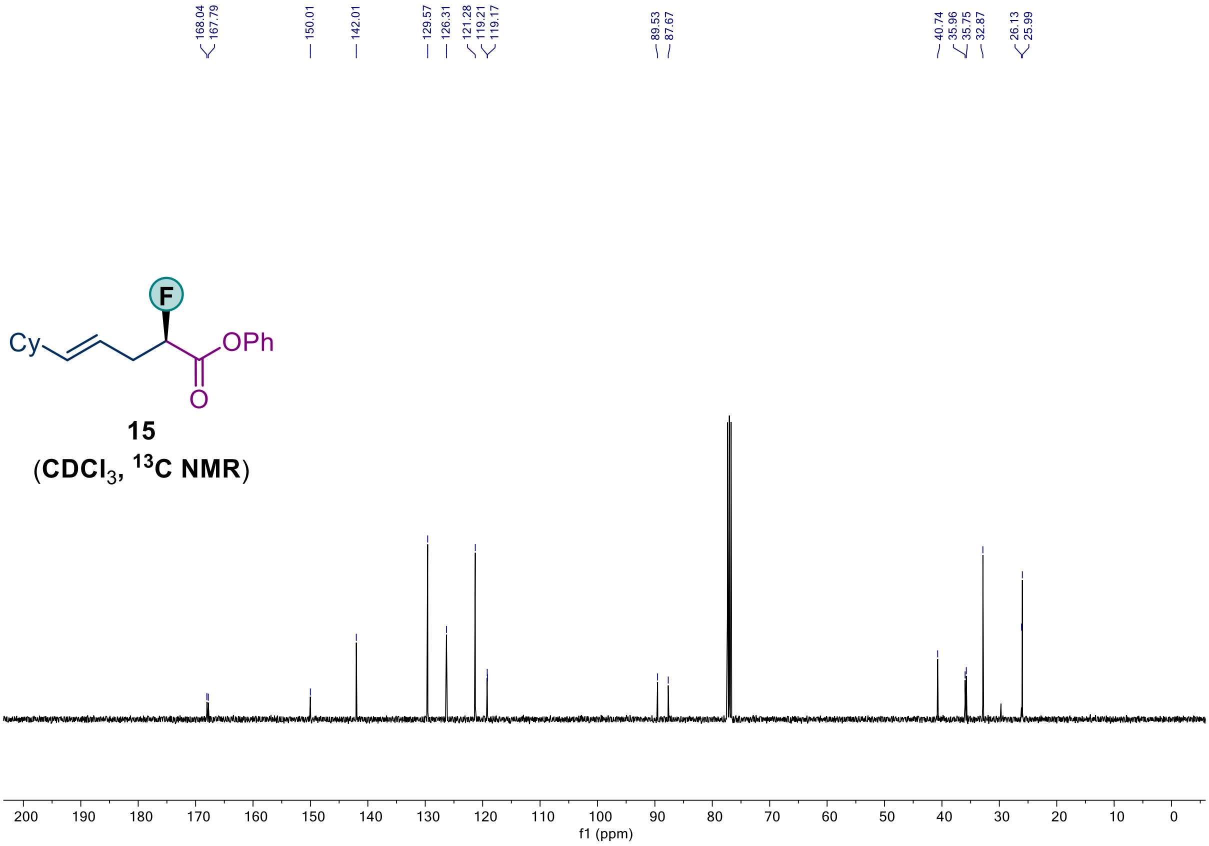

15

(**CDCl$_3$, ^{13}C NMR**)

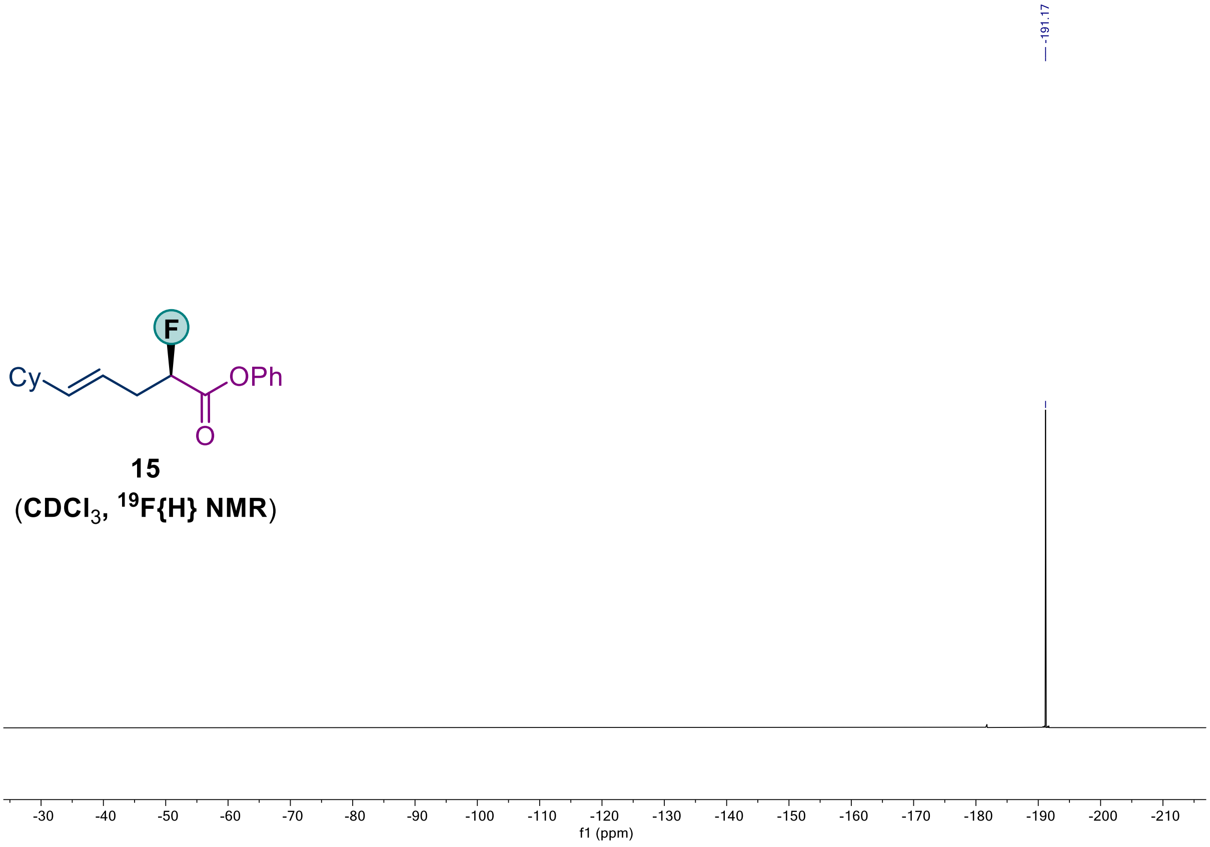

-191.17
Cy
F
OPh
O
15
(CDCl3, 19F{H} NMR)
-30 -40 -50 -60 -70 -80 -90 -100 -110 -120 -130 -140 -150 -160 -170 -180 -190 -200 -210
f1 (ppm)

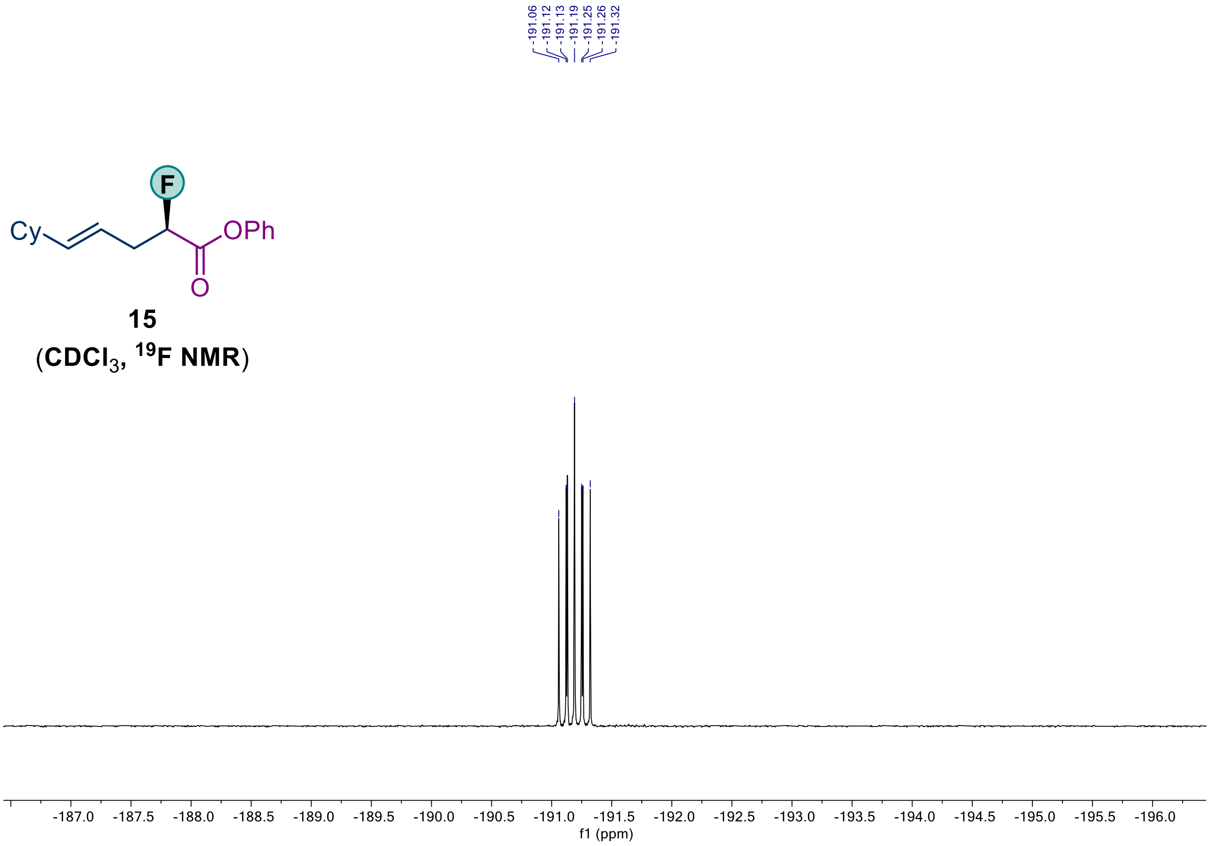

-191.06
-191.12
-191.13
-191.19
-191.25
-191.26
-191.32
Cy
F
OPh
O
15
(CDCl3, 19F NMR)
-187.0 -187.5 -188.0 -188.5 -189.0 -189.5 -190.0 -190.5 -191.0 -191.5 -192.0 -192.5 -193.0 -193.5 -194.0 -194.5 -195.0 -195.5 -196.0
f1 (ppm)

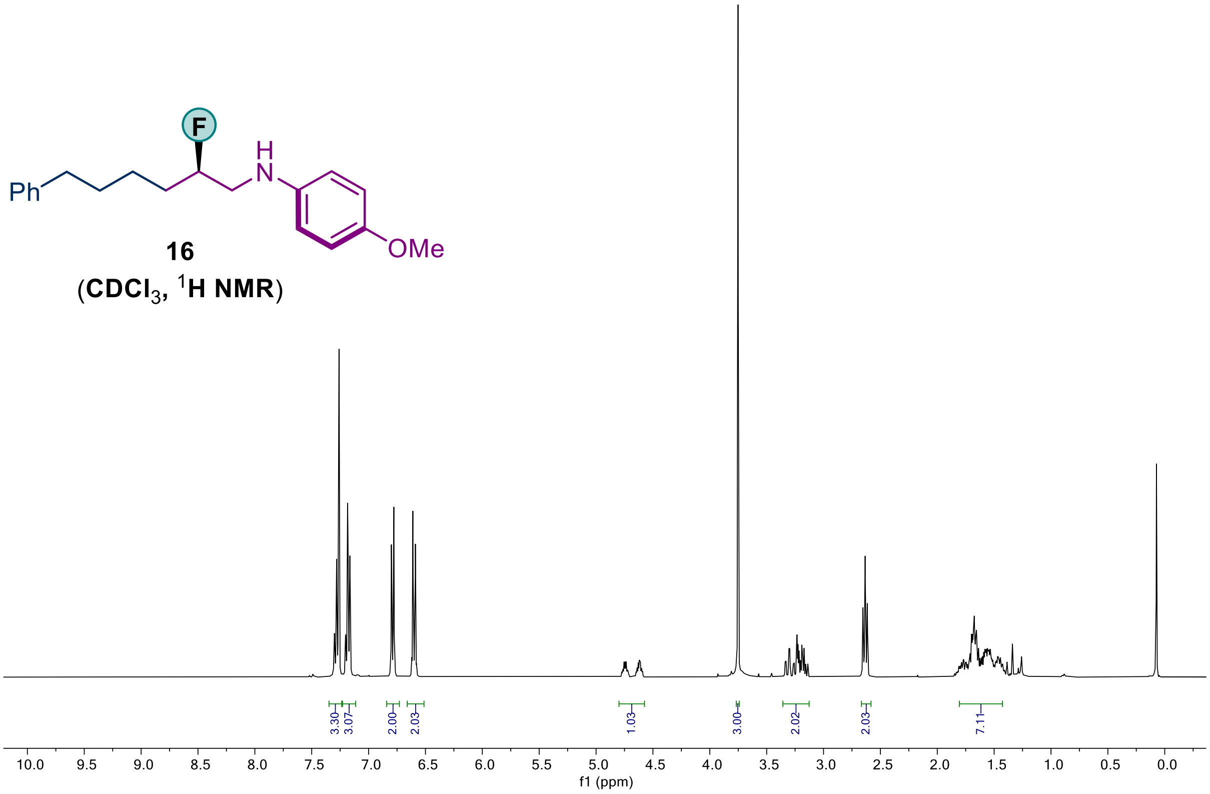
Ph
F
H
N
OMe
16
(CDCl3, 1H NMR)
3.30
3.07
2.00
2.03
1.03
3.00
2.02
2.03
7.11
10.0 9.5 9.0 8.5 8.0 7.5 7.0 6.5 6.0 5.5 5.0 4.5 4.0 3.5 3.0 2.5 2.0 1.5 1.0 0.5 0.0
f1 (ppm)

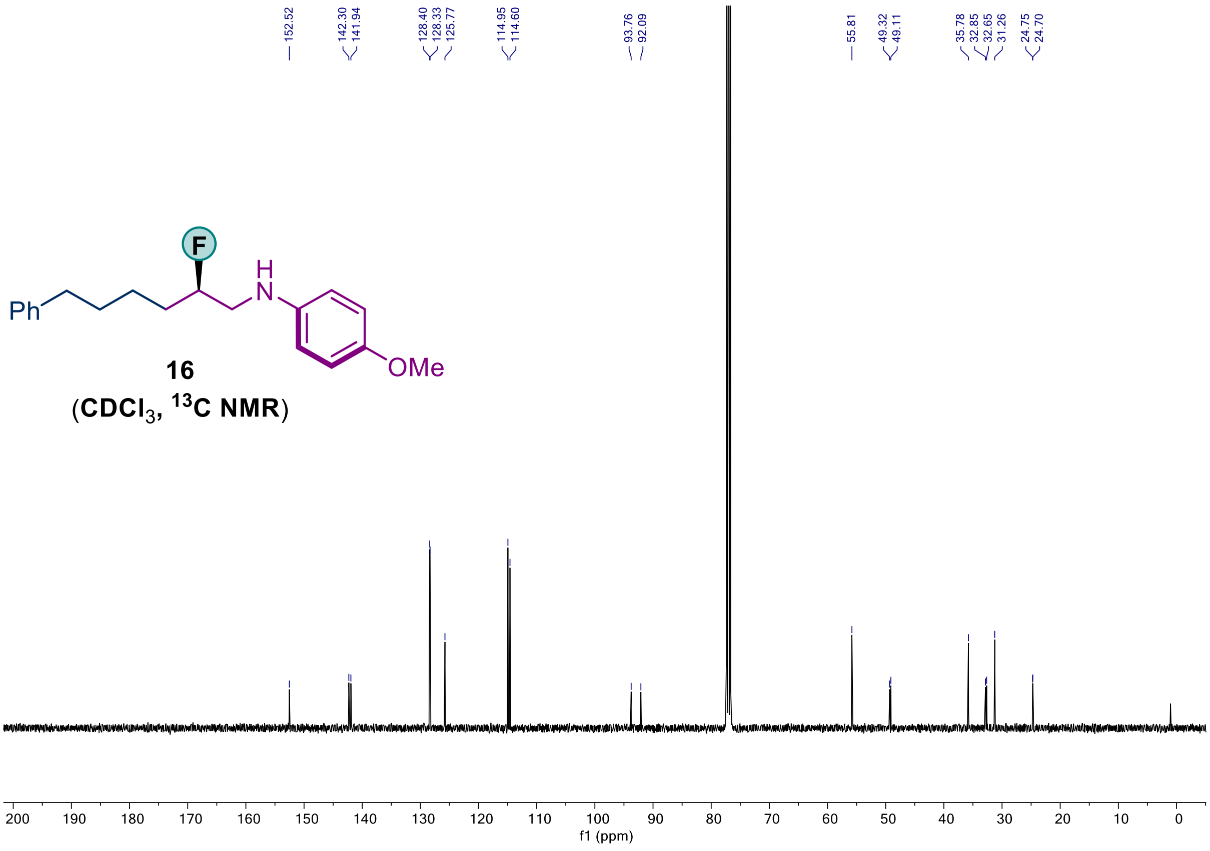
152.52
142.30
141.94
128.40
128.33
125.77
114.95
114.60
93.76
92.09
55.81
49.32
49.11
35.78
32.85
32.65
31.26
24.75
24.70
Ph
F
H
N
OMe
16
(CDCl3, 13C NMR)
200 190 180 170 160 150 140 130 120 110 100 90 80 70 60 50 40 30 20 10 0
f1 (ppm)

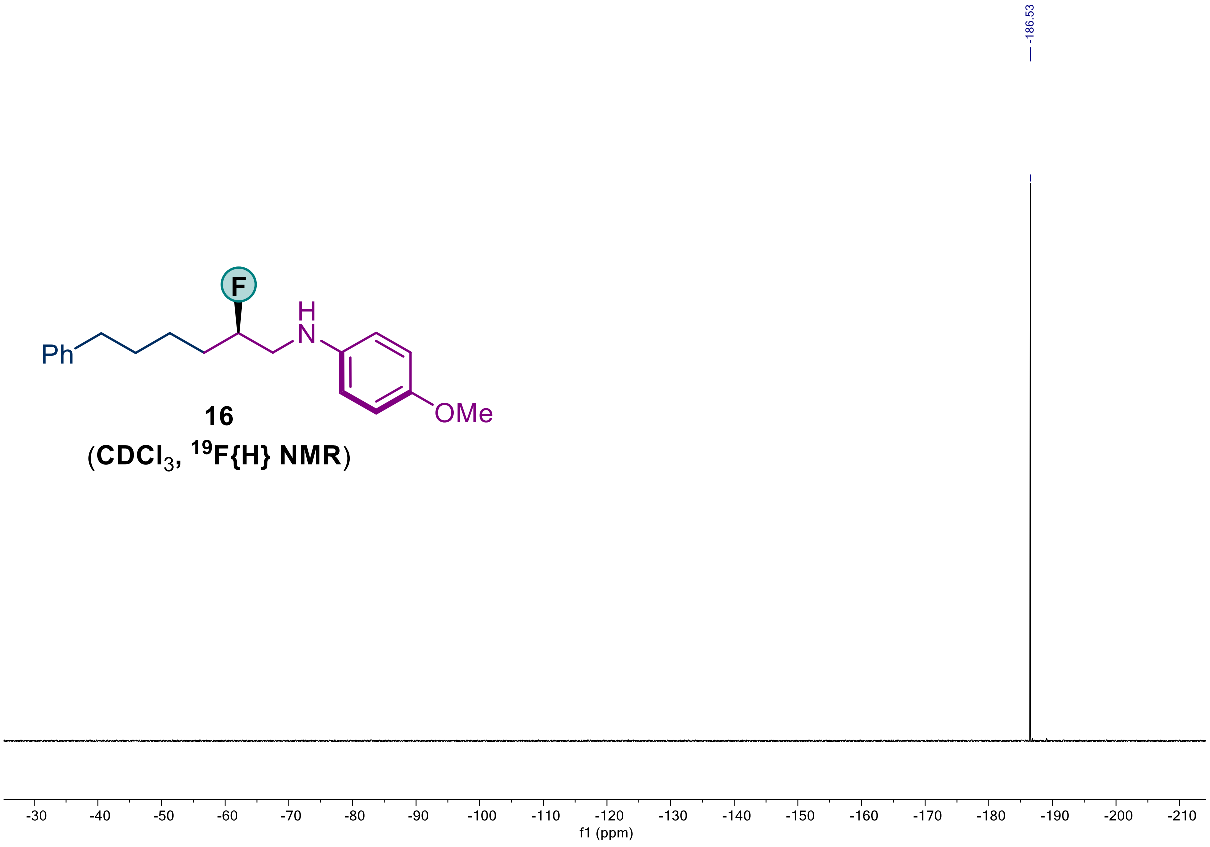
-186.53
Ph
H
N
F
OMe
16
(CDCl3, 19F{H} NMR)
-30
-40
-50
-60
-70
-80
-90
-100
-110
-120
-130
-140
-150
-160
-170
-180
-190
-200
-210
f1 (ppm)

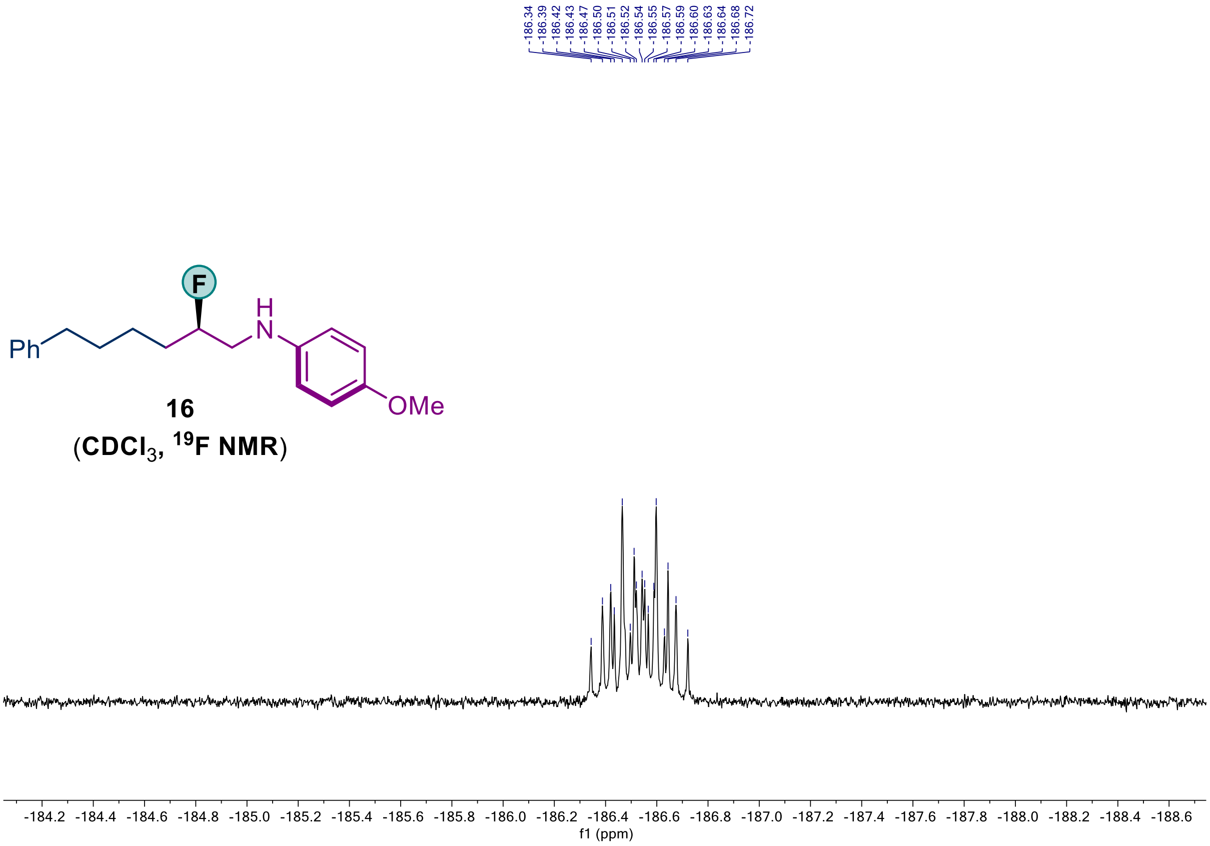
-186.34
-186.39
-186.42
-186.43
-186.47
-186.50
-186.51
-186.52
-186.54
-186.55
-186.57
-186.59
-186.60
-186.63
-186.64
-186.68
-186.72
Ph
H
N
F
OMe
16
(CDCl3, 19F NMR)
-184.2
-184.4
-184.6
-184.8
-185.0
-185.2
-185.4
-185.6
-185.8
-186.0
-186.2
-186.4
-186.6
-186.8
-187.0
-187.2
-187.4
-187.6
-187.8
-188.0
-188.2
-188.4
-188.6
f1 (ppm)

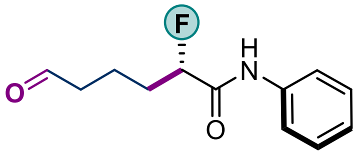

5cb-2
($CDCl_3$, ^{1}H NMR)

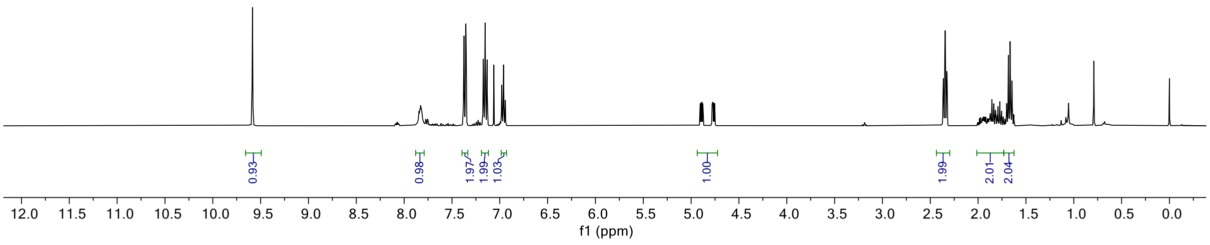

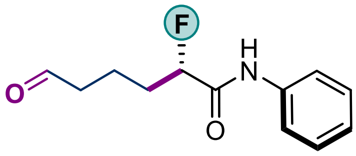

5cb-2
($CDCl_3$, ^{13}C NMR)

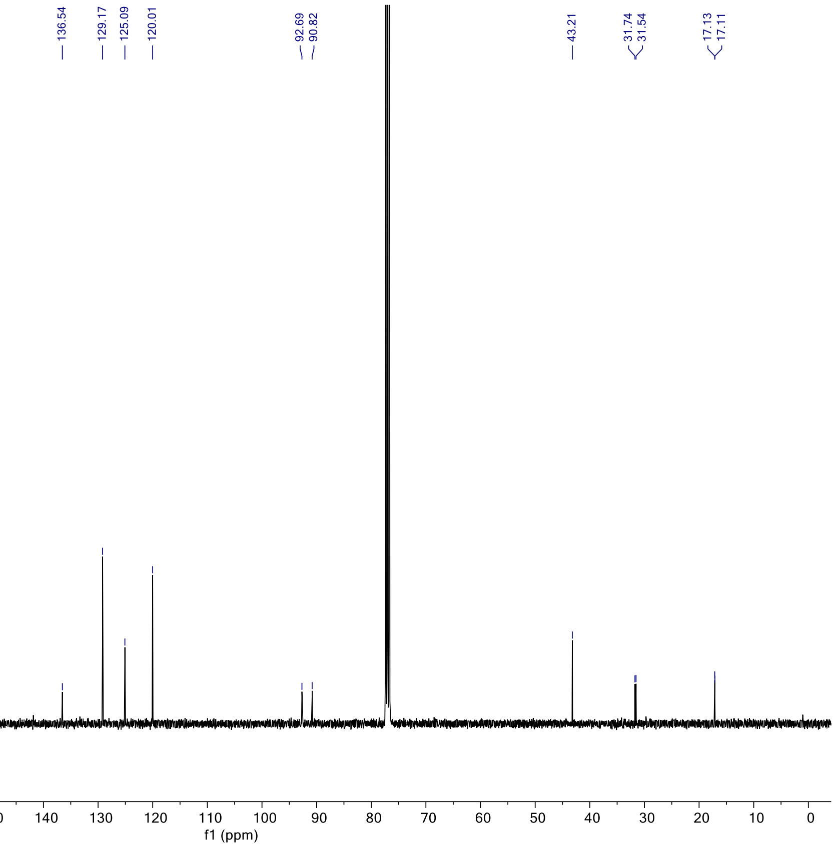

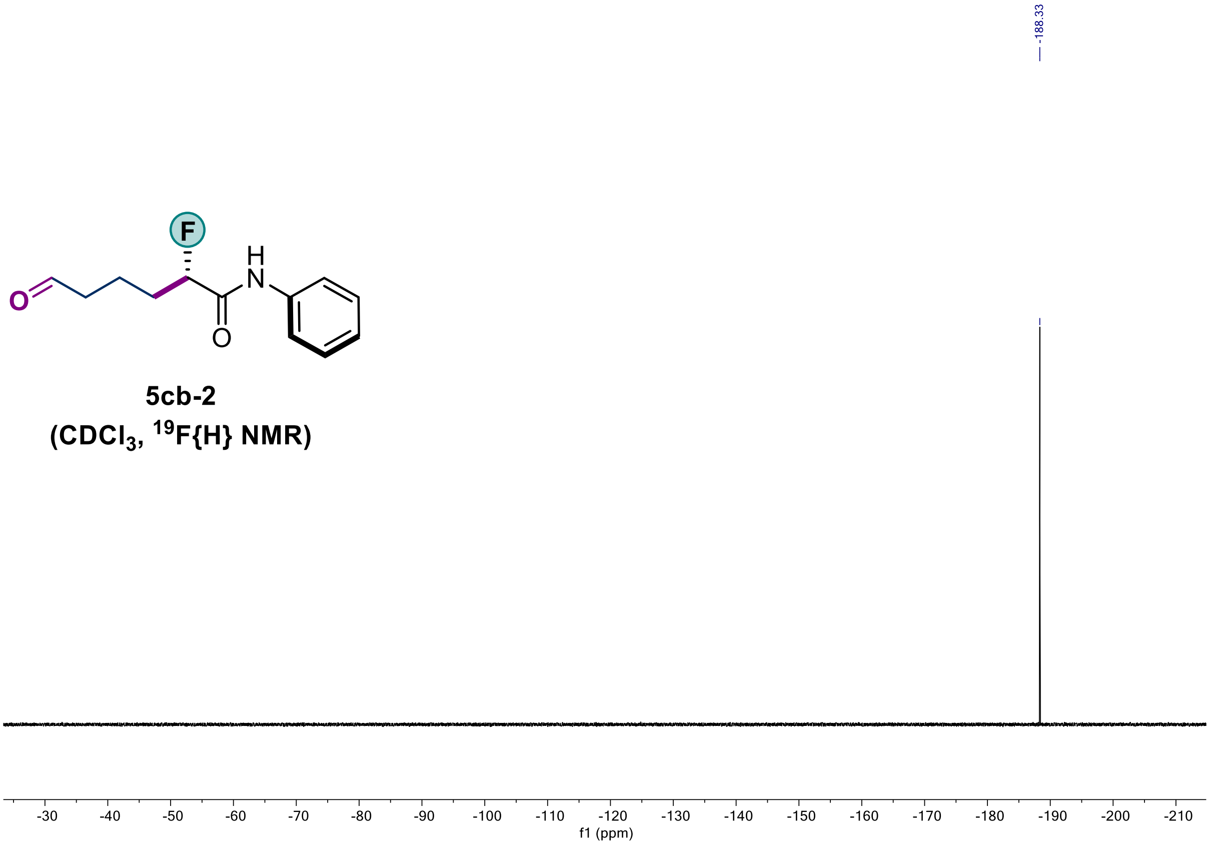

-188.33
O
F
H
N
O
5cb-2
(CDCl3, 19F{H} NMR)
-30 -40 -50 -60 -70 -80 -90 -100 -110 -120 -130 -140 -150 -160 -170 -180 -190 -200 -210
f1 (ppm)

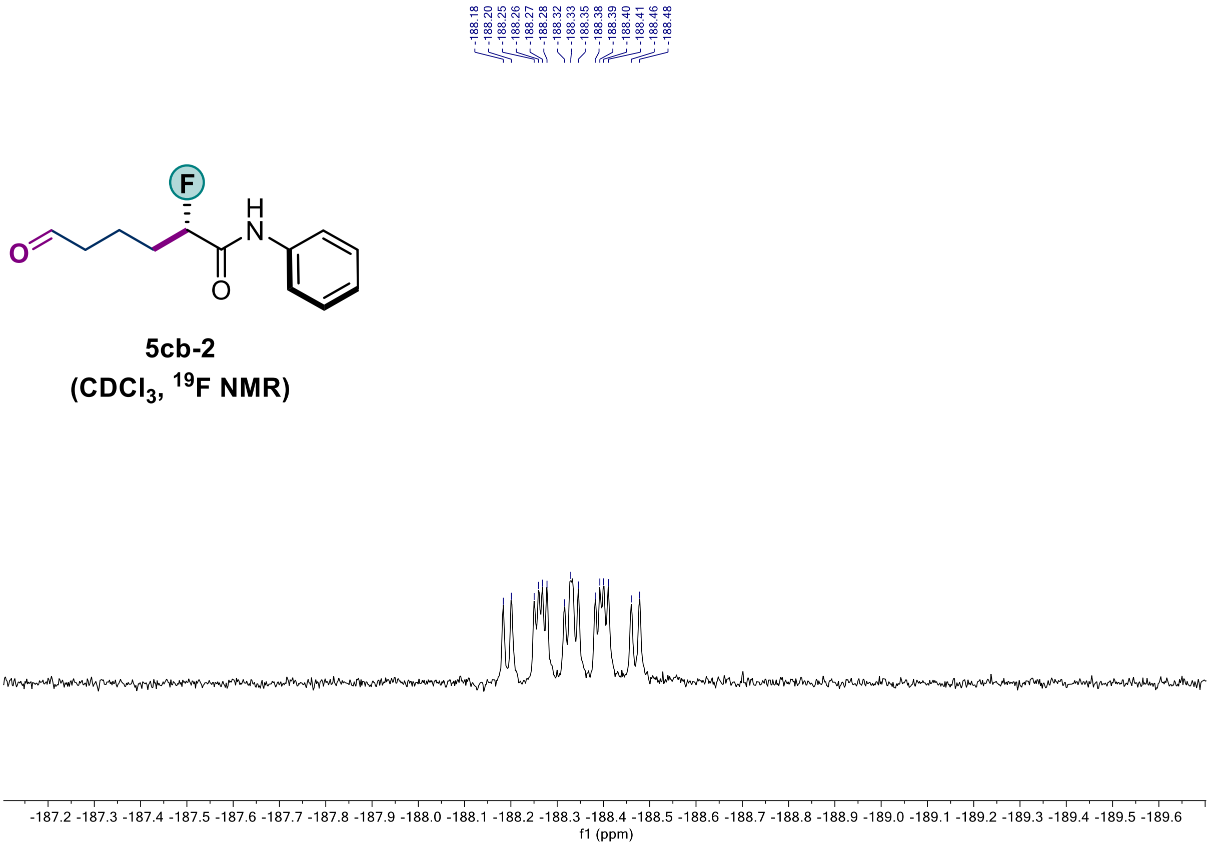

-188.18
-188.20
-188.25
-188.26
-188.27
-188.28
-188.32
-188.33
-188.35
-188.38
-188.39
-188.40
-188.41
-188.46
-188.48
O
F
H
N
O
5cb-2
(CDCl3, 19F NMR)
-187.2 -187.3 -187.4 -187.5 -187.6 -187.7 -187.8 -187.9 -188.0 -188.1 -188.2 -188.3 -188.4 -188.5 -188.6 -188.7 -188.8 -188.9 -189.0 -189.1 -189.2 -189.3 -189.4 -189.5 -189.6
f1 (ppm)

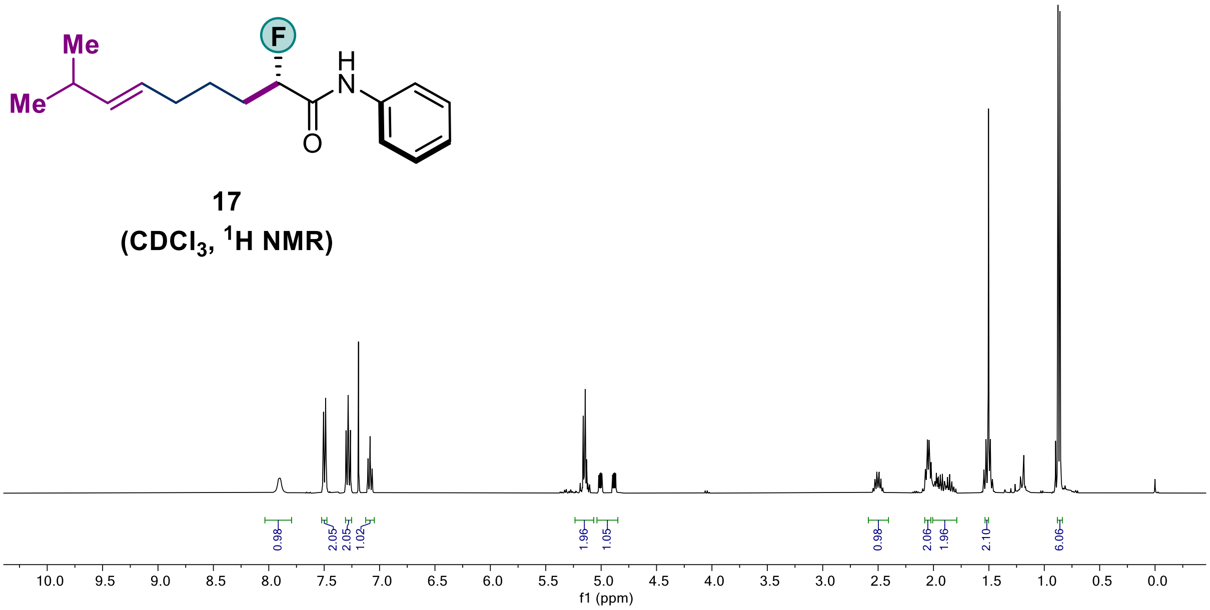

17
($CDCl_3$, ^{1}H NMR)

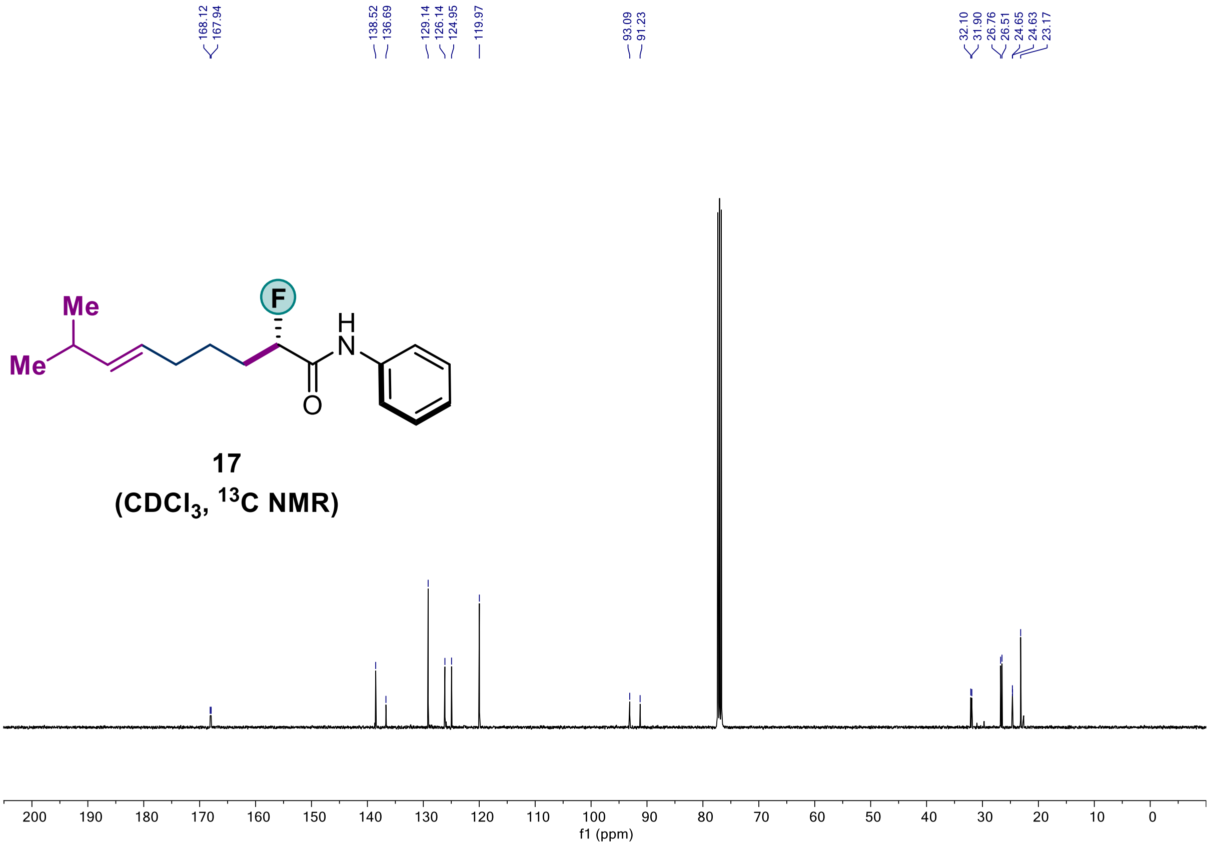

17
($CDCl_3$, ^{13}C NMR)

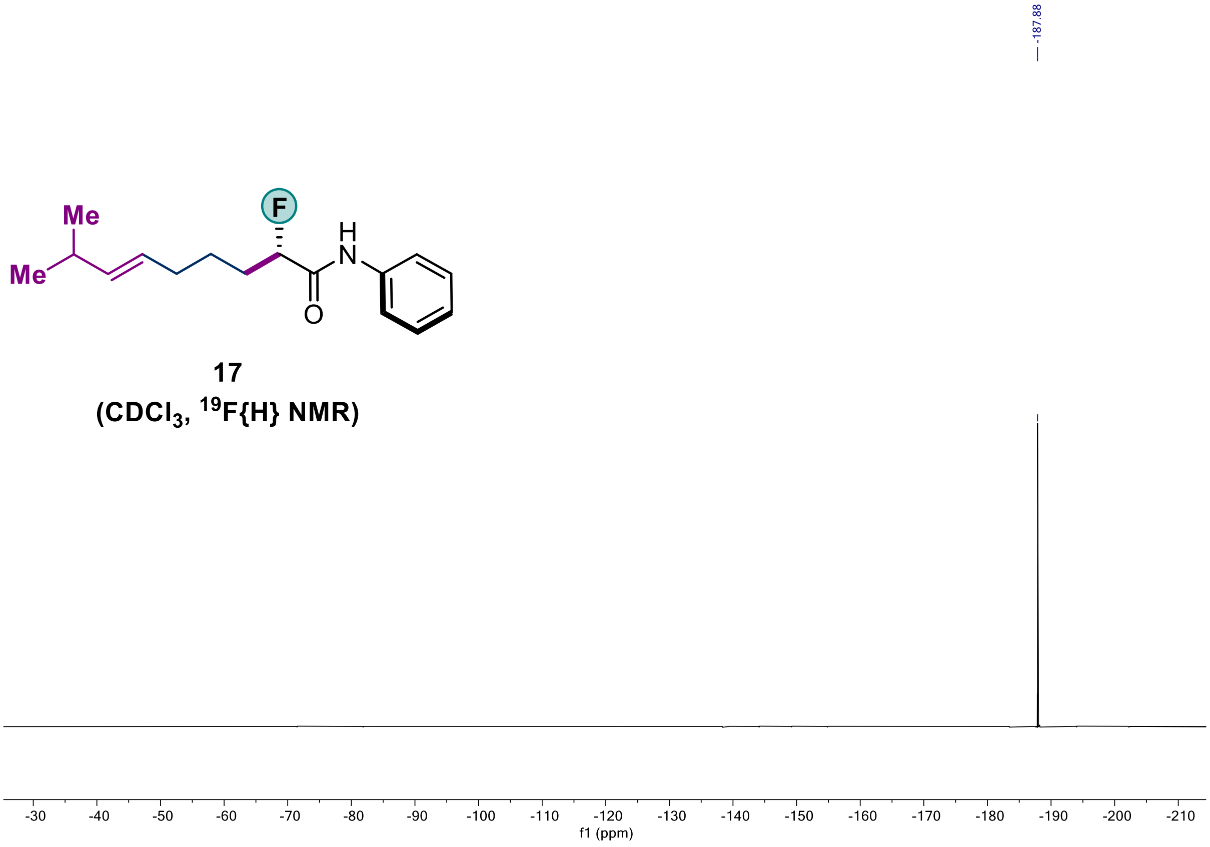

17
($CDCl_3$, $^{19}F\{H\}$ NMR)

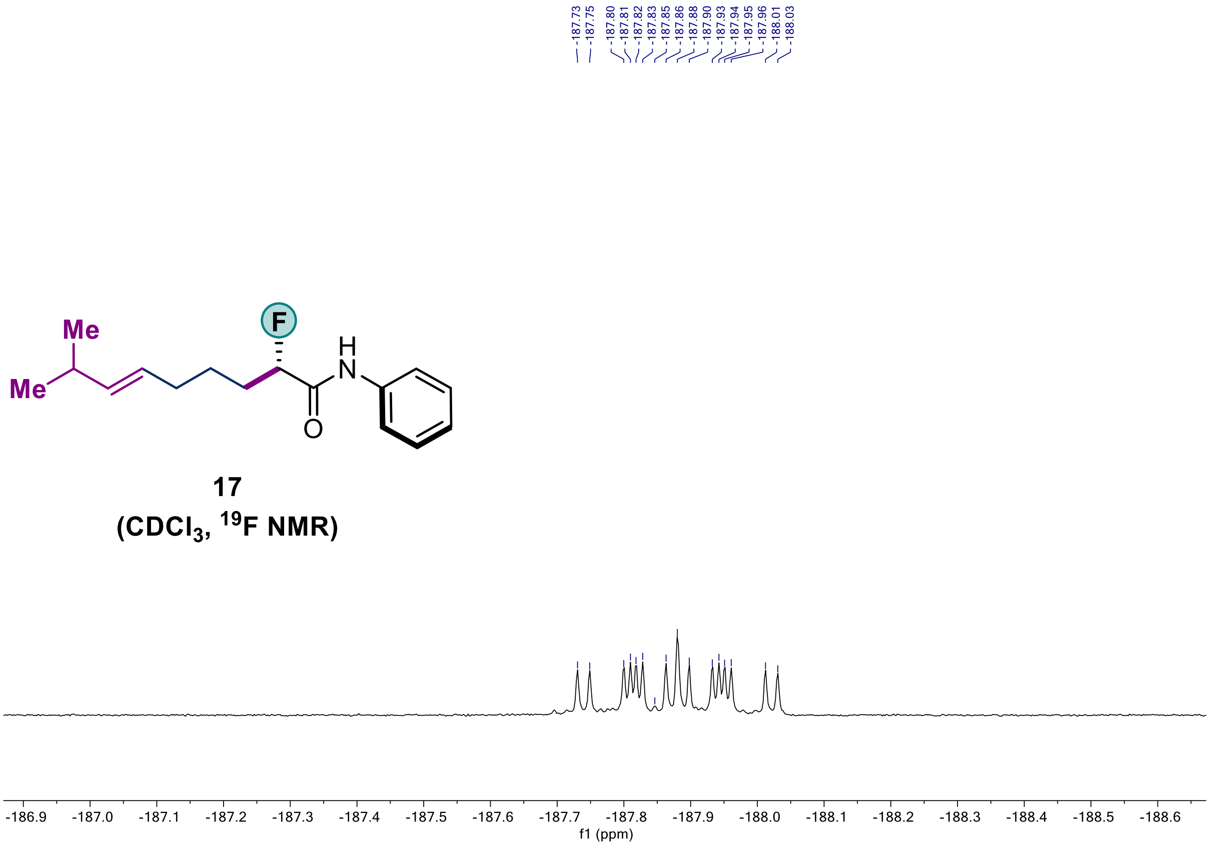

17
($CDCl_3$, ^{19}F NMR)

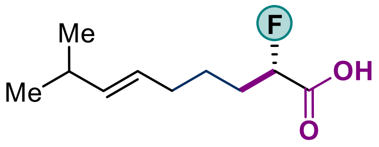

18
($CDCl_3$, ^1H NMR)

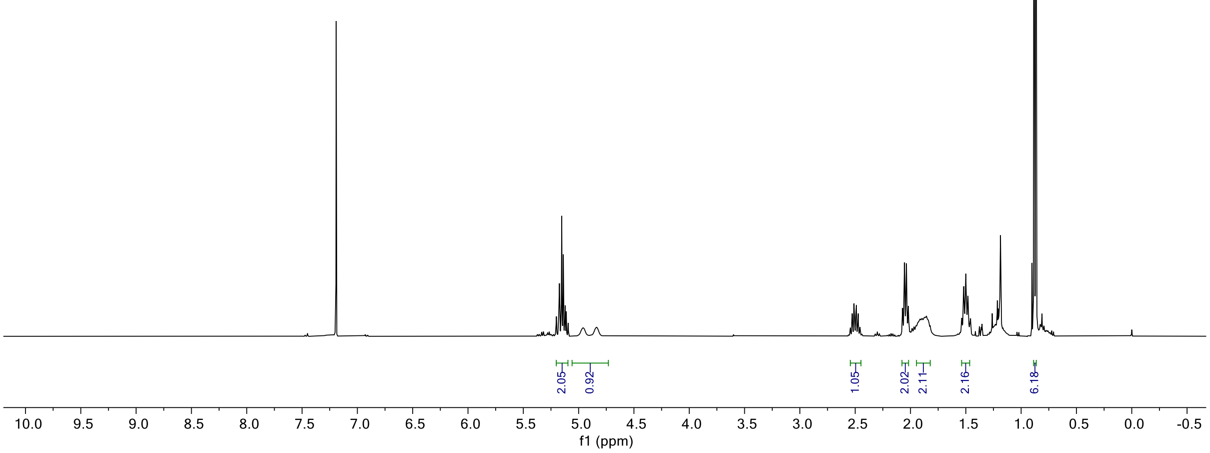

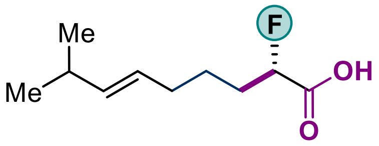

18
($CDCl_3$, ^{13}C NMR)

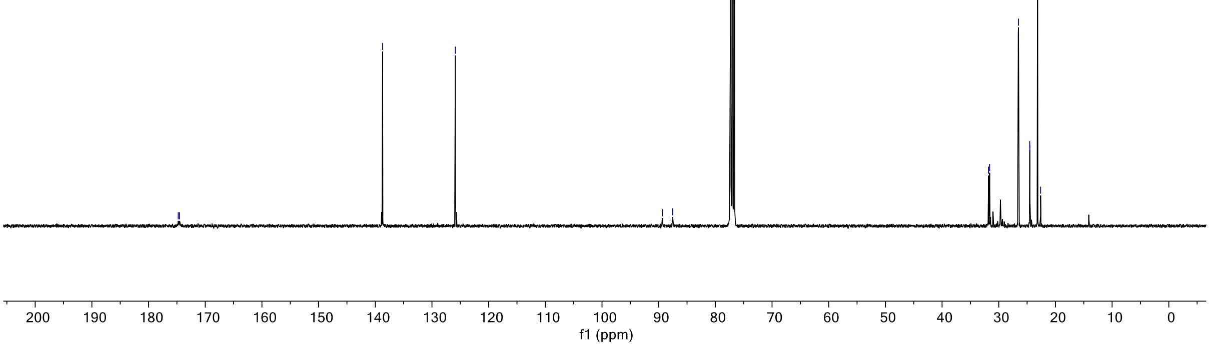

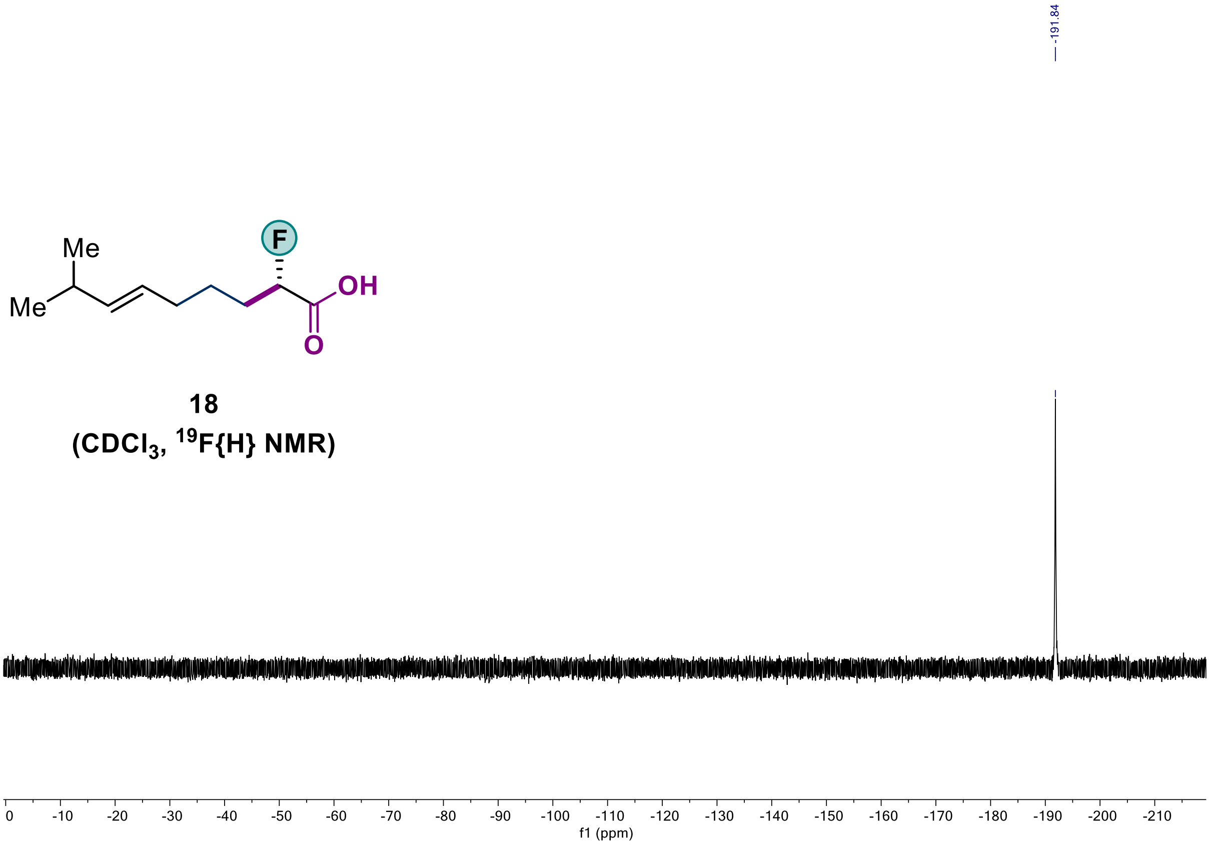

18
($CDCl_3$, $^{19}F\{H\}$ NMR)

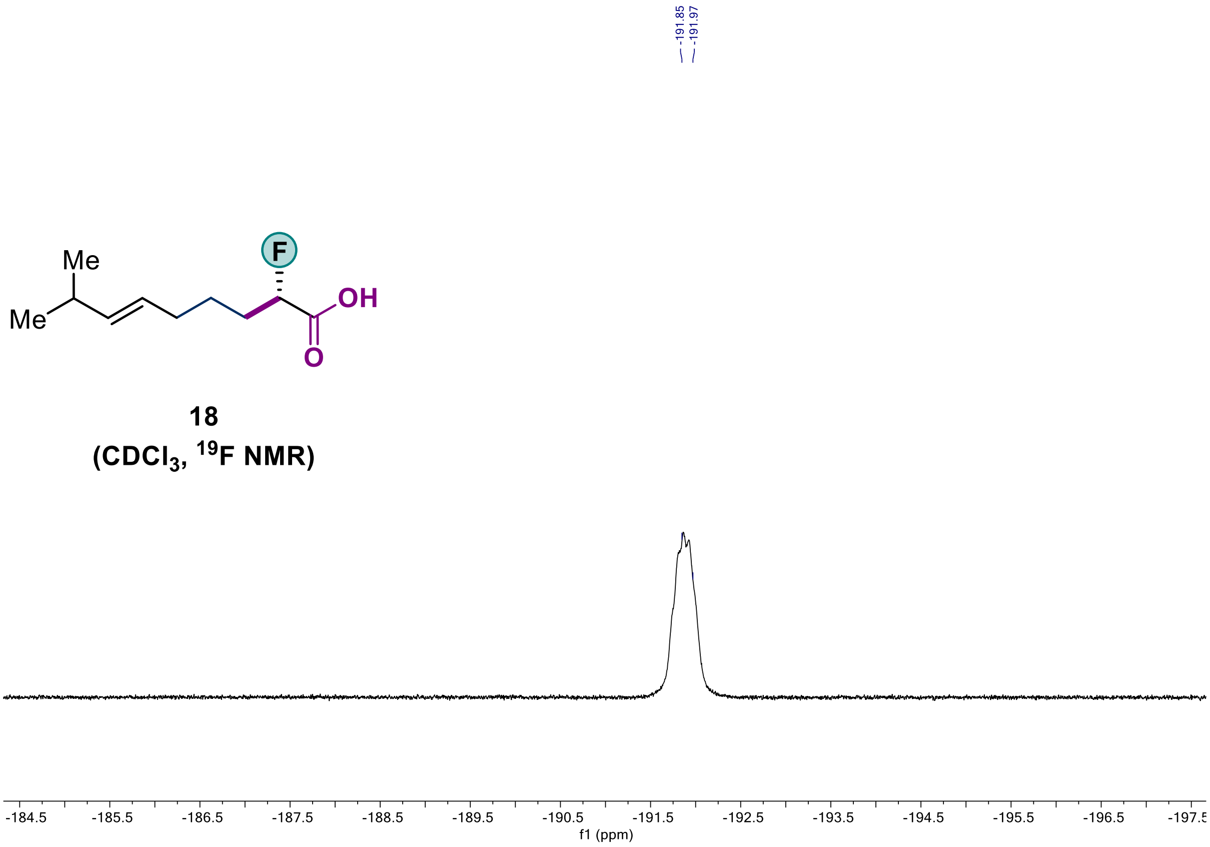

18
($CDCl_3$, ^{19}F NMR)

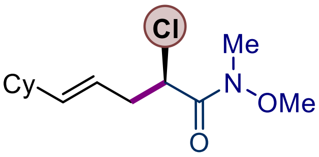

S2
($CDCl_3$, ^{1}H NMR)

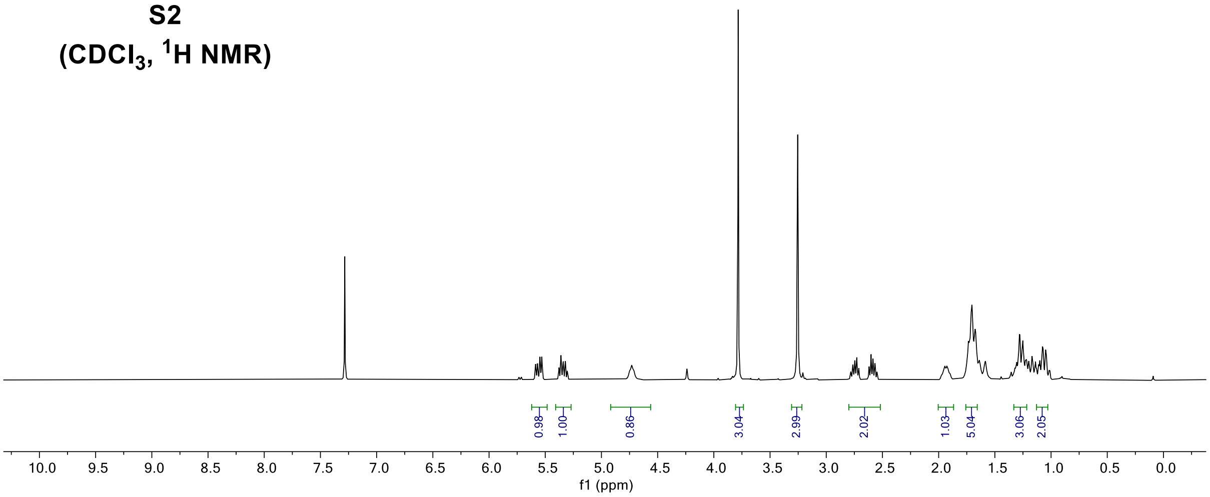

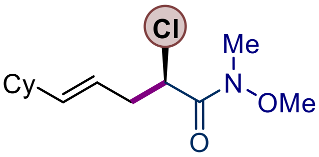

S2
($CDCl_3$, ^{13}C NMR)

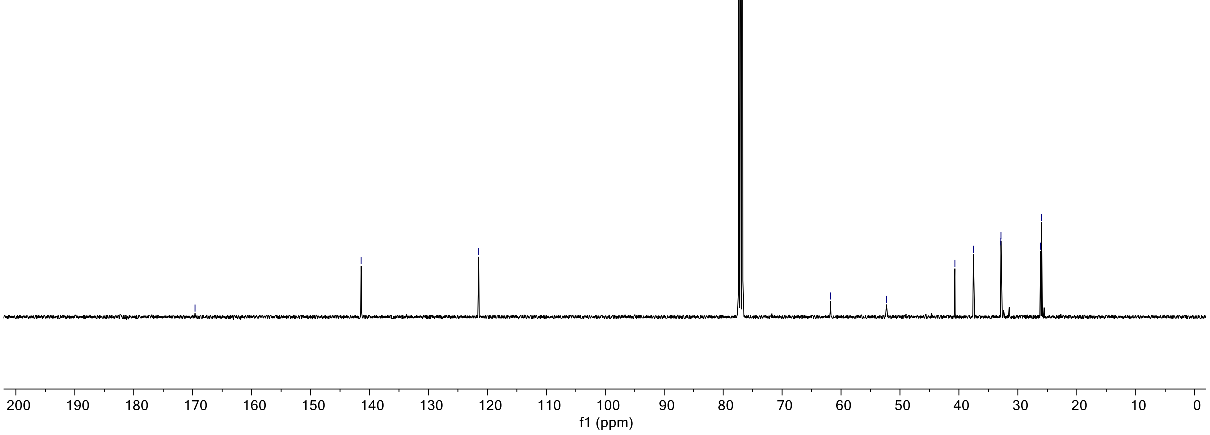

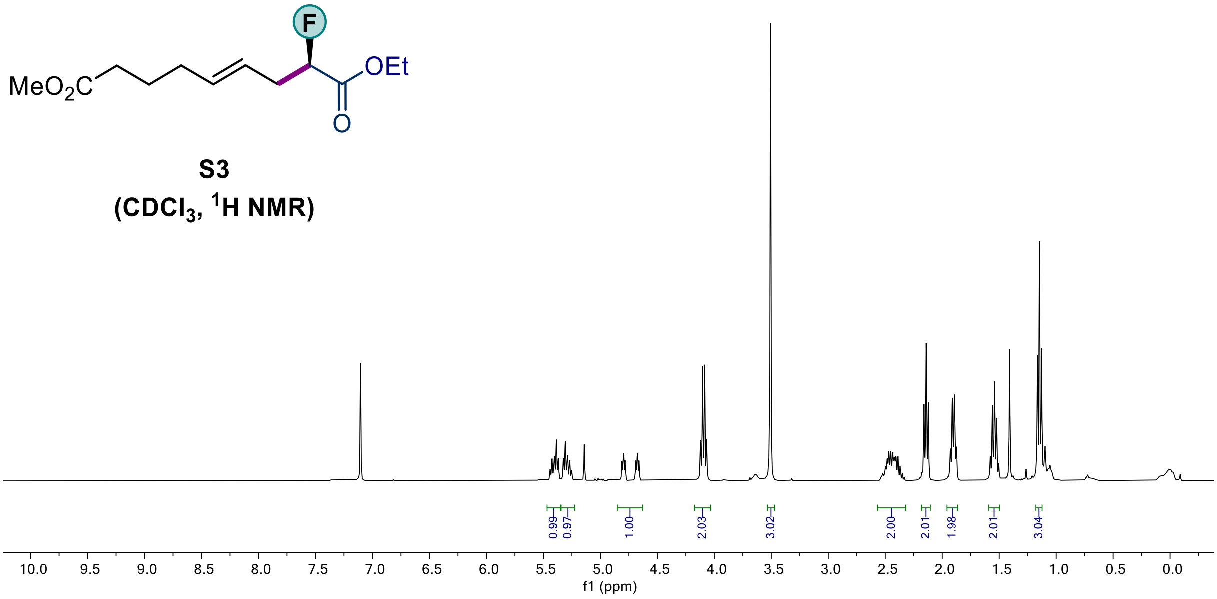

MeO2C
F
OEt
O
S3
(CDCl3, 1H NMR)
0.99
0.97
1.00
2.03
3.02
2.00
2.01
1.98
2.01
3.04
10.0
9.5
9.0
8.5
8.0
7.5
7.0
6.5
6.0
5.5
5.0
4.5
4.0
3.5
3.0
2.5
2.0
1.5
1.0
0.5
0.0
f1 (ppm)

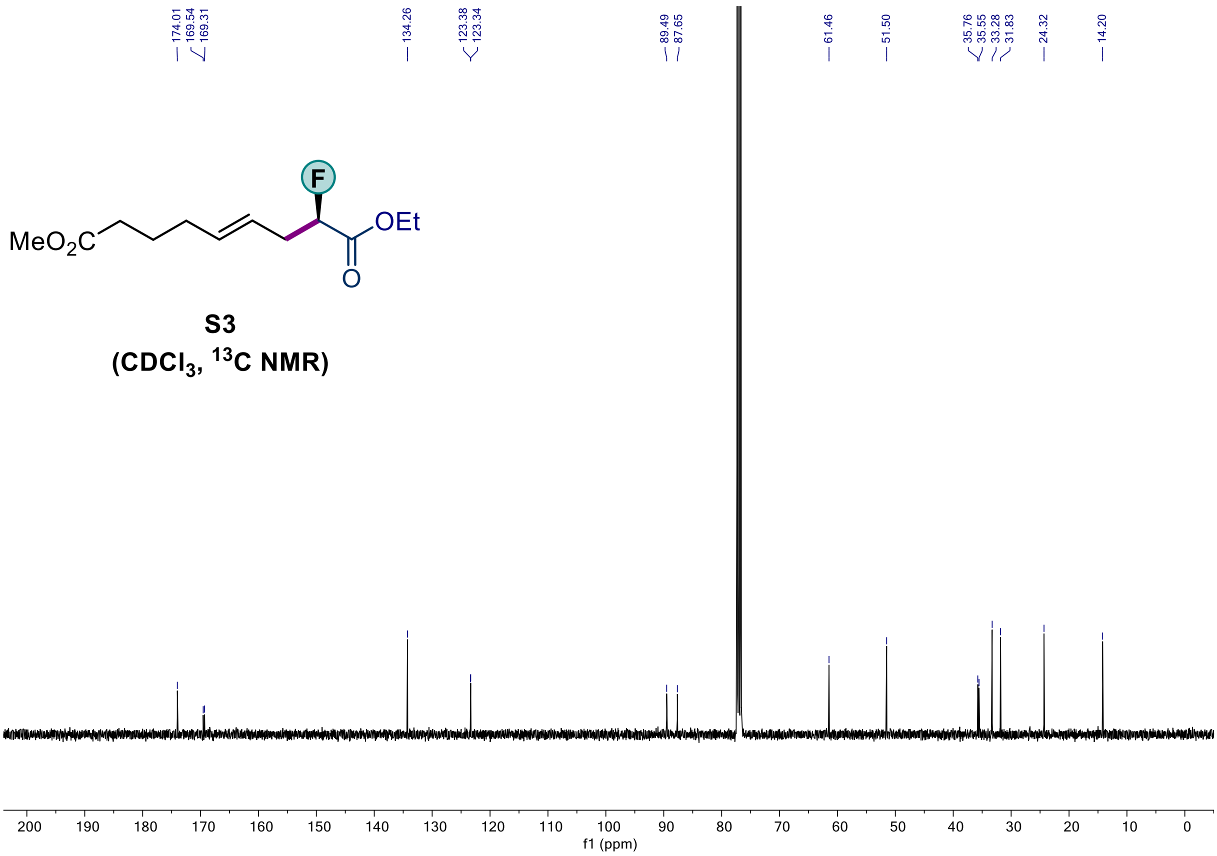

174.01
169.54
169.31
134.26
123.38
123.34
89.49
87.65
61.46
51.50
35.76
35.55
33.28
31.83
24.32
14.20
MeO2C
F
OEt
O
S3
(CDCl3, 13C NMR)
200
190
180
170
160
150
140
130
120
110
100
90
80
70
60
50
40
30
20
10
0
f1 (ppm)

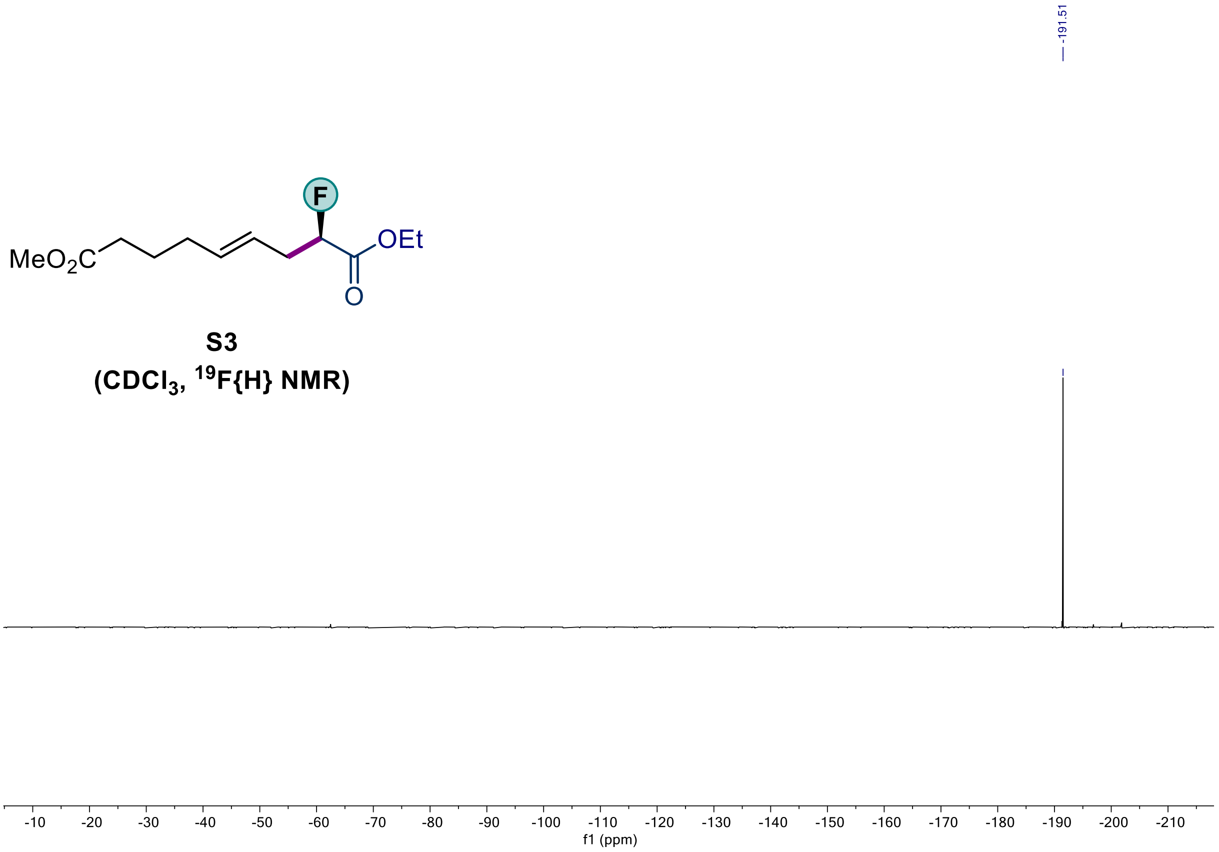
-191.51
F
MeO2C
OEt
O
S3
(CDCl3, 19F{H} NMR)
-10 -20 -30 -40 -50 -60 -70 -80 -90 -100 -110 -120 -130 -140 -150 -160 -170 -180 -190 -200 -210
f1 (ppm)

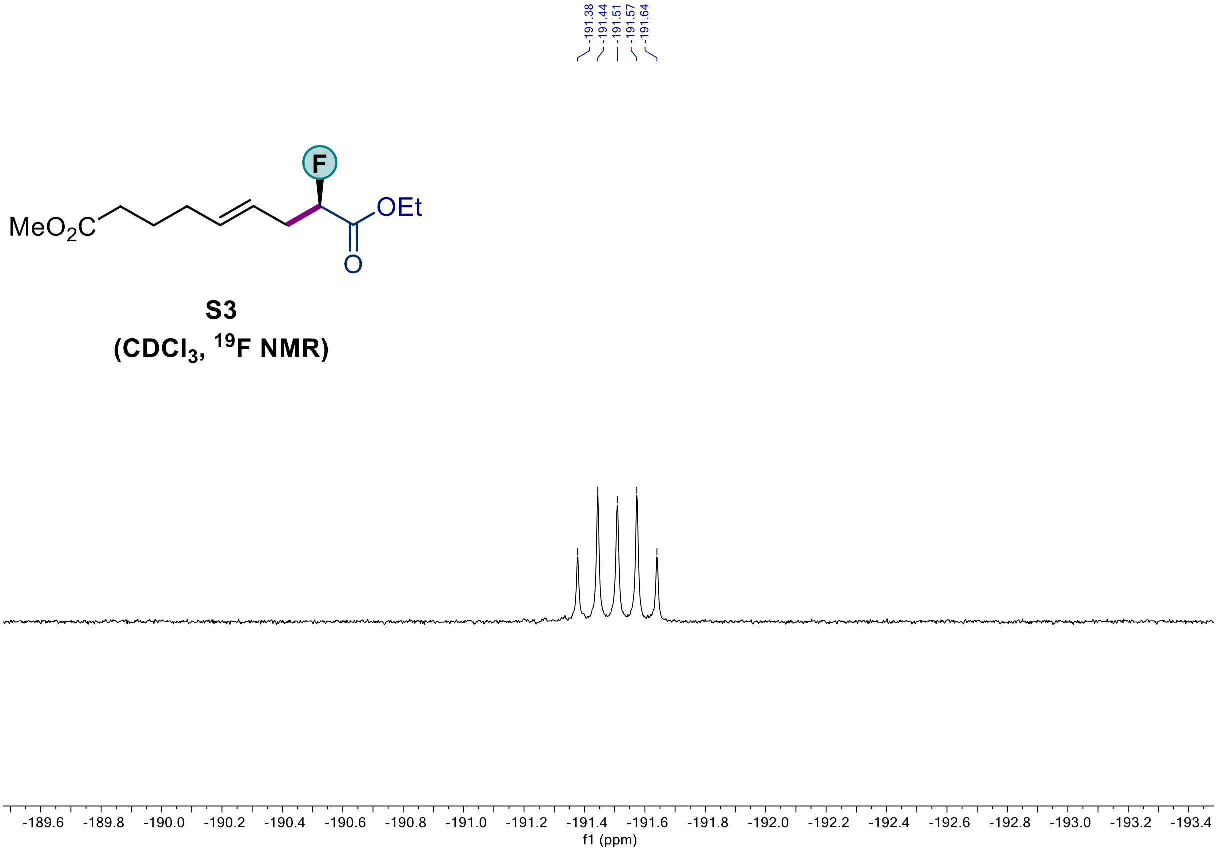
-191.38
-191.44
-191.51
-191.57
-191.64
F
MeO2C
OEt
O
S3
(CDCl3, 19F NMR)
-189.6 -189.8 -190.0 -190.2 -190.4 -190.6 -190.8 -191.0 -191.2 -191.4 -191.6 -191.8 -192.0 -192.2 -192.4 -192.6 -192.8 -193.0 -193.2 -193.4
f1 (ppm)

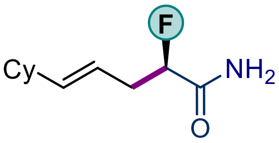

S9
(CDCl$_3$, ^{1}H NMR)

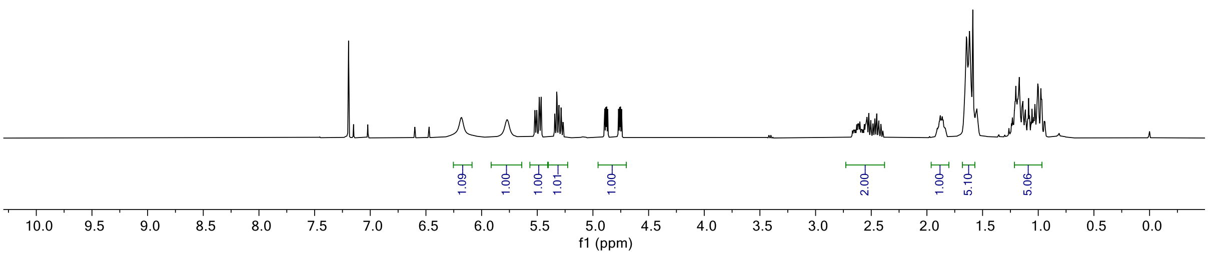

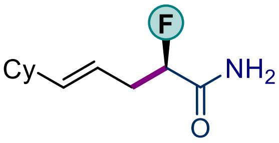

S9
(CDCl$_3$, ^{13}C NMR)

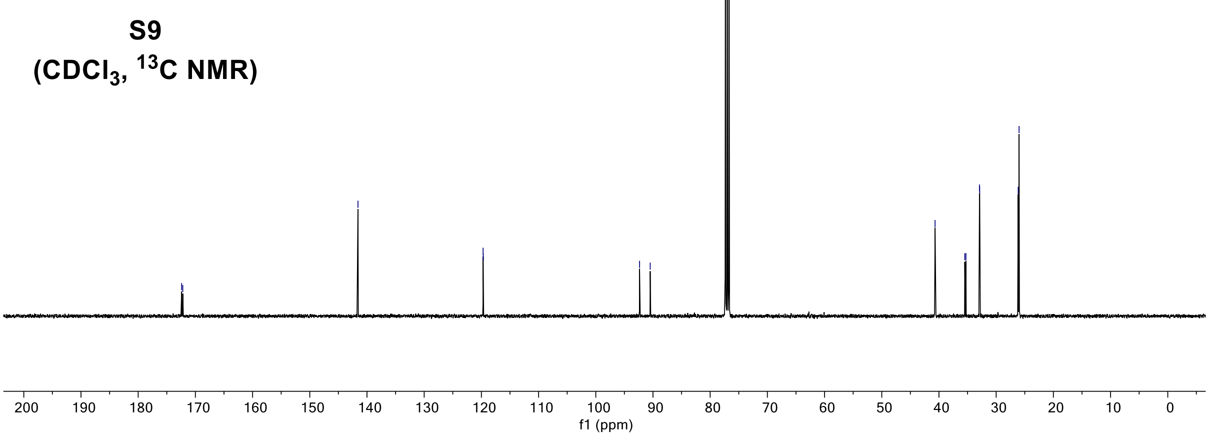

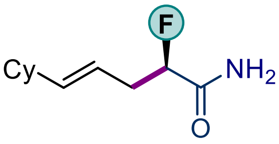

S9
($CDCl_3$, $^{19}F\{H\}$ NMR)

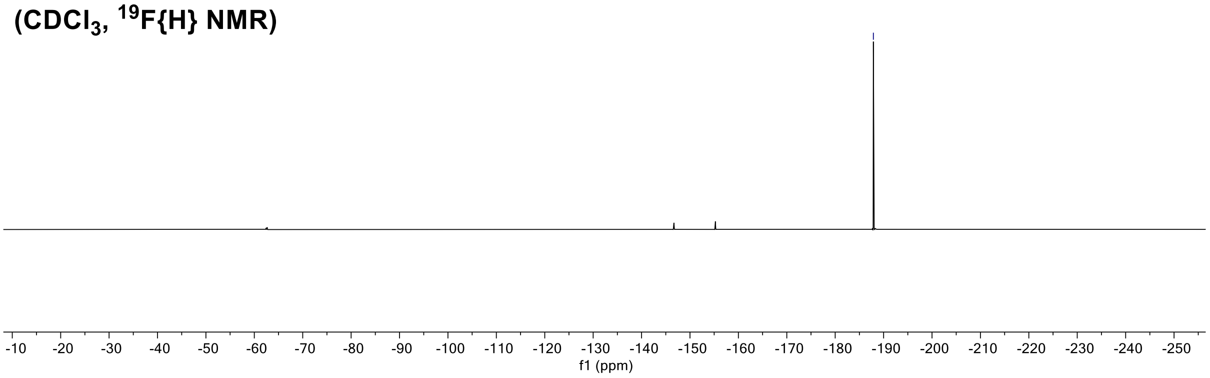

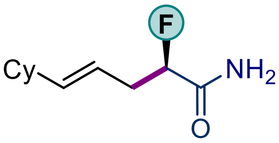

S9
($CDCl_3$, ^{19}F NMR)

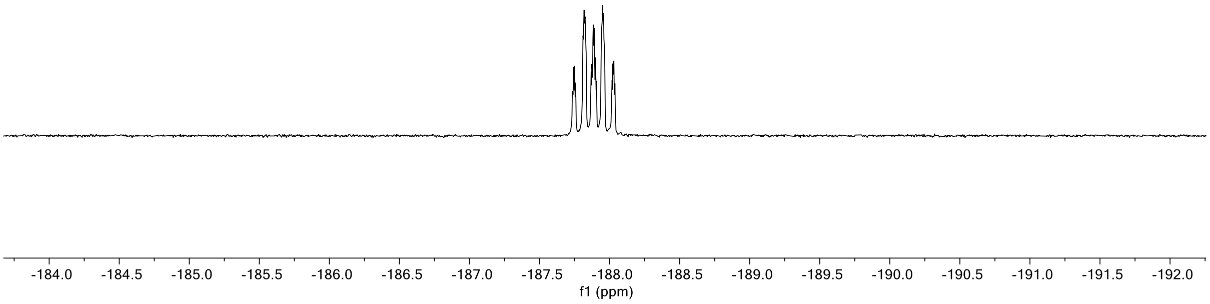

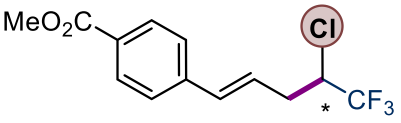

S10
($CDCl_3$, ^{1}H NMR)

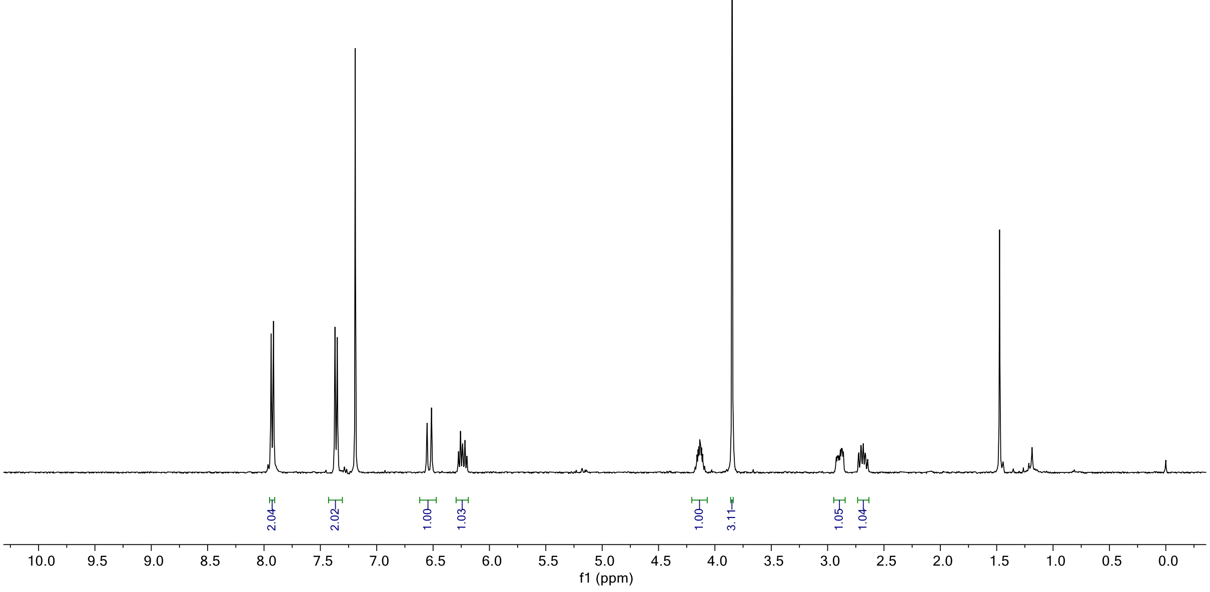

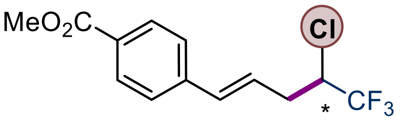

S10
($CDCl_3$, ^{13}C NMR)

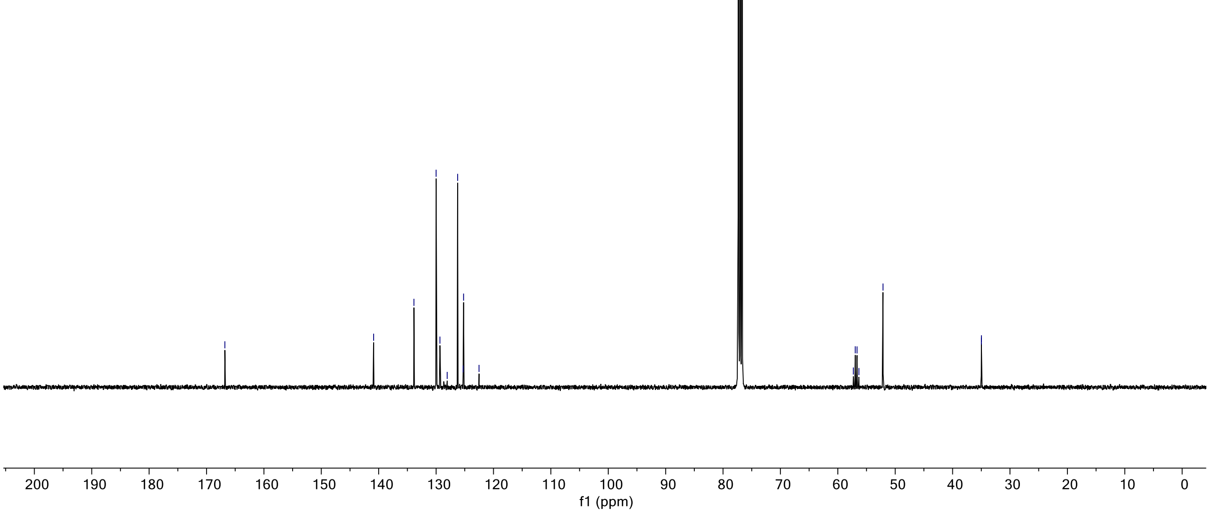

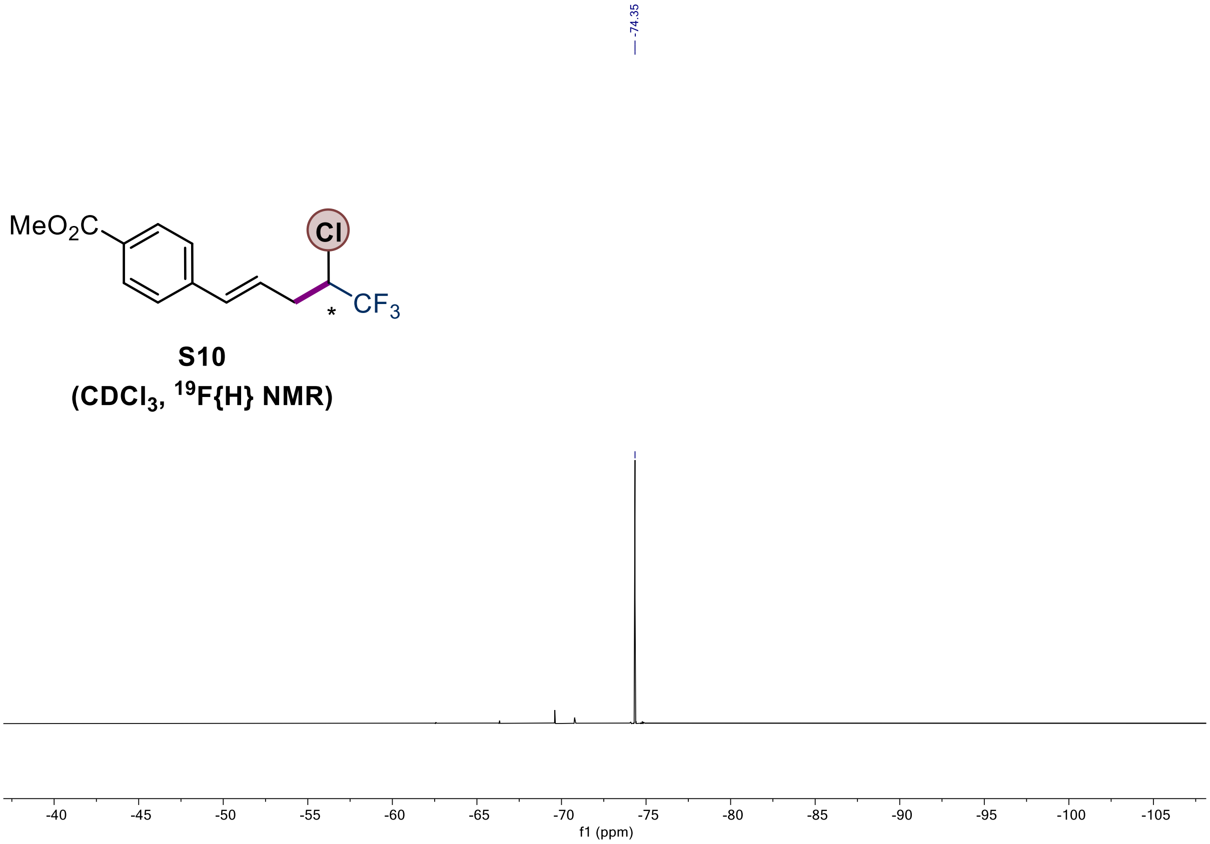
-74.35
MeO2C
Cl
CF3
S10
(CDCl3, 19F{H} NMR)
-40
-45
-50
-55
-60
-65
-70
-75
-80
-85
-90
-95
-100
-105
f1 (ppm)

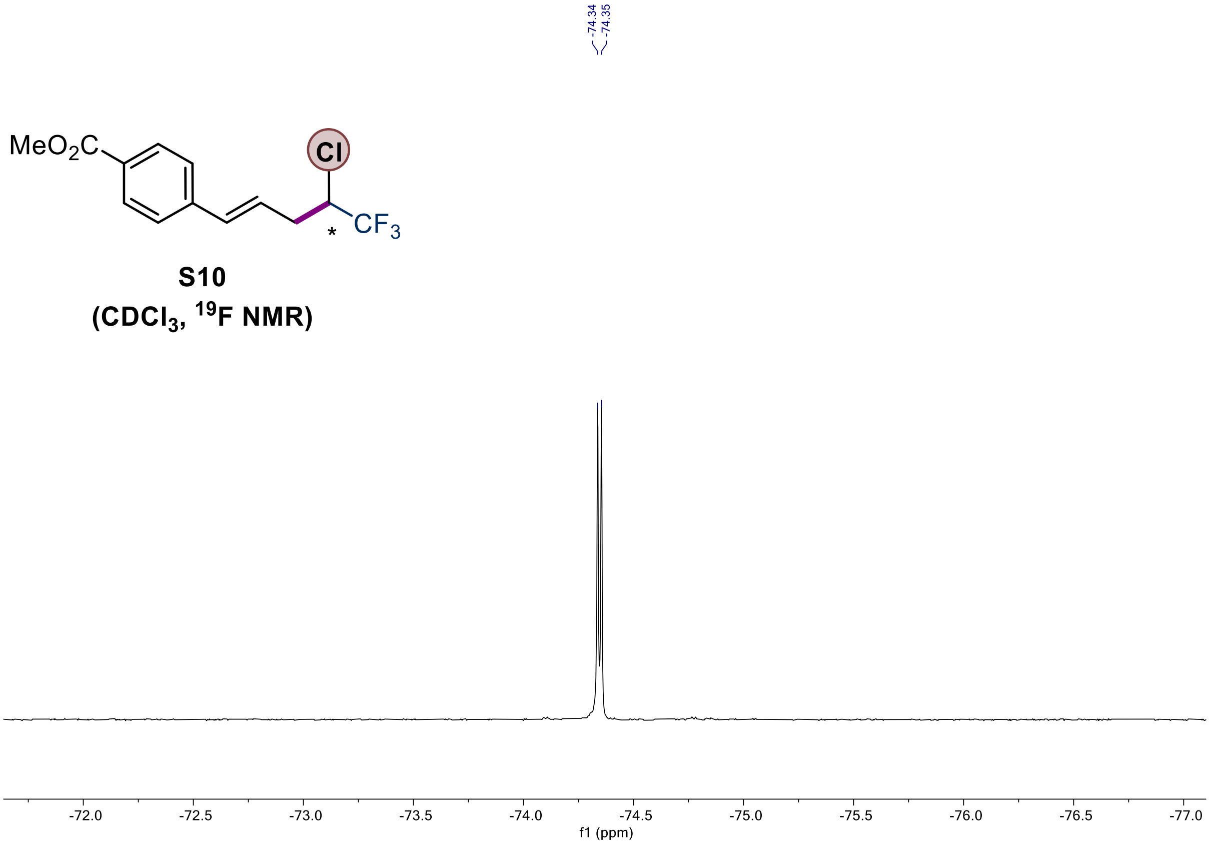
-74.34
-74.35
MeO2C
Cl
CF3
S10
(CDCl3, 19F NMR)
-72.0
-72.5
-73.0
-73.5
-74.0
-74.5
-75.0
-75.5
-76.0
-76.5
-77.0
f1 (ppm)

10. References

[12] F. Chen, Q. Zhang, Y. Li, Z.-X. Yu, L. Chu, *J. Am. Chem. Soc.* **2024**, *146*, 11418–11431.

[36] F. Liang, N. Chen, K. Cheng, Q. Wang, *Org. Lett.* **2023**, *25*, 8168–8172.

[37] D. Yamane, R. Tetsukawa, N. Zenmyo, K. Tabata, Y. Yoshida, N. Matsunaga, N. Shindo, A. Ojida, *J. Med. Chem.* **2023**, *66*, 9130–9146.

[38] U. Dhawa, L. Lavrencic, X. Hu, *ACS Cent. Sci.* **2024**, *10*, 1657–1666.

[52] Y. Wang, S. G. Scrivener, X.-D. Zuo, R. Wang, P. N. Palermo, E. Murphy, A. C. Durham, Y.-M. Wang, *J. Am. Chem. Soc.* **2021**, *143*, 14998–15004.

[53] C.-Y. Chang, A. Aponick, *J. Am. Chem. Soc.* **2024**, *146*, 16996–17002.

[54] C. Li, B. Breit, *J. Am. Chem. Soc.* **2014**, *136*, 862–865.

[55] X. Yang, G. C. Tsui, *Org. Lett.* **2020**, *22*, 4562–4567.

[56] Q. Gao, W.-C. Xu, X. Nie, K.-J. Bian, H.-R. Yuan, W. Zhang, B.-B. Wu, X.-S. Wang, *Nat. Commun.* **2024**, *15*, 6556.

[57] R. Wang, S. An, Y.-X. Xin, Y.-Y. Jiang, W. H. Liu, *JACS Au* **2024**, *4*, 4435–4444.

[58] Y. Li, D. Liu, L. Wan, J.-Y. Zhang, X. Lu, Y. Fu, *J. Am. Chem. Soc.* **2022***, 144,* 13961–13972.
